# Design rules for tuning charge-transfer emission in donor–acceptor nanohoops

**DOI:** 10.1039/d6sc03959f

**Published:** 2026-07-16

**Authors:** Gabriela M. Bailey, Ethan Q. Nguyen, Melanie A. Sheldon, Lev N. Zakharov, Ramesh Jasti

**Affiliations:** a Department of Chemistry and Biochemistry, Materials Science Institute, and Knight Campus for Accelerating Scientific Impact, University of Oregon Eugene Oregon 97403 USA rjasti@uoregon.edu; b CAMCOR – Center for Advanced Materials Characterization in Oregon, University of Oregon Eugene Oregon 97403 USA

## Abstract

Donor–acceptor (D–A) nanohoops provide a unique platform for tuning emission in curved π-systems through modulation of the frontier molecular orbitals as well as through charge-transfer (CT) interactions. Herein, we report a modular, building-block synthetic strategy enabling a systematic structure–property study of D–A nanohoops incorporating the electron acceptor benzothiadiazole (BT) and the electron donor thiophene (thio). Independent variations of ring size, D–A connectivity, and donor incorporation afford predictable tuning of the fluorescent emission across the visible spectrum, large effective Stokes shifts, and pronounced solvatofluorochromism. Combined experimental and computational analysis establishes design rules for fluorescent emission, demonstrating that increased acceptor strain, direct D–A connectivity, and increased content of electron-modulating units lead to bathochromic shifts in emission, enabling access to highly red-shifted emission as exemplified by compound 7. StrainViz calculations reveal that five-membered donor units redistribute strain across the nanohoop scaffold, influencing molecular geometry, while the observed photophysical trends are primarily governed by D–A charge-transfer interactions. Together, these findings position D–A nanohoops as a predictive platform for engineering curved π-systems with a range of different photophysical properties.

## Abstract 2

Donor–acceptor (D–A) nanohoops provide a unique platform for tuning emission in curved π-systems through modulation of the frontier molecular orbitals as well as through charge-transfer (CT) interactions.

## Graphical abstract

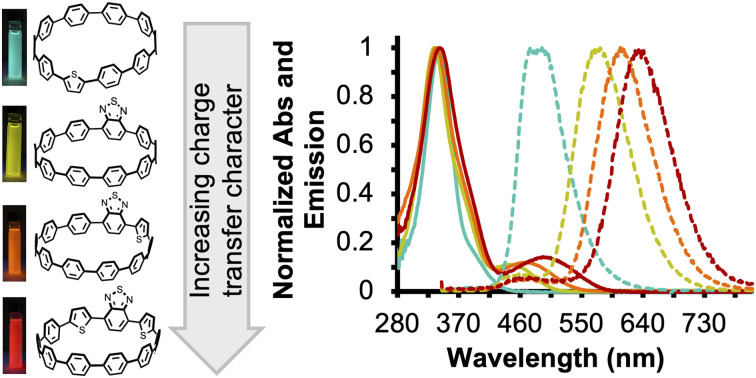

## Introduction

Incorporating electron donor (D) and electron acceptor (A) units into organic molecules with linear, planar π-extended conjugation is an active area of research central to the development of donor–acceptor (D–A) conjugated oligomers and polymers. Such systems underpin a wide range of applications in organic electronics and photonics, where control over frontier molecular orbitals, emission properties, and charge-transfer (CT) character is essential.^1–10^ Charge-transfer interactions in D–A systems are particularly important, as they enable precise control over excited-state polarization, emission energy, and charge separation processes. These properties are central to technologies ranging from organic photovoltaics to light-emitting devices and environment-responsive fluorophores, highlighting the importance of developing design principles that govern CT behavior. Emerging work has demonstrated embedding donors and acceptor motifs into curved π-systems offers additional opportunities to modulate electronic structure by molecular strain and geometry.^11–14^

A class of curved π-systems of particular interest is the family of nanohoops known as [*n*]cycloparaphenylenes ([*n*]CPPs). These nanohoops confine π-conjugation within a cyclic framework, resulting in radially oriented π-systems, extended conjugation, and an internal cavity capable of supramolecular interactions.^15^ Importantly, the inherent curvature and strain of [*n*]CPPs provide a unique platform for probing how geometric constraints influence electronic and photophysical properties. Previous studies have shown that incorporating donor and/or acceptor units into [*n*]CPP scaffolds can redistribute frontier molecular orbitals, shift absorption and emission maxima, modulate quantum yields and molar absorptivities, impart CT character, and tune singlet-triplet energy gaps.^16–25^ These features have motivated interest in D–A nanohoops for applications ranging from organic light-emitting diodes (OLEDs) to functional fluorophores for biological and materials contexts.

**Fig. 1:**
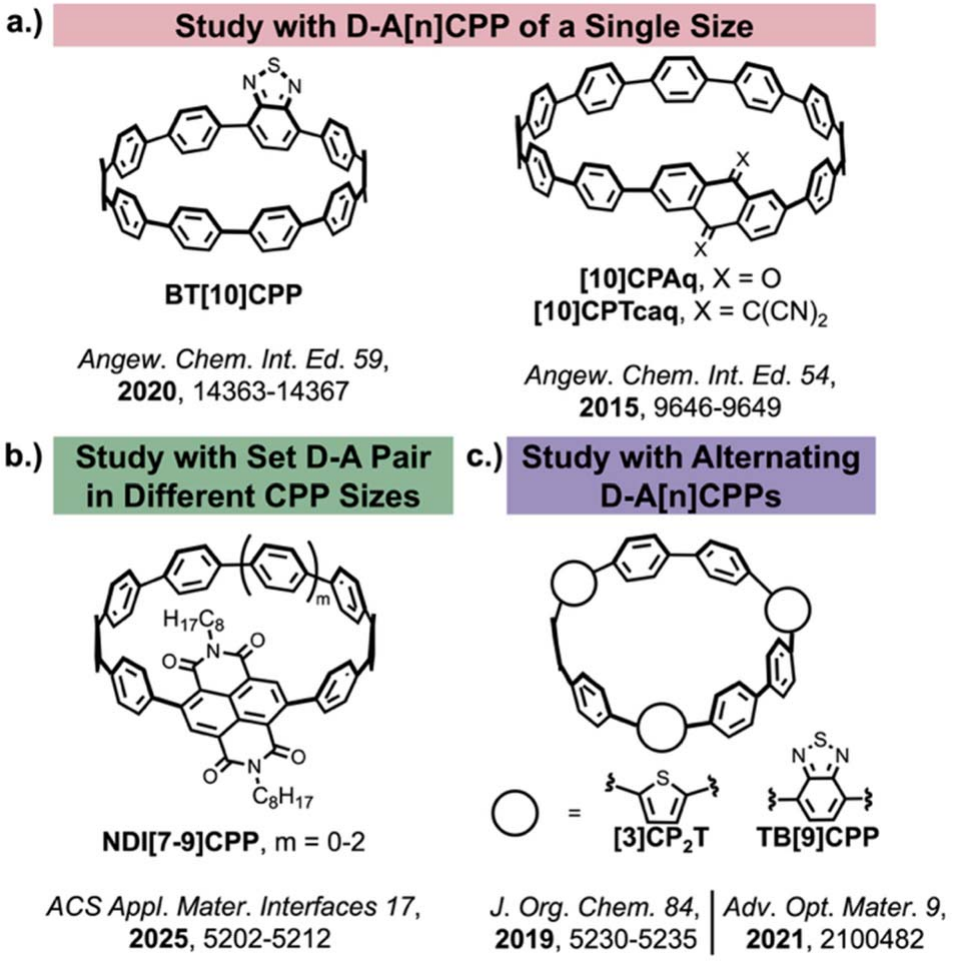
Previous literature examples of D–A[*n*]CPPs. (a) Changing the acceptor in a D–A[*n*]CPP of a fixed size. (b) Changing the acceptor in a series of different D–A[*n*]CPP sizes. (c) Examples of alternating D[*n*]CPPs or D–A[*n*]CPPs.

Despite this progress, most reported D–A nanohoop systems have focused on a limited subset of structural variations. Prior work has largely examined D–A nanohoops of a single size and only incorporated donor units intrinsic to the phenylene backbone (Fig. 1a),^26,27^ or kept the identity of the acceptor constant while varying the size of the nanohoop framework (Fig. 1b).^28^ Alternating D–A nanohoops have also been reported, including examples featuring heterocyclic donors such as thiophene (thio) or selenophene (Fig. 1c);^29,30^ however, systematic investigations that independently vary key structural parameters – such as D–A placement, donor identity, and nanohoop size – remain scarce. As a result, general design principles governing the photophysics in D–A nanohoops are not yet well established. Addressing this challenge requires access to a well-defined library of D–A nanohoops in which precise structural modifications can be introduced in a controlled manner, motivating the use of a modular, building-block synthetic approach capable of enabling direct structure–property comparisons.

Herein, we report a systematic structure–property study of seven new D–A nanohoops – along with two previously reported compounds as controls.^17,26^ A series of thio- and benzothiadiazole-containing (BT-containing) nanohoops was designed to probe three key variables: (1) the effect of strain on D–A nanohoop photophysics, (2) the influence of donor–acceptor placement and connectivity, and (3) the impact of increasing the number of electron modulating units. Through combined experimental and computational analysis, we demonstrate that these parameters can be tuned in a predictable manner to control emission energy and charge-transfer character. These results establish general design principles linking molecular structure, strain, and D–A interactions, providing a framework for the rational design of emissive curved π-systems.

## Results and discussion

### Design and synthesis of substrates

#### Design of D–A nanohoop series

The design of the D–A nanohoop series was guided by the goal of systematically probing how strain, donor–acceptor connectivity, and heterocycle incorporation influence photophysical properties. Using the all-hydrocarbon [*n*]CPP naming system as inspiration, we refer to the compounds using the general formula A-*x*-D[*n*]CPP, where *n* corresponds to the total number of cyclic, aromatic rings present in the nanohoop, A denotes the electron acceptor BT (designated as cyclic, aromatic ring one); *x* is a number indicating which cyclic, aromatic ring is the donor; while D defines the donor, either thio or a *meta*-substituted phenylene unit (abbreviated as ‘*m*’). For example, compound 5 contains both BT and thiophene units, therefore A = BT and D = thio. Thiophene is the seventh cyclic, aromatic ring, resulting in *x* = 7. The nanohoop is twelve cyclic, aromatic rings large, designating compound 5 as BT-7-thio[12]CPP. While designing the series, predicted emission values (*λ*_em_ (calc)) were computed using Gaussian09 (ref. 31) by optimizing the target molecules and the control compounds using a 6-31G** basis set and a CAM-B3LYP functional with the self-consistent reaction field solvent model set to the dielectric constant of DCM or DMSO.

**Fig. 2:**
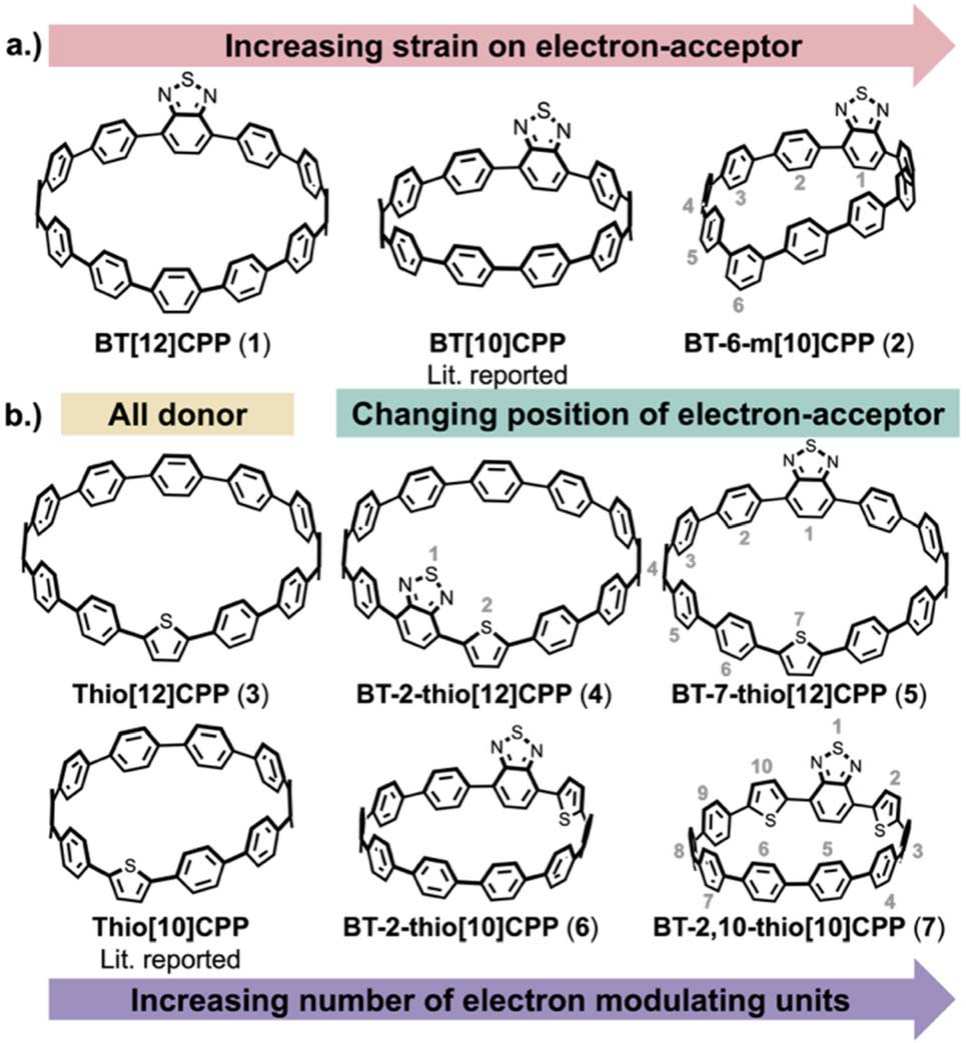
Depiction of properties under investigation in systematic series. (a) Compounds 1, BT[10]CPP, and 2 are organized from left to right by the strain induced on the acceptor. (b) Compounds 3, 4, 5, Thio[10]CPP, 6, and 7 are organized by property studied: all D[*n*]CPPs (3 and Thio[10]CPP), changing position of the acceptor in relation to the donor (4 and 5), or increasing the number of heterocycles (bottom row; left to right).

The rationale behind this systematic study is subsequently described and condensed in Fig. 2. To investigate the effect of strain on the acceptor, we first considered BT[10]CPP, which exhibits a 105 nm bathochromic shift in emission relative to [10]CPP (maximum emission (*λ*_em_) = 571 nm *vs.* 466 nm). Increasing the nanohoop size, as in compound 1, was expected to reduce strain and induce a hypsochromic shift in emission (*λ*_em_ (calc) = 571 nm). This value is 24 nm blue-shifted from the *λ*_em_ (calc) = 595 nm value calculated for BT[10]CPP using the computational basis set and functional described above. In contrast, having a *meta*-linked phenylene as the sixth cyclic, aromatic ring in BT-6-*m*[10]CPP (2) was anticipated to increase strain localized on the acceptor,^32^ leading to a bathochromic shift (*λ*_em_ (calc) = 617 nm). Embedding five-membered heterocycles such as thiophene can influence both electronic structure and molecular geometry. Literature precedence has readily shown incorporating thiophene into molecular scaffolds can influence the frontier molecular orbitals and therefore, the photophysical proper-ties. Also, the internal bond angle of thiophene (158°) differs significantly from that of *para*-substituted phenylenes (180°), where this angular mismatch has been computationally shown to redistribute strain across the nanohoop (see StrainViz Calculations). With this in mind, we selected thiophene as the donor unit and first examined its placement relative to the electron acceptor. Control compound 3, containing a single thiophene unit (*λ*_em_ (calc) = 479 nm), was predicted to exhibit only a modest bathochromic shift compared to [12]CPP (*λ*_em_ = 450 nm), suggesting that donor incorporation alone is insufficient to induce large emission changes in the absence of an acceptor. Comparison of compounds 3, 4, and 5 enables evaluation of how donor–acceptor placement along the nanohoop framework influences emission. In compound 4, the donor and acceptor are directly connected through the conjugated framework, whereas in compound 5, the donor and acceptor are positioned farther apart around the nanohoop. Calculations predict a larger bathochromic shift for 4 (*λ*_em_ (calc) = 598 nm) than for 5 (*λ*_em_ (calc) = 584 nm), suggesting that direct donor–acceptor connectivity more effectively enhances CT character than a more spatially separated arrangement.

We further explored the effects of strain on the donor or a D–A pair by reducing nanohoop size. Comparison of compound 3 with previously reported Thio[10]CPP (*λ*_em_ (calc) = 495 nm), and of 4 and 6 (*λ*_em_ (calc) = 606 nm), demonstrates that decreasing ring size leads to bathochromic shifts in emission relative to their larger counterparts. In the preparation of compounds 4 and 6, the intermediate necessary to synthesize compound 7 (*λ*_em_ (calc) = 653 nm) was readily isolated, enabling investigation of the effect of increasing the number of heterocycles within the nanohoop. This progression allows evaluation of how multiple electron-modulating units influence charge-transfer behavior and emission properties, completing the systematic exploration of structural variables in this D–A nanohoop series.

#### Synthesis of D–A nanohoop series

**Fig. 3:**
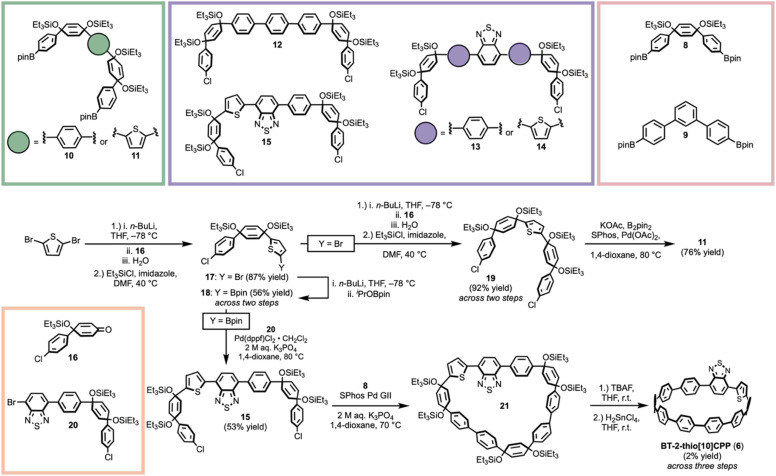
Eight key cross-coupling partners used to synthesize macrocyclic precursors necessary for the preparation of target D–A[*n*]CPPs (top). Synthesis of a few select intermediates (17, 18, 19, 11, and 15) and target compound 6 – BT-2-thio[10]CPP (bottom).

Synthesizing the target library focused on preparing eight key intermediates (*i.e.*, coupling partners; Fig. 3 (top)) that could be combined *via* Suzuki cross-coupling reactions to yield the desired macrocyclic intermediates. These eight intermediates fall into three-size classes: (1) three-ring, (2) five-ring, and (3) seven-ring building blocks. The identity of each cyclic unit includes phenylene (connected at the 1,4-*para* or 1,3-*meta* positions), cyclohexadiene rings acting as “masked benzenes” (where sp^3^-hybridized carbons impart the curvature necessary for macrocyclization), or heterocycles such as thio (2,5-linked) and BT (4,7-linked). Formation of the D–A[12]CPPs was achieved through coupling of five- and seven-ring intermediates, while D–A[10]CPPs were obtained from coupling three- and seven-ring coupling partners.

Intermediates 8, 9, and 10 were prepared *via* literature procedures.^33,34^ The synthesis of intermediates 11, 12, and 15 are outlined in Fig. 3 (bottom). Lithium–halogen exchange of 2,5-dibromothiophene followed by nucleophilic addition into electrophile 16 afforded the corresponding alcohol, which was protected using triethylsilyl chloride (Et_3_SiCl) in DMF to give intermediate 17. Subsequent lithium–halogen exchange at the bromide of 17 enabled formation of a nucleophile that could undergo addition into either ^*i*^PrOBpin to afford intermediate 18, or into electrophile 16 to generate an asymmetric five-ring intermediate. The resulting asymmetric five-ring intermediate with the free alcohol was again protected using Et_3_SiCl (intermediate 19), and the chlorides were converted to boronate esters *via* Miyaura borylation to afford the desired cross-coupling partner 11. Alternatively, intermediate 18 underwent Suzuki cross-coupling with intermediate 20 (Scheme S10) to yield cross-coupling partner 15. Intermediates 12, 13 and 14 were prepared using analogous synthetic sequences (Schemes S7–S9).

With the eight key coupling partners in hand, macrocyclization reactions were conducted under dilute conditions using SPhos Pd GII or GIII pre-catalysts to afford our macrocyclic precursors. Two of the seven macrocycles were readily purified by column chromatography on alumina. The remaining five macrocycles proved more challenging to purify; repeated column chromatography, size-exclusion chromatography, and/or trituration yielded only small amounts of clean material (<5 mg). In these cases, partially purified material was carried forward to subsequent steps without further purification. Both pure and partially purified macrocycles were subjected to silyl ether deprotection using TBAF to yield the corresponding diols, followed by reductive aromatization using H_2_SnCl_4_ to afford the target molecule (example for 6 shown in Fig. 3 (bottom)). For specific macrocyclization and target molecule procedures see Schemes S11–S17 for full details. The seven target nanohoops (1–7) were obtained in yields ranging from 2–38% and were fully characterized by ^1^H NMR, ^13^C NMR, and HRMS.

**Fig. 4:**
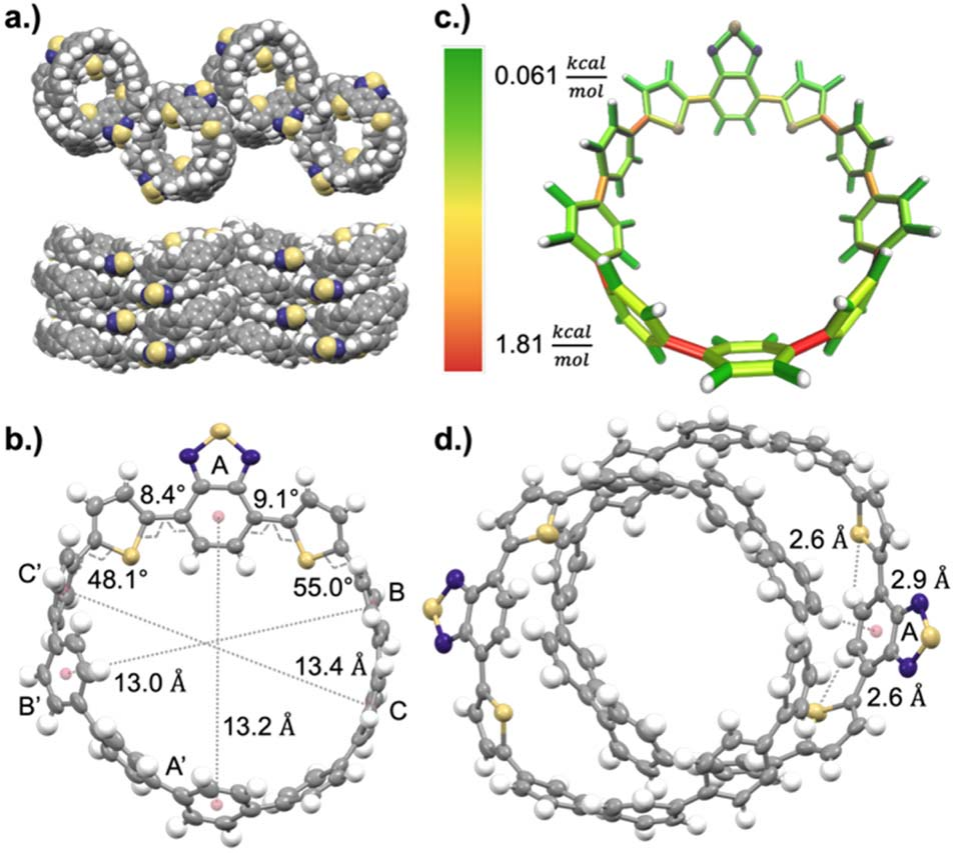
Crystal structure of compound 7. (a) Top and side view of space fill model of 7, highlighting tubular structure. (b) Diameter and torsional angle measurements of the ellipsoid plot drawings of 7. (c) StrainViz analysis of 7. (d) Intermolecular interactions between two molecules of 7 in their ellipsoid plot drawings.

Target compound 7 was further characterized by single-crystal X-ray diffraction. Suitable crystals were obtained by slow evaporation of DCM and diethyl ether at room temperature. Solvent molecules were highly disordered and were treated using SQUEEZE and are not included in the reported formula. The crystal packing of 7 exhibits a tubular arrangement, with the donor–acceptor–donor (D–A–D) motif aligning in an alternating fashion along the stacking axis (Fig. 4a). The nanohoop diameter was measured using centroid-to-centroid distances (Fig. 4b), giving values of approximately 13.2 Å (A–A′, centered on the BT unit), 13.0 Å (B–B′), and 13.4 Å (C–C′). Torsional angles between thiophene and adjacent phenylene units are 48.1° and 55.0°, values greater than the torsional angles of bonded phenylene units in [10]CPP (20° and 45°).^35^ The increase in torsional angle is likely attributed to the fact that the angle between the 2- and 5-positions of thiophene is smaller than the 180° angle of a *para*-substituted phenylene. The D–A torsional angles are significantly smaller (8.4° and 9.1°), indicating increased planarity at the donor–acceptor junction. The torsional angles suggest a redistribution of strain across the molecule, which we investigated *via* StrainViz analysis.^36^ The StrainViz analysis reveals that the D–A–D region is the least strained portion of the nanohoop, while most of the strain (1.81 kcal mol^−1^) is localized along the opposing backbone (Fig. 4c). Intermolecular interactions include S⋯H–C contacts (∼2.6 Å) and C– H⋯π interactions in which the C–H bonds of the electron acceptor unit engage with the π-system of a neighboring molecule (Fig. 4d).

#### Photophysical characterization

**Fig. 5:**
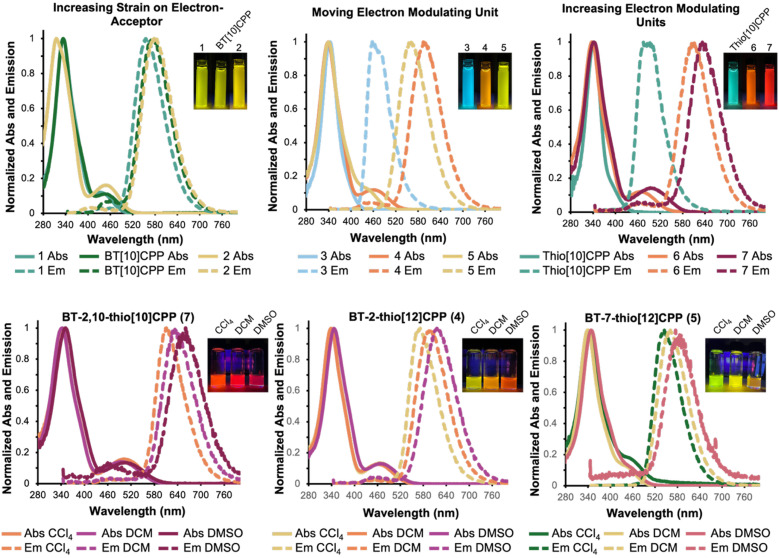
Photophysical data for 1–7. Scaled absorbance and emission profiles, in DCM, for 1–7 plotted in relation to the hypotheses depicted in Fig. 2 (top row). Scaled absorbance and emission profiles for 7, 4, and 5 in CCl_4_, DCM, and DMSO (bottom row).

With the seven target compounds in hand, we investigated their photophysical properties in solution and assigned theoretical transitions from TD-DFT calculations to the major and minor absorption and emission features in DCM (Fig. S49–S62). The absorption and emission profiles for 1–7 and the control compounds are shown in Fig. 5 (top row). Compounds 1 and 3–7 exhibit a major absorption peak where the absorbance maxima (max., *λ*_abs_) are in the range of 335–343 nm (Table 1), consistent with those of [10]CPP (338 nm) and [12]CPP (339 nm). In contrast, compound 2 displays a major absorption peak with a hypsochromic shifted *λ*_abs_ at 316 nm, consistent with previous observations for *m*[*n*]CPPs (*m*[10]CPP = 328 nm). The major absorptions are a result of transitions from the HOMO−2 → LUMO+2, HOMO−1 → LUMO+3, HOMO−1 → LUMO+1, HOMO−1 → LUMO, and HOMO → LUMO+1. Only for compound 3 is there a major contribution from the HOMO → LUMO transition (Fig. S53). All new compounds except 3 and 5 also exhibit a secondary, lower-energy absorption peak with *λ*_abs_ values ranging from 440–495 nm (Table 1). The minor absorptions are a result of transitions from the HOMO−3 → LUMO, HOMO−2 → LUMO, HOMO−1 → LUMO, and HOMO → LUMO. Compound 3 does not have a secondary, lower-energy absorption band, while 5 demonstrates a broad shoulder ranging from 418–500 nm. The secondary absorption bands are attributed to transitions with charge-transfer character. The absorbance profiles in DCM for the small molecule analogues (composed of three cyclic units) for 1, 2 and 7 have been previously reported in the literature.^26,37^ The *λ*_abs_ of the major absorbance peak for all small molecule analogues show a hypsochromic shift in comparison to the *λ*_abs_ of the minor absorbance peak of their D–A nanohoop counterparts (Table S11).

**Table 1:** Tabulated photophysical data for compounds 1–7 and control compounds BT[10]CPP and Thio[10]CPP^a^

Compound	*λ*_abs_ (nm)		*λ*_em_ (nm)		*λ*_em_ (calc) (nm)^d^	Quantum yield^e^, *Ø*		Effective Stokes shift^f^ (nm cm^−1^)
	Main^b^	Minor	Main^c^	Minor		DCM	DMSO	
1	335	440	558	451	571	0.34	0.22	223|11 939
2	316	454	583	415	617	0.26	0.19	267|14 493
3	343	—	469	—	479	0.26	0.29	126|7833
4	339	457	599	453	598	0.16	0.11	260|12 804
5	340	—	565	—	584	0.31	0.01	225|11 713
6	337	466	609	470	606	0.10	0.06	272|13 253
7	342	495	639	479	653	0.06	0.03	297|13 590
BT[10]CPP	334^g^	445^g^	571^g^		595	0.59^g^		237|12 423
Thio[10]CPP	341	—	490		495	0.17 (in CHCl_3_)^h^		152|9042

a Data obtained in DCM unless otherwise specified.

b Maximum absorbance.

c Maximum emission.

d All calculated maximum emissions were computed using the same basis set and functional.

e Quantum yield values in DCM and DMSO.

f Effective Stokes shifts (difference between the dominant absorbance maxima and the dominant emission maxima) in nm and cm^−1^.

g Values from citation 26.

h Values from citation 17.

The emission profiles for compounds 1–7 have a major, emission peak where the emission maximum (*λ*_em_) range between 469–639 nm (Table 1). The major emissions are a result of transitions from the LUMO → HOMO (Fig. S49–S62). All new compounds except 3 and 5 also show a minor emission peak with *λ*_em_ values ranging from 415–479 nm (Table 1), which is consistent with a higher-energy emission feature and may reflect weaker or less stabilized excited states. The minor emissions are a result of transitions from the HOMO−4 → LUMO, HOMO−3 → LUMO, HOMO−2 → LUMO, HOMO−1 → LUMO, HOMO → LUMO+1, and HOMO → LUMO+2. The emission profiles in DCM for the small molecule analogues for 1, 2, and 7 demonstrate a hypsochromic shift to the *λ*_em_ of the major emission peak when compared to the *λ*_em_ of the major emission peak of their D–A nanohoop counterparts.^26,37^ The emission profile in DMSO for the small molecule analogue (composed of two cyclic units) for 4 also demonstrates a hypsochromic shift to the *λ*_em_ of the major emission peak when compared to the *λ*_em_ of the major emission peak of the D–A nanohoop counterpart (Table S11).^38^

Comparison of the absorption and emission profiles of the small molecule analogues to their respective D–A nanohoop also suggests confining the motif to a curved and strained structure results in bathochromic shifts to the *λ*_abs_ and *λ*_em_. From the major emission peaks we observe increasing strain on the electron acceptor results in modest 12 nm and 13 nm bathochromic shifts to *λ*_em_ as we go from 1 to BT[10]CPP to 2. There is a 34 nm difference between the *λ*_em_ of 4 and 5, indicating donor and acceptor placement is enough to tune the *λ*_em_. Future work could investigate analogues in which thio is positioned as the third, fourth, fifth, or sixth cyclic ring within the BT-*x*-thio[12]CPP scaffold. We hypothesize that the *λ*_em_ for these theoretical compounds would fall between the emission maxima of 4 and 5, with the bathochromic shift becoming more pronounced as thio is positioned closer to BT. Lastly, by increasing heterocycle incorporation, we observe a 119 nm and a 30 nm bathochromic shift to *λ*_em_ as we go from Thio[10]CPP to 6 to 7. In summation, these data indicate that increasing strain on the electron acceptor, direct D–A connectivity, and increasing the number of electron-modulating units in the nanohoop scaffold results in the largest bathochromic shift in *λ*_em_. Notably, compound 7 exhibits emission at the red edge of reported donor–acceptor nanohoops in solution (*λ*_em_ = 639–662 nm), providing a clear demonstration that the combined effects of strain modulation, donor–acceptor coupling, and increased heterocycle incorporation can be leveraged to access low-energy CT states in a predictable manner. The major, max. emission values support the computationally derived trends.

To further probe the solvent-dependent effects, the quantum yield for all seven target molecules was measured in DCM (values ranging from 0.34 to 0.06, Table 1) and DMSO (values ranging from 0.22 to 0.01, Table 1). Aside from 3, which is not a D–A nanohoop, the quantum yields are all lower upon being measured in DMSO. For compound 3 the compound undergoes a 0.03 increase as the solvent is changed from DCM to DMSO, potentially suggesting DMSO decreases non-radiative decay pathways. Further investigation of these species is needed. Interestingly, when the acceptor and donor are positioned across the nanohoop (5), the drop in quantum yield upon moving to more polar solvents is the most prominent, suggesting a greater dipole. Overall, these data are further suggestive of CT behavior.

Therefore, the absorption and emission profiles for target compounds 7, 4, and 5 were measured in CCl_4_, DCM, and DMSO (Fig. 5, bottom row). These solvents were chosen to span a wide polarity range (*P*′ = 1.6–7.2) while maintaining partial or full solubility of the compounds. Compound 7 was selected as it exhibits the most red-shifted emission in DCM, and therefore its emission was expected to shift further in more polar solvents such as DMSO. Compounds 4 and 5 provide an informative comparison to evaluate whether D–A positioning influences sol-vatofluorochromic behavior. For all three compounds, a consistent trend is observed, where *λ*_em_ is most hypsochromic shifted in CCl_4_, while in DMSO the *λ*_em_ is the most bathochromic shifted. Specifically, *λ*_em_ values for 7 are 613 nm (CCl_4_), 639 nm (DCM), and 662 nm (DMSO); for 4, 575 nm (CCl_4_), 599 nm (DCM), and 622 nm (DMSO); and for 5, 550 nm (CCl_4_), 565 nm (DCM), and 587 nm (DMSO), respectively. These data plus the major and minor *λ*_abs_ can be found in Table S9. Compounds 4 and 7 each demonstrate approximately 24 nm shifts in *λ*_em_ as the solvent is changed. Compound 5 shows the most distinct jump, with a 15 nm change in *λ*_em_ going from CCl_4_ to DCM, while there is a 22 nm difference in *λ*_em_ between DCM and DMSO. The observed trend of the *λ*_em_ undergoing a bathochromic shift as we increase solvent polarity is observed for the small molecule analogues for 4 and 7.^37,38^ Overall, these findings support that the target compounds exhibit CT behavior.

Furthermore, the effective Stokes shifts (difference between the dominant absorbance maxima and the dominant emission maxima) for compounds 1–7 (in DCM, Table 1) were calculated. The effective Stokes shift values range from 126–297 nm or 7833–14,493 cm^−1^. Also, the solvatofluorochromism data can be further used in the Lippert–Mataga equation (eqn (1) in SI) to plot the effective Stokes shift (in wavenumbers) *versus* the orientation polarizability of the solvent (Figure S48 and Table S10). The slope of the linear fit describes the dipole moment variation (Δ*µ*), which is the difference between the excited state dipole and the ground state dipole. Therefore, a large slope indicates a larger dipole moment variation. Compound 4 has the largest dipole moment variation (Δ*µ* = 2970 cm^−1^), with compound 7 having the second largest dipole moment variation (Δ*µ* = 2510 cm^−1^), and compound 5 has the third largest dipole moment variation (Δ*µ* = 1810 cm^−1^). The data indicates compound 4 undergoes the largest dipole moment variation as the solvent increases in polarity, while compound 5 undergoes the least change in dipole moment variation.

#### StrainViz calculations

**Fig. 6:**
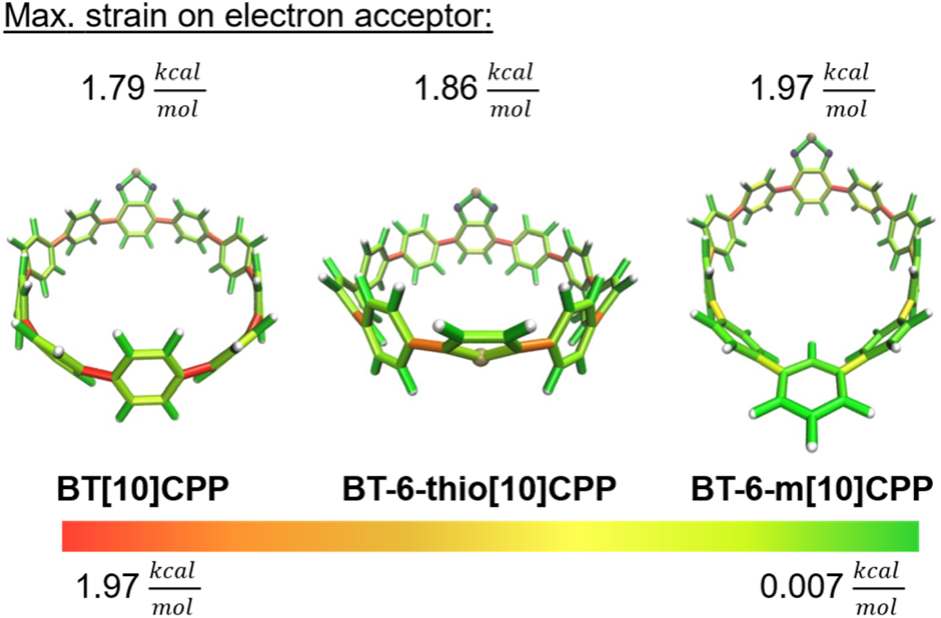
StrainViz analysis of BT[10]CPP, theoretical compound BT-6-thio[10]CPP, and compound 2 to determine the strain on the electron acceptor BT.

To further understand the structural origins of the observed photophysical trends, StrainViz calculations were performed on BT[10]CPP, the theoretical analogue BT-6-thio[10]CPP, and compound 2 (Fig. 6). (See SI for exact details on how structures were normalized to one another.) The inclusion of BT-6-thio[10]CPP enables direct comparison of heterocycle incorporation in the absence of additional structural modifications. In BT[10]CPP, strain is relatively evenly distributed across the nanohoop, with a maximum strain of 1.79 kcal mol^−1^. The theoretical analogue BT-6-thio[10]CPP shows a similar overall strain distribution, with only minor redistribution away from the thiophene-containing region and a maximum localized strain of 1.86 kcal mol^−1^. In contrast, compound 2 exhibits significant strain redistribution, with the highest strain localized on the side of the nanohoop opposite the *meta*-linkage, resulting in a maximum localized strain of 1.97 kcal mol^−1^ near the BT unit. These findings indicate that incorporation of *meta*-linked phenylenes has a more pronounced effect on global strain distribution than the incorporation of thiophene.

Overall, these results suggest that while heterocycle incorporation perturbs local geometry, the observed photophysical trends are more strongly influenced by donor–acceptor interactions and the resulting CT character than by global strain redistribution alone.

## Conclusion

In this work, we establish a set of design rules for tuning CT photophysics in donor–acceptor π-nanohoops through systematic control of molecular strain, donor–acceptor connectivity, and heterocycle incorporation. A modular, building-block synthetic strategy enabled the independent variation of these parameters within a single molecular platform, allowing direct structure–property relationships to be drawn across a family of well-defined donor–acceptor nanohoops incorporating benzothiadiazole acceptors and thiophene donors.

By comparing nanohoops of different sizes, connectivities, and donor counts, we demonstrate that increased strain localized on the electron acceptor leads to pronounced bathochromic shifts in emission, while direct D–A connectivity produces stronger CT character than spatially separated D–A arrangements. Increasing the number of donor heterocycles further amplifies these effects, yielding predictable, red-shifted emission trends and large relative Stokes shifts. Future studies may investigate the limits of heterocycle incorporation while also preserving the structures' radially oriented π-system and maintaining synthetic accessibility, as increasing the number of heterocycles reduces the number of cyclohexadiene units available to impart curvature. Therefore, alternative strategies for introducing structure curvature may be required. Importantly, these experimentally observed trends are supported by TD-DFT calculations, confirming that emission energies and charge-transfer behavior can be anticipated through molecular design. The ability to access highly red-shifted emission, as exemplified by compound 7, further highlights the predictive nature of these design principles. Photophysical measurements reveal broad tunability of emission across the visible spectrum, large relative Stokes shifts, and pronounced solvatofluorochromism, consistent with charge-transfer excited states. The observed solvent-dependent emission and reductions in quantum yield in polar media further support the role of donor–acceptor coupling and excited-state dipole formation. Single-crystal X-ray analysis of a representative D–A–D nanohoop reveals tubular packing and well-defined donor–acceptor geometries, while StrainViz calculations show that strain is preferentially localized away from the donor–acceptor motif, providing a structural basis for the observed photophysical behavior.

Collectively, these results position donor–acceptor nanohoops as a versatile and predictive platform for studying and engineering charge-transfer states in curved π-systems. The modular synthetic approach described here enables systematic exploration of this design space, providing a foundation for the rational design of organic emitters, environment-responsive fluorophores, and curvature-defined semiconducting materials. More broadly, this work highlights how molecular curvature and strain can be leveraged as complementary design elements in functional π-conjugated materials.

## Author contributions

G. M. B. and R. J. conceptualized the project. G. M. B., E. Q. N., and M. A. S. performed the organic chemistry and collected experimental data. G. M. B. performed the computational calculations. L. Z. solved the crystal structure. G. M. B., E. Q. N., M. A. S., and R. J. prepared the manuscript.

## Conflicts of interest

There are no conflicts to declare.

## Supplementary Material

SC-OLF-D6SC03959F-s001

SC-OLF-D6SC03959F-s002

## Data Availability

CCDC 2550070 contains the supplementary crystallographic data for this paper.^39^ The data supporting this article have been included as part of the supplementary information (SI). Supplementary information: general synthetic experimental details, characterization data (1H NMR, 13C NMR, FT-IR, HRMS), X-ray crystallographic data, theoretical and experimental photophysical data, and StrainViz computational data. See DOI: https://doi.org/10.1039/d6sc03959f.
