# Supplementary material for: Design rules for tuning charge-transfer emission in donor–acceptor nanohoops
Source: Chem Sci. 2026 Jul 16;17(34):16439–46. doi: 10.1039/d6sc03959f (PMC13373506; doi:10.1039/d6sc03959f)
Supplement: Supplementary file 1 [file SC-017-D6SC03959F-s001.pdf]

Supplementary Information (SI) for Chemical Science.
This journal is © The Royal Society of Chemistry 2026

# Supporting Information

## Design Rules for Tuning Charge-Transfer Emission in Donor-Acceptor Nanohoops

Gabriela M. Bailey[a], Ethan Q. Nguyen[a], Melanie A. Sheldon[a], Lev N. Zakharov[b], and Ramesh Jasti[a]*

[a] Department of Chemistry and Biochemistry, Materials Science Institute, and Knight Campus for Accelerating Scientific Impact, University of Oregon, Eugene, Oregon 97403, USA
[b] CAMCOR – Center for Advanced Materials Characterization in Oregon, University of Oregon, Eugene, Oregon 97403, USA

*Email: rjasti@uoregon.edu

# Table of Contents

**1. General Information** S1

**2. Synthesis** S2-24

- **a. Intermediate Synthesis**
  - *One and Two Ring Intermediates* S2-S3
  - *Three Ring Containing Intermediates* S3-S4
  - *Five Ring Containing Intermediates* S5-S9
  - *Seven Ring Containing Intermediates* S10-S14
- **b. D-A[12]CPP Derivatives** S15-S20
- **c. D-A[10]CPP Derivatives** S21-S24

**3. Nuclear Magnetic Resonance Spectra** S25-44

**4. Experimental and Computational Photophysical Characterization** S45-S66

- **a. Experimental Photophysical Characterization** S45-S49
  - *Quantum Yield Data* S45-S48
  - *Solvatofluorochromism Data* S49
- **b. Data for Literature Reported Small Molecule, Linear Analogues** S50
- **c. Computational Photophysical Characterization** S51-66
  - *Predicted Absorbance and Emission Values* S51-65
  - *Molecular Orbitals (MOs)* S66

**5. StrainViz Calculations** S67-69

**6. X-Ray Crystallography** S70

**7. References** S71

## 1. General Information

**Synthesis:** Unless otherwise noted, commercially available starting materials, ligands, and catalysts were used without purification. *n*-Butyllithium (2.5 M or 1.6 M in *n*-hexanes) was purchased from either ThermoFischer Scientific with AcroSeal™ or from Sigma-Aldrich with Sure/Seal™ and used as received. Intermediates **S1**, **S2**, **S3**, **S4**, **S5**, **8**, **9**, **10**, **12**, and **16** and catalyst Pd SPhos GIII were prepared according to the literature.[1-5] Control compounds **Thio[10]CPP** and **BT[10]CPP** were synthesized using previous reports.[6,7] Moisture and oxygen-sensitive reactions were carried out in flame-dried or overnight oven-dried glassware under an inert atmosphere of purified nitrogen using standard syringe/Schlenk techniques. Tetrahydrofuran (THF), dimethylformamide (DMF), and 1,4-dioxane were dried by filtration through alumina according to the methods described by Grubbs.[8] Thin-layer chromatography (TLC) was performed either on alumina plates coated with 0.20 mm thickness of Silica Gel 60 $F_{254}$ (Supelco) or alumina plates coated with 0.20 mm thickness of Aluminum Oxide Neutral (Miles Scientific). Developing plates were visualized using UV light at wavelengths of 254 and 365 nm. Silica column chromatography was conducted with Zeochem Zeoprep 60 HYD 40-63 μm silica gel. Alumina column chromatography was conducted with Sorbtech basic alumina (pH 10), Act. II-III, 50-200 μm.

**Instrumentation:** $^1$H and $^{13}$C NMR spectra were recorded on either a Bruker Avance III HD 500 MHz ($^1$H: 500 MHz, $^{13}$C: 126 MHz) or a Bruker Avance III HD 600 MHz ($^1$H: 600 MHz, $^{13}$C: 151 MHz) NMR spectrometer. The samples were measured at 25 °C. The chemical shifts (δ) were reported in parts per million (ppm) and were referenced to the residual protio-solvent ($CD_2Cl_2$, $^1$H: δ = 5.32 ppm and $^{13}$C: δ = 53.84 ppm; $CDCl_3$, $^1$H: δ = 7.26 ppm). Coupling constants (J) are given in Hz and the apparent resonance multiplicity is reported as s (singlet), d (doublet), t (triplet), q (quartet), dd (doublet of doublets), dt (doublet of triplets), dq (doublet of quartets), td (triplet of doublets), or m (multiplet). The resonances for carbon atoms attached to the boron were not observed due to the boron quadrupole. Infrared absorption (IR) spectra were recorded on a Thermo Scientific Nicolet 6700 spectrometer equipped with either a diamond crystal Smart ATR or KBr pellet from Sigma-Aldrich. Characteristic IR absorptions are reported in $cm^{-1}$ and denoted as strong (s), medium (m), and weak (w). High-resolution mass spectra (HRMS) were obtained from the Mass Spectrometry Lab of the University of Illinois at Urbana-Champaign in Champaign, Illinois, USA. UV/Vis absorption and fluorescence spectra were recorded on an Agilant Cary 100 spectrophotometer and a Horiba Jobin Yvon Fluoromax-4 Fluorimeter, respectively. All measurements were carried out under ambient conditions in a Spectrocell RF-1010-T threaded top vacuum formed borosilicate fluorometer cell (10 mm light path).

## 2. Synthesis

### a. Intermediate Synthesis

*Synthesis of One and Two Ring Containing Intermediates*

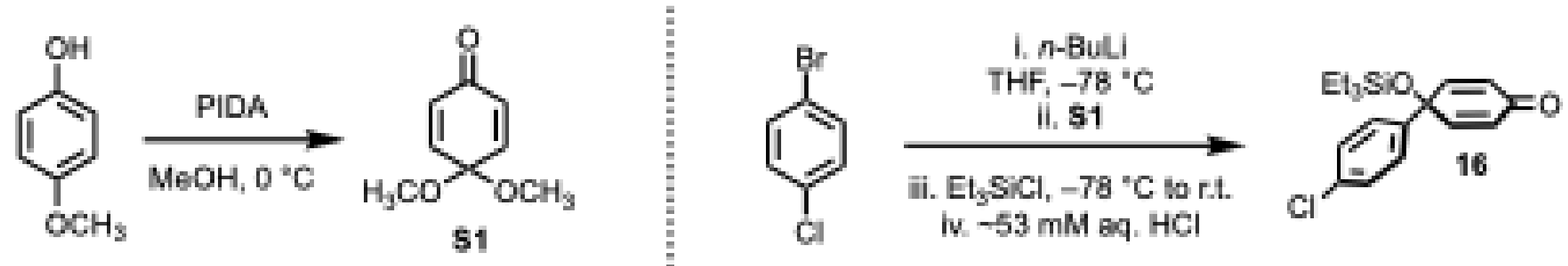


**Scheme S1.** Synthesis of intermediates **S1** and **16**.

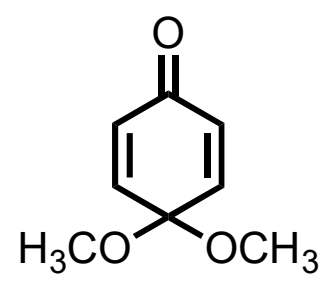


**S1.** To a 2 L round-bottomed flask equipped with a stir bar and containing MeOH (200 mL), 4-methoxyphenol (100 g, 0.806 mol, 1 equiv.) was dissolved in increments of four. Additional MeOH (695 mL) was added, using a portion to rinse any residual solid into the reaction mixture. Cooled the reaction flask to 0 °C over the course of 1 hour before adding PIDA (285 g, 0.886 mol, 1.1 equiv.) over 1.5 hours, observing the reaction color go from colorless to dark yellow. Allowed the reaction vessel to come to r.t. after all the oxidant had been added and left the reaction to run overnight. Neutralized the reaction mixture with sat. sodium bicarbonate (~500 mL) and removed roughly half of excess solvent *in vacuo* - reaction mixture turned black as it was concentrated down. Extracted the organics with DCM (200 mL x 6) and then washed the organics with water (100 mL x 1), brine (200 mL x 3), and aq. NaOH (50 mL x 1, 2 M). Dried the organics over sodium sulfate, gravity filtered the mixture and then removed solvent *in vacuo*. **Purification:** To the crude material, excess iodobenzene was removed via vacuum-distillation until approximately 12% iodobenzene remained (percentage calculated by integration via $^1$H NMR). The material was further purified by column chromatography (loaded neat, pH 10 $AlO_x$, 100% hexanes and then 20% EtOAc:Hex once pdt began eluting) yielding light-yellow oil **S1** (96.5 g, 78% yield). The $^1$H NMR (500 MHz, Chloroform-*d*) matched previous literature reports.

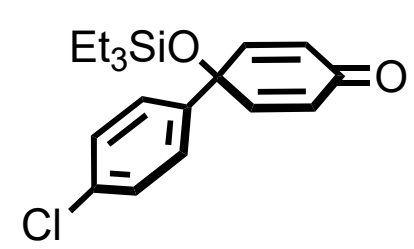


**16.** An oven-dried 500 mL three-necked flask equipped with a stir bar, a glass vacuum adapter, a glass stopper, and a 125 mL addition funnel capped with a glass stopper were cooled on high vacuum. Charged the reaction vessel with 1-bromo-4-chlorobenzene (27.4 g, 0.143 mol, 1 equiv.) before backfilling and evacuating the system (3 cycles x 3 min). Exchanged the glass stoppers for septa under positive nitrogen flow. Dissolved the solid in dry THF (275 mL) and cooled the reaction vessel to –78 °C over the course of 1 hour. Cannula transferred *n*-butyllithium (60.1 mL, 2.5 M in hexanes, 1.05 equiv.) to the addition funnel and set the reagent to add dropwise. Once all the lithiating agent had been added, the addition funnel was replaced with a rubber septum under positive nitrogen flow, any residual *n*-butyllithium on the funnel was quenched, and neat **S1** (20.1 mL, 0.143 mol, 1 equiv.) was added dropwise to the reaction vessel. Allowed the reaction to stir for 1 hour before adding neat $Et_3SiCl$ (28.8 mL, 0.171 mol, 1.2 equiv.) dropwise. Left the reaction to come to r.t. overnight. Into the three-necked flask was slowly poured an acidic, aq. mixture (40 mL DI water with five drops conc. HCl) and was left to stir for 30 minutes. The reaction

mixture was quenched with sat. sodium bicarbonate (~100 mL) and excess organic solvent was removed *in vacuo*. Extracted the organics using ethyl acetate (~150 mL x 3) before the organics were washed with brine (~150 mL x 3), dried over sodium sulfate, gravity filtered, and then removed solvent *in vacuo*. **Purification:** The material was further purified by a short plug (loaded neat, $SiO_2$, 100% hexanes and then 4% EtOAc:Hex once pdt began eluting) to yield light, yellow oil **16** (45.3 g, 96% yield). The $^1$H NMR (500 MHz, Chloroform-*d*) matched previous literature reports.

*Synthesis of Three Ring Containing Intermediates*

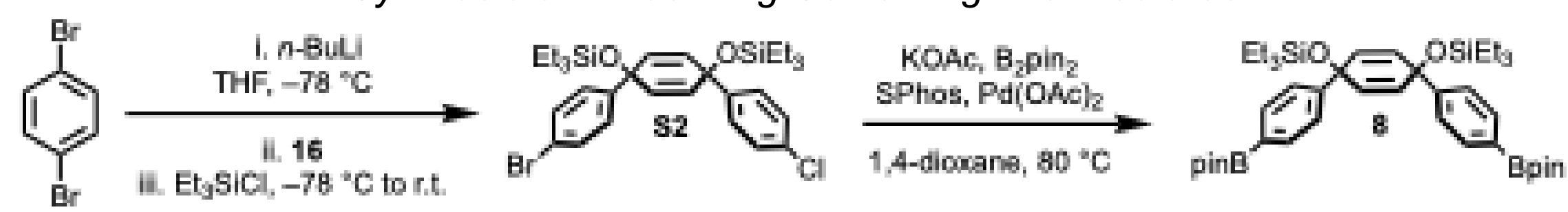


**Scheme S2.** Synthesis of intermediates **S2** and **8**.

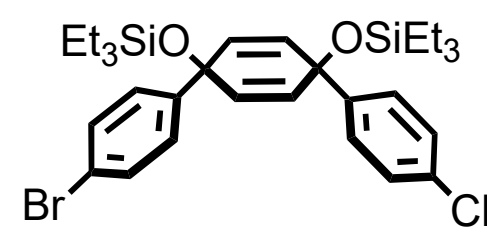

**S2.** Flame-dried a 100 mL three-necked flask equipped with a stir bar, a glass vacuum adapter, and two glass stoppers. Once cool, the flask was charged with 1,4-dibromobenzene (4.65 g, 19.7 mmol, 1 equiv.) and the system was backfilled and evacuated (3 cycles x 3 min). A glass stopper was exchanged for a septum under positive nitrogen flow, and the solid was dissolved in dry THF (66 mL) before cooling the flask to –78 °C over the course of 30 minutes. Added *n*-butyllithium (8 mL, 2.5 M in hexanes, 1.02 equiv.) to the cooled solution dropwise. Allowed the reaction to stir for 10 minutes before adding neat **16** (6.6 g, 19.7 mmol, 1 equiv.) dropwise. The reaction was left to stir for 1.25 hours before taking an aliquot to confirm all 1,4-dibromobenzene had been consumed (checked by TLC: $SiO_2$, 5% EtOAc:Hex). To the reaction vessel was added neat $Et_3SiCl$ (3.5 mL, 0.02 mmol, 1.06 equiv.) dropwise. Brought the flask to r.t. before moving the flask to a pre-heated oil bath set at 40 °C. After 1.5 hours, the crude reaction mixture was worked up – after a second aliquot had confirmed the reaction had fully progressed (checked by TLC: $SiO_2$, 5% EtOAc:Hex). Removed excess solvent *in vacuo* before the organics were extracted with ethyl acetate (~80 mL x 3). The organics were washed with brine (~60 mL x 3), dried over sodium sulfate, gravity filtered, and then removed solvent *in vacuo*. **Purification:** The material was purified by column chromatography (loaded in hexanes, $SiO_2$, 100% hexanes and then 10% DCM:Hex once **16** starting material had all eluted off) to yield crystalline, white solid **S2** (7.12 g, 60% yield). The $^1$H NMR (500 MHz, Chloroform-*d*) matched previous literature reports.

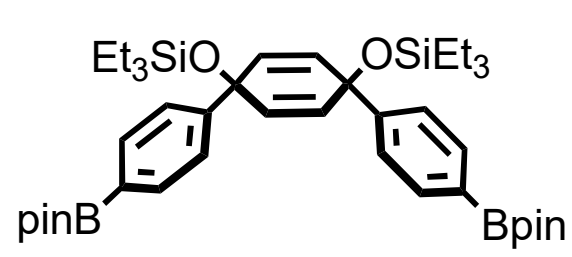

**8.** A 50 mL round-bottomed flask equipped with a stir bar and charged with KOAc (3.74 g, 38.1 mmol, 6.6 equiv.) was oven-dried for ~3.5 hours before cooling the flask on high vacuum. To the reaction vessel was added **S2** (3.5 g, 5.77 mmol, 1 equiv.), $B_2pin_2$ (3.00 g, 1.18 mmol, 2.05 equiv.), SPhos (0.296 g, 0.721 mmol, 0.125 equiv.), and $Pd(OAc)_2$ (19.4 mg, 0.0864 mmol, 0.015 equiv.). Backfilled and evacuated the system with a glass vacuum adapter (3 cycles x 3 min) and then with a septum and needle (3 cycles x 3 min). The solids were dissolved in dry 1,4-dioxane (20 mL) before the reaction vessel was lowered into a pre-heated oil bath set to 80 °C and left to run overnight. An aliquot of the reaction mixture was taken to ensure both starting materials had been consumed (checked by $^1$H NMR). Brought the reaction mixture to r.t. before running the crude

through a plug (Celite topped with a thin layer of sodium sulfate, 100% EtOAc). Concentrated the filtrate *in vacuo* before drying the crude under high vacuum. **Purification:** To remove catalyst, the material was iteratively filtered through a plug (loaded in hexanes, Celite topped with a thin layer of $SiO_2$, 5% EtOAc:Hex) to yield powdery, white solid **8** (2.54 g, 59% yield). The $^1$H NMR (500 MHz, Chloroform-*d*) matched previous literature reports.

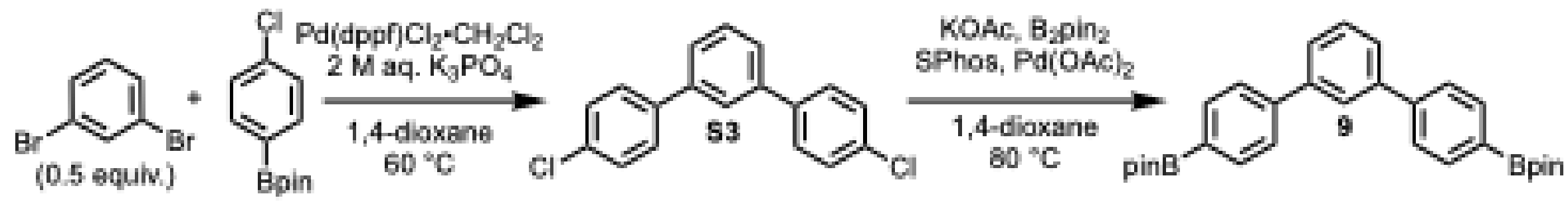


**Scheme S3.** Synthesis of intermediates **S3** and **9**.

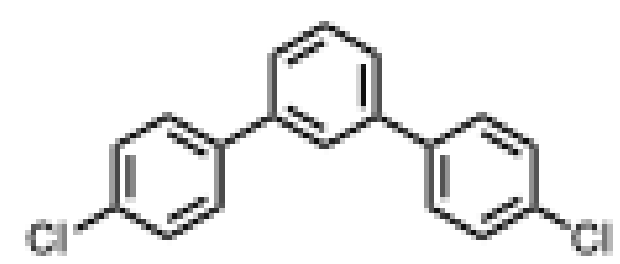

**S3.** Flame-dried a 250 mL round-bottomed flask equipped with a stir bar. Charged the flask with 4-chlorophenylboronic acid pinacol ester (4.05 g, 0.017 mol, 2.05 equiv.) and $Pd(dppf)Cl_2{\bullet}CH_2Cl_2$ (0.303 g, 0.371 mmol, 0.045 equiv.) before the system was backfilled and evacuated using a septum and needle (3 cycles x 3 min). Dissolved the solids in dry 1,4-dioxane (83 mL) before adding neat 1,3-dibromobenzene (1 mL, 8.28 mmol, 1 equiv.) dropwise. The reaction mixture was sparged for 1 hour before lowering the flask into a pre-heated oil bath set to 80 °C. Allowed the reaction vessel to equilibrate to temperature for 15 minutes before adding sparged, aq. $K_3PO_4$ (8.3 mL, 2 M) dropwise. Left the reaction to run overnight. The reaction was monitored to ensure all starting material had been consumed (checked by $^1$H NMR), and if the reaction was stalled, another complete amount of aq. $K_3PO_4$ (8.3 mL, 2 M) was added dropwise. Excess solvent was removed *in vacuo*, and the crude mixture was run through a plug (Celite topped with a thin layer of sodium sulfate, 100% DCM). **Purification:** The crude material was initially purified via automated flash column chromatography (loaded in hexanes, $SiO_2$, 100% hexanes), followed by subsequent trituration in EtOH to yield powdery, white solid **S3** (0.55 g, 22% yield). The $^1$H NMR (500 MHz, Chloroform-*d*) matched previous literature reports.

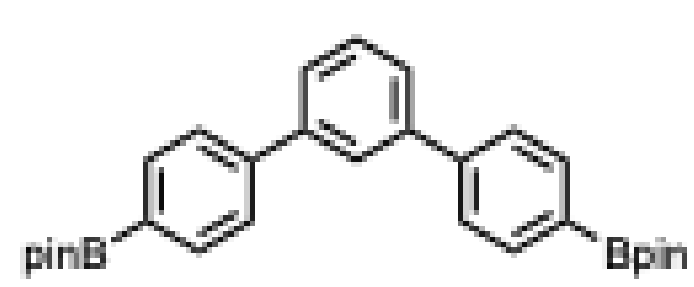

**9.** Flame-dried a 25 mL round-bottomed flask equipped with a stir bar. Once cool, charged the flask with KOAc (1.19 g, 0.0121 mol, 6.6 equiv.) before flame-drying the flask a second time. Backfilled and evacuated the flask once (1 cycle x 3 min) before adding **S3** (0.55 g, 1.84 mmol, 1 equiv.), $B_2pin_2$ (1.40 g, 5.51 mmol, 3 equiv.), SPhos (94.3 mg, 0.230 mmol, 0.125 equiv.), and $Pd(OAc)_2$ (6.10 mg, 0.0272 mmol, 0.015 equiv.). Backfilled and evacuated the flask using a glass vacuum adapter (3 cycles x 3 min) and then a septum and needle (2 cycles x 3 min). Dissolved the solids with dry 1,4-dioxane (6.12 mL) before lowering the reaction vessel into a pre-heated oil bath set to 80 °C. Allowed the reaction to run overnight. The reaction was monitored to ensure all starting material had been consumed (checked by $^1$H NMR). Removed excess solvent *in vacuo* before the crude mixture was run through a plug (loaded in hexanes, $SiO_2$ topped with a thin layer of sodium sulfate, 100% DCM). **Purification:** The crude material was iteratively triturated in EtOH to remove excess $B_2pin_2$ and to afford powdery, white solid **9** (0.77 g, 87% yield). The $^1$H NMR (500 MHz, Chloroform-*d*) matched previous literature reports.

*Synthesis of Five Ring Containing Intermediates*

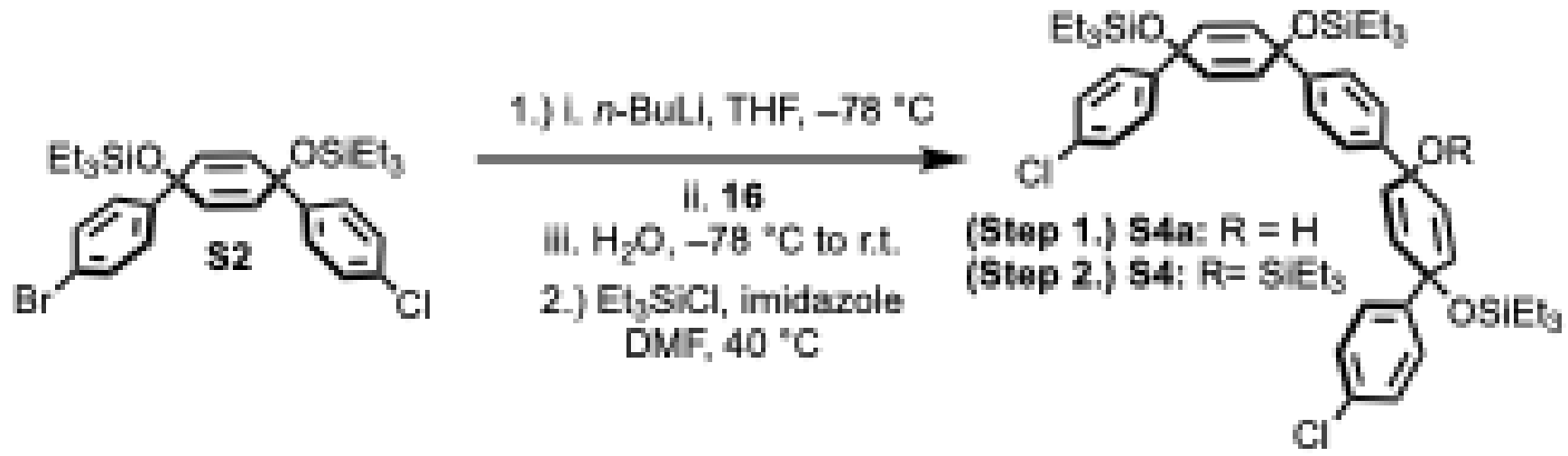


**Scheme S4.** Synthesis of intermediates **S4a** and **S4**.

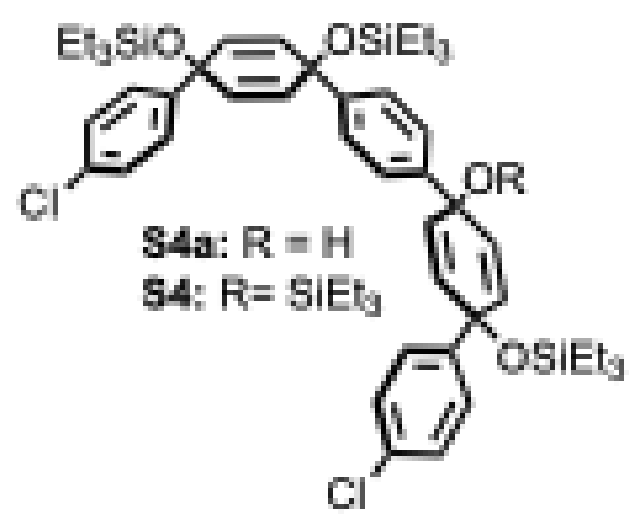


**S4a. Lithium-halogen exchange:** Flame-dried a 25 mL round-bottomed flask and a 5 mL round-bottomed flask, each equipped with a stir bar. Charged the 5 mL flask with neat **16** (0.56 g, 1.67 mmol, 1.01 equiv.) and left the material on high vacuum for 1 hour with stirring. Charged the 25 mL reaction flask with **S2** (1.00 g, 1.65 mmol, 1 equiv.) before the system was backfilled and evacuated using a glass vacuum adapter (3 cycles x 3 min) and then a septum and needle (3 cycles x 3 min). Dissolved the solids in the reaction vessel with dry THF (3.3 mL) before cooling the flask to –78 °C over the course of 15 minutes. To the reaction vessel added *n*-butyllithium (0.69 mL, 2.5 M in hexanes, 1.05 equiv.) dropwise. The reaction mixture went from colorless to light yellow. Let the reaction stir for 12 minutes before dissolving the contents of the 5 mL flask in dry THF (1 mL). Added the contents of the 5 mL flask to the reaction vessel dropwise, then rinsed the smaller flask with dry THF (~0.2 mL), and transferred the contents to the reaction vessel (x2). Allowed the reaction mixture to stir for 1 hour before slowly quenching the reaction mixture with DI water (10 mL). Removed excess solvent *in vacuo* before extracting the organics with ethyl acetate (30 mL x 2). Washed the organics with brine (30 mL x 3), dried over sodium sulfate, gravity filtered, and then removed the solvent *in vacuo*. The crude oil **S4a** was pushed forward without further purification.

**S4. Triethylsilyl Protection:** Flame-dried a 25 mL round-bottomed flask equipped with a stir bar. Backfilled and evacuated the system once (1 cycle x 3 min) before adding crude oil **S4a** (1.03 g, 1.19 mmol, 1 equiv.). To the flask added imidazole (0.244 g, 3.58 mmol, 3 equiv.) before backfilling and evacuating the system using a glass vacuum adapter (3 cycles x 3 min) and then a septum and needle (3 cycles x 3 min). Dissolved the solids in dry DMF (18 mL) before lowering the flask in a pre-heated oil bath set to 40 °C. Allowed the mixture to equilibrate to temperature over the course of 10 minutes before adding $Et_3SiCl$ (0.21 mL, 1.25 mmol, 1.05 equiv.) dropwise. Left the reaction to stir overnight. The reaction mixture was brought to r.t. before quenching with sat. sodium bicarbonate (5 mL) and left to stir for 15 minutes before extracting the organics with ethyl acetate (30 mL x 3). Washed the organics with 5% LiCl (30 mL x 3), dried over sodium sulfate, gravity filtered and then removed the solvent *in vacuo*. To remove residual solvent the crude material was placed on high vacuum for 20 minutes, redissolved in hexanes before removing solvent *in vacuo*, then placed back on high vacuum for 20 minutes (x2). **Purification:** The crude material was triturated in EtOH to afford powdery, white solid **S4** (0.93 g, 80% yield). The $^1H$ NMR (500 MHz, Chloroforom-*d*) matched previous literature reports.

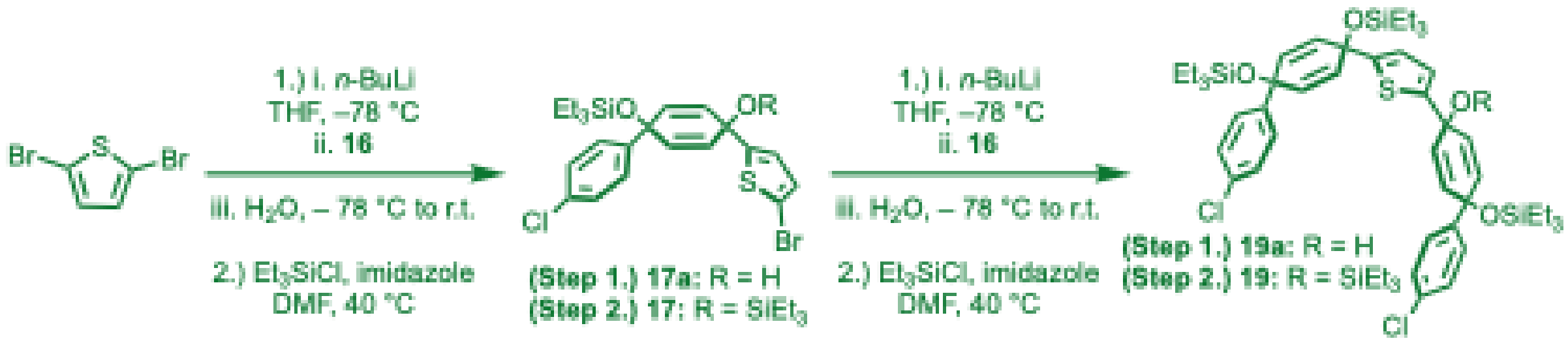


**Scheme S5.** Synthesis of intermediates **17a**, **17**, **19a**, and **19** (store in freezer for long-term storage).

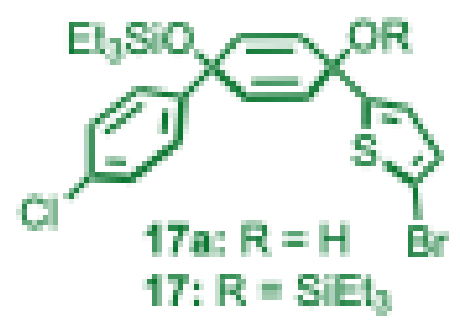


**17a. Lithium-halogen exchange:** Cooled an oven-dried 250 mL three-necked flask equipped with a stir bar, a glass vacuum adapter, a glass stopper, and a 50 mL addition funnel capped with a glass stopper on high vacuum. Charged the reaction vessel with 2,5-dibromothiophene (5 mL, 0.0444 mol, 1 equiv.) and began freeze, pump, thaw cycles (x 3). Before the starting material could fully thaw on the last cycle, exchanged the glass stopper for a septum under positive nitrogen flow and dissolved the liquid in dry THF (85 mL). Cooled the reaction vessel to –78 ° C over the course of 1.5 hours. Cannula transferred *n*-butyllithium (20 mL, 2.5 M in hexanes, 1.13 equiv.) to the addition funnel and set the reagent to add dropwise. The reaction mixture went from colorless to blue to red. Once all the lithiating agent had been added, the addition funnel was replaced with a rubber septum under positive nitrogen flow, any residual *n*-butyllithium on the funnel was quenched, and the reaction mixture was left to stir for 20 minutes. Then, neat **16** (13.5 mL, 0.0443 mol, 1 equiv.) was added dropwise to the reaction vessel and left to stir for 1 hour before slowly quenching the reaction with DI water (10 mL). The reaction mixture went from red to light yellow. The reaction vessel was brought to r.t. and excess solvent was removed *in vacuo*. The organics were extracted with ethyl acetate (50 mL x 3), subsequently the organics were washed with brine (50 mL x 3), dried over sodium sulfate, gravity filtered, and then solvent was removed *in vacuo*. Residual solvent was removed from the crude oil by gently heating (50 °C water bath) the material with stirring while on high vacuum. **Purification and Characterization:** The material was purified by column chromatography (loaded in hexanes, pH 10 $AlO_x$, 8 cm tall, 10% DCM:Hex and once impurities had eluted off 30% DCM:Hex to elute off pdt) to yield olivine-colored oil **17a** (9.95 g, 45% yield). $^1$H NMR (600 MHz, Methylene Chloride-$d_2$) δ 7.32 (d, $J$ = 8.8 Hz, 2H), 7.26 (d, $J$ = 8.8 Hz, 2H), 6.93 (d, $J$ = 3.8 Hz, 1H), 6.65 (d, $J$ = 3.8 Hz, 1H), 6.05 (d, $J$ = 10.0 Hz, 2H), 5.98 (d, $J$ = 10.0 Hz, 2H), 2.35 (s, 1H), 0.98 (t, $J$ = 7.9 Hz, 9H), 0.68 (q, $J$ = 7.9, Hz, 6H); $^{13}$C NMR (151 MHz, Methylene Chloride-$d_2$) δ 151.32, 144.36, 133.92, 133.23, 130.16, 130.02, 128.64, 127.45, 124.31, 112.34, 71.54, 68.21, 7.17, 6.74; $R_f$ = 0.25 ($AlO_x$ N, 30% DCM:Hex); IR (ATR) ṽ 730 (m), 1006 (m), 1432 (w), 2910 (w), 3429 (w); HRMS (ESI, negative mode) $m/z$ calcd for $C_{22}H_{25}BrClO_2SSi$: 495.0216 $[M]^-$, found 495.0206 (-2.0 ppm).

**17. Triethylsilyl protection:** Flame-dried a 500 mL three-necked flask equipped with a stir bar, a glass vacuum adapter, and two glass stoppers. Transferred **17a** (9.95 g, 0.02 mol, 1 equiv.) dissolved in minimal DCM to the reaction vessel, removed the solvent on high vacuum, and left the oil drying on high vacuum for 1.5 hours. To the reaction vessel added imidazole (5.44 g, 0.08 mol, 4 equiv.) before backfilling and evacuating the system (3 cycles x 3 min). Under positive nitrogen flow exchanged a glass stopper for a septum, then dissolved the starting material in dry DMF (100 mL). Lowered the reaction vessel into a pre-heated oil bath set to 40 °C. Let the reaction

equilibrate to temperature over the course of 10 minutes before adding neat $Et_3SiCl$ (6.88 mL, 0.0410 mol, 2.05 equiv.) dropwise. Allowed the reaction to run overnight. Quenched the reaction mixture with sat. sodium bicarbonate (50 mL) and let the mixture stir for 45 minutes. Extracted the organics with ethyl acetate (30 mL x 3) before the organics were washed with 5% LiCl (30 mL x 5), dried over sodium sulfate, gravity filtered, and then removed solvent *in vacuo*. Residual solvent was removed from the crude oil by gently heating (50 °C water bath) the material with stirring while on high vacuum. **Purification and Characterization:** The material was purified by column chromatography (loaded in pentane, pH 10 $AlO_x$, 8 cm tall, 100% pentane) to yield opaque light green-colored oil **17** (10.7 g, 87% yield). $^1H$ NMR (600 MHz, Methylene Chloride-$d_2$) δ 7.28 (d, $J$ = 8.7 Hz, 2H), 7.25 (d, $J$ = 8.7 Hz, 2H), 6.83 (d, $J$ = 3.8 Hz, 1H), 6.36 (d, $J$ = 3.8 Hz, 1H), 6.03 (d, $J$ = 10.1 Hz, 2H), 5.98 (d, $J$ = 10.1 Hz, 2H), 0.94 (dt, $J$ = 15.4, 8.0 Hz, 18H), 0.62 (dq, $J$ = 16.0, 7.9 Hz, 12H); $^{13}C$ NMR (151 MHz, Methylene Chloride-$d_2$) δ 153.68, 144.83, 133.30, 132.32, 130.98, 129.87, 128.61, 127.72, 123.57, 111.73, 71.51, 70.21, 7.15, 7.13, 6.71, 6.62; $R_f$ = 0.74 ($AlO_x$ N, 100% pentane); IR (ATR) ṽ 584 (m), 1017 (m), 1447 (w), 2910 (w); HRMS (ESI, positive mode) $m/z$ calcd for $C_{28}H_{40}BrClO_2SSi_2$: 610.1159 $[M]^+$, found 610.1149 (-1.6 ppm).

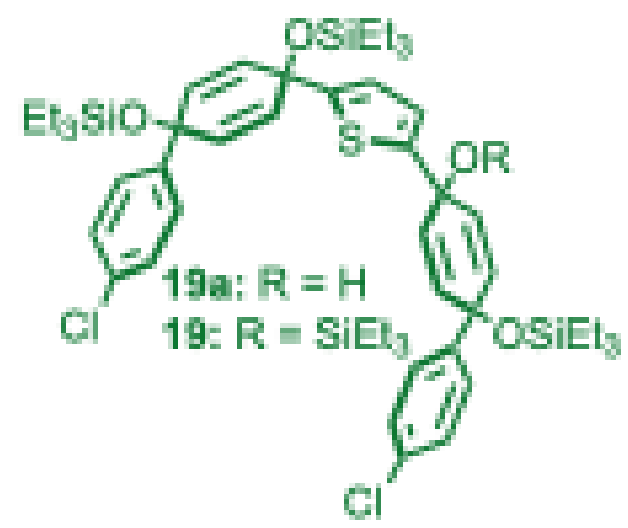


**19a. Lithium-halogen exchange:** Flame-dried a 50 mL three-necked flask equipped with a stir bar, two glass stoppers, and a glass vacuum adapter. Charged the flask with **17** (4 g, 6.53 mmol, 1 equiv.) before backfilling and evacuating the system (3 cycles x 3 min). Exchanged a glass stopper for a septum under positive nitrogen flow and dissolved the oil in dry THF (13 mL). Sonicated the flask under inert atmosphere if the stir bar could not rotate freely. Cooled the reaction vessel to –78 °C over the span of 15 minutes. To the reaction vessel added *n*-butyllithium (2.75 mL, 2.5 M in hexanes, 1.05 equiv.) dropwise. The reaction mixture went from light green to bright orange. Allowed the reaction mixture to stir for 12 minutes. Added neat **16** (1.99 mL, 6.54 mmol, 1 equiv.) dropwise and slowly to ensure the oil was dissolving instead of freezing. Left the reaction to stir for 1 hour before adding DI water (2 mL). Allowed the reaction mixture to come to room temperature over the course of 45 minutes. Removed excess solvent *in vacuo* before extracting the organics with ethyl acetate (50 mL x 3), then the organics were washed with brine (50 mL x 3), dried over sodium sulfate, gravity filtered, and then the solvent was removed *in vacuo*. Residual solvent was removed from the crude oil by stirring the crude material while on high vacuum. **Purification and Characterization:** The material was purified by column chromatography (loaded in 10% DCM:Hex, pH 10 $AlO_x$, 8 cm tall, gradient 10% DCM:Hex - 20% DCM:Hex) to yield colorless oil **19a** that slowly turned a light purple (4.99 g, 88% yield). $^1H$ NMR (600 MHz, Methylene Chloride-$d_2$) δ 7.32 (d, $J$ = 8.6 Hz, 2H), 7.29 (d, $J$ = 8.6 Hz, 2H), 7.23-7.21 (m, 4H), 6.71 (d, $J$ = 3.7 Hz, 2H), 6.55 (d, $J$ = 3.7 Hz), 6.11-6.08 (m, 4H), 5.95 (t, $J$ = 10.2 Hz, 4H), 2.31 (s, 1H), 0.99 (t, $J$ = 7.9 Hz, 9H), 0.95 (t, $J$ = 7.9 Hz, 18H), 0.69 (q, $J$ = 7.9 Hz, 4H), 0.61 (dq, $J$ = 8.0, 4.1 Hz, 12H); $^{13}C$ NMR (151 MHz, Methylene Chloride-$d_2$) δ 151.79, 149.05, 144.95, 144.57, 133.37, 133.19, 133.11, 131.97, 131.40, 130.59, 128.52, 127.74, 127.50, 123.49, 123.21, 71.65, 71.61, 69.92, 68.00, 7.20, 7.18, 7.17, 6.74, 6.74, 6.72, 6.65.

**19. Triethylsilyl protection:** Flame-dried a 100 mL round-bottomed flask equipped with a stir bar. Backfilled and evacuated the system (1 cycle x 3 min) before charging the flask with **19a** (3.07 g, 3.54 mmol, 1 equiv.) dissolved in minimal DCM. Removed the solvent on high vacuum

and left the oil to dry on high vacuum for 30 minutes. To the reaction vessel added imidazole (0.962 g, 0.0141 mol, 4 equiv.) before backfilling and evacuating the system (3 cycles x 3 min). Under positive nitrogen flow exchanged a glass stopper for a septum, then dissolved the starting material in dry DMF (18 mL). Lowered the reaction vessel into a pre-heated oil bath set to 40 °C. Let the reaction equilibrate to temperature over the course of 10 minutes before adding neat $Et_3SiCl$ (0.62 mL, 3.69 mmol, 1.04 equiv.) dropwise. Allowed the reaction to run overnight. Quenched the reaction mixture with sat. sodium bicarbonate (8 mL) and let the mixture stir for 5 minutes. Extracted the organics with ethyl acetate (20 mL x 3) before the organics were washed with 5% LiCl (20 mL x 5), dried over sodium sulfate, gravity filtered, and then removed solvent *in vacuo*. Residual solvent was removed from the crude oil by gently heating (50 °C water bath) the material with stirring while on high vacuum. **Purification and Characterization:** The material was purified by column chromatography to yield light brown oil **19** (3.2 g, 92% yield). $^1H$ NMR (600 MHz, Methylene Chloride-$d_2$) δ 7.26 (d, $J$ = 8.6 Hz, 4H), 7.18 (d, $J$ = 8.6 Hz, 4H), 6.52 (s, 2H), 6.11 (d, $J$ = 10.0 Hz, 4H), 5.92 (d, $J$ = 10.0 Hz, 4H), 0.97-0.92 (m, 36H), 0.65-0.58 (m, 24H); $^{13}C$ NMR (151 MHz, Methylene Chloride-$d_2$) δ 151.16, 144.98, 133.09, 131.87, 131.43, 128.45, 127.69, 123.05, 71.68, 69.76, 7.20, 7.17, 6.71, 6.68.

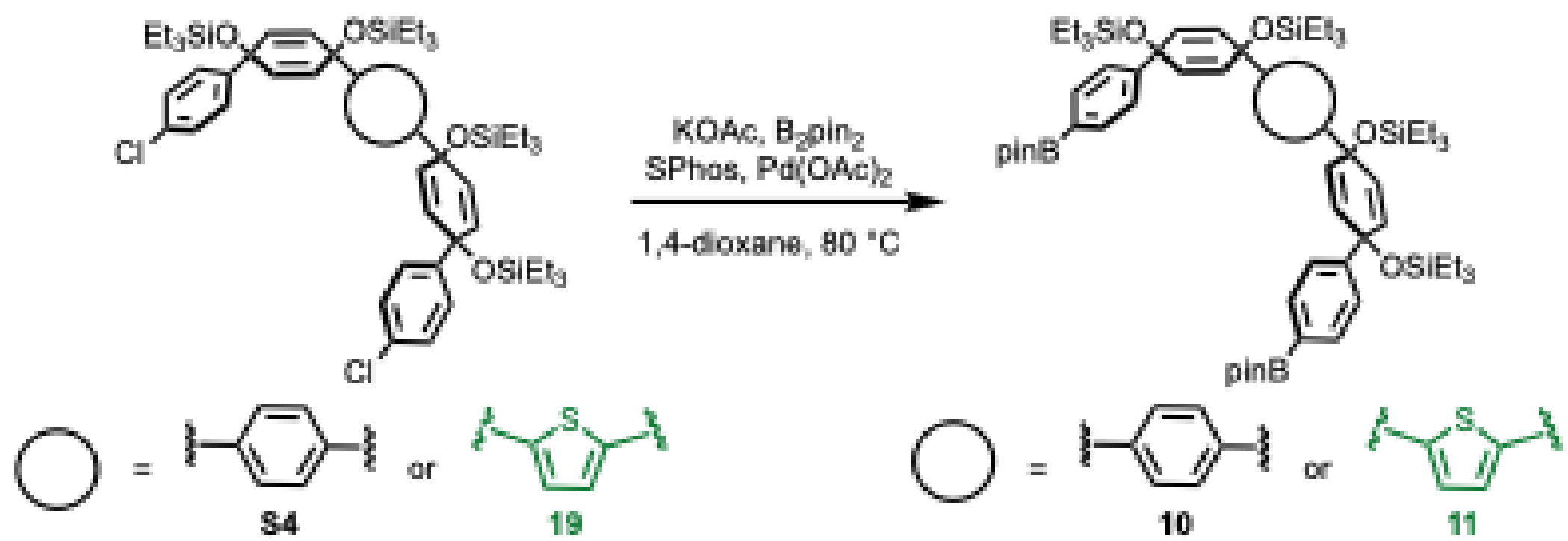


**Scheme S6.** Synthesis of intermediates **10** and **11** (store **11** in freezer for long-term storage).

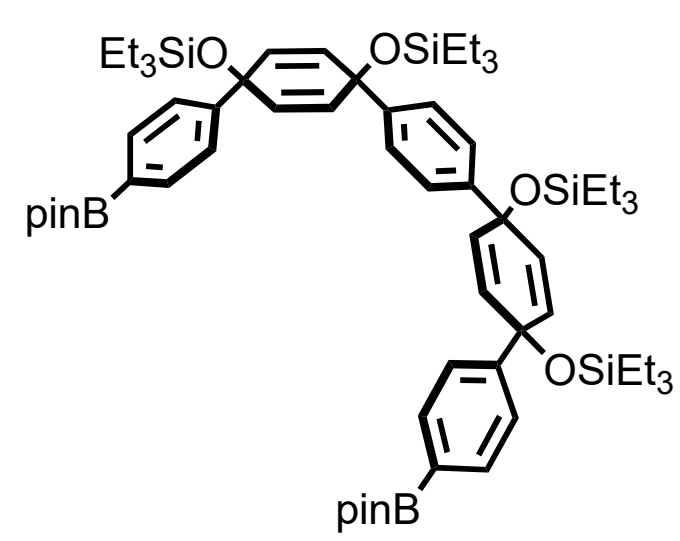


**10.** A 15 mL two-necked flask equipped with a stir bar and charged with KOAc (0.940 g, 9.57 mmol, 6.7 equiv.) was oven-dried, alongside a glass stopper and glass vacuum adapter, overnight. The reaction vessel was cooled on high vacuum before adding **S4** (1.4 g, 1.43 mmol, 1 equiv.), $B_2pin_2$ (0.910 g, 3.58 mmol, 2.5 equiv.), SPhos (0.074 g, 0.180 mmol, 0.125 equiv.), and $Pd(OAc)_2$ (4.8 mg, 0.0214 mmol, 0.0150 equiv.). Backfilled and evacuated the system (3 cycles x 4 min) before exchanging the glass stopper for a septum under positive nitrogen flow. The solids were dissolved in dry 1,4-dioxane (4.80 mL) before the reaction vessel was lowered into a pre-heated oil bath set to 80 °C and left to run overnight. An aliquot was taken to confirm all **S4** had been consumed (checked by TLC: $SiO_2$, 20% DCM:Hex). Brought the reaction mixture to r.t. before extracting the organics with DCM (20 mL x 3). Washed the organics with brine (20 mL x 3), dried over sodium sulfate, gravity filtered and then removed the solvent *in vacuo*. To remove residual solvent the crude material was placed on high vacuum for 30 minutes, redissolved in acetone before removing solvent *in vacuo*, then placed back on high vacuum for

30 minutes (x2). **Purification:** The off-white material was triturated in EtOH:MeOH (~50:50) to remove excess $B_2pin_2$ and to afford shiny, off-white solid **10** (1.19 g, 72% yield). The $^1$H NMR (500 MHz, Chloroform-*d*) matched previous literature reports.

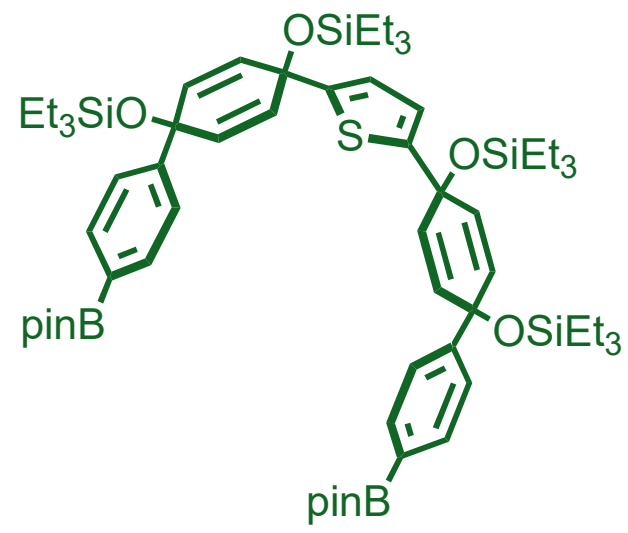


**11.** Flame-dried a 15 mL two-necked equipped with a stir bar, a glass stopper, and a glass vacuum adapter. Once cool, charged the flask with KOAc (0.363 g, 3.70 mmol, 6.6 equiv.) before flame-drying the flask a second time. To the flask was added **19** (0.550 g, 0.560 mmol, 1 equiv.), $B_2pin_2$ (0.291 g, 1.15 mmol, 2.05 equiv.), SPhos (0.029 g, 0.0706 mmol, 0.126 equiv.), and $Pd(OAc)_2$ (1.89 mg, 8.91 µmol, 0.0159 equiv.). Backfilled and evacuated the system (3 cycles x 3 min) before exchanging the glass stopper for a septum under positive nitrogen flow. The solids were dissolved in dry 1,4-dioxane (4 mL) before the reaction vessel was lowered into a pre-heated oil bath set to 80 °C and left to run overnight. An aliquot was taken to confirm all **19** had been consumed (checked by TLC: $AlO_x$ N, 10% DCM:Hex). Brought the reaction mixture to r.t. before extracting the organics with ethyl acetate (15 mL x 3). Washed the organics with 5% LiCl (15 mL x 4), dried over sodium sulfate, gravity filtered and then removed the solvent *in vacuo*. To remove residual solvent the crude material was placed on high vacuum for 30 minutes, redissolved in acetone before removing solvent *in vacuo*, then placed back on high vacuum for 30 minutes (x 3). **Purification and Characterization:** The off-white material was triturated in EtOH to remove excess $B_2pin_2$ and small impurities to afford white solid **11** (0.497 g, 76% yield). $^1$H NMR (600 MHz, Methylene Chloride-$d_2$) δ 7.62 (d, *J* = 8.2 Hz, 4H), 7.33 (d, *J* = 8.2 Hz, 4H), 6.50 (s, 2H), 6.08 (d, *J* = 10.0 Hz, 4H), 5.95 (d, *J* = 10.1 Hz, 4H), 1.30 (s, 24H), 0.94 (t, *J* = 7.7 Hz, 36H), 0.62 (p, *J* = 7.9 Hz, 24H); $^{13}$C NMR (151 MHz, Methylene Chloride-$d_2$) δ 151.18, 149.30, 134.89, 131.93, 131.29, 125.47, 123.01, 84.06, 72.05, 69.92, 25.04, 7.21, 7.18, 6.74, 6.67; IR (KBr) ṽ 727 (m), 1089 (m), 1361 (m), 2875 (w), 2956 (w); HRMS (MALDI) *m*/*z* calcd for $C_{64}H_{102}B_2O_8SSi_4$: 1164.6559 [M]+, found 1164.6553 (1.6 ppm).

*Synthesis of Seven Ring Containing Intermediates*

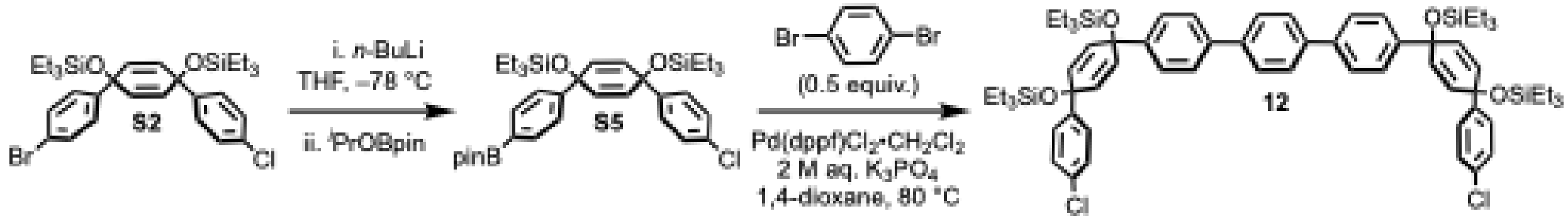

**Scheme S7.** Synthesis of intermediate **S5** and **12**.

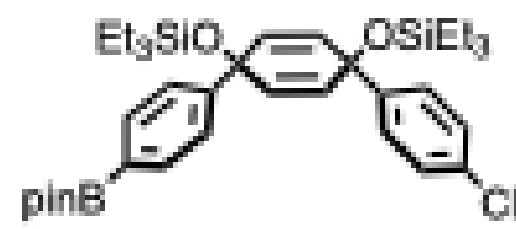

**S5.** Cooled an overnight, oven-dried 100 mL three-necked flask equipped with a stir bar, two glass stoppers, and a glass vacuum adapter on high vacuum. Charged the flask with **S2** (5 g, 8.25 mmol, 1 equiv.) before backfilling and evacuating the system (3 cycles x 3 min). Exchanged a glass stopper for a septum under positive nitrogen flow before dissolving the solid in dry THF (41.2 mL). Cooled the flask to –78 ·C over the course of 45 minutes. To the reaction vessel added *n*-butyllithium (3.46 mL, 2.5 M in hexanes, 1.05 equiv.) dropwise, left the reaction to stir for 10 minutes, and then added $^{i}$PrOBpin (1.77 mL, 8.68 mmol, 1.05 equiv.) dropwise. Allowed the reaction to stir for 1 hour before quenching the reaction mixture with DI water (8 mL). Let come to room temperature before excess solvent was removed *in vacuo* before extracting the organics with ethyl acetate (30 mL x 3). Washed the organics with brine (30 mL x 3), dried over sodium sulfate, gravity filtered and then removed the solvent *in vacuo*. To remove residual solvent the crude material was placed on high vacuum for 30 minutes, redissolved in acetone before removing solvent *in vacuo*, then placed back on high vacuum for 30 minutes (x2). **Purification:** The crude oil was triturated in EtOH to yield off-white solid **S5** (3.36 g, 62% yield). The $^{1}$H NMR (500 MHz, Chloroform-*d*) matched previous literature reports.

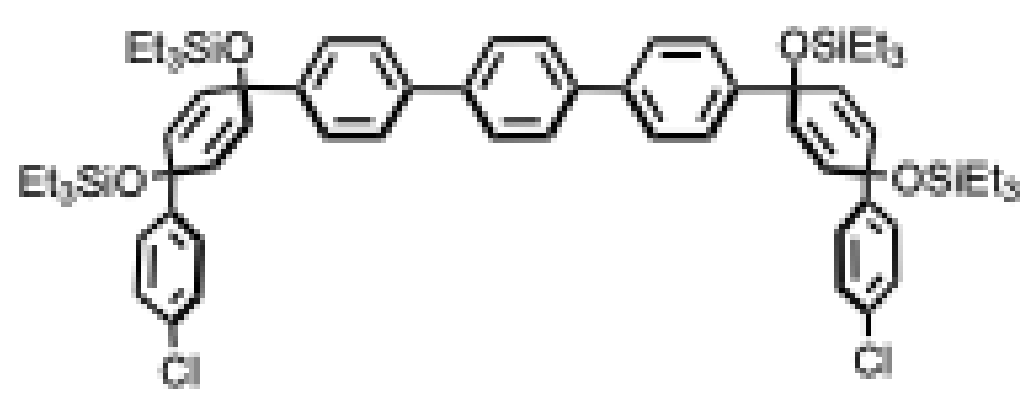

**12.** Flame dried a 50 mL three-necked flask equipped with a stir bar, two glass stoppers, and a glass vacuum adapter. Charged the flask with 1,4-dibromobenzene (0.155, 0.657 mmol, 1 equiv.), **S5** (0.900 g, 1.38 mmol, 2.10 equiv.), and Pd(dppf)$Cl_2$ (0.027 g, 0.033 mmol, 0.05 equiv.) under positive nitrogen flow. Backfilled and evacuated the system with the glass vacuum adapter (3 cycles x 3 min) before dissolving the solids in dry 1,4-dioxane (13.78 mL). The reaction vessel was lowered into a pre-heated oil bath set to 80 °C, the flask was left to equilibrate to temperature over the course of 10 minutes, and then sparged aq. $K_3PO_4$ (1.38 mL, 2 M) was added dropwise before the reaction was left to run overnight. The reaction mixture was brought to r.t. before extracting the organics with ethyl acetate (20 mL x 3). Washed the organics with brine (20 mL x 3), dried over sodium sulfate, gravity filtered and then removed the solvent *in vacuo*. **Purification:** The material was purified by column chromatography (loaded in 5% DCM:Hex, $SiO_2$, gradient 10% DCM:Hex - 20% DCM:Hex) to yield white solid **12** (0.411 g, 56% yield). $^{1}$H NMR (600 MHz, Methylene Chloride-*d*$_2$) δ 7.68 (s, 4H), 7.58 (d, *J* = 8.5 Hz, 4H), 7.41 (d, *J* = 8.4 Hz, 4H), 7.32 (d, *J* = 8.7 Hz, 4H), 7.25 (d, *J* = 8.6 Hz, 4H), 6.07 (d, *J* = 10.1 Hz, 4H), 6.00 (d, *J* = 10.2 Hz, 4H), 0.95 (td, *J* = 7.9, 5.3 Hz, 36H), 0.64 (td, *J* = 7.9, 5.6 Hz, 24H); $^{13}$C NMR (151 MHz, Methylene Chloride-*d*$_2$) δ 145.51, 145.24, 139.91, 139.79, 133.20, 132.21, 131.62, 128.56, 127.85, 127.68, 127.03,

126.75, 71.69, 71.56, 7.22, 7.20, 6.78, 6.75; $R_f$ = 0.26 ($SiO_2$, 20% DCM:Hex); HRMS (TOF MS AP+) *m/z* calcd for $C_{66}H_{88}Cl_2O_4Si_4$: 1126.5137 [M]+, found 1126.5134 (-0.3 ppm).

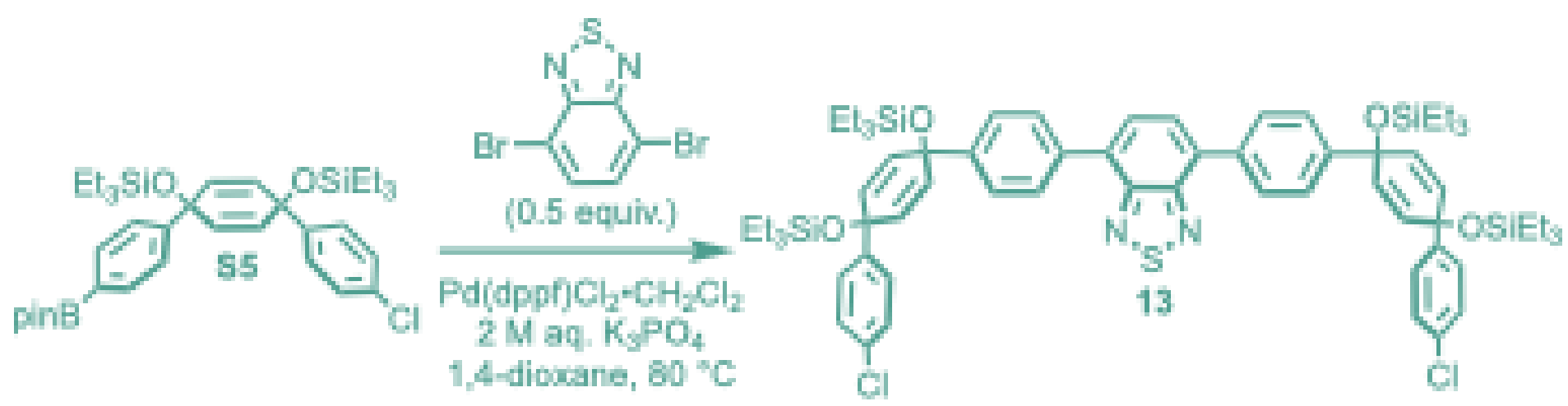


**Scheme S8.** Synthesis of intermediate **13**.

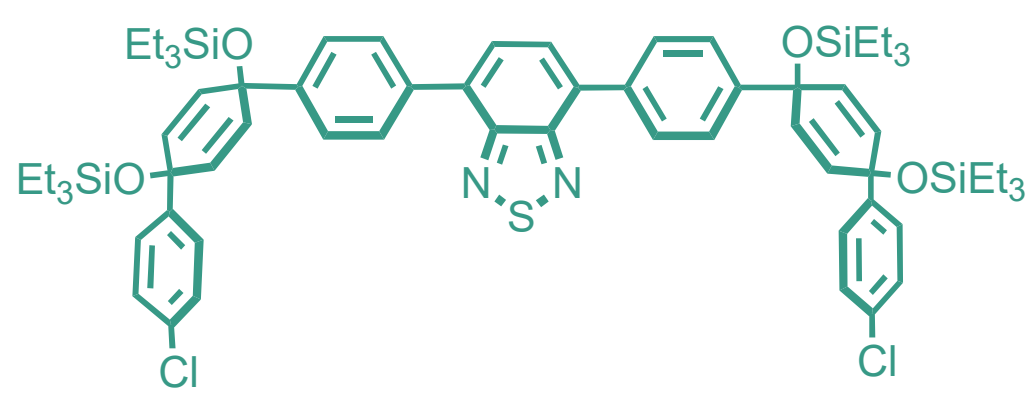


**13.** Flame dried a 25 mL Schlenk flask equipped with a stir bar and glass stopper. Charged the flask with 4,7-dibromo-2,1,3-benzothiadiazole (0.220 g, 0.748 mmol, 1 equiv.), **S5** (1.00 g, 1.53 mmol, 2.05 equiv.), and Pd(dppf)$Cl_2$ (0.030 g, 0.037 mmol, 0.049 equiv.), under positive nitrogen flow. Backfilled and evacuated the system (3 cycles x 3 min) before dissolving the solids in dry 1,4-dioxane (15.3 mL). The reaction vessel was lowered into a pre-heated oil bath set to 80 °C, the flask was left to equilibrate to temperature over the course of 10 minutes, and then sparged aq. $K_3PO_4$ (1.53 mL, 2 M) was added dropwise before the reaction was left to run overnight. An aliquot was taken to confirm **13** was the predominant species (checked by TLC: $SiO_2$, 20% DCM:Hex). The reaction mixture was brought to r.t. before excess solvent was removed *in vacuo*. The crude was redissolved in ethyl acetate before extracting the organics with ethyl acetate (50 mL x 3). Washed the organics with brine (40 mL x 3), dried over sodium sulfate, gravity filtered and then removed the solvent *in vacuo*. To remove residual solvent the crude material was placed on high vacuum for 30 minutes, redissolved in acetone before removing solvent *in vacuo*, then placed back on high vacuum for 30 minutes (x2). **Purification and Characterization:** The material was purified by column chromatography (dry loaded, $SiO_2$, gradient 100% Hex - 40% DCM:Hex) to yield fluffy, neon green solid **12** (0.710 g, 39% yield). $^{1}$H NMR (600 MHz, Methylene Chloride-$d_2$) δ 7.91 (d, $J$ = 8.5 Hz, 4H), 7.80 (s, 2H), 7.48 (d, $J$ = 8.5 Hz, 4H), 7.36 (d, $J$ = 8.6 Hz, 4H), 7.27 (d, $J$ = 8.6 Hz, 4H), 6.10 (d, $J$ = 10.2 Hz, 4H), 6.05 (d, $J$ = 10.2 Hz, 4H), 1.02 - 0.93 (m, 36H), 0.70 - 0.61 (m, 24H); $^{13}$C NMR (151 MHz, Methylene Chloride-$d_2$) δ 154.53, 146.44, 145.28, 136.81, 133.29, 133.18, 132.27, 131.71, 129.50, 128.63, 128.41, 127.97, 126.39, 71.85, 71.56, 7.25, 7.21, 6.84, 6.80; $R_f$ = 0.44 ($SiO_2$, 20% DCM:Hex); IR (ATR) ṽ 2917 (w), 1072 (m), 709 (m); HRMS (MALDI) *m*/*z* calcd for $C_{66}H_{87}Cl_2N_2O_4SSi_4$: 1,184.4762 [M]+, found 1,185.4835 (0.5841 ppm).

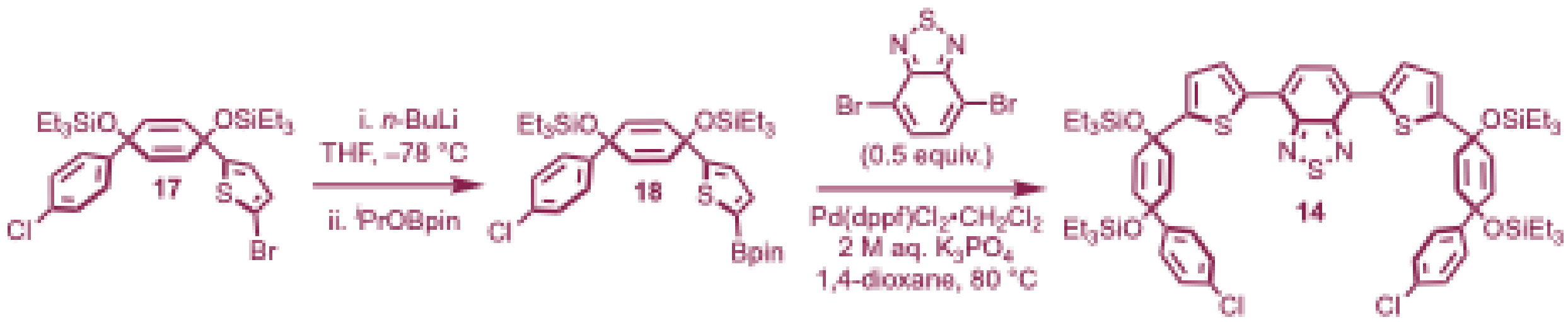


**Scheme S9.** Synthesis of intermediates **18** and **14** (store **18** and **14** in freezer for long-term storage).

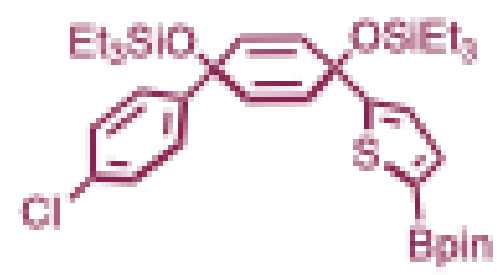

**18.** Flame dried a 100 mL three-necked flask equipped with a stir bar, two glass stoppers, and a glass vacuum adapter. Once cool exchanged a glass stopper for a septum under positive nitrogen flow, then exchanged the glass vacuum adapter for a septum under positive nitrogen flow coming from a needle inserted in the first septa. Pre-weighed the flask using a scale inside the fume hood. Kept the flask under positive nitrogen flow to transfer **17** (3.2 g, 5.23 mmol, 1 equiv.) using a Pasteur pipette, took off the Schlenk line to weigh, and repeated this process until the desired amount was in the flask. Backfilled and evacuated the neat oil (3 cycles x 3 min) before dissolving the oil in dry THF (42 mL). Cooled the flask to –78 °C over the course of 45 minutes. To the reaction vessel added *n*-butyllithium (2.20 mL, 2.5 M in hexanes, 1.05 equiv.) dropwise, left the reaction to stir for 15 minutes, and then added $^{i}$PrOBpin (1.12 mL, 5.49 mmol, 1.05 equiv.) dropwise. Allowed the reaction to stir for 1 hour before quenching the reaction mixture with DI water (10 mL). Let come to room temperature before excess solvent was removed *in vacuo* before extracting the organics with ethyl acetate (40 mL x 3). Washed the organics with brine (40 mL x 3), dried over sodium sulfate, gravity filtered and then removed the solvent *in vacuo*. **Purification and Characterization:** The material was either pushed forward without further purification or purified via minimal sonication in acetonitrile to give a light brown filtrate that was concentrated down to give light brown oil **18** (2.48 g, 72% yield). $^{1}$H NMR (600 MHz, Methylene Chloride-$d_2$) δ 7.36 (d, *J* = 3.5 Hz, 1H), 7.27 (d, *J* = 8.7 Hz, 2H), 7.22 (d, *J* = 8.6 Hz, 2H), 6.75 (d, *J* = 3.5 Hz, 1H), 6.11 (d, *J* = 10.0 Hz, 2H), 5.95 (d, *J* = 10.0 Hz, 2H), 1.32 (s, 12H), 0.94 (td, *J* = 8.0, 1.9 Hz, 18H), 0.62 (p, *J* = 7.8 Hz, 12H); $^{13}$C NMR (151 MHz, Methylene Chloride-$d_2$) δ 159.03, 144.96, 137.19, 133.21, 131.95, 131.50, 128.55, 127.75, 125.11, 84.49, 71.63, 69.99, 24.97, 7.19, 7.17, 6.75, 6.66; IR (ATR) ṽ 2914 (w), 1072 (m), 719 (m); HRMS (ESI, positive mode) *m*/*z* calcd for $C_{34}H_{52}BClO_4SSi_2$: 658.2906 [M]+, found 658.2906 (-0.5 ppm).

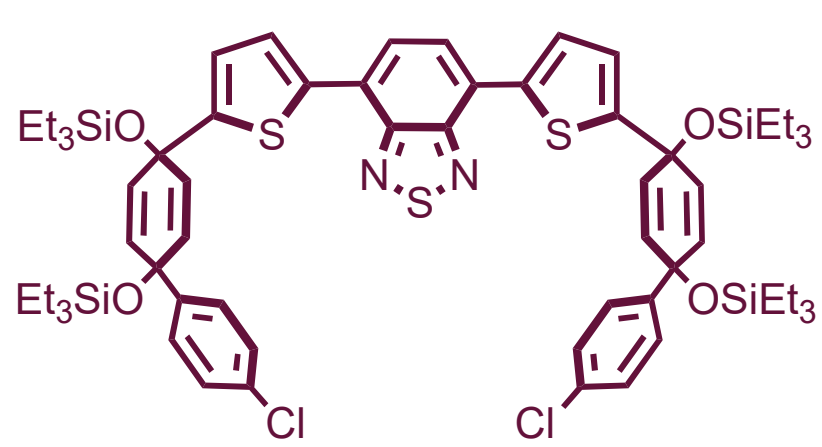

**14.** Cooled an overnight oven dried 50 mL three-necked flask equipped with a stir bar, two glass stoppers, and a glass vacuum adapter before charging the flask with 4,7-dibromo-2,1,3-benzothiadiazole (1.5 g, 5.10 mmol, 1 equiv.), **18** (6.73 g, 0.010 mol, 2.00 equiv.), and $Pd(dppf)Cl_2 \cdot CH_2Cl_2$ (0.187 g, 0.228 mmol, 0.045 equiv.), under positive nitrogen flow. Backfilled and evacuated the system (3 cycles x 3 min) before dissolving the solids in dry 1,4-dioxane (17 mL). The reaction vessel was lowered into a pre-heated oil bath set to 80 °C, the flask was left to

equilibrate to temperature over the course of 10 minutes, and then sparged aq. $K_3PO_4$ (5.10 mL, 2 M) was added dropwise before the reaction was left to run overnight. An aliquot was taken to confirm all the starting material had been consumed (checked by $^1$H NMR). The reaction mixture was brought to r.t. before excess solvent was removed *in vacuo*. The crude was redissolved in ethyl acetate before extracting the organics with ethyl acetate (80 mL x 3). Washed the organics with brine (80 mL x 2), dried over sodium sulfate, gravity filtered and then removed the solvent *in vacuo*. To remove residual solvent the crude material was placed on high vacuum for 30 minutes, redissolved in acetone before removing solvent *in vacuo*, then placed back on high vacuum for 30 minutes (x2). **Purification and Characterization:** The material was purified by automated flash column chromatography (loaded in hexanes, pH 10 $AlO_x$, gradient 100% Hex - 15% DCM:Hex) to yield fluffy, cherry red solid **14** (1.75 g, 29% yield). $^1$H NMR (500 MHz, Chloroform-*d*) δ 7.96 (d, *J* = 3.8 Hz, 2H), 7.81 (s, 2H), 7.33 (d, *J* = 8.6 Hz, 4H), 7.24 (d, *J* = 8.6 Hz, 4H), 6.79 (d, *J* = 3.8 Hz, 2H), 6.17 (d, *J* = 10.1 Hz, 4H), 5.98 (d, *J* = 10.0 Hz, 4H), 0.97 (dt, *J* = 10.7, 7.9 Hz, 36H), 0.70 - 0.61 (m, 24H); $^{13}$C NMR (126 MHz, Methylene Chloride-*d*$_2$) δ 153.26, 152.99, 144.98, 139.18, 133.25, 132.16, 131.38, 128.60, 127.84, 127.69, 126.28, 125.82, 124.68, 71.67, 70.13, 7.22, 7.21, 6.77, 6.75; $R_f$ = 0.44 ($AlO_x$ N, 20% DCM:Hex); HRMS (MALDI) *m*/*z* calcd for $C_{62}H_{82}Cl_2N_2O_4S_3Si_4$: 1,196.3891 [M]+, found 1,196.3885 (1.5227 ppm).

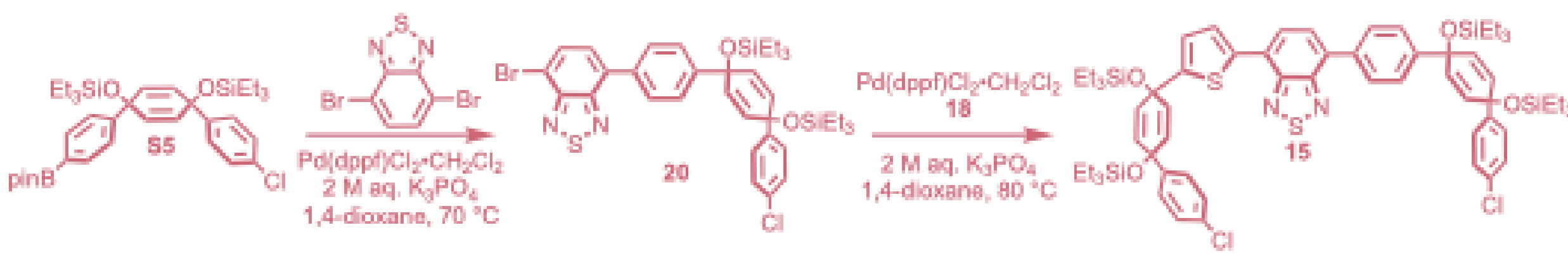


**Scheme S10.** Synthesis of intermediates **20** and **15** (store **20** and **15** in freezer for long-term storage).

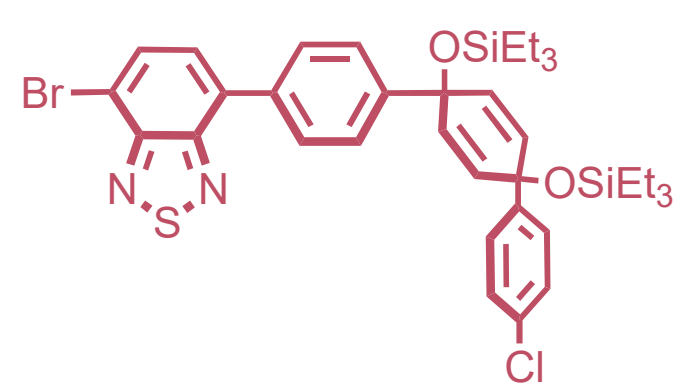


**20.** Placed a 50 mL Schlenk flask, a stir bar, and a glass stopper in the oven for a minimum of 3 hours before cooling on high vacuum. Charged the flask with 4,7-dibromo-2,1,3-benzothiadiazole (1.4 g, 4.76 mmol, 1 equiv.), **S5** (3.11 g, 4.76 mmol, 1 equiv.), and $Pd(dppf)Cl_2•CH_2Cl_2$ (0.300 g, 0.368 mmol, 0.077 equiv.), under positive nitrogen flow. Backfilled and evacuated the system (3 cycles x 3 min) before dissolving the solids in dry 1,4-dioxane (16 mL). The reaction vessel was lowered into a pre-heated oil bath set to 70 °C, the flask was left to equilibrate to temperature over the course of 10 minutes, and then sparged aq. $K_3PO_4$ (4.76 mL, 2 M) was added dropwise before the reaction was left to run overnight. An aliquot was taken to confirm all **S5** starting material had been consumed (checked by $^1$H NMR). The reaction mixture was brought to r.t. before excess solvent was removed *in vacuo*. The crude was redissolved in ethyl acetate before extracting the organics with ethyl acetate (50 mL x 3). Washed the organics with brine (50 mL x 2), dried over sodium sulfate, gravity filtered and then removed the solvent *in vacuo*. To remove residual solvent the crude material was placed on high vacuum for 30 minutes, redissolved in acetone before removing solvent *in vacuo*, then placed back on high vacuum for 30 minutes (x2). **Purification and Characterization:** The material was purified by automated flash column chromatography (loaded in hexanes, $SiO_2$, gradient 100% Hex - 18% DCM:Hex) to yield sticky, yellow-green oil **20** (0.933 g, 26% yield). $^1$H NMR (600 MHz, Methylene Chloride-*d*$_2$) δ 7.93 (d, *J* = 7.6 Hz, 1H), 7.83 (d, *J* = 8.4 Hz, 2H), 7.59 (d, *J* = 7.5 Hz, 1H), 7.47 (d, *J* = 8.4 Hz, 2H), 7.34 (d, *J* = 8.7 Hz,

2H), 7.26 (d, *J* = 8.6 Hz, 2H), 6.10 - 6.02 (m, 4H), 0.96 (dt, *J* = 15.6, 7.9 Hz, 18H), 0.65 (dq, *J* = 24.0, 7.9 Hz, 12H); $^{13}C$ NMR (151 MHz, Methylene Chloride-$d_2$) δ 154.31, 153.60, 146.83, 145.24, 136.04, 133.95, 133.31, 132.70 ,132.18, 131.77, 129.46, 128.63, 128.58, 127.92, 126.47, 113.34, 71.83, 71.52, 7.24, 7.20, 6.82, 6.80; $R_f$ = 0.52 ($SiO_2$, 40% DCM:Hex); IR (ATR) ṽ 2910 (w), 1069 (m), 715 (m); HRMS (ESI, positive mode) *m*/*z* calcd for $C_{36}H_{44}BrClN_2O_2SSi_2$: 738.1534 [M]+, found 738.1530 (-0.5 ppm).

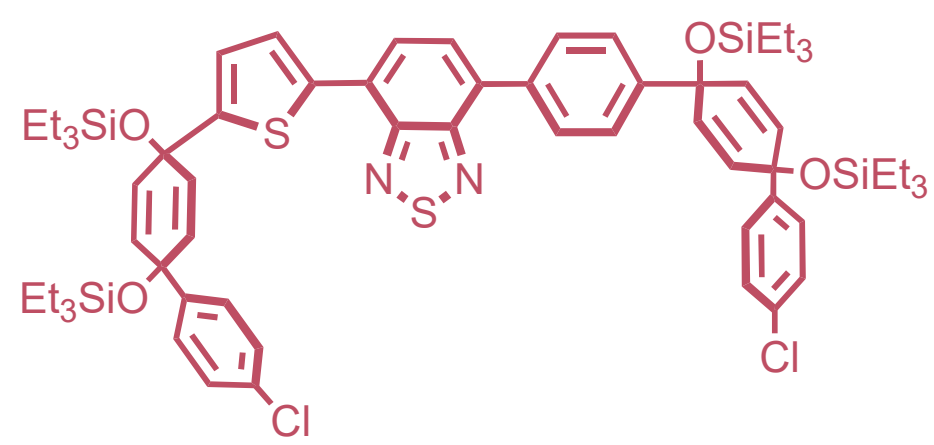


**15.** Cooled an oven dried 5 mL two-necked flask equipped with a stir bar, a glass stopper, and a glass vacuum adapter on high vacuum. Charged the flask with **20** (0.4 g, 0.540 mmol, 1 equiv.) dissolved in DCM and removed the solvent *in vacuo* before leaving the oil to dry on high vacuum for 1 hour. Charged the flask with **18** (0.356 g, 0.540 mmol, 1 equiv.) and Pd(dppf)$Cl_2$•$CH_2Cl_2$ (0.04 g, 0.048 mmol, 0.090 equiv.), under positive nitrogen flow. Backfilled and evacuated the system (3 cycles x 4 min) before dissolving the solids in dry 1,4-dioxane (4 mL). The reaction vessel was lowered into a pre-heated oil bath set to 80 °C, the flask was left to equilibrate to temperature over the course of 10 minutes, and then sparged aq. $K_3PO_4$ (0.54 mL, 2 M) was added dropwise before the reaction was left to run overnight. An aliquot was taken to confirm all **20** starting material had been consumed (checked by TLC: $AlO_x$ N, 20% DCM:Hex). The reaction mixture was brought to r.t. before extracting the organics with ethyl acetate (20 mL x 3). Washed the organics with brine (20 mL x 2), dried over sodium sulfate, gravity filtered and then removed the solvent *in vacuo*. To remove residual solvent the crude material was placed on high vacuum for 30 minutes, redissolved in acetone before removing solvent *in vacuo*, then placed back on high vacuum for 30 minutes (x 3). **Purification and Characterization:** The material was purified by automated flash column chromatography (loaded in 2% DCM:Hex, $SiO_2$, gradient 2% DCM:Hex - 20% DCM:Hex) to yield fluffy, tangerine-orange solid **15** (0.933 g, 26% yield). $^1H$ NMR (600 MHz, Methylene Chloride-$d_2$) δ 7.97 (d, *J* = 3.8 Hz, 1H), 7.91, (dd, *J* = 7.9, 4.6 Hz, 3H), 7.74 (d, *J* = 7.5 Hz, 1H), 7.48 (d, *J* = 8.4 Hz, 2H), 7.36 (dd, *J* = 8.6, 3.3 Hz, 4H), 7.26 (t, *J* = 8.3 Hz, 4H), 6.83 (d, *J* = 3.8 Hz, 1H), 6.20 (d, *J* = 10.0 Hz, 2H), 6.11 - 6.00 (m, 6H), 1.01 - 0.93 (m, 36H), 0.71 - 0.61 (m, 24H); $^{13}C$ NMR (151 MHz, Methylene Chloride-$d_2$) δ 154.42, 153.24, 153.15, 146.45, 145.28, 144.99, 139.20, 136.67, 133.30, 133.25, 132.66, 132.26, 132.17, 131.72, 131.40, 129.45, 128.63, 128.60, 128.37, 127.94, 127.85, 127.77, 126.80, 126.40, 125.91, 124.66, 71.85, 71.68, 71.56, 70.13, 7.26, 7.21, 6.84, 6.81, 6.78, 6.76; $R_f$ = 0.5 ($AlO_x$ N, 20% DCM:Hex); IR (ATR) ṽ 2909 (w), 1080 (m), 723 (m); HRMS (MALDI) *m*/*z* calcd for $C_{64}H_{84}Cl_2N_2O_4S_2Si_4$: 1,190.4327 [M]+, found 1,190.4321 (2.5106 ppm).

**b. D-A[12]CPP Derivatives: BT[12]CPP (1), Thio[12]CPP (3), BT-2-thio[12]CPP (4), and BT-7-thio[12]CPP (5)**

**General Method to Prepare Tin(II) Chloride Dihydrochloride ($H_2SnCl_4$) Solution:** Flame dried a 25 mL Schlenk flask equipped with a stir bar and a glass stopper. Once cool charged the flask with $SnCl_2$ • 2 $H_2O$ (0.181 g, 0.802 mmol, 1 equiv.) before backfilling and evacuating the flask (3 cycles x 3 min). Dissolved the solid in dry THF (20 mL) before adding concentrated HCl (0.13 mL, 12 M) dropwise. Allowed the reaction to stir for at least one hour before use.

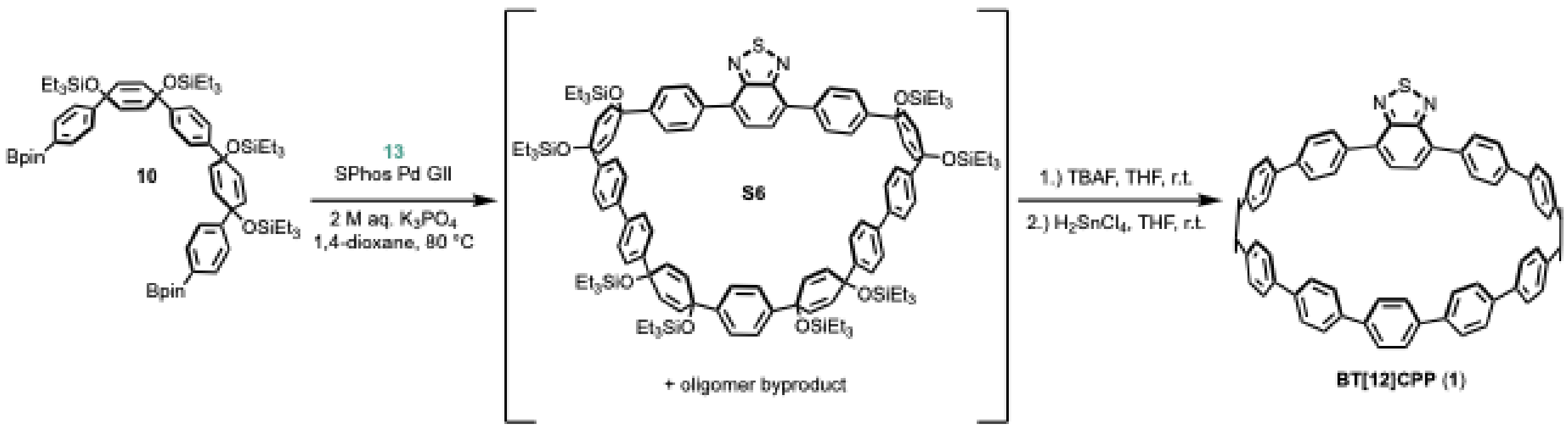


**Scheme S11.** Synthesis of non-isolated intermediate **S6** and target compound **1** (store **S6** and **1** in freezer for long term storage).

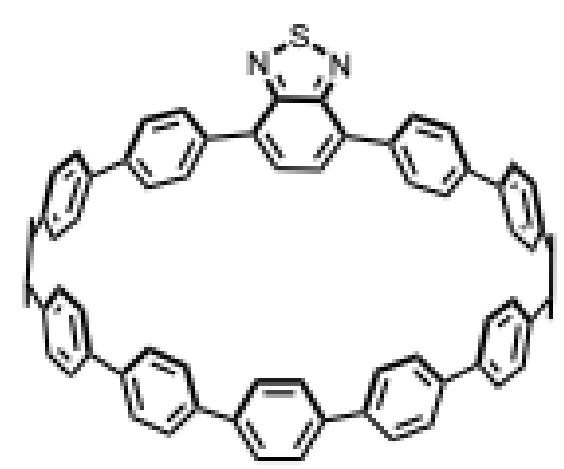

**S6. Macrocyclization:** Flame dried a 250 mL three-necked flask equipped with a stir bar, two glass stoppers, and a glass vacuum adapter. Once cool, charged the flask with **13** (0.10 g, 0.084 mmol, 1 equiv.), **10** (0.103 g, 0.089 mmol, 1.05 equiv.), and SPhos Pd GII (6.0 mg, 8.3 µmol, 0.1 equiv.). Backfilled and evacuated the system (3 cycles x 3 min) before dissolving the solid in dry 1,4-dioxane (87 mL). Lowered the reaction vessel into a pre-heated oil bath set to 80 °C, let the reaction equilibrate to temperature over the course of 15 minutes before adding sparged, aq. $K_3PO_4$ (4.21 mL, 2 M). Left the reaction to run overnight. Took an aliquot of the reaction mixture (checked by TLC: $SiO_2$, 30% DCM:Hex) to reveal **13** starting material was still present. Brought the flask to room temperature and sparged the mixture with nitrogen for 10 minutes before adding **10** (0.103 g, 0.089 mmol, 1.05 equiv.) and SPhos Pd GII (6.0 mg, 8.3 µmol, 0.1 equiv.). Sparged the reaction mixture with nitrogen for another 30 minutes before allowing it to run for another 3 hours. A second aliquot of the reaction mixture (checked by TLC: $SiO_2$, 30% DCM:Hex) showed all **13** had been consumed. The reaction was worked up the next day by bringing the flask to room temperature before removing excess solvent *in vacuo*. The organics were extracted with ethyl acetate (10 mL x 3). The organics were washed with brine (10 mL x 2), dried over sodium sulfate, gravity filtered, and excess solvent was removed *in vacuo*. The material was partially purified by column chromatography (loaded in hexanes, $SiO_2$, gradient 100% Hex - 20% DCM:Hex) to yield a sticky, green oil that was pushed forward without further purification.

**1.Triethylsilyl deprotection and reductive aromatization:** Using a syringe and needle, backfilled and evacuated (3 cycles x 3 min) a septum capped, pre-weighed, 20 mL scintillation vial containing a stir bar and partially pure **S6** (~0.191 g, 0.094 mmol, 1 equiv.). Dissolved the

material in dry THF (11.8 mL). To the flask added TBAF (0.76 mL, 1 M in THF) dropwise and left the reaction to run for 1 hour before quenching the reaction mixture with DI water (5 mL). Removed organic solvent *in vacuo*, collected the precipitated solid by vacuum filtration, and redissolved remaining solid in acetone (x 3). The crude solid was left out at room temperature, overnight, to dry. No further purification was attempted.

Using a syringe and needle, backfilled and evacuated (3 cycles x 3 min) a septum capped, pre-weighed, 20 mL scintillation vial containing a stir bar and the crude solid (~0.118 g, 0.107 mmol, 1 equiv.) from the previous step. Dissolved the solid in dry THF (9 mL) before adding a $H_2SnCl_4$ solution (11 mL, 0.04 M in THF) dropwise. Let the reaction run for 1 hour before adding DI water (5 mL) and extracting the organics with DCM (15 mL x 3). Washed the organics with brine (15 mL x 3), dried over sodium sulfate, gravity filtered the mixture, and then removed the solvent *in vacuo*. To remove residual solvent the crude material was placed on high vacuum for 30 minutes, redissolved in acetone before removing solvent *in vacuo*, then placed back on high vacuum for 30 minutes (x 3). **Purification and Characterization:** The material was purified by column chromatography (loaded in 10% DCM:Hex, pH 10 $AlO_x$, gradient 10% DCM:Hex - 50% DCM:Hex) and residual grease was removed via trituration in diethyl ether to yield yellow solid **1** (2.5 mg, 2% yield). $^1$H NMR (600 MHz, Methylene Chloride-*d₂*) δ 8.02 (d, *J* = 8.7 Hz, 4H), 7.70 (dd, *J* = 8.8, 2.6 Hz, 8H), 7.65 - 7.64 (m, 32H), 7.57 (s, 2H); $^{13}$C NMR (151 MHz, Methylene Chloride-$d_2$) δ 154.65, 140.14, 139.17, 138.92, 138.88, 138.83, 138.62, 136.80, 131.68, 130.57, 128.81, 127.77, 127.74, 127.70, 127.67, 127.65, 127.39; $R_f$ = 0.41 ($AlO_x$ N, 50% DCM:Hex); HRMS (ESI, positive mode) *m*/*z* calcd for $C_{72}H_{47}N_2S$: 971.3460 [M+H]+, found 971.3459 (-0.2 ppm).

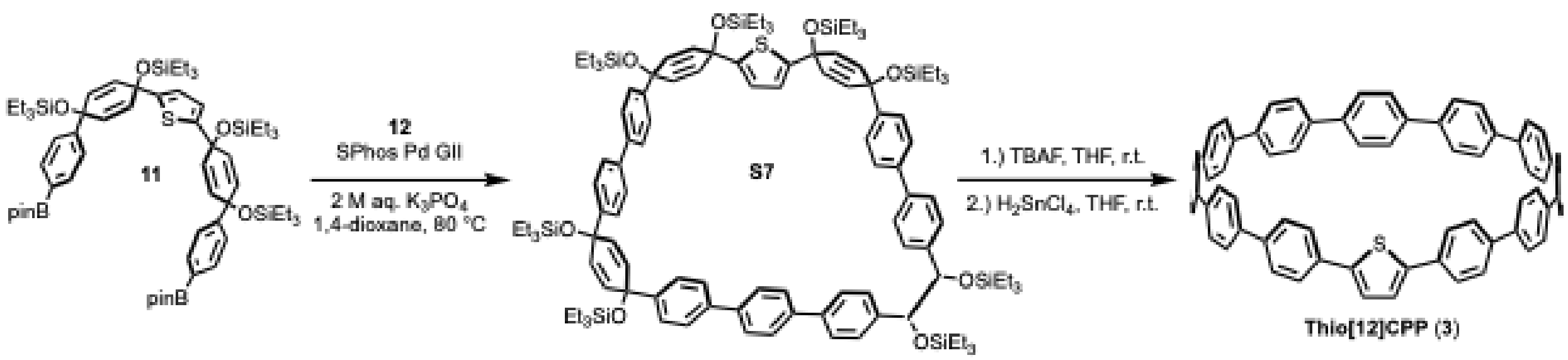


**Scheme S12.** Synthesis of intermediate **S7** and target compound **3** (store **S7** and **3** in freezer for long term storage)..

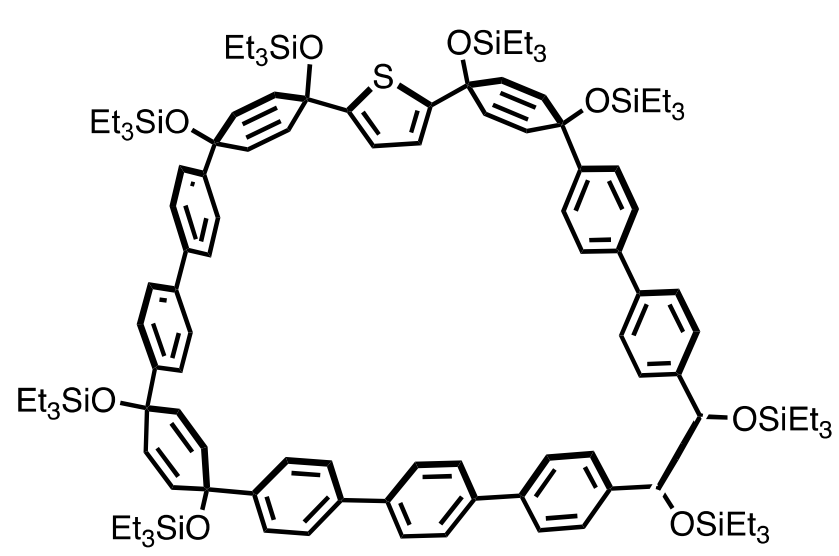


**S7. Macrocyclization:** Flame dried a 250 mL three-necked flask equipped with a stir bar, two glass stoppers, and a glass vacuum adapter. Charged the flask with **11** (0.126 g, 0.108 mmol, 1.05 equiv.), **12** (0.116 g, 0.103 mmol, 1 equiv.), and SPhos Pd GII (7.0 mg, 1.03 µmol, 0.1 equiv.) before backfilling and evacuated the system (3 cycles x 3 min). Exchanged a glass stopper for a septum under positive nitrogen flow before the solids were dissolved in dry 1,4-dioxane (102.8 mL). The reaction mixture was sparged with nitrogen for an hour. The flask was lowered into a pre-heated oil bath set to 80 °C and left to equilibrate for 30 minutes. Sparged, aq. $K_3PO_4$ (5.14 mL, 2 M) was added dropwise into the reaction vessel, which was then stirred overnight. An

aliquot of the reaction mixture was taken to monitor the consumption of starting material (checked by TLC: $AlO_x$ N, 15% DCM:Hex). The reaction mixture was brought to room temperature before removing excess solvent *in vacuo*. The organics were extracted with ethyl acetate (20 mL x 3). The organics were washed with brine (20 mL x 2), dried over sodium sulfate, gravity filtered, and excess solvent was removed *in vacuo*. **Purification and Characterization:** The material was purified by column chromatography (loaded in hexanes, pH 10 $AlO_x$, gradient 2% Hex - 24% DCM:Hex), and triturated in EtOH, then pentane to give white solid **S7** (0.55 mg, 28% yield). $^1$H NMR (600 MHz, Methylene Chloride-$d_2$) δ 7.58 (s, 4H), 7.47 - 7.43 (m, 12H), 7.40 (d, *J* = 8.4 Hz, 4H), 7.33 (dd, *J* = 8.4, 6.6 Hz, 8H), 6.48 (s, 2H), 6.13 (dd, *J* = 18.8, 10.1 Hz, 8H), 6.04 (d, *J* = 10.1 Hz, 4H), 5.94 (d, *J* = 10.1 Hz, 4H), 1.00 - 0.90 (m, 72H), 0.70 - 0.56 (m, 48H); $^{13}$C NMR (151 MHz, Methylene Chloride-$d_2$) δ 152.19, 145.42, 145.27, 145.18, 140.28, 139.92, 139.87, 139.68, 132.39 (broad), 132.29, 131.87, 130.80 (broad), 127.60, 127.13, 126.94, 126.81, 126.77, 126.41, 122.25, 72.21, 71.95, 71.84, 69.67, 7.24, 7.20, 7.17, 6.80, 6.80, 6.73, 6.63; $R_f$ = 0.47 ($AlO_x$ N, 15% DCM:Hex); IR (KBr) ṽ 2957 (m), 2874 (m), 1080 (s), 1005 (m), 962 (m), 815 (m), 723 (s); HRMS (MALDI) *m/z* calcd for $C_{118}H_{166}O_8SSi_8$: 1,967.0458 [M]+, found 1,967.0452 (3.8475 ppm).

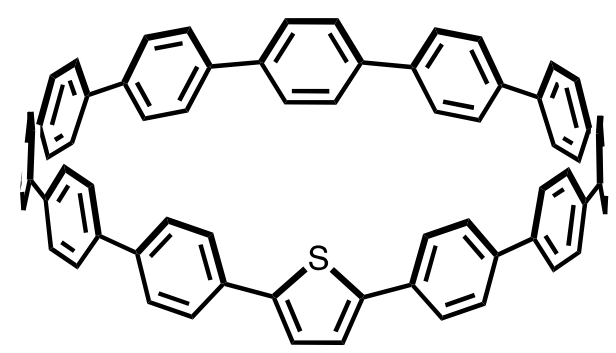


**3. Triethylsilyl deprotection and reductive aromatization:** Using a syringe and needle, backfilled and evacuated (3 cycles x 3 min) a septum capped, pre-weighed, 20 mL scintillation vial containing a stir bar and partially pure **S7** 54.8 mg, 0.028 mmol, 1 equiv.). Dissolved the material in dry THF (3.48 mL). To the flask added TBAF (0.24 mL, 1 M in THF) dropwise and left the reaction to run for 30 minutes. Transferred the contents to a round bottom flask before quenching the reaction mixture with DI water (10 mL). Removed organic solvent *in vacuo*, collected the precipitated solid by vacuum filtration, and redissolved remaining solid in acetone (x 2). The crude solid was stored in the fridge until further use - no further purification was attempted.

Using a syringe and needle, backfilled and evacuated (3 cycles x 3 min) a septum capped, pre-weighed, 20 mL scintillation vial containing a stir bar and the crude solid (0.028 g, 0.03 mmol, 1 equiv.) from the previous step. Dissolved the solid in dry THF (7.5 mL) before adding a $H_2SnCl_4$ solution (4.5 mL, 0.04 M in THF) dropwise. Let the reaction run for 1 hour before removing excess solvent *in vacuo*. The organics were extracted with DCM (10 mL x 3). Washed the organics with brine (10 mL x 3), dried over sodium sulfate, gravity filtered the mixture, and then removed the solvent *in vacuo*. **Purification and Characterization:** The material was purified by column chromatography (loaded in hexanes, pH 10 $AlO_x$, gradient 0% Hex - 30% DCM:Hex) and triturated in pentane to remove excess grease to yield light yellow solid **3** (1.9 mg, 7% yield). $^1$H NMR (600 MHz, Methylene Chloride-$d_2$) δ 7.66 - 7.62 (m, 36H), 7.59 (d, *J* = 8.6 Hz, 4H), 7.51 (d, *J* = 8.6 Hz, 4H), 7.27 (s, 2H); $^{13}$C NMR (151 MHz, Methylene Chloride-$d_2$) δ 146.98, 139.61, 139.05, 139.01, 138.92, 138.91, 138.89, 138.88, 138.84, 138.83, 133.88, 127.79, 127.73, 127.70, 127.67, 127.38, 123.24; $R_f$ = 0.33 ($AlO_x$ N, 35% DCM:Hex); IR (KBr) ṽ 2924 (s), 2853 (s), 1734 (w), 1669 (w), 1594 (w), 1484.61 (m), 1464.33 (m), 1378 (s), 1262 (m), 1095 (m), 1027 (m), 1001 (s), 860 (s), 809 (s), 744 (w), 668 (s), 510.26 (w); HRMS (ESI, positive mode) *m/z* calcd for $C_{70}H_{47}S$: 919.3398 [M+H]+, found 919.3390 (-0.9 ppm).

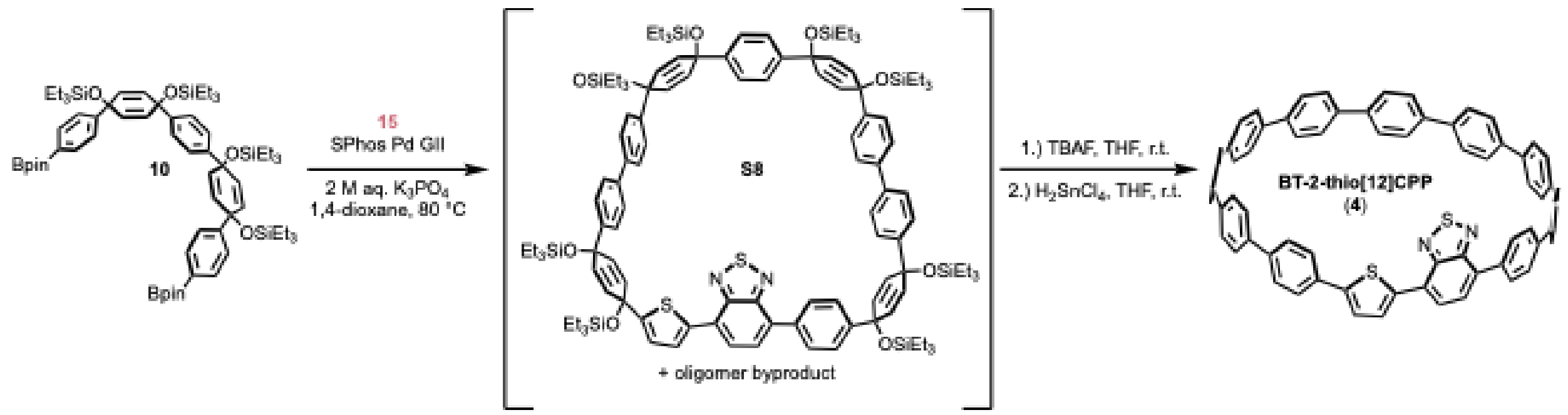


**Scheme S13.** Synthesis of intermediate **S8** and target compound **4** (store **S8** and **4** in freezer for long term storage)..

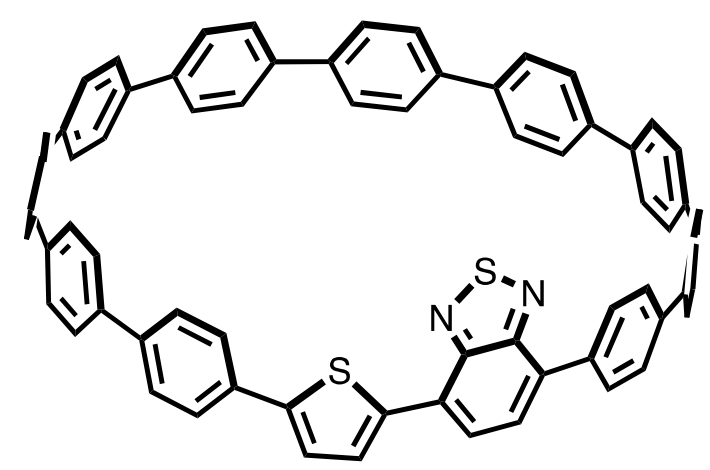

**S8. Macrocyclization:** Flame dried a 250 mL three-necked flask equipped with a stir bar, two glass stoppers, and a glass vacuum adapter. Once cool, charged the flask with **15** (0.217 g, 0.182 mmol, 1 equiv.) dissolved in DCM. Removed the solvent *in vacuo* and left the material on high vacuum for 40 minutes. Under positive nitrogen flow added **10** (0.201 g, 0.173 mmol, 0.95 equiv.), and SPhos Pd GII (0.014 g, 0.018 mmol, 0.1 equiv.). Backfilled and evacuated the system (3 cycles x 3 min), exchanged a glass stopper for a septum under positive nitrogen flow before dissolving the solid in dry 1,4-dioxane (182 mL). Sparged the reaction mixture for 30 minutes. Lowered the reaction vessel into a pre-heated oil bath set to 80 °C before adding sparged, aq. $K_3PO_4$ (18.2 mL, 2 M). Left the reaction to run overnight. Took an aliquot of the reaction mixture (checked by TLC: $AlO_x$ N, 20% DCM:Hex) to reveal the reaction had progressed yet side products were noticeably present. The reaction was worked up by bringing the flask to room temperature before removing excess solvent *in vacuo*. The organics were extracted with ethyl acetate (20 mL x 3). The organics were washed with brine (15 mL x 3), dried over sodium sulfate, gravity filtered, and excess solvent was removed *in vacuo*. The material was partially purified by automated column chromatography (loaded in hexanes, pH 10 $AlO_x$, gradient 100% Hex - 40% DCM:Hex) to yield a sticky, green-yellow oil that was pushed forward without further purification.

**4.Triethylsilyl deprotection and reductive aromatization:** Using a syringe and needle, backfilled and evacuated (3 cycles x 3 min) a septum capped, pre-weighed, 20 mL scintillation vial containing a stir bar and partially pure **S8** (~0.085 g, 0.0418 mmol, 1 equiv.). Dissolved the material in dry THF (5.5 mL). To the flask added TBAF (0.35 mL, 1 M in THF) dropwise and left the reaction to run for 1 hour before quenching the reaction mixture with DI water (2 mL). Removed organic solvent *in vacuo*, collected the precipitated solid by vacuum filtration, and redissolved remaining solid in acetone (x 3). The crude solid was stored in the fridge overnight – no further purification was attempted.

Using a syringe and needle, backfilled and evacuated (3 cycles x 3 min) a septum capped, pre-weighed, 20 mL scintillation vial containing a stir bar and the crude solid (~0.047 g, 0.0418 mmol, 1 equiv.) from the previous step. Dissolved the solid in dry THF (6 mL) before adding a $H_2SnCl_4$ solution (4.5 mL, 0.04 M in THF) dropwise. Let the reaction run for 1 hour before adding DI water (5 mL) and extracting the organics with DCM (15 mL x 3). Washed the organics with brine (15 mL

x 3), dried over sodium sulfate, gravity filtered the mixture, and then removed the solvent *in vacuo*. **Purification and Characterization:** The material was purified by column chromatography (dry loaded, pH 10 $AlO_x$, 0% Hex - 50% Hex:DCM) and residual grease was removed via trituration in pentane to yield orange solid **4** (1.0 mg, 2% yield). $^1H$ NMR (600 MHz, Methylene Chloride-$d_2$) δ 8.13 (d, $J$ = 3.9 Hz, 1H), 7.98 (d, $J$ = 8.7 Hz, 2H), 7.77 (d, $J$ = 7.7 Hz, 1H), 7.74 - 7.68 (m, 4H), 7.67 - 7.60 (m, 32H), 7.57 - 7.52 (m, 3H), 7.39 (d, $J$ = 3.9 Hz, 1H); $^{13}C$ NMR (151 MHz, Methylene Chloride-$d_2$) δ 154.14, 154.06, 147.52, 142.13, 140.11, 139.93, 139.25, 139.11, 139.02, 138.96, 138.95, 138.92, 138.90, 138.87, 138.83, 138.78, 138.58, 136.84, 133.59, 132.56, 130.73, 129.41, 129.10, 127.82, 127.81, 127.77, 127.72, 127.68, 127.63, 127.52, 127.34, 126.62, 126.01, 123.65; $R_f$ = 0.33 ($AlO_x$ N, 50% DCM:Hex); HRMS (ESI, positive mode) $m/z$ calcd for $C_{70}H_{45}N_2S_2$: 977.3024 [M+H]+, found 977.3037 (1.3 ppm).

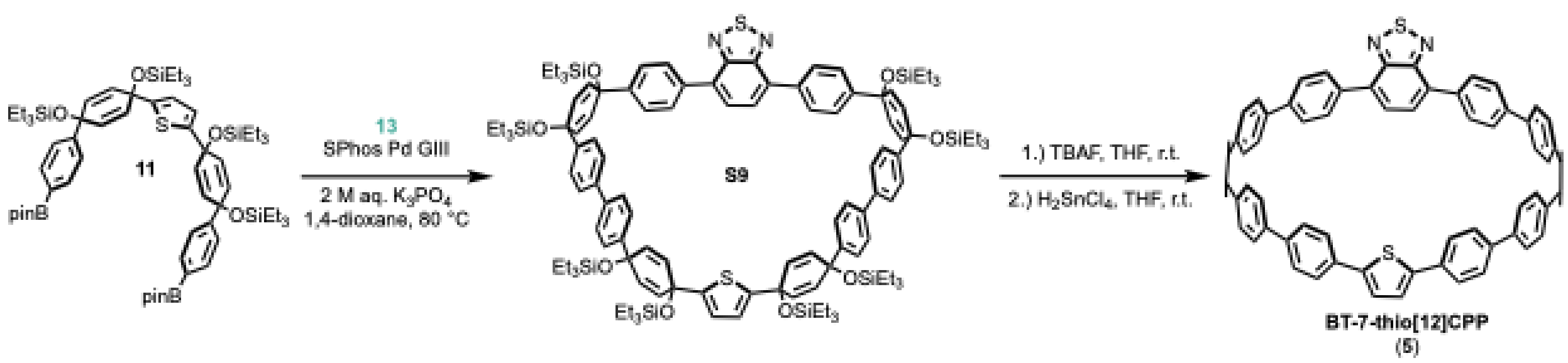


**Scheme S14.** Synthesis of intermediate **S9** and target compound **5** (store **S9** and **5** in freezer for long term storage).

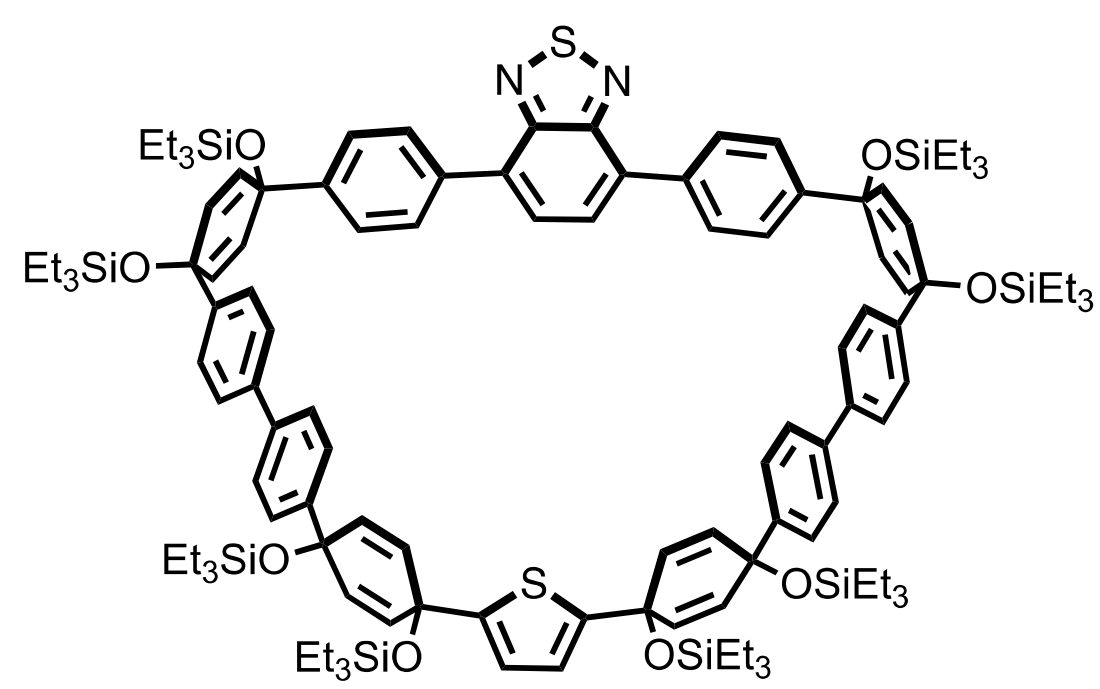


**S9. Macrocyclization:** Flame dried a 500 mL round bottomed flask, plus a 1 L round bottomed flask equipped with a stir bar and glass vacuum adapter. To the 500 mL round bottomed flask added dry 1,4-dioxane and sparged the solvent for 1 hour. Backfilled and evacuated (3 cycles x 2 min) the 1 L round bottomed flask before charging the flask with **13** (0.550 g, 0.463 mmol, 1 equiv.), **11** (0.594 g, 0.510 mmol, 1.1 equiv.), and SPhos Pd GIII (0.037 g, 0.0463 mmol, 0.10 equiv.). Backfilled and evacuated (3 cycles x 5 min) the reaction vessel before quickly exchanging the glass vacuum adapter for a septum. Cannula transferred the contents of the 500 mL round bottomed flask to the 1 L reaction vessel, then sparged the reaction mixture for 15 minutes. The flask was lowered into a pre-heated oil bath set to 80 °C and left to equilibrate for 15 minutes. Sparged, aq. $K_3PO_4$ (46.4 mL, 2 M) was added dropwise into the reaction vessel, which was then stirred overnight. The reaction was brought to room temperature before the material was poured through a Celite plug and rinsed with DCM. The filtrate was dried over sodium sulfate, gravity filtered, and excess organic solvent was removed *in vacuo*. To remove residual solvent the crude material was placed on high vacuum for 30 minutes, redissolved in acetone before removing solvent *in vacuo*, then placed back on high vacuum for 30 minutes (x 4). **Purification and Characterization:** The material was purified by column chromatography (loaded in 10% DCM:Hex, pH 10 $AlO_x$, gradient 10% DCM:Hex - 20% DCM:Hex) to give fluffy, neon green solid

**S9** (0.279 g, 30% yield). $^1$H NMR (600 MHz, Methylene Chloride-*d$_2$*) δ 7.79 (d, *J* = 8.5 Hz, 4H), 7.68 (s, 2H), 7.48 (d, *J* = 8.6 Hz, 4H), 7.45 - 7.40 (m, 12H), 7.33 (d, *J* = 8.5 Hz, 4H), 6.46 (s, 2H), 6.16 (t, *J* = 9.8 Hz, 8H), 6.08 (d, *J* = 10.2 Hz, 4H), 5.94 (d, *J* = 10.1 Hz, 4H), 1.03 - 0.91 (m, 72H), 0.75 - 0.57 (m, 48H); $^{13}$C NMR (151 MHz, Methylene Chloride-*d$_2$*) δ 154.46, 152.14, 146.26, 145.47, 145.26, 140.36, 139.97, 136.68, 133.13, 132.35, 132.00, 130.89, 129.34, 128.37, 127.19, 127.15, 127.06, 126.48, 126.45, 122.37, 72.39, 72.03, 71.87, 69.74, 7.32, 7.28, 7.25, 7.22, 6.90, 6.89, 6.81, 6.71; $R_f$ = 0.25 ($AlO_x$ N, 20% DCM:Hex); HRMS (MALDI) *m/z* calcd for $C_{118}H_{164}N_2O_8S_2Si_8$: 2,025.0083 [M]+, found 2,025.0078 (2.5552 ppm).

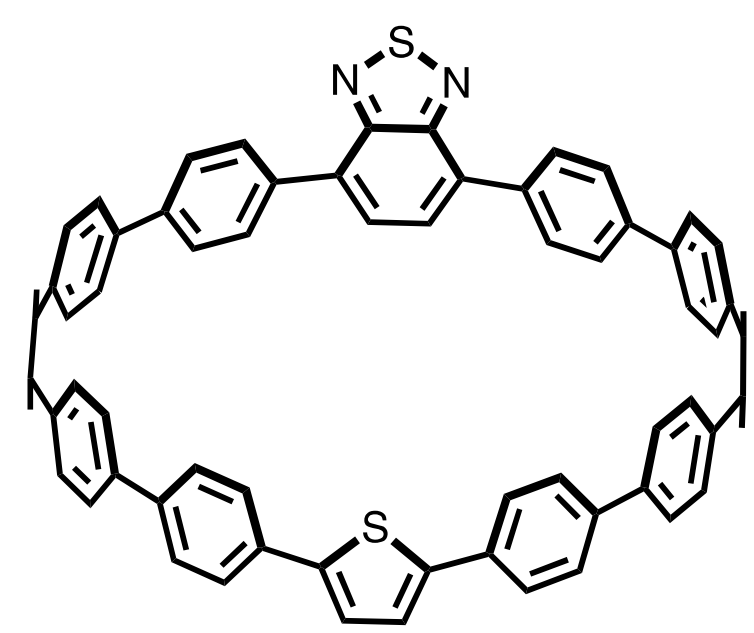


**5. Triethylsilyl deprotection and reductive aromatization:** Using a syringe and needle, backfilled and evacuated (3 cycles x 3 min) a septum capped, pre-weighed, 20 mL scintillation vial containing a stir bar and **S9** (0.109 g, 0.054 mmol, 1 equiv.). Dissolved the material in dry THF (6.75 mL). To the flask added TBAF (0.48 mL, 1 M in THF) dropwise and left the reaction to run for 1 hour. Quenched the reaction mixture with DI water (2 mL). Removed organic solvent *in vacuo*, collected the precipitated solid by vacuum filtration, and redissolved remaining solid in acetone (x 3). The crude solid was stored in the fridge until further use - no further purification was attempted.

Flame dried a 25 mL round bottomed flask equipped with a stir bar and glass vacuum adapter. Once cool, quickly transferred the crude material (0.039 g, 0.035 mmol, 1 equiv.) from the previous step into the reaction vessel. Backfilled and evacuated the flask (3 cycles x 3 min) before dissolving the solid in dry THF (8 mL). Added a $H_2SnCl_4$ solution (3.5 mL, 0.04 M in THF) dropwise. Let the reaction run for 1 hour before quenching the reaction with sat. sodium bicarbonate (5 mL). The organics were extracted with DCM (20 mL x 3). Washed the organics with brine (20 mL x 3), dried over sodium sulfate, gravity filtered the mixture, and then removed the solvent *in vacuo*. **Purification and Characterization:** The material was purified by column chromatography (dry loaded, pH 10 $AlO_x$, gradient 0% Hex - 50% DCM:Hex) and triturated in diethyl ether to yield light yellow solid **5** (13.0 mg, 38% yield). $^1$H NMR (500 MHz, Methylene Chloride-*d$_2$*) δ 8.03 (d, *J* = 8.6 Hz, 4H), 7.72 - 7.58 (m, 34H), 7.50 (d, *J* = 8.4 Hz, 4H), 7.26 (s, 2H); $^{13}$C NMR (151 MHz, Methylene Chloride-*d$_2$*) δ 154.69, 147.02, 140.10, 139.59, 139.19, 139.06, 139.02, 138.98, 138.84, 138.62, 136.81, 133.88, 131.64, 130.67, 128.77, 127.80, 127.77, 127.67, 127.64, 127.40, 127.38, 123.23; $R_f$ = 0.38 ($AlO_x$ N, 50% DCM:Hex); IR (ATR) ṽ 3024 (w), 1482 (w), 812 (m), 746 (w); HRMS (ESI, positive mode) *m/z* calcd for $C_{70}H_{45}N_2S_2$: 977.3024 [M+H]+, found 977.3016 (-0.8 ppm).

c. **D-A[10]CPP Derivatives: BT-m[10]CPP (2), BT-2-thio[10]CPP (6), and BT-2,2-dithio[10]CPP (7)**

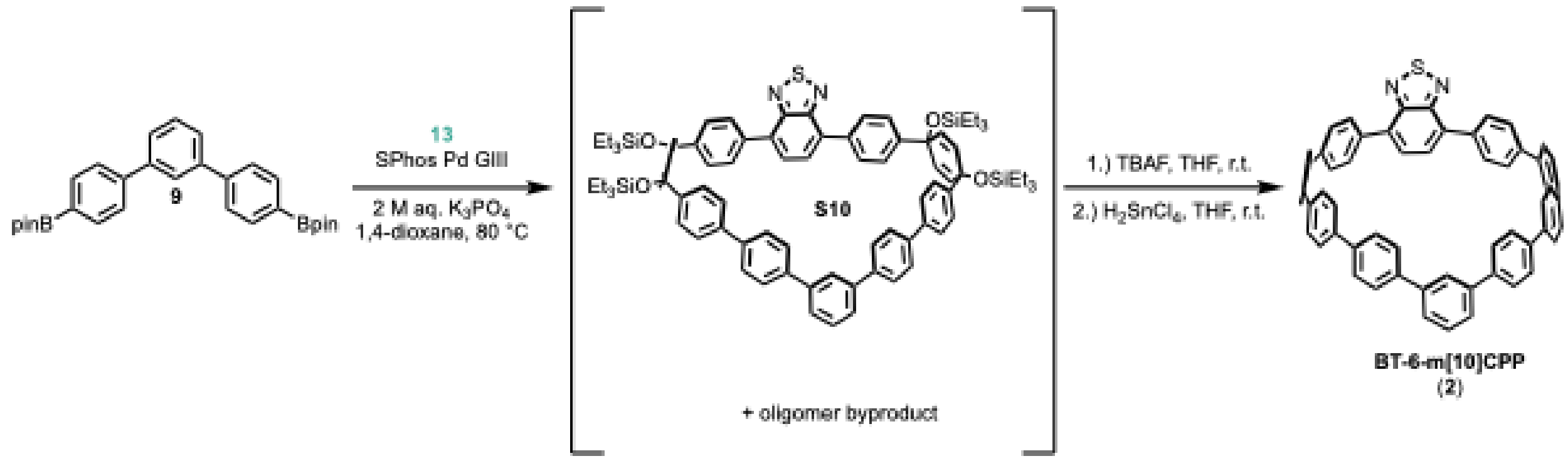


**Scheme S15.** Synthesis of intermediate **S10** and target compound **2** (store **S10** and **2** in freezer for long term storage).

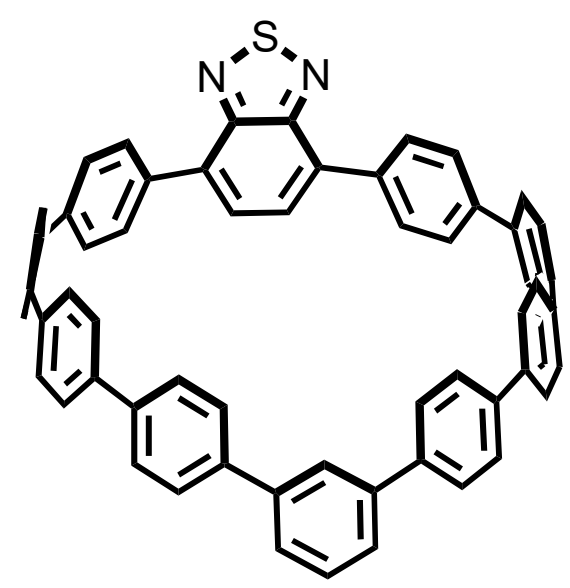

**S10. Macrocyclization:** Flame dried a 500 mL round bottomed flask equipped with a stir bar and a glass vacuum adapter. Once cool, quickly added **9** (0.229 g, 0.475 mmol, 1.01 equiv.), **13** (0.6 g, 0.470 mmol, 1 equiv.), and SPhos Pd GIII (0.037 g, 0.047 mmol, 0.1 equiv.). Exchanged the glass vacuum adapter for a septum, then cannula transferred dry 1,4-dioxane (392 mL) and sparged the reaction mixture for 1 hour. Lowered the reaction vessel into a pre-heated oil bath set to 80 °C and let equilibrate for 20 minutes. Added sparged, aq. $K_3PO_4$ (39.2 mL, 2 M). Left the reaction to run overnight. The reaction was worked up by bringing the flask to room temperature before removing excess solvent *in vacuo*. The organics were extracted with DCM (50 mL x 3). The organics were washed with brine (50 mL x 3), dried over sodium sulfate, gravity filtered, and excess solvent was removed *in vacuo*. The material was partially purified by automated column chromatography (loaded in 5% DCM:Hex, $SiO_2$, gradient 5% Hex - 45% DCM:Hex) to yield a sticky, green oil that was pushed forward without further purification.

**2.Triethylsilyl deprotection and reductive aromatization:** Flame dried a 15 mL round bottomed flask equipped with a stir bar and a glass vacuum adapter. Backfilled and evacuated (3 cycles x 3 min) the flask before adding partially pure **S10** (~0.05 g, 0.037 mmol, 1 equiv.). Backfilled and evacuated the flask (3 cycles x 3 min) a second time before dissolving the material in dry THF (5 mL). To the flask added TBAF (0.20 mL, 1 M in THF) dropwise and left the reaction to run for 1 hour before quenching the reaction mixture with DI water (5 mL). Collected the precipitated solid by vacuum filtration, rinsed the solid with DI water (5 mL), and allowed to dry over the course of 3 hours. The crude solid was stored in the fridge overnight - no further purification was attempted.

Flame dried a 15 mL round bottomed flask equipped with a stir bar and a glass vacuum adapter. Backfilled and evacuated (3 cycles x 3 min) the flask before adding the crude solid (~0.011 g,

0.0123 mmol, 1 equiv.) from the previous step. Backfilled and evacuated the flask (3 cycles x 3 min) a second time before dissolving the solid in dry THF (4 mL). To the flask added $H_2SnCl_4$ solution (0.62 mL, 0.04 M in THF) dropwise. Let the reaction run for 1 hour before adding sat. sodium bicarbonate (2 mL). Removed excess solvent *in vacuo* before extracting the organics with DCM (15 mL x 3). Washed the organics with brine (15 mL x 3), dried over sodium sulfate, gravity filtered the mixture, and then removed the solvent *in vacuo*. **Purification and Characterization:** The material was purified by column chromatography (wet loaded 30% DCM:Hex, pH 10 $AlO_x$, 30% DCM:Hex - 100% DCM) to yield yellow solid **2** (2.0 mg, 22% yield). $^1$H NMR (500 MHz, Methylene Chloride-$d_2$) δ 7.98 (d, $J$ = 8.9 Hz, 4H), 7.64 - 7.60 (m, 19H), 7.56 - 7.53 (m, 8H), 7.51 (s, 2H), 7.44 (d, $J$ = 8.4 Hz, 4H), 6.84 (broad s, 1H); $^{13}$C NMR (126 MHz, Methylene Chloride-$d_2$) δ 7 154.87, 142.75, 141.60, 140.12, 139.82, 139.67, 138.73, 138.65, 138.62, 136.77, 133.88, 131.57, 131.10, 129.50, 128.93, 128.32, 127.97, 127.82, 127.65, 127.56, 127.53, 124.59; $R_f$ = 0.33 ($AlO_x$ N, 50% DCM:Hex); HRMS (ESI, positive mode) $m/z$ calcd for $C_{60}H_{38}N_2S$: 818.2756 [M]+, found 818.2782 (3.2 ppm).

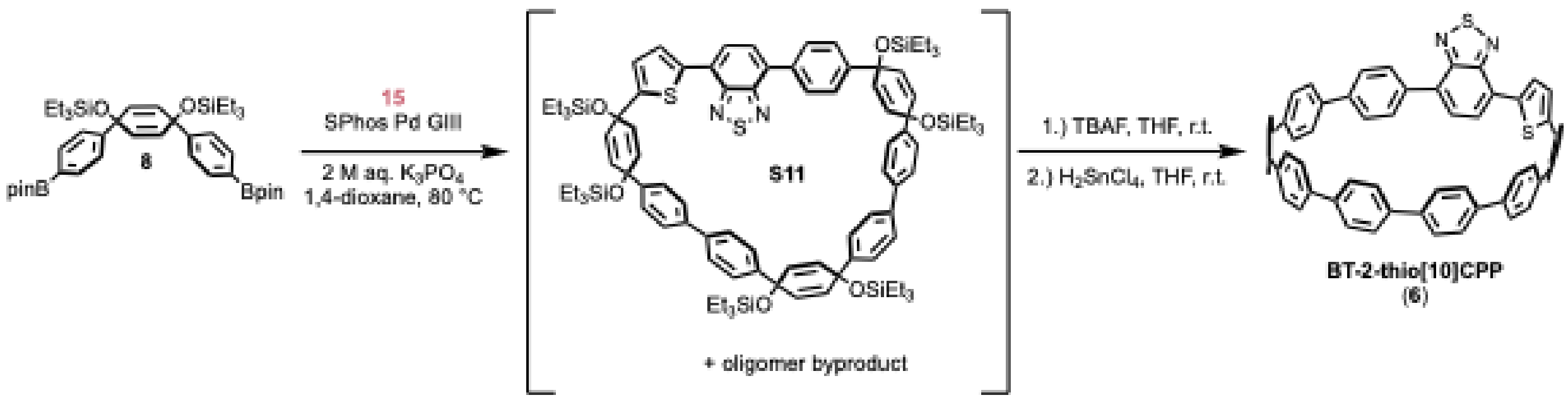


**Scheme S16.** Synthesis of intermediate **S11** and target compound **6** (store **S11** and **6** in freezer for long term storage).

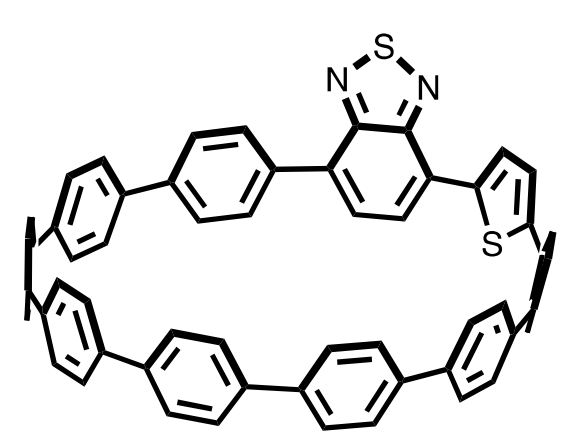

**S11. Macrocyclization:** Flame dried a 100 mL three-necked flask equipped with a stir bar, two glass stopers, and a glass vacuum adapter. Under positive nitrogen flow added **15** (0.049 g, 0.041 mmol, 1 equiv.) dissolved in DCM, removed the solvent *in vacuo*, and left on high vacuum for 1 hour. To the flask then added **8** (0.032 g, 0.043 mmol, 1.05 equiv.) and SPhos Pd GIII (3.2 mg, 4.02 μmol, 0.1 equiv.). Backfilled and evacuated the system (3 cycles x 3 min). Exchanged the glass vacuum adapter for a septum, then dissolved the solids in dry 1,4-dioxane (43 mL). Lowered the reaction vessel into a pre-heated oil bath set to 80 °C and let equilibrate for 15 minutes. Added sparged, aq. $K_3PO_4$ (4.09 mL, 2 M). Left the reaction to run overnight. The reaction was worked up by bringing the flask to room temperature before removing excess solvent *in vacuo*. The organics were extracted with DCM (20 mL x 3). The organics were washed with brine (20 mL x 3), dried over sodium sulfate, gravity filtered, and excess solvent was removed *in vacuo*. The material was partially purified by automated column chromatography (dry loaded, pH 10 $AlO_x$, gradient 100% Hex - 40% DCM:Hex) to yield a sticky, orange oil that was pushed forward without further purification.

**6.Triethylsilyl deprotection and reductive aromatization:** Flame dried a 15 mL heart shaped flask equipped with a stir bar, a glass stopper, and a glass vacuum adapter. Added partially pure

**S11** (~0.084 g, 0.052 mmol, 1 equiv.) and backfilled and evacuated the flask (3 cycles x 3 min) before dissolving the material in dry THF (6.53 mL). To the flask added TBAF (0.31 mL, 1 M in THF) dropwise and left the reaction to run for 1 hour before quenching the reaction mixture with DI water (1.5 mL). Collected the precipitated solid by vacuum filtration and allowed to dry over the course of 35 minutes. The crude solid was stored in the fridge overnight - no further purification was attempted.

Connected a 15 mL heart shaped flask - equipped with a stir bar, a glass stopper, and a glass vacuum adapter - containing the crude solid (0.048 g, 0.052 mmol, 1 equiv.) from the previous step onto the Schlenk line. Backfilled and evacuated the system (3 cycles x 3 min) before dissolving the solid in dry THF (4 mL). Added $H_2SnCl_4$ solution (4.3 mL, 0.04 M in THF) dropwise. Let the reaction run for 1 hour before quenching with sat. sodium bicarbonate (4 mL). Remove the solvent *in vacuo*, then extracted the organics with DCM (20 mL x 3). Washed the organics with brine (20 mL x 3), dried over sodium sulfate, gravity filtered the mixture, and then removed the solvent *in vacuo*. To remove residual solvent the crude material was placed on high vacuum for 30 minutes, redissolved in acetone before removing solvent *in vacuo*, then placed back on high vacuum for 30 minutes (x2). **Purification and Characterization:** The material was purified by column chromatography (wet loaded 20% DCM:Hex, pH 10 $AlO_x$, 20% DCM:Hex - 40% DCM) and triturated in diethyl ether to remove residual grease to yield orange solid **6** (1.0 mg, 2% yield). $^1$H NMR (600 MHz, Methylene Chloride-$d_2$) δ 8.03 (d, $J$ = 3.9 Hz, 1H), 7.92 (d, $J$ = 8.7 Hz, 2H), 7.70 (d, $J$ = 7.8 Hz, 1H), 7.71 - 7.56 (m, 28H), 7.47 - 7.45 (m, 3H), 7.32 (d, $J$ = 3.9 Hz, 1H); $^{13}$C NMR (151 MHz, Methylene Chloride-$d_2$) δ 154.53, 154.00, 148.79, 143.07, 139.99, 139.68, 138.98, 138.92, 138.71, 138.60, 138.58, 138.54, 138.52, 138.47, 138.21, 136.68, 133.54, 132.51, 131.28, 129.17, 128.80, 127.83, 127.80, 127.76, 127.74, 127.71, 127.67, 127.64, 127.39, 126.56, 125.85, 123.31; $R_f$ = 0.57 ($AlO_x$ N, 50% DCM:Hex); HRMS (ESI, positive mode) $m/z$ calcd for $C_{58}H_{37}N_2S_2$: 825.2398 [M+H]+, found 825.2382 (-1.9 ppm).

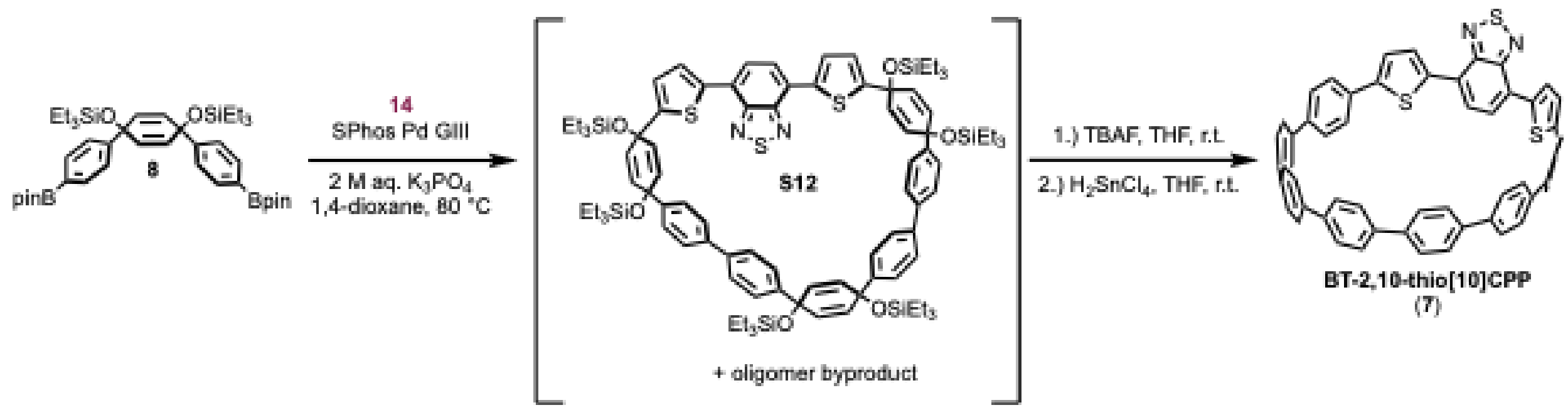


**Scheme S17.** Synthesis of intermediate **S12** and target compound **7** (store **S12** and **7** in freezer for long term storage).

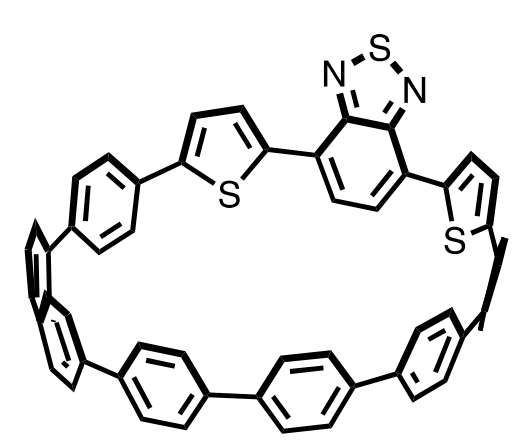

**S12. Macrocyclization:** Flame dried a 100 mL three-necked flask equipped with a stir bar, two glass stopers, and a glass vacuum adapter. Under positive nitrogen flow added **14** (0.05 g, 0.042 mmol, 1 equiv.), **8** (0.033 g, 0.044 mmol, 1.05 equiv.) and SPhos Pd GIII (3.0 mg, 3.89 μmol, 0.1 equiv.). Backfilled and evacuated the system (3 cycles x 3 min). Exchanged the glass vacuum adapter for a septum, then dissolved the solids in dry 1,4-dioxane (42 mL). Lowered the reaction

vessel into a pre-heated oil bath set to 80 °C and let equilibrate for 15 minutes. Added sparged, aq. $K_3PO_4$ (4.17 mL, 2 M). Left the reaction to run overnight. The reaction was worked up by bringing the flask to room temperature before removing excess solvent *in vacuo*. The organics were extracted with DCM (20 mL x 4). The organics were washed with brine (20 mL x 3), dried over sodium sulfate, gravity filtered, and excess solvent was removed *in vacuo*. To remove residual solvent the crude material was placed on high vacuum for 30 minutes, redissolved in acetone before removing solvent *in vacuo*, then placed back on high vacuum for 30 minutes (x2). The material was partially purified by automated column chromatography (loaded in hexanes, pH 10 $AlO_x$, gradient 100% Hex - 40% DCM:Hex) to yield a sticky, red oil that was pushed forward without further purification.

**7.Triethylsilyl deprotection and reductive aromatization:** To a septum capped, pre-weighed, 20 mL scintillation vial containing a stir bar, added partially pure **S11** (~0.019 g, 0.012 mmol, 1 equiv.) dissolved in DCM. Removed the solvent *in vacuo* and left the material on high vacuum for 1 hour. Dissolved the material in dry THF (1.5 mL). To the flask added TBAF (0.073 mL, 1 M in THF) dropwise and left the reaction to run for 30 minutes. Quenched the reaction mixture with DI water (3 mL). Removed organic solvent *in vacuo*, collected the precipitated solid by vacuum filtration, and allowed the material to dry for 30 minutes. The crude solid was stored in the fridge until further use - no further purification was attempted.

Flame dried a 15 mL heart shaped flask equipped with a stir bar, a glass stopper, and a glass vacuum adapter. Added partially pure **S12** (~8.8 mg, 9.43 μmol, 1 equiv.) and backfilled and evacuated the flask (3 cycles x 3 min) before dissolving the material in dry THF (5 mL). Added $H_2SnCl_4$ solution (0.78 mL, 0.04 M in THF) dropwise. Let the reaction run for 30 minutes before the solvent was removed *in vacuo*. Extracted the organics with DCM (20 mL x 3). Washed the organics with brine (20 mL x 3), dried over sodium sulfate, gravity filtered the mixture, and then removed the solvent *in vacuo*. To remove residual solvent the crude material was placed on high vacuum for 30 minutes, redissolved in acetone before removing solvent *in vacuo*, then placed back on high vacuum for 30 minutes (x2). **Purification and Characterization:** The material was purified by column chromatography (wet loaded 20% DCM:Hex, pH 10 $AlO_x$, 20% DCM:Hex - 40% DCM) to yield red solid **7** (4.0 mg, 27% yield). $^1$H NMR (600 MHz, Methylene Chloride-$d_2$) δ 7.99 (d, $J$ = 3.8 Hz, 2H), 7.67 (s, 2H), 7.61 - 7.57 (m, 24H), 7.49 (d, $J$ = 8.4 Hz, 4H), 7.31 (d, $J$ = 3.9 Hz, 2H); $^{13}$C NMR (151 MHz, Methylene Chloride-$d_2$) δ 153.72, 148.40, 143.01, 139.83, 138.90, 138.67, 138.58, 138.56, 138.46, 133.55, 129.08, 127.85, 127.82, 127.78, 127.76, 127.74, 126.70, 126.62, 123.29; $R_f$ = 0.35 ($AlO_x$ N, 40% DCM:Hex); HRMS (MALDI) *m/z* calcd for $C_{56}H_{34}N_2S_3$: 830.1884 [M]+, found 830.1879 (2.3901 ppm).

## 3. Nuclear Magnetic Resonance Spectra

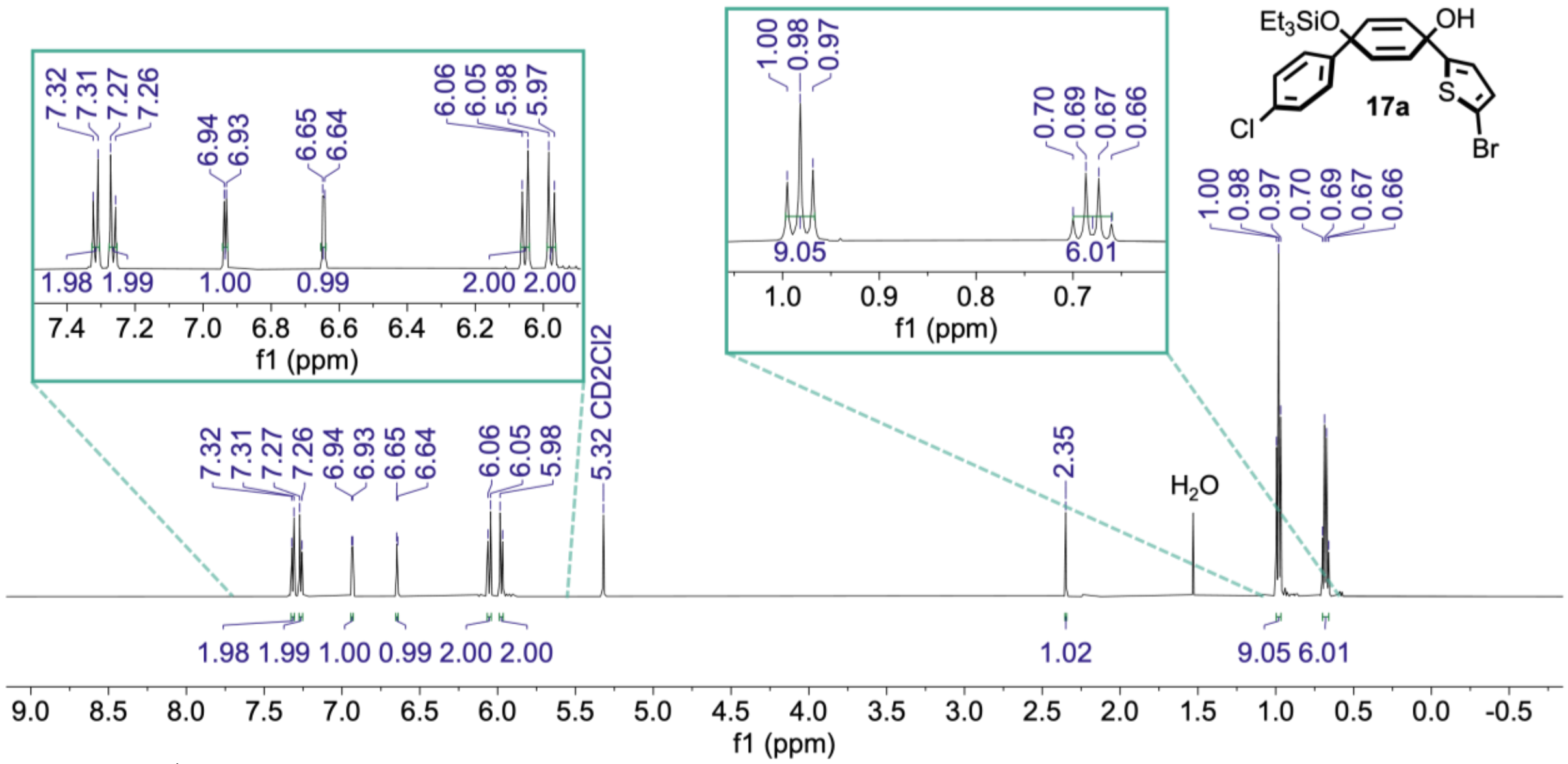


**Figure S1.** $^{1}$H NMR spectrum (600 MHz) of **17a** in Methylene Chloride-$d_2$ at room temperature.

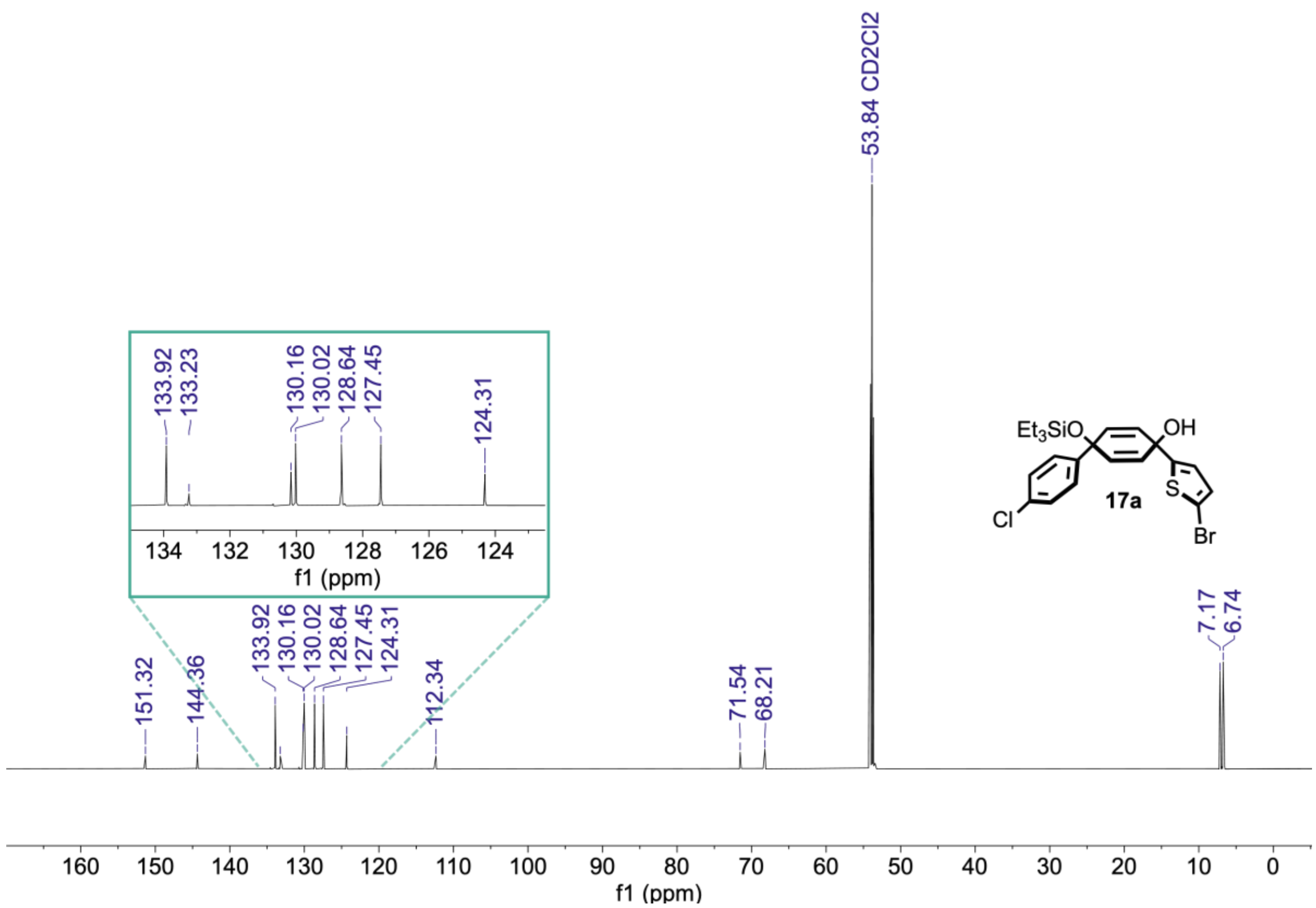


**Figure S2.** $^{13}$C NMR spectrum (151 MHz) of **17a** in Methylene Chloride-$d_2$ at room temperature.

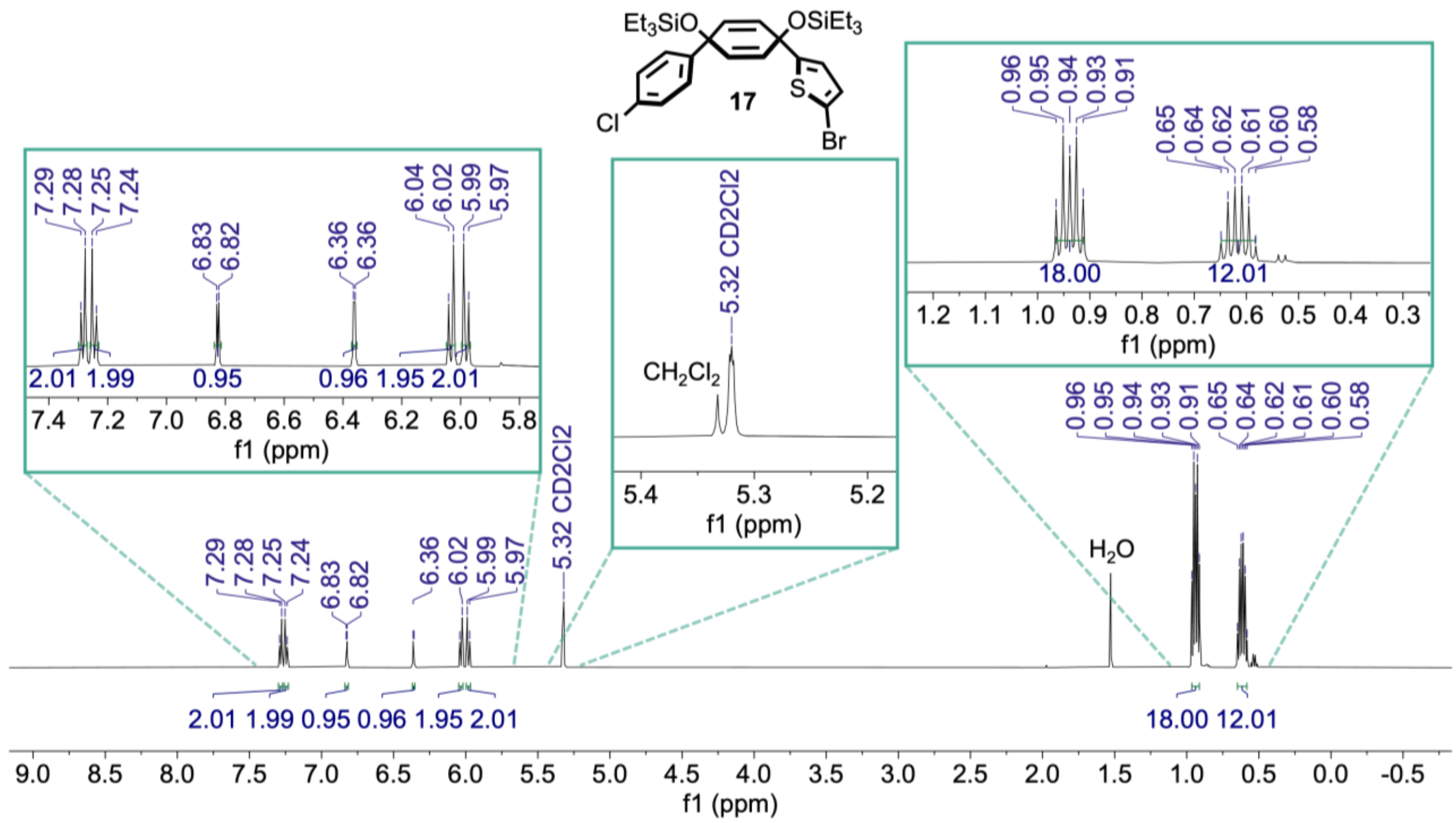


**Figure S3.** $^{1}H$ NMR spectrum (600 MHz) of **17** in Methylene Chloride-$d_2$ at room temperature.

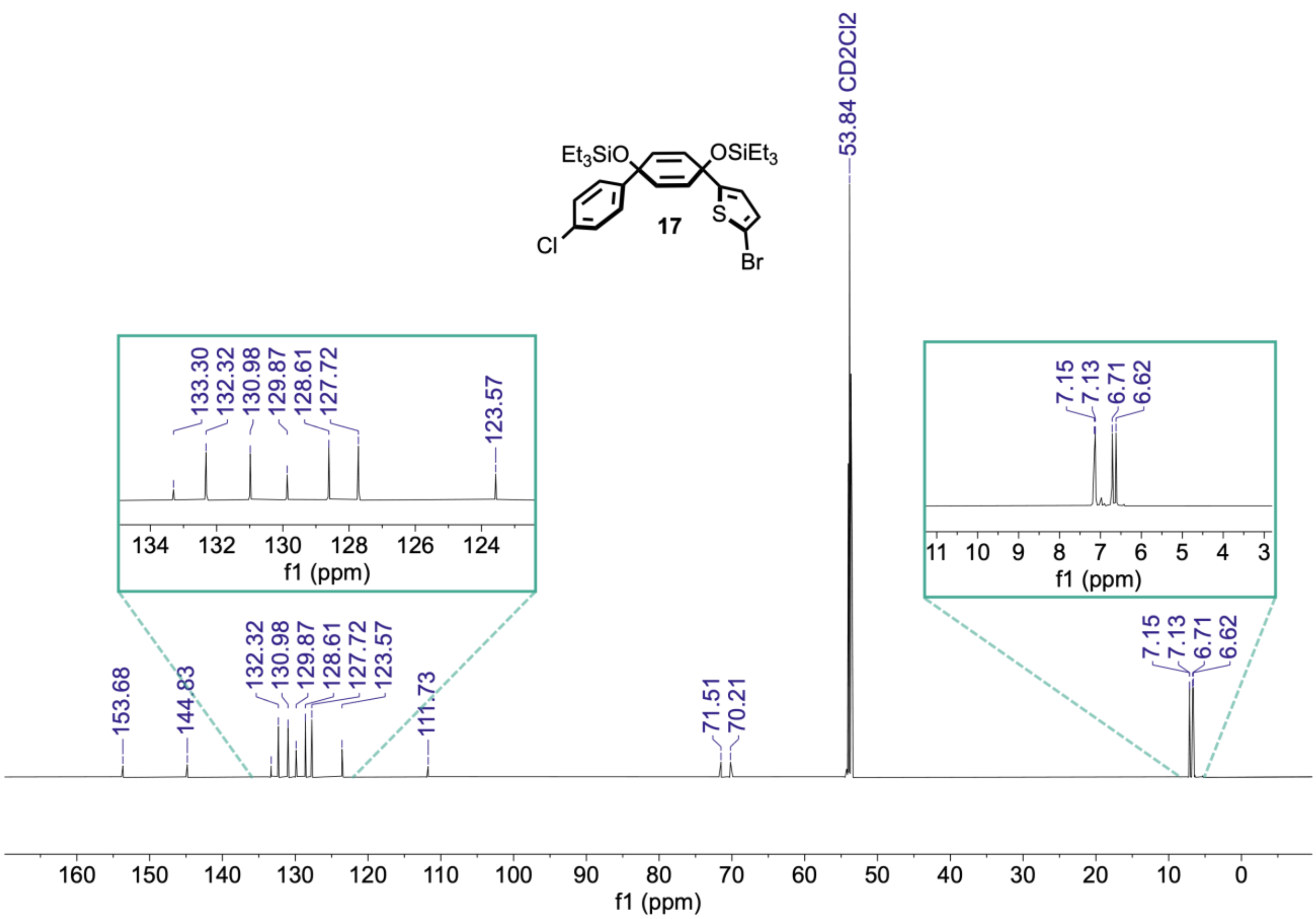


**Figure S4.** $^{13}C$ NMR spectrum (151 MHz) of **17** in Methylene Chloride-$d_2$ at room temperature.

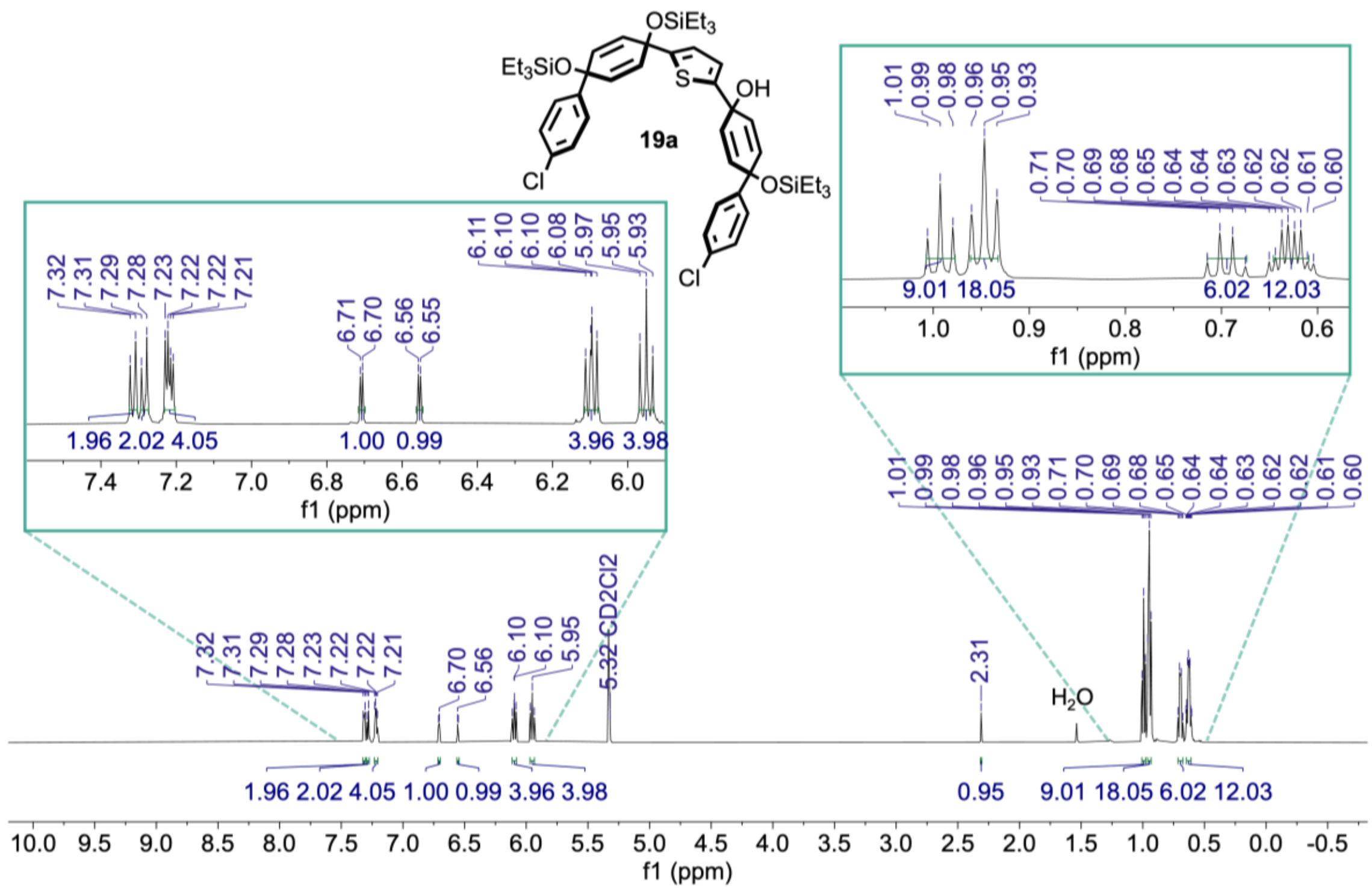

**Figure S5.** $^{1}H$ NMR spectrum (600 MHz) of **19a** in Methylene Chloride-$d_2$ at room temperature.

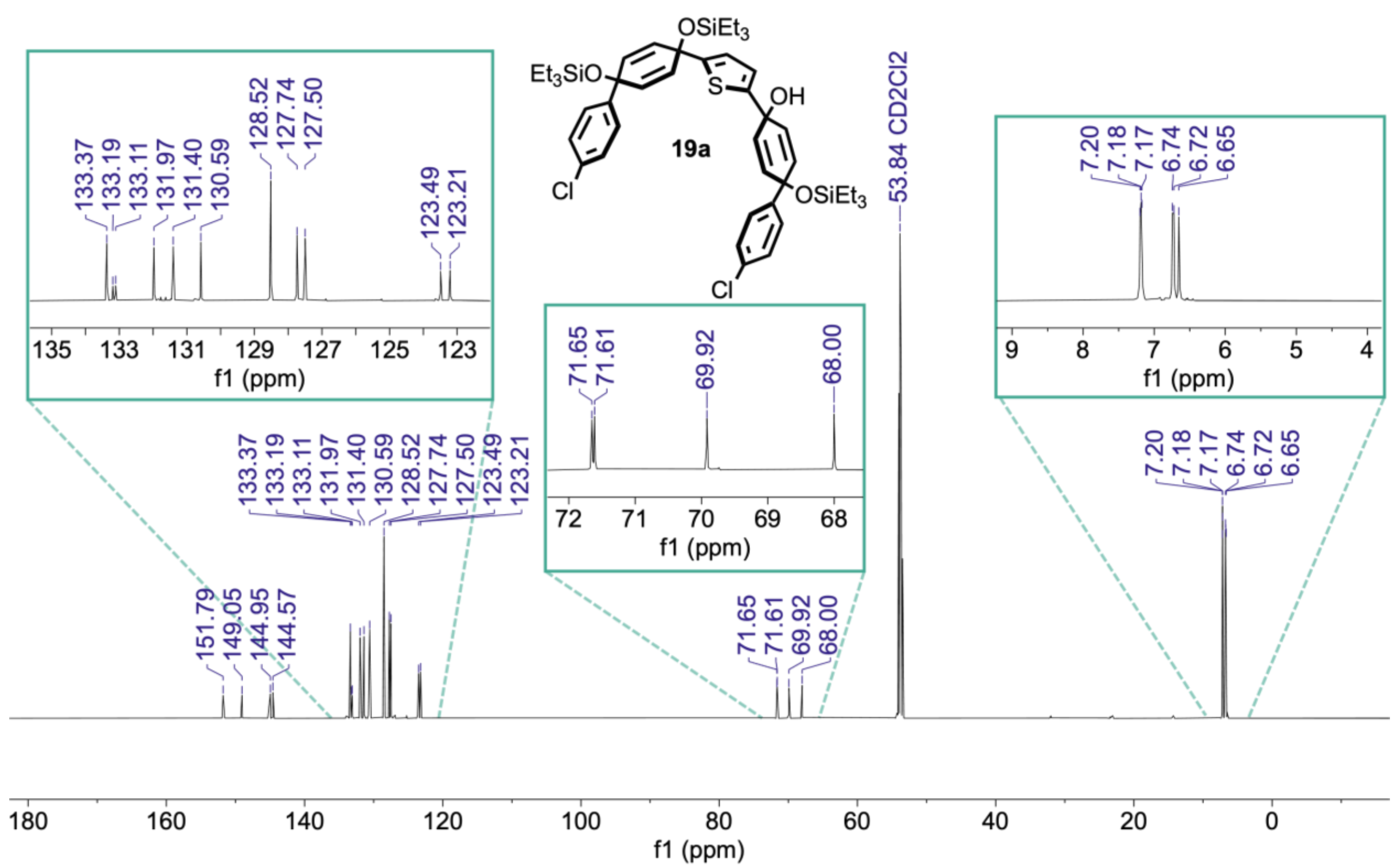

**Figure S6.** $^{13}C$ NMR spectrum (151 MHz) of **19a** in Methylene Chloride-$d_2$ at room temperature.

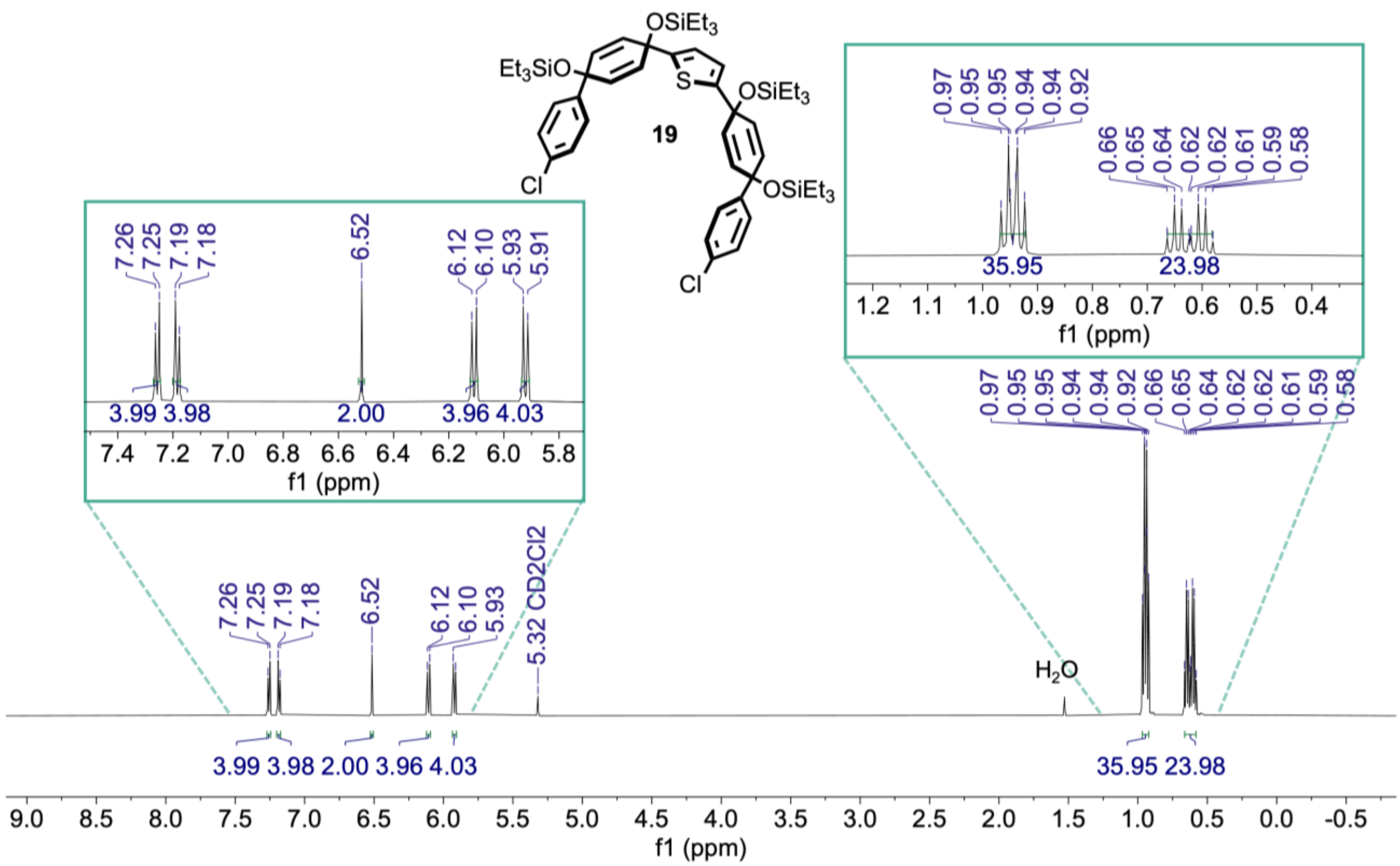


**Figure S7.** $^{1}$H NMR spectrum (600 MHz) of **19** in Methylene Chloride-$d_2$ at room temperature.

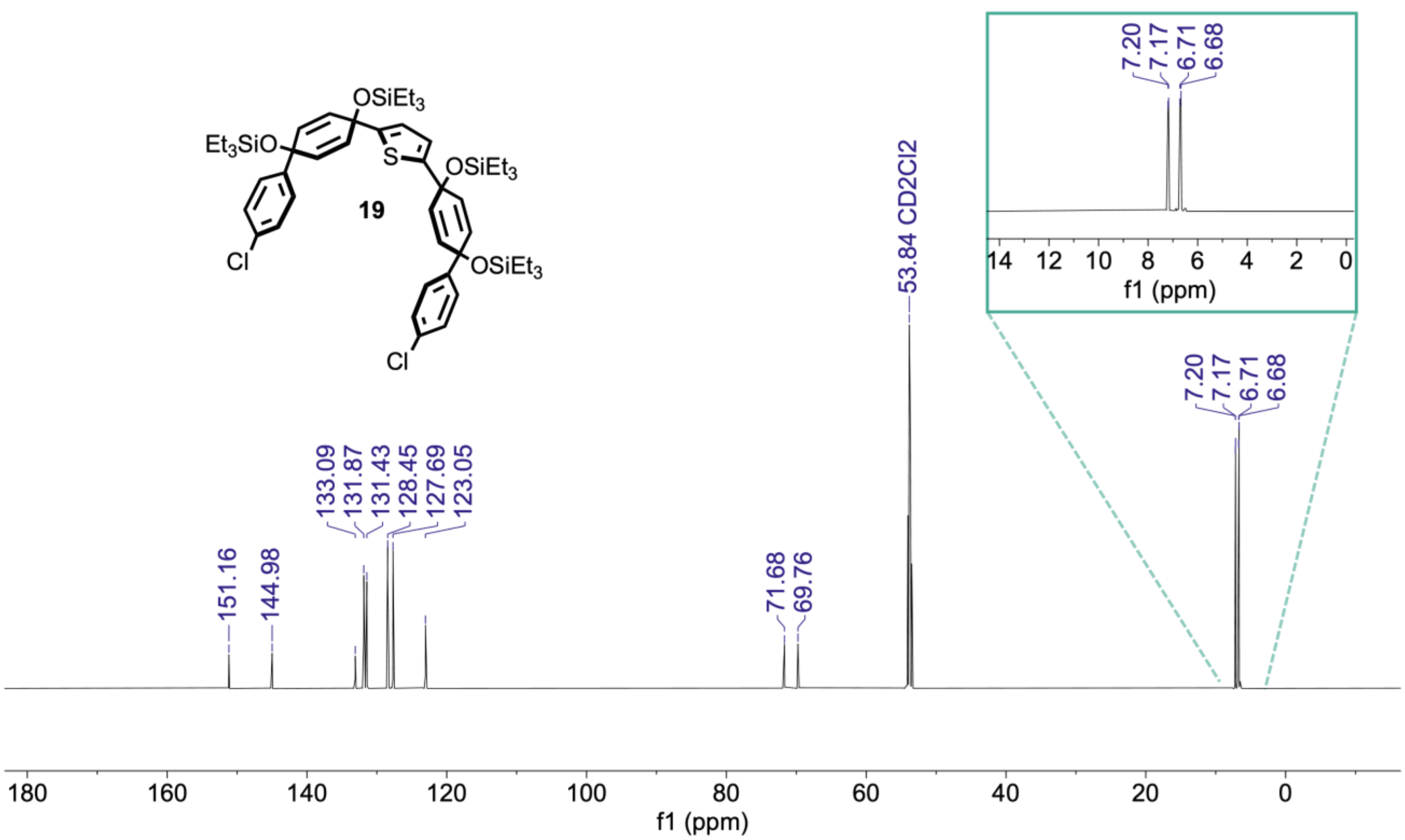


**Figure S8.** $^{13}$C NMR spectrum (151 MHz) of **19** in Methylene Chloride-$d_2$ at room temperature.

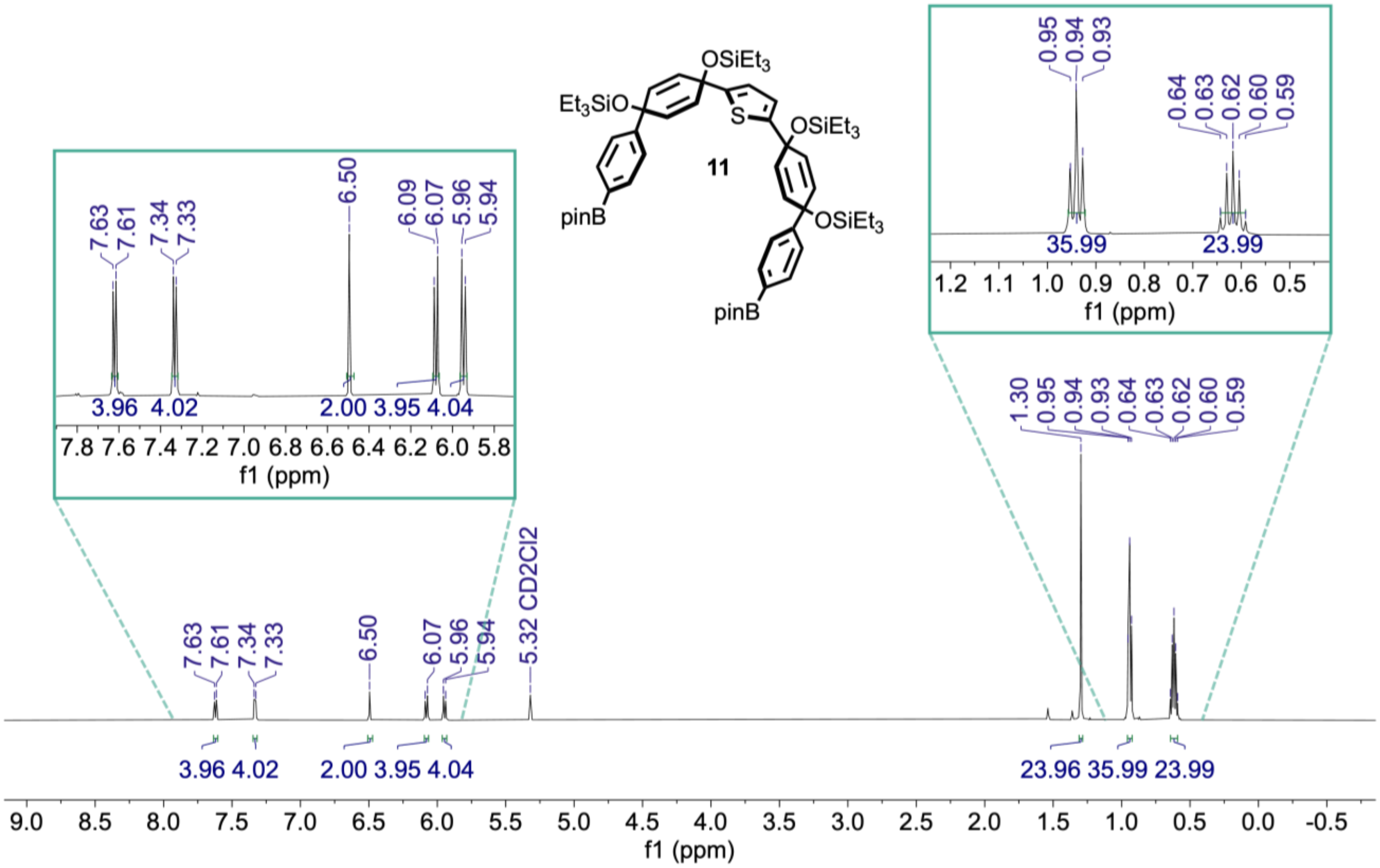


**Figure S9.** $^{1}$H NMR spectrum (600 MHz) of **11** in Methylene Chloride-$d_2$ at room temperature.

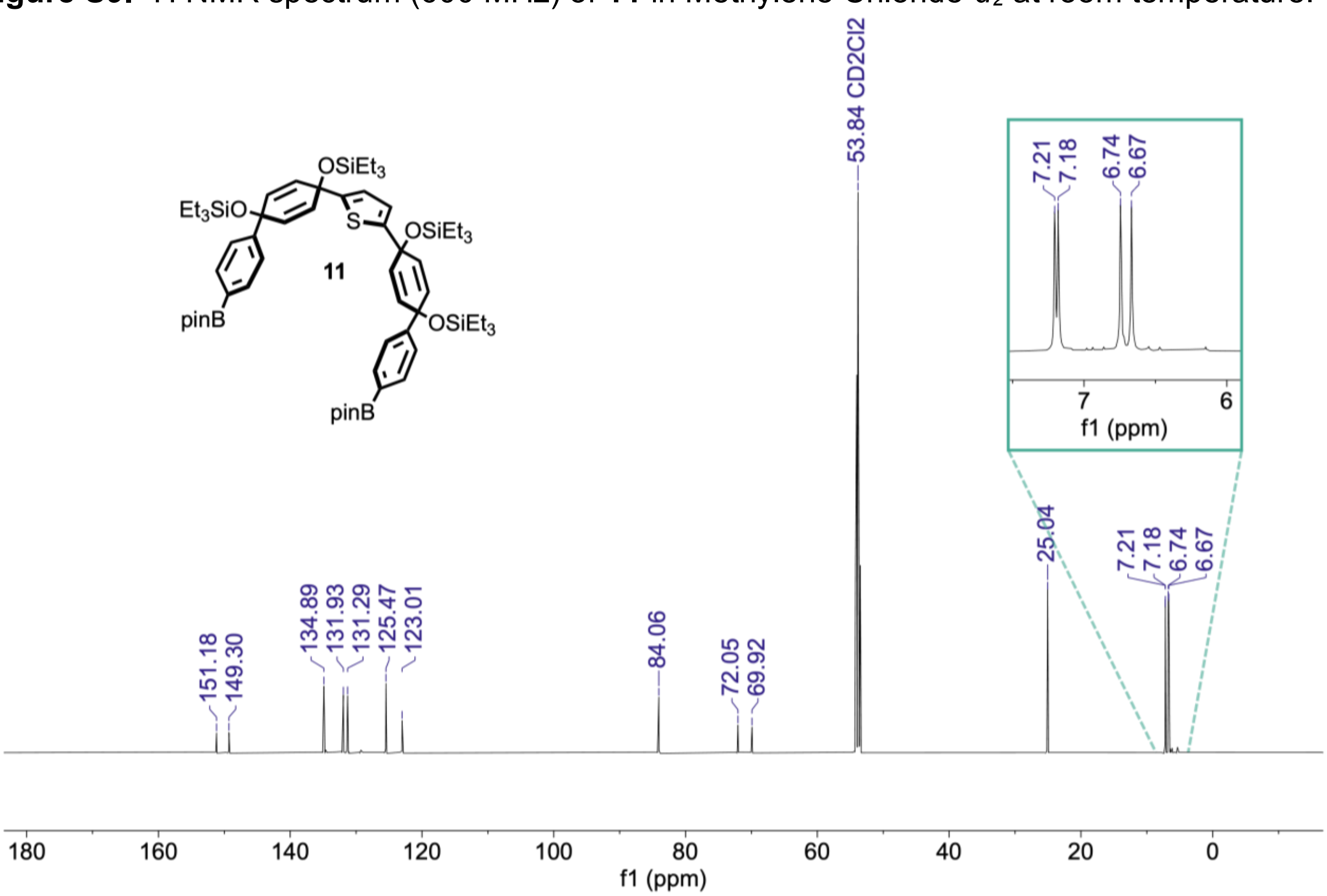


**Figure S10.** $^{13}$C NMR spectrum (151 MHz) of **11** in Methylene Chloride-$d_2$ at room temperature.

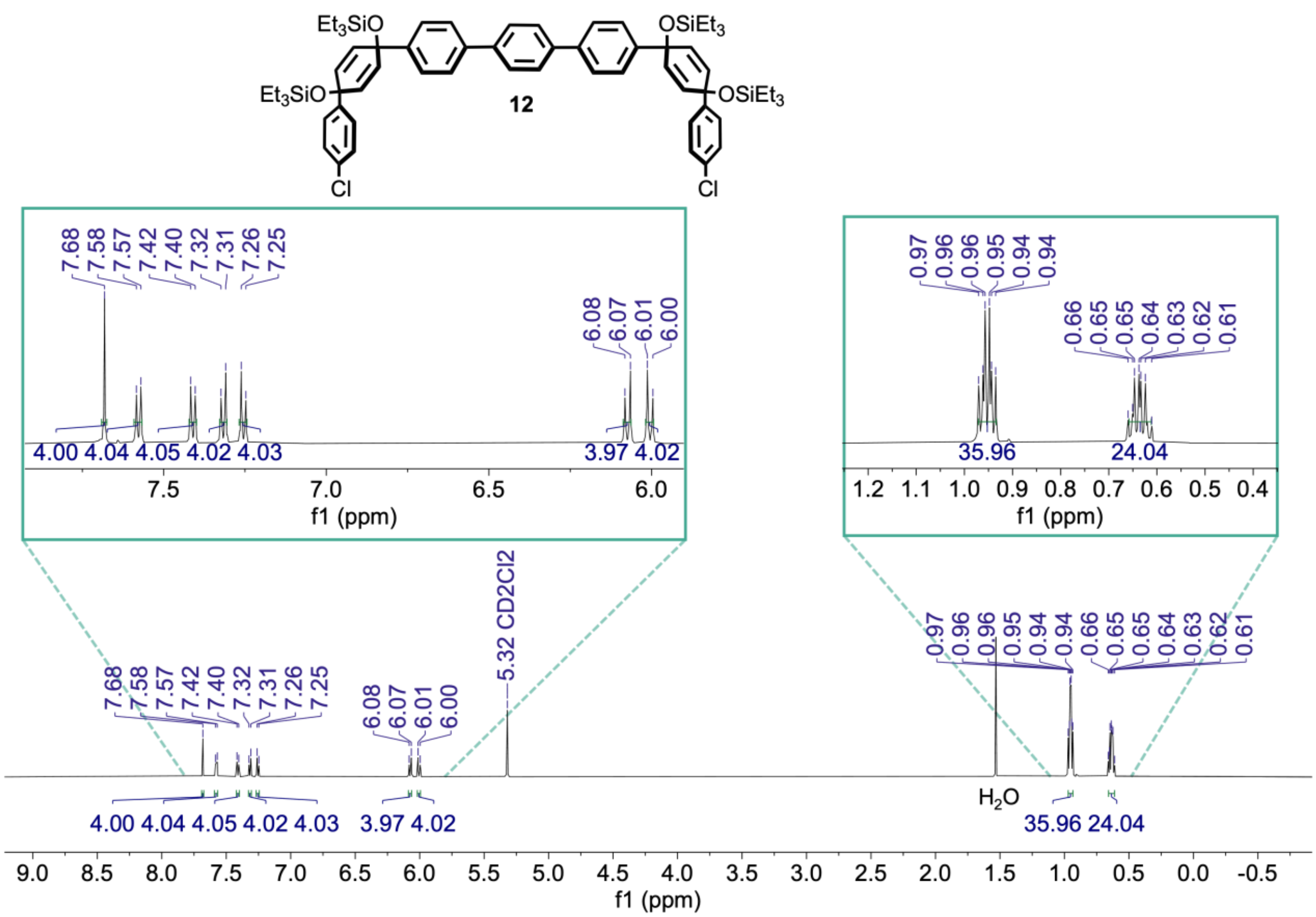

**Figure S11.** $^{1}$H NMR spectrum (600 MHz) of **12** in Methylene Chloride-$d_2$ at room temperature.

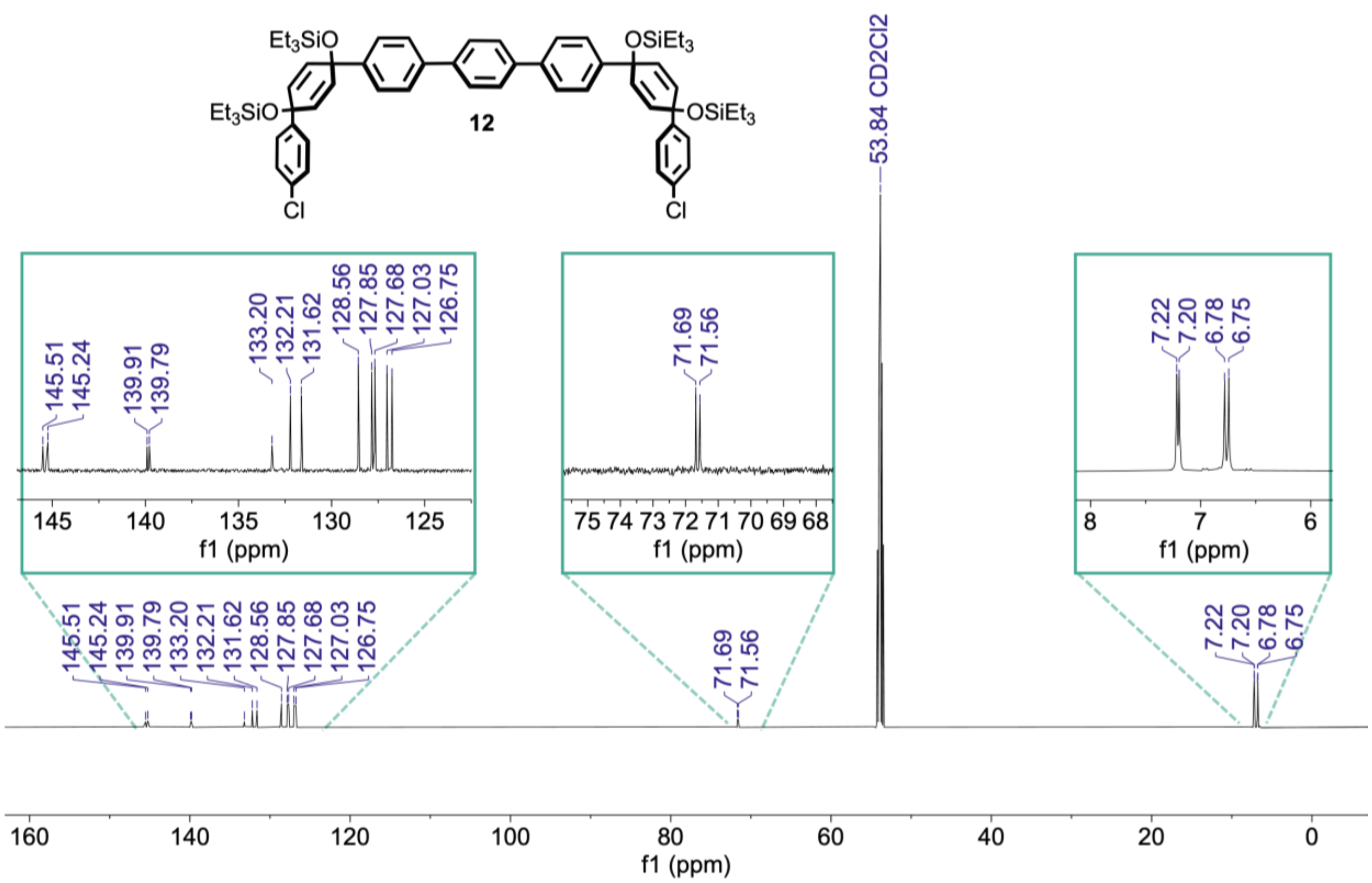

**Figure S12.** $^{13}$C NMR spectrum (151 MHz) of **12** in Methylene Chloride-$d_2$ at room temperature.

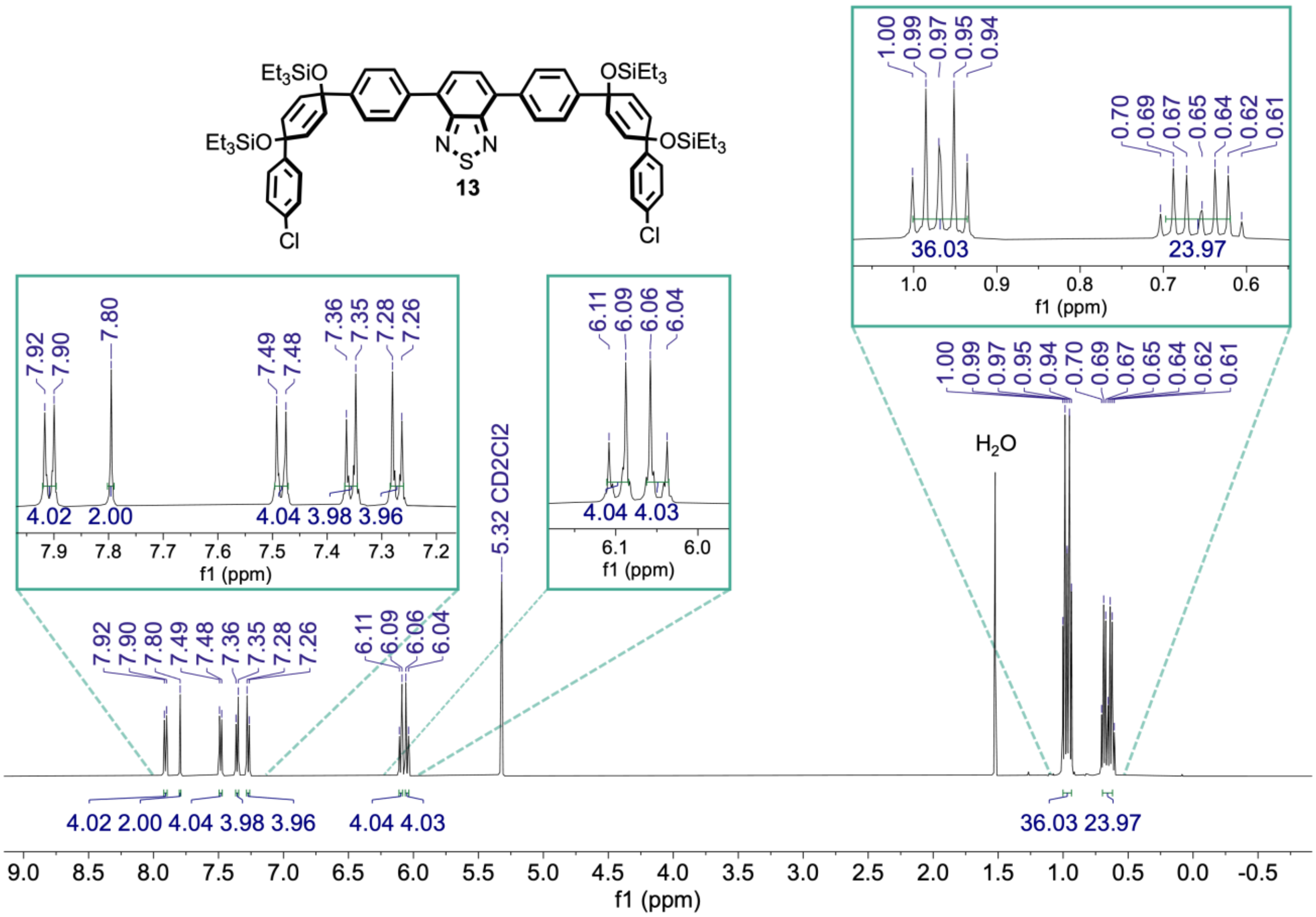


**Figure S13.** $^{1}$H NMR spectrum (600 MHz) of **12** in Methylene Chloride-$d_2$ at room temperature.

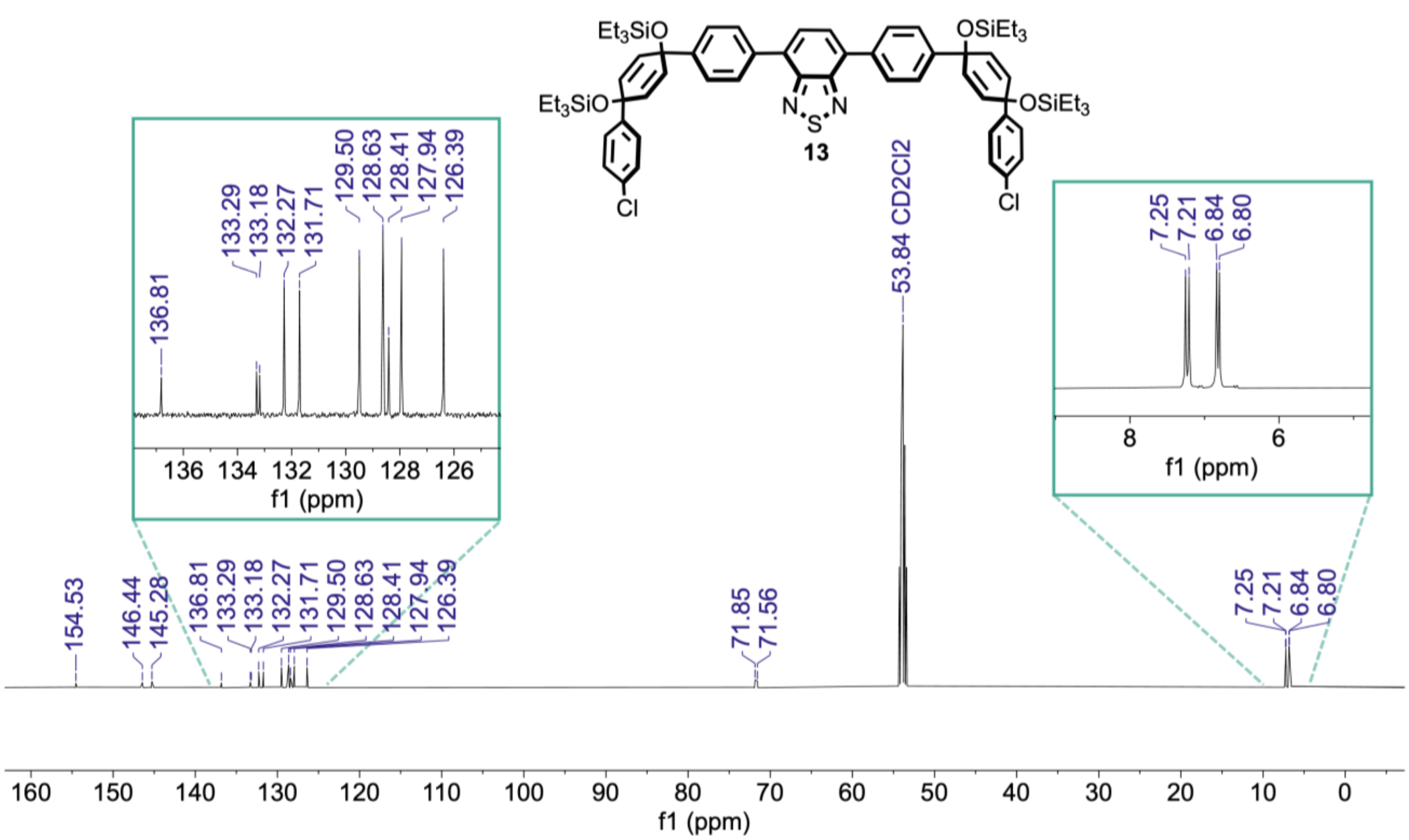


**Figure S14.** $^{13}$C NMR spectrum (151 MHz) of **12** in Methylene Chloride-$d_2$ at room temperature.

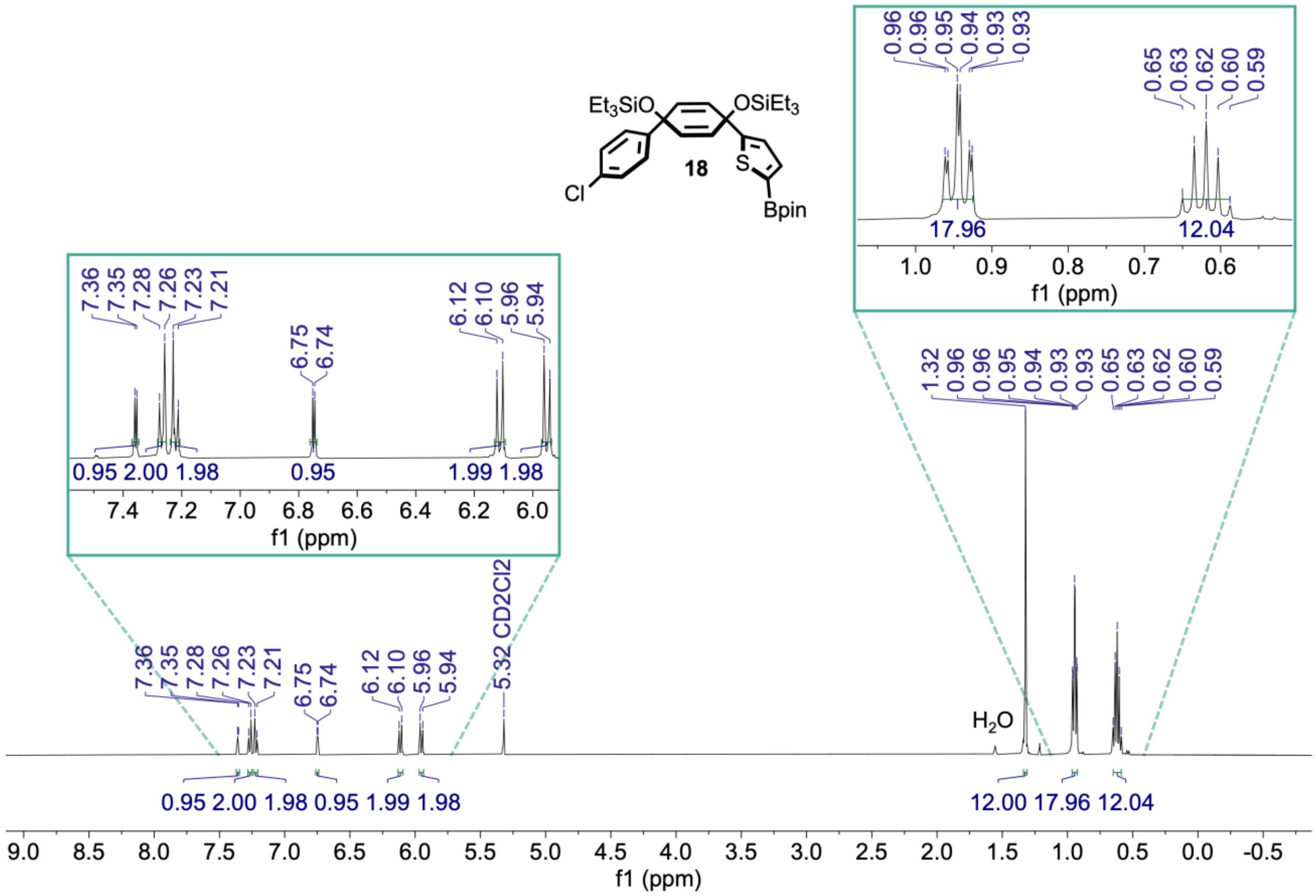

**Figure S15.** $^{1}H$ NMR spectrum (600 MHz) of **18** in Methylene Chloride-$d_2$ at room temperature.

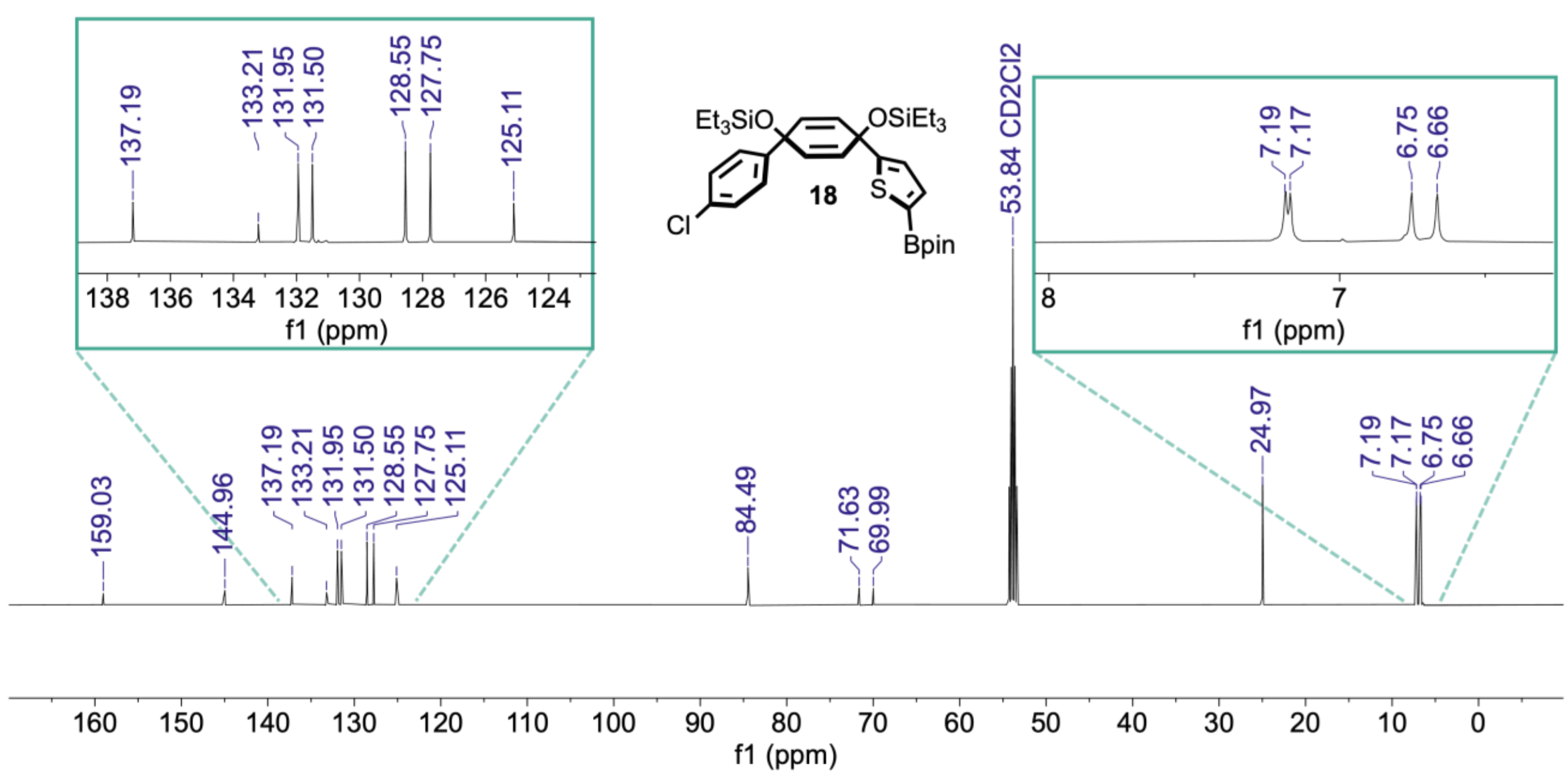

**Figure S16.** $^{13}C$ NMR spectrum (151 MHz) of **18** in Methylene Chloride-$d_2$ at room temperature.

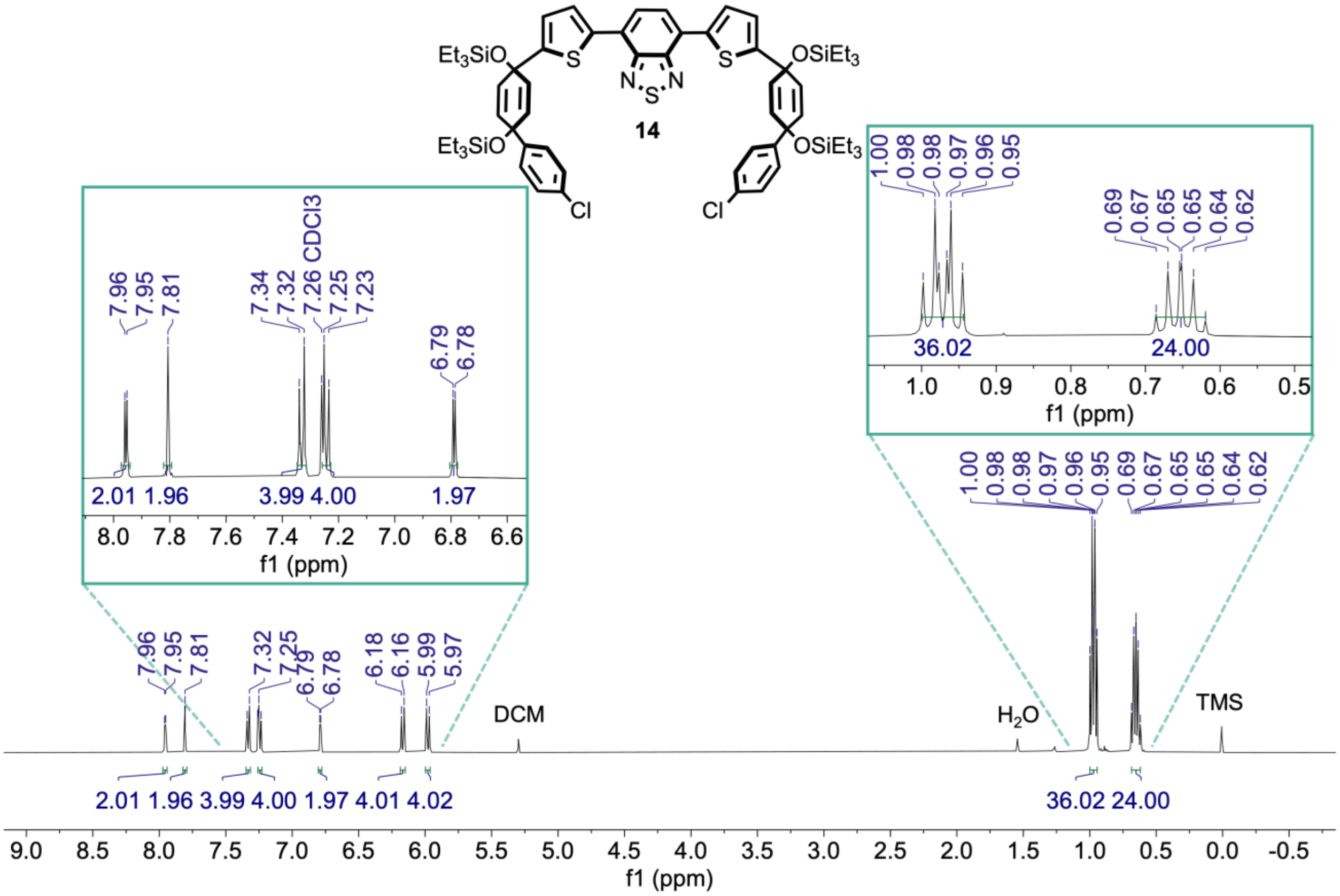

**Figure S17.** $^{1}$H NMR spectrum (500 MHz) of **14** in Chloroform-*d* at room temperature.

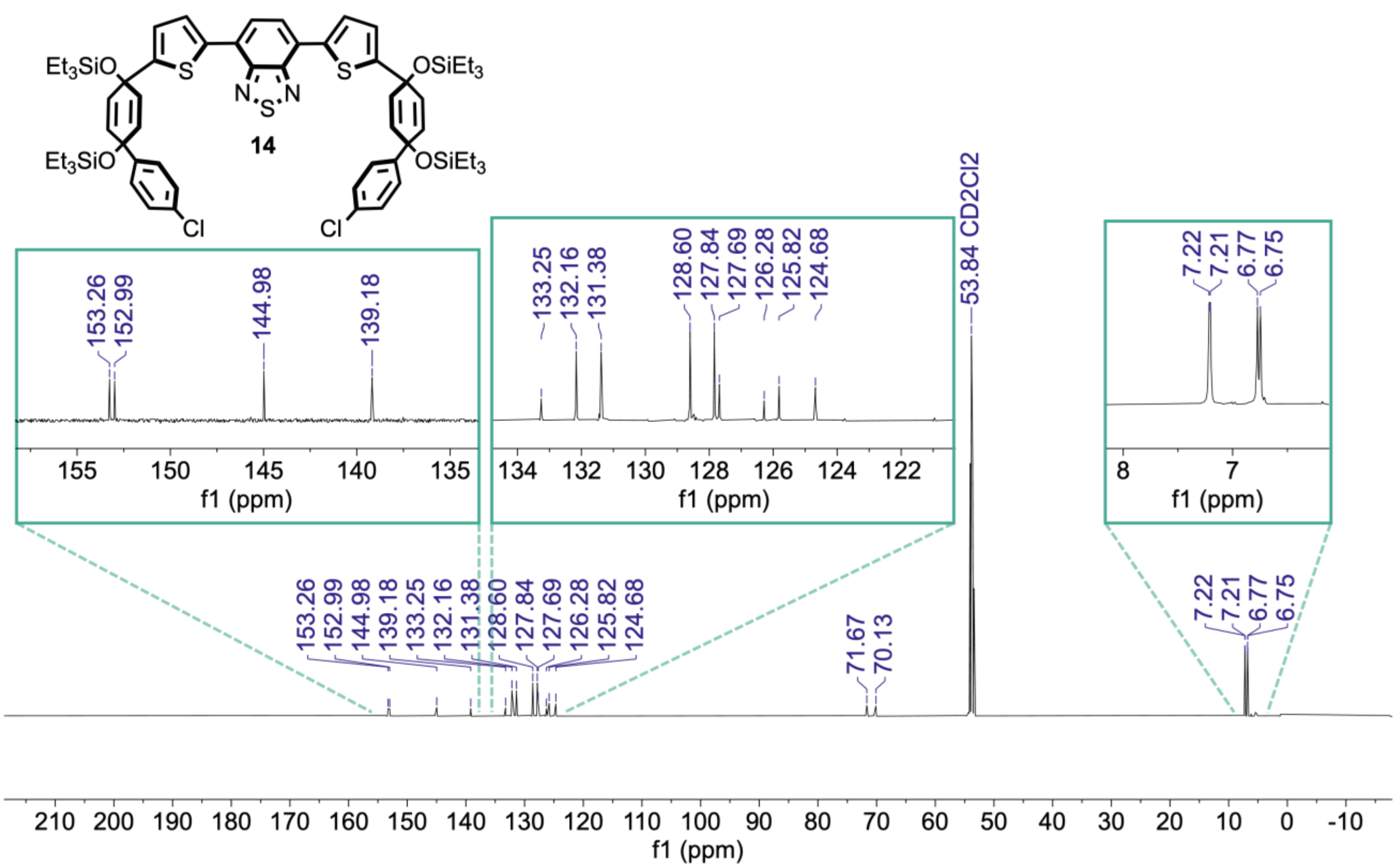

**Figure S18.** $^{13}$C NMR spectrum (126 MHz) of **14** in Methylene Chloride-$d_2$ at room temperature.

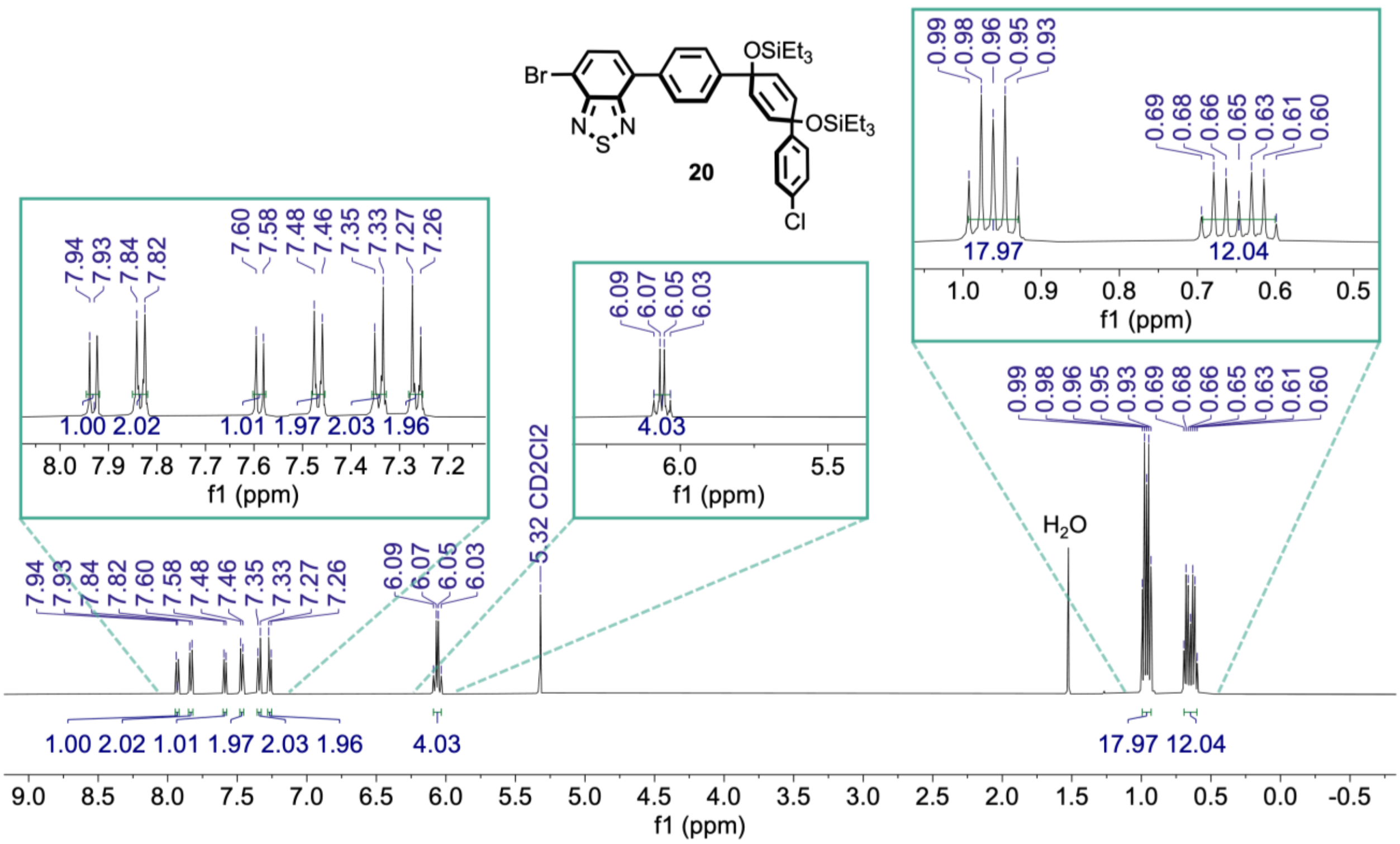

**Figure S19.** $^1$H NMR spectrum (600 MHz) of **20** in Methylene Chloride-$d_2$ at room temperature.

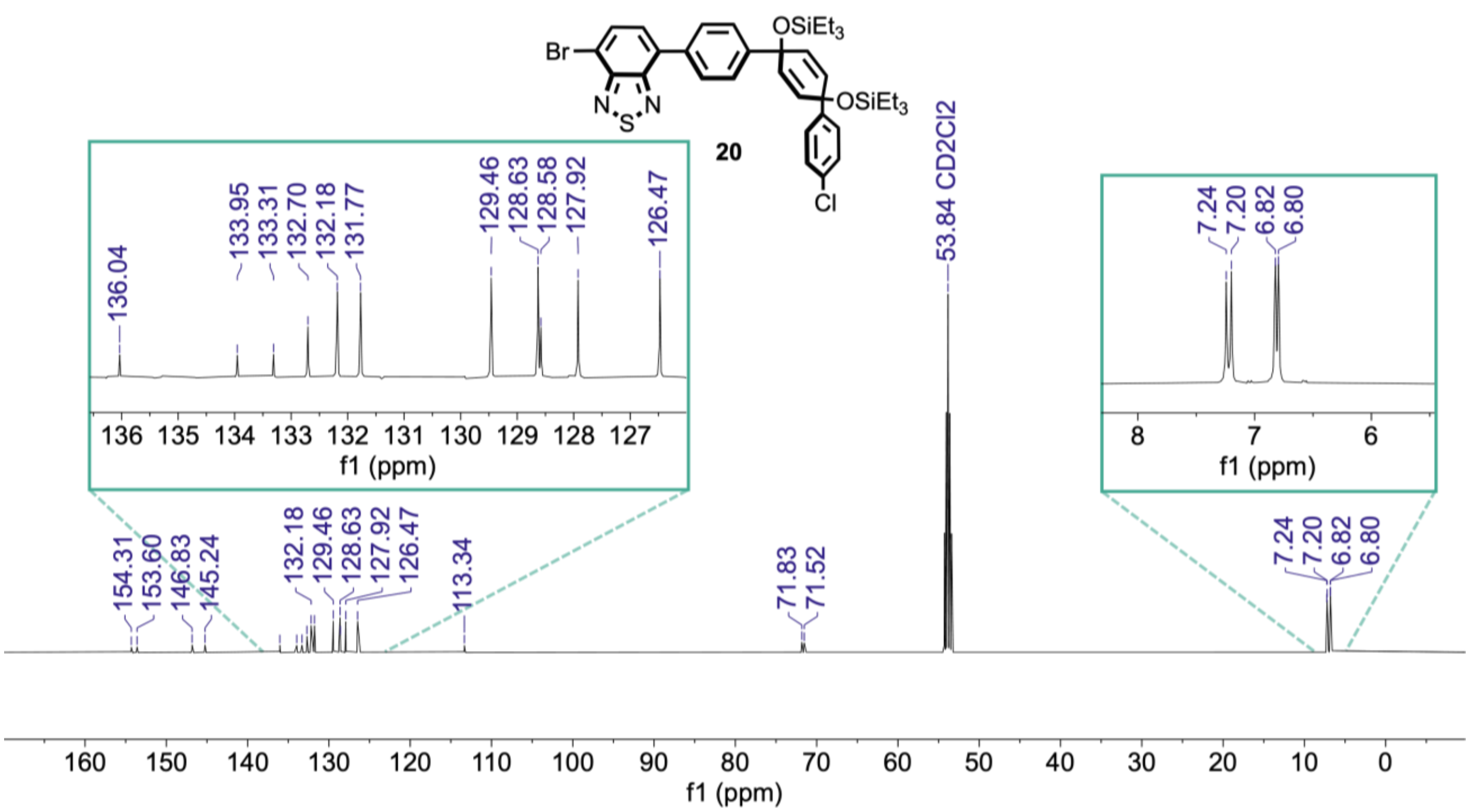

**Figure S20.** $^{13}$C NMR spectrum (151 MHz) of **20** in Methylene Chloride-$d_2$ at room temperature.

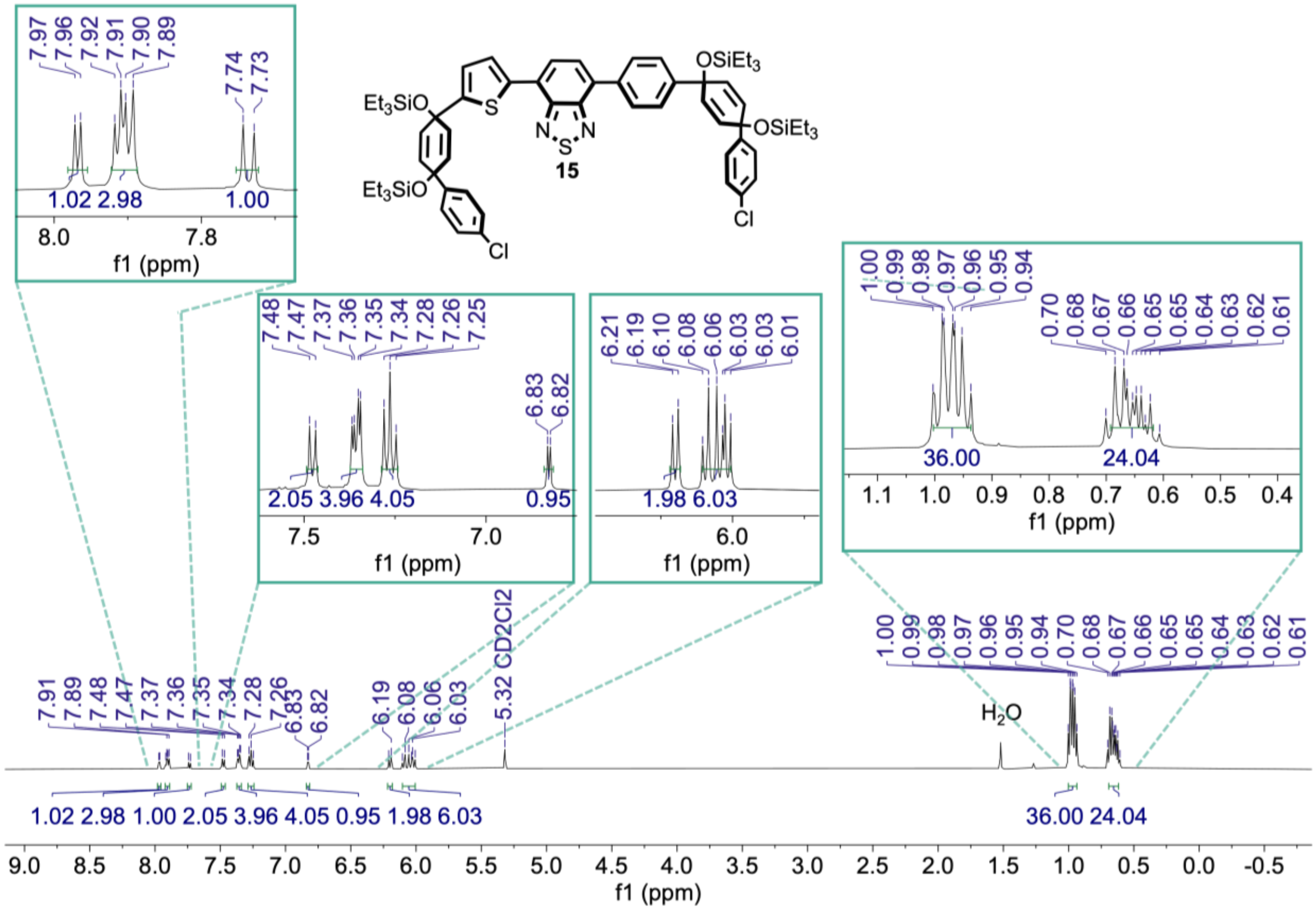


**Figure S21.** $^{1}H$ NMR spectrum (600 MHz) of **15** in Methylene Chloride-$d_2$ at room temperature.

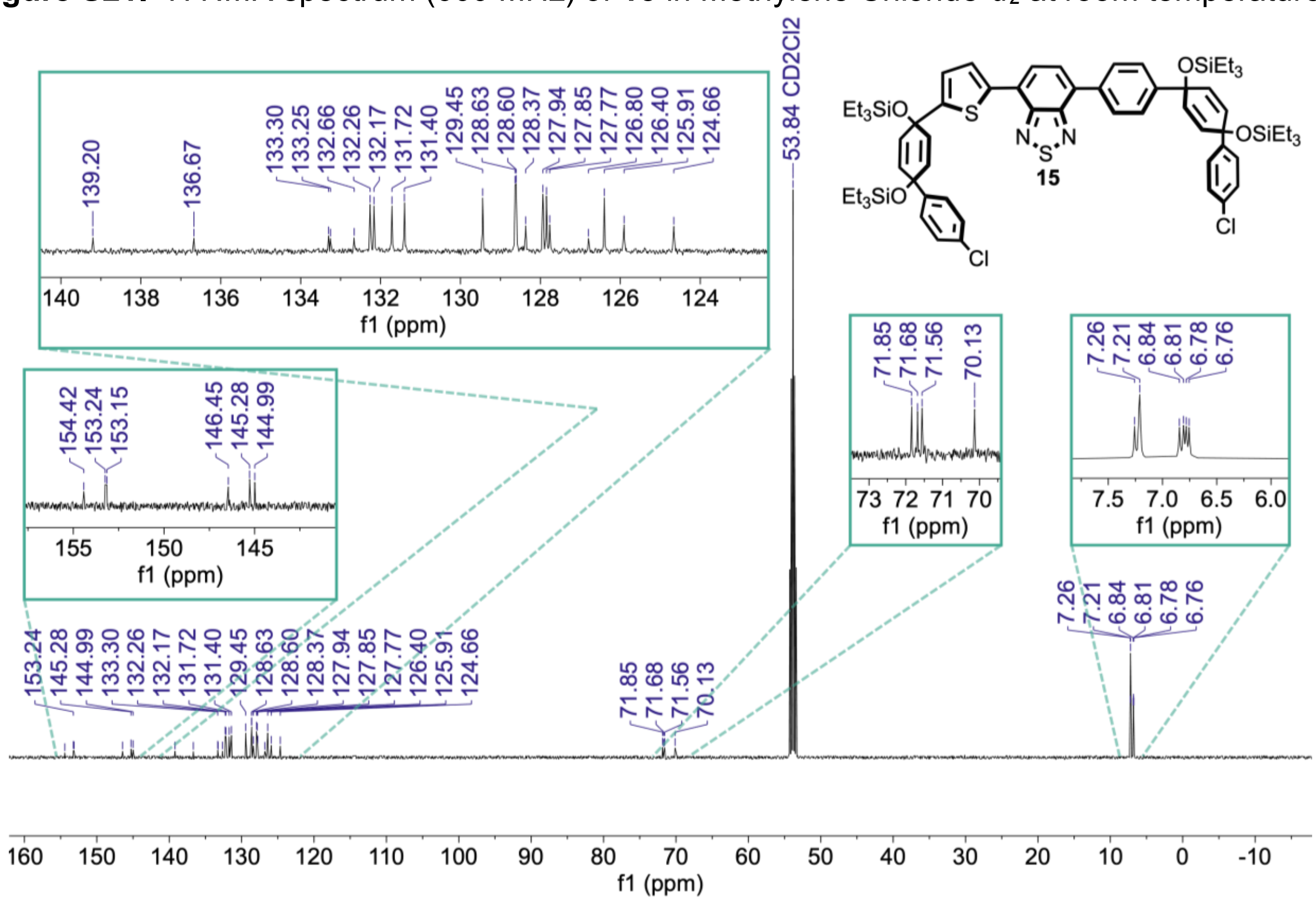


**Figure S22.** $^{13}C$ NMR spectrum (151 MHz) of **15** in Methylene Chloride-$d_2$ at room temperature.

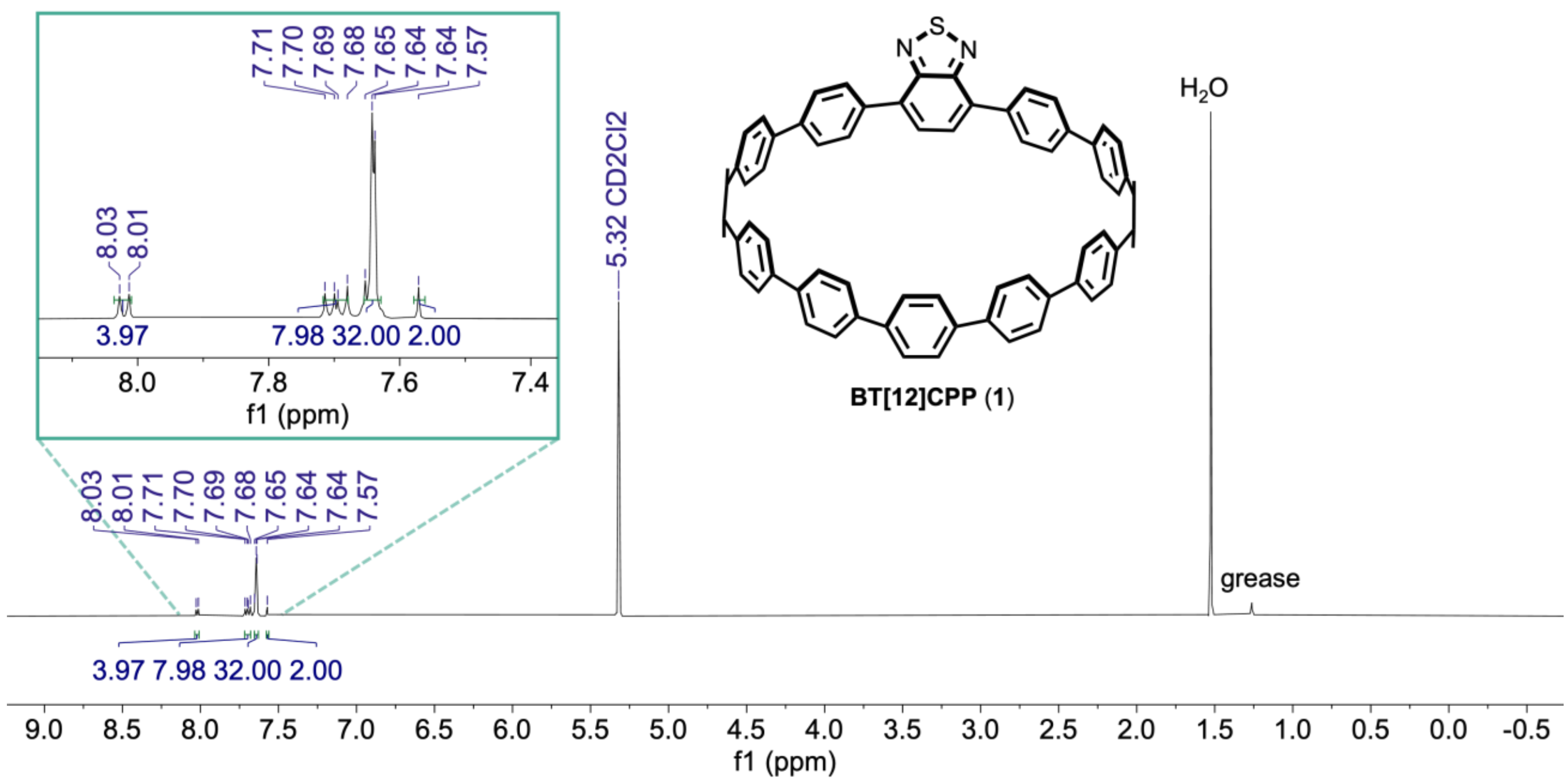


**Figure S23.** $^{1}$H NMR spectrum (600 MHz) of **1** in Methylene Chloride-$d_2$ at room temperature.

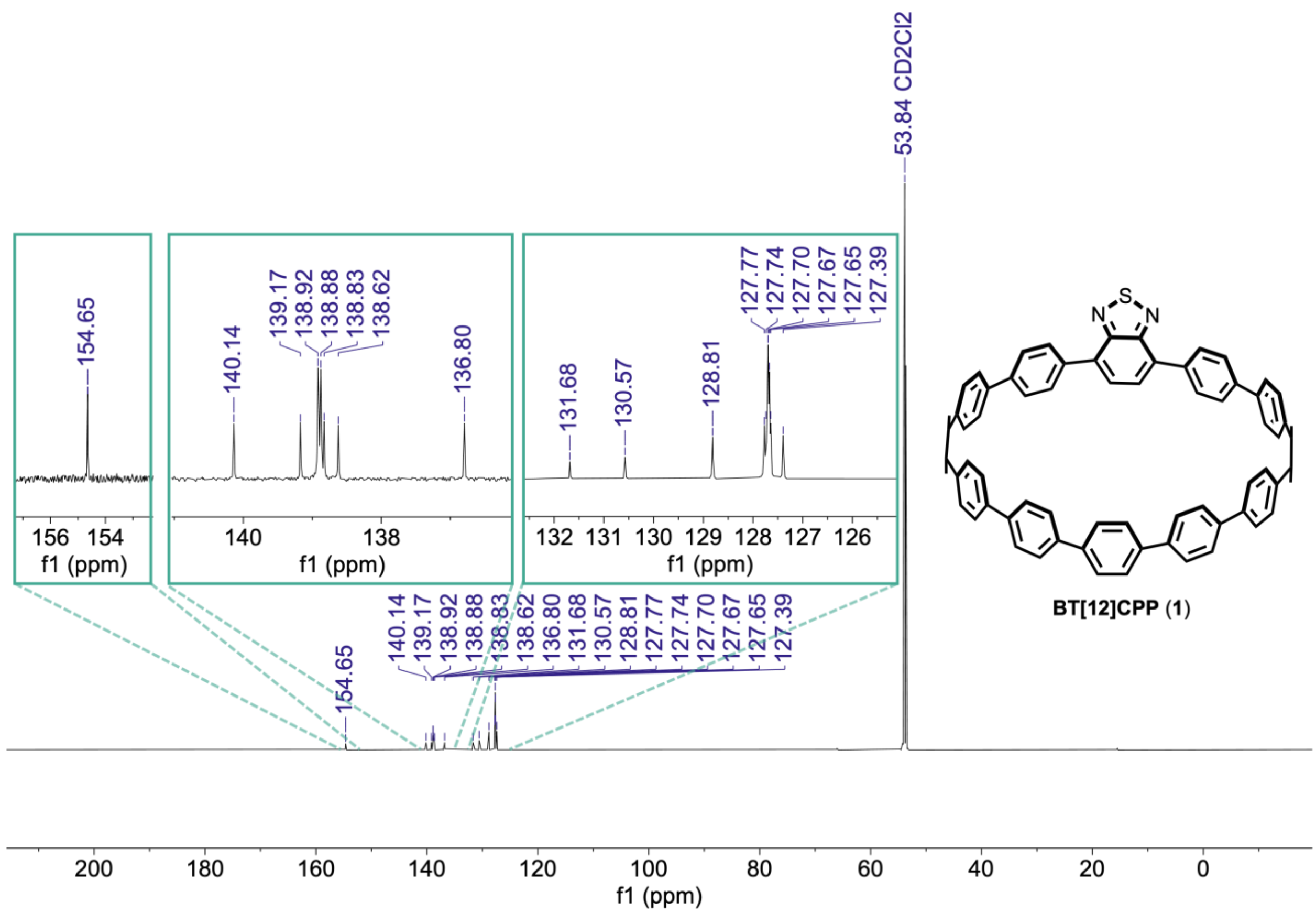


**Figure S24.** $^{13}$C NMR spectrum (151 MHz) of **1** in Methylene Chloride-$d_2$ at room temperature.

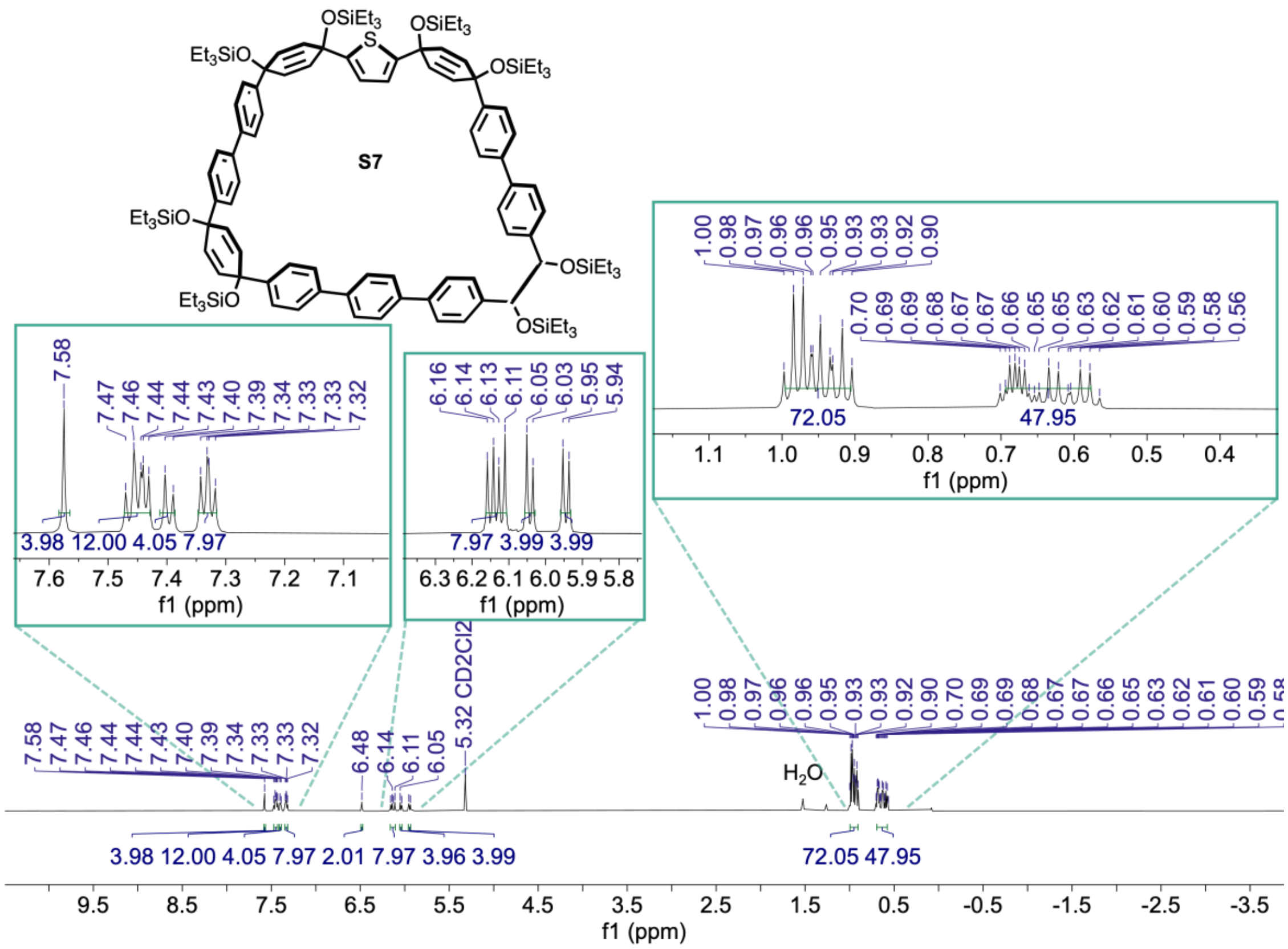

**Figure S25.** $^{1}$H NMR spectrum (600 MHz) of **S7** in Methylene Chloride-$d_2$ at room temperature.

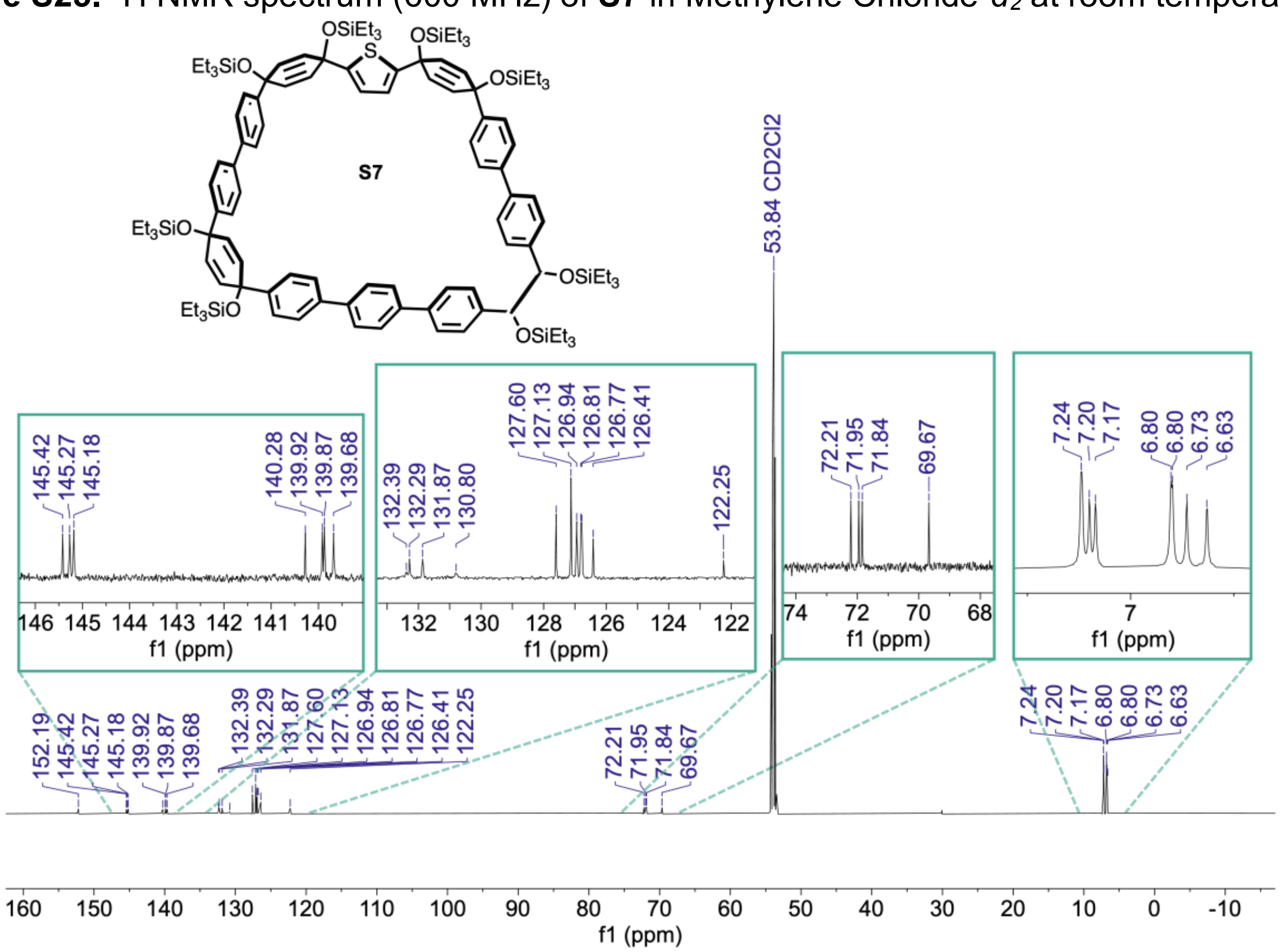

**Figure S26.** $^{13}$C NMR spectrum (151 MHz) of **S7** in Methylene Chloride-$d_2$ at room temperature.

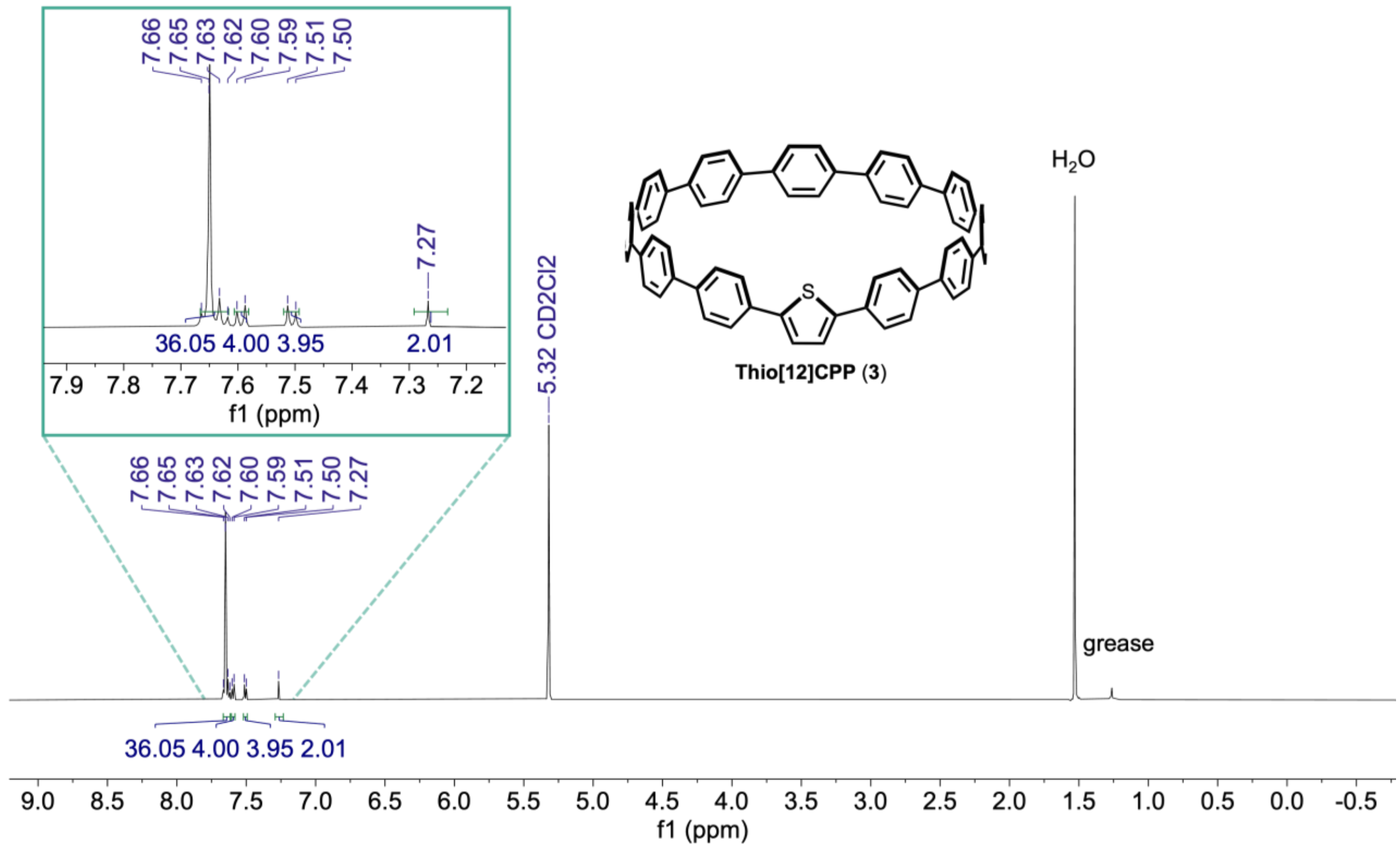


**Figure S27.** $^{1}$H NMR spectrum (600 MHz) of **3** in Methylene Chloride-$d_2$ at room temperature.

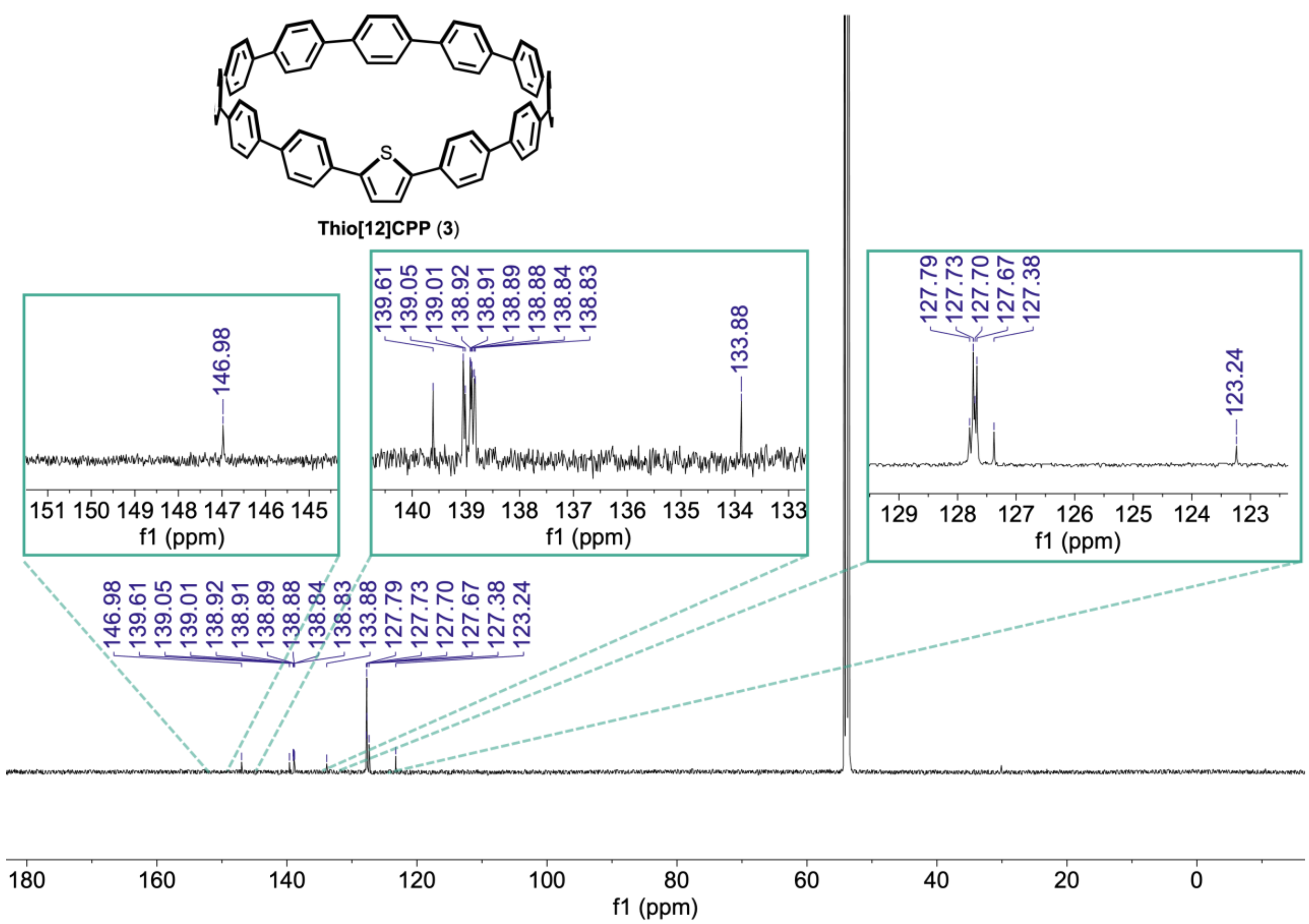


**Figure S28.** $^{13}$C NMR spectrum (151 MHz) of **3** in Methylene Chloride-$d_2$ at room temperature.

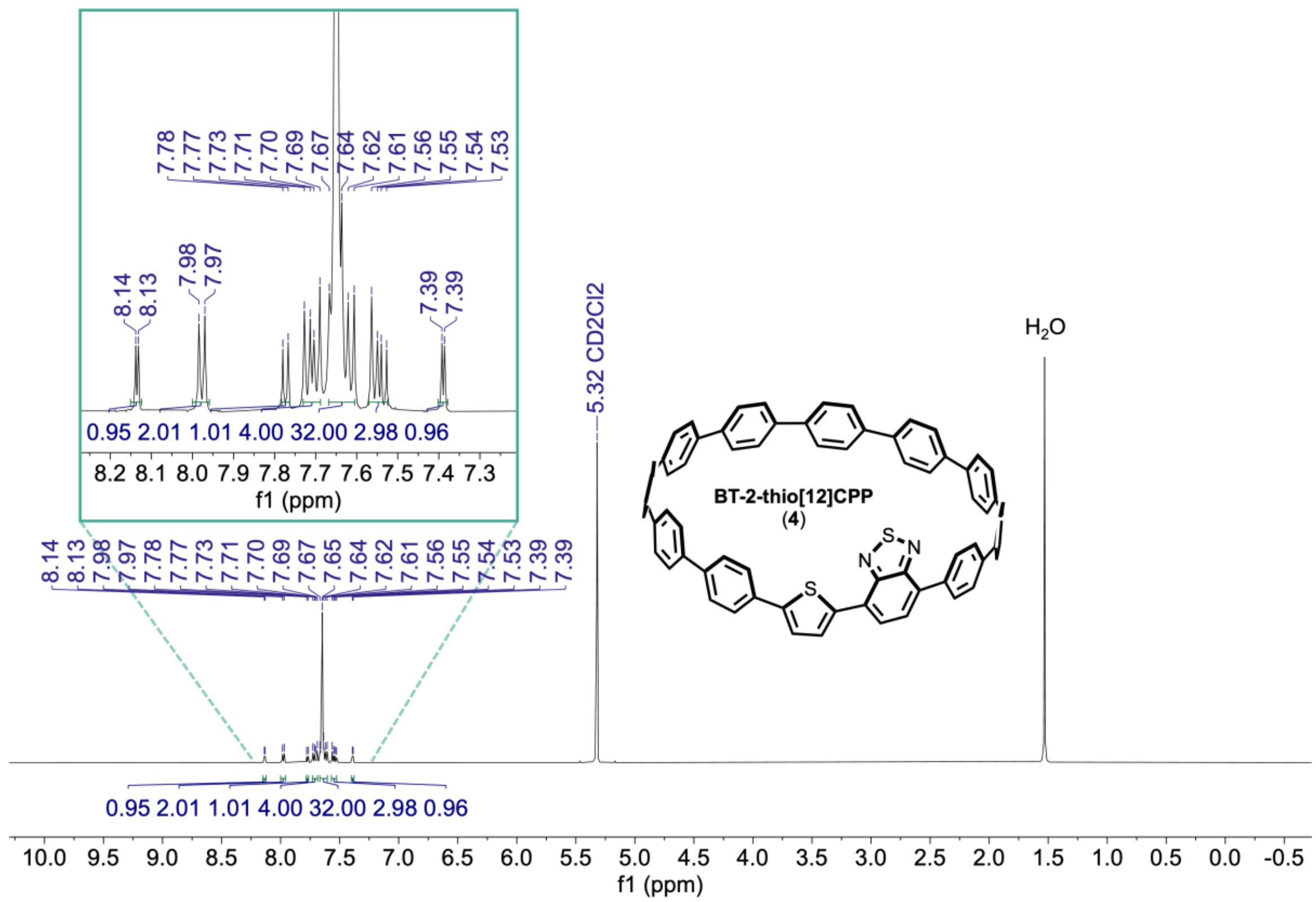

**Figure S29.** $^{1}$H NMR spectrum (600 MHz) of **4** in Methylene Chloride-$d_2$ at room temperature.

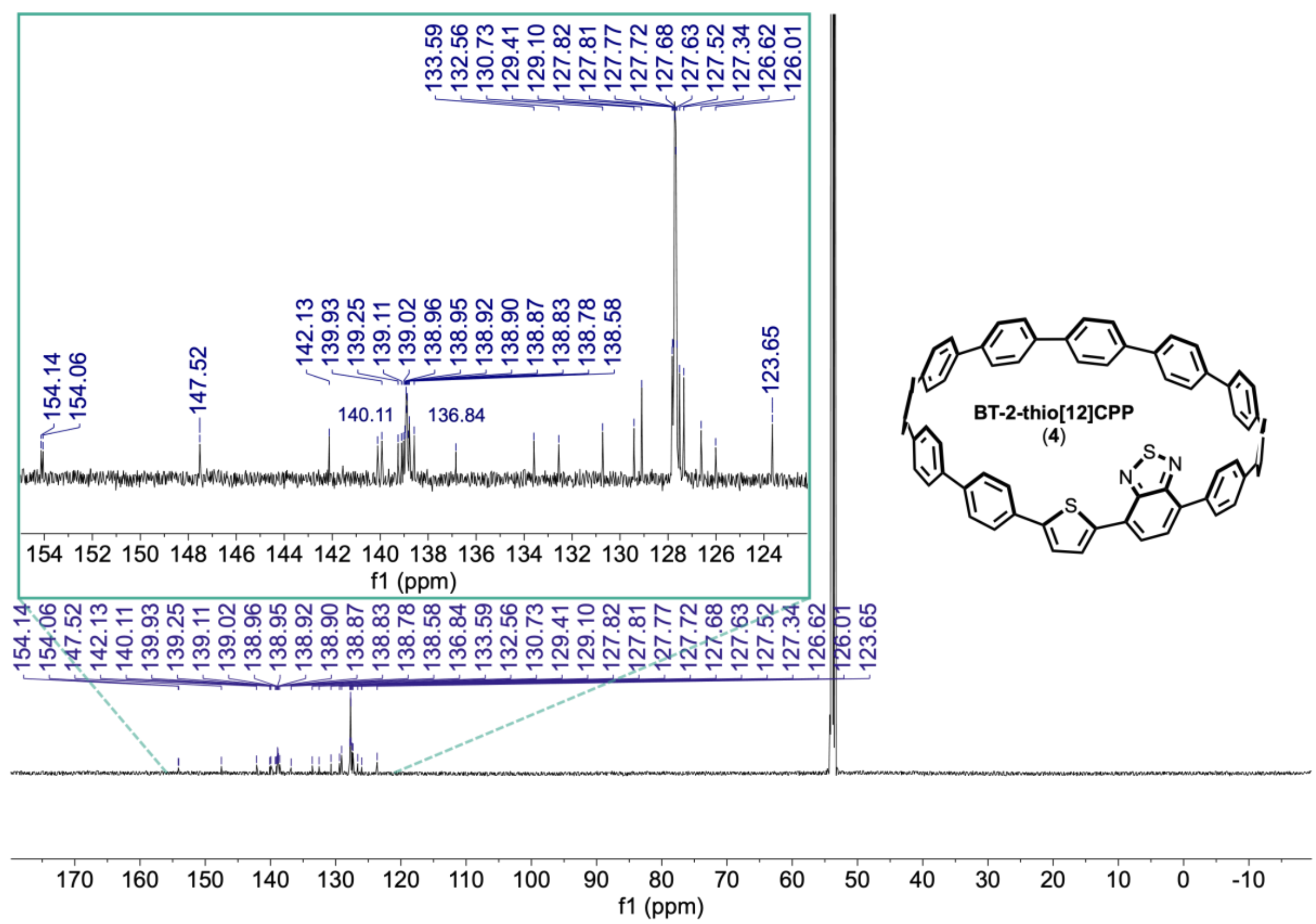

**Figure S30.** $^{13}$C NMR spectrum (151 MHz) of **4** in Methylene Chloride-$d_2$ at room temperature.

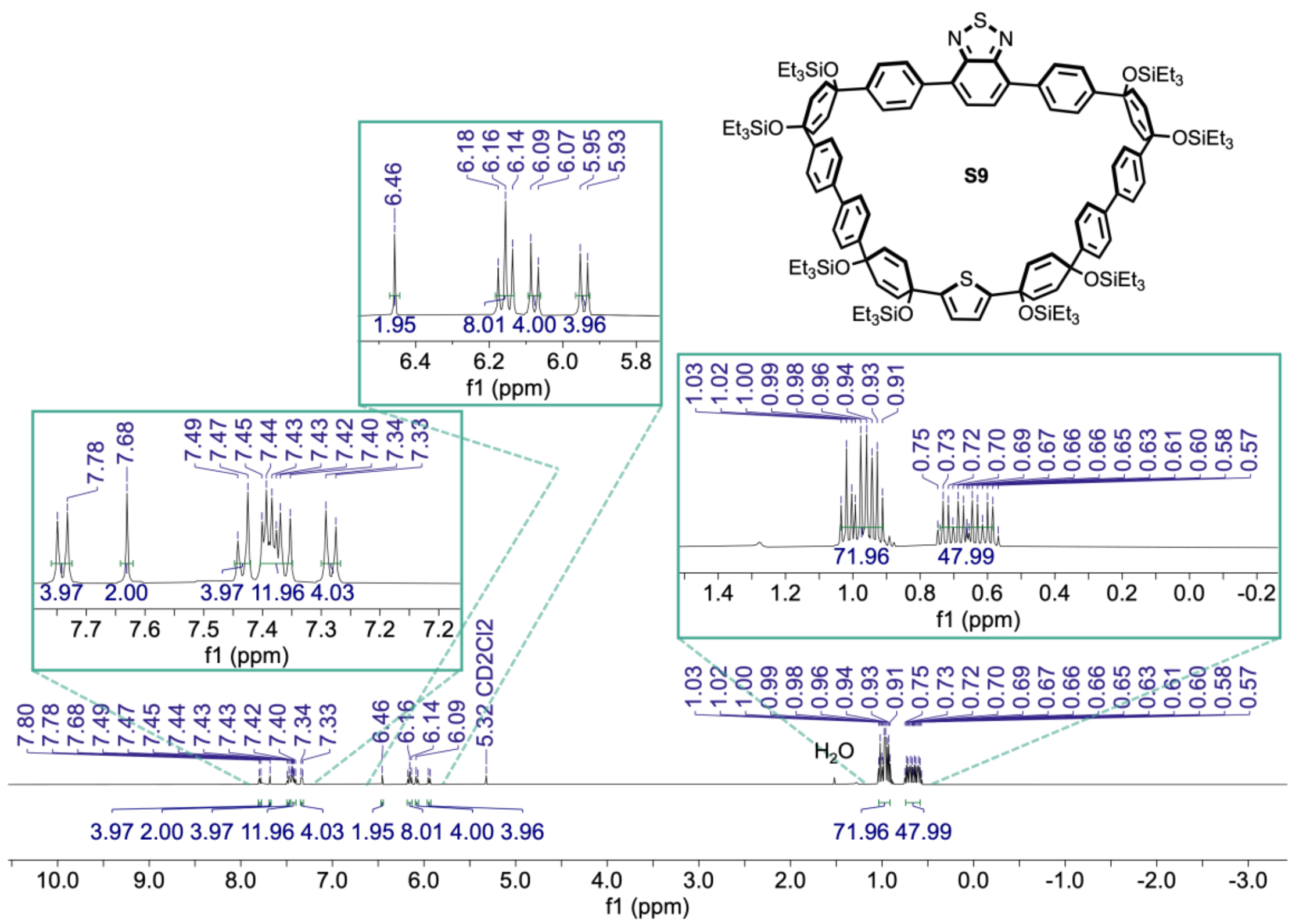


**Figure S31.** $^{1}$H NMR spectrum (600 MHz) of **S9** in Methylene Chloride-$d_2$ at room temperature.

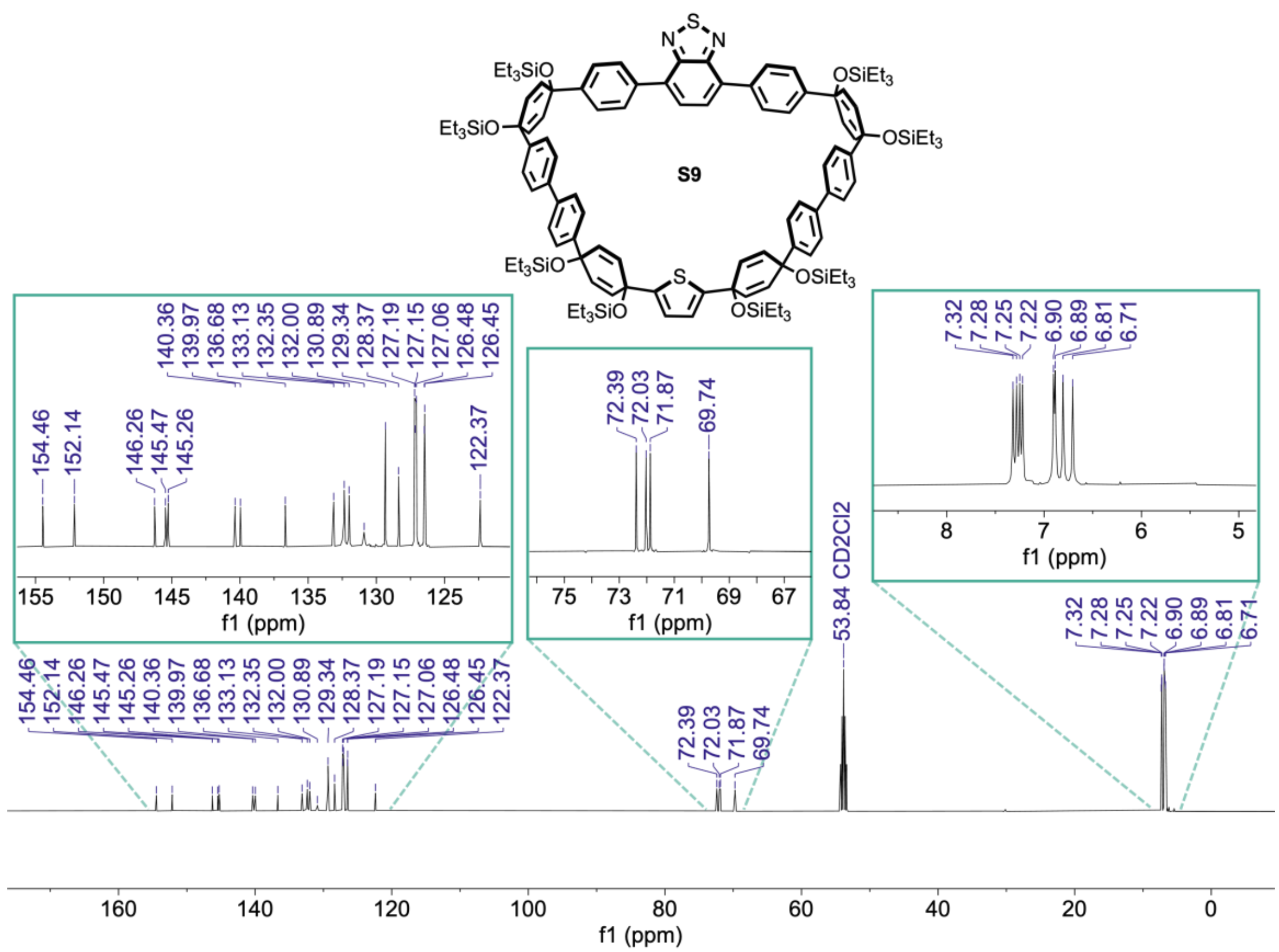


**Figure S32.** $^{13}$C NMR spectrum (151 MHz) of **S9** in Methylene Chloride-$d_2$ at room temperature.

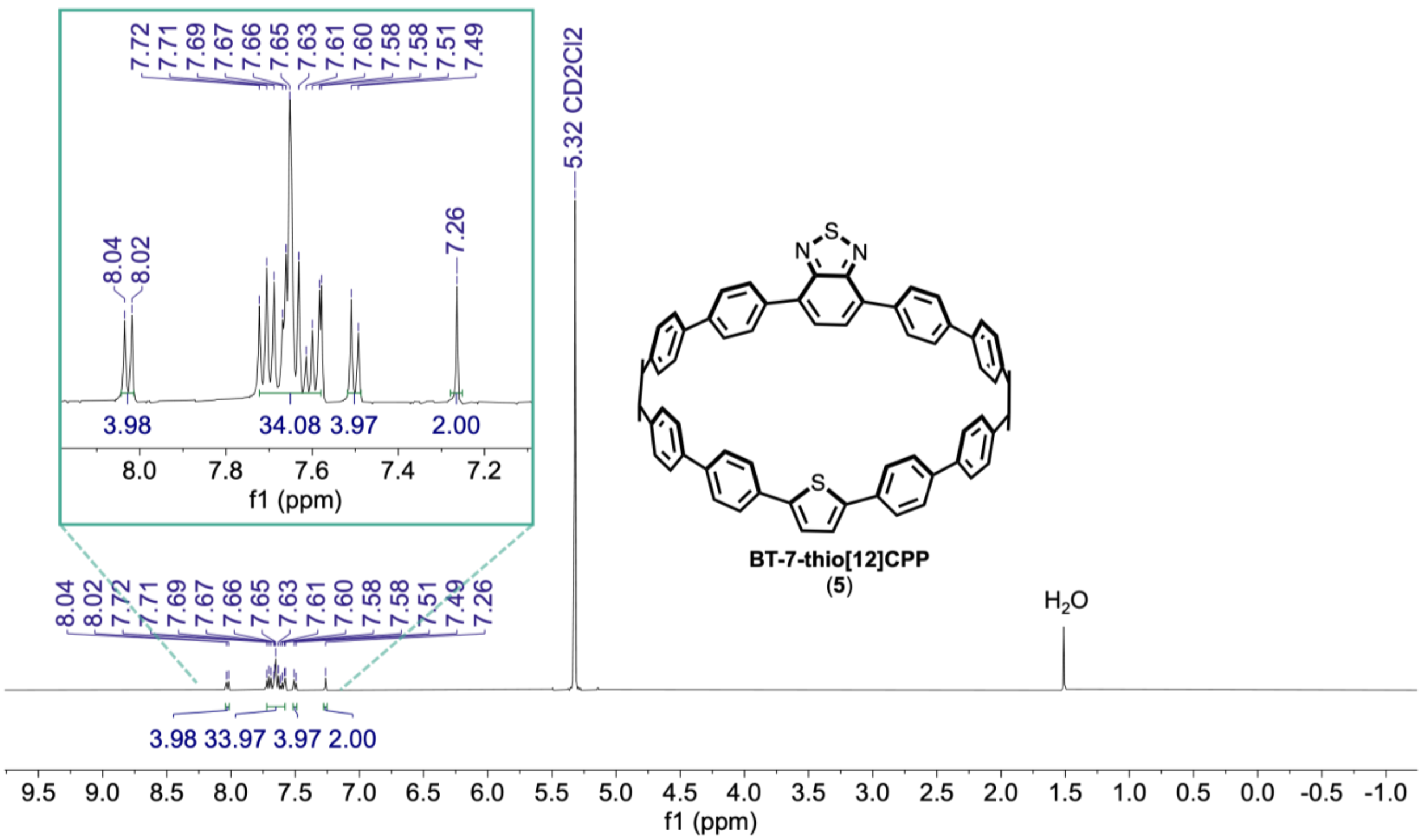


**Figure S33.** $^{1}H$ NMR spectrum (500 MHz) of **5** in Methylene Chloride-$d_2$ at room temperature.

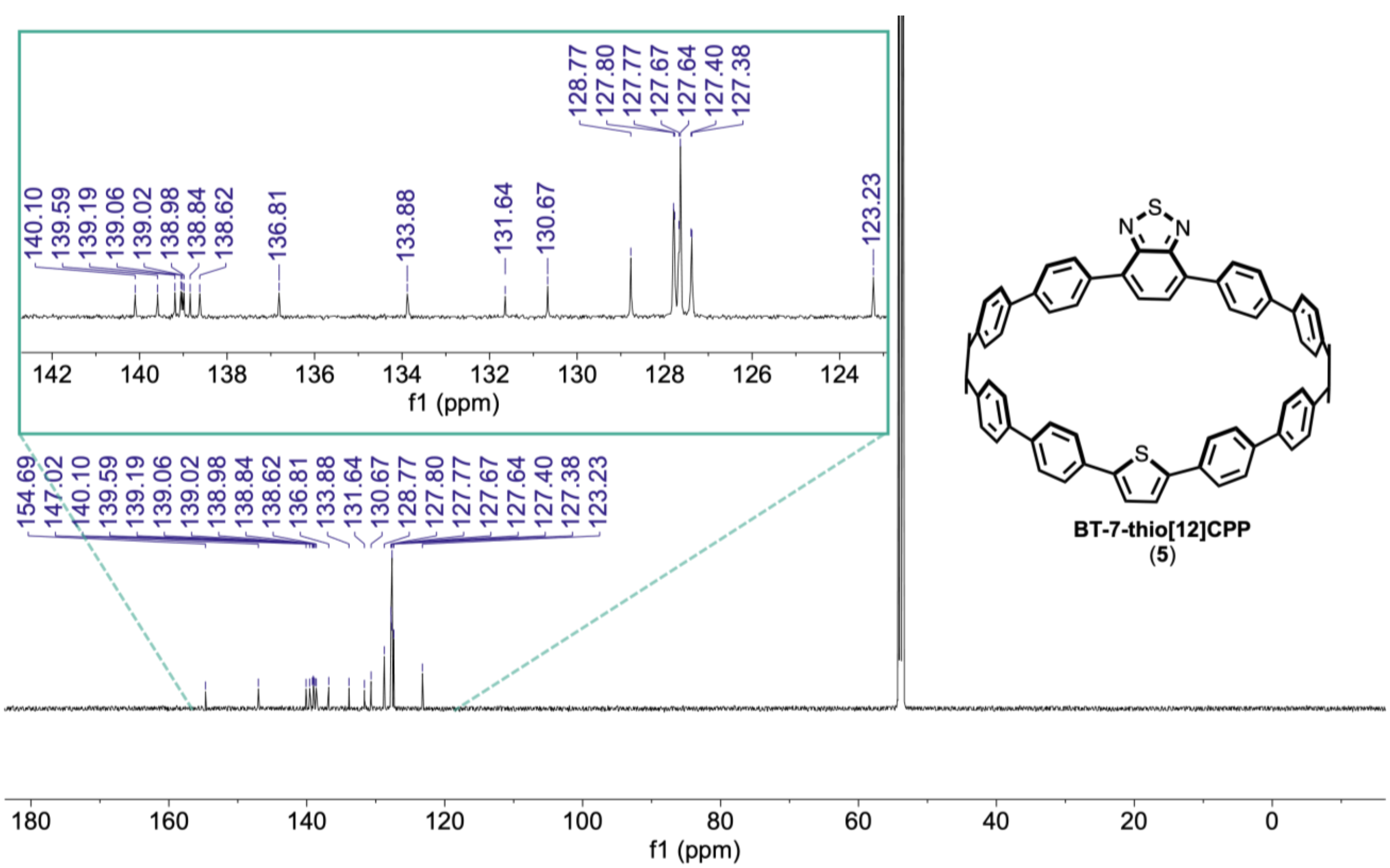


**Figure S34.** $^{13}C$ NMR spectrum (151 MHz) of **5** in Methylene Chloride-$d_2$ at room temperature.

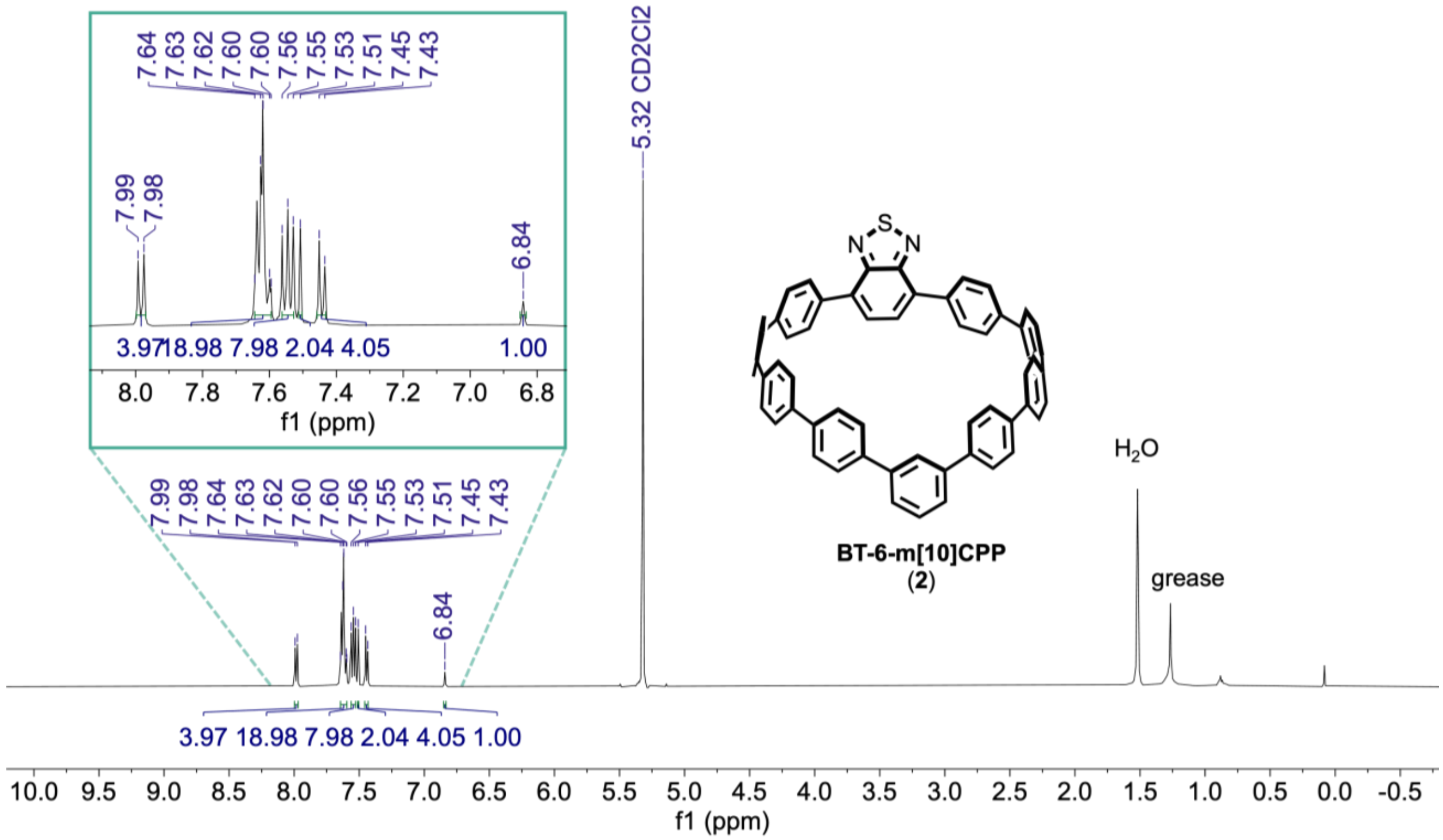


**Figure S35.** $^{1}H$ NMR spectrum (500 MHz) of **2** in Methylene Chloride-$d_2$ at room temperature.

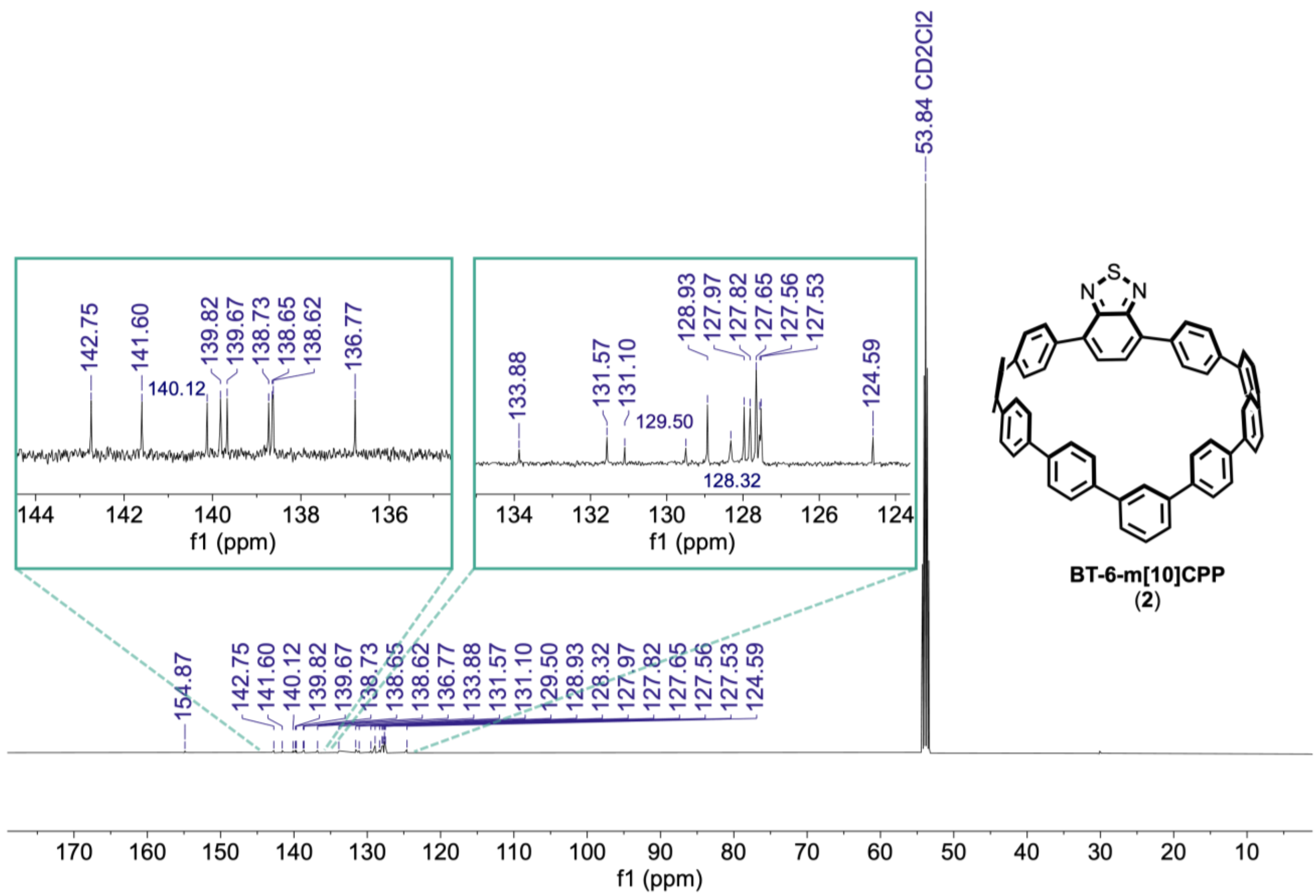


**Figure S36.** $^{13}C$ NMR spectrum (126 MHz) of **2** in Methylene Chloride-$d_2$ at room temperature.

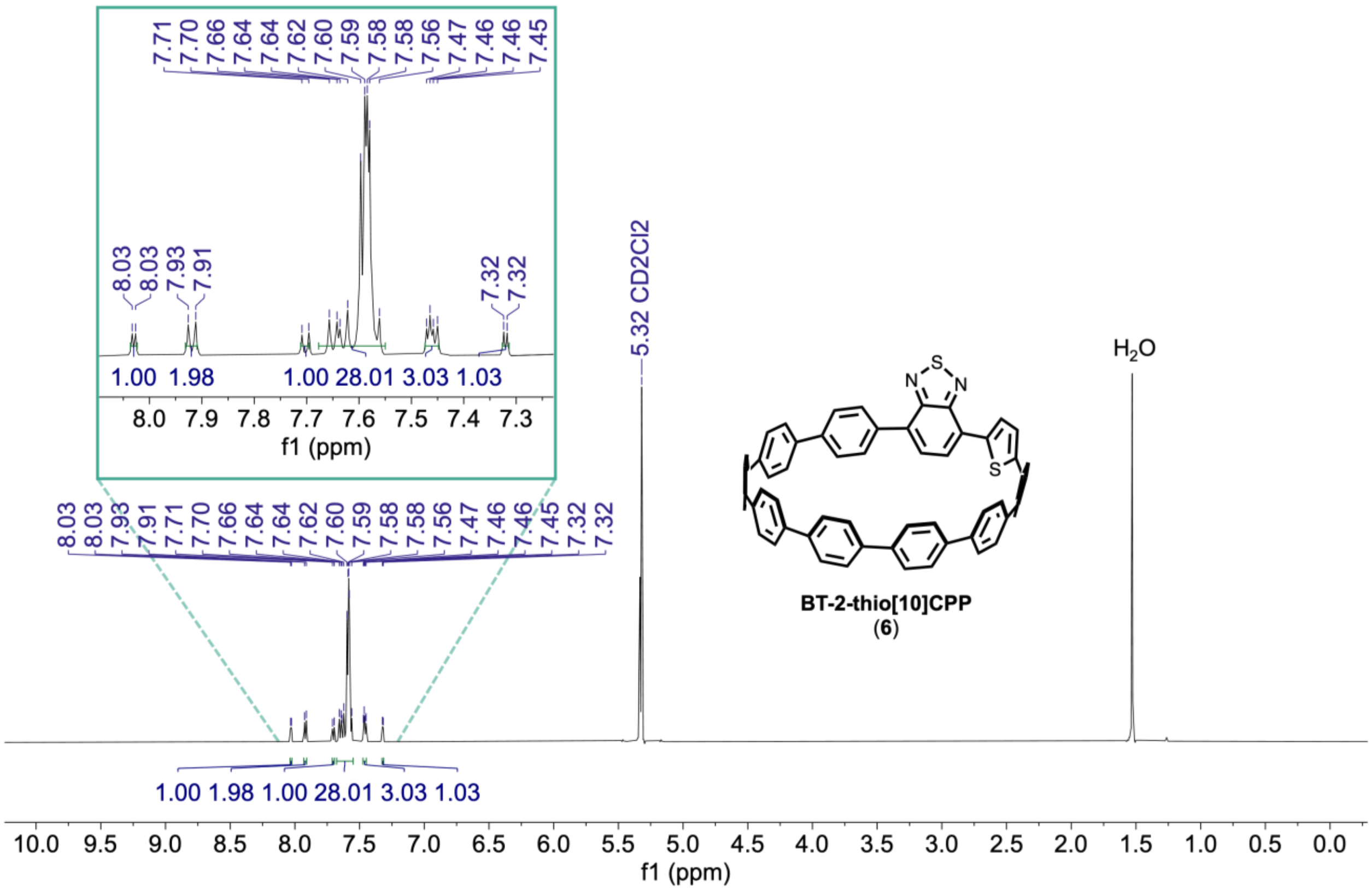

**Figure S37.** $^{1}H$ NMR spectrum (600 MHz) of **6** in Methylene Chloride-$d_2$ at room temperature.

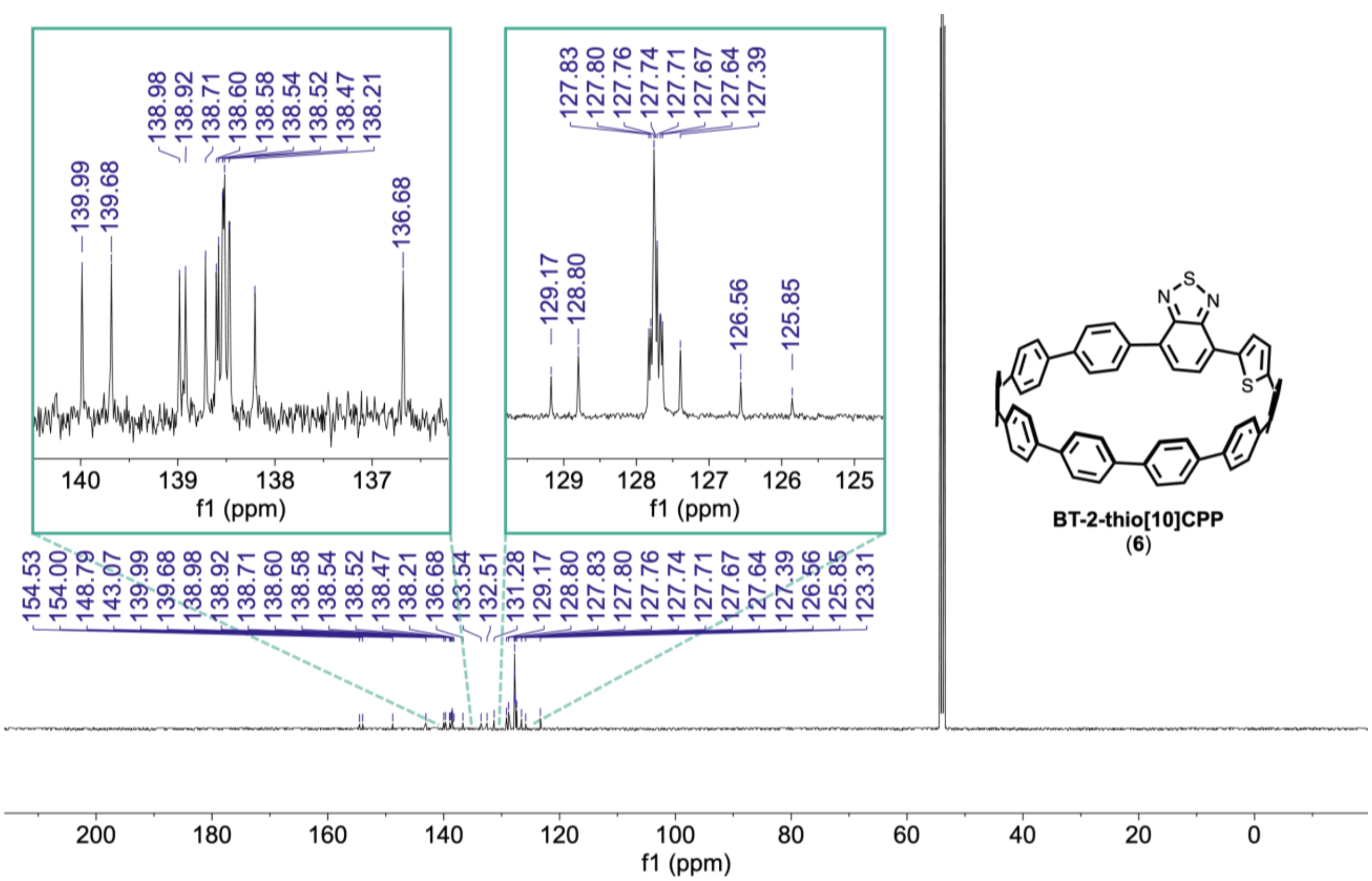

**Figure S38.** $^{13}C$ NMR spectrum (151 MHz) of **6** in Methylene Chloride-$d_2$ at room temperature.

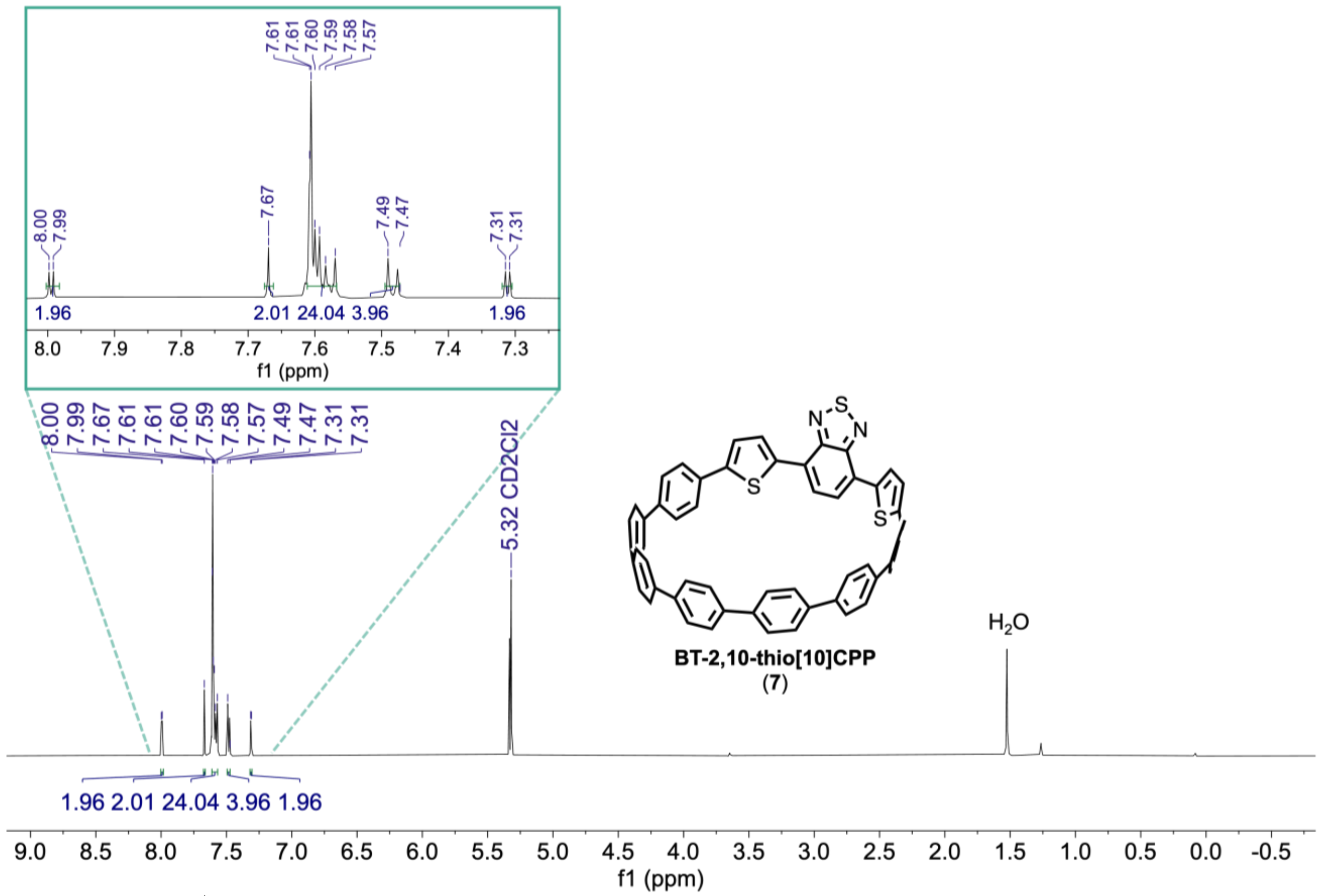


**Figure S39.** $^{1}H$ NMR spectrum (600 MHz) of **7** in Methylene Chloride-$d_2$ at room temperature.

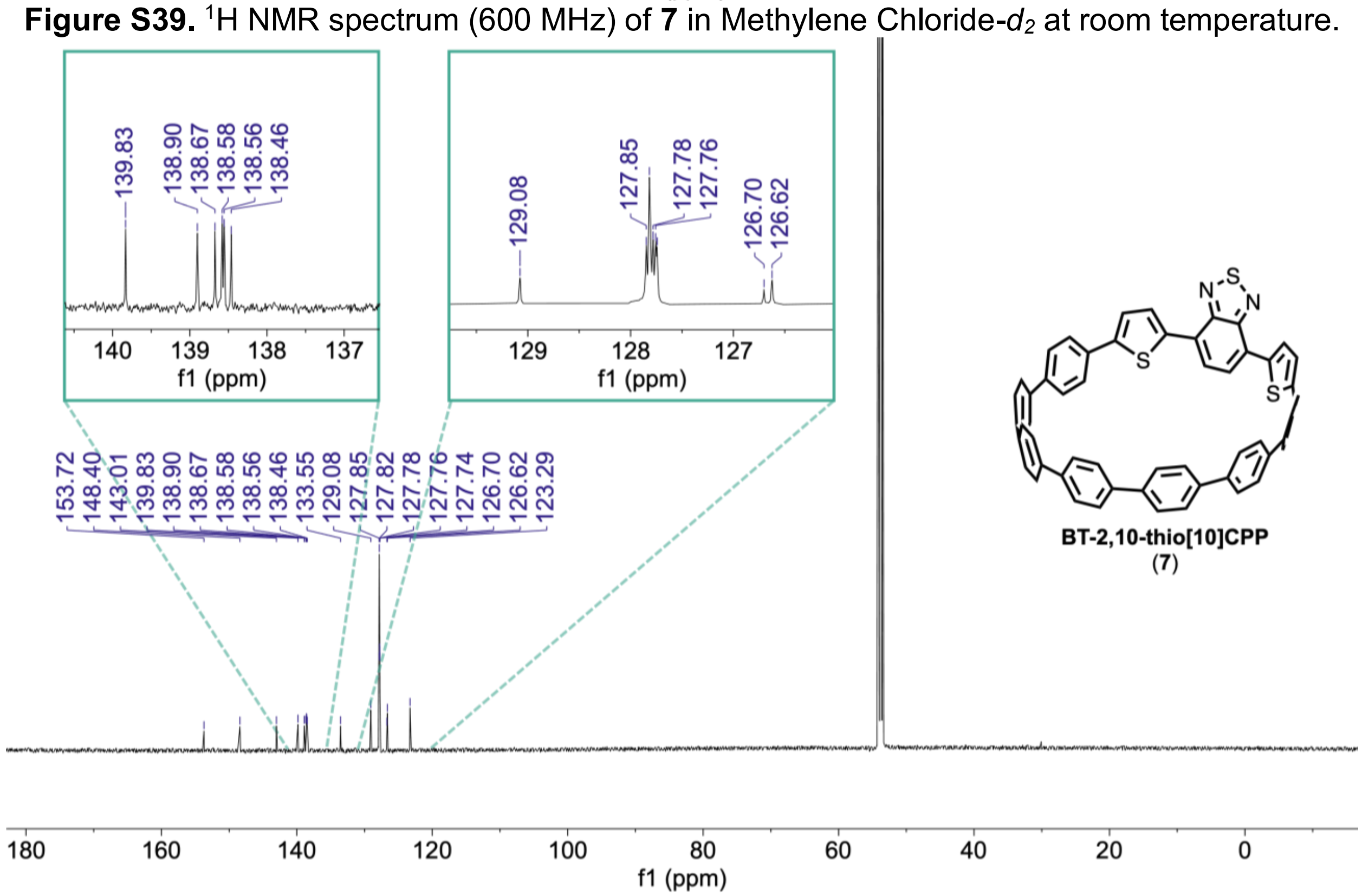


**Figure S40.** $^{13}C$ NMR spectrum (151 MHz) of **7** in Methylene Chloride-$d_2$ at room temperature.

# 4. Experimental and Computational Photophysical Characterization

## a. Experimental Photophysical Characterization

*Quantum Yield Data*

Quantum yield measurements were performed using the method described in "A Guide to Recording Fluorescence Quantum Yields" by Horiba Scientific.[9] The internal standards used were quinine sulfate (0.1 M $H_2SO_4$ aq., lit. value $\Phi$ = 0.60, fluorescence signal integrated from 400-600 nm) and anthracene (ethanol, lit. value $\Phi$ = 0.27, fluorescence signal integrated from 360-480 nm). Absorbance values plotted below were measured at 340 nm. For fluorescence measurements, all compounds were excited at a wavelength of 340 nm with consistent excitation and emission slit widths of 1 nm. Fluorescence signal integrations for compounds of interest are listed below:

**Note:** A second order diffraction peak was observed at 680 nm - two times the excitation wavelength (340 nm x 2); therefore, did not integrate ± 5 nm from this region.

**Table S1.** Compound integration ranges in DCM and DMSO.

| Compound | Integration Range in DCM (nm) | Integration Range in DMSO (nm) |
|---|---|---|
| 1 | 480 - 675 | 500 - 675 \| 685 - 700 |
| 2 | 500 - 675 \| 685 - 720 | 510 - 675 \| 685 - 730 |
| 3 | 420 - 600 | 420 - 600 |
| 4 | 505 - 675 \| 685 - 740 | 530 - 675 \| 685 - 750 |
| 5 | 480 - 675 \| 685 - 700 | 500 - 675 \| 685 - 730 |
| 6 | 520 - 675 \| 685 - 750 | 550 - 675 \| 685 - 770 |
| 7 | 550 - 675 \| 685 - 760 | 570 - 675 \| 685 - 780 |

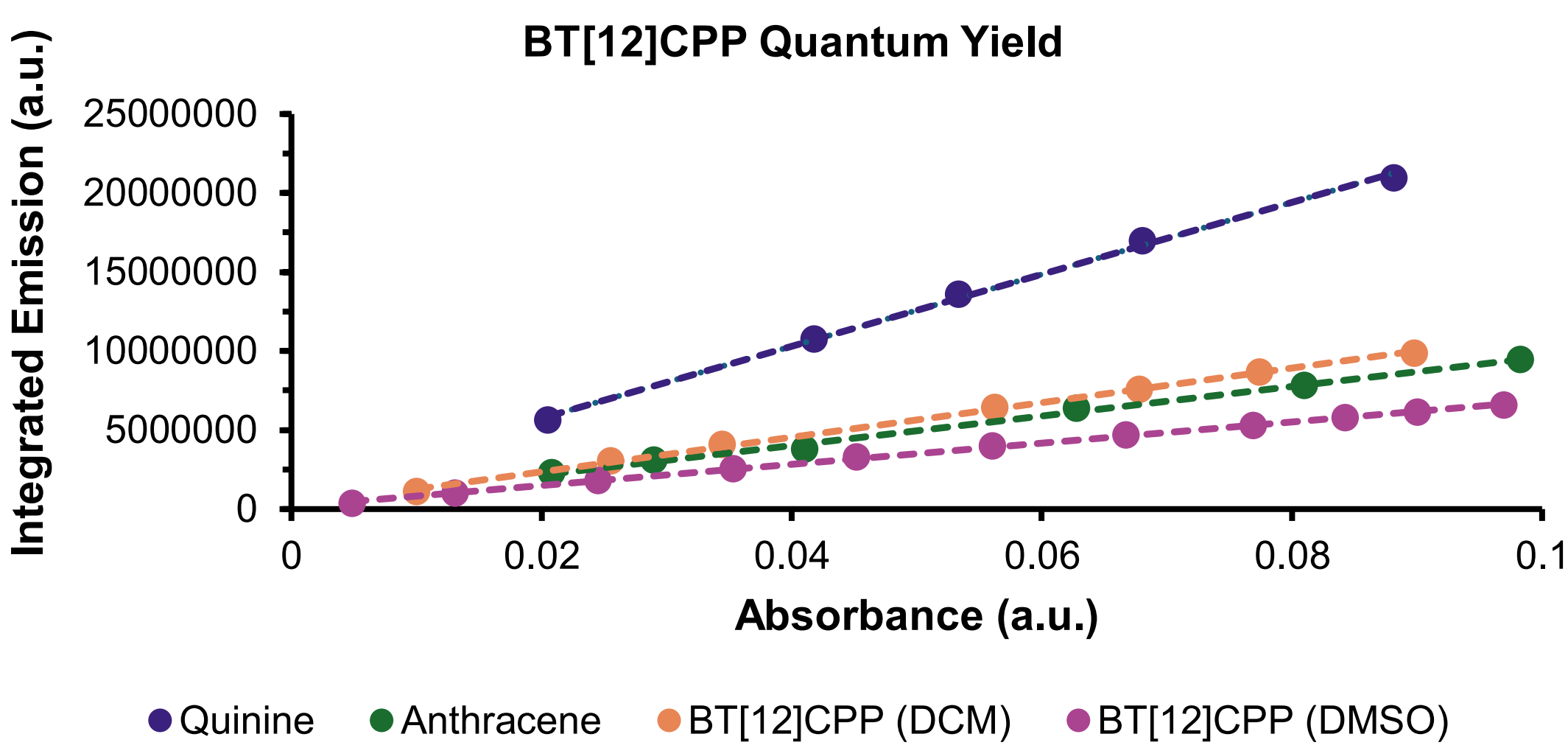


**Figure S41.** Quantum yield plot for **1**.

**Table S2**: Quantum yield data for **1** (BT[12]CPP).

| Compound | Solvent | Slope | $\phi$ w.r.t. quinine | $\phi$ w.r.t. anthracene | Avg. | Std. dev. |
|---|---|---|---|---|---|---|
| Quinine | 0.1 M $H_2SO_4$ (aq.) | $2.28x10^8$ | 0.6 (lit.) | 0.63 | - | - |
| Anthracene | EtOH | $9.35x10^7$ | 0.26 | 0.27 (lit.) | - | - |
| 1 | DCM | $1.09x10^8$ | 0.35 | 0.33 | 0.34 | 0.012 |
| 1 | DMSO | $6.70x10^7$ | 0.23 | 0.22 | 0.22 | 0.0079 |

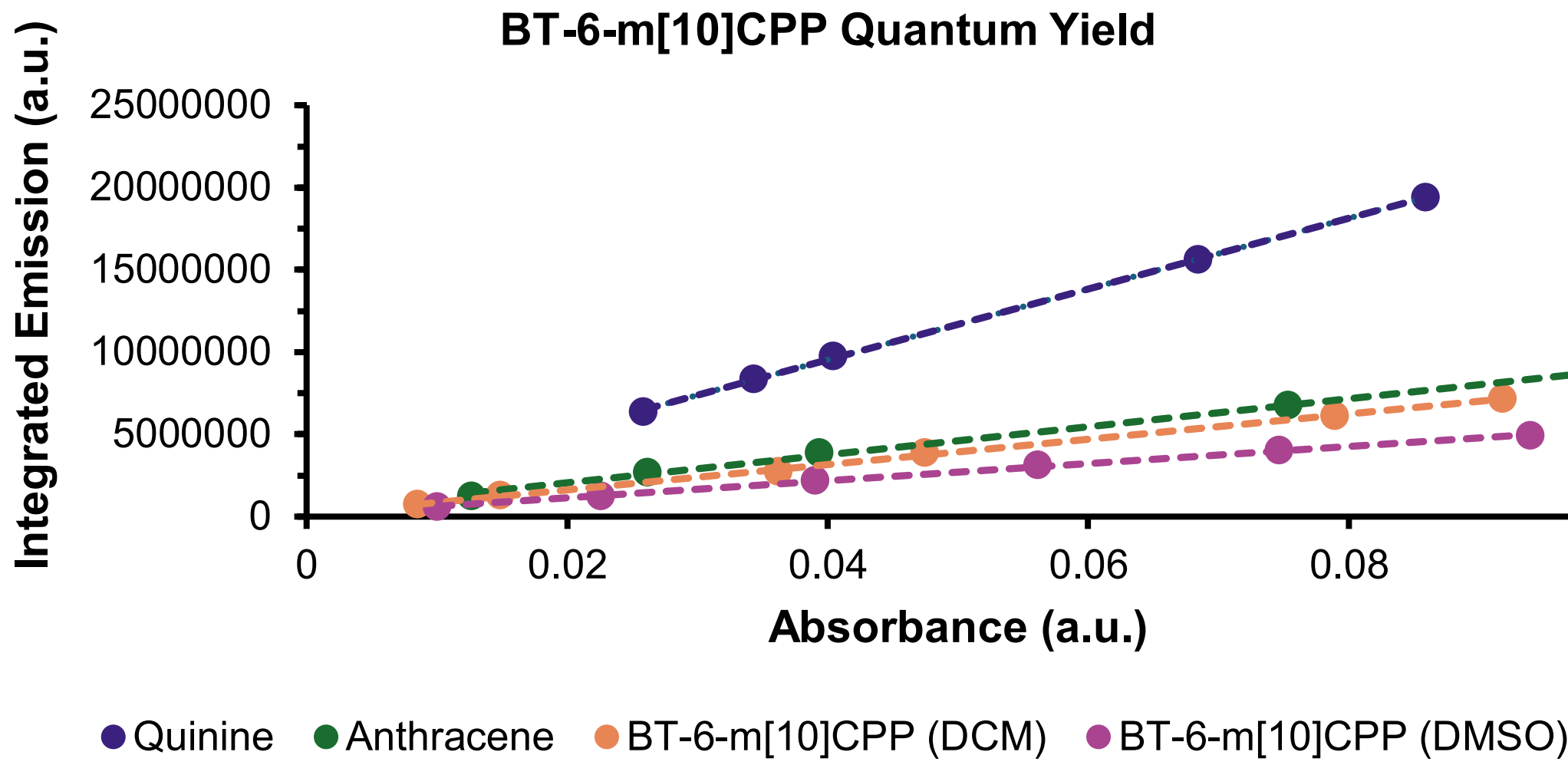


**Figure S42.** Quantum yield plot for **2**.

**Table S3**: Quantum yield data for **2** (BT-6-m[10]CPP).

| Compound | Solvent | Slope | $\phi$ w.r.t. quinine | $\phi$ w.r.t. anthracene | Avg. | Std. dev. |
|---|---|---|---|---|---|---|
| Quinine | 0.1 M $H_2SO_4$ (aq.) | $2.15x10^8$ | 0.6 (lit.) | 0.65 | - | - |
| Anthracene | EtOH | $8.51x10^7$ | 0.25 | 0.27 (lit.) | - | - |
| 2 | DCM | $7.69x10^7$ | 0.27 | 0.25 | 0.26 | 0.016 |
| 2 | DMSO | $5.20x10^7$ | 0.19 | 0.18 | 0.19 | 0.011 |

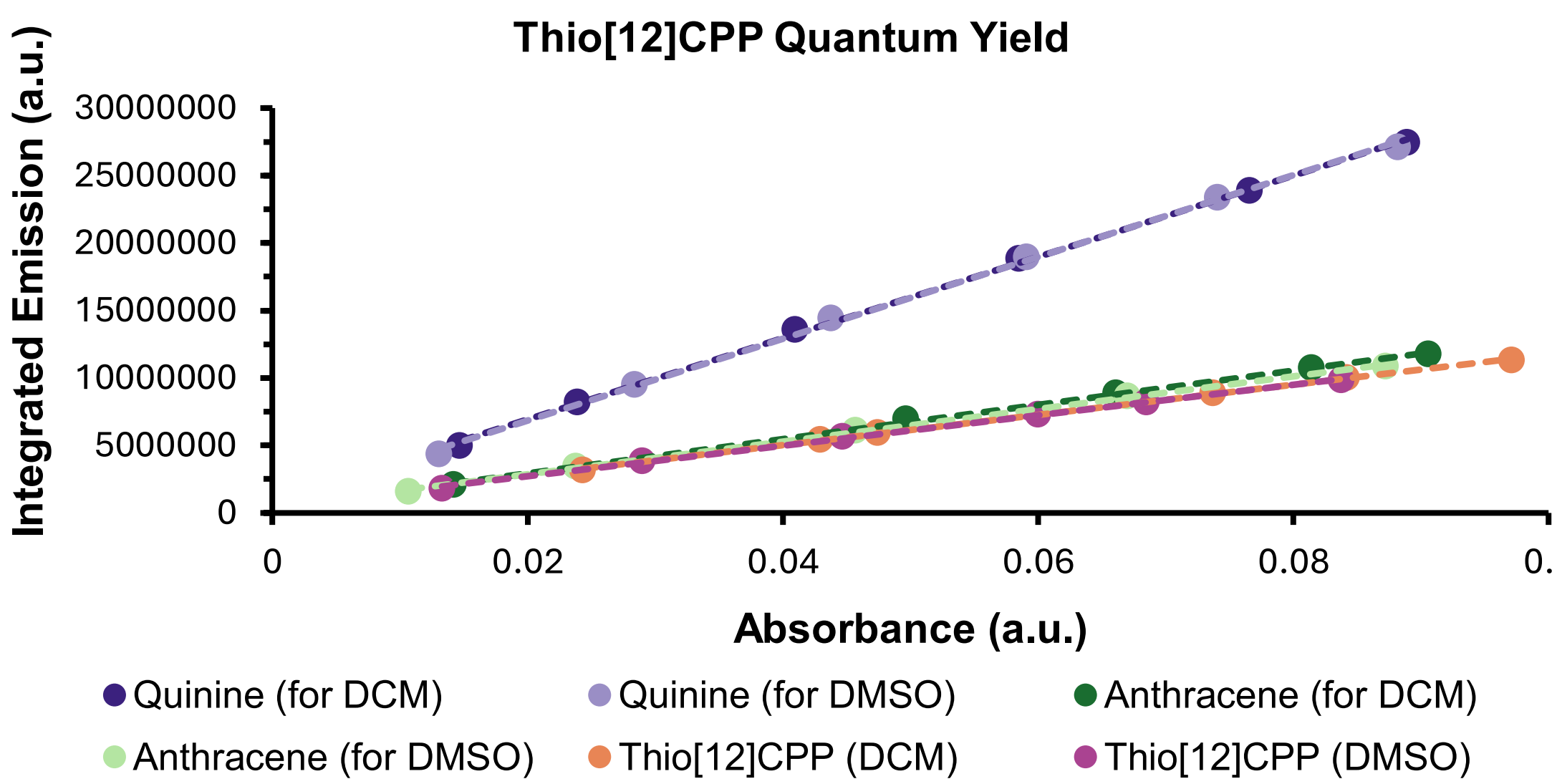


**Figure S43.** Quantum yield plot for **3**.

**Table S4**: Quantum yield data for **3** (Thio[12]CPP).

| Compound | Solvent | Slope | $\phi$ w.r.t. quinine | $\phi$ w.r.t. anthracene | Avg. | Std. dev. |
|---|---|---|---|---|---|---|
| Quinine (for DCM) | 0.1 M $H_2SO_4$ (aq.) | $3.01x10^8$ | 0.6 (lit.) | 0.61 | - | - |
| Anthracene (for DCM) | EtOH | $1.27x10^8$ | 0.26 | 0.27 (lit.) | - | - |
| 3 | DCM | $1.12x10^8$ | 0.26 | 0.25 | 0.26 | 0.0044 |
| Quinine (for DMSO) | 0.1 M $H_2SO_4$ (aq.) | $3.03x10^8$ | 0.6 (lit.) | 0.65 | - | - |
| Anthracene (for DMSO) | EtOH | $1.21x10^8$ | 0.25 | 0.27 (lit.) | - | - |
| 3 | DMSO | $1.13x10^8$ | 0.30 | 0.28 | 0.29 | 0.016 |

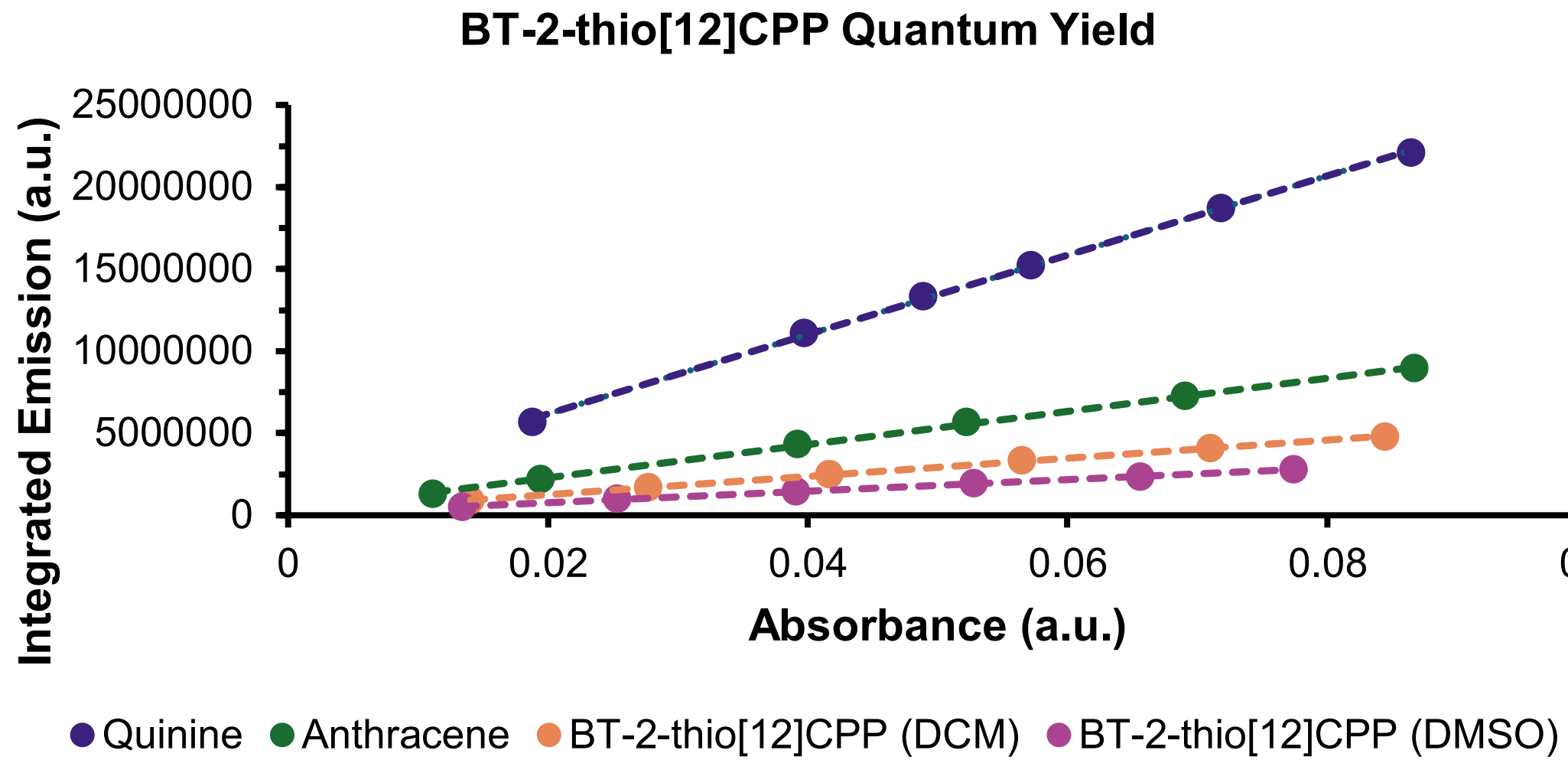


**Figure S44.** Quantum yield plot for **4**.

**Table S5**: Quantum yield data for **4** (BT-2-thio[12]CPP).

| Compound | Solvent | Slope | *ϕ* w.r.t. quinine | *ϕ* w.r.t. anthracene | Avg. | Std. dev. |
|---|---|---|---|---|---|---|
| Quinine | 0.1 M $H_2SO_4$ (aq.) | $2.42 \times 10^8$ | 0.6 (lit.) | 0.61 | - | - |
| Anthracene | EtOH | $1.03 \times 10^8$ | 0.27 | 0.27 (lit.) | - | - |
| 4 | DCM | $5.54 \times 10^7$ | 0.16 | 0.16 | 0.16 | 0.0011 |
| 4 | DMSO | $3.52 \times 10^7$ | 0.11 | 0.11 | 0.11 | 0.00078 |

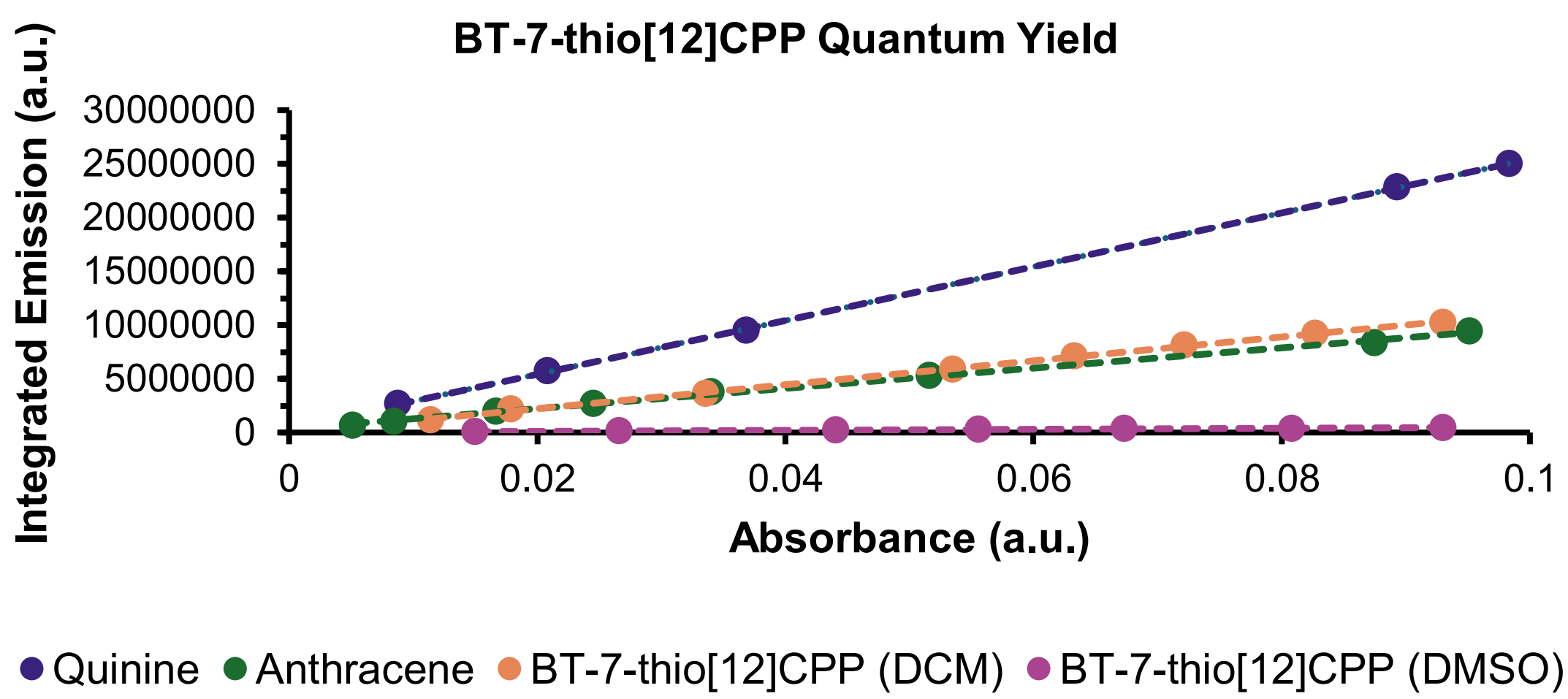


**Figure S45.** Quantum yield plot for **5**.

**Table S6**: Quantum yield data for **5** (BT-7-thio[12]CPP).

| Compound | Solvent | Slope | *ϕ* w.r.t. quinine | *ϕ* w.r.t. anthracene | Avg. | Std. dev. |
|---|---|---|---|---|---|---|
| Quinine | 0.1 M $H_2SO_4$ (aq.) | $2.50 \times 10^8$ | 0.6 (lit.) | 0.61 | - | - |
| Anthracene | EtOH | $1.06 \times 10^8$ | 0.27 | 0.27 (lit.) | - | - |
| 5 | DCM | $1.11 \times 10^8$ | 0.31 | 0.30 | 0.31 | 0.0033 |
| 5 | DMSO | $4.83 \times 10^6$ | 0.01 | 0.01 | 0.01 | 0.00016 |

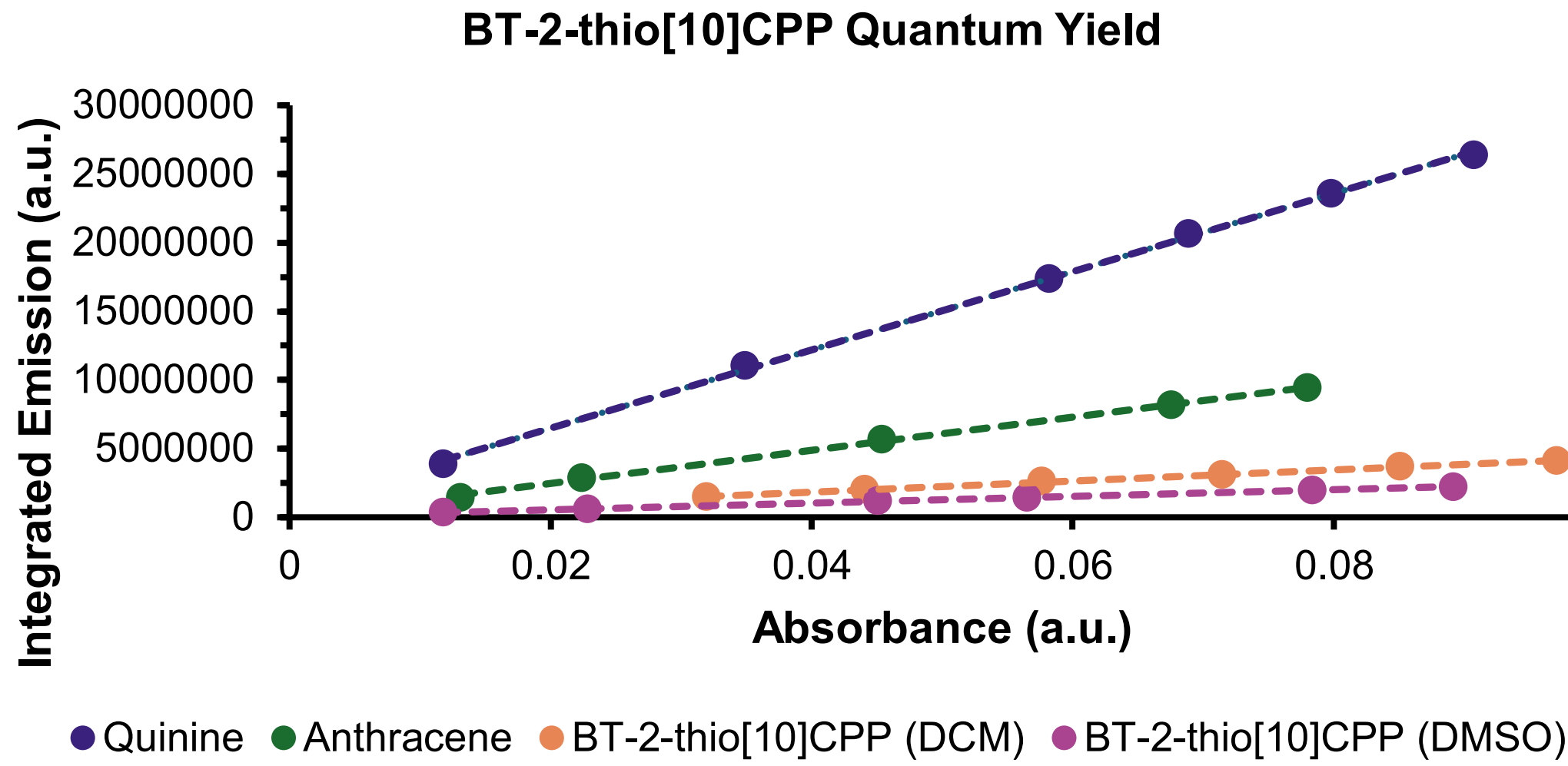


**Figure S46.** Quantum yield plot for **6**.

**Table S7**: Quantum yield data for **6** (BT-2-thio[10]CPP).

| Compound | Solvent | Slope | $\phi$ w.r.t. quinine | $\phi$ w.r.t. anthracene | Avg. | Std. dev. |
|---|---|---|---|---|---|---|
| Quinine | 0.1 M $H_2SO_4$ (aq.) | $2.85x10^8$ | 0.6 (lit.) | 0.61 | - | - |
| Anthracene | EtOH | $1.21x10^8$ | 0.27 | 0.27 (lit.) | - | - |
| 6 | DCM | $4.05x10^7$ | 0.10 | 0.10 | 0.10 | 0.0013 |
| 6 | DMSO | $2.45x10^7$ | 0.06 | 0.06 | 0.06 | 0.00085 |

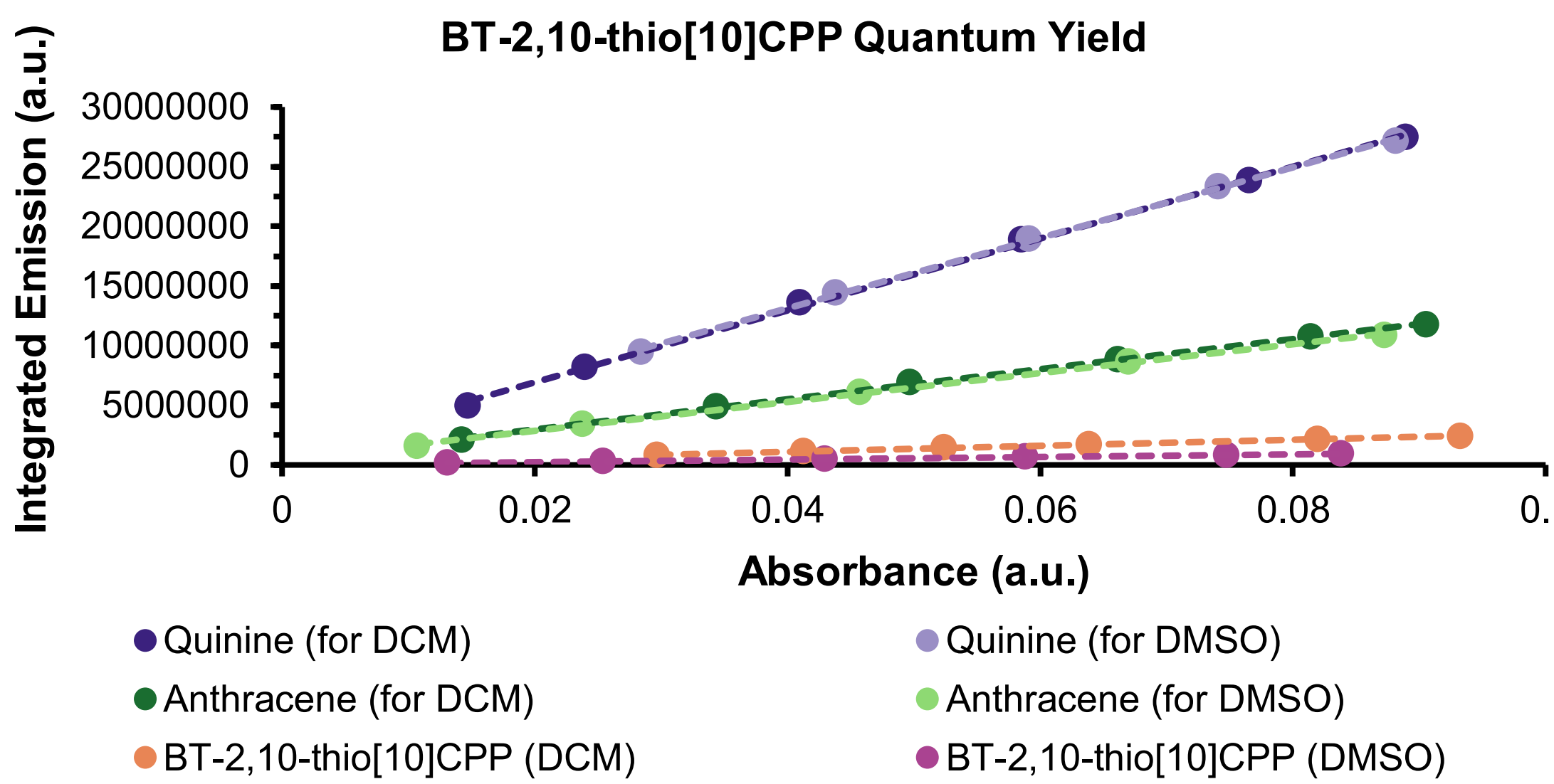


**Figure S47.** Quantum yield plot for **7**.

**Table S8**: Quantum yield data for **7** (BT-2,10-thio[10]CPP).

| Compound | Solvent | Slope | $\phi$ w.r.t. quinine | $\phi$ w.r.t. anthracene | Avg. | Std. dev. |
|---|---|---|---|---|---|---|
| Quinine (for DCM) | 0.1 M $H_2SO_4$ (aq.) | $3.01x10^8$ | 0.6 (lit.) | 0.62 | - | - |
| Anthracene (for DCM) | EtOH | $1.26x10^8$ | 0.26 | 0.27 (lit.) | - | - |
| 7 | DCM | $2.53x10^7$ | 0.06 | 0.06 | 0.06 | 0.0012 |
| Quinine (for DMSO) | 0.1 M $H_2SO_4$ (aq.) | $2.95x10^8$ | 0.6 (lit.) | 0.63 | - | - |
| Anthracene (for DMSO) | EtOH | $1.21x10^8$ | 0.26 | 0.27 (lit.) | - | - |
| 7 | DMSO | $1.09x10^7$ | 0.03 | 0.03 | 0.03 | 0.001 |

*Solvatofluorochromism Data*

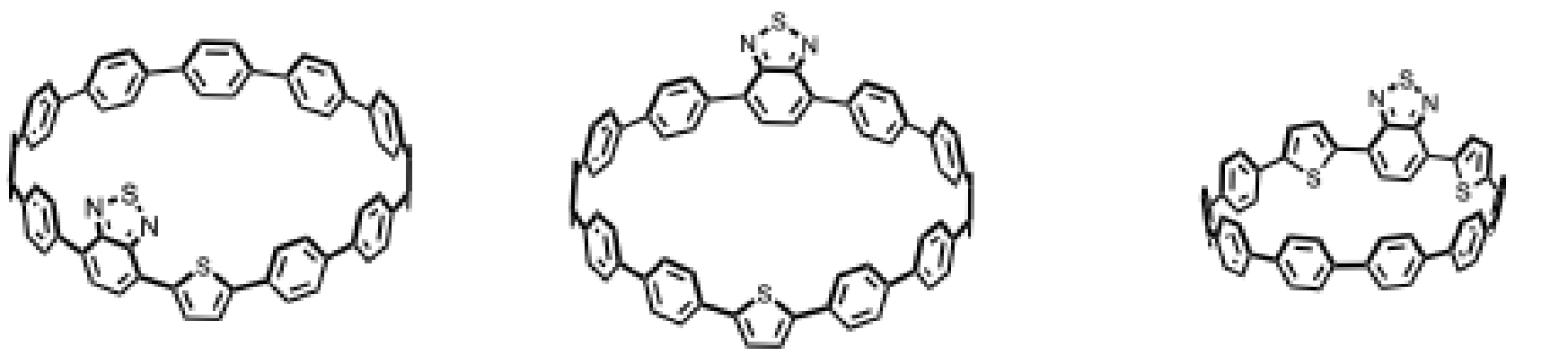

BT-2-thio[12]CPP (4) BT-7-thio[12]CPP (5) BT-2,10-thio[10]CPP (7)

**Table S9**: Tabulated solvatofluorochromism data for compounds **4**, **5**, and **7**.

| Compound | Major $\lambda_{abs}$ (nm) $CCl_4$, DCM, DMSO | Minor $\lambda_{abs}$ (nm) $CCl_4$, DCM, DMSO | $\lambda_{em}$ (nm) $CCl_4$, DCM, DMSO |
|---|---|---|---|
| **4** | 340, 339, 348 | 467, 457, 470 | 575, 599, 622 |
| **5** | 340, 340, 349 | - | 550, 565, 587 |
| **7** | 342, 342, 350 | 512, 495, 510 | 613, 639, 662 |

The Lippert-Mataga **Equation (1)**[10] was used to plot the effective Stokes shift ($\Delta\nu$) in wavenumbers versus the orientation polarizability ($\Delta f$) of the solvent. The orientation polarizability was determined by using lit. known values for the refractive index ($\eta$) and the dielectric constant ($\varepsilon$) of the solvent. A large slope means a large dipole moment variation ($\Delta\mu$), which is the difference between the excited state dipole moment ($\mu_e$) and the ground state dipole moment ($\mu_g$).

**Equation (1)**: $$\Delta\nu = \frac{2(\Delta\mu)^2}{r^3hc}\Delta f + C \text{ with } \Delta f = \left(\frac{\varepsilon-1}{2\varepsilon+1} - \frac{\eta^2-1}{\eta^2+1}\right)$$

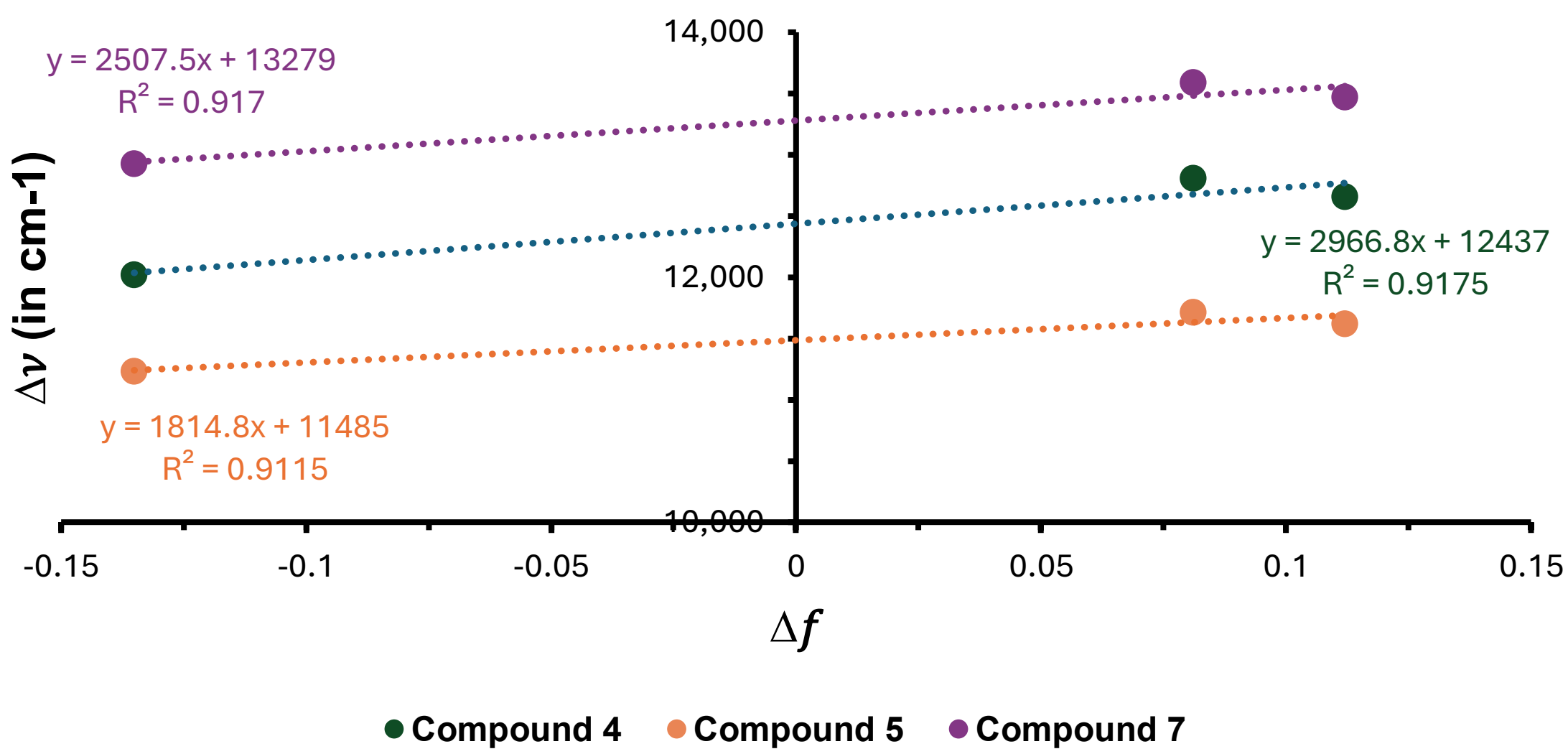


**Figure S48.** Lippert-Mataga plot for compounds **4**, **5**, and **7**.

**Table S10**: Tabulated solvatofluorochromism data for compounds **4**, **5**, and **7**.

| Compound | $\Delta\nu$ ($cm^{-1}$) $CCl_4$ | $\Delta\nu$ ($cm^{-1}$) DCM | $\Delta\nu$ ($cm^{-1}$) DMSO | $\varepsilon$ $CCl_4$, DCM, DMSO | $\eta$ $CCl_4$, DCM, DMSO | $\Delta f$ $CCl_4$, DCM, DMSO |
|---|---|---|---|---|---|---|
| **4** | 12,020 | 12,804 | 12,658 | 2.24, 8.93, 46.7 | 1.4601, 1.4241, 1.4783 | -0.1351, 0.0810, 0.1120 |
| **5** | 11,230 | 11,713 | 11,618 | | | |
| **7** | 12,927 | 13,590 | 13,466 | | | |

## b. Data for Literature Reported Small Molecule, Linear Analogues

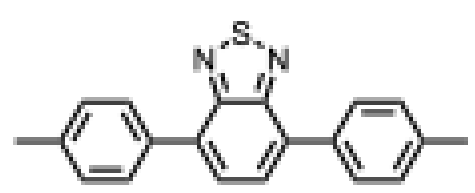

**S13**

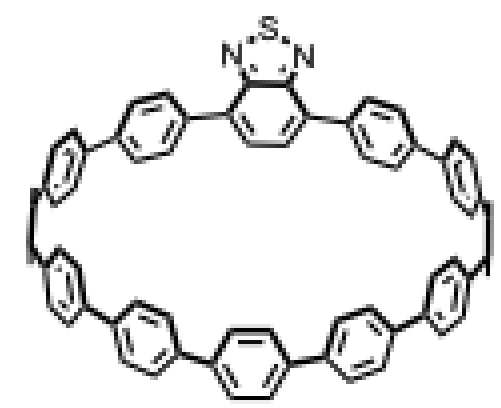

**BT[12]CPP (1)**

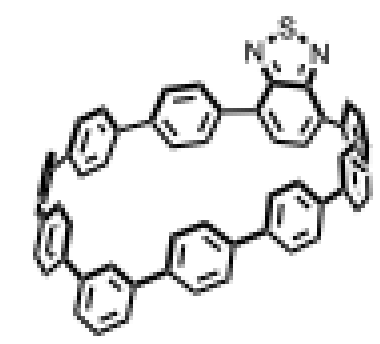

**BT-6-m[10]CPP (2)**

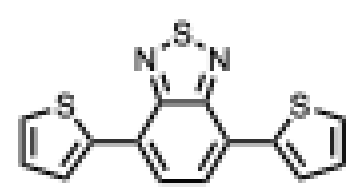

**S14**

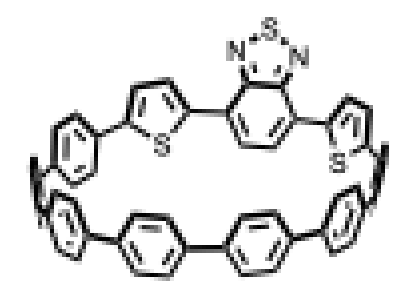

**BT-2,10-thio[10]CPP (7)**

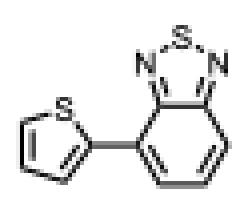

**S15**

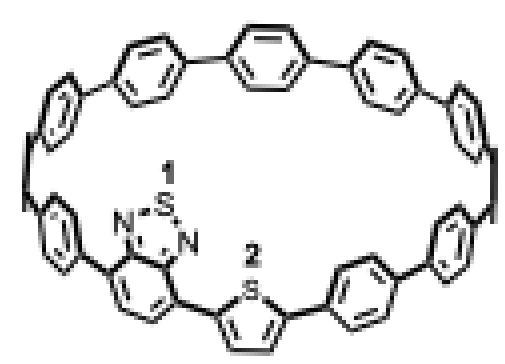

**BT-2-thio[12]CPP (4)**

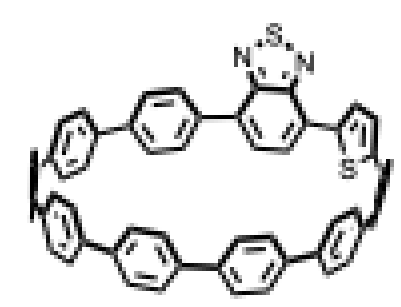

**BT-2-thio[10]CPP (6)**

**Table S11**: Literature absorbance and emission values for the small molecule, linear analogues (**S13**, **S14**, and **S15**)[7,11,12] of D-A nanohoops **1**, **2**, **7** (in DCM), and **4** and **6** (in DMSO).

| Compound | Solvent | $\lambda_{abs}$ (HOMO→LUMO, nm) | $\lambda_{em}$ (nm) |
|---|---|---|---|
| **S13** | DCM | 390 | 505 |
| **1** | | 440 | 558 |
| **2** | | 454 | 583 |
| **S14** | | 445 | 577 |
| **7** | | 495 | 639 |
| **S15** | DMSO | - | 536 |
| **4** | | 470 | 622 |
| **6** | | 473 | 631 |

### c. Computational Photophysical Characterization

*Predicted Absorbance and Emission Values*

All calculations were performed using Gaussian 09. All calculations were conducted using the CAM-B3LYP function, the 6-31G** basis set, and the effect of the solvent was included by the polarizable continuum model (PCM) using either dichloromethane or dimethylsulfoxide as the solvent.

**Geometry optimizations:** Initial, ground state geometries (S0 orbitals) were obtained using the keyword “Opt Freq”.

**Predicted absorbance data:** The ground state geometry optimizations were used to perform a time dependent calculation of 12 state to give predicted UV-vis spectra of **1**-**7**, **BT[10]CPP**, and **Thio[10]CPP**. Calculations were run using the keyword “TD=NSTATE=12”. The results were analyzed using GaussSum.

**Excited state geometries:** The ground state geometry optimizations were used to perform a time dependent optimization to yield the excited state geometries (S1’ orbitals). Calculations were run using the keyword “Opt TD”.

**Predicted fluorescence data:** The excited state geometry optimizations were used to perform a time dependent calculation of 12 state to give predicted fluorescence spectra of **1**-**7**, **BT[10]CPP**, and **Thio[10]CPP**. Calculations were run using the keyword “TD=NSTATE=12”.

**Note:** Molecular orbital transitions ascribed to major (light blue) and minor (light green) absorbance and emission maxima values are highlighted below.

Link to computational data on FigShare: https://doi.org/10.6084/m9.figshare.32719806

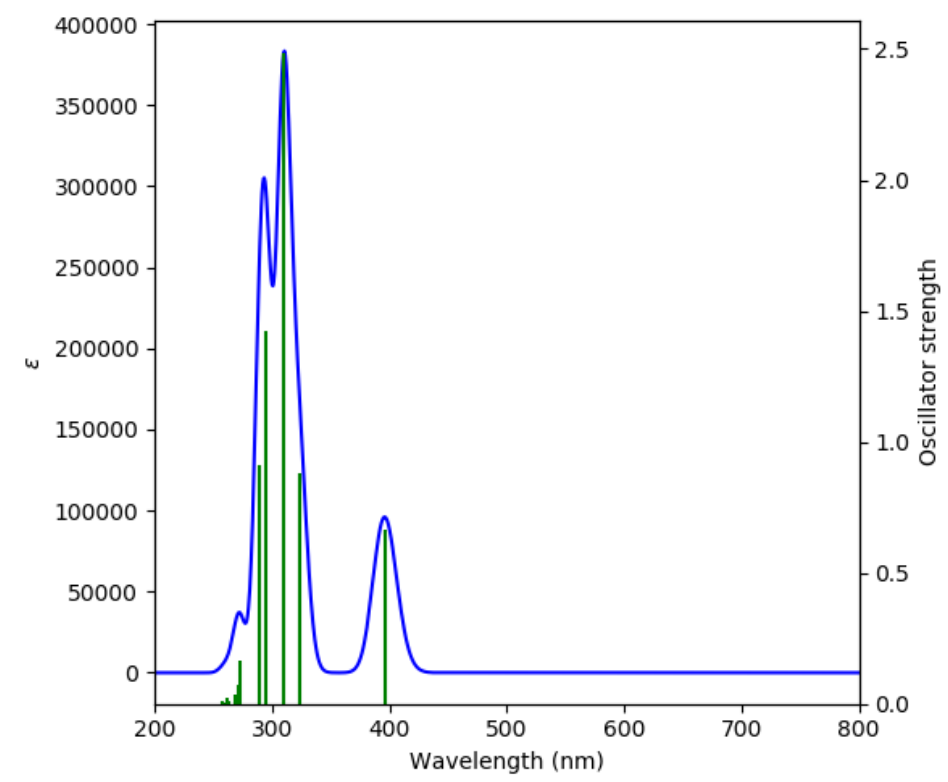


| No. | Energy (cm$^{-1}$) | $\lambda$ (nm) | Osc. Strength | Major Contributions |
|---|---|---|---|---|
| 1 | 25250.7995 | 396.0270 | 0.6632 | H-1->LUMO (37%), HOMO->LUMO (45%) |
| 2 | 30934.5885 | 323.2627 | 0.8819 | H-2->L+2 (11%), H-1->L+1 (18%), HOMO->L+1 (43%) |
| 3 | 32233.9477 | 310.2319 | 2.4864 | H-2->LUMO (26%), H-2->L+1 (12%), HOMO->L+2 (23%) |
| 4 | 33909.1613 | 294.9055 | 1.4223 | H-1->L+1 (19%), HOMO->L+3 (29%) |
| 5 | 34547.9524 | 289.4527 | 0.9123 | H-2->LUMO (16%), H-2->L+1 (20%), HOMO->L+2 (11%) |
| 6 | 36584.5023 | 273.3397 | 0.1649 | H-18->LUMO (49%) |
| 7 | 36921.6420 | 270.8438 | 0.0693 | H-1->LUMO (17%), HOMO->LUMO (41%) |
| 8 | 37300.7226 | 268.0913 | 0.0360 | H-18->LUMO (18%), H-3->L+1 (11%), HOMO->L+4 (11%) |
| 9 | 37916.1237 | 263.7400 | 0.0084 | H-4->L+1 (16%), H-1->L+3 (11%), HOMO->L+5 (16%) |
| 10 | 38187.1259 | 261.8683 | 0.0225 | H-8->LUMO (10%) |
| 11 | 38656.5406 | 258.6884 | 0.0027 | H-7->L+1 (15%), H-1->L+6 (25%), HOMO->L+6 (20%) |
| 12 | 38846.0809 | 257.4262 | 0.0104 | H-24->LUMO (16%) |

**Figure S49.** Calculated absorbance spectrum and electronic transitions for **1** in DCM.

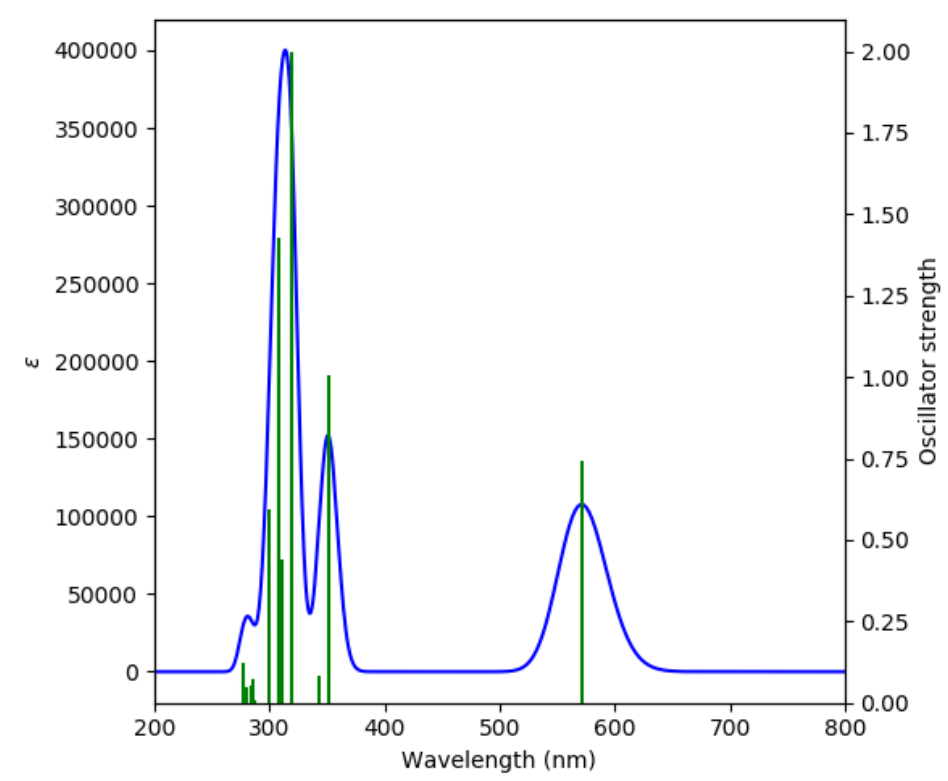


| No. | Energy (cm$^{-1}$) | $\lambda$ (nm) | Osc. Strength | Major Contributions |
|---|---|---|---|---|
| 1 | 17503.8442 | 571.3030 | 0.7437 | HOMO->LUMO (92%) |
| 2 | 28474.5975 | 351.1902 | 1.0074 | H-4->LUMO (20%), H-2->LUMO (56%) |
| 3 | 29209.3686 | 342.3559 | 0.0818 | H-2->L+2 (10%), HOMO->L+1 (22%), HOMO->L+3 (33%) |
| 4 | 31383.0328 | 318.6435 | 1.9996 | H-1->L+1 (45%), HOMO->L+3 (20%) |
| 5 | 32243.6264 | 310.1388 | 0.4377 | H-3->LUMO (14%), H-1->LUMO (45%) |
| 6 | 32505.7565 | 307.6378 | 1.4297 | H-2->L+1 (11%), HOMO->L+2 (31%) |
| 7 | 33417.1631 | 299.2474 | 0.5931 | H-18->LUMO (30%) |
| 8 | 34790.7252 | 287.4329 | 0.0079 | H-5->LUMO (10%), H-3->LUMO (23%), H-1->LUMO (20%) |
| 9 | 35108.5077 | 284.8312 | 0.0734 | H-17->LUMO (19%) |
| 10 | 35262.5596 | 283.5869 | 0.0525 | H-3->LUMO (11%) |
| 11 | 35674.7089 | 280.3106 | 0.0495 | H-18->LUMO (32%), H-4->LUMO (11%) |
| 12 | 36072.3402 | 277.2207 | 0.1214 | H-4->LUMO (17%), H-2->LUMO (20%) |

**Figure S50.** Calculated emission spectrum and electronic transitions for **1** in DCM.

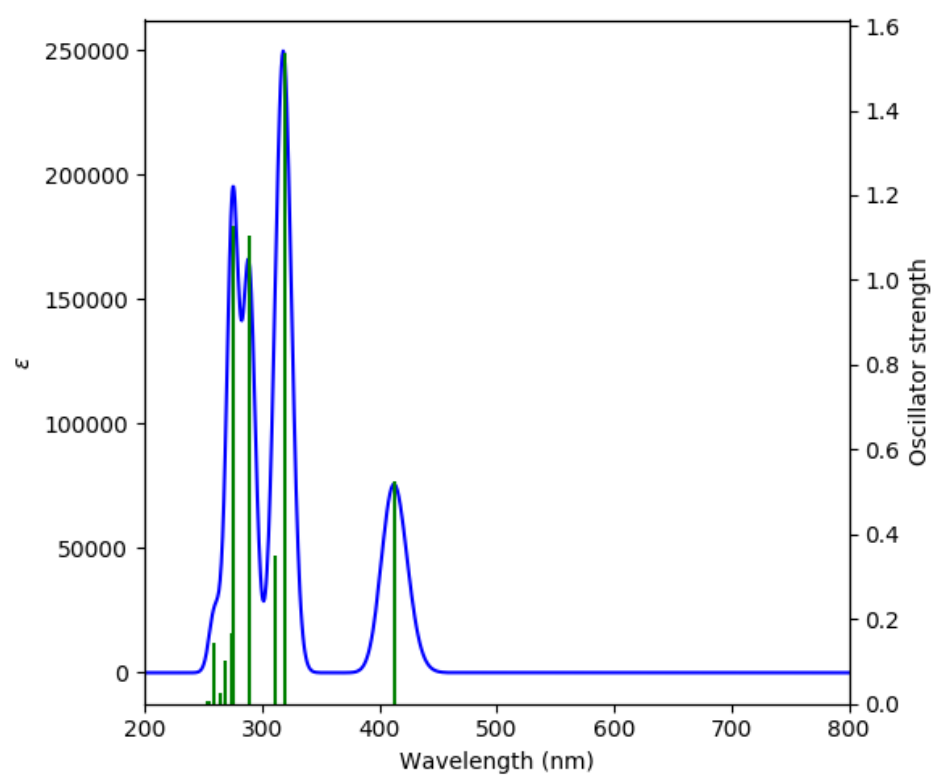


| No. | Energy (cm$^{-1}$) | $\lambda$ (nm) | Osc. Strength | Major Contributions |
|---|---|---|---|---|
| 1 | 24240.1868 | 412.5380 | 0.5233 | H-2->LUMO (12%), HOMO->LUMO (79%) |
| 2 | 31338.6723 | 319.0945 | 1.5367 | H-1->LUMO (37%), H-1->L+1 (14%), HOMO->L+1 (27%) |
| 3 | 32093.6072 | 311.5885 | 0.3486 | H-1->LUMO (13%), H-1->L+2 (17%), HOMO->L+1 (15%), HOMO->L+2 (26%) |
| 4 | 34596.3456 | 289.0478 | 1.1039 | H-1->LUMO (21%), H-1->L+1 (11%), HOMO->L+1 (12%), HOMO->L+2 (17%) |
| 5 | 36269.9461 | 275.7103 | 1.1292 | H-2->L+2 (16%), HOMO->L+3 (16%) |
| 6 | 36543.3680 | 273.6474 | 0.1648 | H-15->LUMO (47%) |
| 7 | 37244.2638 | 268.4977 | 0.1037 | H-4->LUMO (19%), H-2->LUMO (32%), HOMO->LUMO (14%) |
| 8 | 37729.8096 | 265.0424 | 0.0083 | H-6->LUMO (10%), H-6->L+1 (12%), H-1->L+5 (12%), HOMO->L+5 (24%) |
| 9 | 37845.1469 | 264.2346 | 0.0264 | H-15->LUMO (15%), H-7->LUMO (14%), HOMO->L+6 (13%) |
| 10 | 38564.5934 | 259.3052 | 0.1427 | H-2->L+1 (13%), H-2->L+2 (14%), HOMO->L+4 (11%) |
| 11 | 39128.3750 | 255.5690 | 0.0068 | H-20->LUMO (21%), H-13->LUMO (10%) |
| 12 | 39507.4555 | 253.1167 | 0.0075 | H-20->LUMO (31%) |

**Figure S51.** Calculated absorbance spectrum and electronic transitions for **2** in DCM.

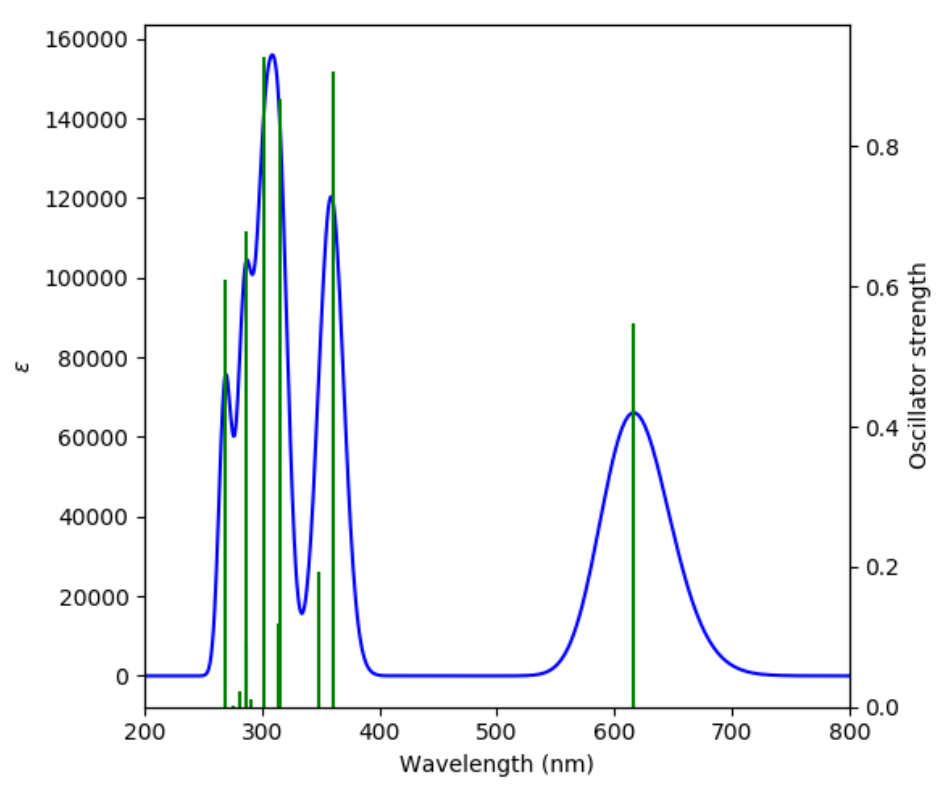


| No. | Energy (cm$^{-1}$) | $\lambda$ (nm) | Osc. Strength | Major Contributions |
|---|---|---|---|---|
| 1 | 16217.3899 | 616.6220 | 0.5474 | HOMO->LUMO (95%) |
| 2 | 27760.7968 | 360.2202 | 0.9065 | H-3->LUMO (19%), H-1->LUMO (69%) |
| 3 | 28751.2457 | 347.8110 | 0.1936 | HOMO->L+1 (61%), HOMO->L+3 (12%) |
| 4 | 31765.3396 | 314.8085 | 0.8683 | H-15->LUMO (18%), HOMO->L+2 (33%), HOMO->L+4 (11%) |
| 5 | 31816.9591 | 314.2977 | 0.1184 | H-4->LUMO (15%), H-2->LUMO (57%) |
| 6 | 33135.6756 | 301.7895 | 0.9286 | H-15->LUMO (16%), HOMO->L+2 (23%) |
| 7 | 34401.9660 | 290.6810 | 0.0118 | H-13->LUMO (27%), H-11->LUMO (25%), H-9->LUMO (12%) |
| 8 | 34905.2560 | 286.4898 | 0.0902 | H-15->LUMO (38%) |
| 9 | 34983.4918 | 285.8491 | 0.6800 | H-2->L+1 (18%), H-1->L+2 (30%), HOMO->L+3 (17%) |
| 10 | 35609.3780 | 280.8248 | 0.0222 | H-3->LUMO (39%), H-1->LUMO (18%) |
| 11 | 36297.3689 | 275.5020 | 0.0023 | H-20->LUMO (24%), H-19->LUMO (50%) |
| 12 | 37186.9985 | 268.9111 | 0.6098 | H-2->L+2 (28%), H-1->L+1 (11%), HOMO->L+4 (14%) |

**Figure S52.** Calculated emission spectrum and electronic transitions for **2** in DCM.

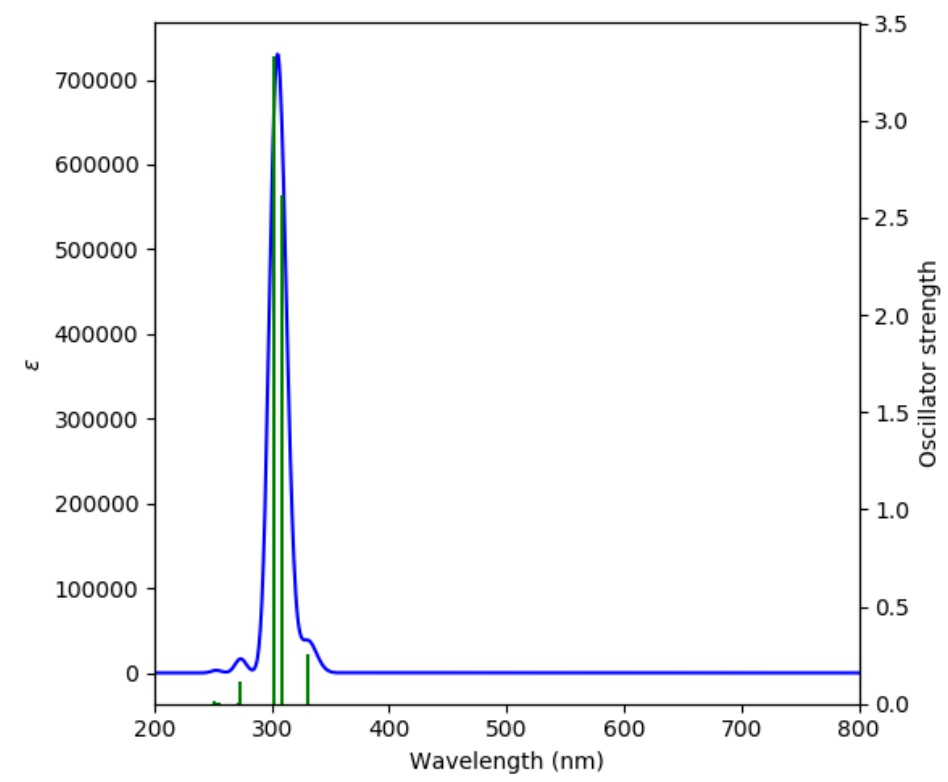


| No. | Energy (cm$^{-1}$) | $\lambda$ (nm) | Osc. Strength | Major Contributions |
|---|---|---|---|---|
| 1 | 30192.5584 | 331.2074 | 0.2568 | H-2->L+2 (14%), H-1->L+1 (19%), HOMO->LUMO (51%) |
| 2 | 32347.6719 | 309.1412 | 2.6151 | H-1->LUMO (35%), HOMO->L+1 (32%) |
| 3 | 33089.7020 | 302.2088 | 3.3334 | H-2->LUMO (36%), HOMO->L+2 (35%) |
| 4 | 36554.6598 | 273.5629 | 0.1112 | H-3->LUMO (22%), H-2->L+2 (23%), H-1->L+1 (19%), HOMO->L+4 (19%) |
| 5 | 36763.5574 | 272.0084 | 0.0035 | H-4->LUMO (18%), H-2->L+1 (18%), H-1->L+2 (23%), HOMO->L+3 (23%) |
| 6 | 39180.8010 | 255.2270 | 0.0056 | HOMO->L+5 (25%) |
| 7 | 39430.8329 | 253.6086 | 0.0039 | H-1->L+7 (15%), HOMO->L+7 (13%) |
| 8 | 39470.3540 | 253.3547 | 0.0008 | H-2->L+5 (12%), HOMO->L+6 (23%) |
| 9 | 39757.4874 | 251.5249 | 0.0111 | HOMO->L+8 (15%) |
| 10 | 39905.0869 | 250.5946 | 0.0001 | HOMO->L+9 (13%) |
| 11 | 39943.8015 | 250.3517 | 0.0005 | HOMO->L+10 (15%) |
| 12 | 40046.2339 | 249.7113 | 0.0002 | HOMO->L+11 (16%) |

**Figure S53.** Calculated absorbance spectrum and electronic transitions for **3** in DCM.

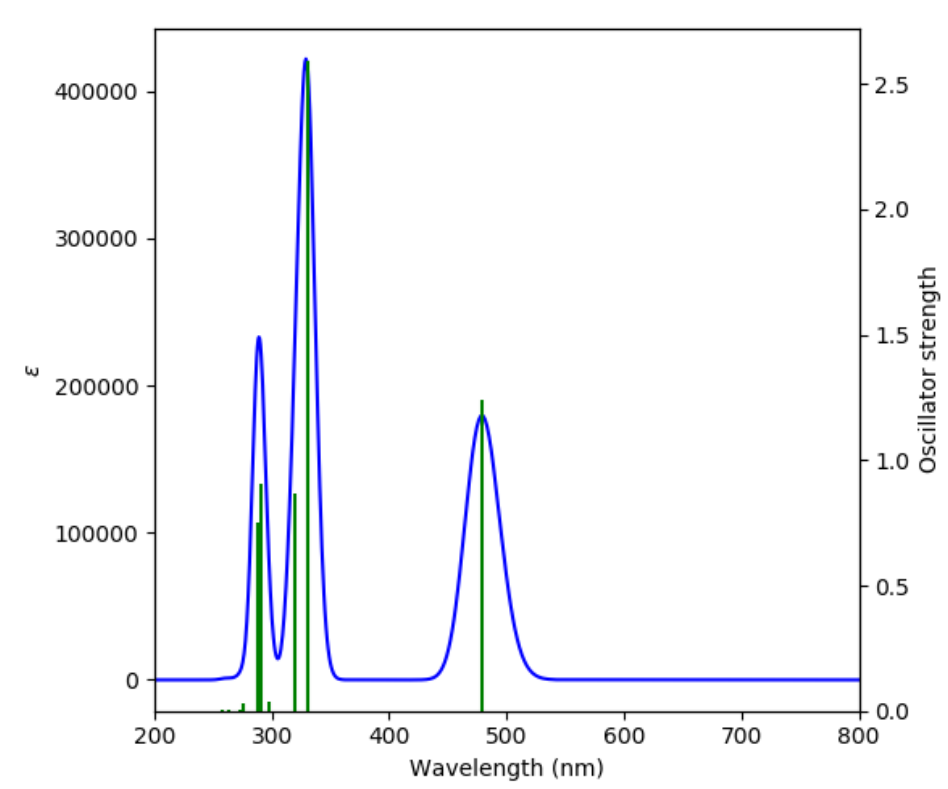


| No. | Energy (cm$^{-1}$) | $\lambda$ (nm) | Osc. Strength | Major Contributions |
|---|---|---|---|---|
| 1 | 20875.2417 | 479.0363 | 1.2404 | HOMO->LUMO (91%) |
| 2 | 30262.7287 | 330.4394 | 2.5940 | H-2->LUMO (30%), HOMO->L+2 (34%) |
| 3 | 31222.5285 | 320.2815 | 0.8665 | H-2->L+2 (18%), H-1->L+1 (43%) |
| 4 | 33581.7002 | 297.7812 | 0.0384 | H-2->LUMO (33%), HOMO->L+2 (29%), HOMO->L+4 (12%) |
| 5 | 34430.1954 | 290.4427 | 0.9066 | H-3->LUMO (13%), H-1->L+1 (24%), HOMO->L+3 (14%) |
| 6 | 34786.6925 | 287.4662 | 0.7521 | H-4->LUMO (13%), H-2->L+1 (22%), H-1->L+2 (17%) |
| 7 | 36211.0676 | 276.1586 | 0.0275 | H-11->LUMO (11%), HOMO->L+5 (15%), HOMO->L+11 (11%) |
| 8 | 36561.1122 | 273.5146 | 0.0040 | H-17->LUMO (20%), HOMO->L+6 (18%), HOMO->L+10 (15%) |
| 9 | 37084.5660 | 269.6539 | 0.0006 | H-3->LUMO (11%), H-1->LUMO (36%), HOMO->L+1 (30%), HOMO->L+3 (12%) |
| 10 | 38046.7855 | 262.8342 | 0.0066 | H-18->LUMO (19%) |
| 11 | 38687.9962 | 258.4781 | 0.0011 | - |
| 12 | 38782.3631 | 257.8491 | 0.0016 | - |

**Figure S54.** Calculated emission spectrum and electronic transitions for **3** in DCM.

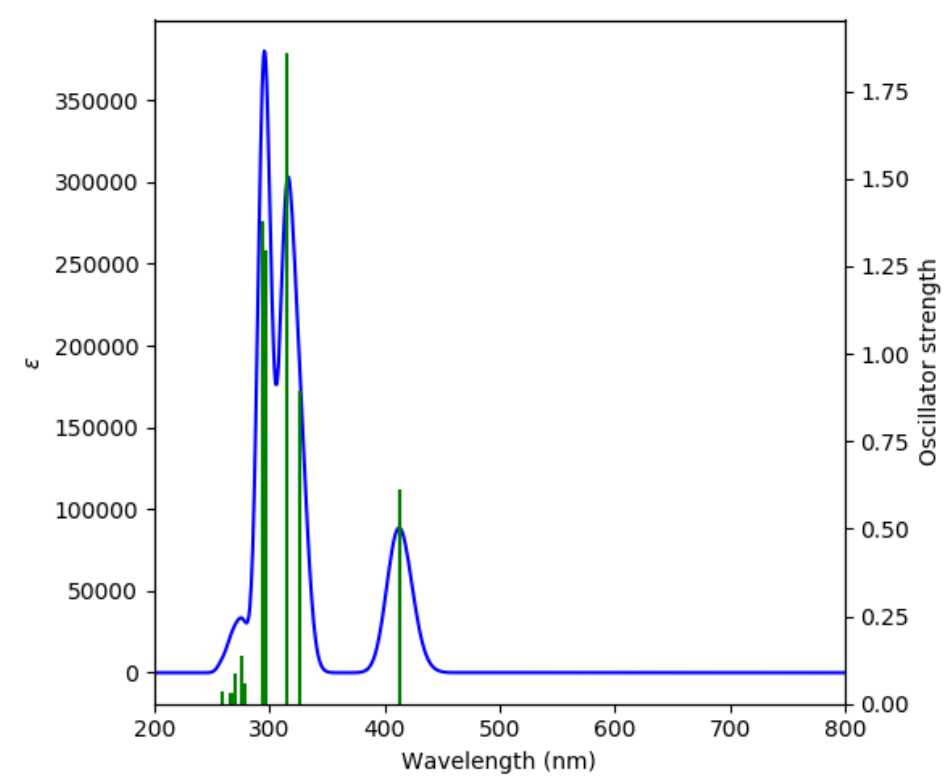


| No. | Energy (cm$^{-1}$) | $\lambda$ (nm) | Osc. Strength | Major Contributions |
|---|---|---|---|---|
| 1 | 24233.7343 | 412.6479 | 0.6117 | H-1->LUMO (45%), HOMO->LUMO (41%) |
| 2 | 30643.4224 | 326.3343 | 0.8932 | H-2->L+2 (12%), H-1->L+1 (15%), HOMO->L+1 (46%) |
| 3 | 31770.1789 | 314.7605 | 1.8601 | H-4->LUMO (13%), H-2->LUMO (32%), HOMO->L+2 (16%) |
| 4 | 33700.2637 | 296.7335 | 1.2933 | H-1->L+1 (15%), HOMO->L+2 (14%), HOMO->L+3 (13%) |
| 5 | 33967.2332 | 294.4013 | 1.3771 | H-4->LUMO (10%), H-2->L+1 (19%), HOMO->L+3 (17%) |
| 6 | 35839.2460 | 279.0237 | 0.0578 | H-1->LUMO (13%), HOMO->LUMO (37%) |
| 7 | 36199.7758 | 276.2448 | 0.1390 | H-18->LUMO (54%) |
| 8 | 37108.7627 | 269.4781 | 0.0859 | H-3->L+1 (12%), H-2->L+3 (11%), H-1->L+2 (13%), HOMO->L+4 (14%) |
| 9 | 37502.3612 | 266.6498 | 0.0326 | H-24->LUMO (19%), H-13->LUMO (15%), H-10->LUMO (18%) |
| 10 | 37616.8920 | 265.8380 | 0.0324 | H-2->L+2 (12%), H-1->L+3 (15%), HOMO->L+5 (17%) |
| 11 | 38559.7541 | 259.3377 | 0.0376 | H-11->LUMO (20%), H-10->LUMO (24%) |
| 12 | 38687.9962 | 258.4781 | 0.0037 | H-7->L+1 (14%), H-1->L+6 (12%), HOMO->L+6 (17%) |

**Figure S55.** Calculated absorbance spectrum and electronic transitions for **4** in DCM.

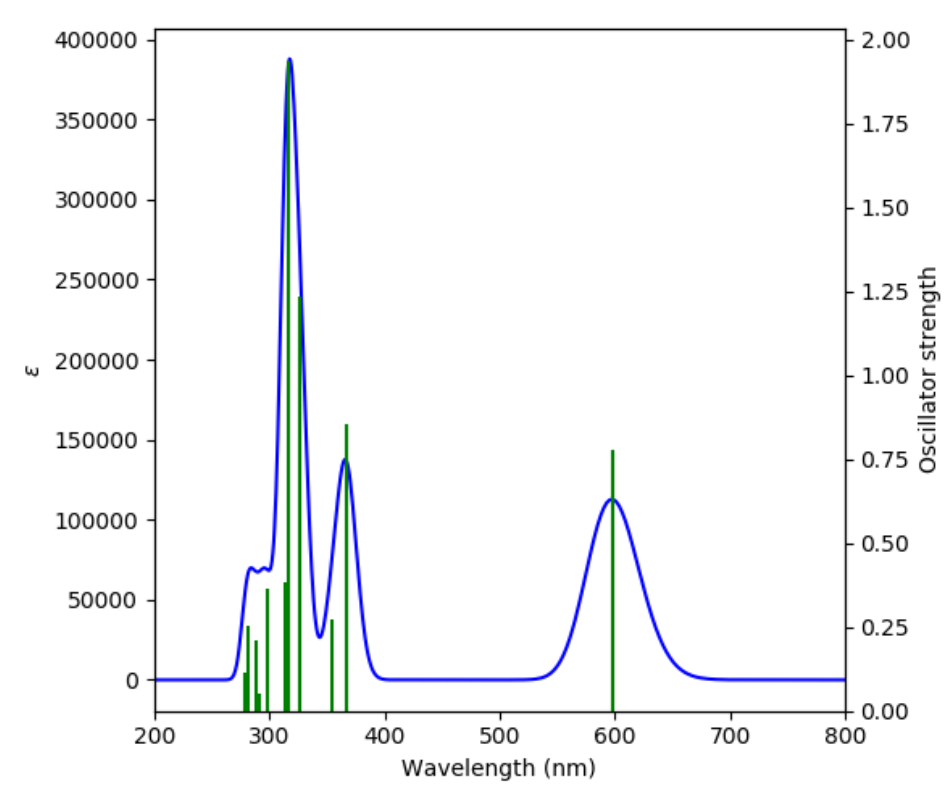


| No. | Energy (cm$^{-1}$) | $\lambda$ (nm) | Osc. Strength | Major Contributions |
|---|---|---|---|---|
| 1 | 16734.3912 | 597.5717 | 0.7783 | HOMO->LUMO (94%) |
| 2 | 27227.6644 | 367.2735 | 0.8527 | H-4->LUMO (15%), H-2->LUMO (32%), HOMO->L+1 (17%), HOMO->L+2 (11%) |
| 3 | 28198.7559 | 354.6255 | 0.2744 | H-2->LUMO (27%), HOMO->L+1 (20%), HOMO->L+3 (17%) |
| 4 | 30626.4847 | 326.5147 | 1.2364 | H-1->LUMO (15%), H-1->L+1 (33%), HOMO->L+2 (11%) |
| 5 | 31643.5499 | 316.0201 | 1.9391 | H-2->L+1 (11%), HOMO->L+2 (18%), HOMO->L+3 (21%) |
| 6 | 31947.6209 | 313.0123 | 0.3848 | H-3->LUMO (14%), H-1->LUMO (46%) |
| 7 | 33578.4739 | 297.8098 | 0.3664 | H-18->LUMO (25%) |
| 8 | 34332.6023 | 291.2683 | 0.0498 | H-3->LUMO (27%), H-1->LUMO (28%) |
| 9 | 34672.1617 | 288.4158 | 0.2119 | - |
| 10 | 35523.8832 | 281.5007 | 0.2528 | H-4->LUMO (10%), H-2->LUMO (12%) |
| 11 | 35652.9319 | 280.4818 | 0.0136 | H-16->LUMO (19%), H-12->LUMO (12%), H-9->LUMO (11%) |
| 12 | 35857.7968 | 278.8793 | 0.1119 | H-4->LUMO (13%), H-3->LUMO (12%), H-2->LUMO (14%) |

**Figure S56.** Calculated emission spectrum and electronic transitions for **4** in DCM.

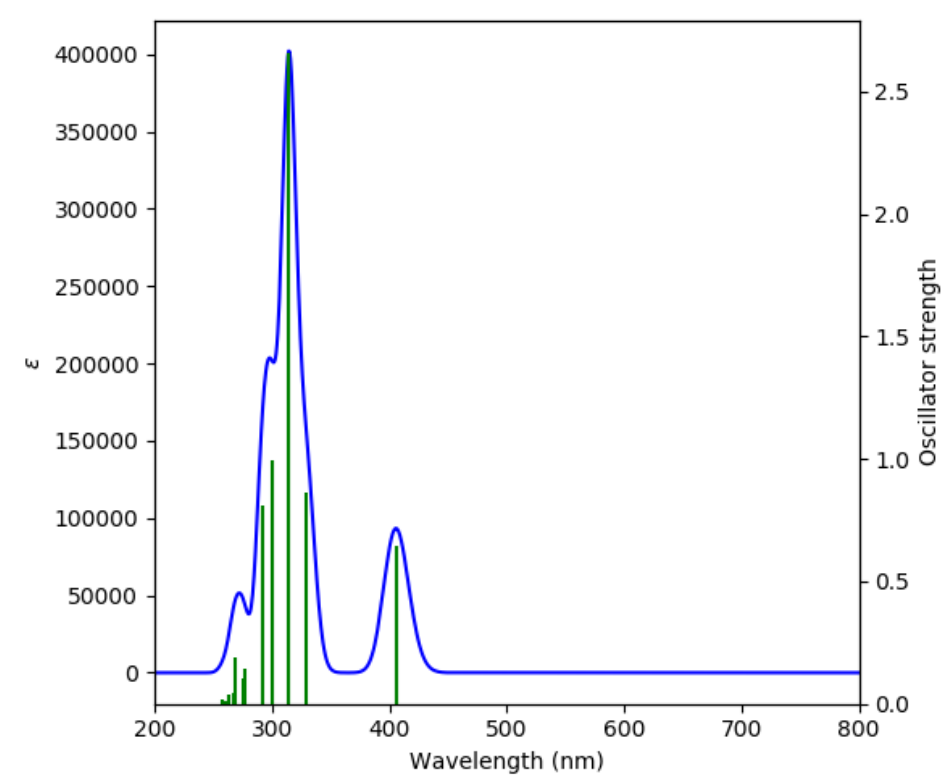


| No. | Energy (cm$^{-1}$) | $\lambda$ (nm) | Osc. Strength | Major Contributions |
|---|---|---|---|---|
| 1 | 24649.1099 | 405.6941 | 0.6447 | H-3->LUMO (10%), H-1->LUMO (42%), HOMO->LUMO (41%) |
| 2 | 30338.5448 | 329.6136 | 0.8609 | H-2->L+2 (13%), H-1->L+1 (16%), H-1->L+3 (11%), HOMO->L+1 (44%) |
| 3 | 31791.9559 | 314.5449 | 2.6596 | H-2->LUMO (31%), H-2->L+1 (15%), HOMO->L+2 (22%) |
| 4 | 33338.9273 | 299.9496 | 0.9951 | H-2->L+2 (13%), H-1->L+1 (21%), HOMO->L+3 (29%) |
| 5 | 34300.3402 | 291.5422 | 0.8081 | H-4->LUMO (13%), H-2->LUMO (26%), H-2->L+1 (16%), HOMO->L+2 (15%) |
| 6 | 36143.3170 | 276.6763 | 0.1422 | H-18->LUMO (43%) |
| 7 | 36323.9852 | 275.3001 | 0.1022 | H-18->LUMO (10%), H-1->LUMO (15%), HOMO->LUMO (40%) |
| 8 | 37192.6443 | 268.8703 | 0.1888 | H-3->L+1 (15%), H-2->L+2 (14%), H-1->L+3 (19%), HOMO->L+5 (19%) |
| 9 | 37331.3717 | 267.8712 | 0.0448 | H-4->L+1 (12%), H-2->L+3 (10%), H-1->L+2 (22%), HOMO->L+4 (17%) |
| 10 | 38025.8151 | 262.9792 | 0.0353 | H-18->LUMO (18%), H-9->LUMO (27%) |
| 11 | 38371.0204 | 260.6133 | 0.0111 | H-13->LUMO (17%) |
| 12 | 38787.2024 | 257.8169 | 0.0164 | H-3->LUMO (20%), H-1->LUMO (21%) |

**Figure S57.** Calculated absorbance spectrum and electronic transitions for **5** in DCM.

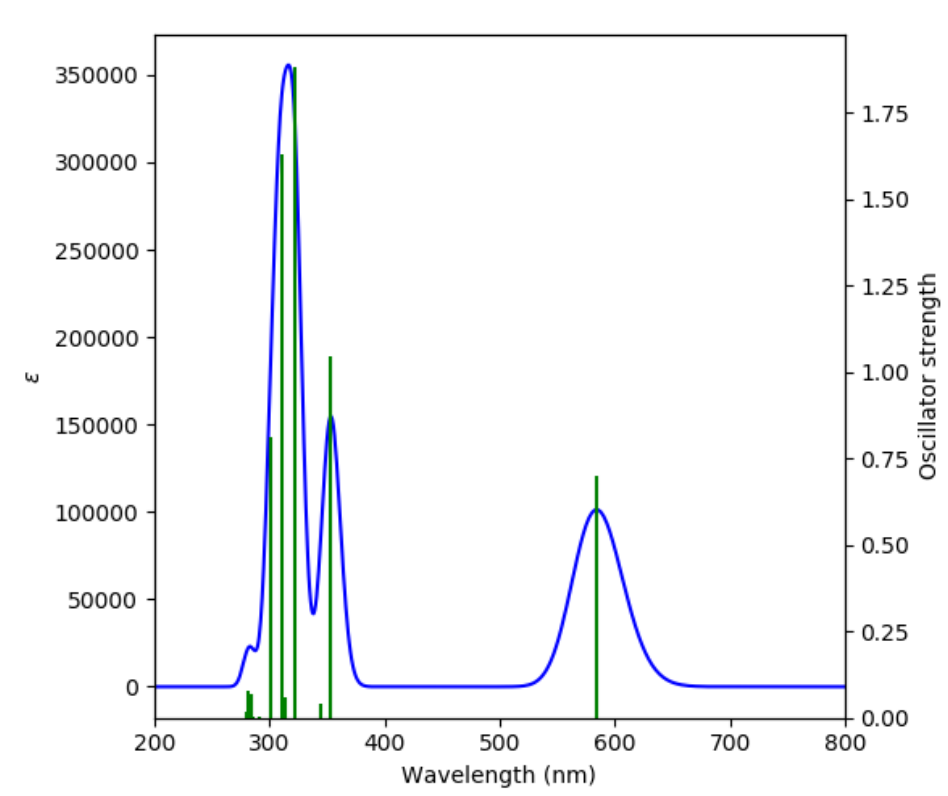


| No. | Energy (cm$^{-1}$) | $\lambda$ (nm) | Osc. Strength | Major Contributions |
|---|---|---|---|---|
| 1 | 17113.4718 | 584.3349 | 0.6994 | HOMO->LUMO (92%) |
| 2 | 28288.2834 | 353.5032 | 1.0473 | H-4->LUMO (22%), H-2->LUMO (60%) |
| 3 | 29078.7068 | 343.8942 | 0.0430 | H-2->L+2 (10%), HOMO->L+1 (23%), HOMO->L+3 (29%) |
| 4 | 31071.7028 | 321.8362 | 1.8838 | H-1->L+1 (44%), HOMO->L+3 (21%) |
| 5 | 31921.8111 | 313.2654 | 0.0577 | H-3->LUMO (17%), H-1->LUMO (53%) |
| 6 | 32212.1707 | 310.4416 | 1.6314 | H-18->LUMO (10%), H-2->L+1 (12%), HOMO->L+2 (34%) |
| 7 | 33165.5181 | 301.5179 | 0.8115 | H-18->LUMO (28%), H-10->LUMO (10%), H-2->L+1 (10%) |
| 8 | 34367.2842 | 290.9744 | 0.0046 | H-5->LUMO (11%), H-3->LUMO (24%), H-1->LUMO (26%) |
| 9 | 34943.1640 | 286.1790 | 0.0057 | H-14->LUMO (10%), H-8->LUMO (10%) |
| 10 | 35145.6092 | 284.5305 | 0.0672 | H-13->LUMO (11%), H-10->LUMO (11%) |
| 11 | 35541.6274 | 281.3602 | 0.0775 | H-18->LUMO (33%) |
| 12 | 35746.4922 | 279.7477 | 0.0156 | H-4->LUMO (13%), H-3->L+1 (12%), H-2->LUMO (11%), H-2->L+2 (15%) |

**Figure S58.** Calculated emission spectrum and electronic transitions for **5** in DCM.

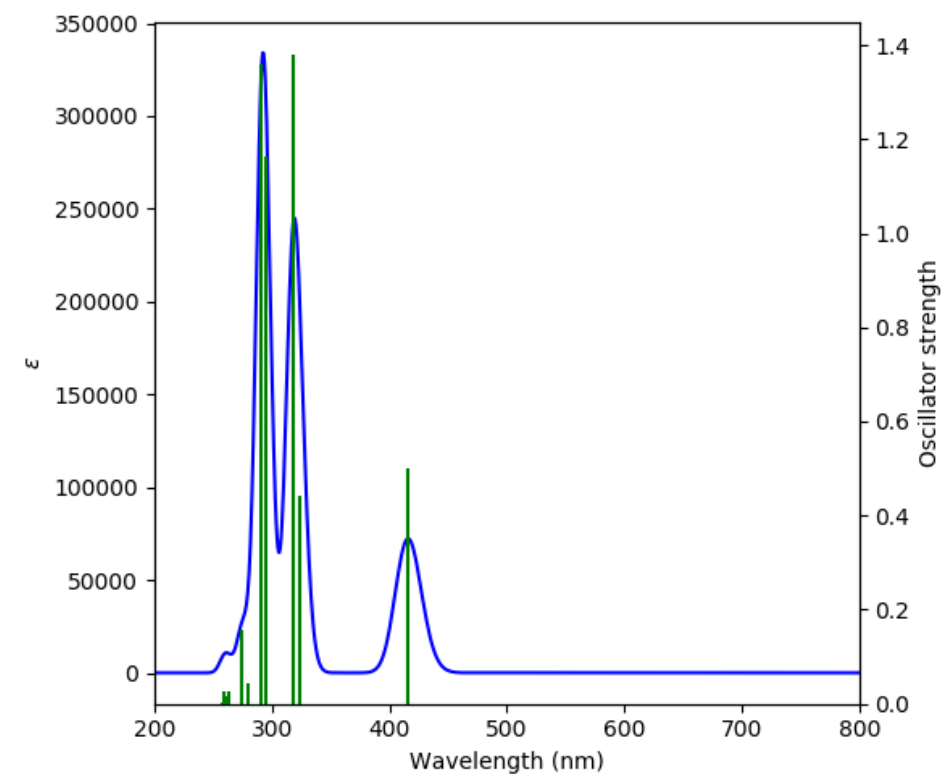


| No. | Energy (cm$^{-1}$) | $\lambda$ (nm) | Osc. Strength | Major Contributions |
|---|---|---|---|---|
| 1 | 24040.9678 | 415.9566 | 0.4994 | H-1->LUMO (25%), HOMO->LUMO (62%) |
| 2 | 30858.7724 | 324.0569 | 0.4416 | H-2->L+2 (11%), HOMO->L+1 (47%) |
| 3 | 31451.5899 | 317.9489 | 1.3805 | H-2->LUMO (42%), HOMO->L+2 (15%) |
| 4 | 33888.1908 | 295.0880 | 1.1629 | H-2->LUMO (10%), H-1->L+1 (27%), HOMO->L+2 (19%), HOMO->L+3 (15%) |
| 5 | 34433.4216 | 290.4155 | 1.3601 | H-2->L+1 (25%), HOMO->L+2 (11%), HOMO->L+3 (22%) |
| 6 | 35686.0007 | 280.2219 | 0.0433 | H-1->LUMO (37%), HOMO->LUMO (32%) |
| 7 | 36384.4768 | 274.8424 | 0.1576 | H-16->LUMO (20%), H-15->LUMO (37%), H-14->LUMO (12%) |
| 8 | 37912.0909 | 263.7680 | 0.0257 | H-3->L+1 (10%), H-1->L+2 (18%), HOMO->L+4 (15%) |
| 9 | 38219.3881 | 261.6473 | 0.0142 | H-7->LUMO (15%), HOMO->L+5 (11%) |
| 10 | 38498.4560 | 259.7506 | 0.0151 | - |
| 11 | 38649.2816 | 258.7370 | 0.0261 | H-7->LUMO (23%) |
| 12 | 38900.9266 | 257.0632 | 0.0018 | HOMO->L+6 (22%) |

**Figure S59.** Calculated absorbance spectrum and electronic transitions for **6** in DCM.

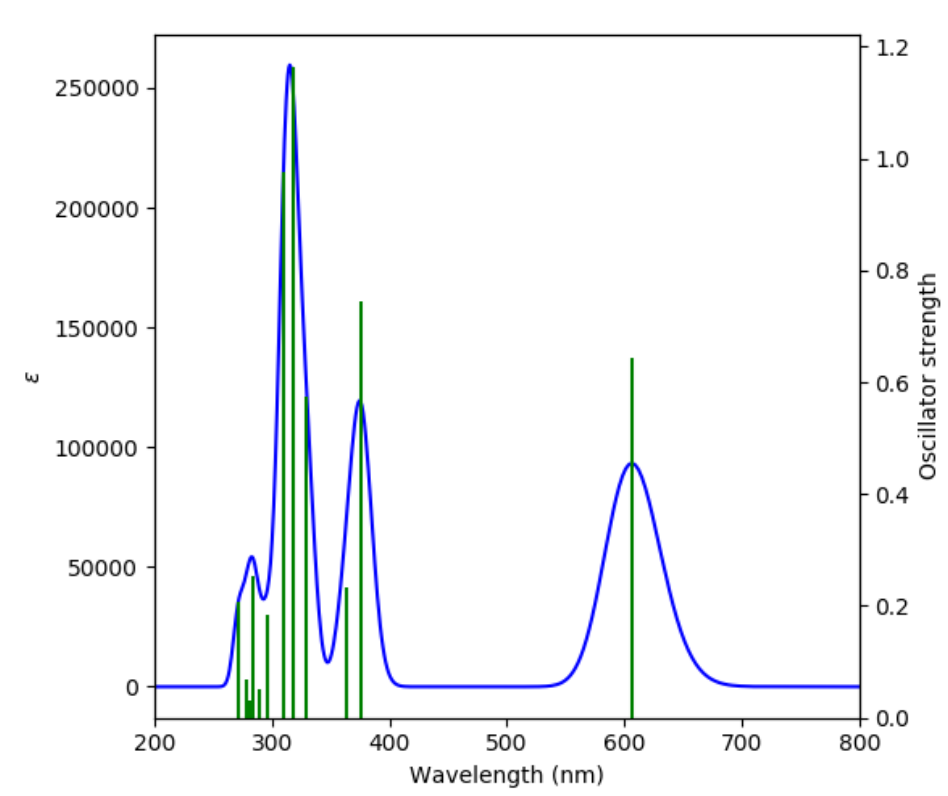


| No. | Energy (cm$^{-1}$) | $\lambda$ (nm) | Osc. Strength | Major Contributions |
|---|---|---|---|---|
| 1 | 16489.1987 | 606.4576 | 0.6440 | HOMO->LUMO (93%) |
| 2 | 26581.6143 | 376.1998 | 0.7441 | H-2->LUMO (35%), HOMO->L+1 (32%) |
| 3 | 27561.5779 | 362.8239 | 0.2318 | H-2->LUMO (36%), HOMO->L+1 (27%) |
| 4 | 30322.4137 | 329.7890 | 0.5751 | H-1->LUMO (36%), H-1->L+1 (13%), HOMO->L+2 (18%) |
| 5 | 31439.4916 | 318.0713 | 1.1644 | H-2->L+1 (10%), H-1->LUMO (15%), HOMO->L+2 (29%), HOMO->L+3 (22%) |
| 6 | 32260.5640 | 309.9759 | 0.9767 | H-1->LUMO (17%), H-1->L+1 (22%), HOMO->L+3 (10%) |
| 7 | 33698.6505 | 296.7477 | 0.1839 | H-16->LUMO (24%), H-14->LUMO (12%), H-8->LUMO (12%) |
| 8 | 34651.9979 | 288.5836 | 0.0511 | H-8->LUMO (12%), H-3->LUMO (19%), H-1->LUMO (13%) |
| 9 | 35257.7203 | 283.6258 | 0.2548 | H-4->LUMO (19%), H-1->L+2 (14%) |
| 10 | 35647.2860 | 280.5262 | 0.0298 | H-16->LUMO (14%), H-12->LUMO (16%), H-6->LUMO (11%) |
| 11 | 35914.2556 | 278.4409 | 0.0684 | H-16->LUMO (12%), H-3->LUMO (19%) |
| 12 | 36812.7572 | 271.6449 | 0.2064 | H-4->LUMO (14%), H-2->LUMO (11%) |

**Figure S60.** Calculated emission spectrum and electronic transitions for **6** in DCM.

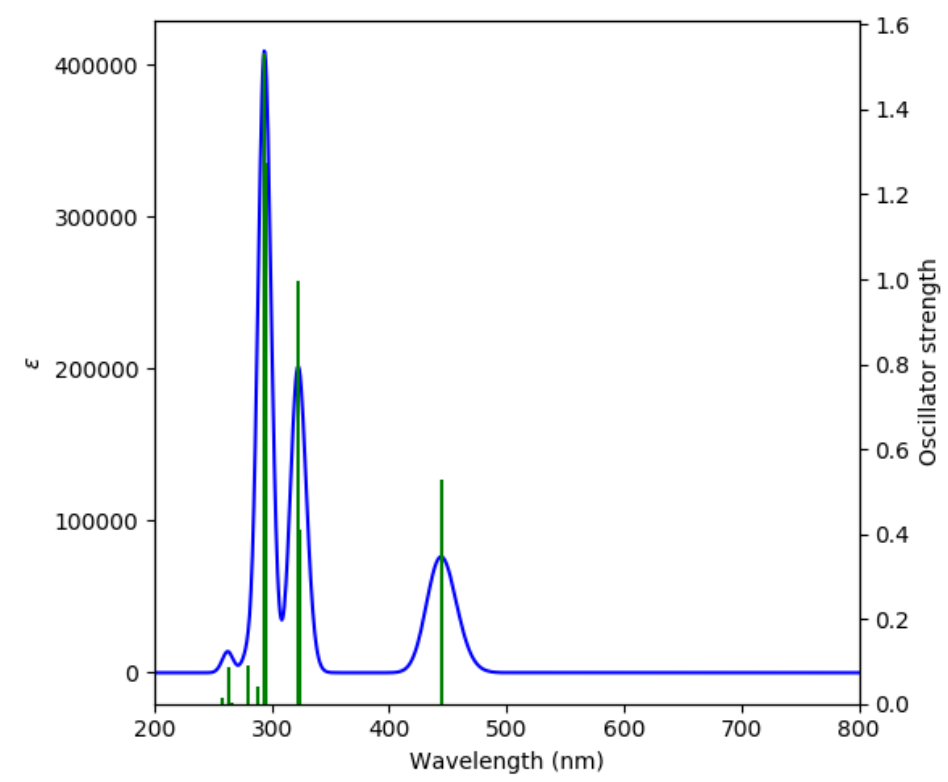


| No. | Energy (cm$^{-1}$) | $\lambda$ (nm) | Osc. Strength | Major Contributions |
|---|---|---|---|---|
| 1 | 22510.1275 | 444.2444 | 0.5274 | H-1->LUMO (22%), HOMO->LUMO (71%) |
| 2 | 30868.4511 | 323.9553 | 0.4095 | H-2->L+2 (16%), H-1->L+1 (19%), HOMO->L+1 (39%) |
| 3 | 31062.0241 | 321.9365 | 0.9957 | H-4->LUMO (23%), H-2->LUMO (51%) |
| 4 | 33980.9446 | 294.2825 | 1.2760 | H-1->L+1 (30%), HOMO->L+3 (40%) |
| 5 | 34074.5049 | 293.4745 | 1.5343 | H-4->LUMO (10%), H-2->L+1 (29%), HOMO->L+2 (35%) |
| 6 | 34773.7876 | 287.5729 | 0.0395 | H-1->LUMO (50%), HOMO->LUMO (23%) |
| 7 | 35675.5155 | 280.3042 | 0.0919 | H-15->LUMO (53%), H-7->LUMO (11%) |
| 8 | 37258.7818 | 268.3931 | 0.0005 | H-10->LUMO (14%), H-6->LUMO (36%) |
| 9 | 37649.1541 | 265.6102 | 0.0013 | H-7->LUMO (16%), H-1->L+2 (17%), HOMO->L+4 (14%) |
| 10 | 38004.8446 | 263.1243 | 0.0021 | H-3->L+1 (14%), H-2->L+2 (17%), H-1->L+3 (16%), HOMO->L+5 (21%) |
| 11 | 38035.4937 | 262.9123 | 0.0855 | H-15->LUMO (14%), H-7->LUMO (31%) |
| 12 | 38786.3959 | 257.8223 | 0.0146 | H-4->LUMO (29%), H-2->LUMO (21%) |

**Figure S61.** Calculated absorbance spectrum and electronic transitions for **7** in DCM.

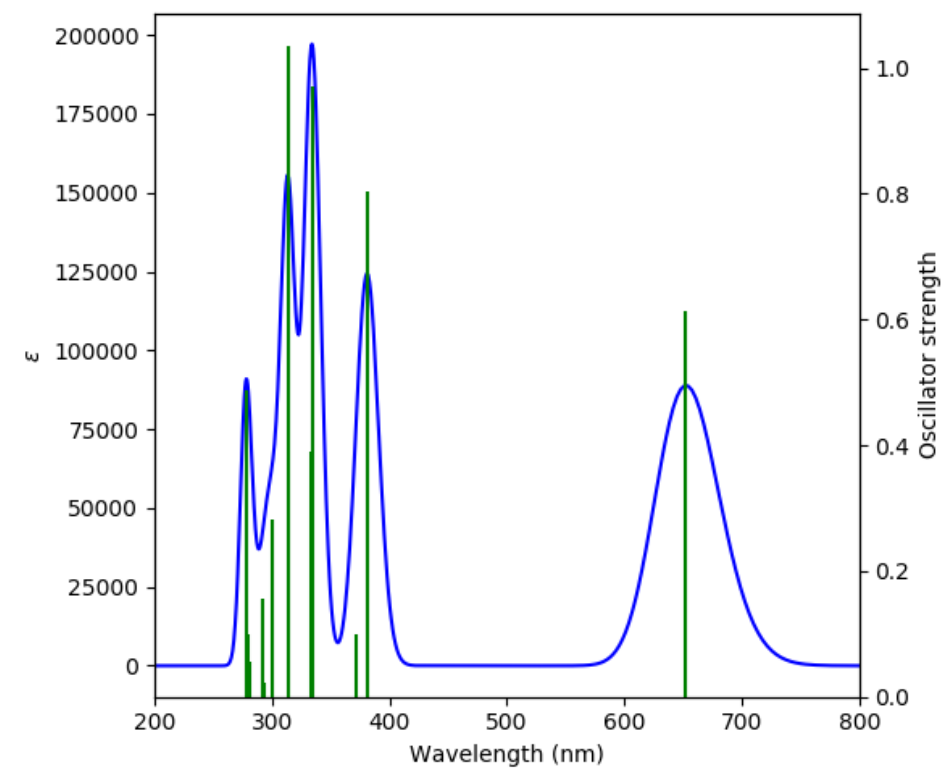


| No. | Energy (cm$^{-1}$) | $\lambda$ (nm) | Osc. Strength | Major Contributions |
|---|---|---|---|---|
| 1 | 15322.9210 | 652.6170 | 0.6134 | HOMO->LUMO (95%) |
| 2 | 26195.2747 | 381.7482 | 0.8037 | H-4->LUMO (17%), H-2->LUMO (72%) |
| 3 | 26909.8819 | 371.6106 | 0.0991 | HOMO->L+1 (47%), HOMO->L+3 (27%) |
| 4 | 29871.5498 | 334.7666 | 0.9711 | HOMO->L+2 (67%) |
| 5 | 30051.4114 | 332.7630 | 0.3909 | H-3->LUMO (10%), H-1->LUMO (58%) |
| 6 | 31887.9359 | 313.5982 | 1.0359 | H-1->LUMO (11%), H-1->L+1 (35%), H-1->L+3 (13%), HOMO->L+3 (11%) |
| 7 | 33284.0816 | 300.4439 | 0.2812 | H-16->LUMO (30%), H-9->LUMO (13%) |
| 8 | 34112.4130 | 293.1484 | 0.0229 | H-11->LUMO (32%), H-3->LUMO (28%), H-1->LUMO (15%) |
| 9 | 34176.9373 | 292.5949 | 0.1561 | H-9->LUMO (31%), H-4->LUMO (23%) |
| 10 | 35497.2669 | 281.7118 | 0.0555 | H-15->LUMO (13%), H-3->LUMO (19%), HOMO->L+1 (10%), HOMO->L+5 (13%) |
| 11 | 35777.9479 | 279.5017 | 0.0992 | H-16->LUMO (33%), H-9->LUMO (13%), H-4->LUMO (14%) |
| 12 | 36016.6880 | 277.6490 | 0.4878 | H-4->LUMO (18%), H-2->LUMO (11%), H-2->L+1 (11%), H-1->L+2 (11%), HOMO->L+4 (14%) |

**Figure S62.** Calculated emission spectrum and electronic transitions for **7** in DCM.

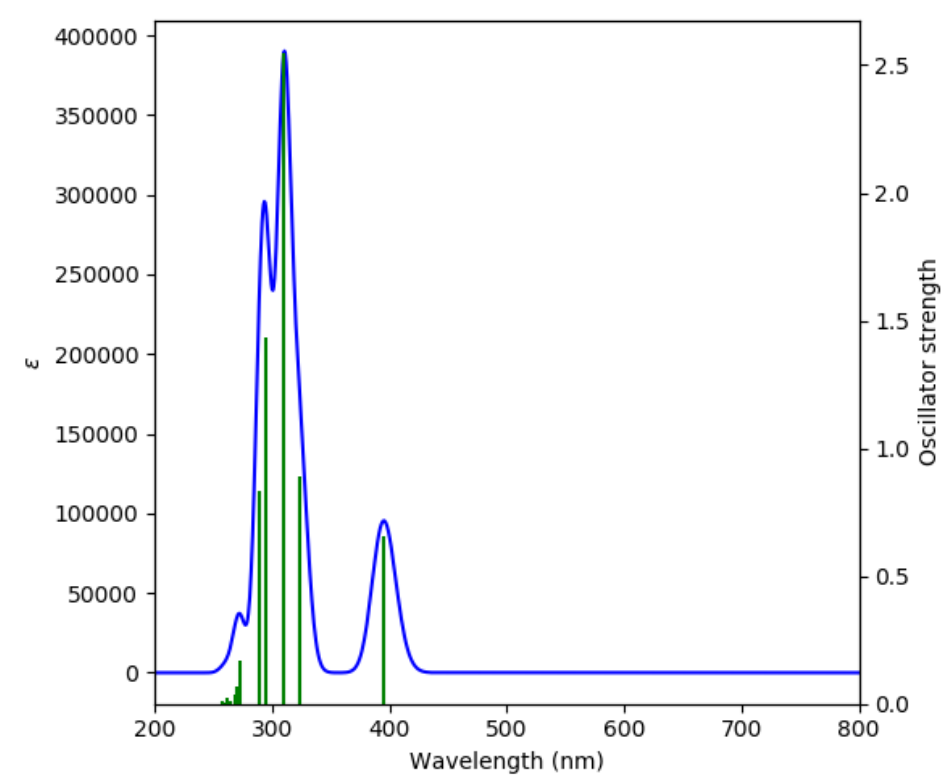


| No. | Energy (cm⁻¹) | λ (nm) | Osc. Strength | Major Contributions |
|---|---|---|---|---|
| 1 | 25293.5468 | 395.3577 | 0.6587 | H-1->LUMO (37%), HOMO->LUMO (44%) |
| 2 | 30861.9986 | 324.0230 | 0.8897 | H-2->L+2 (11%), H-1->L+1 (18%), HOMO->L+1 (43%) |
| 3 | 32210.5576 | 310.4572 | 2.5493 | H-2->LUMO (25%), H-2->L+1 (12%), HOMO->L+2 (24%) |
| 4 | 33883.3515 | 295.1301 | 1.4363 | H-1->L+1 (19%), HOMO->L+3 (30%) |
| 5 | 34546.3393 | 289.4662 | 0.8311 | H-2->LUMO (17%), H-2->L+1 (19%), HOMO->L+2 (11%) |
| 6 | 36568.3712 | 273.4603 | 0.1660 | H-18->LUMO (49%) |
| 7 | 36942.6125 | 270.6901 | 0.0701 | H-1->LUMO (17%), HOMO->LUMO (40%) |
| 8 | 37287.8178 | 268.1841 | 0.0357 | H-18->LUMO (18%), H-3->L+1 (11%), HOMO->L+4 (11%) |
| 9 | 37890.3139 | 263.9196 | 0.0091 | H-4->L+1 (16%), H-1->L+3 (10%), HOMO->L+5 (16%) |
| 10 | 38210.5160 | 261.7080 | 0.0214 | H-16->LUMO (10%) |
| 11 | 38625.0850 | 258.8991 | 0.0025 | H-7->L+1 (15%), H-1->L+6 (24%), HOMO->L+6 (20%) |
| 12 | 38886.4086 | 257.1592 | 0.0097 | H-24->LUMO (13%), H-15->LUMO (14%) |

**Figure S63.** Calculated absorbance spectrum and electronic transitions for **1** in DMSO.

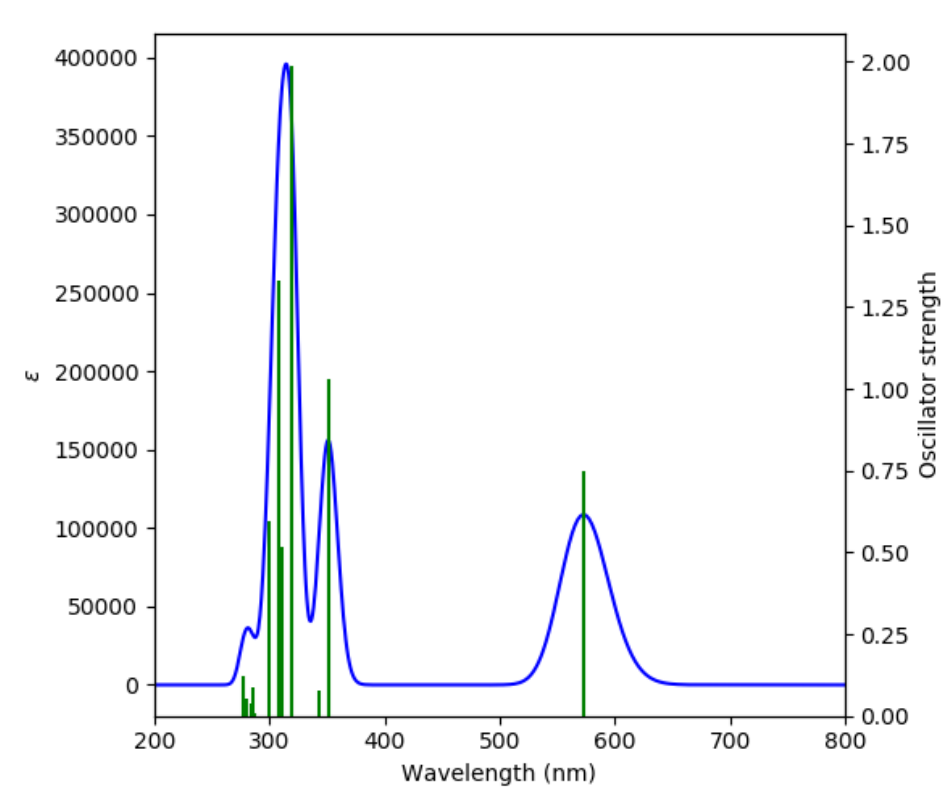


| No. | Energy (cm⁻¹) | Wavelength (nm) | Osc. Strength | Major Contributions |
|---|---|---|---|---|
| 1 | 17453.0312 | 572.9663 | 0.7495 | HOMO->LUMO (92%) |
| 2 | 28461.6926 | 351.3494 | 1.0305 | H-4->LUMO (20%), H-2->LUMO (56%) |
| 3 | 29136.7787 | 343.2088 | 0.0800 | HOMO->L+1 (22%), HOMO->L+3 (34%) |
| 4 | 31312.8625 | 319.3575 | 1.9893 | H-1->L+1 (45%), HOMO->L+3 (20%) |
| 5 | 32204.9117 | 310.5116 | 0.5156 | H-3->LUMO (13%), H-1->LUMO (43%) |
| 6 | 32443.6519 | 308.2267 | 1.3290 | H-2->L+1 (10%), H-1->LUMO (11%), HOMO->L+2 (29%) |
| 7 | 33376.0288 | 299.6162 | 0.5980 | H-18->LUMO (30%), H-15->LUMO (10%) |
| 8 | 34775.4007 | 287.5595 | 0.0072 | H-5->LUMO (10%), H-3->LUMO (24%), H-1->LUMO (21%) |
| 9 | 35083.5045 | 285.0342 | 0.0892 | H-17->LUMO (18%) |
| 10 | 35264.9792 | 283.5674 | 0.0396 | H-3->LUMO (13%) |
| 11 | 35644.0598 | 280.5516 | 0.0531 | H-18->LUMO (34%) |
| 12 | 36056.2091 | 277.3447 | 0.1222 | H-4->LUMO (18%), H-2->LUMO (21%) |

**Figure S64.** Calculated emission spectrum and electronic transitions for **1** in DMSO.

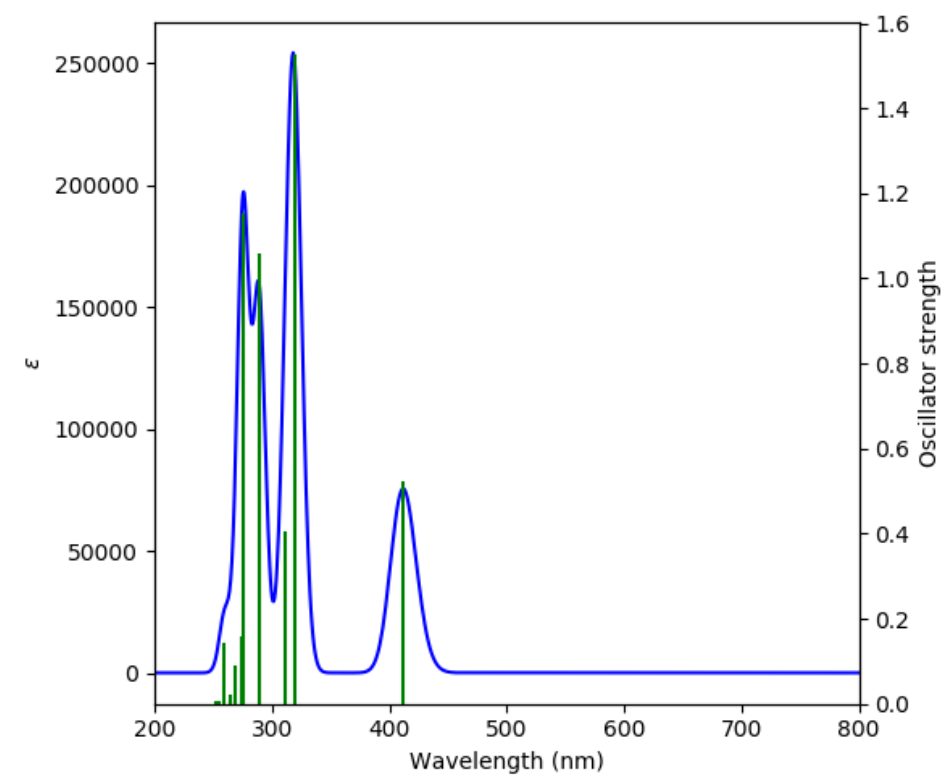


| No. | Energy (cm$^{-1}$) | $\lambda$ (nm) | Osc. Strength | Major Contributions |
|---|---|---|---|---|
| 1 | 24283.7407 | 411.7981 | 0.5215 | H-2->LUMO (12%), HOMO->LUMO (79%) |
| 2 | 31330.6067 | 319.1767 | 1.5270 | H-1->LUMO (35%), H-1->L+1 (15%), HOMO->L+1 (29%) |
| 3 | 32068.6040 | 311.8314 | 0.4061 | H-1->LUMO (14%), H-1->L+2 (17%), HOMO->L+1 (12%), HOMO->L+2 (28%) |
| 4 | 34594.7325 | 289.0613 | 1.0602 | H-1->LUMO (22%), H-1->L+1 (10%), HOMO->L+1 (12%), HOMO->L+2 (16%) |
| 5 | 36242.5232 | 275.9189 | 1.1553 | H-2->L+2 (16%), HOMO->L+3 (16%) |
| 6 | 36536.9156 | 273.6957 | 0.1574 | H-15->LUMO (43%) |
| 7 | 37265.2342 | 268.3466 | 0.0919 | H-4->LUMO (20%), H-2->LUMO (32%), HOMO->LUMO (14%) |
| 8 | 37720.9375 | 265.1047 | 0.0081 | H-6->LUMO (10%), H-6->L+1 (13%), H-1->L+5 (12%), HOMO->L+5 (25%) |
| 9 | 37855.6321 | 264.1614 | 0.0232 | H-15->LUMO (13%), H-7->LUMO (13%), HOMO->L+6 (13%) |
| 10 | 38529.9116 | 259.5386 | 0.1420 | H-2->L+1 (13%), H-2->L+2 (14%), HOMO->L+4 (11%) |
| 11 | 39163.8634 | 255.3374 | 0.0070 | H-20->LUMO (15%), H-13->LUMO (12%) |
| 12 | 39551.8160 | 252.8328 | 0.0070 | H-20->LUMO (31%) |

**Figure S65.** Calculated absorbance spectrum and electronic transitions for **2** in DMSO.

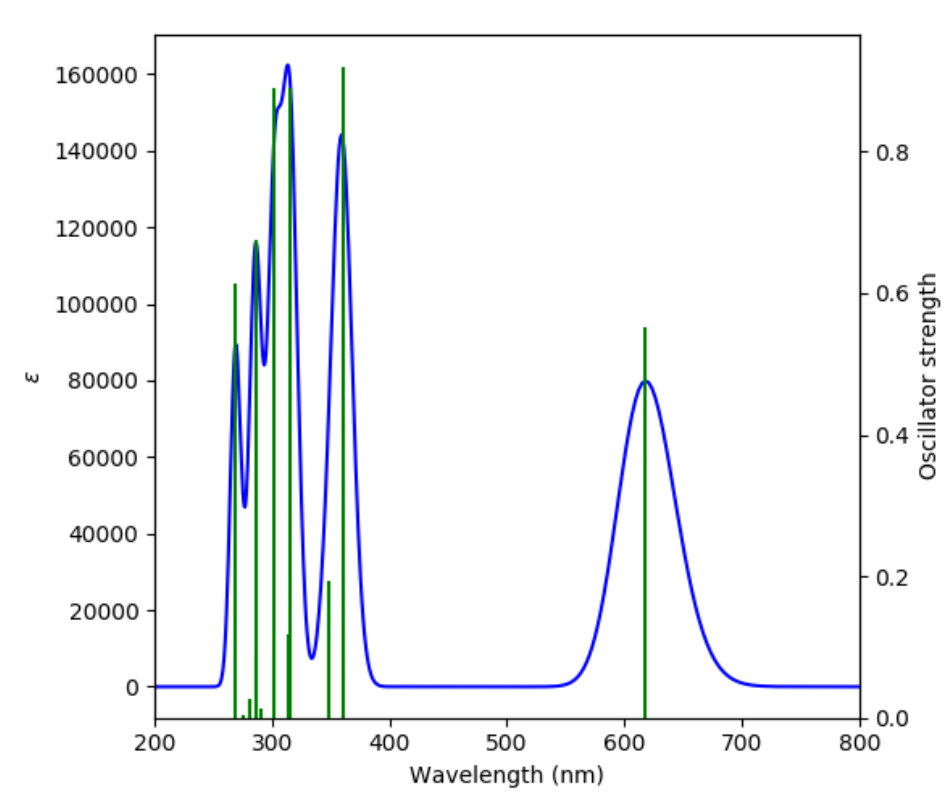


| No. | Energy (cm$^{-1}$) | $\lambda$ (nm) | Osc. Strength | Major Contributions |
|---|---|---|---|---|
| 1 | 16175.4490 | 618.2208 | 0.5511 | HOMO->LUMO (95%) |
| 2 | 27758.3772 | 360.2516 | 0.9204 | H-3->LUMO (19%), H-1->LUMO (69%) |
| 3 | 28673.8165 | 348.7502 | 0.1927 | HOMO->L+1 (61%), HOMO->L+3 (13%) |
| 4 | 31698.3956 | 315.4733 | 0.8891 | H-15->LUMO (17%), HOMO->L+2 (34%), HOMO->L+4 (11%) |
| 5 | 31784.6969 | 314.6168 | 0.1185 | H-4->LUMO (15%), H-2->LUMO (57%) |
| 6 | 33084.0561 | 302.2603 | 0.8901 | H-15->LUMO (16%), H-12->LUMO (10%), HOMO->L+2 (22%) |
| 7 | 34420.5168 | 290.5244 | 0.0124 | H-13->LUMO (29%), H-11->LUMO (24%), H-9->LUMO (11%) |
| 8 | 34896.3839 | 286.5626 | 0.0873 | H-15->LUMO (39%), H-8->LUMO (10%) |
| 9 | 34949.6165 | 286.1261 | 0.6745 | H-2->L+1 (18%), H-1->L+2 (30%), HOMO->L+3 (17%) |
| 10 | 35592.4403 | 280.9585 | 0.0268 | H-3->LUMO (39%), H-1->LUMO (18%) |
| 11 | 36354.6343 | 275.0680 | 0.0029 | H-20->LUMO (13%), H-19->LUMO (61%) |
| 12 | 37157.9625 | 269.1213 | 0.6128 | H-2->L+2 (28%), H-1->L+1 (11%), HOMO->L+4 (14%) |

**Figure S66.** Calculated emission spectrum and electronic transitions for **2** in DMSO.

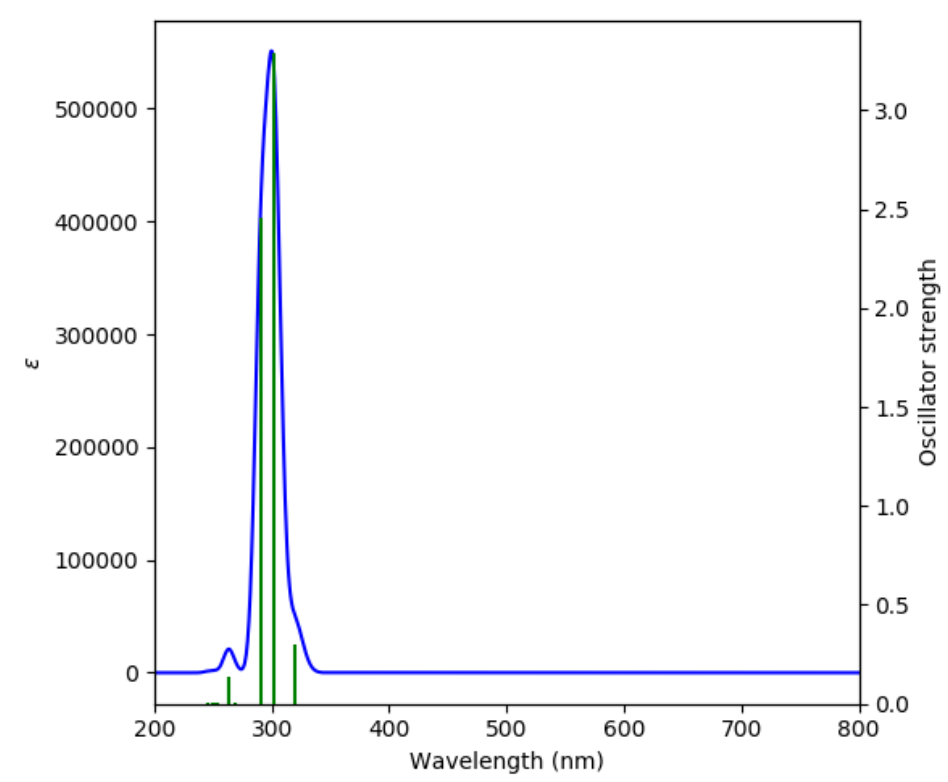


| No. | Energy (cm$^{-1}$) | $\lambda$ (nm) | Osc. Strength | Major Contributions |
|---|---|---|---|---|
| 1 | 31253.9841 | 319.9592 | 0.3019 | H-2->L+2 (10%), H-1->L+1 (22%), HOMO->LUMO (53%) |
| 2 | 33123.5772 | 301.8997 | 3.2917 | H-1->LUMO (32%), HOMO->L+1 (30%) |
| 3 | 34351.1531 | 291.1110 | 2.4546 | H-2->LUMO (21%), H-1->L+1 (23%), HOMO->L+2 (20%) |
| 4 | 37283.7850 | 268.2131 | 0.0077 | H-3->LUMO (18%), H-2->L+1 (17%), H-1->L+2 (21%), HOMO->L+3 (16%) |
| 5 | 37979.0349 | 263.3031 | 0.1401 | H-3->L+1 (21%), H-1->L+3 (18%) |
| 6 | 39342.1119 | 254.1805 | 0.0002 | H-7->LUMO (11%), HOMO->L+6 (16%) |
| 7 | 39509.8752 | 253.1012 | 0.0039 | HOMO->L+7 (14%) |
| 8 | 39747.8088 | 251.5861 | 0.0020 | HOMO->L+8 (13%) |
| 9 | 39928.4770 | 250.4478 | 0.0014 | HOMO->L+11 (10%) |
| 10 | 40126.8893 | 249.2094 | 0.0034 | HOMO->L+12 (12%) |
| 11 | 40424.5079 | 247.3746 | 0.0007 | - |
| 12 | 40685.8316 | 245.7858 | 0.0058 | - |

**Figure S67.** Calculated absorbance spectrum and electronic transitions for **3** in DMSO.

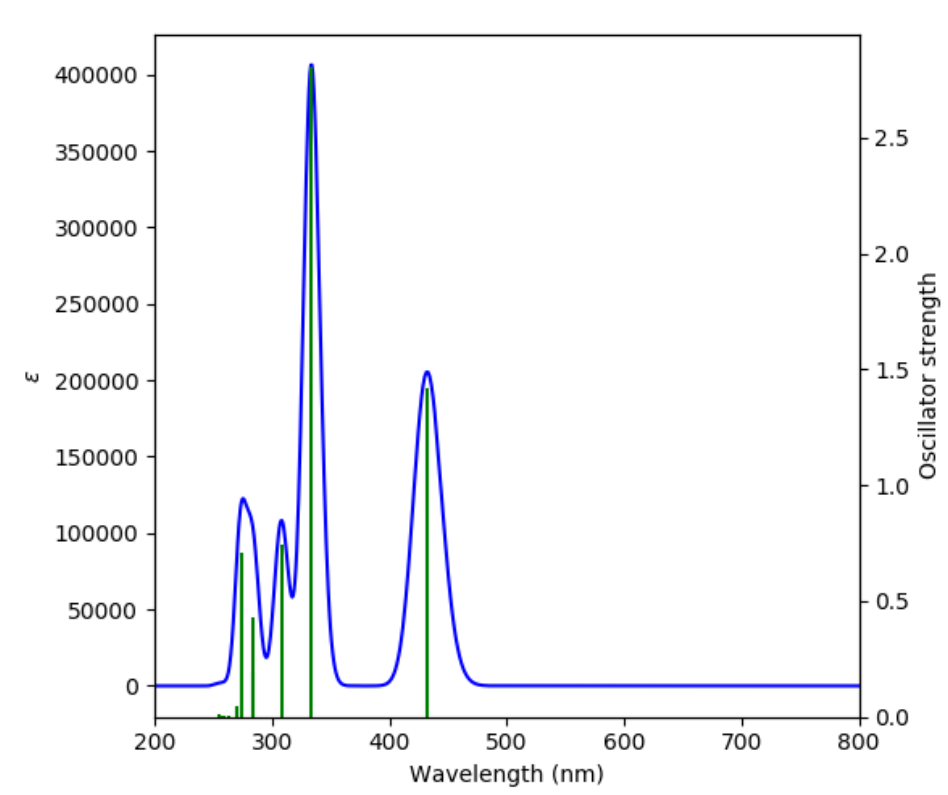


| No. | Energy (cm$^{-1}$) | $\lambda$ (nm) | Osc. Strength | Major Contributions |
|---|---|---|---|---|
| 1 | 23131.9810 | 432.3019 | 1.4192 | HOMO->LUMO (88%) |
| 2 | 29971.5625 | 333.6496 | 2.8083 | H-1->LUMO (32%), HOMO->L+1 (30%) |
| 3 | 32454.1371 | 308.1271 | 0.7461 | H-2->LUMO (14%), H-2->L+2 (14%), H-1->L+1 (36%), HOMO->L+2 (12%) |
| 4 | 35020.5933 | 285.5462 | 0.0010 | H-1->LUMO (16%), HOMO->L+1 (17%), HOMO->L+6 (22%) |
| 5 | 35167.3862 | 284.3543 | 0.1920 | H-1->LUMO (13%), H-1->L+2 (11%), HOMO->L+1 (12%), HOMO->L+6 (16%) |
| 6 | 35253.6875 | 283.6582 | 0.4298 | H-2->L+1 (22%), H-1->L+2 (16%), HOMO->L+3 (12%) |
| 7 | 36517.5583 | 273.8408 | 0.7067 | H-3->L+1 (20%), H-1->L+3 (19%) |
| 8 | 36958.7436 | 270.5719 | 0.0477 | HOMO->L+7 (13%) |
| 9 | 38044.3658 | 262.8510 | 0.0048 | H-19->LUMO (12%) |
| 10 | 38619.4391 | 258.9369 | 0.0022 | H-2->LUMO (27%), HOMO->L+2 (28%) |
| 11 | 38855.7596 | 257.3621 | 0.0025 | H-17->LUMO (11%) |
| 12 | 39176.7682 | 255.2533 | 0.0087 | H-19->LUMO (27%), HOMO->L+18 (11%) |

**Figure S68.** Calculated emission spectrum and electronic transitions for **3** in DMSO.

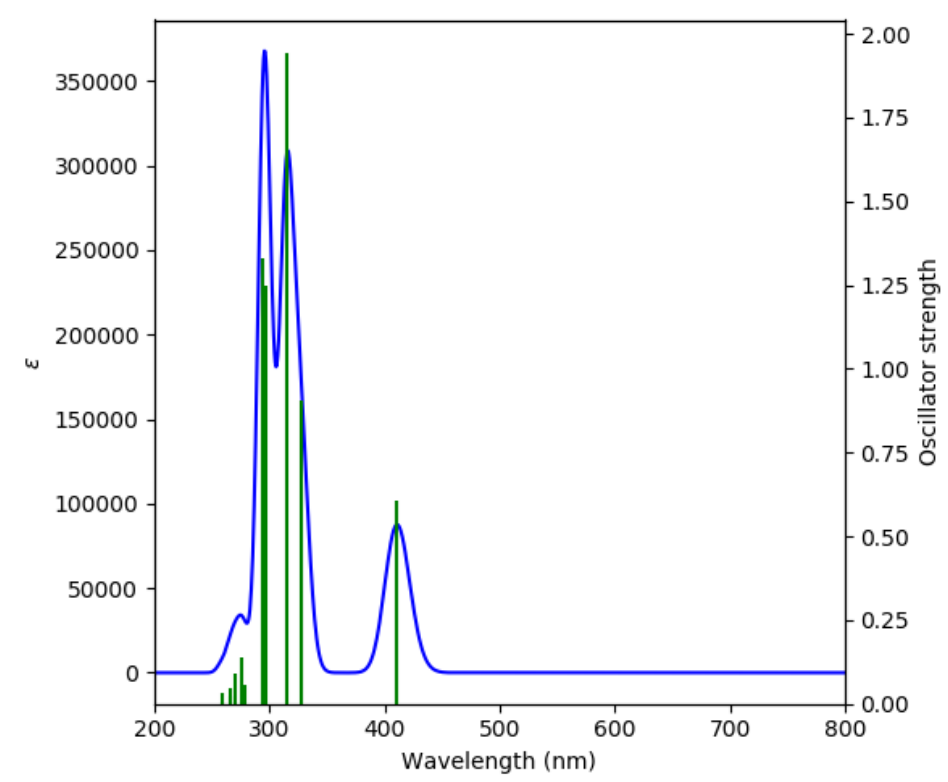


| No. | Energy (cm$^{-1}$) | $\lambda$ (nm) | Osc. Strength | Major Contributions |
|---|---|---|---|---|
| 1 | 24339.3930 | 410.8565 | 0.6054 | H-1->LUMO (46%), HOMO->LUMO (39%) |
| 2 | 30563.5735 | 327.1868 | 0.9056 | H-2->L+2 (12%), H-1->L+1 (15%), HOMO->L+1 (47%) |
| 3 | 31770.9855 | 314.7525 | 1.9442 | H-4->LUMO (12%), H-2->LUMO (31%), HOMO->L+2 (17%) |
| 4 | 33688.1653 | 296.8401 | 1.2494 | H-1->L+1 (15%), HOMO->L+2 (14%), HOMO->L+3 (13%) |
| 5 | 33942.2300 | 294.6182 | 1.3295 | H-4->LUMO (10%), H-2->L+1 (19%), HOMO->L+3 (17%) |
| 6 | 35862.6361 | 278.8417 | 0.0568 | H-1->LUMO (12%), HOMO->LUMO (36%) |
| 7 | 36215.9069 | 276.1217 | 0.1387 | H-18->LUMO (53%), HOMO->LUMO (10%) |
| 8 | 37088.5988 | 269.6246 | 0.0879 | H-3->L+1 (12%), H-2->L+3 (11%), H-1->L+2 (13%), HOMO->L+4 (14%) |
| 9 | 37548.3348 | 266.3233 | 0.0472 | H-24->LUMO (18%), H-13->LUMO (14%), H-10->LUMO (11%) |
| 10 | 37621.7313 | 265.8038 | 0.0216 | H-2->L+2 (10%), H-1->L+3 (13%), HOMO->L+5 (14%) |
| 11 | 38632.3440 | 258.8504 | 0.0327 | H-11->LUMO (23%), H-10->LUMO (11%) |
| 12 | 38644.4423 | 258.7694 | 0.0055 | H-7->L+1 (13%), H-1->L+6 (13%), HOMO->L+6 (19%) |

**Figure S69.** Calculated absorbance spectrum and electronic transitions for **4** in DMSO.

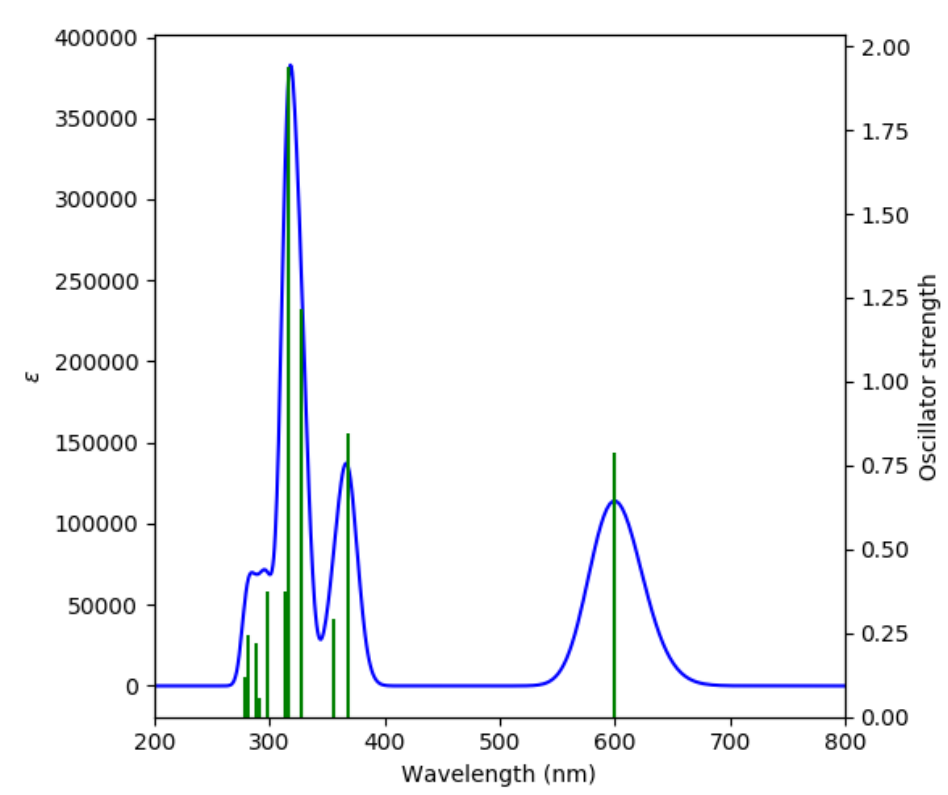


| No. | Energy (cm$^{-1}$) | $\lambda$ (nm) | Osc. Strength | Major Contributions |
|---|---|---|---|---|
| 1 | 16669.0603 | 599.9138 | 0.7872 | HOMO->LUMO (94%) |
| 2 | 27160.7203 | 368.1787 | 0.8474 | H-4->LUMO (14%), H-2->LUMO (30%), HOMO->L+1 (17%), HOMO->L+2 (12%) |
| 3 | 28149.5561 | 355.2453 | 0.2908 | H-4->LUMO (11%), H-2->LUMO (29%), HOMO->L+1 (19%), HOMO->L+3 (17%) |
| 4 | 30540.1834 | 327.4374 | 1.2171 | H-1->LUMO (15%), H-1->L+1 (34%), HOMO->L+2 (11%) |
| 5 | 31567.7337 | 316.7791 | 1.9394 | H-2->L+1 (11%), HOMO->L+2 (19%), HOMO->L+3 (21%) |
| 6 | 31896.8080 | 313.5109 | 0.3747 | H-3->LUMO (14%), H-1->LUMO (47%) |
| 7 | 33530.0807 | 298.2396 | 0.3730 | H-18->LUMO (24%) |
| 8 | 34300.3402 | 291.5422 | 0.0544 | H-3->LUMO (27%), H-1->LUMO (28%) |
| 9 | 34616.5095 | 288.8795 | 0.2206 | - |
| 10 | 35493.2342 | 281.7438 | 0.2461 | H-2->LUMO (12%) |
| 11 | 35661.8040 | 280.4120 | 0.0136 | H-16->LUMO (19%), H-12->LUMO (13%) |
| 12 | 35821.5018 | 279.1619 | 0.1170 | H-4->LUMO (16%), H-2->LUMO (14%) |

**Figure S70.** Calculated emission spectrum and electronic transitions for **4** in DMSO.

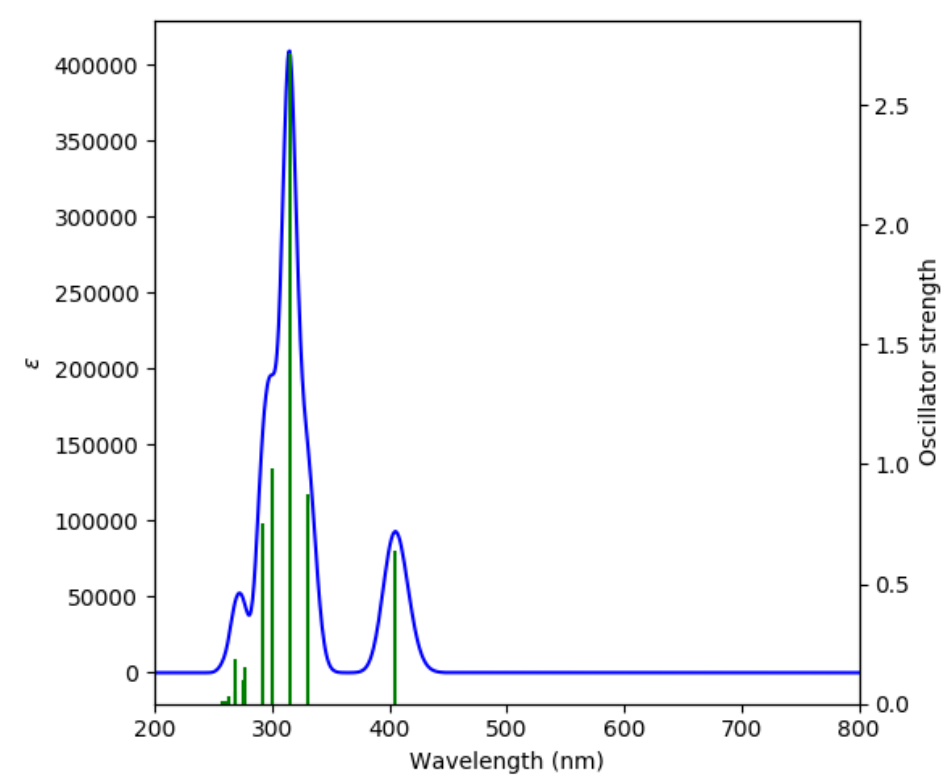


| No. | Energy (cm-1) | λ (nm) | Osc. Strength | Major Contributions |
|---|---|---|---|---|
| 1 | 24679.7589 | 405.1903 | 0.6413 | H-3->LUMO (10%), H-1->LUMO (42%), HOMO->LUMO (41%) |
| 2 | 30254.6631 | 330.5275 | 0.8720 | H-2->L+2 (13%), H-1->L+1 (16%), H-1->L+3 (10%), HOMO->L+1 (44%) |
| 3 | 31766.1461 | 314.8005 | 2.7183 | H-2->LUMO (30%), H-2->L+1 (15%), HOMO->L+2 (23%) |
| 4 | 33305.8585 | 300.2474 | 0.9845 | H-2->L+2 (13%), H-1->L+1 (21%), HOMO->L+3 (29%) |
| 5 | 34301.1467 | 291.5354 | 0.7501 | H-4->LUMO (14%), H-2->LUMO (27%), H-2->L+1 (16%), HOMO->L+2 (15%) |
| 6 | 36132.0253 | 276.7627 | 0.1508 | H-18->LUMO (45%) |
| 7 | 36335.2770 | 275.2146 | 0.0966 | H-3->LUMO (10%), H-1->LUMO (16%), HOMO->LUMO (42%) |
| 8 | 37162.8018 | 269.0862 | 0.1847 | H-3->L+1 (14%), H-2->L+2 (14%), H-1->L+3 (19%), HOMO->L+5 (19%) |
| 9 | 37310.4013 | 268.0217 | 0.0502 | H-4->L+1 (11%), H-2->L+3 (10%), H-1->L+2 (22%), HOMO->L+4 (17%) |
| 10 | 38061.3035 | 262.7340 | 0.0339 | H-18->LUMO (18%), H-9->LUMO (29%) |
| 11 | 38400.0563 | 260.4162 | 0.0098 | H-13->LUMO (17%) |
| 12 | 38824.3039 | 257.5706 | 0.0149 | H-3->LUMO (20%), H-1->LUMO (21%) |

**Figure S71.** Calculated absorbance spectrum and electronic transitions for **5** in DMSO.

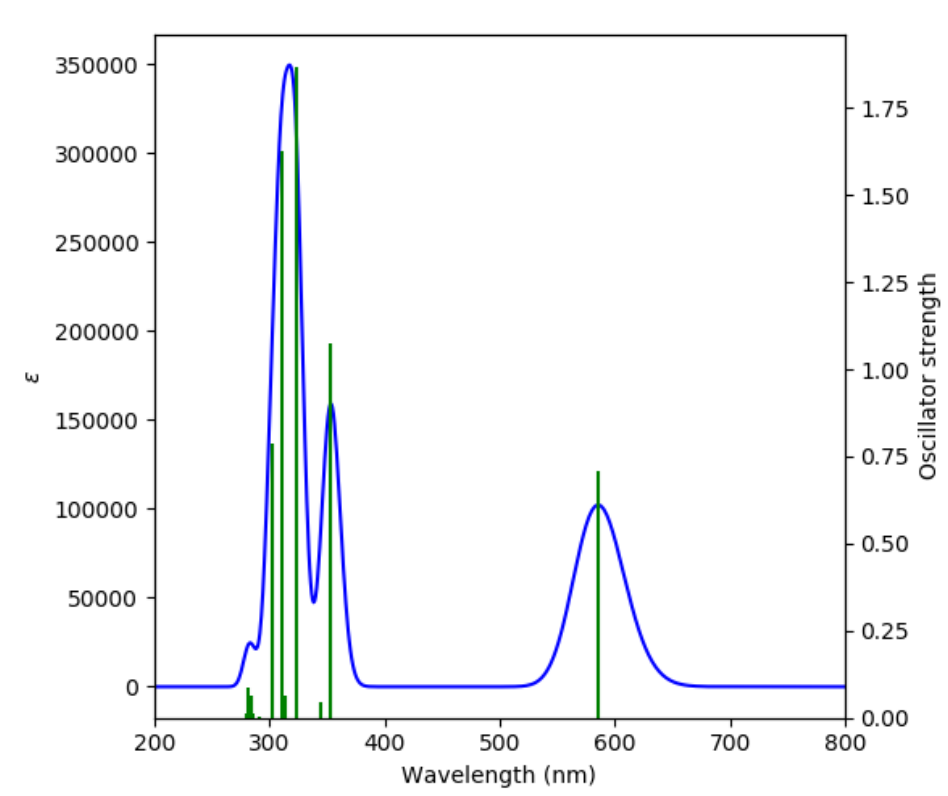


| No. | Energy (cm-1) | λ (nm) | Osc. Strength | Major Contributions |
|---|---|---|---|---|
| 1 | 17074.7572 | 585.6598 | 0.7060 | HOMO->LUMO (93%) |
| 2 | 28288.2834 | 353.5032 | 1.0740 | H-4->LUMO (21%), H-2->LUMO (61%) |
| 3 | 28997.2448 | 344.8603 | 0.0446 | H-2->L+2 (10%), HOMO->L+1 (22%), HOMO->L+3 (30%) |
| 4 | 30990.2408 | 322.6822 | 1.8691 | H-1->L+1 (44%), HOMO->L+3 (21%) |
| 5 | 31902.4538 | 313.4555 | 0.0640 | H-3->LUMO (18%), H-1->LUMO (53%) |
| 6 | 32145.2267 | 311.0881 | 1.6268 | H-18->LUMO (10%), H-2->L+1 (12%), HOMO->L+2 (34%) |
| 7 | 33134.0624 | 301.8042 | 0.7862 | H-18->LUMO (28%), H-10->LUMO (10%), H-2->L+1 (10%) |
| 8 | 34376.1563 | 290.8993 | 0.0042 | H-5->LUMO (11%), H-3->LUMO (25%), H-1->LUMO (27%) |
| 9 | 34954.4558 | 286.0865 | 0.0123 | H-16->LUMO (11%) |
| 10 | 35138.3502 | 284.5893 | 0.0639 | H-13->LUMO (13%) |
| 11 | 35507.7521 | 281.6286 | 0.0876 | H-18->LUMO (36%) |
| 12 | 35725.5218 | 279.9119 | 0.0135 | H-4->LUMO (12%), H-3->L+1 (13%), H-2->LUMO (10%), H-2->L+2 (16%) |

**Figure S72.** Calculated emission spectrum and electronic transitions for **5** in DMSO.

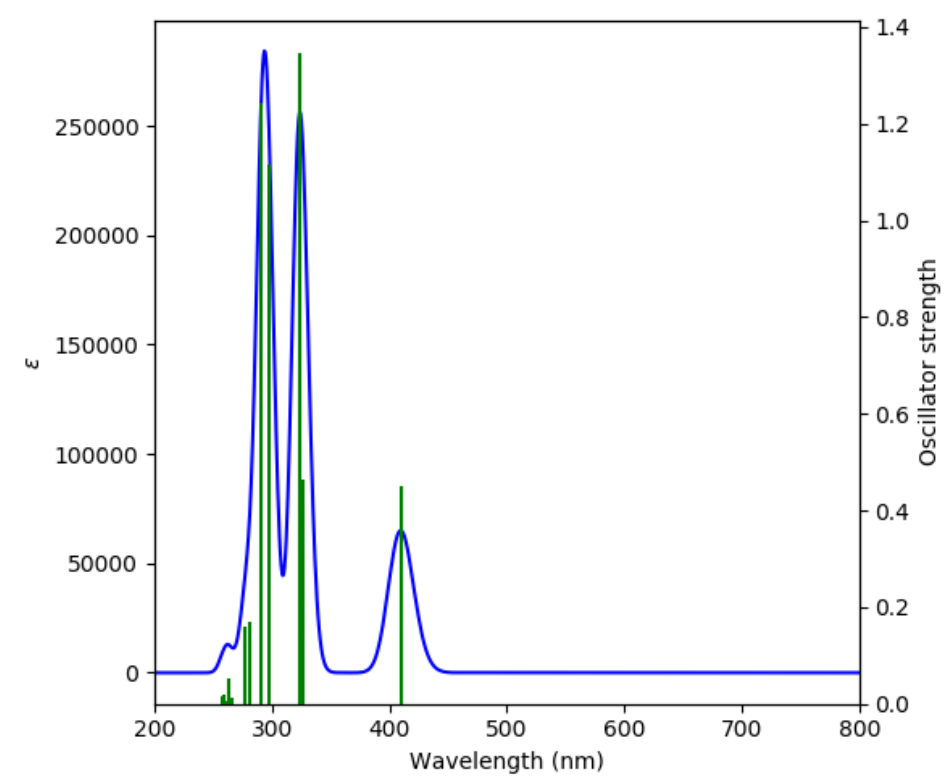


| No. | Energy (cm$^{-1}$) | $\lambda$ (nm) | Osc. Strength | Major Contributions |
|---|---|---|---|---|
| 1 | 24410.3697 | 409.6619 | 0.4491 | H-2->LUMO (18%), H-1->LUMO (14%), HOMO->LUMO (55%) |
| 2 | 30632.9372 | 326.4460 | 0.4651 | H-1->LUMO (13%), H-1->L+2 (12%), HOMO->L+1 (43%) |
| 3 | 30957.9786 | 323.0185 | 1.3475 | H-4->LUMO (12%), H-2->LUMO (22%), H-1->LUMO (23%), HOMO->L+2 (14%) |
| 4 | 33598.6378 | 297.6311 | 1.1151 | H-1->L+1 (26%), HOMO->L+2 (28%) |
| 5 | 34386.6415 | 290.8106 | 1.2434 | H-2->L+1 (28%), HOMO->L+3 (28%) |
| 6 | 35502.1063 | 281.6734 | 0.1685 | H-3->LUMO (10%), H-2->LUMO (12%), H-1->LUMO (16%), HOMO->LUMO (34%) |
| 7 | 36155.4153 | 276.5837 | 0.1600 | H-15->LUMO (63%) |
| 8 | 37636.2493 | 265.7012 | 0.0126 | H-20->LUMO (13%) |
| 9 | 37988.7136 | 263.2360 | 0.0527 | H-2->L+2 (21%), HOMO->L+4 (16%) |
| 10 | 38430.7054 | 260.2085 | 0.0054 | - |
| 11 | 38617.0195 | 258.9531 | 0.0196 | HOMO->L+5 (16%) |
| 12 | 38827.5302 | 257.5492 | 0.0143 | H-9->LUMO (13%) |

**Figure S73.** Calculated absorbance spectrum and electronic transitions for **6** in DMSO.

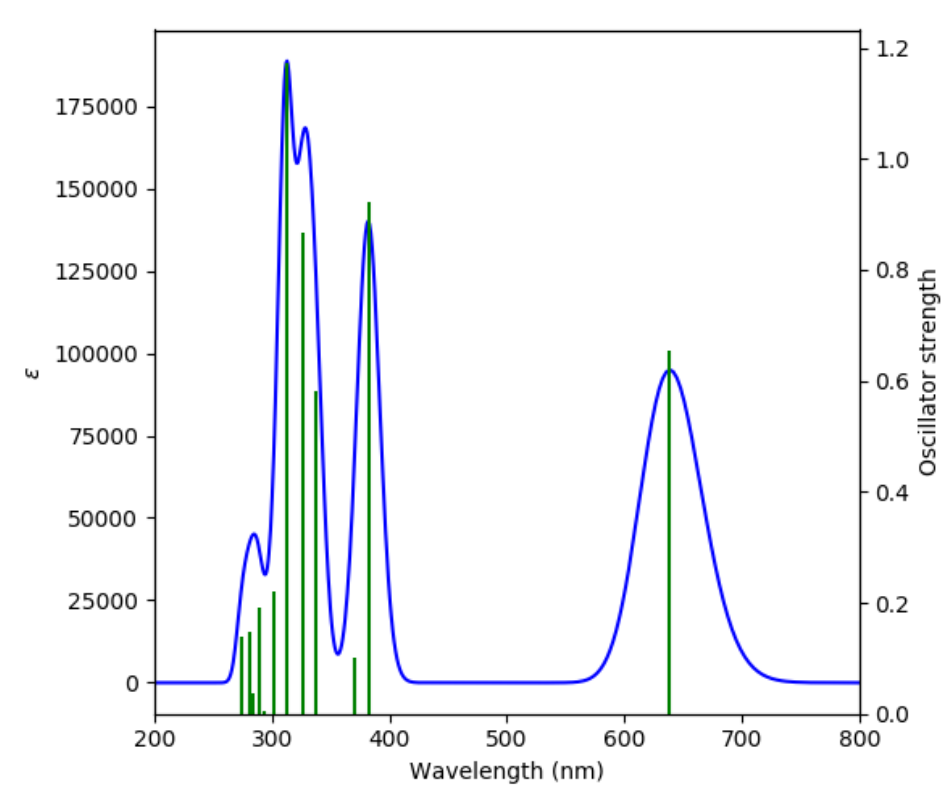


| No. | Energy (cm$^{-1}$) | $\lambda$ (nm) | Osc. Strength | Major Contributions |
|---|---|---|---|---|
| 1 | 15652.8018 | 638.8632 | 0.6550 | HOMO->LUMO (94%) |
| 2 | 26138.0093 | 382.5846 | 0.9220 | H-4->LUMO (11%), H-2->LUMO (57%), HOMO->L+1 (12%) |
| 3 | 26980.8587 | 370.6331 | 0.1024 | H-2->LUMO (12%), HOMO->L+1 (42%), HOMO->L+3 (15%) |
| 4 | 29653.7801 | 337.2251 | 0.5805 | H-1->LUMO (28%), HOMO->L+2 (28%) |
| 5 | 30603.9012 | 326.7557 | 0.8675 | H-1->LUMO (27%), HOMO->L+2 (31%), HOMO->L+3 (13%) |
| 6 | 32033.9222 | 312.1690 | 1.1737 | H-1->L+1 (26%), H-1->L+3 (10%), HOMO->L+3 (14%) |
| 7 | 33097.7675 | 302.1351 | 0.2203 | H-16->LUMO (20%), H-9->LUMO (11%) |
| 8 | 34097.8950 | 293.2732 | 0.0052 | H-8->LUMO (14%), H-3->LUMO (23%), H-1->LUMO (11%) |
| 9 | 34636.6733 | 288.7113 | 0.1924 | H-4->LUMO (21%), H-1->L+2 (11%) |
| 10 | 35156.9010 | 284.4391 | 0.0368 | H-16->LUMO (11%), H-14->LUMO (16%), H-3->LUMO (10%) |
| 11 | 35587.6010 | 280.9967 | 0.1468 | H-16->LUMO (25%), H-3->LUMO (18%) |
| 12 | 36457.8732 | 274.2891 | 0.1377 | H-2->L+2 (12%), H-1->L+3 (13%), HOMO->L+5 (14%) |

**Figure S74.** Calculated emission spectrum and electronic transitions for **6** in DMSO.

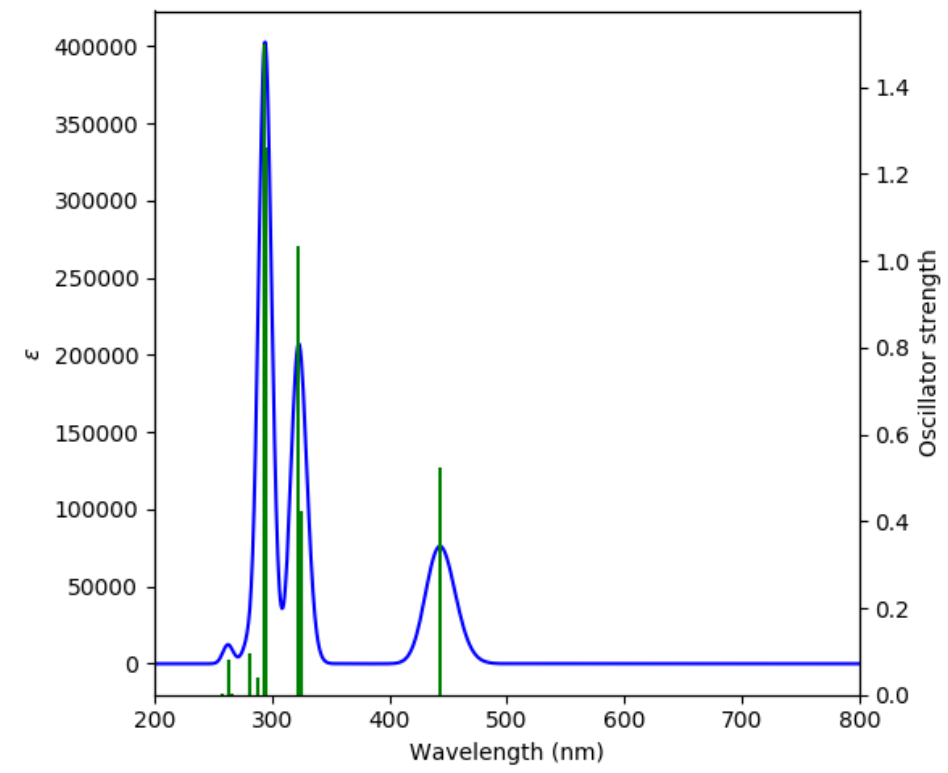


| No. | Energy (cm$^{-1}$) | $\lambda$ (nm) | Osc. Strength | Major Contributions |
|---|---|---|---|---|
| 1 | 22566.5863 | 443.1330 | 0.5257 | H-1->LUMO (23%), HOMO->LUMO (71%) |
| 2 | 30790.2153 | 324.7785 | 0.4226 | H-2->L+2 (16%), H-1->L+1 (19%), HOMO->L+1 (40%) |
| 3 | 31051.5389 | 322.0452 | 1.0331 | H-4->LUMO (22%), H-2->LUMO (51%) |
| 4 | 33963.2004 | 294.4363 | 1.2606 | H-1->L+1 (30%), HOMO->L+3 (40%) |
| 5 | 34031.7575 | 293.8431 | 1.5007 | H-4->LUMO (10%), H-2->L+1 (29%), HOMO->L+2 (35%) |
| 6 | 34773.7876 | 287.5729 | 0.0399 | H-1->LUMO (49%), HOMO->LUMO (24%) |
| 7 | 35664.2237 | 280.3930 | 0.0946 | H-15->LUMO (52%) |
| 8 | 37313.6275 | 267.9986 | 0.0004 | H-10->LUMO (17%), H-6->LUMO (26%) |
| 9 | 37643.5083 | 265.6500 | 0.0010 | H-1->L+2 (20%), HOMO->L+4 (15%) |
| 10 | 38004.8446 | 263.1243 | 0.0016 | H-3->L+1 (14%), H-2->L+2 (17%), H-1->L+3 (16%), HOMO->L+5 (20%) |
| 11 | 38084.6935 | 262.5726 | 0.0823 | H-15->LUMO (13%), H-9->LUMO (13%), H-7->LUMO (31%) |
| 12 | 38802.5270 | 257.7151 | 0.0013 | H-5->L+1 (12%), H-1->L+8 (16%), HOMO->L+6 (12%) |

**Figure S75.** Calculated absorbance spectrum and electronic transitions for **7** in DMSO.

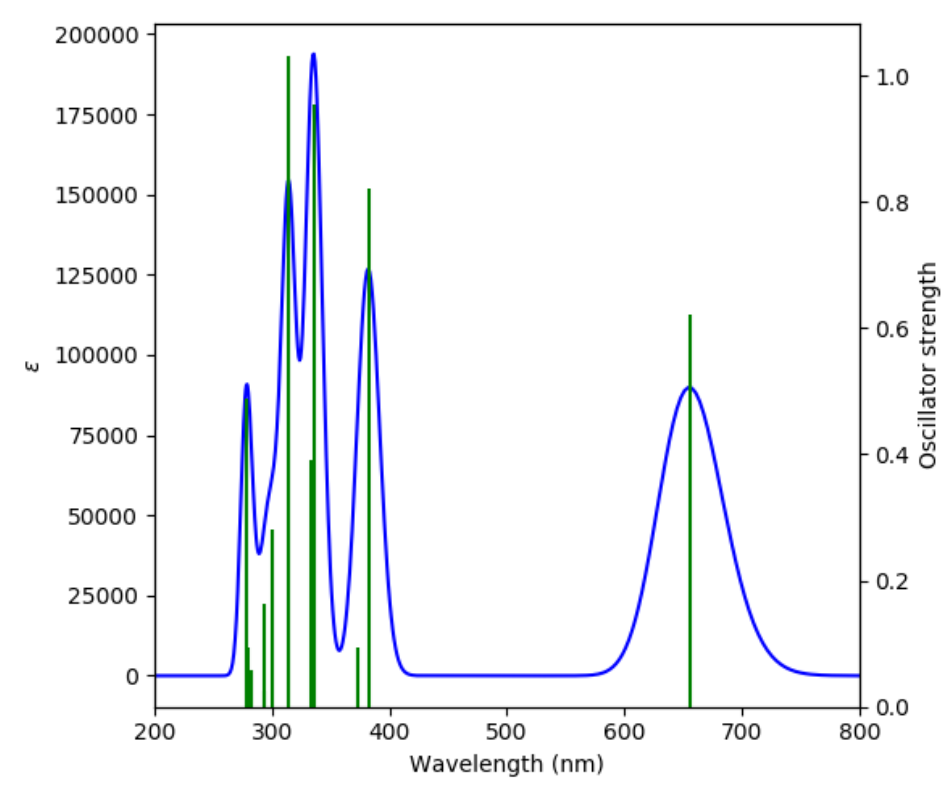


| No. | Energy (cm$^{-1}$) | $\lambda$ (nm) | Osc. Strength | Major Contributions |
|---|---|---|---|---|
| 1 | 15255.9770 | 655.4807 | 0.6204 | HOMO->LUMO (95%) |
| 2 | 26135.5897 | 382.6200 | 0.8218 | H-4->LUMO (17%), H-2->LUMO (72%) |
| 3 | 26825.1937 | 372.7838 | 0.0928 | HOMO->L+1 (47%), HOMO->L+3 (27%) |
| 4 | 29745.7273 | 336.1827 | 0.9540 | HOMO->L+2 (67%) |
| 5 | 29952.2052 | 333.8652 | 0.3922 | H-1->LUMO (59%) |
| 6 | 31820.1853 | 314.2659 | 1.0319 | H-1->LUMO (11%), H-1->L+1 (35%), H-1->L+3 (12%), HOMO->L+3 (11%) |
| 7 | 33234.0752 | 300.8959 | 0.2813 | H-16->LUMO (29%), H-9->LUMO (12%) |
| 8 | 34089.8294 | 293.3426 | 0.0245 | H-11->LUMO (30%), H-3->LUMO (29%), H-1->LUMO (15%) |
| 9 | 34138.2227 | 292.9267 | 0.1642 | H-9->LUMO (29%), H-4->LUMO (23%) |
| 10 | 35446.4540 | 282.1156 | 0.0585 | H-15->LUMO (13%), H-3->LUMO (18%), HOMO->L+1 (11%), HOMO->L+5 (14%) |
| 11 | 35768.2692 | 279.5774 | 0.0952 | H-16->LUMO (34%), H-9->LUMO (13%), H-4->LUMO (13%) |
| 12 | 35970.7144 | 278.0039 | 0.4874 | H-4->LUMO (19%), H-2->LUMO (11%), H-2->L+1 (11%), H-1->L+2 (11%), HOMO->L+4 (14%) |

**Figure S76.** Calculated emission spectrum and electronic transitions for **7** in DMSO.

*Molecular Orbitals (MOs)*

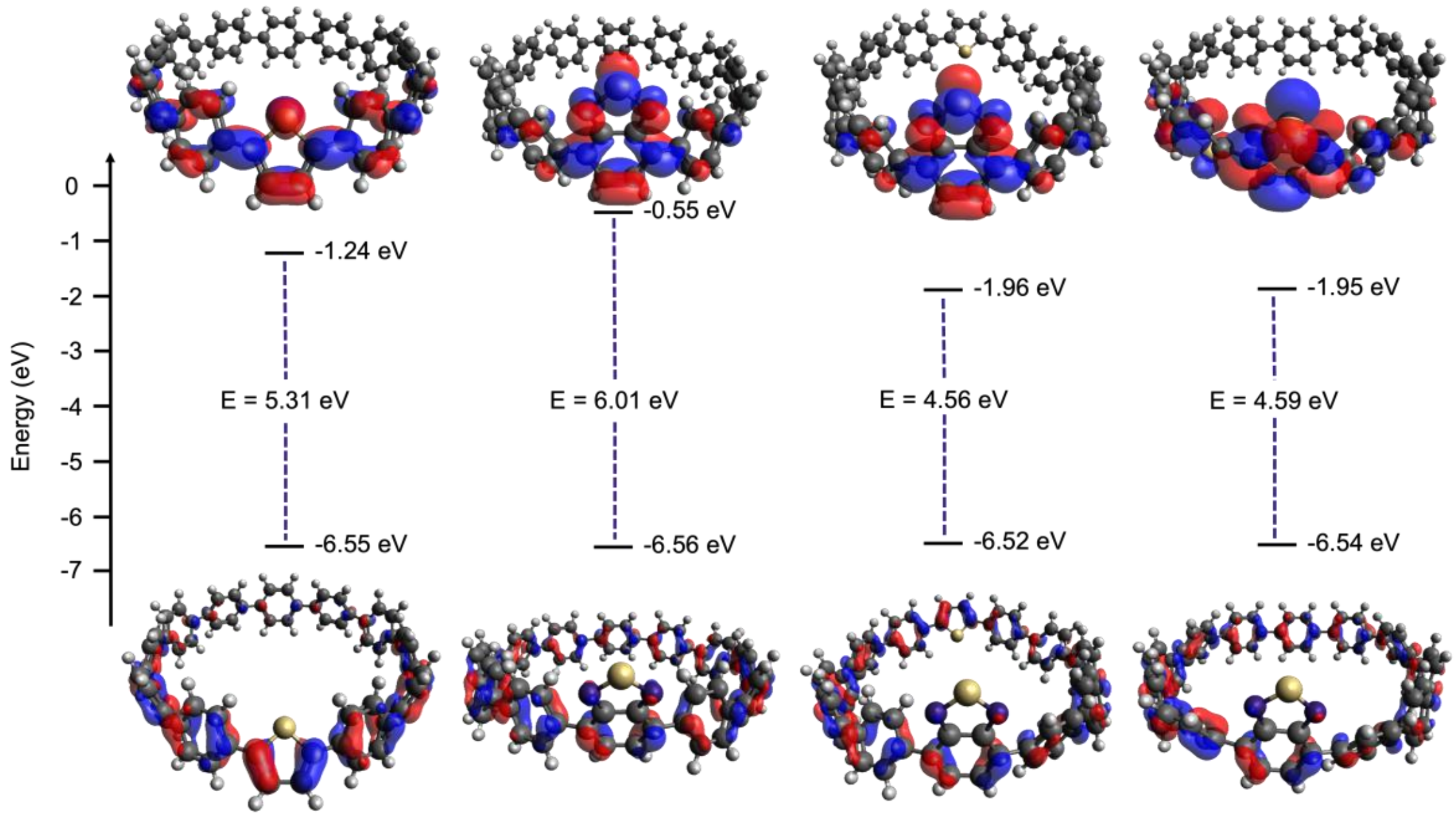


**Figure S77.** HOMO and $S_{1'}$ of **3**, **1**, **5**, and **4** using a CAM-B3LYP function, a 6-31G** basis set, and the solvent being dichloromethane.

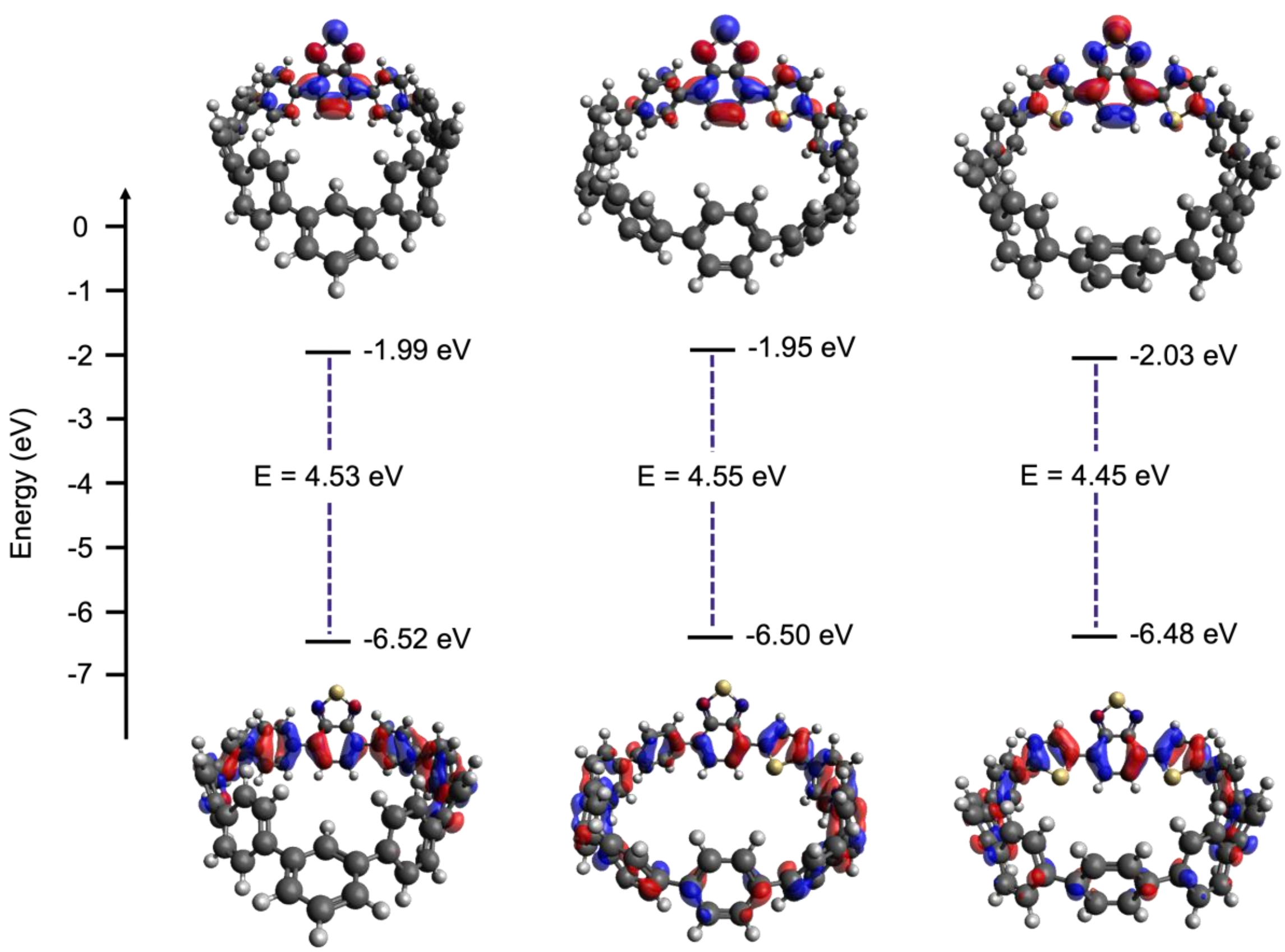


**Figure S78.** HOMO and $S_{1'}$ of **2**, **6**, and **7** using a CAM-B3LYP function, a 6-31G** basis set, and the solvent being dichloromethane.

## 5. StrainViz Calculations

All calculations were performed using Gaussian 09. The optimized ground state geometries used to calculate the predicted UV-vis spectra in DCM, were used to prepare the .xyz files of **2**, **7**, **BT[10]CPP**, and **BT-6-thio[10]CPP** and their corresponding fragments. StrainViz calculations were visualized in VMD.

Outputs from individual StrainViz calculations for **2**, **BT[10]CPP**, and **BT-6-thio[10]CPP** were re-submitted with the maximum bond energy set to that of [8]CPP to generate a relative strain scale. This setting re-colors each bond to generate StrainViz structures that are now comparative. In the “bond_scripts.py” file, line 171 “max(norm_values/31” was replaced with the max bond strain value in the “total_bond.tcl” file for [8]CPP. Values in the “total_bond.tcl” file are in kcal mol$^{-1}$ and therefore need to be converted to Hartrees before being inputted in the “bond_scripts.py” file.

Attempts to use the highest strained bond between the three molecules yielded incorrect coloring in the StrainViz heat map (e.g., whole molecule was green). Using the maximum bond energy for [8]CPP was found to re-color the structures analogous to the initial, individual StrainViz calculations. The ground state geometry for [8]CPP, necessary to make the .xyz files, was obtained by using a CAM-B3LYP function, the 6-31G** basis set, and the effect of the solvent was included by the polarizable continuum model (PCM) using dichloromethane.

Fragment geometries are shown below:

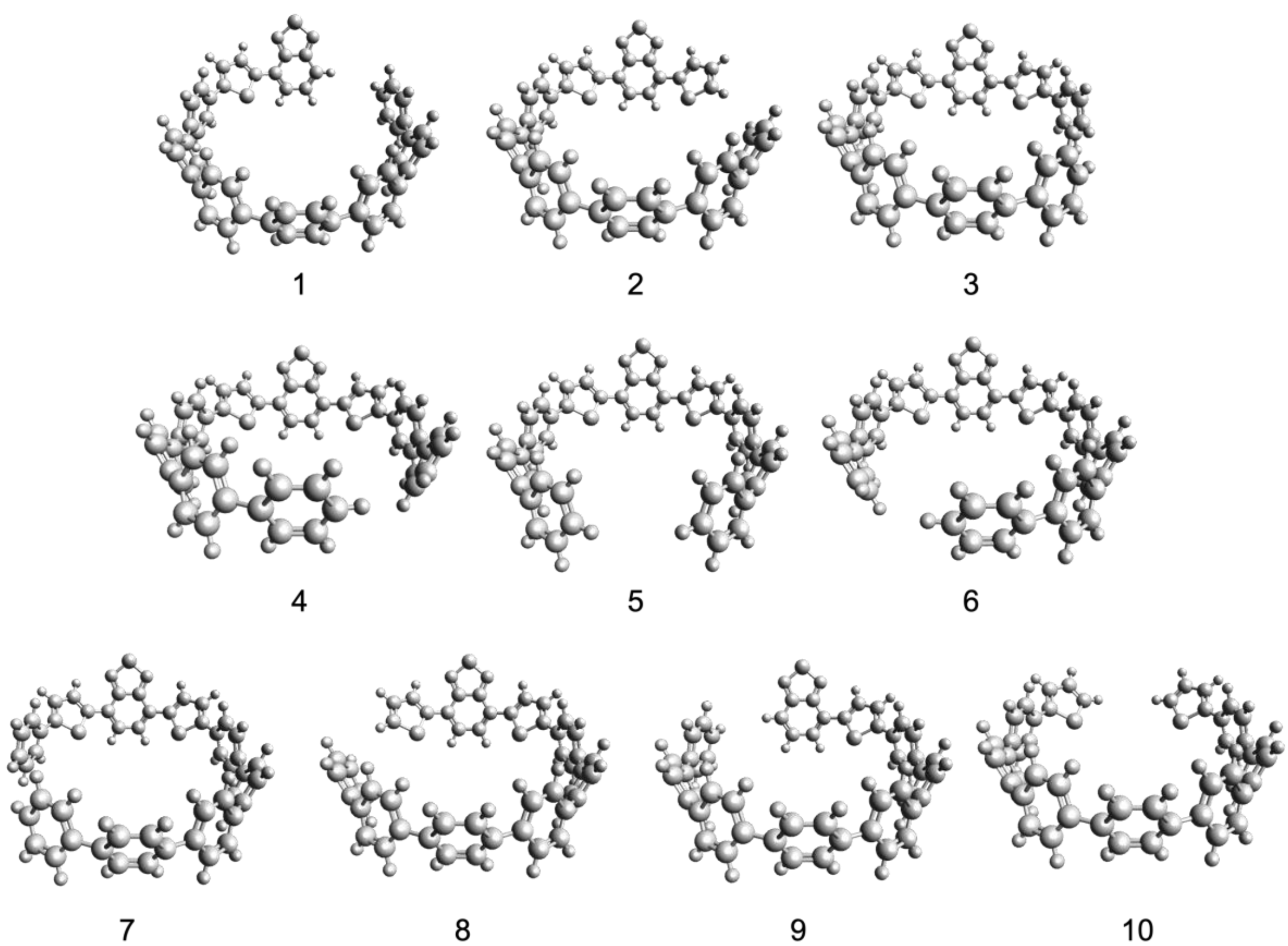


**Figure S79.** Fragments of **7** for use in StrainViz.

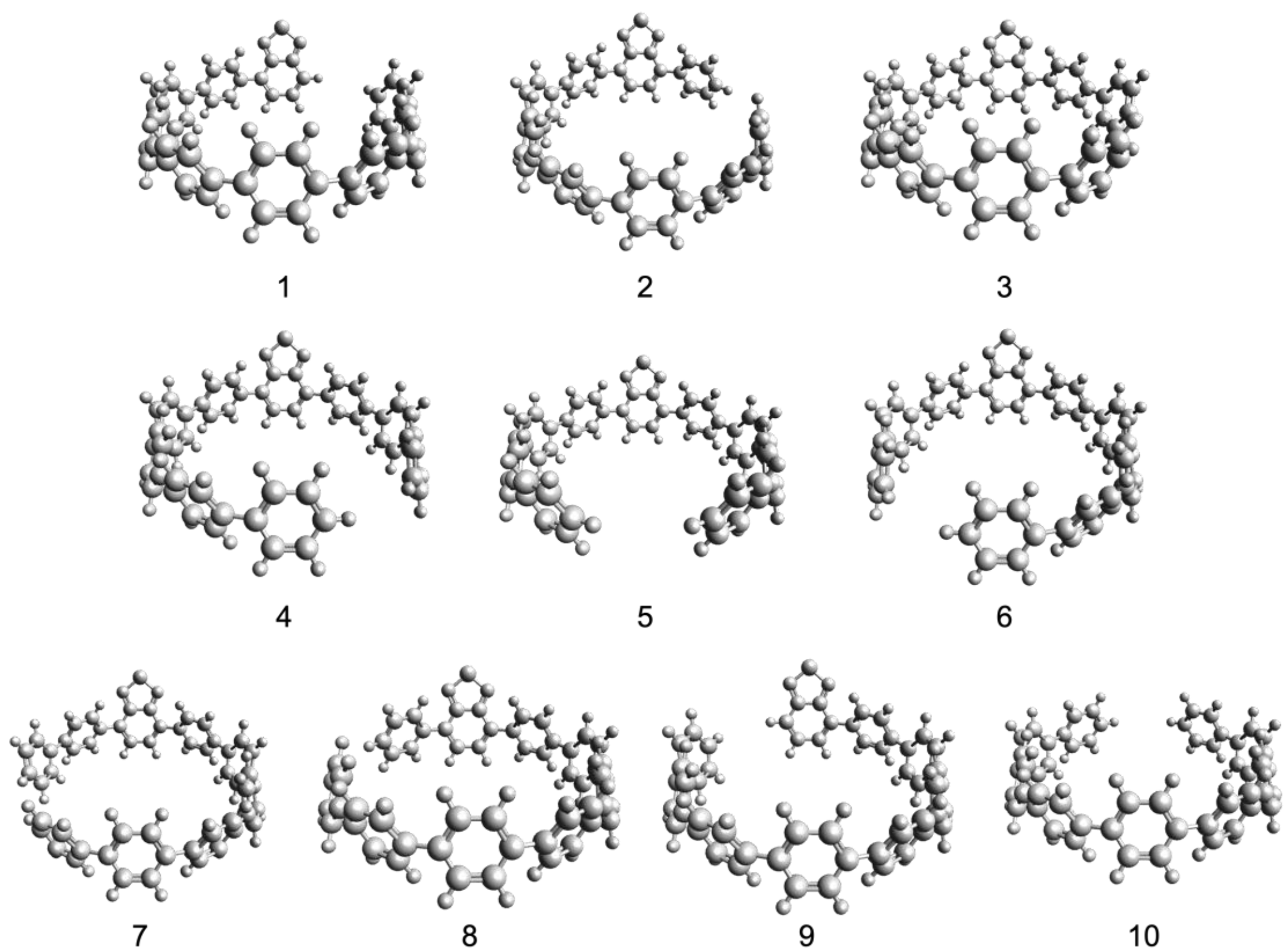


**Figure S80.** Fragments of **BT[10]CPP** for use in StrainViz.

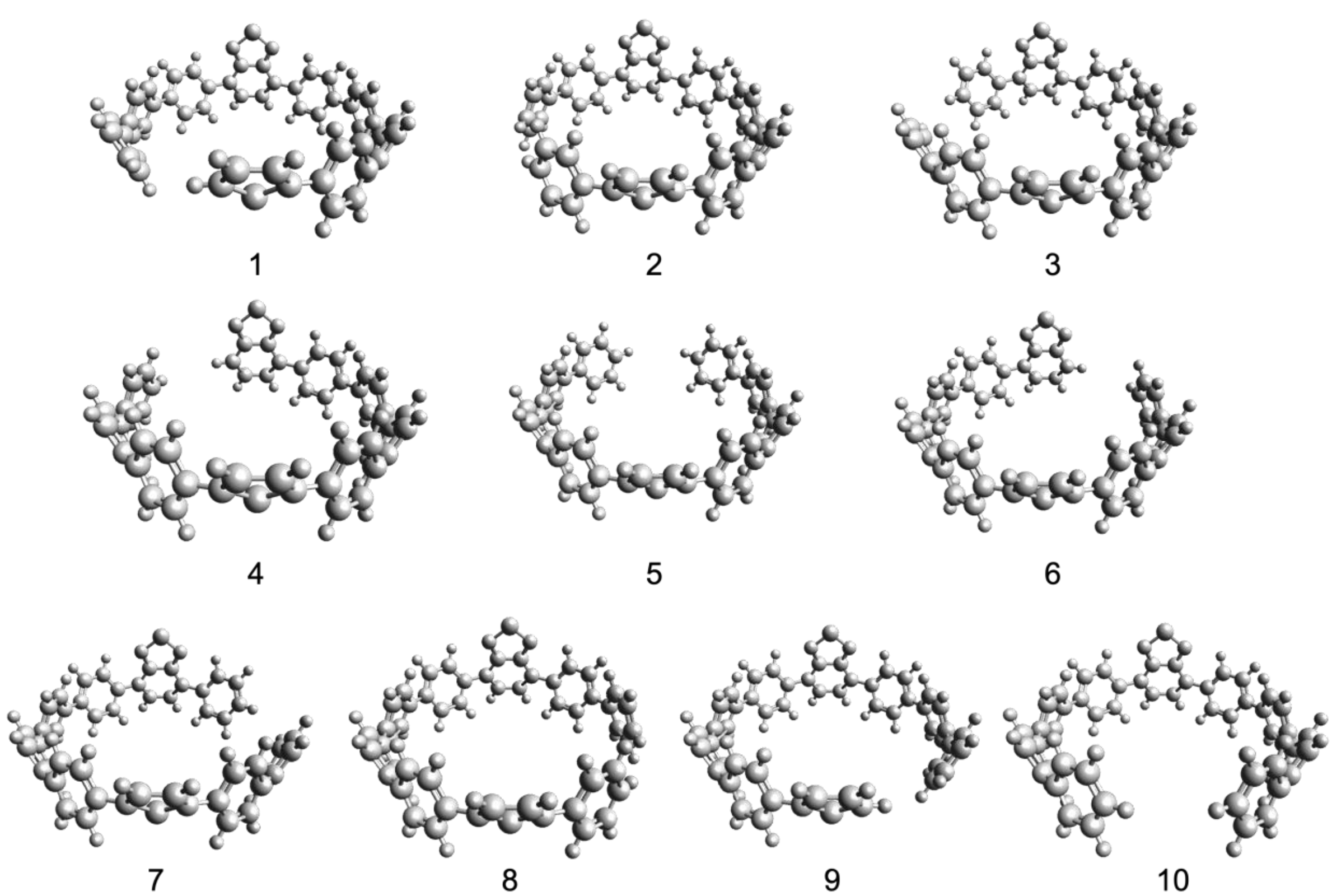


**Figure S81.** Fragments for **BT-6-thio[10]CPP** for use in StrainViz.

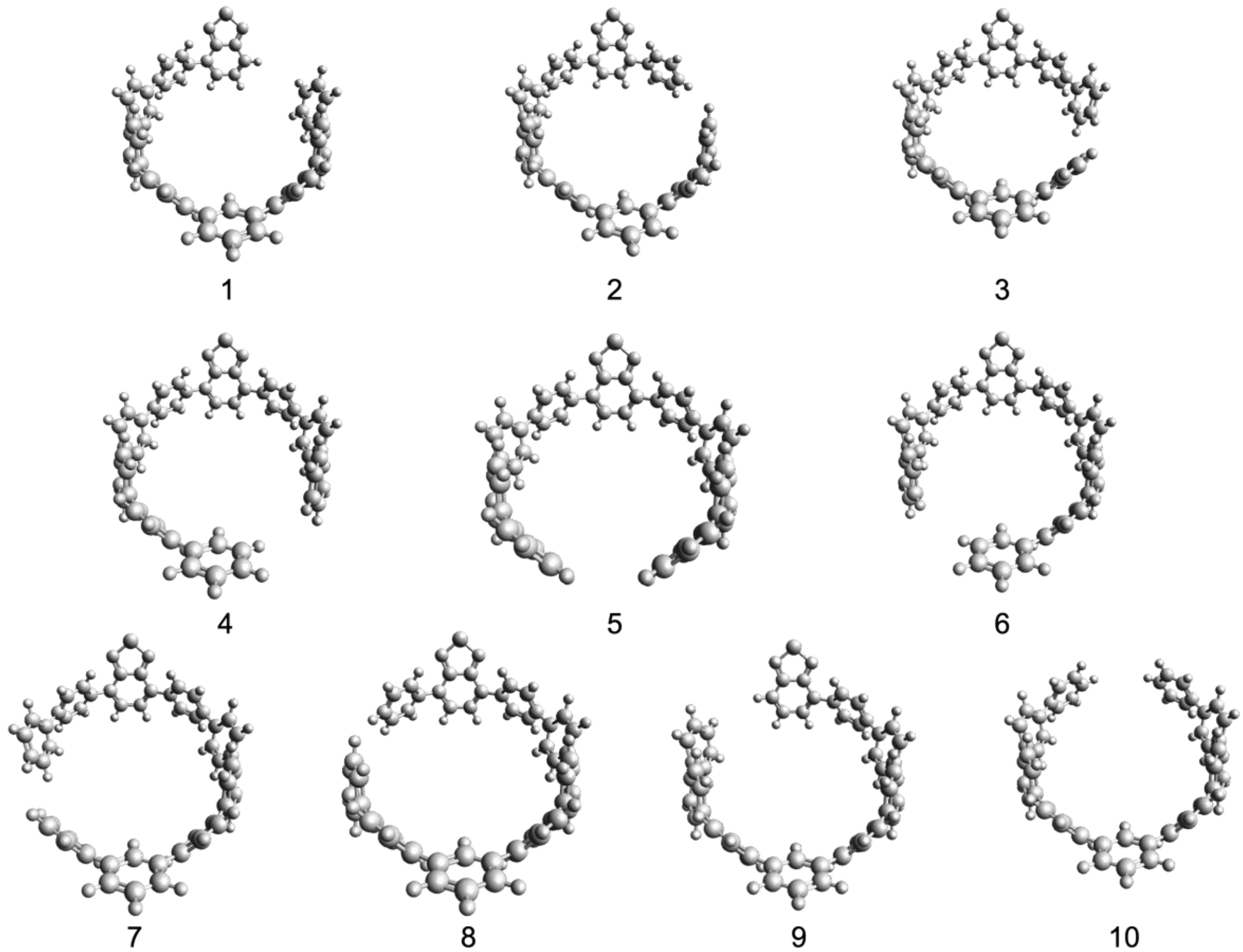


**Figure S82.** Fragments of **2** for use in StrainViz.

## 6. X-Ray Crystallography

Single crystals of **7** were grown by slow evaporation of DCM and diethyl ether at room temperature.

*X-ray crystallography*. Diffraction intensities for **7** were collected at 173 K on Bruker Apex2 diffractometer using CuK$\alpha$ radiation, 1.54178 Å. Space group was determined based on systematic absences. Absorption correction was applied by SADABS.[13] Structure was solved by direct methods and Fourier techniques and refined on $F^2$ using full matrix least-squares procedures. All non-H atoms were refined with anisotropic thermal parameters. H atoms in the structure were refined in calculated positions in a rigid group model. X-ray diffraction from crystals at high angles was very weak due to a high disorder of solvent molecules inside the structure. Even by using a strong X-ray micro focus source it was possible to collect diffraction data only up to $2\theta_{max}$ = 99.73°. However, the data collected provide appropriate number of measured reflections per refined parameters, 4993 per 550. The main molecules form columns in the crystal structure. Solvent molecules ($CH_2Cl_2$ and $Et_2O$ were used) fill out a space inside these columns sharing the same positions. Their positions are highly disordered or partially occupied. These solvent molecules were not resolved and were treated by SQUEEZE.[14] They are not included in the formula of the compound given in the CIF file. All calculations were performed by the Bruker SHELXL-2014/7 package.[15]

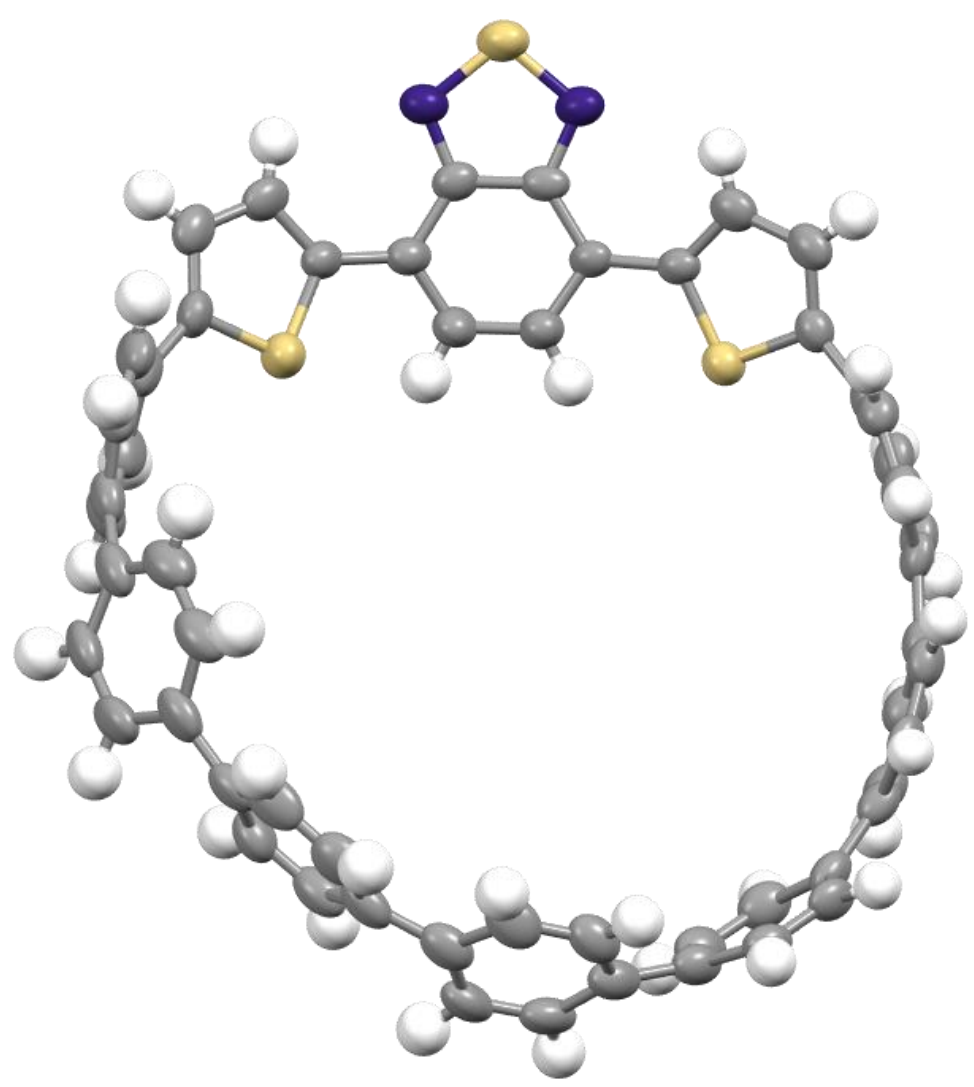

*Crystallographic Data for* **7** *(CCDC 2550070)*: $C_{56}H_{34}N_2S_3$, M = 831.03, 0.14 x 0.10 x 0.02 mm, T = 173(2) K, Monoclinic, space group *P*2$_1$/*n*, *a* = 9.6588(6) Å, *b* = 28.3941(18) Å, *c* = 10.0364(10) Å, $\beta$ = 98.163(5)°, *V* = 4896.4(5) Å$^3$, *Z* = 4, Z' = 1, *D*c = 1.127 Mg/m$^3$, $\mu$(Cu) = 1.659 mm$^{-1}$, F(000) = 1728, $2\theta_{max}$ = 99.73°, 17025 reflections, 4993 independent reflections [$R_{int}$ = 0.0820], R1 = 0.0504, wR2 = 0.0993 and GOF = 1.041 for 4993 reflections (550 parameters) with I>2σ(I), R1 = 0.0883, wR2 = 0.1092 and GOF = 1.041 for all reflections, max/min residual electron density +0.174/-0.264 eÅ$^{-3}$.

## 7. References


1. T.C. Lovell, C. E. Colwell, L. N. Zakharov, and R. Jasti, *Chem. Sci.* 2019, **10**, 3786–3790.
2. J. M. Fehr, N. Myrthil, A. L. Garrison, T. W. Price, S. A. Lopez, and R. Jasti, *Chem. Sci.* 2023, **14**, 2839–2848.
3. E. R. Darzi, B. M. White, L. K. Loventhal, L. N. Zakharov, and R. Jasti, *J. Am. Chem. Soc.* 2017, **139**, 3106–3114.
4. Sigma-Aldrich (Spectral data were obtained from Advanced Chemistry Development, Inc.
5. N. C. Bruno, M. T. Tudge, and S. L. Buchwald. Chem. Sci., 2013, 4, 916–920.
6. X. Li, L. Liu, L. Jia, Z. Lian, J. He, S. Guo, Y. Wang, X. Chen, and H. Jiang, *Nat Commun* 2025, **16**, 467
7. T. C. Lovell, T. Z. R. Garrison, and R. Jasti, *Angew. Chem. Int. Ed.* 2020, **59**, 14363–14367.
8. A. B. Pangborn, M. A. Giardello, R. H. Grubbs, R. K. Rosen, and F. J. Timmers. Organometallics, 1996, 15, 1518–1520.
9. Horiba Scientific: A Guide to Recording Fluorescence Quantum Yields. https://static.horiba.com/fileadmin/Horiba/Application/Materials/Material_Research/Quantum_Dots/quantumyieldstrad.pdf.
10. N. Mataga, Y. Kaifu, and M. Koizumi, *Bull. Chem. Soc. Jpn*. 1955, **28**, 690-691.
11. S. Canola, L. Mardegan, G. Bergamini, M. Villa, A. Acocella, M. Zangoli, L. Ravotto, S. A. Vinogradov, F. Di Maria, P. Ceroni, and F. Negri, *Photochem. Photobiol. Sci*. 2019, **18**, 2180-2190.
12. R. Yoshida, T. Tachikawa, and S. Ito, *Cryst. Growth Des*. 2022, **22**, 547-558.
13. G. M. Sheldrick, *Bruker/Siemens Area Detector Absorption Correction Program*, Bruker AXS, Madison, WI, 1998.
14. P. Van der Sluis and A. L. Spek, *Acta Cryst., Sect. A* 1990, **A46**, 194-201.
15. G. M. Sheldrick, *Acta Cryst.* 2015 **C71**, 3-8.
