# Supplementary material for: Design rules for tuning charge-transfer emission in donor–acceptor nanohoops
Source: Chem Sci. 2026 Jul 16. Online ahead of print. doi: 10.1039/d6sc03959f (PMC13373506; doi:10.1039/d6sc03959f)
Supplement: SC-OLF-D6SC03959F-s001 [file SC-OLF-D6SC03959F-s001.pdf]

Supplementary Information (SI) for Chemical Science.
This journal is © The Royal Society of Chemistry 2026

Supporting Information

Design Rules for Tuning Charge-Transfer Emission in Donor-Acceptor Nanohoops

Gabriela M. Bailey[a], Ethan Q. Nguyen[a], Melanie A. Sheldon[a], Lev N. Zakharov[b], and Ramesh Jasti[a]*

[a] Department of Chemistry and Biochemistry, Materials Science Institute, and Knight Campus for Accelerating Scientific Impact, University of Oregon, Eugene, Oregon 97403, USA
[b] CAMCOR – Center for Advanced Materials Characterization in Oregon, University of Oregon, Eugene, Oregon 97403, USA

*Email: rjasti@uoregon.edu

Table of Contents

1. General Information S1

2. Synthesis S2-24

- a. **Intermediate Synthesis**
  - *One and Two Ring Intermediates* S2-S3
  - *Three Ring Containing Intermediates* S3-S4
  - *Five Ring Containing Intermediates* S5-S9
  - *Seven Ring Containing Intermediates* S10-S14
- b. **D-A[12]CPP Derivatives** S15-S20
- c. **D-A[10]CPP Derivatives** S21-S24

3. Nuclear Magnetic Resonance Spectra S25-44

4. Experimental and Computational Photophysical Characterization S45-S66

- a. **Experimental Photophysical Characterization** S45-S49
  - *Quantum Yield Data* S45-S48
  - *Solvatofluorochromism Data* S49
- b. **Data for Literature Reported Small Molecule, Linear Analogues** S50
- c. **Computational Photophysical Characterization** S51-66
  - *Predicted Absorbance and Emission Values* S51-65
  - *Molecular Orbitals (MOs)* S66

5. StrainViz Calculations S67-69

6. X-Ray Crystallography S70

7. References S71

1. General Information

Synthesis: Unless otherwise noted, commercially available starting materials, ligands, and catalysts were used without purification. *n*-Butyllithium (2.5 M or 1.6 M in *n*-hexanes) was purchased from either ThermoFischer Scientific with AcroSeal^{TM} or from Sigma-Aldrich with Sure/Seal^{TM} and used as received. Intermediates **S1**, **S2**, **S3**, **S4**, **S5**, **8**, **9**, **10**, **12**, and **16** and catalyst Pd SPhos GIII were prepared according to the literature.[1-5] Control compounds **Thio[10]CPP** and **BT[10]CPP** were synthesized using previous reports.[6,7] Moisture and oxygen-sensitive reactions were carried out in flame-dried or overnight oven-dried glassware under an inert atmosphere of purified nitrogen using standard syringe/Schlenk techniques. Tetrahydrofuran (THF), dimethylformamide (DMF), and 1,4-dioxane were dried by filtration through alumina according to the methods described by Grubbs.[8] Thin-layer chromatography (TLC) was performed either on alumina plates coated with 0.20 mm thickness of Silica Gel 60 F_{254} (Supelco) or alumina plates coated with 0.20 mm thickness of Aluminum Oxide Neutral (Miles Scientific). Developing plates were visualized using UV light at wavelengths of 254 and 365 nm. Silica column chromatography was conducted with Zeochem Zeoprep 60 HYD 40-63 µm silica gel. Alumina column chromatography was conducted with Sorbtech basic alumina (pH 10), Act. II-III, 50-200 µm.

Instrumentation: ^{1}H and ^{13}C NMR spectra were recorded on either a Bruker Avance III HD 500 MHz (^{1}H: 500 MHz, ^{13}C: 126 MHz) or a Bruker Avance III HD 600 MHz (^{1}H: 600 MHz, ^{13}C: 151 MHz) NMR spectrometer. The samples were measured at 25 °C. The chemical shifts (δ) were reported in parts per million (ppm) and were referenced to the residual protio-solvent (CD_2Cl_2, ^{1}H: δ = 5.32 ppm and ^{13}C: δ = 53.84 ppm; $CDCl_3$, ^{1}H: δ = 7.26 ppm). Coupling constants (J) are given in Hz and the apparent resonance multiplicity is reported as s (singlet), d (doublet), t (triplet), q (quartet), dd (doublet of doublets), dt (doublet of triplets), dq (doublet of quartets), td (triplet of doublets), or m (multiplet). The resonances for carbon atoms attached to the boron were not observed due to the boron quadrupole. Infrared absorption (IR) spectra were recorded on a Thermo Scientific Nicolet 6700 spectrometer equipped with either a diamond crystal Smart ATR or KBr pellet from Sigma-Aldrich. Characteristic IR absorptions are reported in cm^{-1} and denoted as strong (s), medium (m), and weak (w). High-resolution mass spectra (HRMS) were obtained from the Mass Spectrometry Lab of the University of Illinois at Urbana-Champaign in Champaign, Illinois, USA. UV/Vis absorption and fluorescence spectra were recorded on an Agilant Cary 100 spectrophotometer and a Horiba Jobin Yvon Fluoromax-4 Fluorimeter, respectively. All measurements were carried out under ambient conditions in a Spectrocell RF-1010-T threaded top vacuum formed borosilicate fluorometer cell (10 mm light path).

2. Synthesis

a. Intermediate Synthesis

Synthesis of One and Two Ring Containing Intermediates

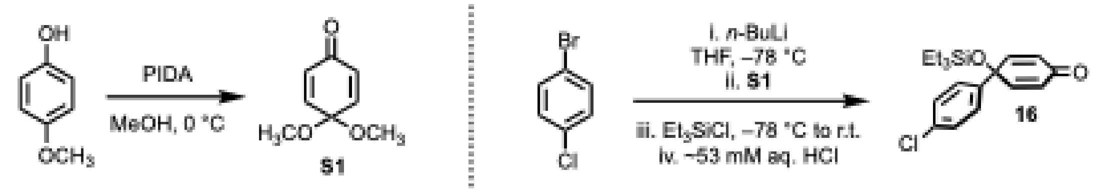

Scheme S1. Synthesis of intermediates **S1** and **16**.

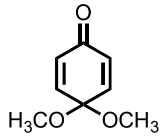

S1. To a 2 L round-bottomed flask equipped with a stir bar and containing MeOH (200 mL), 4-methoxyphenol (100 g, 0.806 mol, 1 equiv.) was dissolved in increments of four. Additional MeOH (695 mL) was added, using a portion to rinse any residual solid into the reaction mixture. Cooled the reaction flask to 0 °C over the course of 1 hour before adding PIDA (285 g, 0.886 mol, 1.1 equiv.) over 1.5 hours, observing the reaction color go from colorless to dark yellow. Allowed the reaction vessel to come to r.t. after all the oxidant had been added and left the reaction to run overnight. Neutralized the reaction mixture with sat. sodium bicarbonate (~500 mL) and removed roughly half of excess solvent *in vacuo* - reaction mixture turned black as it was concentrated down. Extracted the organics with DCM (200 mL x 6) and then washed the organics with water (100 mL x 1), brine (200 mL x 3), and aq. NaOH (50 mL x 1, 2 M). Dried the organics over sodium sulfate, gravity filtered the mixture and then removed solvent *in vacuo*. **Purification:** To the crude material, excess iodobenzene was removed via vacuum-distillation until approximately 12% iodobenzene remained (percentage calculated by integration via ^{1}H NMR). The material was further purified by column chromatography (loaded neat, pH 10 AlO_x, 100% hexanes and then 20% EtOAc:Hex once pdt began eluting) yielding light-yellow oil **S1** (96.5 g, 78% yield). The ^{1}H NMR (500 MHz, Chloroform-*d*) matched previous literature reports.

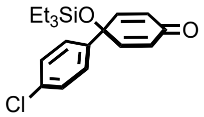

16. An oven-dried 500 mL three-necked flask equipped with a stir bar, a glass vacuum adapter, a glass stopper, and a 125 mL addition funnel capped with a glass stopper were cooled on high vacuum. Charged the reaction vessel with 1-bromo-4-chlorobenzene (27.4 g, 0.143 mol, 1 equiv.) before backfilling and evacuating the system (3 cycles x 3 min). Exchanged the glass stoppers for septa under positive nitrogen flow. Dissolved the solid in dry THF (275 mL) and cooled the reaction vessel to –78 °C over the course of 1 hour. Cannula transferred *n*-butyllithium (60.1 mL, 2.5 M in hexanes, 1.05 equiv.) to the addition funnel and set the reagent to add dropwise. Once all the lithiating agent had been added, the addition funnel was replaced with a rubber septum under positive nitrogen flow, any residual *n*-butyllithium on the funnel was quenched, and neat **S1** (20.1 mL, 0.143 mol, 1 equiv.) was added dropwise to the reaction vessel. Allowed the reaction to stir for 1 hour before adding neat Et_3SiCl (28.8 mL, 0.171 mol, 1.2 equiv.) dropwise. Left the reaction to come to r.t. overnight. Into the three-necked flask was slowly poured an acidic, aq. mixture (40 mL DI water with five drops conc. HCl) and was left to stir for 30 minutes. The reaction

mixture was quenched with sat. sodium bicarbonate (~100 mL) and excess organic solvent was removed *in vacuo*. Extracted the organics using ethyl acetate (~150 mL x 3) before the organics were washed with brine (~150 mL x 3), dried over sodium sulfate, gravity filtered, and then removed solvent *in vacuo*. **Purification:** The material was further purified by a short plug (loaded neat, SiO_2, 100% hexanes and then 4% EtOAc:Hex once pdt began eluting) to yield light, yellow oil **16** (45.3 g, 96% yield). The ^{1}H NMR (500 MHz, Chloroform-*d*) matched previous literature reports.

Synthesis of Three Ring Containing Intermediates

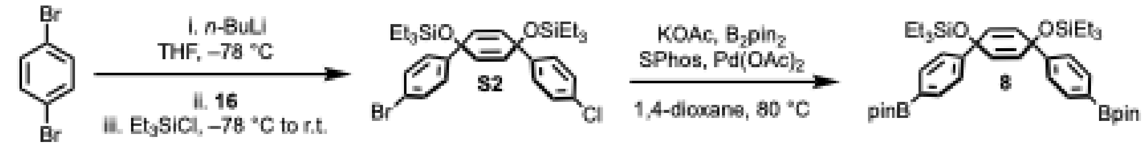

Scheme S2. Synthesis of intermediates **S2** and **8**.

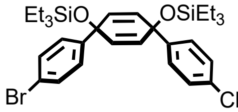

S2. Flame-dried a 100 mL three-necked flask equipped with a stir bar, a glass vacuum adapter, and two glass stoppers. Once cool, the flask was charged with 1,4-dibromobenzene (4.65 g, 19.7 mmol, 1 equiv.) and the system was backfilled and evacuated (3 cycles x 3 min). A glass stopper was exchanged for a septum under positive nitrogen flow, and the solid was dissolved in dry THF (66 mL) before cooling the flask to –78 °C over the course of 30 minutes. Added *n*-butyllithium (8 mL, 2.5 M in hexanes, 1.02 equiv.) to the cooled solution dropwise. Allowed the reaction to stir for 10 minutes before adding neat **16** (6.6 g, 19.7 mmol, 1 equiv.) dropwise. The reaction was left to stir for 1.25 hours before taking an aliquot to confirm all 1,4-dibromobenzene had been consumed (checked by TLC: SiO_2, 5% EtOAc:Hex). To the reaction vessel was added neat Et_3SiCl (3.5 mL, 0.02 mmol, 1.06 equiv.) dropwise. Brought the flask to r.t. before moving the flask to a pre-heated oil bath set at 40 °C. After 1.5 hours, the crude reaction mixture was worked up – after a second aliquot had confirmed the reaction had fully progressed (checked by TLC: SiO_2, 5% EtOAc:Hex). Removed excess solvent *in vacuo* before the organics were extracted with ethyl acetate (~80 mL x 3). The organics were washed with brine (~60 mL x 3), dried over sodium sulfate, gravity filtered, and then removed solvent *in vacuo*. **Purification:** The material was purified by column chromatography (loaded in hexanes, SiO_2, 100% hexanes and then 10% DCM:Hex once **16** starting material had all eluted off) to yield crystalline, white solid **S2** (7.12 g, 60% yield). The ^{1}H NMR (500 MHz, Chloroform-*d*) matched previous literature reports.

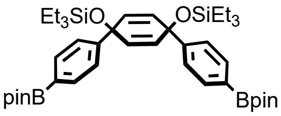

8. A 50 mL round-bottomed flask equipped with a stir bar and charged with KOAc (3.74 g, 38.1 mmol, 6.6 equiv.) was oven-dried for ~3.5 hours before cooling the flask on high vacuum. To the reaction vessel was added **S2** (3.5 g, 5.77 mmol, 1 equiv.), B_2pin_2 (3.00 g, 1.18 mmol, 2.05 equiv.), SPhos (0.296 g, 0.721 mmol, 0.125 equiv.), and $Pd(OAc)_2$ (19.4 mg, 0.0864 mmol, 0.015 equiv.). Backfilled and evacuated the system with a glass vacuum adapter (3 cycles x 3 min) and then with a septum and needle (3 cycles x 3 min). The solids were dissolved in dry 1,4-dioxane (20 mL) before the reaction vessel was lowered into a pre-heated oil bath set to 80 °C and left to run overnight. An aliquot of the reaction mixture was taken to ensure both starting materials had been consumed (checked by ^{1}H NMR). Brought the reaction mixture to r.t. before running the crude

through a plug (Celite topped with a thin layer of sodium sulfate, 100% EtOAc). Concentrated the filtrate *in vacuo* before drying the crude under high vacuum. **Purification:** To remove catalyst, the material was iteratively filtered through a plug (loaded in hexanes, Celite topped with a thin layer of SiO_2, 5% EtOAc:Hex) to yield powdery, white solid **8** (2.54 g, 59% yield). The ^1H NMR (500 MHz, Chloroform-*d*) matched previous literature reports.

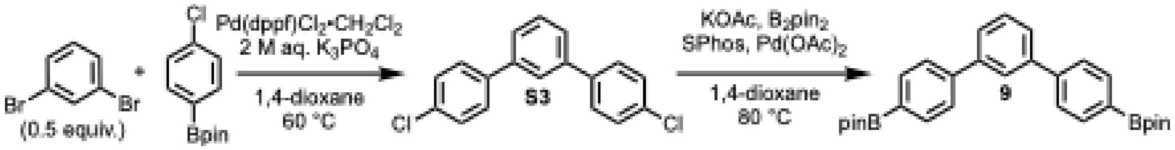

Scheme S3. Synthesis of intermediates **S3** and **9**.

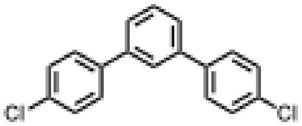

S3. Flame-dried a 250 mL round-bottomed flask equipped with a stir bar. Charged the flask with 4-chlorophenylboronic acid pinacol ester (4.05 g, 0.017 mol, 2.05 equiv.) and $Pd(dppf)Cl_2•CH_2Cl_2$ (0.303 g, 0.371 mmol, 0.045 equiv.) before the system was backfilled and evacuated using a septum and needle (3 cycles x 3 min). Dissolved the solids in dry 1,4-dioxane (83 mL) before adding neat 1,3-dibromobenzene (1 mL, 8.28 mmol, 1 equiv.) dropwise. The reaction mixture was sparged for 1 hour before lowering the flask into a pre-heated oil bath set to 80 °C. Allowed the reaction vessel to equilibrate to temperature for 15 minutes before adding sparged, aq. K_3PO_4 (8.3 mL, 2 M) dropwise. Left the reaction to run overnight. The reaction was monitored to ensure all starting material had been consumed (checked by ^1H NMR), and if the reaction was stalled, another complete amount of aq. K_3PO_4 (8.3 mL, 2 M) was added dropwise. Excess solvent was removed *in vacuo*, and the crude mixture was run through a plug (Celite topped with a thin layer of sodium sulfate, 100% DCM). **Purification:** The crude material was initially purified via automated flash column chromatography (loaded in hexanes, SiO_2, 100% hexanes), followed by subsequent trituration in EtOH to yield powdery, white solid **S3** (0.55 g, 22% yield). The ^1H NMR (500 MHz, Chloroform-*d*) matched previous literature reports.

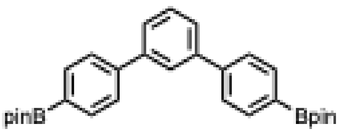

9. Flame-dried a 25 mL round-bottomed flask equipped with a stir bar. Once cool, charged the flask with KOAc (1.19 g, 0.0121 mol, 6.6 equiv.) before flame-drying the flask a second time. Backfilled and evacuated the flask once (1 cycle x 3 min) before adding **S3** (0.55 g, 1.84 mmol, 1 equiv.), B_2pin_2 (1.40 g, 5.51 mmol, 3 equiv.), SPhos (94.3 mg, 0.230 mmol, 0.125 equiv.), and $Pd(OAc)_2$ (6.10 mg, 0.0272 mmol, 0.015 equiv.). Backfilled and evacuated the flask using a glass vacuum adapter (3 cycles x 3 min) and then a septum and needle (2 cycles x 3 min). Dissolved the solids with dry 1,4-dioxane (6.12 mL) before lowering the reaction vessel into a pre-heated oil bath set to 80 °C. Allowed the reaction to run overnight. The reaction was monitored to ensure all starting material had been consumed (checked by ^1H NMR). Removed excess solvent *in vacuo* before the crude mixture was run through a plug (loaded in hexanes, SiO_2 topped with a thin layer of sodium sulfate, 100% DCM). **Purification:** The crude material was iteratively triturated in EtOH to remove excess B_2pin_2 and to afford powdery, white solid **9** (0.77 g, 87% yield). The ^1H NMR (500 MHz, Chloroform-*d*) matched previous literature reports.

Synthesis of Five Ring Containing Intermediates

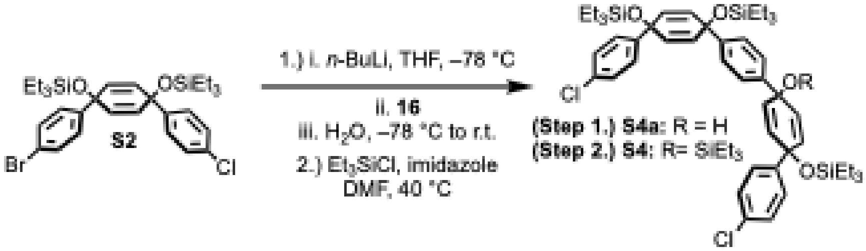

Scheme S4. Synthesis of intermediates **S4a** and **S4**.

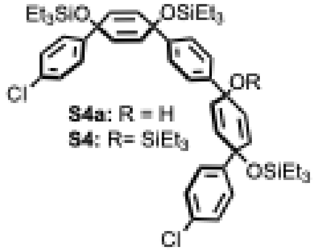

S4a. Lithium-halogen exchange: Flame-dried a 25 mL round-bottomed flask and a 5 mL round-bottomed flask, each equipped with a stir bar. Charged the 5 mL flask with neat **16** (0.56 g, 1.67 mmol, 1.01 equiv.) and left the material on high vacuum for 1 hour with stirring. Charged the 25 mL reaction flask with **S2** (1.00 g, 1.65 mmol, 1 equiv.) before the system was backfilled and evacuated using a glass vacuum adapter (3 cycles x 3 min) and then a septum and needle (3 cycles x 3 min). Dissolved the solids in the reaction vessel with dry THF (3.3 mL) before cooling the flask to –78 °C over the course of 15 minutes. To the reaction vessel added *n*-butyllithium (0.69 mL, 2.5 M in hexanes, 1.05 equiv.) dropwise. The reaction mixture went from colorless to light yellow. Let the reaction stir for 12 minutes before dissolving the contents of the 5 mL flask in dry THF (1 mL). Added the contents of the 5 mL flask to the reaction vessel dropwise, then rinsed the smaller flask with dry THF (~0.2 mL), and transferred the contents to the reaction vessel (x2). Allowed the reaction mixture to stir for 1 hour before slowly quenching the reaction mixture with DI water (10 mL). Removed excess solvent *in vacuo* before extracting the organics with ethyl acetate (30 mL x 2). Washed the organics with brine (30 mL x 3), dried over sodium sulfate, gravity filtered, and then removed the solvent *in vacuo*. The crude oil **S4a** was pushed forward without further purification.

S4. Triethylsilyl Protection: Flame-dried a 25 mL round-bottomed flask equipped with a stir bar. Backfilled and evacuated the system once (1 cycle x 3 min) before adding crude oil **S4a** (1.03 g, 1.19 mmol, 1 equiv.). To the flask added imidazole (0.244 g, 3.58 mmol, 3 equiv.) before backfilling and evacuating the system using a glass vacuum adapter (3 cycles x 3 min) and then a septum and needle (3 cycles x 3 min). Dissolved the solids in dry DMF (18 mL) before lowering the flask in a pre-heated oil bath set to 40 °C. Allowed the mixture to equilibrate to temperature over the course of 10 minutes before adding Et_3SiCl (0.21 mL, 1.25 mmol, 1.05 equiv.) dropwise. Left the reaction to stir overnight. The reaction mixture was brought to r.t. before quenching with sat. sodium bicarbonate (5 mL) and left to stir for 15 minutes before extracting the organics with ethyl acetate (30 mL x 3). Washed the organics with 5% LiCl (30 mL x 3), dried over sodium sulfate, gravity filtered and then removed the solvent *in vacuo*. To remove residual solvent the crude material was placed on high vacuum for 20 minutes, redissolved in hexanes before removing solvent *in vacuo*, then placed back on high vacuum for 20 minutes (x2). **Purification:** The crude material was triturated in EtOH to afford powdery, white solid **S4** (0.93 g, 80% yield). The ^1H NMR (500 MHz, Chloroforom-*d*) matched previous literature reports.

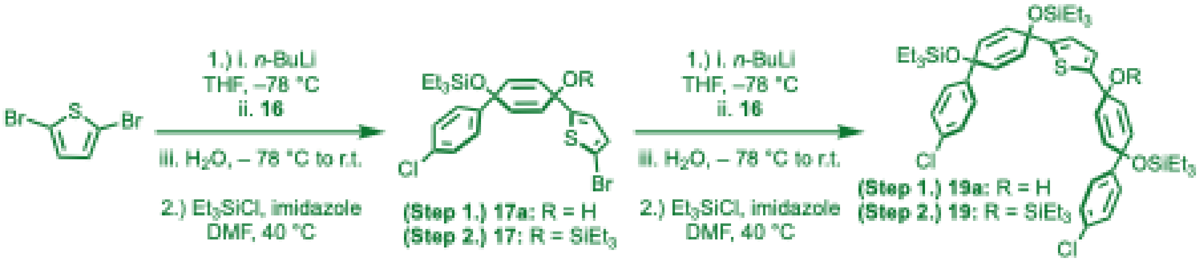

Scheme S5. Synthesis of intermediates **17a**, **17**, **19a**, and **19** (store in freezer for long-term storage).

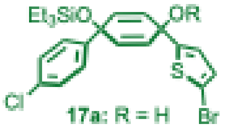

17a. Lithium-halogen exchange: Cooled an oven-dried 250 mL three-necked flask equipped with a stir bar, a glass vacuum adapter, a glass stopper, and a 50 mL addition funnel capped with a glass stopper on high vacuum. Charged the reaction vessel with 2,5-dibromothiophene (5 mL, 0.0444 mol, 1 equiv.) and began freeze, pump, thaw cycles (x 3). Before the starting material could fully thaw on the last cycle, exchanged the glass stopper for a septum under positive nitrogen flow and dissolved the liquid in dry THF (85 mL). Cooled the reaction vessel to –78 ° C over the course of 1.5 hours. Cannula transferred *n*-butyllithium (20 mL, 2.5 M in hexanes, 1.13 equiv.) to the addition funnel and set the reagent to add dropwise. The reaction mixture went from colorless to blue to red. Once all the lithiating agent had been added, the addition funnel was replaced with a rubber septum under positive nitrogen flow, any residual *n*-butyllithium on the funnel was quenched, and the reaction mixture was left to stir for 20 minutes. Then, neat **16** (13.5 mL, 0.0443 mol, 1 equiv.) was added dropwise to the reaction vessel and left to stir for 1 hour before slowly quenching the reaction with DI water (10 mL). The reaction mixture went from red to light yellow. The reaction vessel was brought to r.t. and excess solvent was removed *in vacuo*. The organics were extracted with ethyl acetate (50 mL x 3), subsequently the organics were washed with brine (50 mL x 3), dried over sodium sulfate, gravity filtered, and then solvent was removed *in vacuo*. Residual solvent was removed from the crude oil by gently heating (50 °C water bath) the material with stirring while on high vacuum. **Purification and Characterization:** The material was purified by column chromatography (loaded in hexanes, pH 10 AlO_x, 8 cm tall, 10% DCM:Hex and once impurities had eluted off 30% DCM:Hex to elute off pdt) to yield olivine-colored oil **17a** (9.95 g, 45% yield). ^{1}H NMR (600 MHz, Methylene Chloride-d_2) δ 7.32 (d, *J* = 8.8 Hz, 2H), 7.26 (d, *J* = 8.8 Hz, 2H), 6.93 (d, *J* = 3.8 Hz, 1H), 6.65 (d, *J* = 3.8 Hz, 1H), 6.05 (d, *J* = 10.0 Hz, 2H), 5.98 (d, *J* = 10.0 Hz, 2H), 2.35 (s, 1H), 0.98 (t, *J* = 7.9 Hz, 9H), 0.68 (q, *J* = 7.9, Hz, 6H); ^{13}C NMR (151 MHz, Methylene Chloride-d_2) δ 151.32, 144.36, 133.92, 133.23, 130.16, 130.02, 128.64, 127.45, 124.31, 112.34, 71.54, 68.21, 7.17, 6.74; R_f = 0.25 (AlO_x N, 30% DCM:Hex); IR (ATR) ṽ 730 (m), 1006 (m), 1432 (w), 2910 (w), 3429 (w); HRMS (ESI, negative mode) *m*/*z* calcd for $C_{22}H_{25}BrClO_2SSi$: 495.0216 $[M]^-$, found 495.0206 (-2.0 ppm).

17. Triethylsilyl protection: Flame-dried a 500 mL three-necked flask equipped with a stir bar, a glass vacuum adapter, and two glass stoppers. Transferred **17a** (9.95 g, 0.02 mol, 1 equiv.) dissolved in minimal DCM to the reaction vessel, removed the solvent on high vacuum, and left the oil drying on high vacuum for 1.5 hours. To the reaction vessel added imidazole (5.44 g, 0.08 mol, 4 equiv.) before backfilling and evacuating the system (3 cycles x 3 min). Under positive nitrogen flow exchanged a glass stopper for a septum, then dissolved the starting material in dry DMF (100 mL). Lowered the reaction vessel into a pre-heated oil bath set to 40 °C. Let the reaction

equilibrate to temperature over the course of 10 minutes before adding neat Et_3SiCl (6.88 mL, 0.0410 mol, 2.05 equiv.) dropwise. Allowed the reaction to run overnight. Quenched the reaction mixture with sat. sodium bicarbonate (50 mL) and let the mixture stir for 45 minutes. Extracted the organics with ethyl acetate (30 mL x 3) before the organics were washed with 5% LiCl (30 mL x 5), dried over sodium sulfate, gravity filtered, and then removed solvent *in vacuo*. Residual solvent was removed from the crude oil by gently heating (50 °C water bath) the material with stirring while on high vacuum. **Purification and Characterization:** The material was purified by column chromatography (loaded in pentane, pH 10 AlO_x, 8 cm tall, 100% pentane) to yield opaque light green-colored oil **17** (10.7 g, 87% yield). ^{1}H NMR (600 MHz, Methylene Chloride-d_2) δ 7.28 (d, J = 8.7 Hz, 2H), 7.25 (d, J = 8.7 Hz, 2H), 6.83 (d, J = 3.8 Hz, 1H), 6.36 (d, J = 3.8 Hz, 1H), 6.03 (d, J = 10.1 Hz, 2H), 5.98 (d, J = 10.1 Hz, 2H), 0.94 (dt, J = 15.4, 8.0 Hz, 18H), 0.62 (dq, J = 16.0, 7.9 Hz, 12H); ^{13}C NMR (151 MHz, Methylene Chloride-d_2) δ 153.68, 144.83, 133.30, 132.32, 130.98, 129.87, 128.61, 127.72, 123.57, 111.73, 71.51, 70.21, 7.15, 7.13, 6.71, 6.62; R_f = 0.74 (AlO_x N, 100% pentane); IR (ATR) ṽ 584 (m), 1017 (m), 1447 (w), 2910 (w); HRMS (ESI, positive mode) *m/z* calcd for $C_{28}H_{40}BrClO_2SSi_2$: 610.1159 [M]$^+$, found 610.1149 (-1.6 ppm).

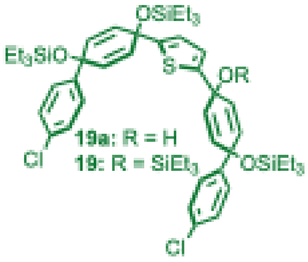

19a. Lithium-halogen exchange: Flame-dried a 50 mL three-necked flask equipped with a stir bar, two glass stoppers, and a glass vacuum adapter. Charged the flask with **17** (4 g, 6.53 mmol, 1 equiv.) before backfilling and evacuating the system (3 cycles x 3 min). Exchanged a glass stopper for a septum under positive nitrogen flow and dissolved the oil in dry THF (13 mL). Sonicated the flask under inert atmosphere if the stir bar could not rotate freely. Cooled the reaction vessel to –78 °C over the span of 15 minutes. To the reaction vessel added *n*-butyllithium (2.75 mL, 2.5 M in hexanes, 1.05 equiv.) dropwise. The reaction mixture went from light green to bright orange. Allowed the reaction mixture to stir for 12 minutes. Added neat **16** (1.99 mL, 6.54 mmol, 1 equiv.) dropwise and slowly to ensure the oil was dissolving instead of freezing. Left the reaction to stir for 1 hour before adding DI water (2 mL). Allowed the reaction mixture to come to room temperature over the course of 45 minutes. Removed excess solvent *in vacuo* before extracting the organics with ethyl acetate (50 mL x 3), then the organics were washed with brine (50 mL x 3), dried over sodium sulfate, gravity filtered, and then the solvent was removed *in vacuo*. Residual solvent was removed from the crude oil by stirring the crude material while on high vacuum. **Purification and Characterization:** The material was purified by column chromatography (loaded in 10% DCM:Hex, pH 10 AlO_x, 8 cm tall, gradient 10% DCM:Hex - 20% DCM:Hex) to yield colorless oil **19a** that slowly turned a light purple (4.99 g, 88% yield). ^{1}H NMR (600 MHz, Methylene Chloride-d_2) δ 7.32 (d, J = 8.6 Hz, 2H), 7.29 (d, J = 8.6 Hz, 2H), 7.23-7.21 (m, 4H), 6.71 (d, J = 3.7 Hz, 2H), 6.55 (d, J = 3.7 Hz), 6.11-6.08 (m, 4H), 5.95 (t, J = 10.2 Hz, 4H), 2.31 (s, 1H), 0.99 (t, J = 7.9 Hz, 9H), 0.95 (t, J = 7.9 Hz, 18H), 0.69 (q, J = 7.9 Hz, 4H), 0.61 (dq, J = 8.0, 4.1 Hz, 12H); ^{13}C NMR (151 MHz, Methylene Chloride-d_2) δ 151.79, 149.05, 144.95, 144.57, 133.37, 133.19, 133.11, 131.97, 131.40, 130.59, 128.52, 127.74, 127.50, 123.49, 123.21, 71.65, 71.61, 69.92, 68.00, 7.20, 7.18, 7.17, 6.74, 6.74, 6.72, 6.65.

19. Triethylsilyl protection: Flame-dried a 100 mL round-bottomed flask equipped with a stir bar. Backfilled and evacuated the system (1 cycle x 3 min) before charging the flask with **19a** (3.07 g, 3.54 mmol, 1 equiv.) dissolved in minimal DCM. Removed the solvent on high vacuum

and left the oil to dry on high vacuum for 30 minutes. To the reaction vessel added imidazole (0.962 g, 0.0141 mol, 4 equiv.) before backfilling and evacuating the system (3 cycles x 3 min). Under positive nitrogen flow exchanged a glass stopper for a septum, then dissolved the starting material in dry DMF (18 mL). Lowered the reaction vessel into a pre-heated oil bath set to 40 °C. Let the reaction equilibrate to temperature over the course of 10 minutes before adding neat Et_3SiCl (0.62 mL, 3.69 mmol, 1.04 equiv.) dropwise. Allowed the reaction to run overnight. Quenched the reaction mixture with sat. sodium bicarbonate (8 mL) and let the mixture stir for 5 minutes. Extracted the organics with ethyl acetate (20 mL x 3) before the organics were washed with 5% LiCl (20 mL x 5), dried over sodium sulfate, gravity filtered, and then removed solvent *in vacuo*. Residual solvent was removed from the crude oil by gently heating (50 °C water bath) the material with stirring while on high vacuum. **Purification and Characterization:** The material was purified by column chromatography to yield light brown oil **19** (3.2 g, 92% yield). ^{1}H NMR (600 MHz, Methylene Chloride-d_2) δ 7.26 (d, J = 8.6 Hz, 4H), 7.18 (d, J = 8.6 Hz, 4H), 6.52 (s, 2H), 6.11 (d, J = 10.0 Hz, 4H), 5.92 (d, J = 10.0 Hz, 4H), 0.97-0.92 (m, 36H), 0.65-0.58 (m, 24H); ^{13}C NMR (151 MHz, Methylene Chloride-d_2) δ 151.16, 144.98, 133.09, 131.87, 131.43, 128.45, 127.69, 123.05, 71.68, 69.76, 7.20, 7.17, 6.71, 6.68.

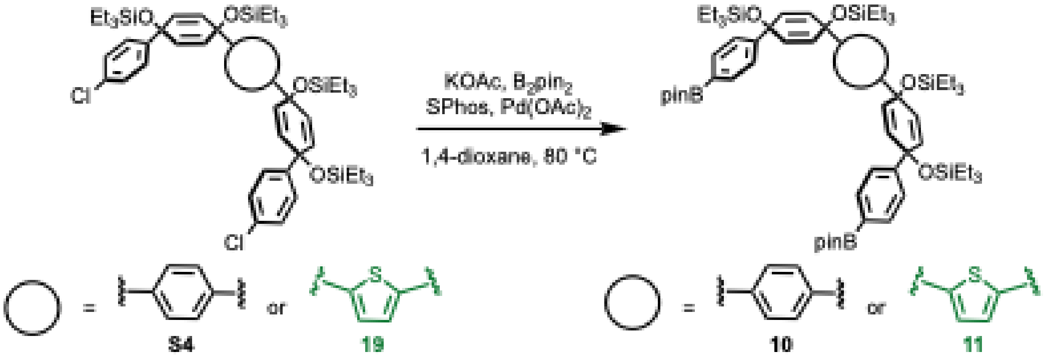

Scheme S6. Synthesis of intermediates **10** and **11** (store **11** in freezer for long-term storage).

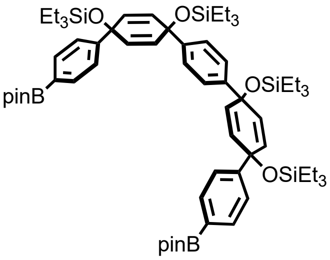

10. A 15 mL two-necked flask equipped with a stir bar and charged with KOAc (0.940 g, 9.57 mmol, 6.7 equiv.) was oven-dried, alongside a glass stopper and glass vacuum adapter, overnight. The reaction vessel was cooled on high vacuum before adding **S4** (1.4 g, 1.43 mmol, 1 equiv.), B_2pin_2 (0.910 g, 3.58 mmol, 2.5 equiv.), SPhos (0.074 g, 0.180 mmol, 0.125 equiv.), and $Pd(OAc)_2$ (4.8 mg, 0.0214 mmol, 0.0150 equiv.). Backfilled and evacuated the system (3 cycles x 4 min) before exchanging the glass stopper for a septum under positive nitrogen flow. The solids were dissolved in dry 1,4-dioxane (4.80 mL) before the reaction vessel was lowered into a pre-heated oil bath set to 80 °C and left to run overnight. An aliquot was taken to confirm all **S4** had been consumed (checked by TLC: SiO_2, 20% DCM:Hex). Brought the reaction mixture to r.t. before extracting the organics with DCM (20 mL x 3). Washed the organics with brine (20 mL x 3), dried over sodium sulfate, gravity filtered and then removed the solvent *in vacuo*. To remove residual solvent the crude material was placed on high vacuum for 30 minutes, redissolved in acetone before removing solvent *in vacuo*, then placed back on high vacuum for

30 minutes (x2). **Purification:** The off-white material was triturated in EtOH:MeOH (~50:50) to remove excess B_2pin_2 and to afford shiny, off-white solid **10** (1.19 g, 72% yield). The ^1H NMR (500 MHz, Chloroform-*d*) matched previous literature reports.

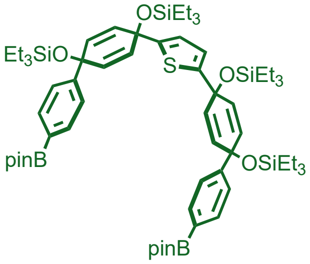

11. Flame-dried a 15 mL two-necked equipped with a stir bar, a glass stopper, and a glass vacuum adapter. Once cool, charged the flask with KOAc (0.363 g, 3.70 mmol, 6.6 equiv.) before flame-drying the flask a second time. To the flask was added **19** (0.550 g, 0.560 mmol, 1 equiv.), B_2pin_2 (0.291 g, 1.15 mmol, 2.05 equiv.), SPhos (0.029 g, 0.0706 mmol, 0.126 equiv.), and $Pd(OAc)_2$ (1.89 mg, 8.91 µmol, 0.0159 equiv.). Backfilled and evacuated the system (3 cycles x 3 min) before exchanging the glass stopper for a septum under positive nitrogen flow. The solids were dissolved in dry 1,4-dioxane (4 mL) before the reaction vessel was lowered into a pre-heated oil bath set to 80 °C and left to run overnight. An aliquot was taken to confirm all **19** had been consumed (checked by TLC: AlO_x N, 10% DCM:Hex). Brought the reaction mixture to r.t. before extracting the organics with ethyl acetate (15 mL x 3). Washed the organics with 5% LiCl (15 mL x 4), dried over sodium sulfate, gravity filtered and then removed the solvent *in vacuo*. To remove residual solvent the crude material was placed on high vacuum for 30 minutes, redissolved in acetone before removing solvent *in vacuo*, then placed back on high vacuum for 30 minutes (x 3). **Purification and Characterization:** The off-white material was triturated in EtOH to remove excess B_2pin_2 and small impurities to afford white solid **11** (0.497 g, 76% yield). ^1H NMR (600 MHz, Methylene Chloride-d_2) δ 7.62 (d, *J* = 8.2 Hz, 4H), 7.33 (d, *J* = 8.2 Hz, 4H), 6.50 (s, 2H), 6.08 (d, *J* = 10.0 Hz, 4H), 5.95 (d, *J* = 10.1 Hz, 4H), 1.30 (s, 24H), 0.94 (t, *J* = 7.7 Hz, 36H), 0.62 (p, *J* = 7.9 Hz, 24H); ^{13}C NMR (151 MHz, Methylene Chloride-d_2) δ 151.18, 149.30, 134.89, 131.93, 131.29, 125.47, 123.01, 84.06, 72.05, 69.92, 25.04, 7.21, 7.18, 6.74, 6.67; IR (KBr) ṽ 727 (m), 1089 (m), 1361 (m), 2875 (w), 2956 (w); HRMS (MALDI) *m*/*z* calcd for $C_{64}H_{102}B_2O_8SSi_4$: 1164.6559 [M]+, found 1164.6553 (1.6 ppm).

Synthesis of Seven Ring Containing Intermediates

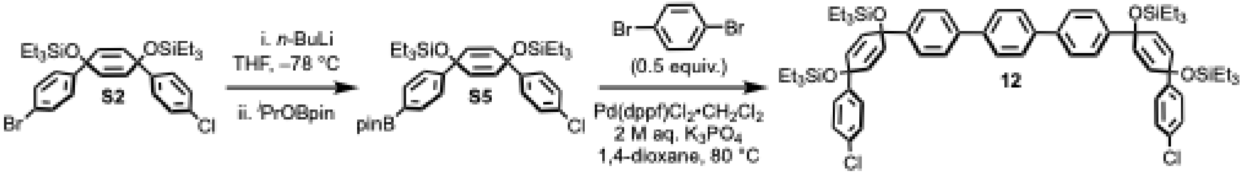

Scheme S7. Synthesis of intermediate **S5** and **12**.

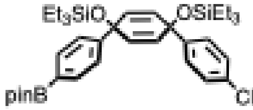

S5. Cooled an overnight, oven-dried 100 mL three-necked flask equipped with a stir bar, two glass stoppers, and a glass vacuum adapter on high vacuum. Charged the flask with **S2** (5 g, 8.25 mmol, 1 equiv.) before backfilling and evacuating the system (3 cycles x 3 min). Exchanged a glass stopper for a septum under positive nitrogen flow before dissolving the solid in dry THF (41.2 mL). Cooled the flask to –78 ·C over the course of 45 minutes. To the reaction vessel added *n*-butyllithium (3.46 mL, 2.5 M in hexanes, 1.05 equiv.) dropwise, left the reaction to stir for 10 minutes, and then added ^{i}PrOBpin (1.77 mL, 8.68 mmol, 1.05 equiv.) dropwise. Allowed the reaction to stir for 1 hour before quenching the reaction mixture with DI water (8 mL). Let come to room temperature before excess solvent was removed *in vacuo* before extracting the organics with ethyl acetate (30 mL x 3). Washed the organics with brine (30 mL x 3), dried over sodium sulfate, gravity filtered and then removed the solvent *in vacuo*. To remove residual solvent the crude material was placed on high vacuum for 30 minutes, redissolved in acetone before removing solvent *in vacuo*, then placed back on high vacuum for 30 minutes (x2). **Purification:** The crude oil was triturated in EtOH to yield off-white solid **S5** (3.36 g, 62% yield). The ^{1}H NMR (500 MHz, Chloroform-*d*) matched previous literature reports.

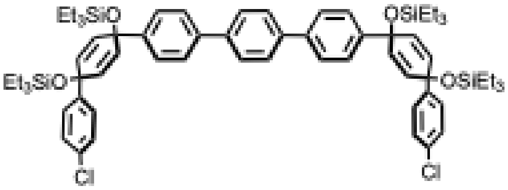

12. Flame dried a 50 mL three-necked flask equipped with a stir bar, two glass stoppers, and a glass vacuum adapter. Charged the flask with 1,4-dibromobenzene (0.155, 0.657 mmol, 1 equiv.), **S5** (0.900 g, 1.38 mmol, 2.10 equiv.), and Pd(dppf)Cl_2 (0.027 g, 0.033 mmol, 0.05 equiv.) under positive nitrogen flow. Backfilled and evacuated the system with the glass vacuum adapter (3 cycles x 3 min) before dissolving the solids in dry 1,4-dioxane (13.78 mL). The reaction vessel was lowered into a pre-heated oil bath set to 80 °C, the flask was left to equilibrate to temperature over the course of 10 minutes, and then sparged aq. K_3PO_4 (1.38 mL, 2 M) was added dropwise before the reaction was left to run overnight. The reaction mixture was brought to r.t. before extracting the organics with ethyl acetate (20 mL x 3). Washed the organics with brine (20 mL x 3), dried over sodium sulfate, gravity filtered and then removed the solvent *in vacuo*. **Purification:** The material was purified by column chromatography (loaded in 5% DCM:Hex, SiO_2, gradient 10% DCM:Hex - 20% DCM:Hex) to yield white solid **12** (0.411 g, 56% yield). ^{1}H NMR (600 MHz, Methylene Chloride-d_2) δ 7.68 (s, 4H), 7.58 (d, J = 8.5 Hz, 4H), 7.41 (d, J = 8.4 Hz, 4H), 7.32 (d, J = 8.7 Hz, 4H), 7.25 (d, J = 8.6 Hz, 4H), 6.07 (d, J = 10.1 Hz, 4H), 6.00 (d, J = 10.2 Hz, 4H), 0.95 (td, J = 7.9, 5.3 Hz, 36H), 0.64 (td, J = 7.9, 5.6 Hz, 24H); ^{13}C NMR (151 MHz, Methylene Chloride-d_2) δ 145.51, 145.24, 139.91, 139.79, 133.20, 132.21, 131.62, 128.56, 127.85, 127.68, 127.03,

126.75, 71.69, 71.56, 7.22, 7.20, 6.78, 6.75; R_f = 0.26 (SiO_2, 20% DCM:Hex); HRMS (TOF MS AP+) *m/z* calcd for $C_{66}H_{88}Cl_2O_4Si_4$: 1126.5137 [M]+, found 1126.5134 (-0.3 ppm).

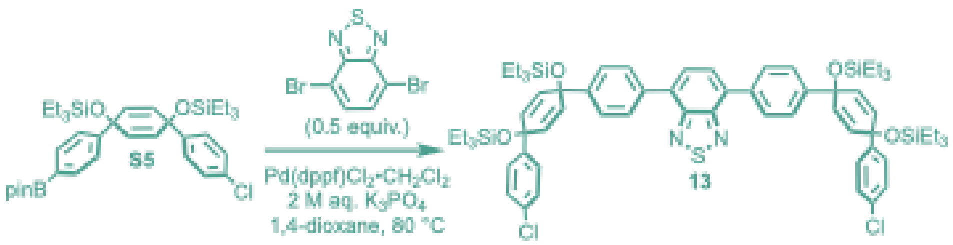

Scheme S8. Synthesis of intermediate **13**.

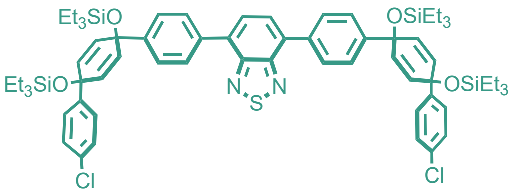

13. Flame dried a 25 mL Schlenk flask equipped with a stir bar and glass stopper. Charged the flask with 4,7-dibromo-2,1,3-benzothiadiazole (0.220 g, 0.748 mmol, 1 equiv.), **S5** (1.00 g, 1.53 mmol, 2.05 equiv.), and Pd(dppf)Cl_2 (0.030 g, 0.037 mmol, 0.049 equiv.), under positive nitrogen flow. Backfilled and evacuated the system (3 cycles x 3 min) before dissolving the solids in dry 1,4-dioxane (15.3 mL). The reaction vessel was lowered into a pre-heated oil bath set to 80 °C, the flask was left to equilibrate to temperature over the course of 10 minutes, and then sparged aq. K_3PO_4 (1.53 mL, 2 M) was added dropwise before the reaction was left to run overnight. An aliquot was taken to confirm **13** was the predominant species (checked by TLC: SiO_2, 20% DCM:Hex). The reaction mixture was brought to r.t. before excess solvent was removed *in vacuo*. The crude was redissolved in ethyl acetate before extracting the organics with ethyl acetate (50 mL x 3). Washed the organics with brine (40 mL x 3), dried over sodium sulfate, gravity filtered and then removed the solvent *in vacuo*. To remove residual solvent the crude material was placed on high vacuum for 30 minutes, redissolved in acetone before removing solvent *in vacuo*, then placed back on high vacuum for 30 minutes (x2). **Purification and Characterization:** The material was purified by column chromatography (dry loaded, SiO_2, gradient 100% Hex - 40% DCM:Hex) to yield fluffy, neon green solid **12** (0.710 g, 39% yield). ^{1}H NMR (600 MHz, Methylene Chloride-d_2) δ 7.91 (d, *J* = 8.5 Hz, 4H), 7.80 (s, 2H), 7.48 (d, *J* = 8.5 Hz, 4H), 7.36 (d, *J* = 8.6 Hz, 4H), 7.27 (d, *J* = 8.6 Hz, 4H), 6.10 (d, *J* = 10.2 Hz, 4H), 6.05 (d, *J* = 10.2 Hz, 4H), 1.02 - 0.93 (m, 36H), 0.70 - 0.61 (m, 24H); ^{13}C NMR (151 MHz, Methylene Chloride-d_2) δ 154.53, 146.44, 145.28, 136.81, 133.29, 133.18, 132.27, 131.71, 129.50, 128.63, 128.41, 127.97, 126.39, 71.85, 71.56, 7.25, 7.21, 6.84, 6.80; R_f = 0.44 (SiO_2, 20% DCM:Hex); IR (ATR) ṽ 2917 (w), 1072 (m), 709 (m); HRMS (MALDI) *m/z* calcd for $C_{66}H_{87}Cl_2N_2O_4SSi_4$: 1,184.4762 [M]+, found 1,185.4835 (0.5841 ppm).

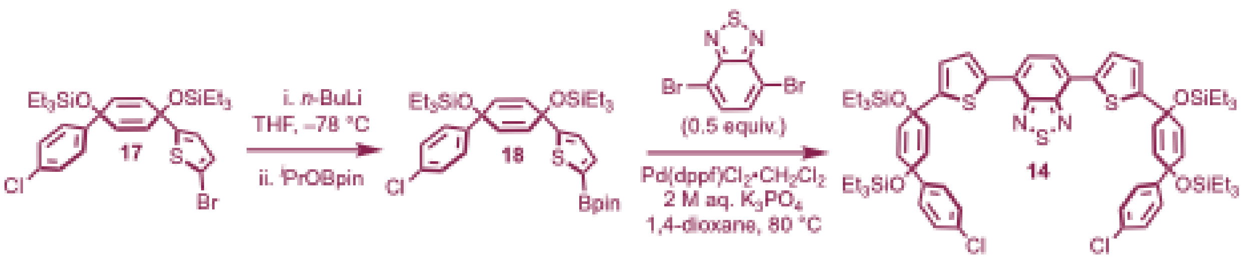

Scheme S9. Synthesis of intermediates **18** and **14** (store **18** and **14** in freezer for long-term storage).

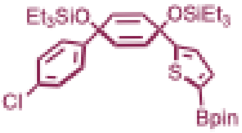

18. Flame dried a 100 mL three-necked flask equipped with a stir bar, two glass stoppers, and a glass vacuum adapter. Once cool exchanged a glass stopper for a septum under positive nitrogen flow, then exchanged the glass vacuum adapter for a septum under positive nitrogen flow coming from a needle inserted in the first septa. Pre-weighed the flask using a scale inside the fume hood. Kept the flask under positive nitrogen flow to transfer **17** (3.2 g, 5.23 mmol, 1 equiv.) using a Pasteur pipette, took off the Schlenk line to weigh, and repeated this process until the desired amount was in the flask. Backfilled and evacuated the neat oil (3 cycles x 3 min) before dissolving the oil in dry THF (42 mL). Cooled the flask to –78 °C over the course of 45 minutes. To the reaction vessel added *n*-butyllithium (2.20 mL, 2.5 M in hexanes, 1.05 equiv.) dropwise, left the reaction to stir for 15 minutes, and then added ^{i}PrOBpin (1.12 mL, 5.49 mmol, 1.05 equiv.) dropwise. Allowed the reaction to stir for 1 hour before quenching the reaction mixture with DI water (10 mL). Let come to room temperature before excess solvent was removed *in vacuo* before extracting the organics with ethyl acetate (40 mL x 3). Washed the organics with brine (40 mL x 3), dried over sodium sulfate, gravity filtered and then removed the solvent *in vacuo*. **Purification and Characterization:** The material was either pushed forward without further purification or purified via minimal sonication in acetonitrile to give a light brown filtrate that was concentrated down to give light brown oil **18** (2.48 g, 72% yield). ^{1}H NMR (600 MHz, Methylene Chloride-*d*$_2$) δ 7.36 (d, *J* = 3.5 Hz, 1H), 7.27 (d, *J* = 8.7 Hz, 2H), 7.22 (d, *J* = 8.6 Hz, 2H), 6.75 (d, *J* = 3.5 Hz, 1H), 6.11 (d, *J* = 10.0 Hz, 2H), 5.95 (d, *J* = 10.0 Hz, 2H), 1.32 (s, 12H), 0.94 (td, *J* = 8.0, 1.9 Hz, 18H), 0.62 (p, *J* = 7.8 Hz, 12H); ^{13}C NMR (151 MHz, Methylene Chloride-*d*$_2$) δ 159.03, 144.96, 137.19, 133.21, 131.95, 131.50, 128.55, 127.75, 125.11, 84.49, 71.63, 69.99, 24.97, 7.19, 7.17, 6.75, 6.66; IR (ATR) ṽ 2914 (w), 1072 (m), 719 (m); HRMS (ESI, positive mode) *m*/*z* calcd for $C_{34}H_{52}BClO_4SSi_2$: 658.2906 [M]+, found 658.2906 (-0.5 ppm).

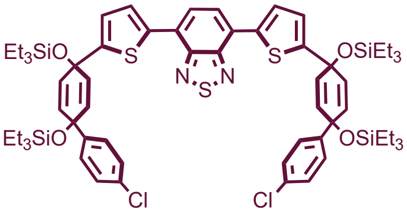

14. Cooled an overnight oven dried 50 mL three-necked flask equipped with a stir bar, two glass stoppers, and a glass vacuum adapter before charging the flask with 4,7-dibromo-2,1,3-benzothiadiazole (1.5 g, 5.10 mmol, 1 equiv.), **18** (6.73 g, 0.010 mol, 2.00 equiv.), and Pd(dppf)Cl_2•CH_2Cl_2 (0.187 g, 0.228 mmol, 0.045 equiv.), under positive nitrogen flow. Backfilled and evacuated the system (3 cycles x 3 min) before dissolving the solids in dry 1,4-dioxane (17 mL). The reaction vessel was lowered into a pre-heated oil bath set to 80 °C, the flask was left to

equilibrate to temperature over the course of 10 minutes, and then sparged aq. K_3PO_4 (5.10 mL, 2 M) was added dropwise before the reaction was left to run overnight. An aliquot was taken to confirm all the starting material had been consumed (checked by ^{1}H NMR). The reaction mixture was brought to r.t. before excess solvent was removed *in vacuo*. The crude was redissolved in ethyl acetate before extracting the organics with ethyl acetate (80 mL x 3). Washed the organics with brine (80 mL x 2), dried over sodium sulfate, gravity filtered and then removed the solvent *in vacuo*. To remove residual solvent the crude material was placed on high vacuum for 30 minutes, redissolved in acetone before removing solvent *in vacuo*, then placed back on high vacuum for 30 minutes (x2). **Purification and Characterization:** The material was purified by automated flash column chromatography (loaded in hexanes, pH 10 AlO_x, gradient 100% Hex - 15% DCM:Hex) to yield fluffy, cherry red solid **14** (1.75 g, 29% yield). ^{1}H NMR (500 MHz, Chloroform-*d*) δ 7.96 (d, *J* = 3.8 Hz, 2H), 7.81 (s, 2H), 7.33 (d, *J* = 8.6 Hz, 4H), 7.24 (d, *J* = 8.6 Hz, 4H), 6.79 (d, *J* = 3.8 Hz, 2H), 6.17 (d, *J* = 10.1 Hz, 4H), 5.98 (d, *J* = 10.0 Hz, 4H), 0.97 (dt, *J* = 10.7, 7.9 Hz, 36H), 0.70 - 0.61 (m, 24H); ^{13}C NMR (126 MHz, Methylene Chloride-d_2) δ 153.26, 152.99, 144.98, 139.18, 133.25, 132.16, 131.38, 128.60, 127.84, 127.69, 126.28, 125.82, 124.68, 71.67, 70.13, 7.22, 7.21, 6.77, 6.75; R_f = 0.44 (AlO_x N, 20% DCM:Hex); HRMS (MALDI) *m/z* calcd for $C_{62}H_{82}Cl_2N_2O_4S_3Si_4$: 1,196.3891 [M]+, found 1,196.3885 (1.5227 ppm).

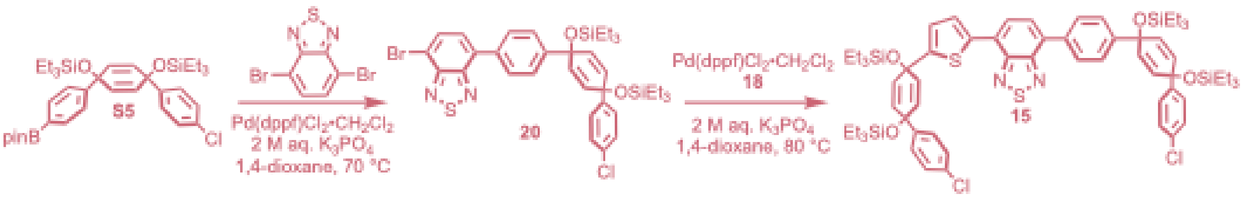

Scheme S10. Synthesis of intermediates **20** and **15** (store **20** and **15** in freezer for long-term storage).

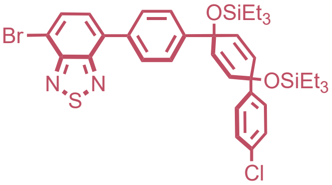

20. Placed a 50 mL Schlenk flask, a stir bar, and a glass stopper in the oven for a minimum of 3 hours before cooling on high vacuum. Charged the flask with 4,7-dibromo-2,1,3-benzothiadiazole (1.4 g, 4.76 mmol, 1 equiv.), **S5** (3.11 g, 4.76 mmol, 1 equiv.), and $Pd(dppf)Cl_2•CH_2Cl_2$ (0.300 g, 0.368 mmol, 0.077 equiv.), under positive nitrogen flow. Backfilled and evacuated the system (3 cycles x 3 min) before dissolving the solids in dry 1,4-dioxane (16 mL). The reaction vessel was lowered into a pre-heated oil bath set to 70 °C, the flask was left to equilibrate to temperature over the course of 10 minutes, and then sparged aq. K_3PO_4 (4.76 mL, 2 M) was added dropwise before the reaction was left to run overnight. An aliquot was taken to confirm all **S5** starting material had been consumed (checked by ^{1}H NMR). The reaction mixture was brought to r.t. before excess solvent was removed *in vacuo*. The crude was redissolved in ethyl acetate before extracting the organics with ethyl acetate (50 mL x 3). Washed the organics with brine (50 mL x 2), dried over sodium sulfate, gravity filtered and then removed the solvent *in vacuo*. To remove residual solvent the crude material was placed on high vacuum for 30 minutes, redissolved in acetone before removing solvent *in vacuo*, then placed back on high vacuum for 30 minutes (x2). **Purification and Characterization:** The material was purified by automated flash column chromatography (loaded in hexanes, SiO_2, gradient 100% Hex - 18% DCM:Hex) to yield sticky, yellow-green oil **20** (0.933 g, 26% yield). ^{1}H NMR (600 MHz, Methylene Chloride-d_2) δ 7.93 (d, *J* = 7.6 Hz, 1H), 7.83 (d, *J* = 8.4 Hz, 2H), 7.59 (d, *J* = 7.5 Hz, 1H), 7.47 (d, *J* = 8.4 Hz, 2H), 7.34 (d, *J* = 8.7 Hz,

2H), 7.26 (d, J = 8.6 Hz, 2H), 6.10 - 6.02 (m, 4H), 0.96 (dt, J = 15.6, 7.9 Hz, 18H), 0.65 (dq, J = 24.0, 7.9 Hz, 12H); ^{13}C NMR (151 MHz, Methylene Chloride-d_2) δ 154.31, 153.60, 146.83, 145.24, 136.04, 133.95, 133.31, 132.70 ,132.18, 131.77, 129.46, 128.63, 128.58, 127.92, 126.47, 113.34, 71.83, 71.52, 7.24, 7.20, 6.82, 6.80; R_f = 0.52 (SiO_2, 40% DCM:Hex); IR (ATR) ṽ 2910 (w), 1069 (m), 715 (m); HRMS (ESI, positive mode) *m*/*z* calcd for $C_{36}H_{44}BrClN_2O_2SSi_2$: 738.1534 [M]+, found 738.1530 (-0.5 ppm).

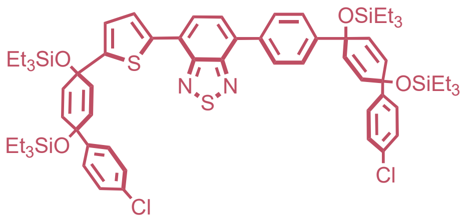

15. Cooled an oven dried 5 mL two-necked flask equipped with a stir bar, a glass stopper, and a glass vacuum adapter on high vacuum. Charged the flask with **20** (0.4 g, 0.540 mmol, 1 equiv.) dissolved in DCM and removed the solvent *in vacuo* before leaving the oil to dry on high vacuum for 1 hour. Charged the flask with **18** (0.356 g, 0.540 mmol, 1 equiv.) and Pd(dppf)Cl_2•CH_2Cl_2 (0.04 g, 0.048 mmol, 0.090 equiv.), under positive nitrogen flow. Backfilled and evacuated the system (3 cycles x 4 min) before dissolving the solids in dry 1,4-dioxane (4 mL). The reaction vessel was lowered into a pre-heated oil bath set to 80 °C, the flask was left to equilibrate to temperature over the course of 10 minutes, and then sparged aq. K_3PO_4 (0.54 mL, 2 M) was added dropwise before the reaction was left to run overnight. An aliquot was taken to confirm all **20** starting material had been consumed (checked by TLC: AlO_x N, 20% DCM:Hex). The reaction mixture was brought to r.t. before extracting the organics with ethyl acetate (20 mL x 3). Washed the organics with brine (20 mL x 2), dried over sodium sulfate, gravity filtered and then removed the solvent *in vacuo*. To remove residual solvent the crude material was placed on high vacuum for 30 minutes, redissolved in acetone before removing solvent *in vacuo*, then placed back on high vacuum for 30 minutes (x 3). **Purification and Characterization:** The material was purified by automated flash column chromatography (loaded in 2% DCM:Hex, SiO_2, gradient 2% DCM:Hex - 20% DCM:Hex) to yield fluffy, tangerine-orange solid **15** (0.933 g, 26% yield). ^{1}H NMR (600 MHz, Methylene Chloride-d_2) δ 7.97 (d, J = 3.8 Hz, 1H), 7.91, (dd, J = 7.9, 4.6 Hz, 3H), 7.74 (d, J = 7.5 Hz, 1H), 7.48 (d, J = 8.4 Hz, 2H), 7.36 (dd, J = 8.6, 3.3 Hz, 4H), 7.26 (t, J = 8.3 Hz, 4H), 6.83 (d, J = 3.8 Hz, 1H), 6.20 (d, J = 10.0 Hz, 2H), 6.11 - 6.00 (m, 6H), 1.01 - 0.93 (m, 36H), 0.71 - 0.61 (m, 24H); ^{13}C NMR (151 MHz, Methylene Chloride-d_2) δ 154.42, 153.24, 153.15, 146.45, 145.28, 144.99, 139.20, 136.67, 133.30, 133.25, 132.66, 132.26, 132.17, 131.72, 131.40, 129.45, 128.63, 128.60, 128.37, 127.94, 127.85, 127.77, 126.80, 126.40, 125.91, 124.66, 71.85, 71.68, 71.56, 70.13, 7.26, 7.21, 6.84, 6.81, 6.78, 6.76; R_f = 0.5 (AlO_x N, 20% DCM:Hex); IR (ATR) ṽ 2909 (w), 1080 (m), 723 (m); HRMS (MALDI) *m*/*z* calcd for $C_{64}H_{84}Cl_2N_2O_4S_2Si_4$: 1,190.4327 [M]+, found 1,190.4321 (2.5106 ppm).

b. **D-A[12]CPP Derivatives: BT[12]CPP (1), Thio[12]CPP (3), BT-2-thio[12]CPP (4), and BT-7-thio[12]CPP (5)**

General Method to Prepare Tin(II) Chloride Dihydrochloride (H_2SnCl_4) Solution: Flame dried a 25 mL Schlenk flask equipped with a stir bar and a glass stopper. Once cool charged the flask with $SnCl_2$ • 2 H_2O (0.181 g, 0.802 mmol, 1 equiv.) before backfilling and evacuating the flask (3 cycles x 3 min). Dissolved the solid in dry THF (20 mL) before adding concentrated HCl (0.13 mL, 12 M) dropwise. Allowed the reaction to stir for at least one hour before use.

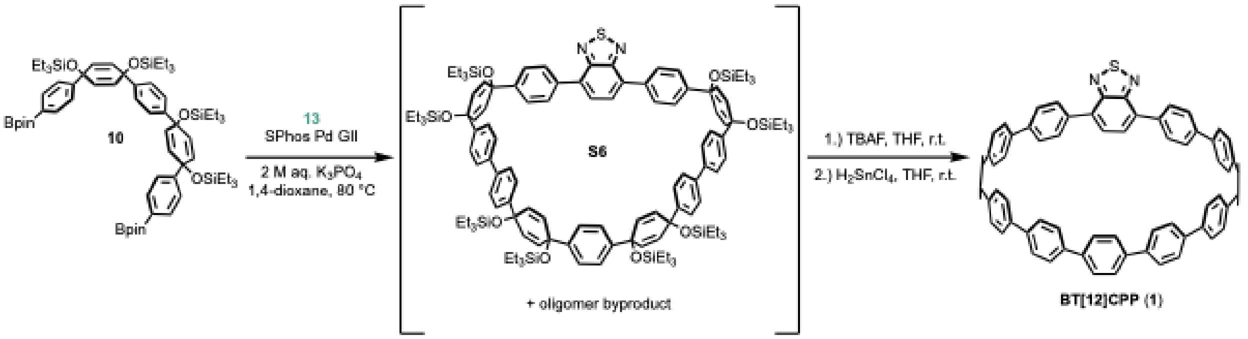

Scheme S11. Synthesis of non-isolated intermediate **S6** and target compound **1** (store **S6** and **1** in freezer for long term storage).

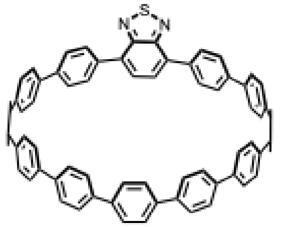

S6. Macrocyclization: Flame dried a 250 mL three-necked flask equipped with a stir bar, two glass stoppers, and a glass vacuum adapter. Once cool, charged the flask with **13** (0.10 g, 0.084 mmol, 1 equiv.), **10** (0.103 g, 0.089 mmol, 1.05 equiv.), and SPhos Pd GII (6.0 mg, 8.3 μmol, 0.1 equiv.). Backfilled and evacuated the system (3 cycles x 3 min) before dissolving the solid in dry 1,4-dioxane (87 mL). Lowered the reaction vessel into a pre-heated oil bath set to 80 °C, let the reaction equilibrate to temperature over the course of 15 minutes before adding sparged, aq. K_3PO_4 (4.21 mL, 2 M). Left the reaction to run overnight. Took an aliquot of the reaction mixture (checked by TLC: SiO_2, 30% DCM:Hex) to reveal **13** starting material was still present. Brought the flask to room temperature and sparged the mixture with nitrogen for 10 minutes before adding **10** (0.103 g, 0.089 mmol, 1.05 equiv.) and SPhos Pd GII (6.0 mg, 8.3 μmol, 0.1 equiv.). Sparged the reaction mixture with nitrogen for another 30 minutes before allowing it to run for another 3 hours. A second aliquot of the reaction mixture (checked by TLC: SiO_2, 30% DCM:Hex) showed all **13** had been consumed. The reaction was worked up the next day by bringing the flask to room temperature before removing excess solvent *in vacuo*. The organics were extracted with ethyl acetate (10 mL x 3). The organics were washed with brine (10 mL x 2), dried over sodium sulfate, gravity filtered, and excess solvent was removed *in vacuo*. The material was partially purified by column chromatography (loaded in hexanes, SiO_2, gradient 100% Hex - 20% DCM:Hex) to yield a sticky, green oil that was pushed forward without further purification.

1.Triethylsilyl deprotection and reductive aromatization: Using a syringe and needle, backfilled and evacuated (3 cycles x 3 min) a septum capped, pre-weighed, 20 mL scintillation vial containing a stir bar and partially pure **S6** (~0.191 g, 0.094 mmol, 1 equiv.). Dissolved the

material in dry THF (11.8 mL). To the flask added TBAF (0.76 mL, 1 M in THF) dropwise and left the reaction to run for 1 hour before quenching the reaction mixture with DI water (5 mL). Removed organic solvent *in vacuo*, collected the precipitated solid by vacuum filtration, and redissolved remaining solid in acetone (x 3). The crude solid was left out at room temperature, overnight, to dry. No further purification was attempted.

Using a syringe and needle, backfilled and evacuated (3 cycles x 3 min) a septum capped, pre-weighed, 20 mL scintillation vial containing a stir bar and the crude solid (~0.118 g, 0.107 mmol, 1 equiv.) from the previous step. Dissolved the solid in dry THF (9 mL) before adding a H_2SnCl_4 solution (11 mL, 0.04 M in THF) dropwise. Let the reaction run for 1 hour before adding DI water (5 mL) and extracting the organics with DCM (15 mL x 3). Washed the organics with brine (15 mL x 3), dried over sodium sulfate, gravity filtered the mixture, and then removed the solvent *in vacuo*. To remove residual solvent the crude material was placed on high vacuum for 30 minutes, redissolved in acetone before removing solvent *in vacuo*, then placed back on high vacuum for 30 minutes (x 3). **Purification and Characterization:** The material was purified by column chromatography (loaded in 10% DCM:Hex, pH 10 AlO_x, gradient 10% DCM:Hex - 50% DCM:Hex) and residual grease was removed via trituration in diethyl ether to yield yellow solid **1** (2.5 mg, 2% yield). ^{1}H NMR (600 MHz, Methylene Chloride-*d₂*) δ 8.02 (d, *J* = 8.7 Hz, 4H), 7.70 (dd, *J* = 8.8, 2.6 Hz, 8H), 7.65 - 7.64 (m, 32H), 7.57 (s, 2H); ^{13}C NMR (151 MHz, Methylene Chloride-d_2) δ 154.65, 140.14, 139.17, 138.92, 138.88, 138.83, 138.62, 136.80, 131.68, 130.57, 128.81, 127.77, 127.74, 127.70, 127.67, 127.65, 127.39; R_f = 0.41 (AlO_x N, 50% DCM:Hex); HRMS (ESI, positive mode) *m*/*z* calcd for $C_{72}H_{47}N_2S$: 971.3460 [M+H]+, found 971.3459 (-0.2 ppm).

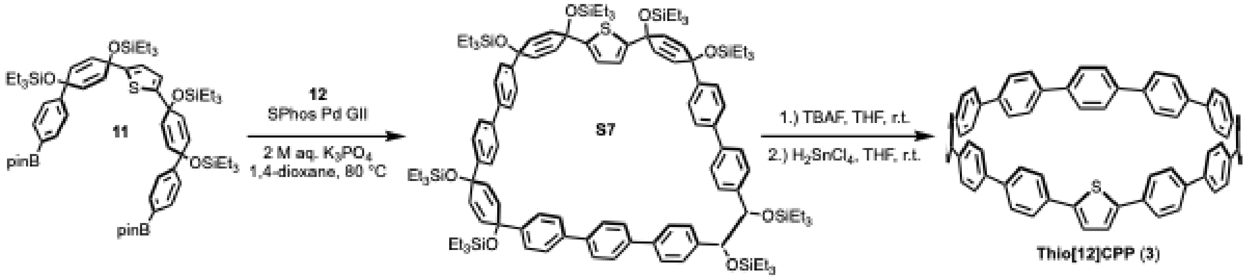

Scheme S12. Synthesis of intermediate **S7** and target compound **3** (store **S7** and **3** in freezer for long term storage)..

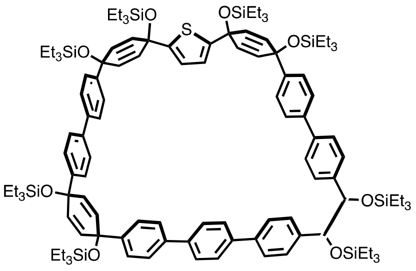

S7. Macrocyclization: Flame dried a 250 mL three-necked flask equipped with a stir bar, two glass stoppers, and a glass vacuum adapter. Charged the flask with **11** (0.126 g, 0.108 mmol, 1.05 equiv.), **12** (0.116 g, 0.103 mmol, 1 equiv.), and SPhos Pd GII (7.0 mg, 1.03 μmol, 0.1 equiv.) before backfilling and evacuated the system (3 cycles x 3 min). Exchanged a glass stopper for a septum under positive nitrogen flow before the solids were dissolved in dry 1,4-dioxane (102.8 mL). The reaction mixture was sparged with nitrogen for an hour. The flask was lowered into a pre-heated oil bath set to 80 °C and left to equilibrate for 30 minutes. Sparged, aq. K_3PO_4 (5.14 mL, 2 M) was added dropwise into the reaction vessel, which was then stirred overnight. An

aliquot of the reaction mixture was taken to monitor the consumption of starting material (checked by TLC: AlO_x N, 15% DCM:Hex). The reaction mixture was brought to room temperature before removing excess solvent *in vacuo*. The organics were extracted with ethyl acetate (20 mL x 3). The organics were washed with brine (20 mL x 2), dried over sodium sulfate, gravity filtered, and excess solvent was removed *in vacuo*. **Purification and Characterization:** The material was purified by column chromatography (loaded in hexanes, pH 10 AlO_x, gradient 2% Hex - 24% DCM:Hex), and triturated in EtOH, then pentane to give white solid **S7** (0.55 mg, 28% yield). ^1H NMR (600 MHz, Methylene Chloride-d_2) δ 7.58 (s, 4H), 7.47 - 7.43 (m, 12H), 7.40 (d, J = 8.4 Hz, 4H), 7.33 (dd, J = 8.4, 6.6 Hz, 8H), 6.48 (s, 2H), 6.13 (dd, J = 18.8, 10.1 Hz, 8H), 6.04 (d, J = 10.1 Hz, 4H), 5.94 (d, J = 10.1 Hz, 4H), 1.00 - 0.90 (m, 72H), 0.70 - 0.56 (m, 48H); ^{13}C NMR (151 MHz, Methylene Chloride-d_2) δ 152.19, 145.42, 145.27, 145.18, 140.28, 139.92, 139.87, 139.68, 132.39 (broad), 132.29, 131.87, 130.80 (broad), 127.60, 127.13, 126.94, 126.81, 126.77, 126.41, 122.25, 72.21, 71.95, 71.84, 69.67, 7.24, 7.20, 7.17, 6.80, 6.80, 6.73, 6.63; R_f = 0.47 (AlO_x N, 15% DCM:Hex); IR (KBr) ṽ 2957 (m), 2874 (m), 1080 (s), 1005 (m), 962 (m), 815 (m), 723 (s); HRMS (MALDI) *m/z* calcd for $C_{118}H_{166}O_8SSi_8$: 1,967.0458 [M]+, found 1,967.0452 (3.8475 ppm).

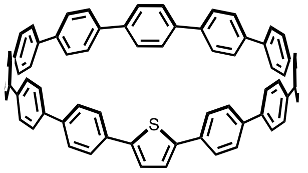

3. Triethylsilyl deprotection and reductive aromatization: Using a syringe and needle, backfilled and evacuated (3 cycles x 3 min) a septum capped, pre-weighed, 20 mL scintillation vial containing a stir bar and partially pure **S7** 54.8 mg, 0.028 mmol, 1 equiv.). Dissolved the material in dry THF (3.48 mL). To the flask added TBAF (0.24 mL, 1 M in THF) dropwise and left the reaction to run for 30 minutes. Transferred the contents to a round bottom flask before quenching the reaction mixture with DI water (10 mL). Removed organic solvent *in vacuo*, collected the precipitated solid by vacuum filtration, and redissolved remaining solid in acetone (x 2). The crude solid was stored in the fridge until further use - no further purification was attempted.

Using a syringe and needle, backfilled and evacuated (3 cycles x 3 min) a septum capped, pre-weighed, 20 mL scintillation vial containing a stir bar and the crude solid (0.028 g, 0.03 mmol, 1 equiv.) from the previous step. Dissolved the solid in dry THF (7.5 mL) before adding a H_2SnCl_4 solution (4.5 mL, 0.04 M in THF) dropwise. Let the reaction run for 1 hour before removing excess solvent *in vacuo*. The organics were extracted with DCM (10 mL x 3). Washed the organics with brine (10 mL x 3), dried over sodium sulfate, gravity filtered the mixture, and then removed the solvent *in vacuo*. **Purification and Characterization:** The material was purified by column chromatography (loaded in hexanes, pH 10 AlO_x, gradient 0% Hex - 30% DCM:Hex) and triturated in pentane to remove excess grease to yield light yellow solid **3** (1.9 mg, 7% yield). ^1H NMR (600 MHz, Methylene Chloride-d_2) δ 7.66 - 7.62 (m, 36H), 7.59 (d, J = 8.6 Hz, 4H), 7.51 (d, J = 8.6 Hz, 4H), 7.27 (s, 2H); ^{13}C NMR (151 MHz, Methylene Chloride-d_2) δ 146.98, 139.61, 139.05, 139.01, 138.92, 138.91, 138.89, 138.88, 138.84, 138.83, 133.88, 127.79, 127.73, 127.70, 127.67, 127.38, 123.24; R_f = 0.33 (AlO_x N, 35% DCM:Hex); IR (KBr) ṽ 2924 (s), 2853 (s), 1734 (w), 1669 (w), 1594 (w), 1484.61 (m), 1464.33 (m), 1378 (s), 1262 (m), 1095 (m), 1027 (m), 1001 (s), 860 (s), 809 (s), 744 (w), 668 (s), 510.26 (w); HRMS (ESI, positive mode) *m/z* calcd for $C_{70}H_{47}S$: 919.3398 [M+H]+, found 919.3390 (-0.9 ppm).

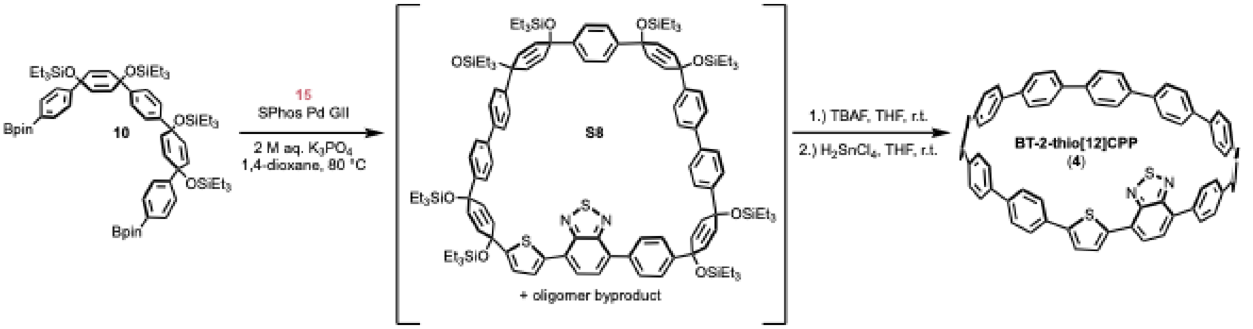

Scheme S13. Synthesis of intermediate **S8** and target compound **4** (store **S8** and **4** in freezer for long term storage)..

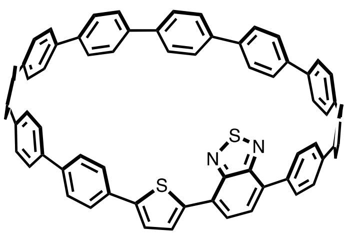

S8. Macrocyclization: Flame dried a 250 mL three-necked flask equipped with a stir bar, two glass stoppers, and a glass vacuum adapter. Once cool, charged the flask with **15** (0.217 g, 0.182 mmol, 1 equiv.) dissolved in DCM. Removed the solvent *in vacuo* and left the material on high vacuum for 40 minutes. Under positive nitrogen flow added **10** (0.201 g, 0.173 mmol, 0.95 equiv.), and SPhos Pd GII (0.014 g, 0.018 mmol, 0.1 equiv.). Backfilled and evacuated the system (3 cycles x 3 min), exchanged a glass stopper for a septum under positive nitrogen flow before dissolving the solid in dry 1,4-dioxane (182 mL). Sparged the reaction mixture for 30 minutes. Lowered the reaction vessel into a pre-heated oil bath set to 80 °C before adding sparged, aq. K_3PO_4 (18.2 mL, 2 M). Left the reaction to run overnight. Took an aliquot of the reaction mixture (checked by TLC: AlO_x N, 20% DCM:Hex) to reveal the reaction had progressed yet side products were noticeably present. The reaction was worked up by bringing the flask to room temperature before removing excess solvent *in vacuo*. The organics were extracted with ethyl acetate (20 mL x 3). The organics were washed with brine (15 mL x 3), dried over sodium sulfate, gravity filtered, and excess solvent was removed *in vacuo*. The material was partially purified by automated column chromatography (loaded in hexanes, pH 10 AlO_x, gradient 100% Hex - 40% DCM:Hex) to yield a sticky, green-yellow oil that was pushed forward without further purification.

4.Triethylsilyl deprotection and reductive aromatization: Using a syringe and needle, backfilled and evacuated (3 cycles x 3 min) a septum capped, pre-weighed, 20 mL scintillation vial containing a stir bar and partially pure **S8** (~0.085 g, 0.0418 mmol, 1 equiv.). Dissolved the material in dry THF (5.5 mL). To the flask added TBAF (0.35 mL, 1 M in THF) dropwise and left the reaction to run for 1 hour before quenching the reaction mixture with DI water (2 mL). Removed organic solvent *in vacuo*, collected the precipitated solid by vacuum filtration, and redissolved remaining solid in acetone (x 3). The crude solid was stored in the fridge overnight – no further purification was attempted.

Using a syringe and needle, backfilled and evacuated (3 cycles x 3 min) a septum capped, pre-weighed, 20 mL scintillation vial containing a stir bar and the crude solid (~0.047 g, 0.0418 mmol, 1 equiv.) from the previous step. Dissolved the solid in dry THF (6 mL) before adding a H_2SnCl_4 solution (4.5 mL, 0.04 M in THF) dropwise. Let the reaction run for 1 hour before adding DI water (5 mL) and extracting the organics with DCM (15 mL x 3). Washed the organics with brine (15 mL

x 3), dried over sodium sulfate, gravity filtered the mixture, and then removed the solvent *in vacuo*. **Purification and Characterization:** The material was purified by column chromatography (dry loaded, pH 10 AlO_x, 0% Hex - 50% Hex:DCM) and residual grease was removed via trituration in pentane to yield orange solid **4** (1.0 mg, 2% yield). ^{1}H NMR (600 MHz, Methylene Chloride-d_2) δ 8.13 (d, J = 3.9 Hz, 1H), 7.98 (d, J = 8.7 Hz, 2H), 7.77 (d, J = 7.7 Hz, 1H), 7.74 - 7.68 (m, 4H), 7.67 - 7.60 (m, 32H), 7.57 - 7.52 (m, 3H), 7.39 (d, J = 3.9 Hz, 1H); ^{13}C NMR (151 MHz, Methylene Chloride-d_2) δ 154.14, 154.06, 147.52, 142.13, 140.11, 139.93, 139.25, 139.11, 139.02, 138.96, 138.95, 138.92, 138.90, 138.87, 138.83, 138.78, 138.58, 136.84, 133.59, 132.56, 130.73, 129.41, 129.10, 127.82, 127.81, 127.77, 127.72, 127.68, 127.63, 127.52, 127.34, 126.62, 126.01, 123.65; R_f = 0.33 (AlO_x N, 50% DCM:Hex); HRMS (ESI, positive mode) m/z calcd for $C_{70}H_{45}N_2S_2$: 977.3024 [M+H]+, found 977.3037 (1.3 ppm).

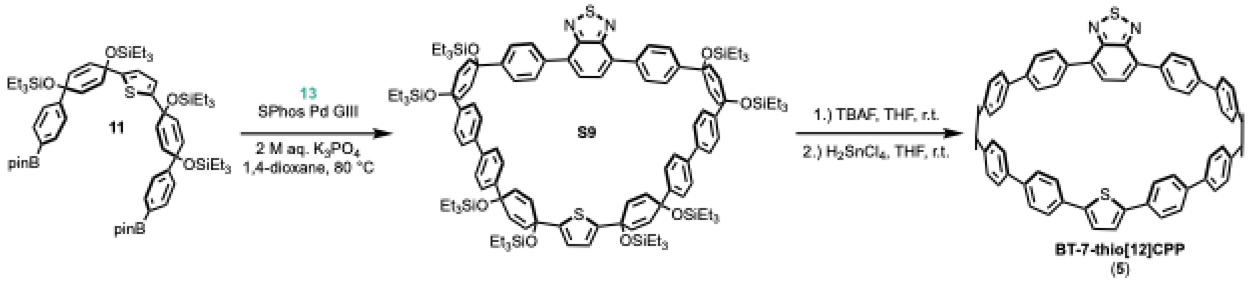

Scheme S14. Synthesis of intermediate **S9** and target compound **5** (store **S9** and **5** in freezer for long term storage).

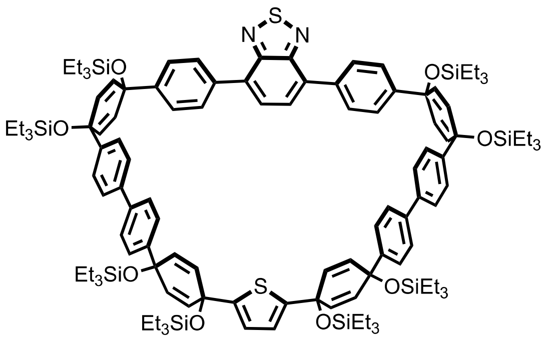

S9. Macrocyclization: Flame dried a 500 mL round bottomed flask, plus a 1 L round bottomed flask equipped with a stir bar and glass vacuum adapter. To the 500 mL round bottomed flask added dry 1,4-dioxane and sparged the solvent for 1 hour. Backfilled and evacuated (3 cycles x 2 min) the 1 L round bottomed flask before charging the flask with **13** (0.550 g, 0.463 mmol, 1 equiv.), **11** (0.594 g, 0.510 mmol, 1.1 equiv.), and SPhos Pd GIII (0.037 g, 0.0463 mmol, 0.10 equiv.). Backfilled and evacuated (3 cycles x 5 min) the reaction vessel before quickly exchanging the glass vacuum adapter for a septum. Cannula transferred the contents of the 500 mL round bottomed flask to the 1 L reaction vessel, then sparged the reaction mixture for 15 minutes. The flask was lowered into a pre-heated oil bath set to 80 °C and left to equilibrate for 15 minutes. Sparged, aq. K_3PO_4 (46.4 mL, 2 M) was added dropwise into the reaction vessel, which was then stirred overnight. The reaction was brought to room temperature before the material was poured through a Celite plug and rinsed with DCM. The filtrate was dried over sodium sulfate, gravity filtered, and excess organic solvent was removed *in vacuo*. To remove residual solvent the crude material was placed on high vacuum for 30 minutes, redissolved in acetone before removing solvent *in vacuo*, then placed back on high vacuum for 30 minutes (x 4). **Purification and Characterization:** The material was purified by column chromatography (loaded in 10% DCM:Hex, pH 10 AlO_x, gradient 10% DCM:Hex - 20% DCM:Hex) to give fluffy, neon green solid

S9 (0.279 g, 30% yield). ^{1}H NMR (600 MHz, Methylene Chloride-*d_2*) δ 7.79 (d, *J* = 8.5 Hz, 4H), 7.68 (s, 2H), 7.48 (d, *J* = 8.6 Hz, 4H), 7.45 - 7.40 (m, 12H), 7.33 (d, *J* = 8.5 Hz, 4H), 6.46 (s, 2H), 6.16 (t, *J* = 9.8 Hz, 8H), 6.08 (d, *J* = 10.2 Hz, 4H), 5.94 (d, *J* = 10.1 Hz, 4H), 1.03 - 0.91 (m, 72H), 0.75 - 0.57 (m, 48H); ^{13}C NMR (151 MHz, Methylene Chloride-*d_2*) δ 154.46, 152.14, 146.26, 145.47, 145.26, 140.36, 139.97, 136.68, 133.13, 132.35, 132.00, 130.89, 129.34, 128.37, 127.19, 127.15, 127.06, 126.48, 126.45, 122.37, 72.39, 72.03, 71.87, 69.74, 7.32, 7.28, 7.25, 7.22, 6.90, 6.89, 6.81, 6.71; R_f = 0.25 (AlO_x N, 20% DCM:Hex); HRMS (MALDI) *m*/*z* calcd for $C_{118}H_{164}N_2O_8S_2Si_8$: 2,025.0083 [M]+, found 2,025.0078 (2.5552 ppm).

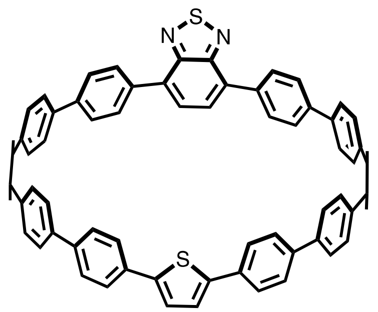

5. Triethylsilyl deprotection and reductive aromatization: Using a syringe and needle, backfilled and evacuated (3 cycles x 3 min) a septum capped, pre-weighed, 20 mL scintillation vial containing a stir bar and **S9** (0.109 g, 0.054 mmol, 1 equiv.). Dissolved the material in dry THF (6.75 mL). To the flask added TBAF (0.48 mL, 1 M in THF) dropwise and left the reaction to run for 1 hour. Quenched the reaction mixture with DI water (2 mL). Removed organic solvent *in vacuo*, collected the precipitated solid by vacuum filtration, and redissolved remaining solid in acetone (x 3). The crude solid was stored in the fridge until further use - no further purification was attempted.

Flame dried a 25 mL round bottomed flask equipped with a stir bar and glass vacuum adapter. Once cool, quickly transferred the crude material (0.039 g, 0.035 mmol, 1 equiv.) from the previous step into the reaction vessel. Backfilled and evacuated the flask (3 cycles x 3 min) before dissolving the solid in dry THF (8 mL). Added a H_2SnCl_4 solution (3.5 mL, 0.04 M in THF) dropwise. Let the reaction run for 1 hour before quenching the reaction with sat. sodium bicarbonate (5 mL). The organics were extracted with DCM (20 mL x 3). Washed the organics with brine (20 mL x 3), dried over sodium sulfate, gravity filtered the mixture, and then removed the solvent *in vacuo*. **Purification and Characterization:** The material was purified by column chromatography (dry loaded, pH 10 AlO_x, gradient 0% Hex - 50% DCM:Hex) and triturated in diethyl ether to yield light yellow solid **5** (13.0 mg, 38% yield). ^{1}H NMR (500 MHz, Methylene Chloride-*d_2*) δ 8.03 (d, *J* = 8.6 Hz, 4H), 7.72 - 7.58 (m, 34H), 7.50 (d, *J* = 8.4 Hz, 4H), 7.26 (s, 2H); ^{13}C NMR (151 MHz, Methylene Chloride-*d_2*) δ 154.69, 147.02, 140.10, 139.59, 139.19, 139.06, 139.02, 138.98, 138.84, 138.62, 136.81, 133.88, 131.64, 130.67, 128.77, 127.80, 127.77, 127.67, 127.64, 127.40, 127.38, 123.23; R_f = 0.38 (AlO_x N, 50% DCM:Hex); IR (ATR) ṽ 3024 (w), 1482 (w), 812 (m), 746 (w); HRMS (ESI, positive mode) *m*/*z* calcd for $C_{70}H_{45}N_2S_2$: 977.3024 [M+H]+, found 977.3016 (-0.8 ppm).

c. **D-A[10]CPP Derivatives: BT-m[10]CPP (2), BT-2-thio[10]CPP (6), and BT-2,2-dithio[10]CPP (7)**

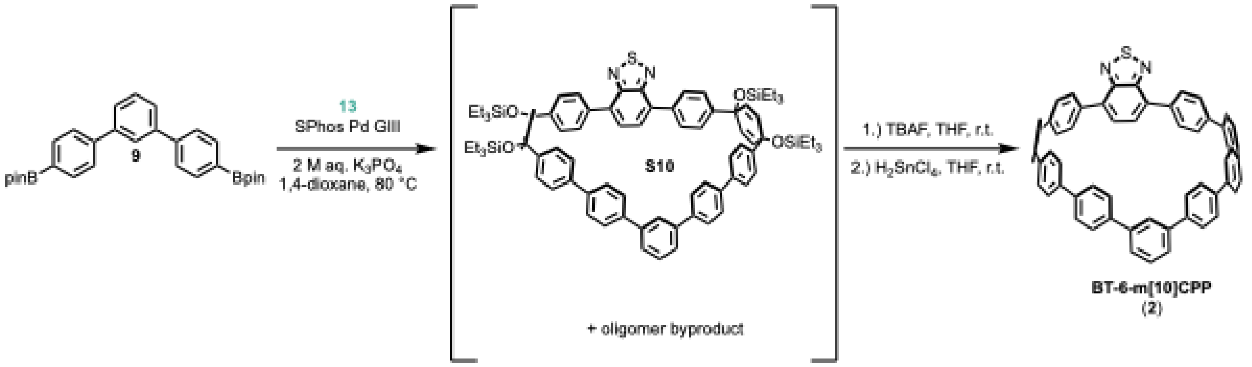

Scheme S15. Synthesis of intermediate **S10** and target compound **2** (store **S10** and **2** in freezer for long term storage).

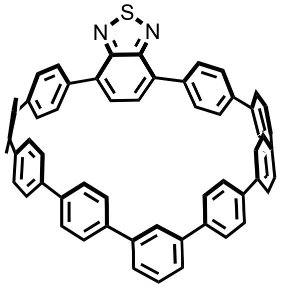

S10. Macrocyclization: Flame dried a 500 mL round bottomed flask equipped with a stir bar and a glass vacuum adapter. Once cool, quickly added **9** (0.229 g, 0.475 mmol, 1.01 equiv.), **13** (0.6 g, 0.470 mmol, 1 equiv.), and SPhos Pd GIII (0.037 g, 0.047 mmol, 0.1 equiv.). Exchanged the glass vacuum adapter for a septum, then cannula transferred dry 1,4-dioxane (392 mL) and sparged the reaction mixture for 1 hour. Lowered the reaction vessel into a pre-heated oil bath set to 80 °C and let equilibrate for 20 minutes. Added sparged, aq. K_3PO_4 (39.2 mL, 2 M). Left the reaction to run overnight. The reaction was worked up by bringing the flask to room temperature before removing excess solvent *in vacuo*. The organics were extracted with DCM (50 mL x 3). The organics were washed with brine (50 mL x 3), dried over sodium sulfate, gravity filtered, and excess solvent was removed *in vacuo*. The material was partially purified by automated column chromatography (loaded in 5% DCM:Hex, SiO_2, gradient 5% Hex - 45% DCM:Hex) to yield a sticky, green oil that was pushed forward without further purification.

2.Triethylsilyl deprotection and reductive aromatization: Flame dried a 15 mL round bottomed flask equipped with a stir bar and a glass vacuum adapter. Backfilled and evacuated (3 cycles x 3 min) the flask before adding partially pure **S10** (~0.05 g, 0.037 mmol, 1 equiv.). Backfilled and evacuated the flask (3 cycles x 3 min) a second time before dissolving the material in dry THF (5 mL). To the flask added TBAF (0.20 mL, 1 M in THF) dropwise and left the reaction to run for 1 hour before quenching the reaction mixture with DI water (5 mL). Collected the precipitated solid by vacuum filtration, rinsed the solid with DI water (5 mL), and allowed to dry over the course of 3 hours. The crude solid was stored in the fridge overnight - no further purification was attempted.

Flame dried a 15 mL round bottomed flask equipped with a stir bar and a glass vacuum adapter. Backfilled and evacuated (3 cycles x 3 min) the flask before adding the crude solid (~0.011 g,

0.0123 mmol, 1 equiv.) from the previous step. Backfilled and evacuated the flask (3 cycles x 3 min) a second time before dissolving the solid in dry THF (4 mL). To the flask added H_2SnCl_4 solution (0.62 mL, 0.04 M in THF) dropwise. Let the reaction run for 1 hour before adding sat. sodium bicarbonate (2 mL). Removed excess solvent *in vacuo* before extracting the organics with DCM (15 mL x 3). Washed the organics with brine (15 mL x 3), dried over sodium sulfate, gravity filtered the mixture, and then removed the solvent *in vacuo*. **Purification and Characterization:** The material was purified by column chromatography (wet loaded 30% DCM:Hex, pH 10 AlO_x, 30% DCM:Hex - 100% DCM) to yield yellow solid **2** (2.0 mg, 22% yield). ^{1}H NMR (500 MHz, Methylene Chloride-d_2) δ 7.98 (d, J = 8.9 Hz, 4H), 7.64 - 7.60 (m, 19H), 7.56 - 7.53 (m, 8H), 7.51 (s, 2H), 7.44 (d, J = 8.4 Hz, 4H), 6.84 (broad s, 1H); ^{13}C NMR (126 MHz, Methylene Chloride-d_2) δ 7 154.87, 142.75, 141.60, 140.12, 139.82, 139.67, 138.73, 138.65, 138.62, 136.77, 133.88, 131.57, 131.10, 129.50, 128.93, 128.32, 127.97, 127.82, 127.65, 127.56, 127.53, 124.59; R_f = 0.33 (AlO_x N, 50% DCM:Hex); HRMS (ESI, positive mode) *m/z* calcd for $C_{60}H_{38}N_2S$: 818.2756 [M]+, found 818.2782 (3.2 ppm).

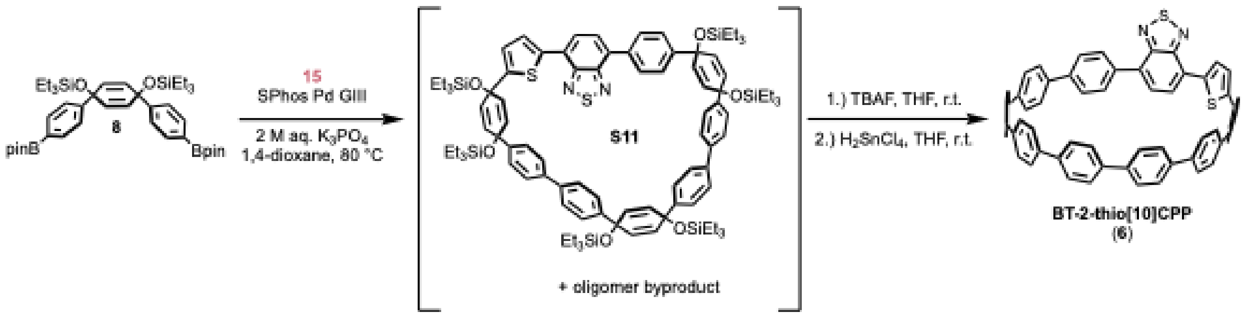

Scheme S16. Synthesis of intermediate **S11** and target compound **6** (store **S11** and **6** in freezer for long term storage).

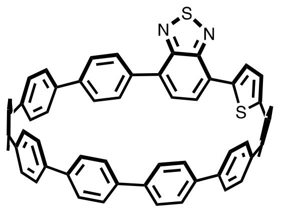

S11. Macrocyclization: Flame dried a 100 mL three-necked flask equipped with a stir bar, two glass stopers, and a glass vacuum adapter. Under positive nitrogen flow added **15** (0.049 g, 0.041 mmol, 1 equiv.) dissolved in DCM, removed the solvent *in vacuo*, and left on high vacuum for 1 hour. To the flask then added **8** (0.032 g, 0.043 mmol, 1.05 equiv.) and SPhos Pd GIII (3.2 mg, 4.02 µmol, 0.1 equiv.). Backfilled and evacuated the system (3 cycles x 3 min). Exchanged the glass vacuum adapter for a septum, then dissolved the solids in dry 1,4-dioxane (43 mL). Lowered the reaction vessel into a pre-heated oil bath set to 80 °C and let equilibrate for 15 minutes. Added sparged, aq. K_3PO_4 (4.09 mL, 2 M). Left the reaction to run overnight. The reaction was worked up by bringing the flask to room temperature before removing excess solvent *in vacuo*. The organics were extracted with DCM (20 mL x 3). The organics were washed with brine (20 mL x 3), dried over sodium sulfate, gravity filtered, and excess solvent was removed *in vacuo*. The material was partially purified by automated column chromatography (dry loaded, pH 10 AlO_x, gradient 100% Hex - 40% DCM:Hex) to yield a sticky, orange oil that was pushed forward without further purification.

6.Triethylsilyl deprotection and reductive aromatization: Flame dried a 15 mL heart shaped flask equipped with a stir bar, a glass stopper, and a glass vacuum adapter. Added partially pure

S11 (~0.084 g, 0.052 mmol, 1 equiv.) and backfilled and evacuated the flask (3 cycles x 3 min) before dissolving the material in dry THF (6.53 mL). To the flask added TBAF (0.31 mL, 1 M in THF) dropwise and left the reaction to run for 1 hour before quenching the reaction mixture with DI water (1.5 mL). Collected the precipitated solid by vacuum filtration and allowed to dry over the course of 35 minutes. The crude solid was stored in the fridge overnight - no further purification was attempted.

Connected a 15 mL heart shaped flask - equipped with a stir bar, a glass stopper, and a glass vacuum adapter - containing the crude solid (0.048 g, 0.052 mmol, 1 equiv.) from the previous step onto the Schlenk line. Backfilled and evacuated the system (3 cycles x 3 min) before dissolving the solid in dry THF (4 mL). Added H_2SnCl_4 solution (4.3 mL, 0.04 M in THF) dropwise. Let the reaction run for 1 hour before quenching with sat. sodium bicarbonate (4 mL). Remove the solvent *in vacuo*, then extracted the organics with DCM (20 mL x 3). Washed the organics with brine (20 mL x 3), dried over sodium sulfate, gravity filtered the mixture, and then removed the solvent *in vacuo*. To remove residual solvent the crude material was placed on high vacuum for 30 minutes, redissolved in acetone before removing solvent *in vacuo*, then placed back on high vacuum for 30 minutes (x2). **Purification and Characterization:** The material was purified by column chromatography (wet loaded 20% DCM:Hex, pH 10 AlO_x, 20% DCM:Hex - 40% DCM) and triturated in diethyl ether to remove residual grease to yield orange solid **6** (1.0 mg, 2% yield). ^{1}H NMR (600 MHz, Methylene Chloride-*d₂*) δ 8.03 (d, *J* = 3.9 Hz, 1H), 7.92 (d, *J* = 8.7 Hz, 2H), 7.70 (d, *J* = 7.8 Hz, 1H), 7.71 - 7.56 (m, 28H), 7.47 - 7.45 (m, 3H), 7.32 (d, *J* = 3.9 Hz, 1H); ^{13}C NMR (151 MHz, Methylene Chloride-d_2) δ 154.53, 154.00, 148.79, 143.07, 139.99, 139.68, 138.98, 138.92, 138.71, 138.60, 138.58, 138.54, 138.52, 138.47, 138.21, 136.68, 133.54, 132.51, 131.28, 129.17, 128.80, 127.83, 127.80, 127.76, 127.74, 127.71, 127.67, 127.64, 127.39, 126.56, 125.85, 123.31; R_f = 0.57 (AlO_x N, 50% DCM:Hex); HRMS (ESI, positive mode) *m*/*z* calcd for $C_{58}H_{37}N_2S_2$: 825.2398 [M+H]+, found 825.2382 (-1.9 ppm).

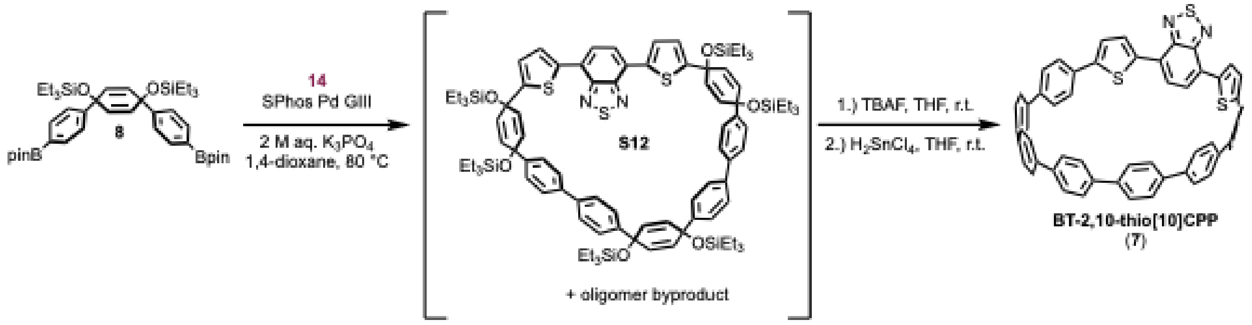

Scheme S17. Synthesis of intermediate **S12** and target compound **7** (store **S12** and **7** in freezer for long term storage).

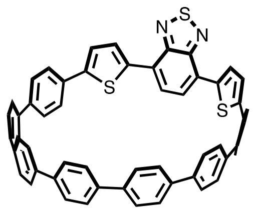

S12. Macrocyclization: Flame dried a 100 mL three-necked flask equipped with a stir bar, two glass stopers, and a glass vacuum adapter. Under positive nitrogen flow added **14** (0.05 g, 0.042 mmol, 1 equiv.), **8** (0.033 g, 0.044 mmol, 1.05 equiv.) and SPhos Pd GIII (3.0 mg, 3.89 µmol, 0.1 equiv.). Backfilled and evacuated the system (3 cycles x 3 min). Exchanged the glass vacuum adapter for a septum, then dissolved the solids in dry 1,4-dioxane (42 mL). Lowered the reaction

vessel into a pre-heated oil bath set to 80 °C and let equilibrate for 15 minutes. Added sparged, aq. K_3PO_4 (4.17 mL, 2 M). Left the reaction to run overnight. The reaction was worked up by bringing the flask to room temperature before removing excess solvent *in vacuo*. The organics were extracted with DCM (20 mL x 4). The organics were washed with brine (20 mL x 3), dried over sodium sulfate, gravity filtered, and excess solvent was removed *in vacuo*. To remove residual solvent the crude material was placed on high vacuum for 30 minutes, redissolved in acetone before removing solvent *in vacuo*, then placed back on high vacuum for 30 minutes (x2). The material was partially purified by automated column chromatography (loaded in hexanes, pH 10 AlO_x, gradient 100% Hex - 40% DCM:Hex) to yield a sticky, red oil that was pushed forward without further purification.

7.Triethylsilyl deprotection and reductive aromatization: To a septum capped, pre-weighed, 20 mL scintillation vial containing a stir bar, added partially pure **S11** (~0.019 g, 0.012 mmol, 1 equiv.) dissolved in DCM. Removed the solvent *in vacuo* and left the material on high vacuum for 1 hour. Dissolved the material in dry THF (1.5 mL). To the flask added TBAF (0.073 mL, 1 M in THF) dropwise and left the reaction to run for 30 minutes. Quenched the reaction mixture with DI water (3 mL). Removed organic solvent *in vacuo*, collected the precipitated solid by vacuum filtration, and allowed the material to dry for 30 minutes. The crude solid was stored in the fridge until further use - no further purification was attempted.

Flame dried a 15 mL heart shaped flask equipped with a stir bar, a glass stopper, and a glass vacuum adapter. Added partially pure **S12** (~8.8 mg, 9.43 µmol, 1 equiv.) and backfilled and evacuated the flask (3 cycles x 3 min) before dissolving the material in dry THF (5 mL). Added H_2SnCl_4 solution (0.78 mL, 0.04 M in THF) dropwise. Let the reaction run for 30 minutes before the solvent was removed *in vacuo*. Extracted the organics with DCM (20 mL x 3). Washed the organics with brine (20 mL x 3), dried over sodium sulfate, gravity filtered the mixture, and then removed the solvent *in vacuo*. To remove residual solvent the crude material was placed on high vacuum for 30 minutes, redissolved in acetone before removing solvent *in vacuo*, then placed back on high vacuum for 30 minutes (x2). **Purification and Characterization:** The material was purified by column chromatography (wet loaded 20% DCM:Hex, pH 10 AlO_x, 20% DCM:Hex - 40% DCM) to yield red solid **7** (4.0 mg, 27% yield). ^{1}H NMR (600 MHz, Methylene Chloride-d_2) δ 7.99 (d, J = 3.8 Hz, 2H), 7.67 (s, 2H), 7.61 - 7.57 (m, 24H), 7.49 (d, J = 8.4 Hz, 4H), 7.31 (d, J = 3.9 Hz, 2H); ^{13}C NMR (151 MHz, Methylene Chloride-d_2) δ 153.72, 148.40, 143.01, 139.83, 138.90, 138.67, 138.58, 138.56, 138.46, 133.55, 129.08, 127.85, 127.82, 127.78, 127.76, 127.74, 126.70, 126.62, 123.29; R_f = 0.35 (AlO_x N, 40% DCM:Hex); HRMS (MALDI) *m/z* calcd for $C_{56}H_{34}N_2S_3$: 830.1884 [M]+, found 830.1879 (2.3901 ppm).

3. Nuclear Magnetic Resonance Spectra

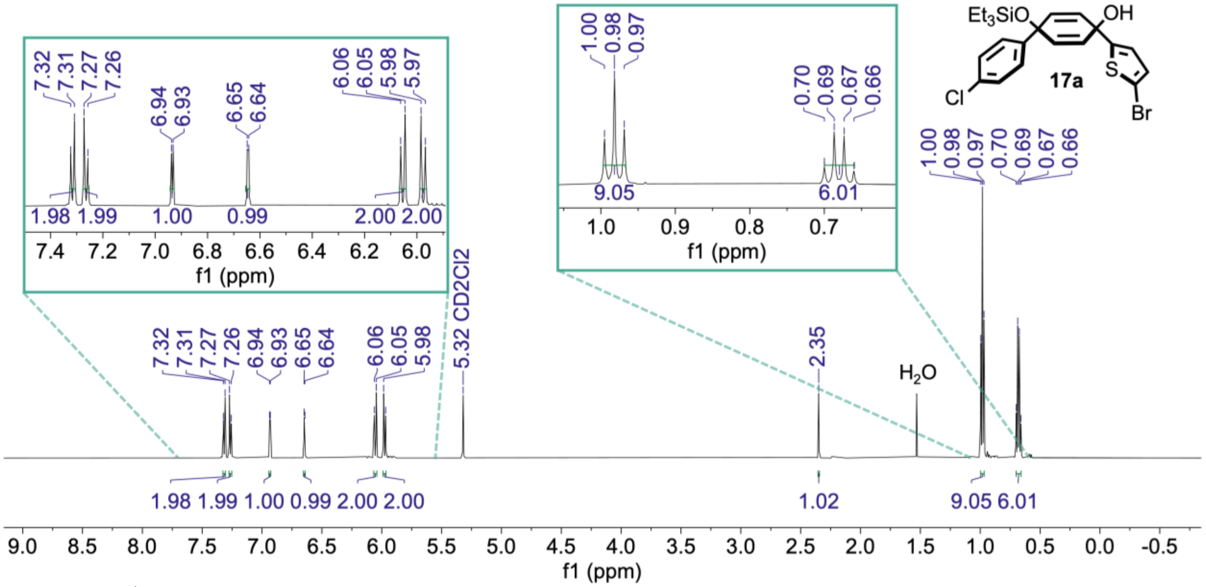

Figure S1. ^{1}H NMR spectrum (600 MHz) of **17a** in Methylene Chloride-d_2 at room temperature.

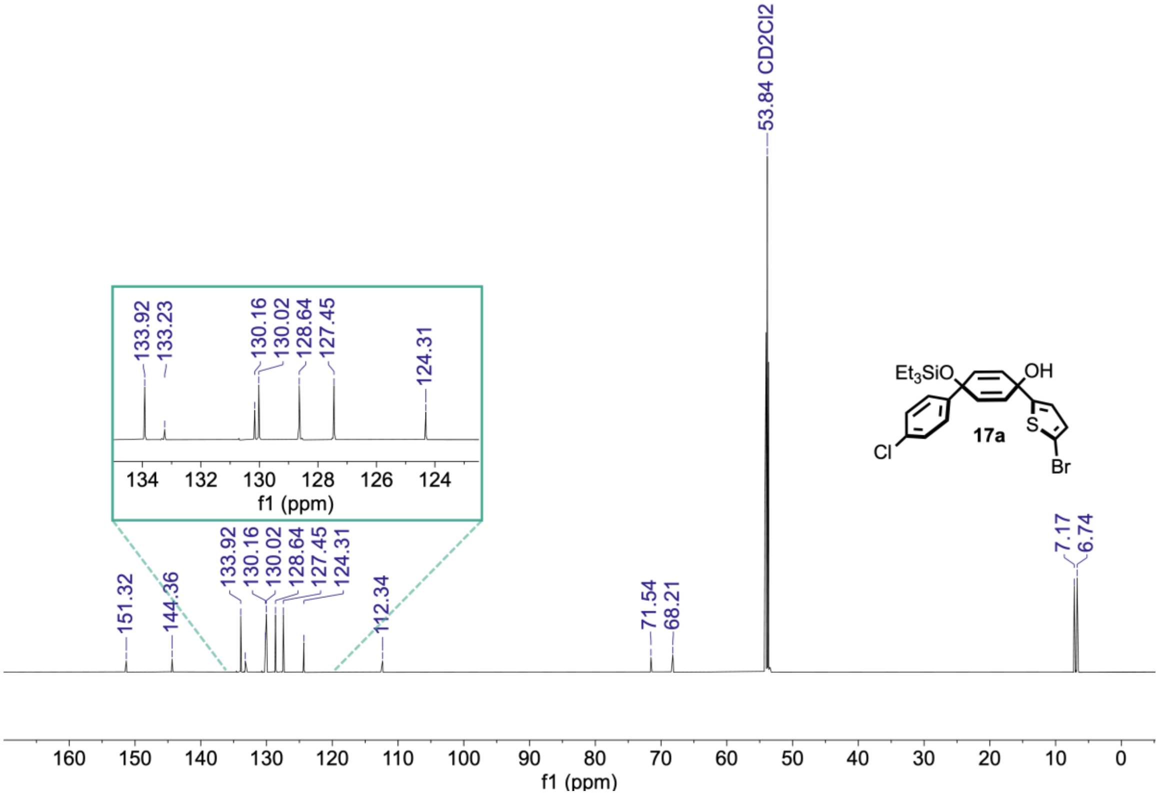

Figure S2. ^{13}C NMR spectrum (151 MHz) of **17a** in Methylene Chloride-d_2 at room temperature.

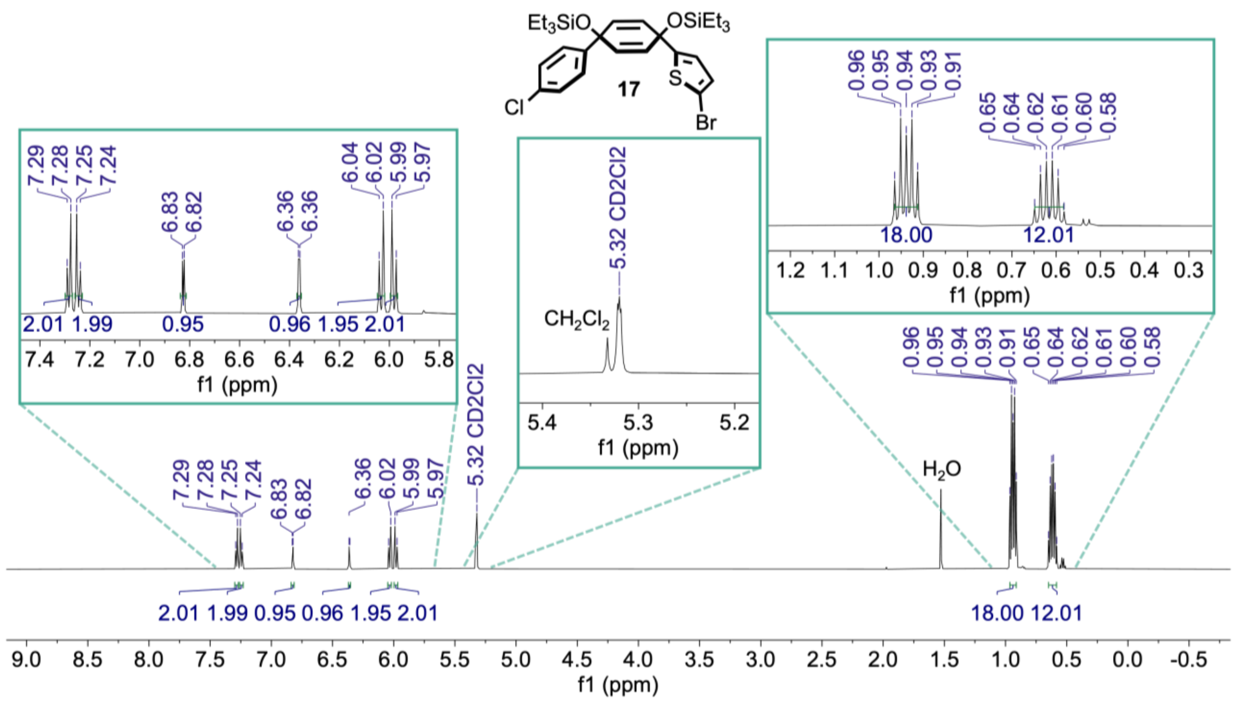

Figure S3. ^{1}H NMR spectrum (600 MHz) of **17** in Methylene Chloride-d_2 at room temperature.

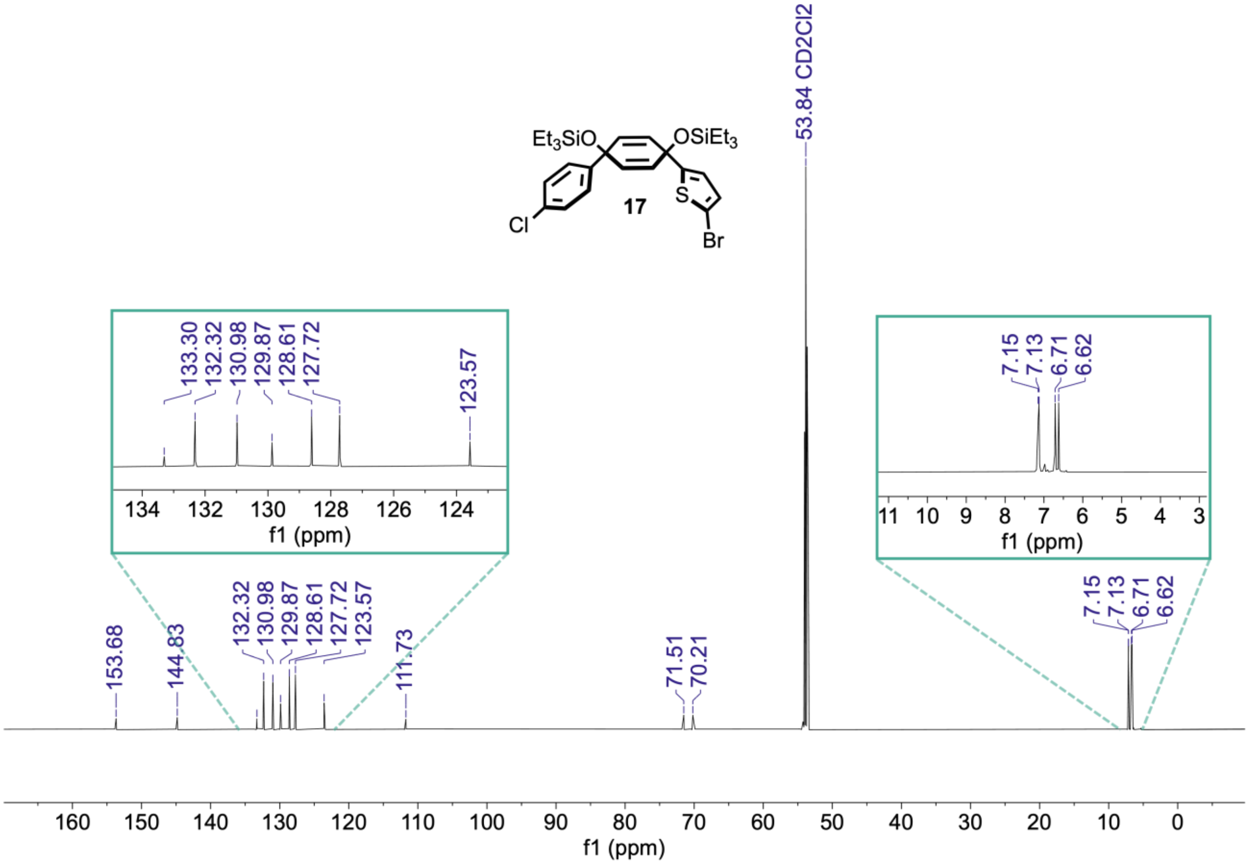

Figure S4. ^{13}C NMR spectrum (151 MHz) of **17** in Methylene Chloride-d_2 at room temperature.

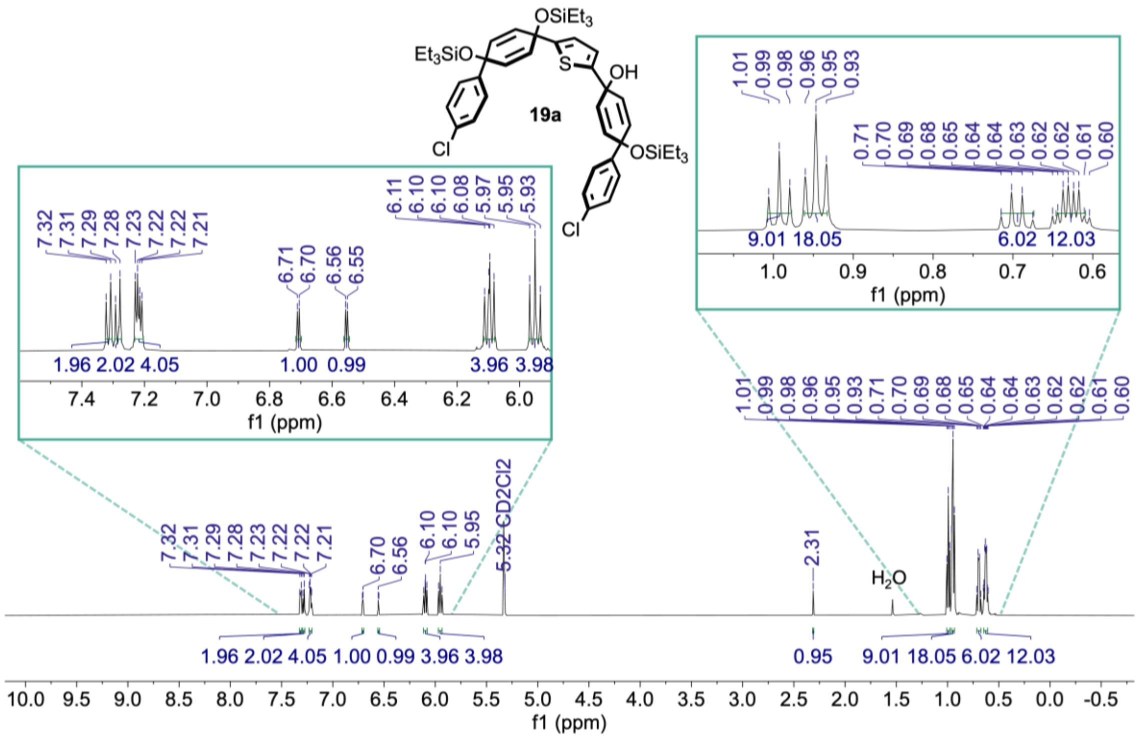

Figure S5. ^{1}H NMR spectrum (600 MHz) of **19a** in Methylene Chloride-d_2 at room temperature.

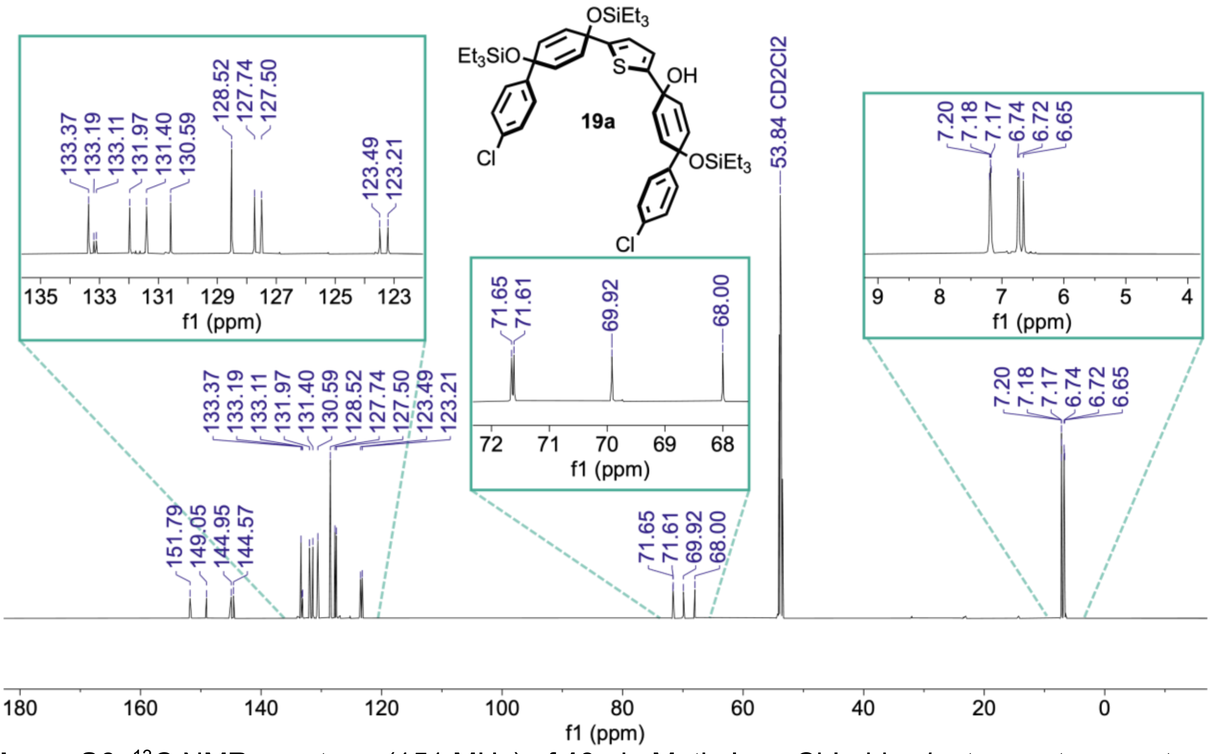

Figure S6. ^{13}C NMR spectrum (151 MHz) of **19a** in Methylene Chloride-d_2 at room temperature.

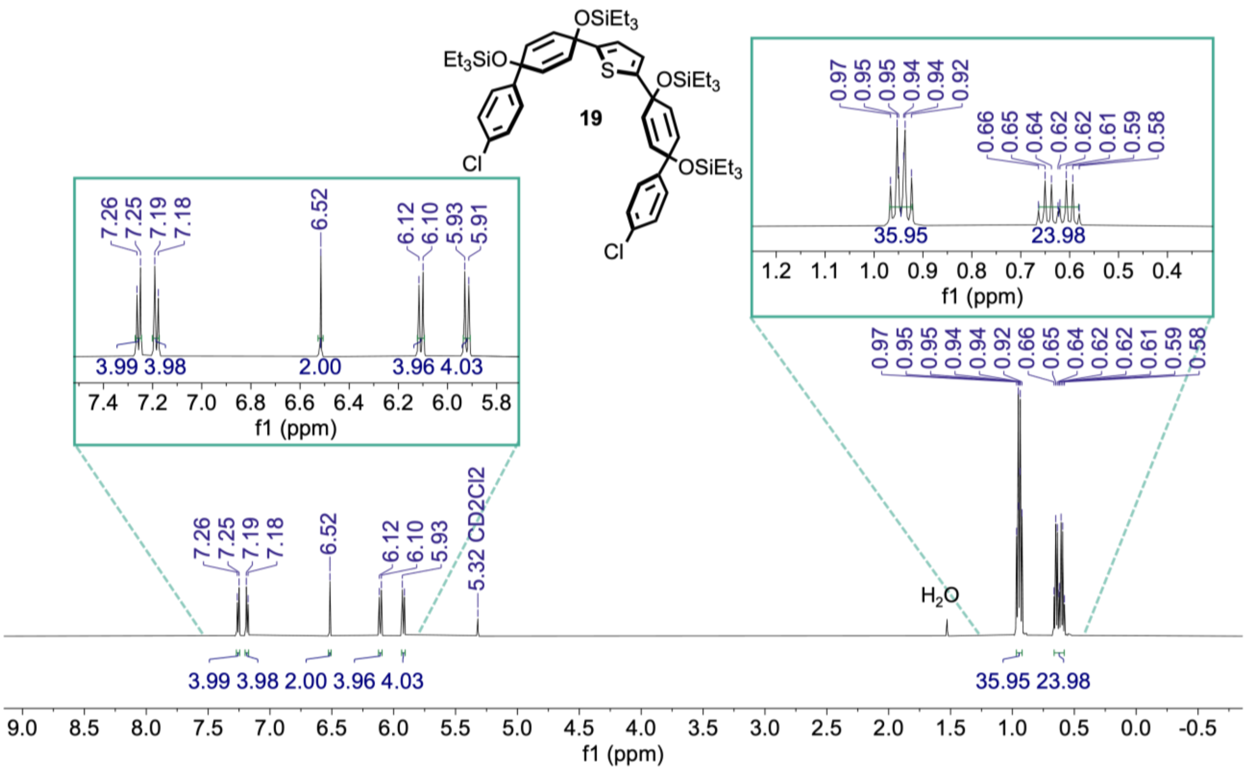

Figure S7. ^{1}H NMR spectrum (600 MHz) of **19** in Methylene Chloride-d_2 at room temperature.

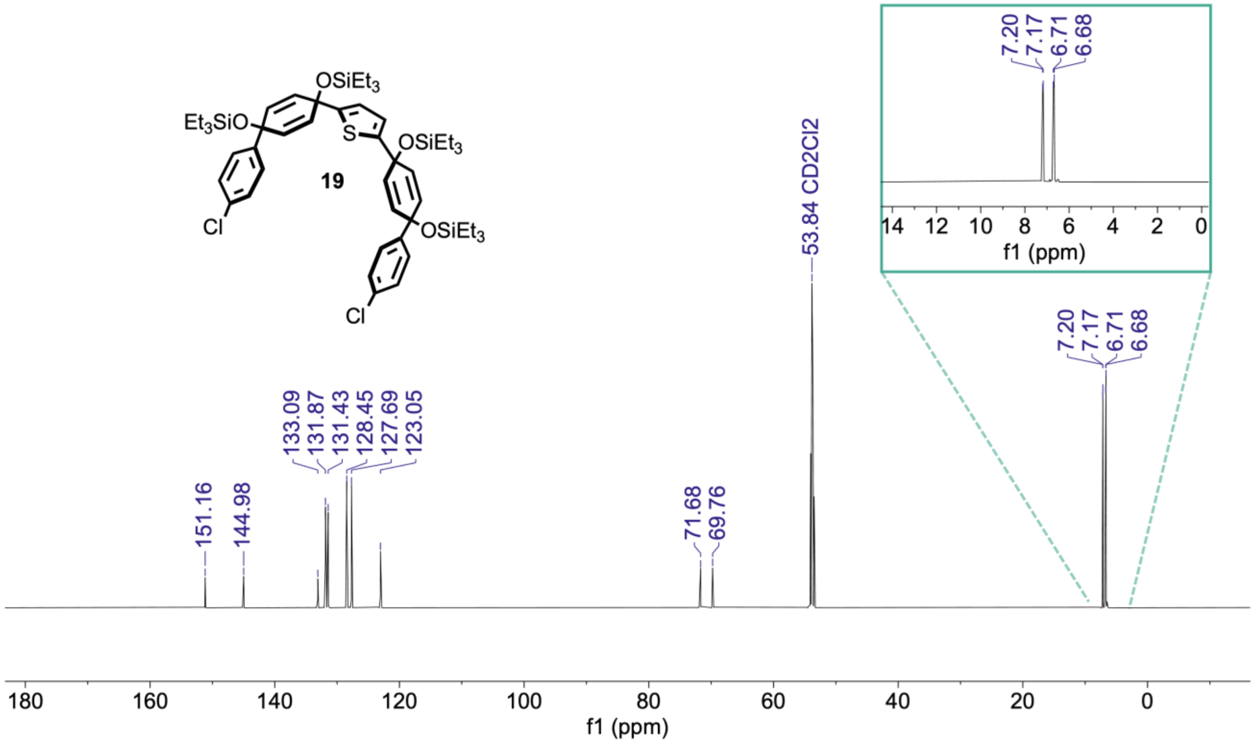

Figure S8. ^{13}C NMR spectrum (151 MHz) of **19** in Methylene Chloride-d_2 at room temperature.

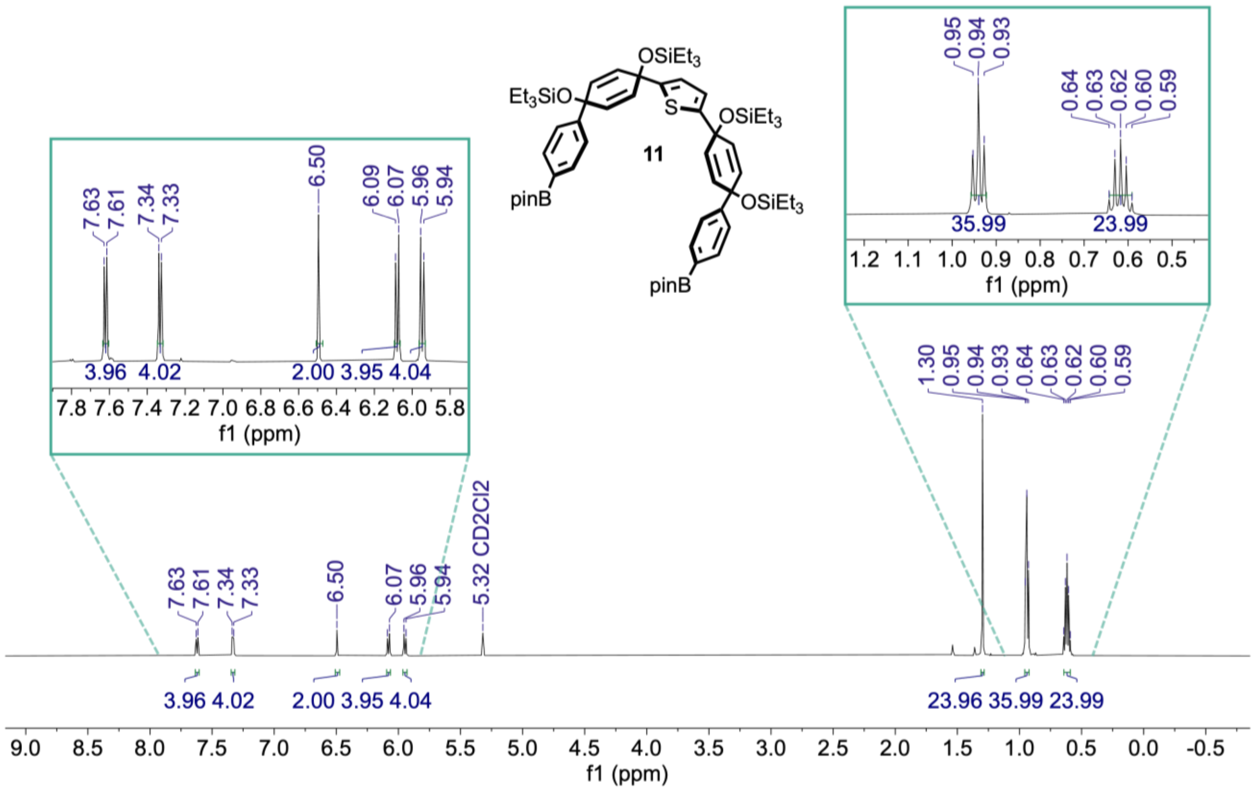

Figure S9. ^{1}H NMR spectrum (600 MHz) of **11** in Methylene Chloride-d_2 at room temperature.

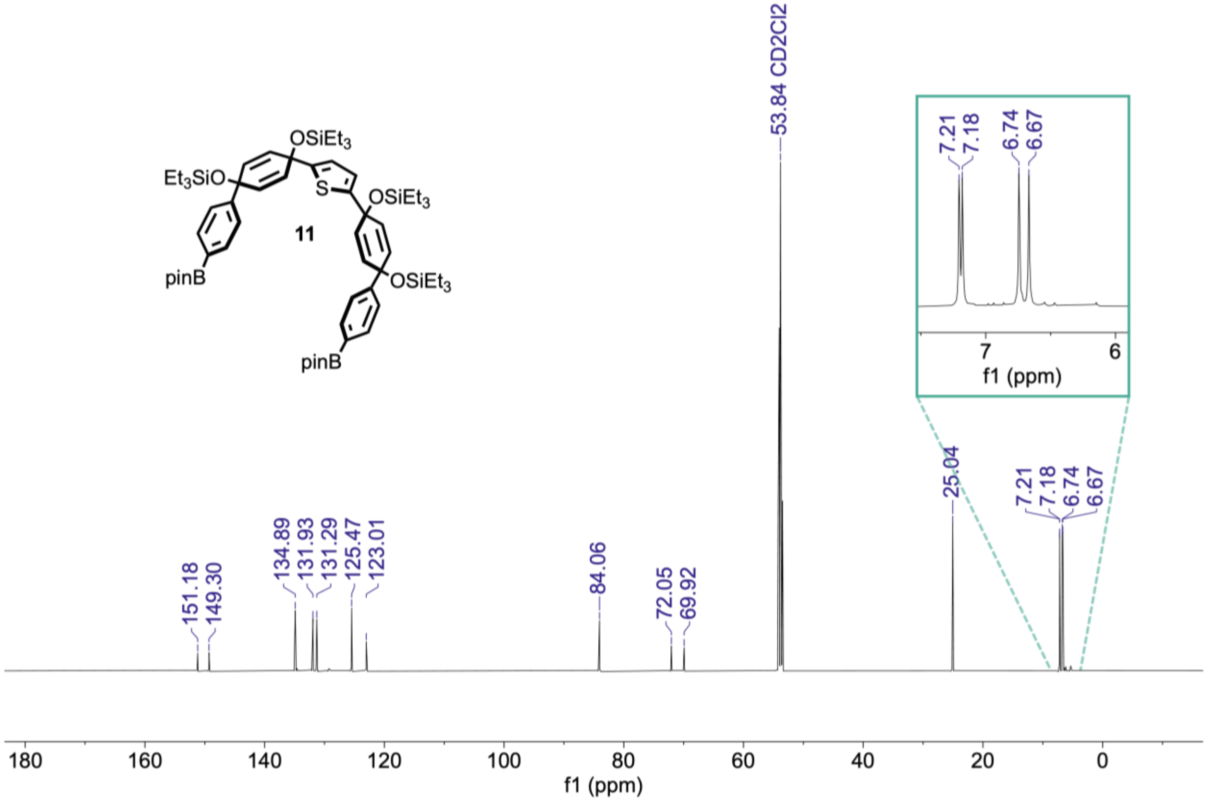

Figure S10. ^{13}C NMR spectrum (151 MHz) of **11** in Methylene Chloride-d_2 at room temperature.

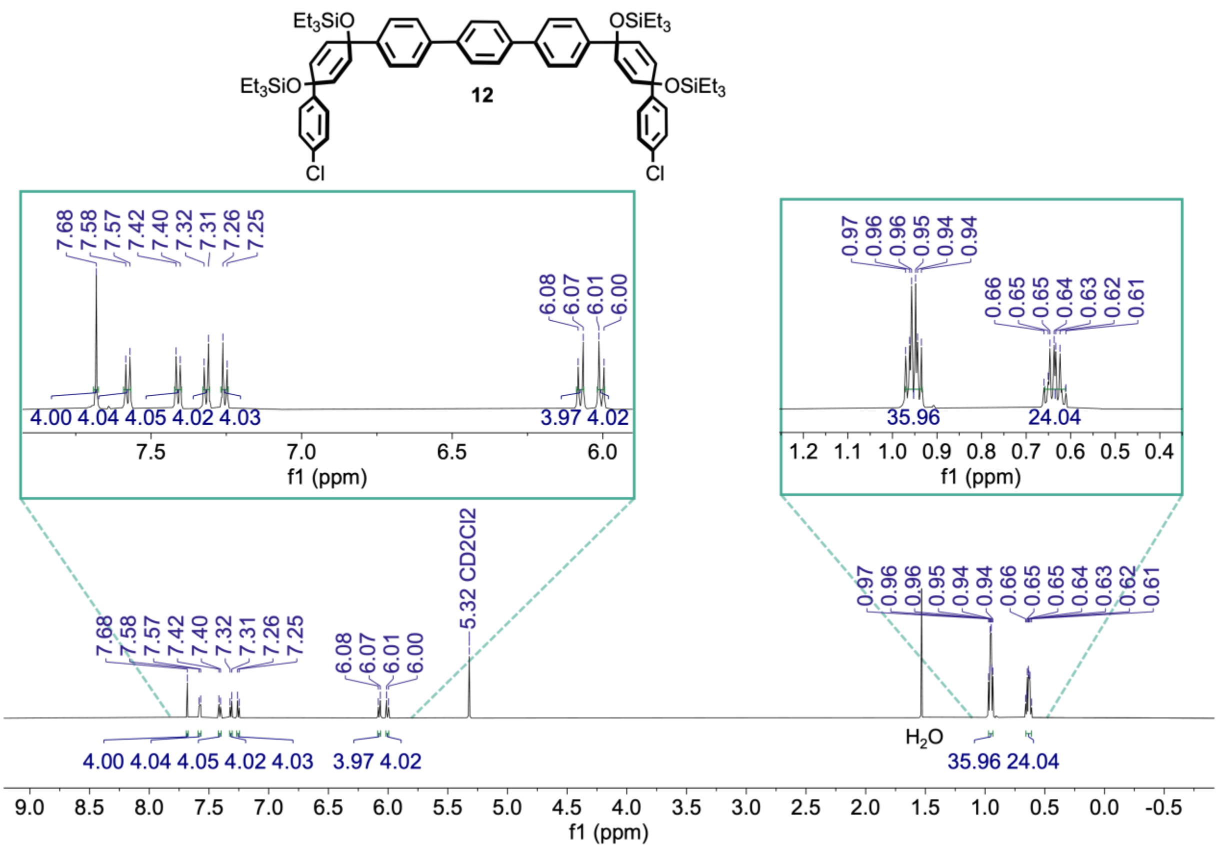

Figure S11. ^{1}H NMR spectrum (600 MHz) of **12** in Methylene Chloride-d_2 at room temperature.

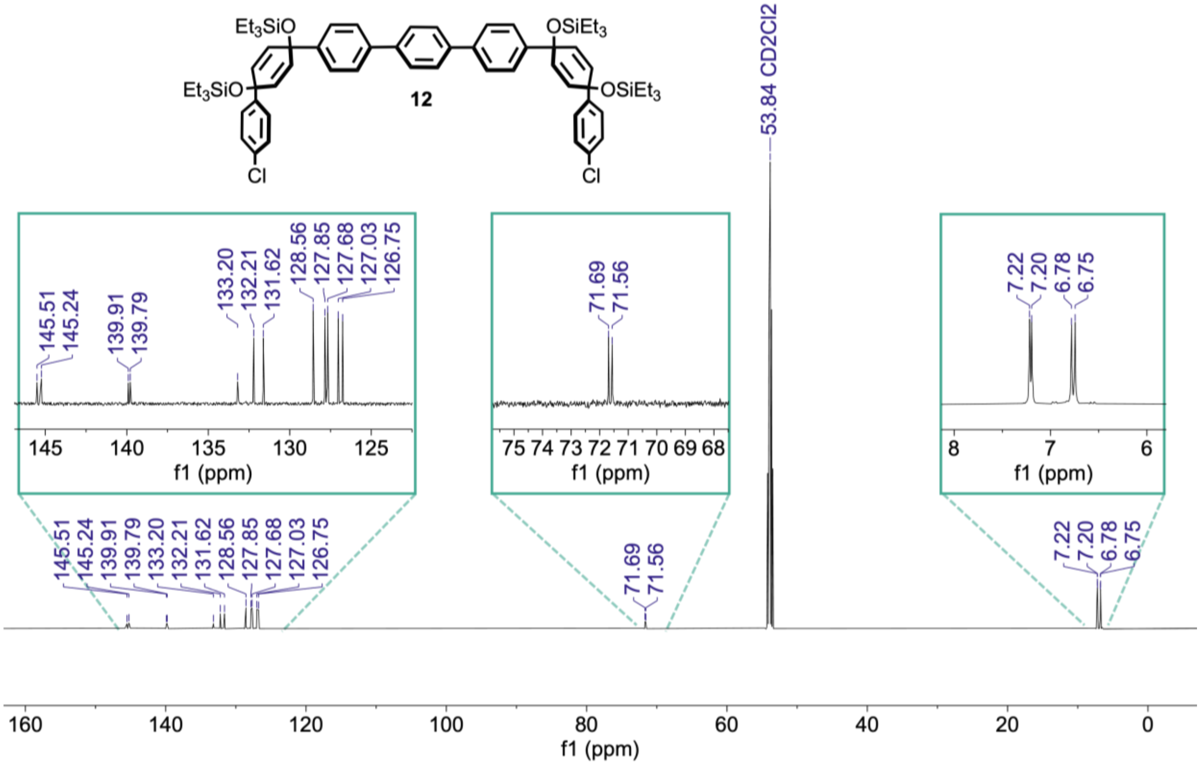

Figure S12. ^{13}C NMR spectrum (151 MHz) of **12** in Methylene Chloride-d_2 at room temperature.

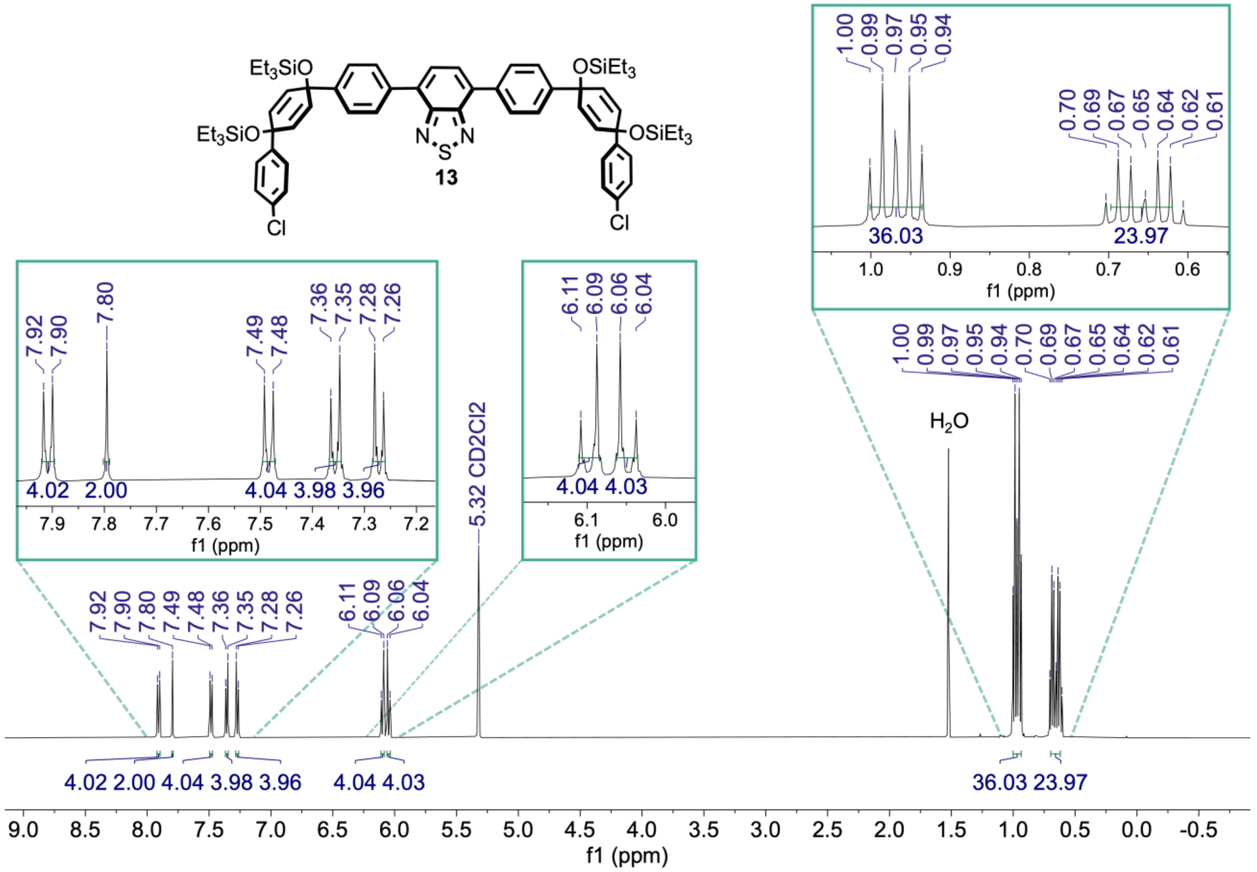

Figure S13. ^{1}H NMR spectrum (600 MHz) of **12** in Methylene Chloride-d_2 at room temperature.

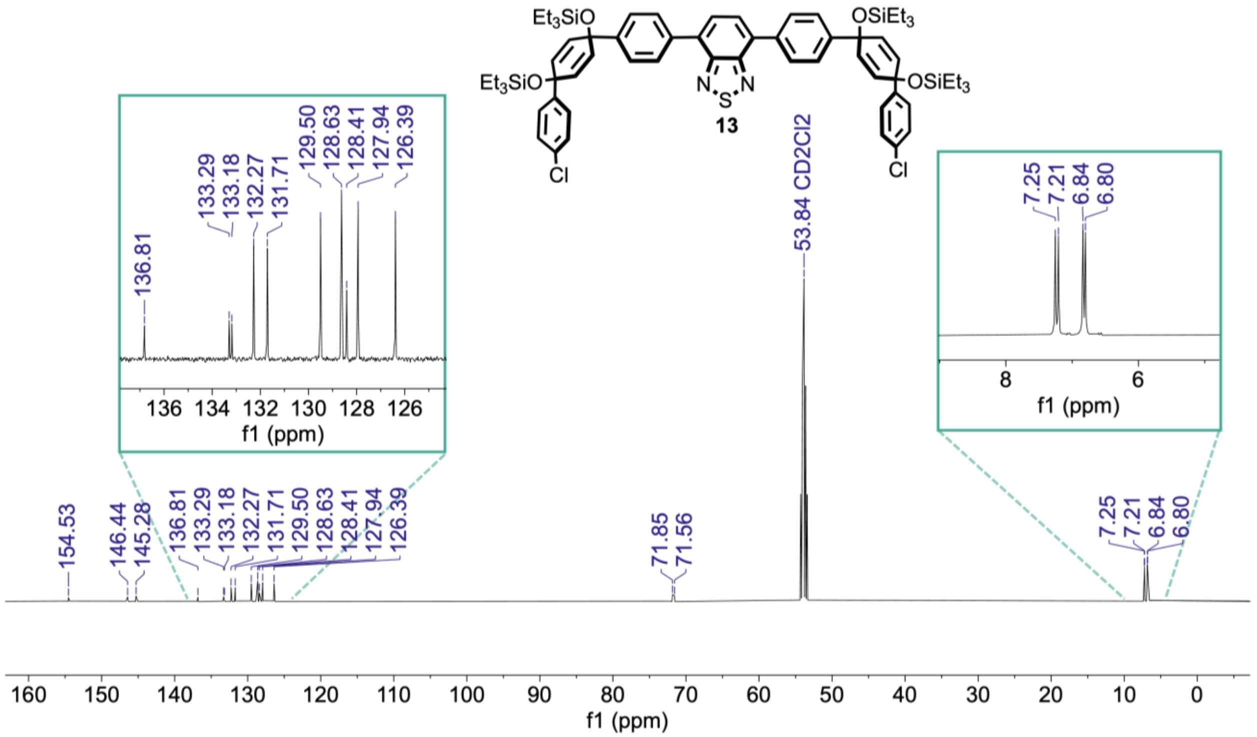

Figure S14. ^{13}C NMR spectrum (151 MHz) of **12** in Methylene Chloride-d_2 at room temperature.

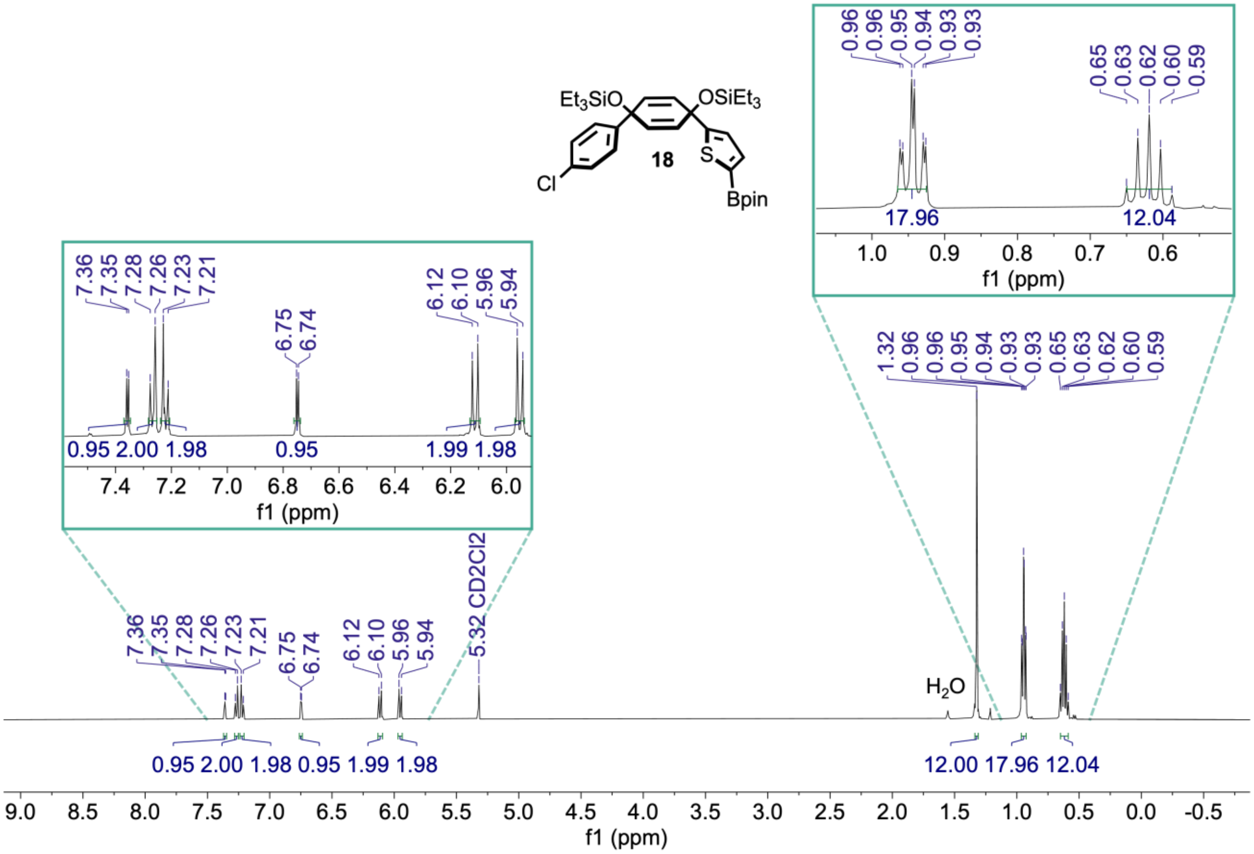

Figure S15. ^{1}H NMR spectrum (600 MHz) of **18** in Methylene Chloride-d_2 at room temperature.

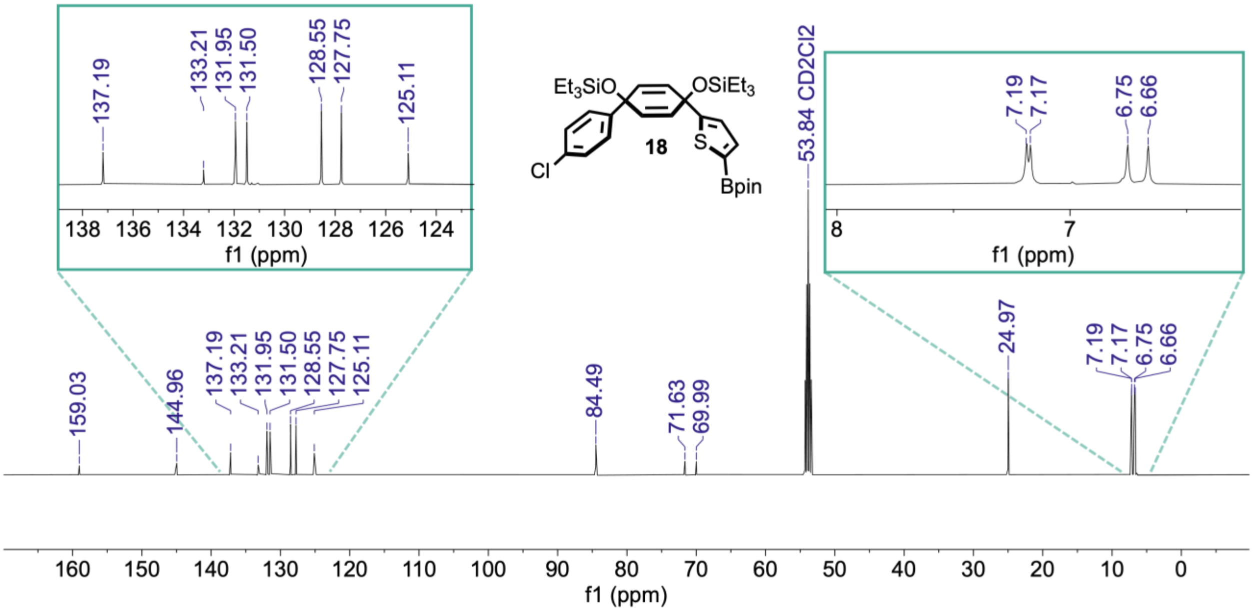

Figure S16. ^{13}C NMR spectrum (151 MHz) of **18** in Methylene Chloride-d_2 at room temperature.

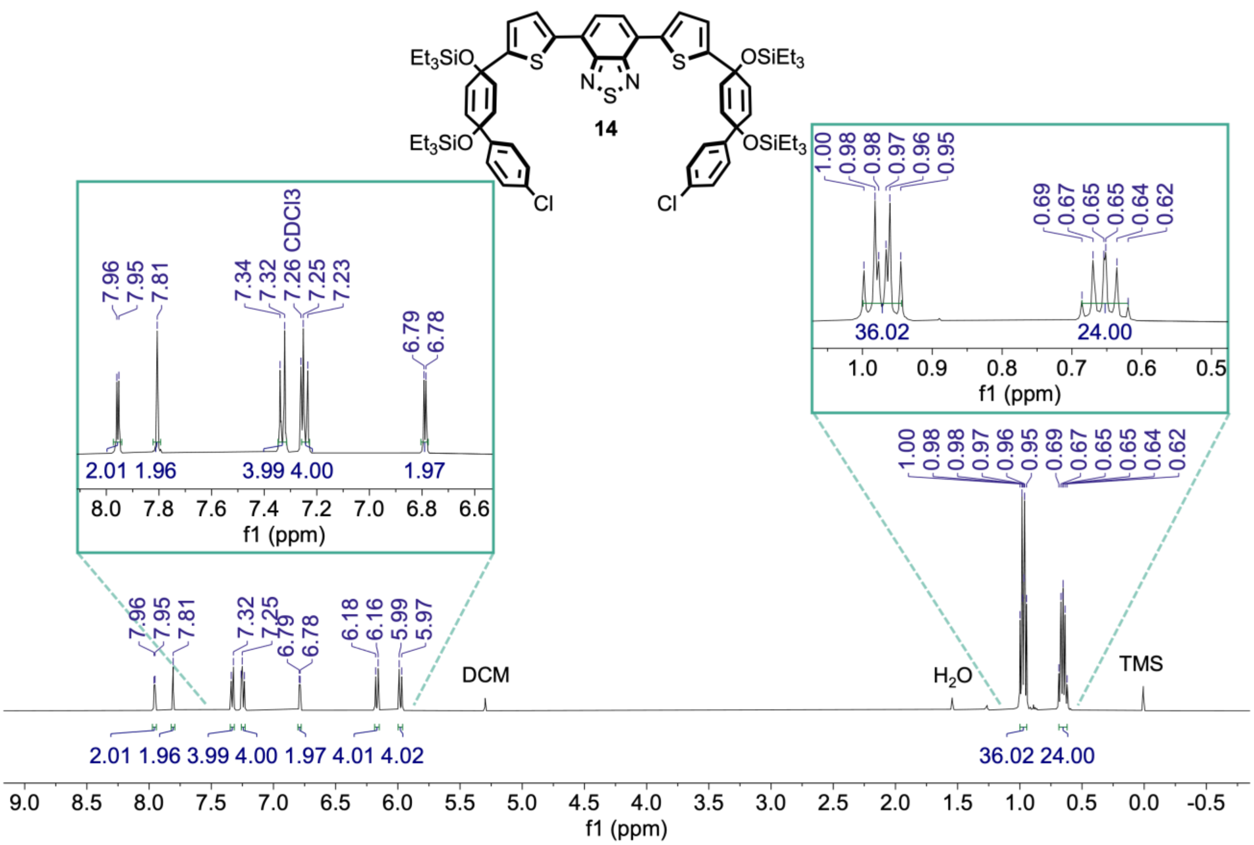

Figure S17. ^{1}H NMR spectrum (500 MHz) of **14** in Chloroform-*d* at room temperature.

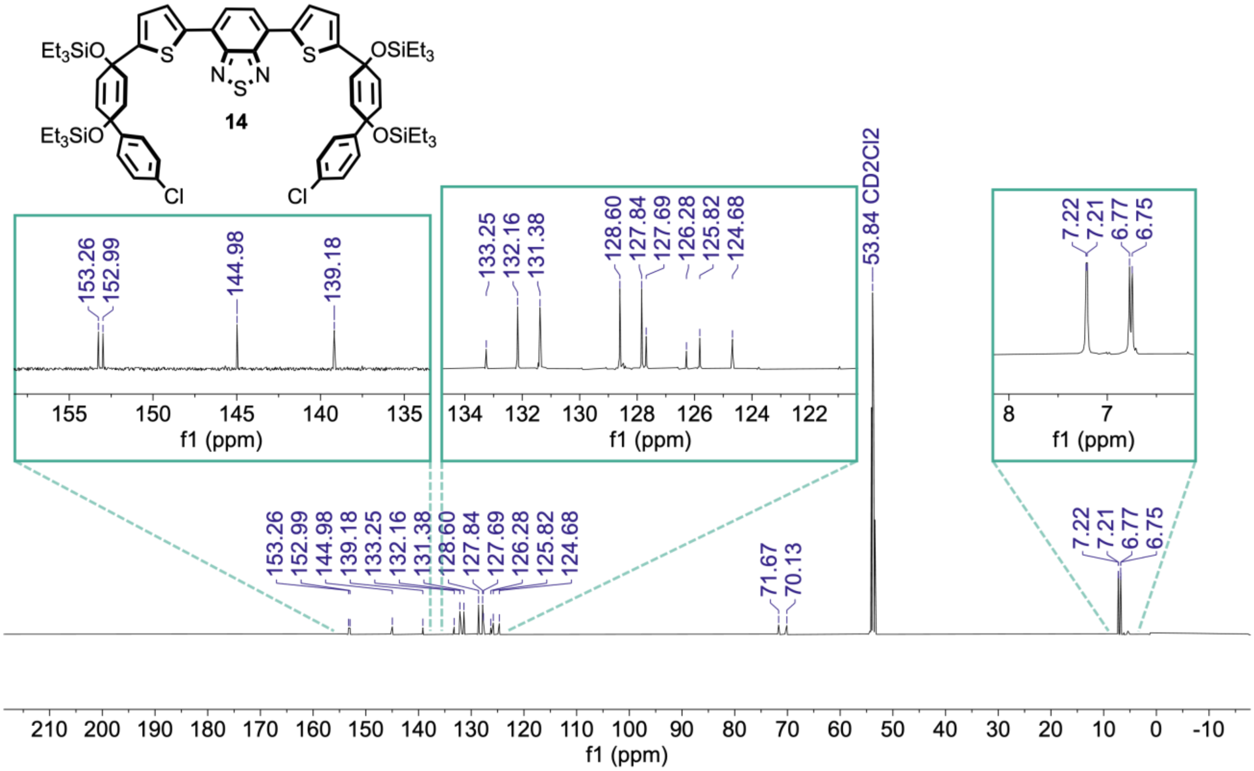

Figure S18. ^{13}C NMR spectrum (126 MHz) of **14** in Methylene Chloride-d_2 at room temperature.

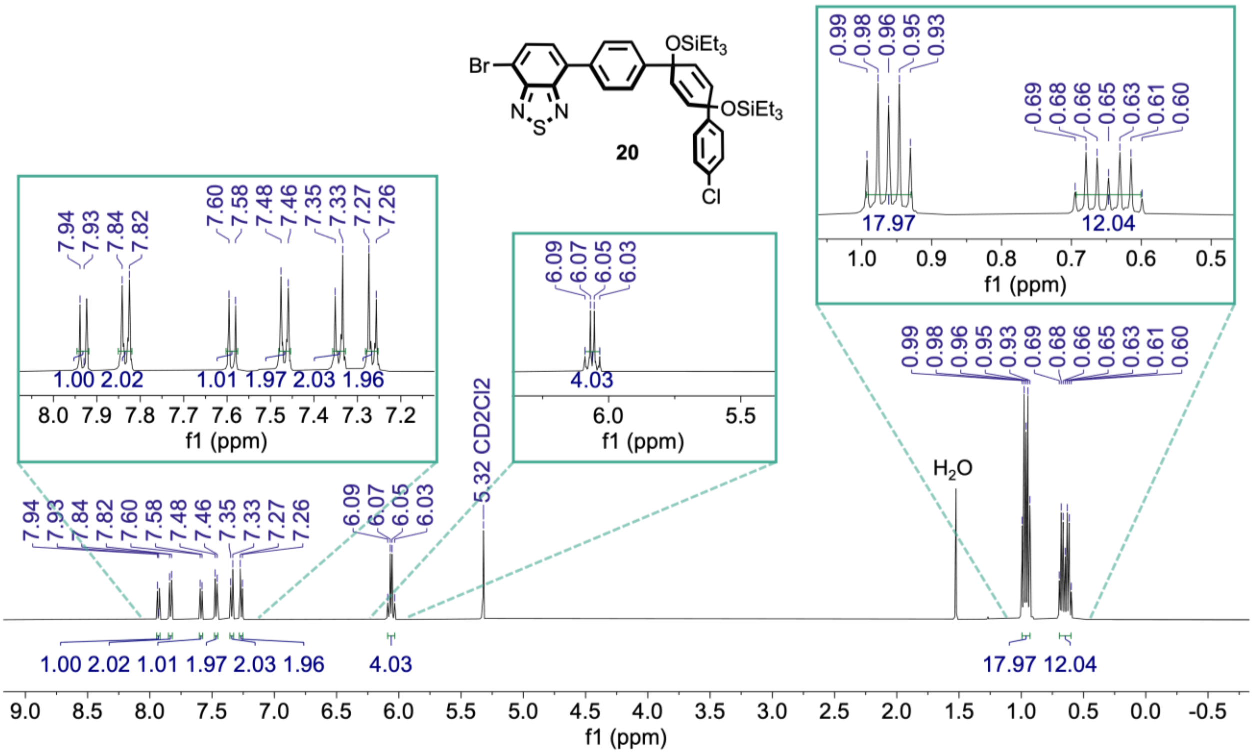

Figure S19. ^{1}H NMR spectrum (600 MHz) of **20** in Methylene Chloride-d_2 at room temperature.

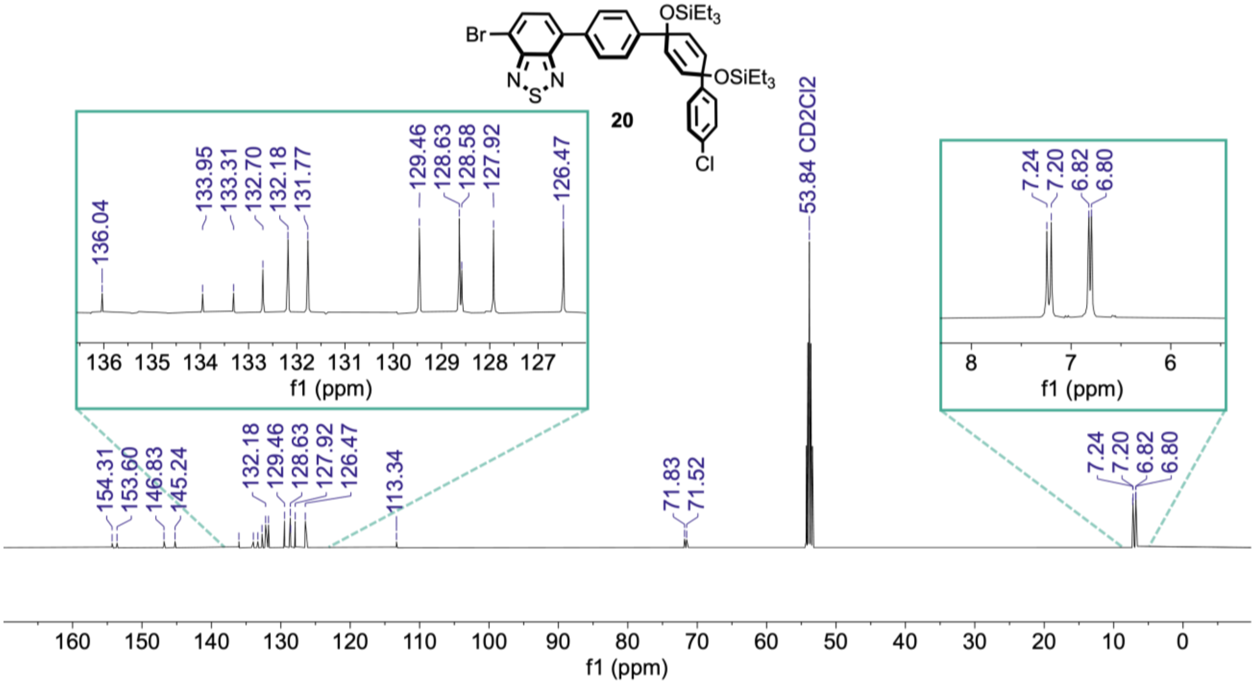

Figure S20. ^{13}C NMR spectrum (151 MHz) of **20** in Methylene Chloride-d_2 at room temperature.

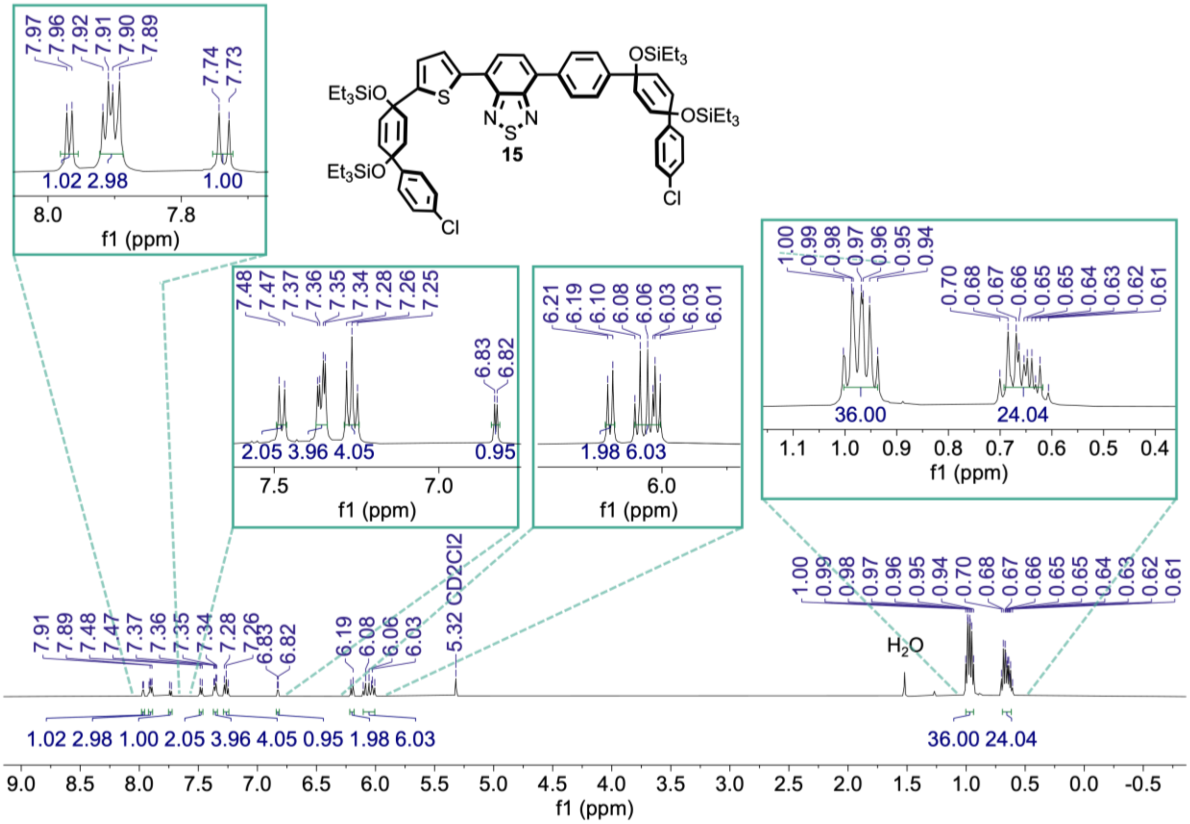

Figure S21. ^{1}H NMR spectrum (600 MHz) of **15** in Methylene Chloride-d_2 at room temperature.

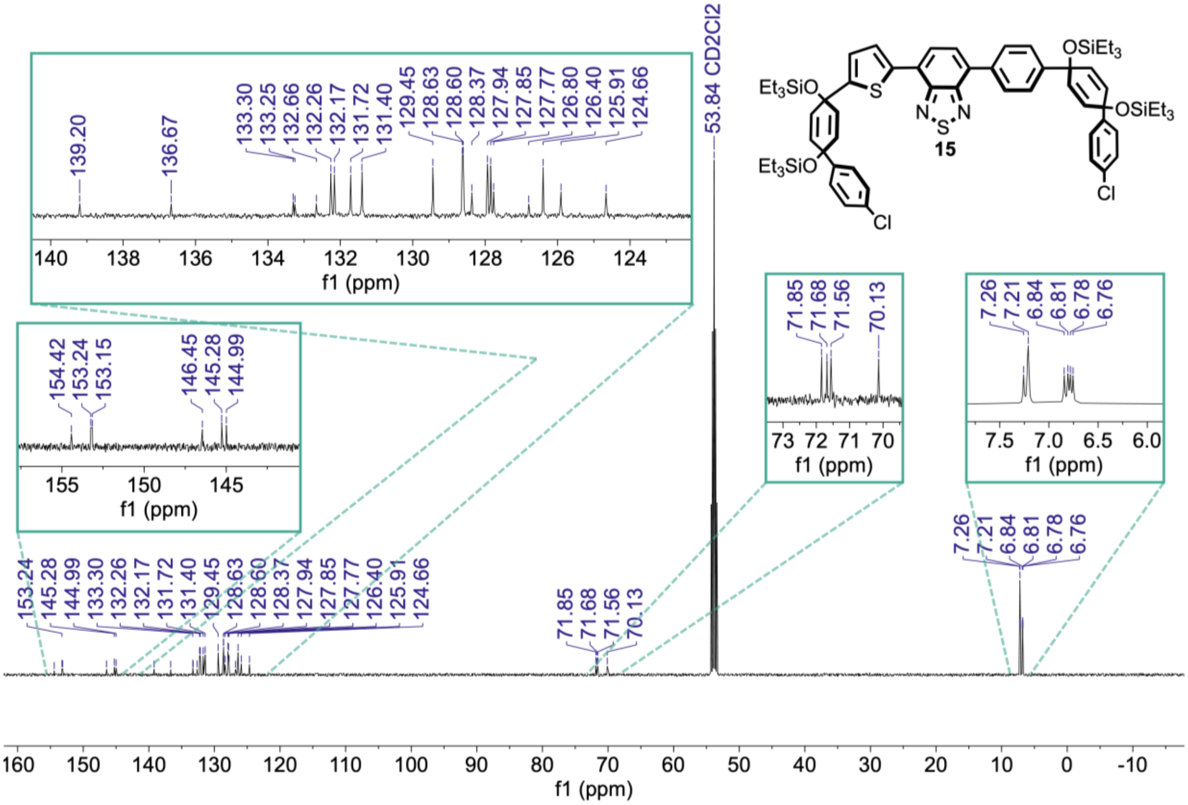

Figure S22. ^{13}C NMR spectrum (151 MHz) of **15** in Methylene Chloride-d_2 at room temperature.

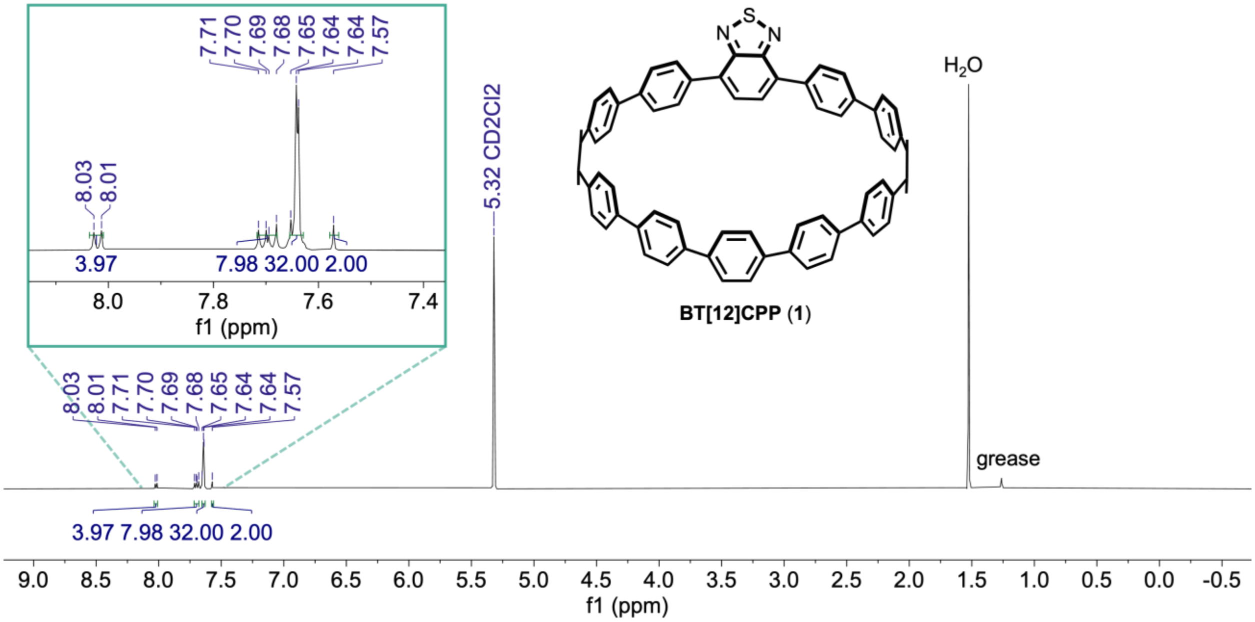

Figure S23. ^{1}H NMR spectrum (600 MHz) of **1** in Methylene Chloride-d_2 at room temperature.

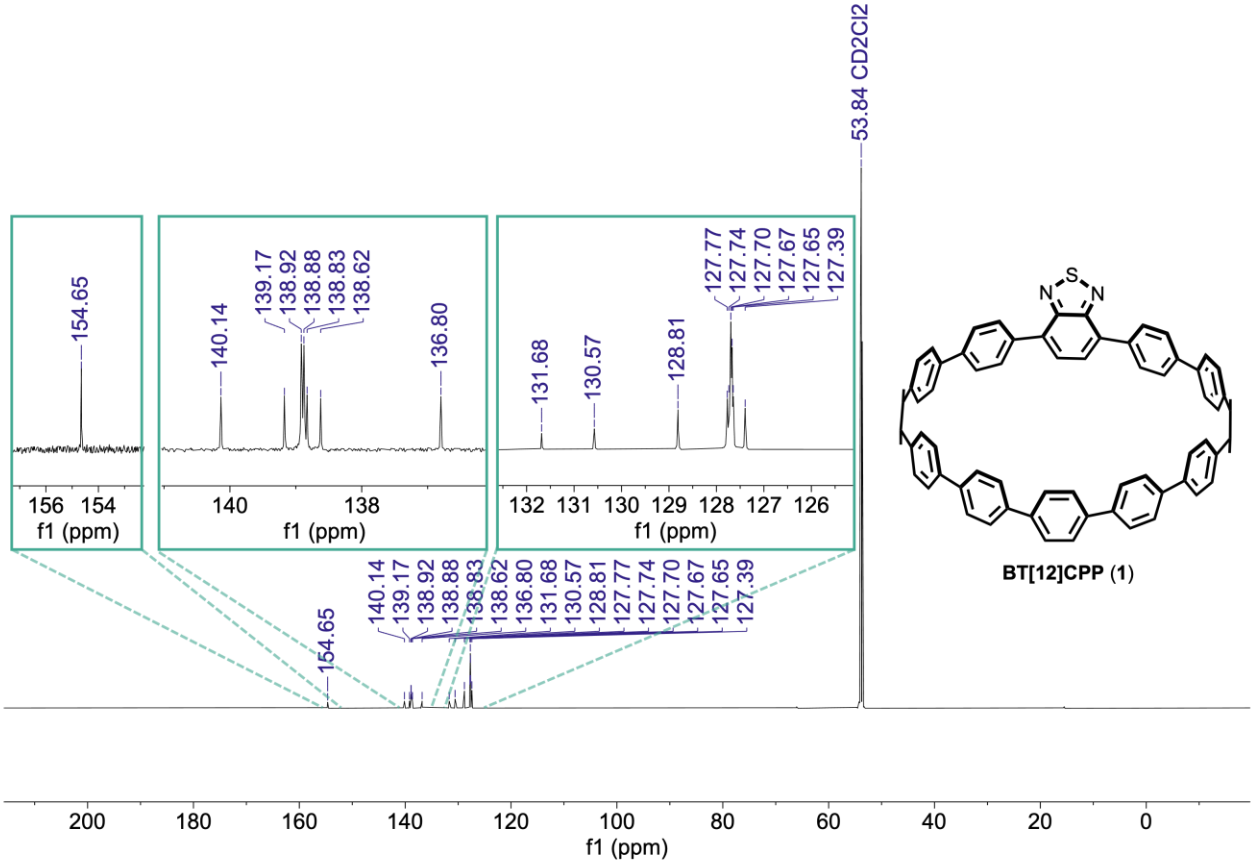

Figure S24. ^{13}C NMR spectrum (151 MHz) of **1** in Methylene Chloride-d_2 at room temperature.

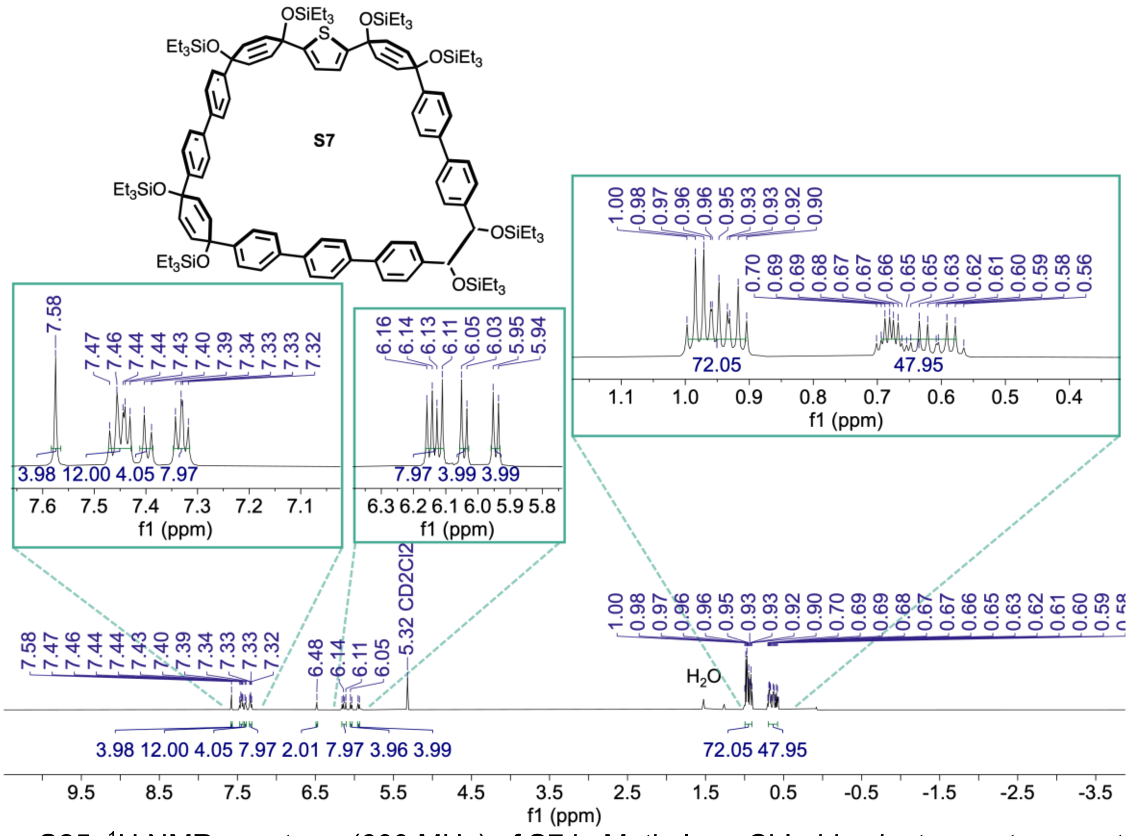

Figure S25. ^{1}H NMR spectrum (600 MHz) of **S7** in Methylene Chloride-d_2 at room temperature.

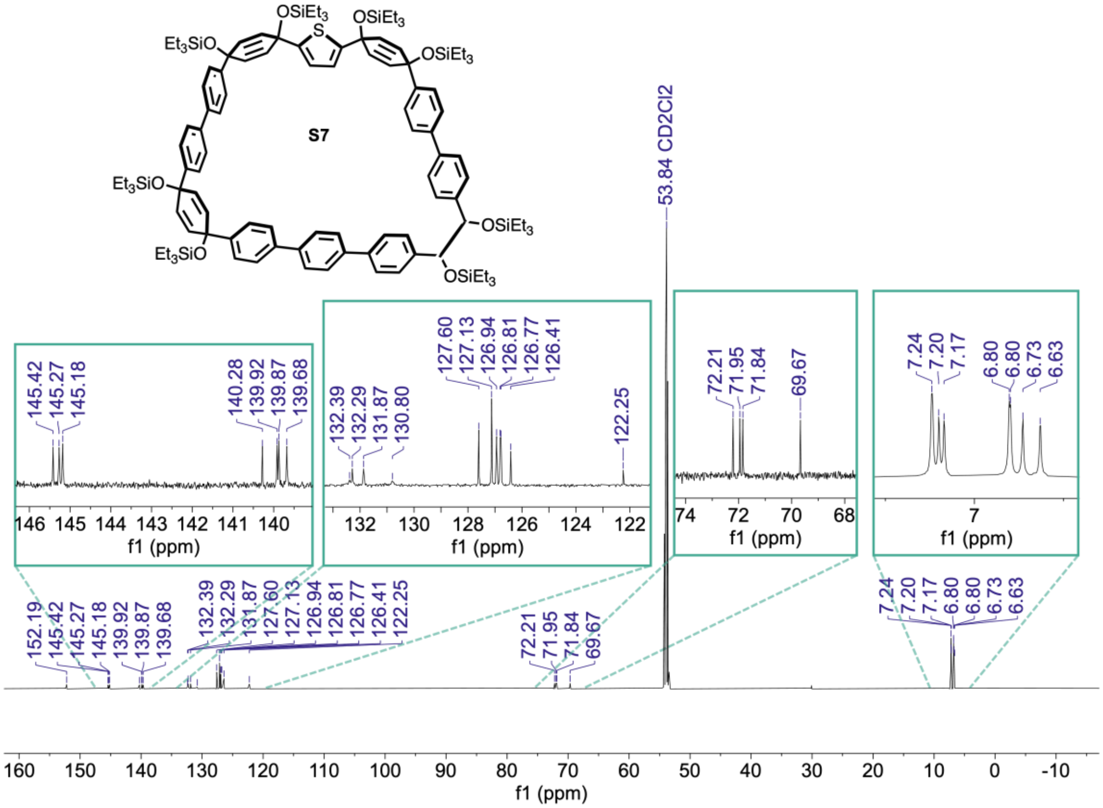

Figure S26. ^{13}C NMR spectrum (151 MHz) of **S7** in Methylene Chloride-d_2 at room temperature.

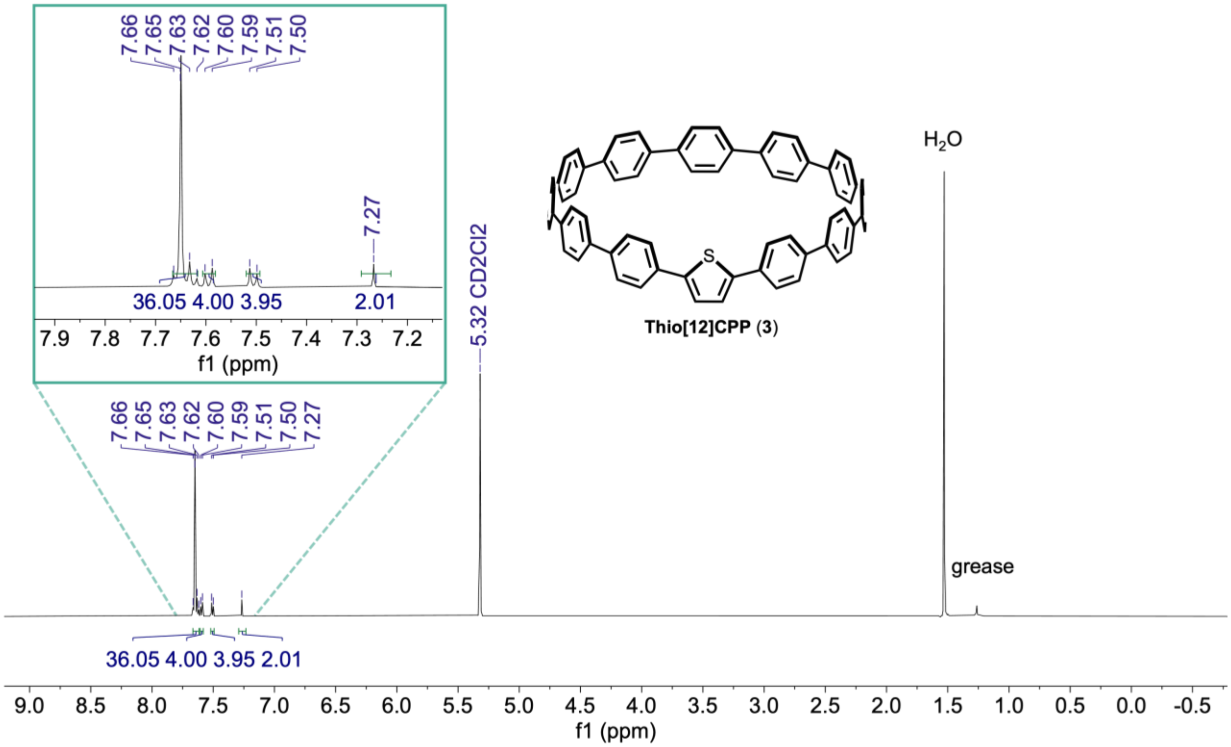

Figure S27. ^{1}H NMR spectrum (600 MHz) of **3** in Methylene Chloride-d_2 at room temperature.

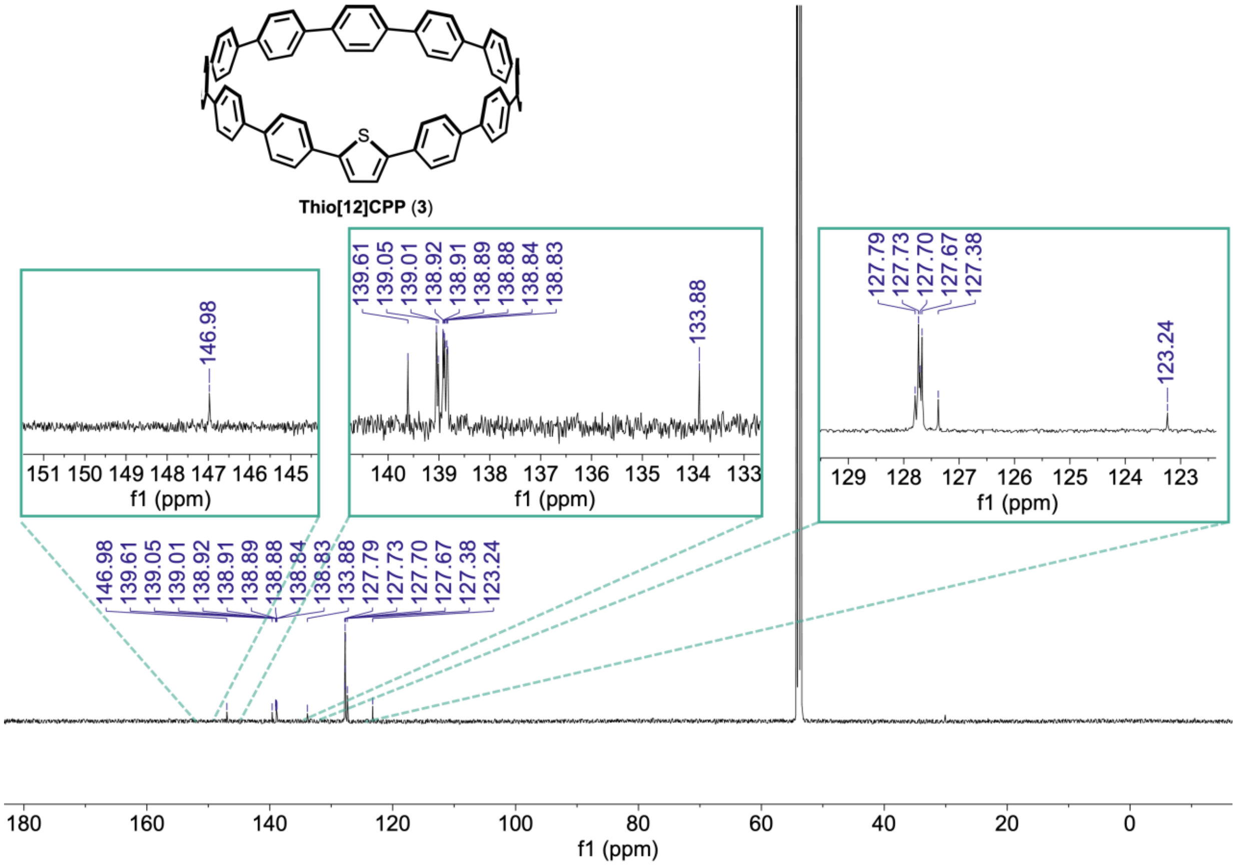

Figure S28. ^{13}C NMR spectrum (151 MHz) of **3** in Methylene Chloride-d_2 at room temperature.

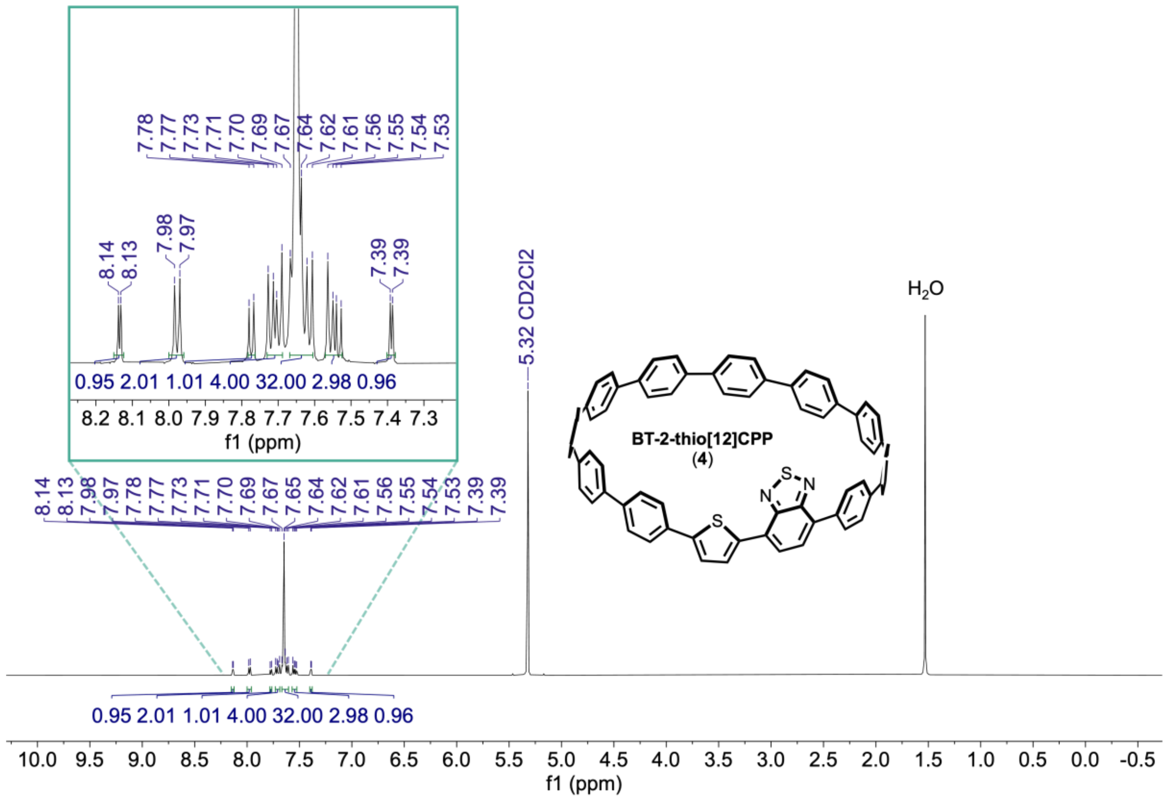

Figure S29. ^{1}H NMR spectrum (600 MHz) of **4** in Methylene Chloride-d_2 at room temperature.

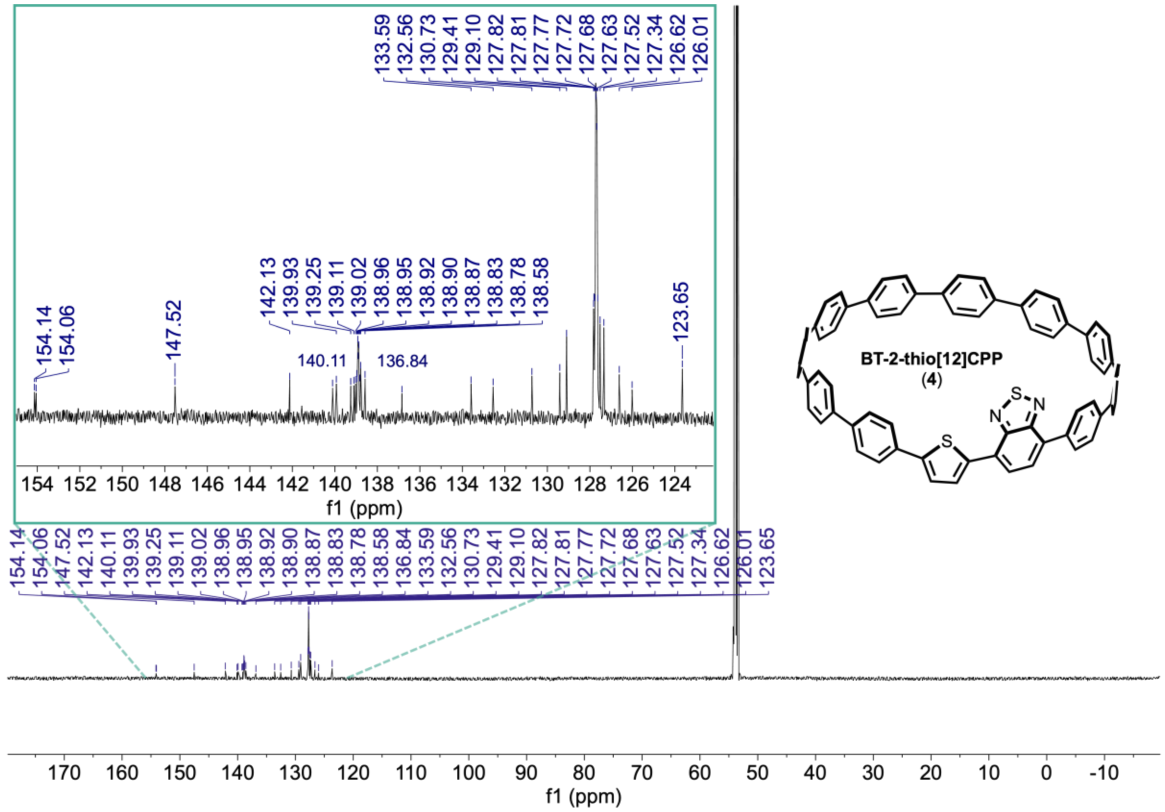

Figure S30. ^{13}C NMR spectrum (151 MHz) of **4** in Methylene Chloride-d_2 at room temperature.

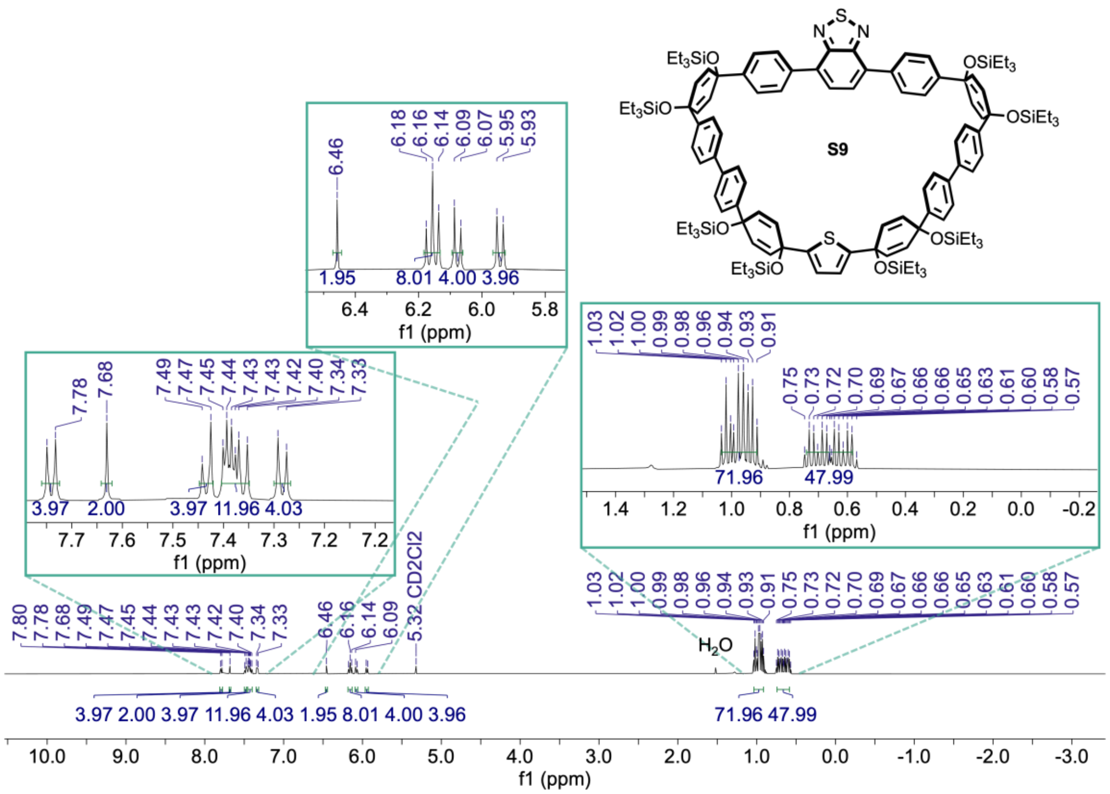

Figure S31. ^{1}H NMR spectrum (600 MHz) of **S9** in Methylene Chloride-d_2 at room temperature.

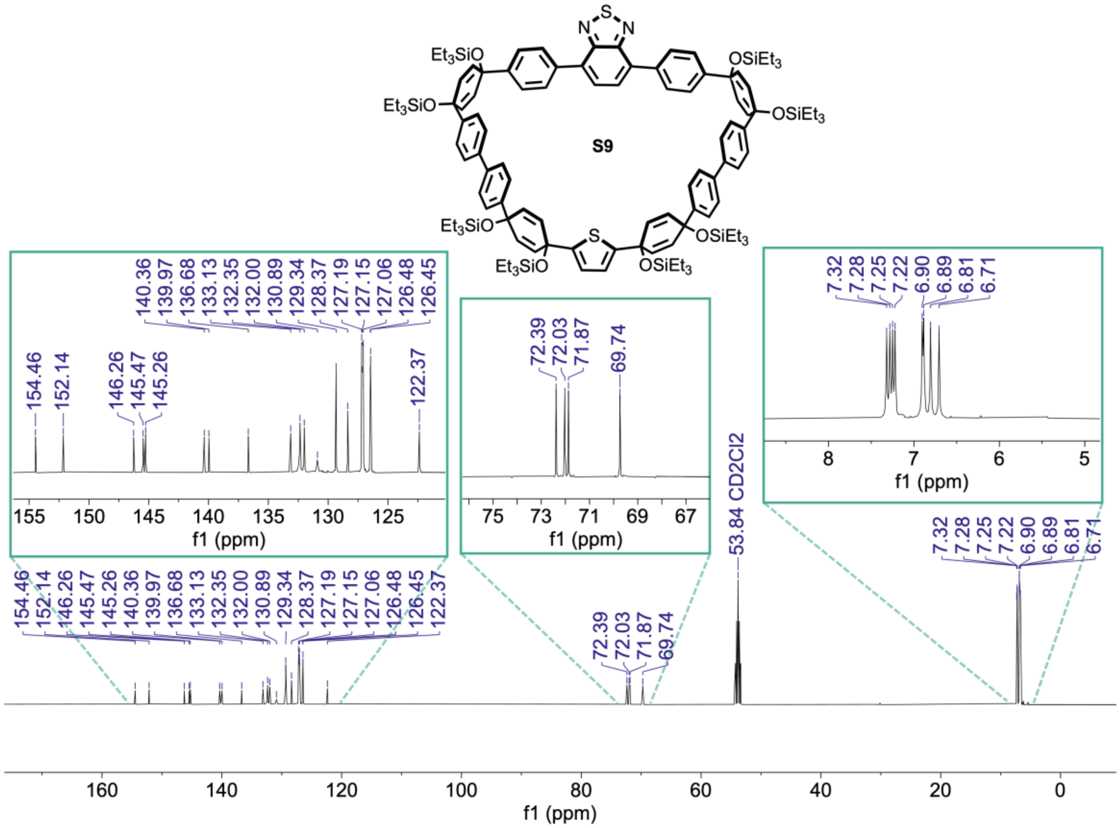

Figure S32. ^{13}C NMR spectrum (151 MHz) of **S9** in Methylene Chloride-d_2 at room temperature.

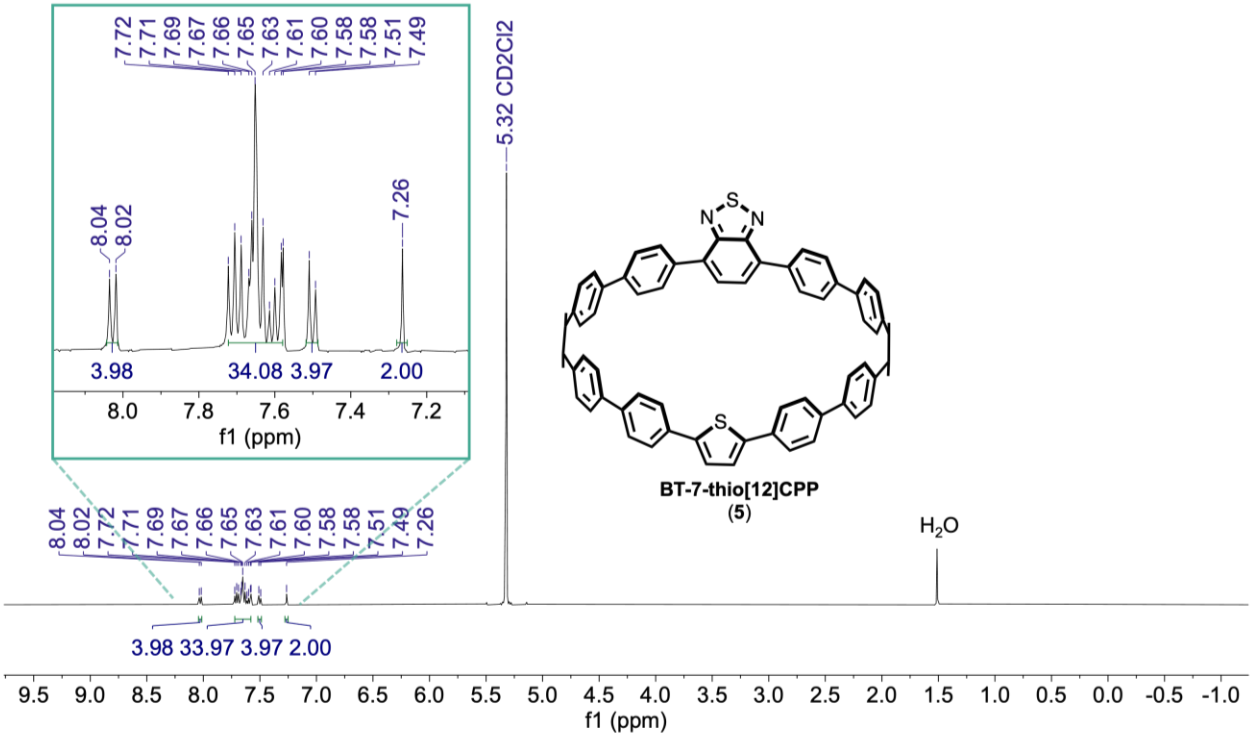

Figure S33. ^{1}H NMR spectrum (500 MHz) of **5** in Methylene Chloride-d_2 at room temperature.

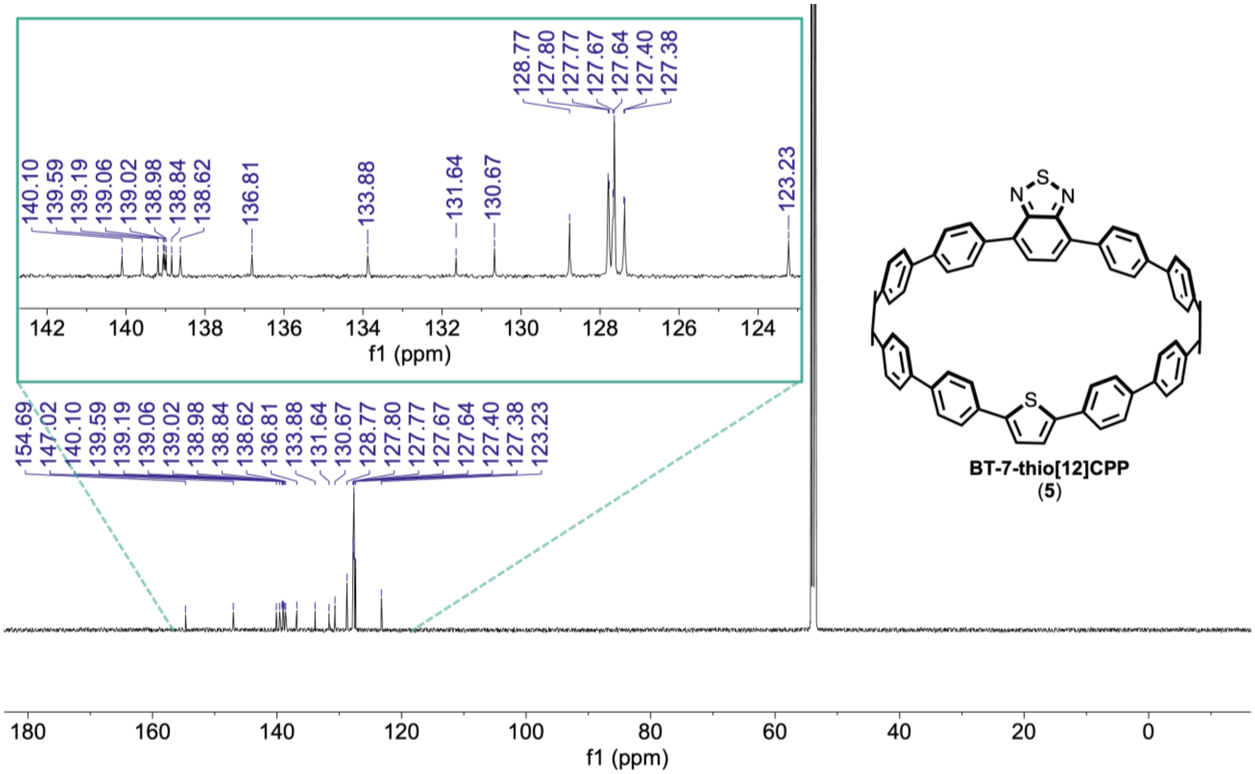

Figure S34. ^{13}C NMR spectrum (151 MHz) of **5** in Methylene Chloride-d_2 at room temperature.

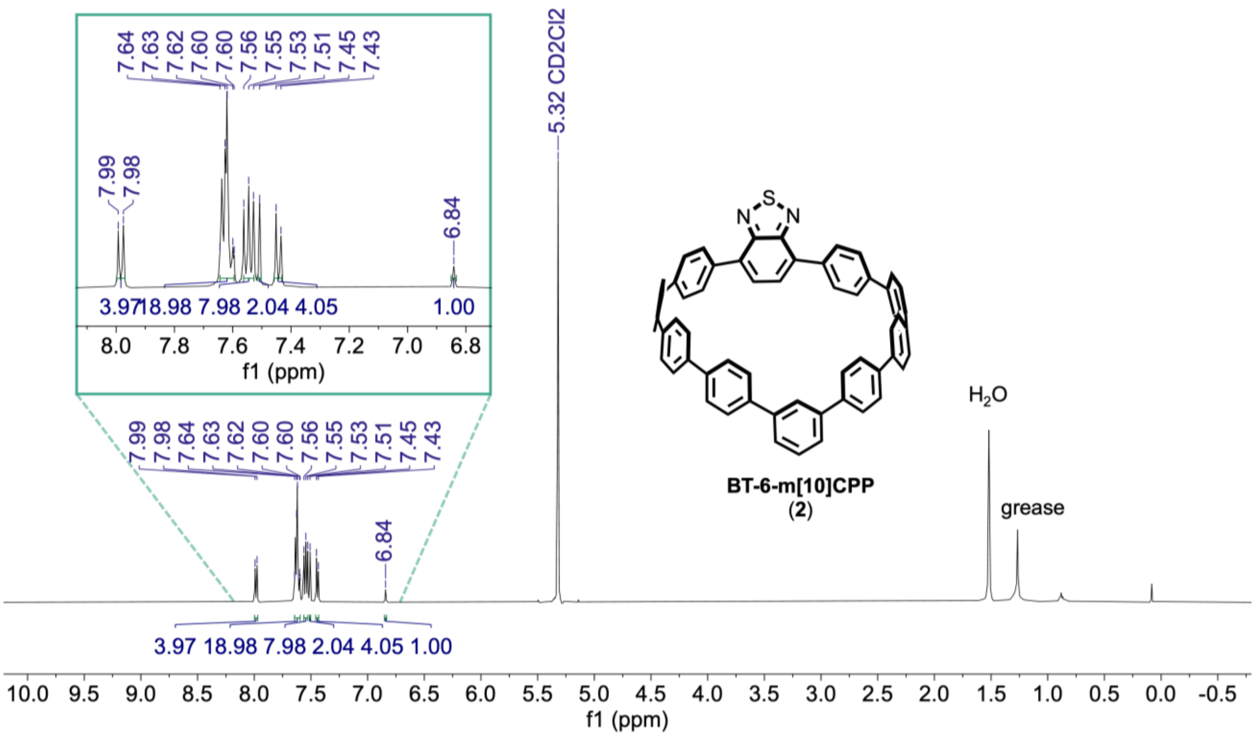

Figure S35. ^{1}H NMR spectrum (500 MHz) of **2** in Methylene Chloride-d_2 at room temperature.

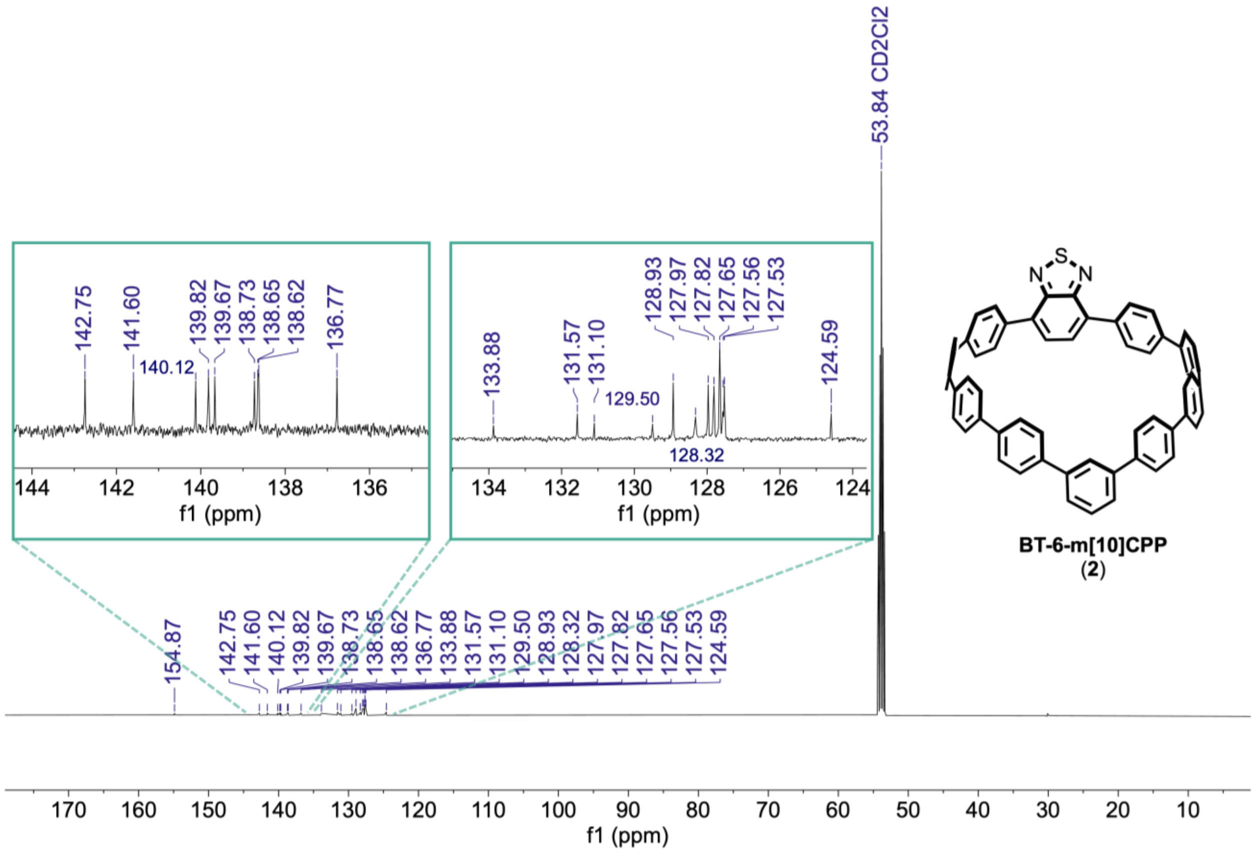

Figure S36. ^{13}C NMR spectrum (126 MHz) of **2** in Methylene Chloride-d_2 at room temperature.

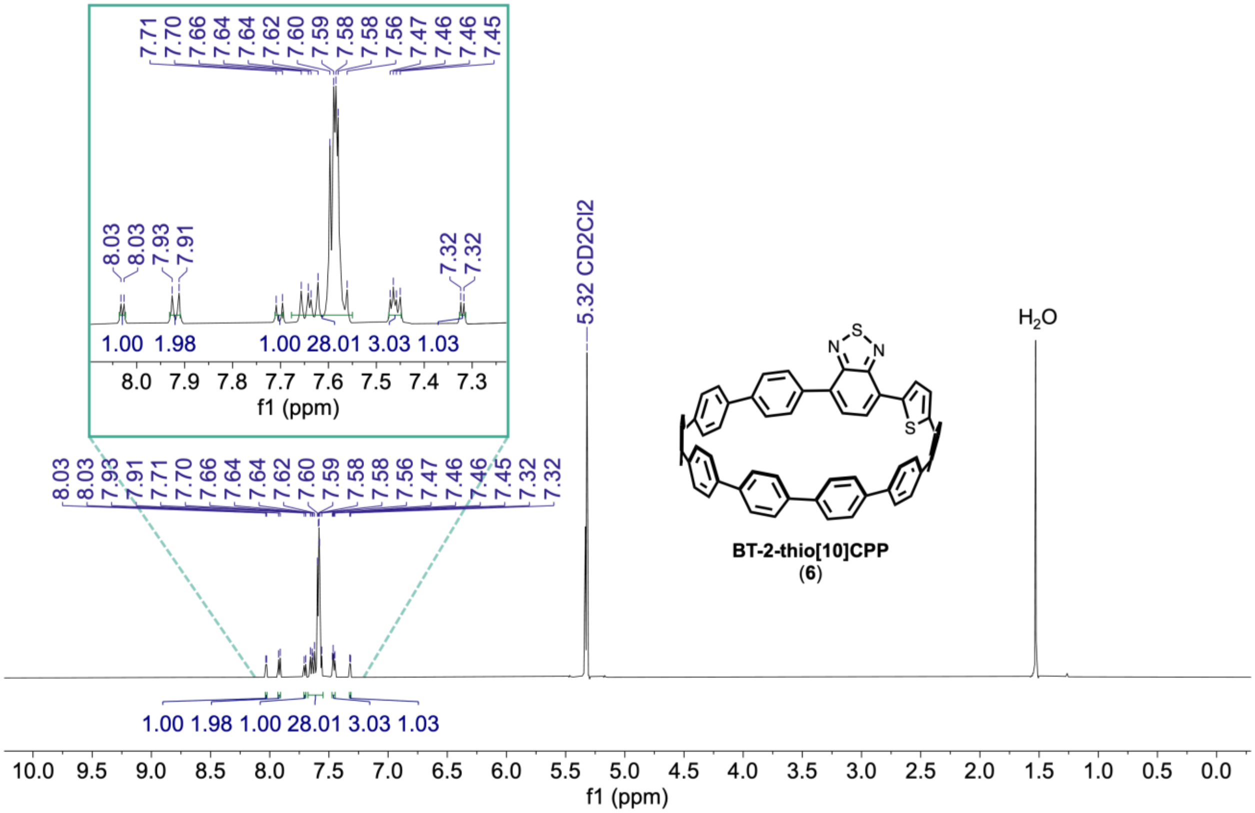

Figure S37. ^{1}H NMR spectrum (600 MHz) of **6** in Methylene Chloride-d_2 at room temperature.

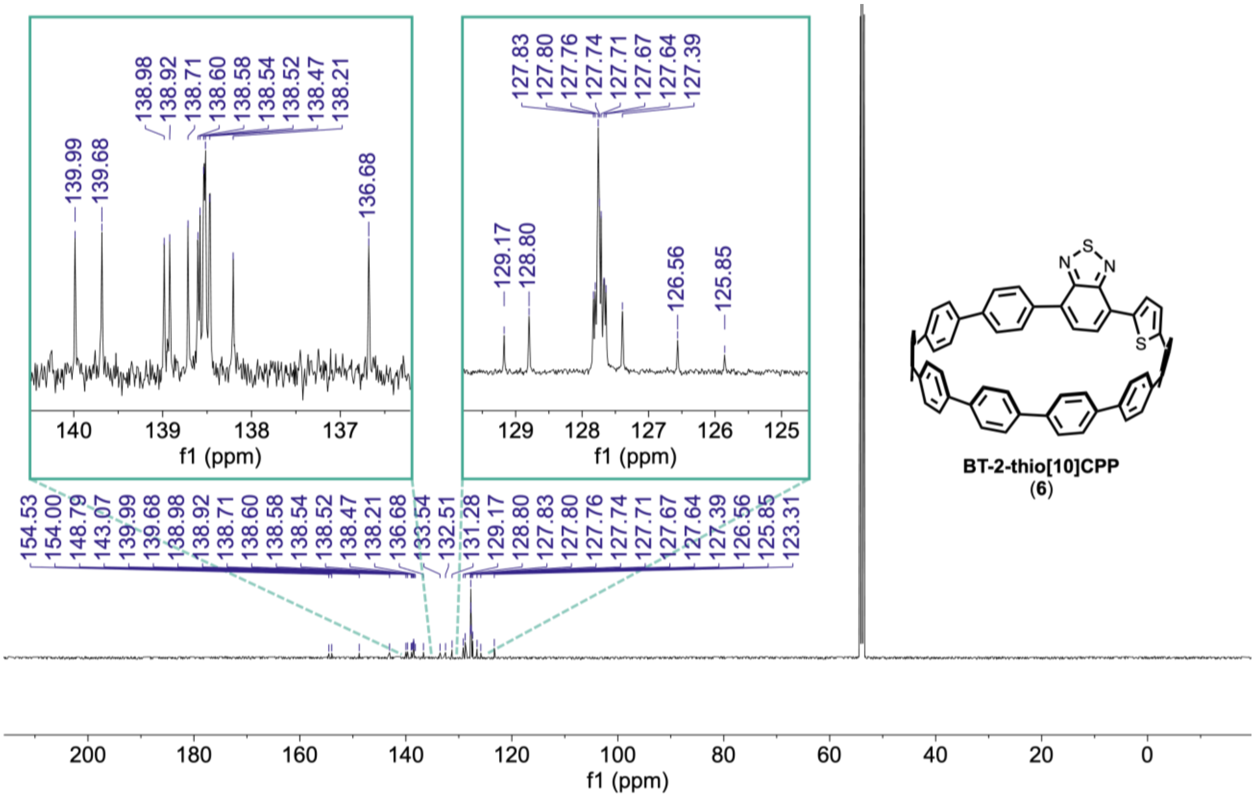

Figure S38. ^{13}C NMR spectrum (151 MHz) of **6** in Methylene Chloride-d_2 at room temperature.

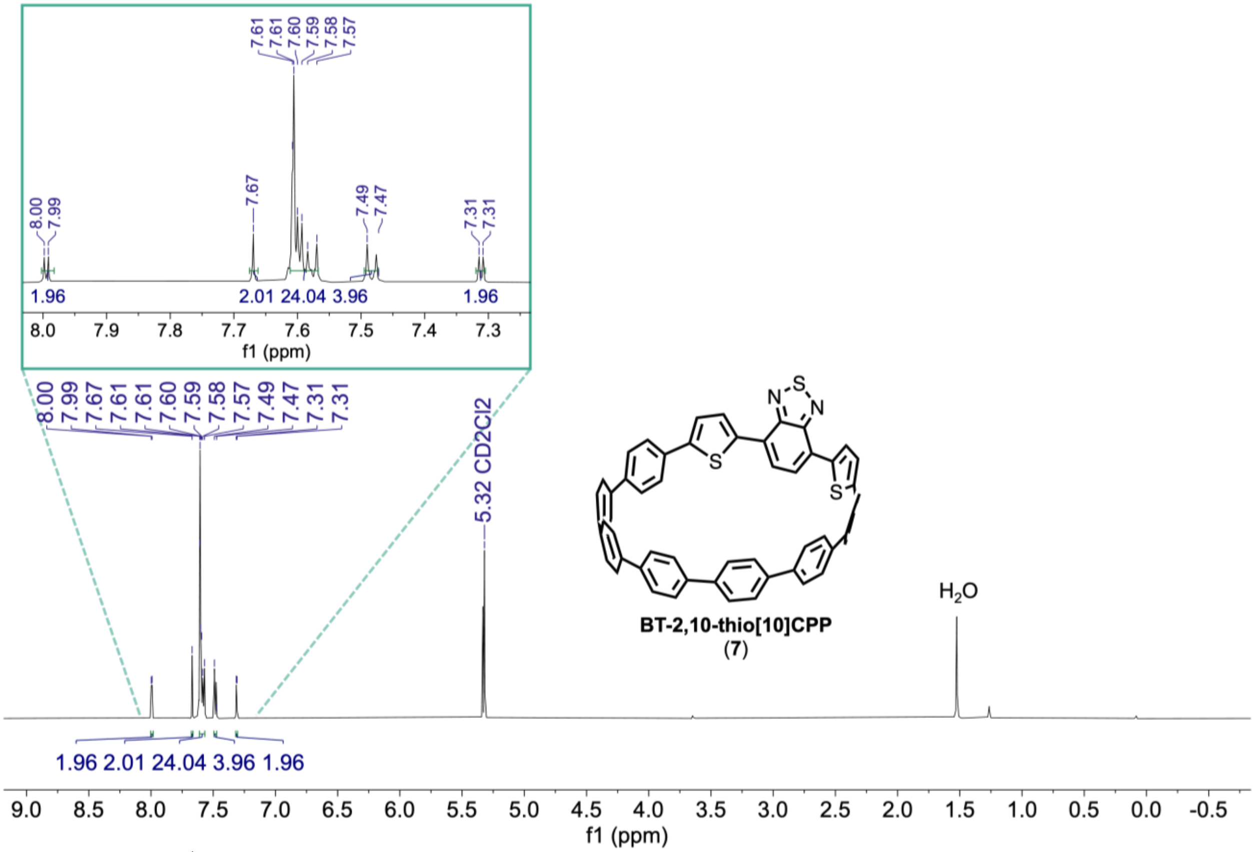

Figure S39. ^{1}H NMR spectrum (600 MHz) of **7** in Methylene Chloride-d_2 at room temperature.

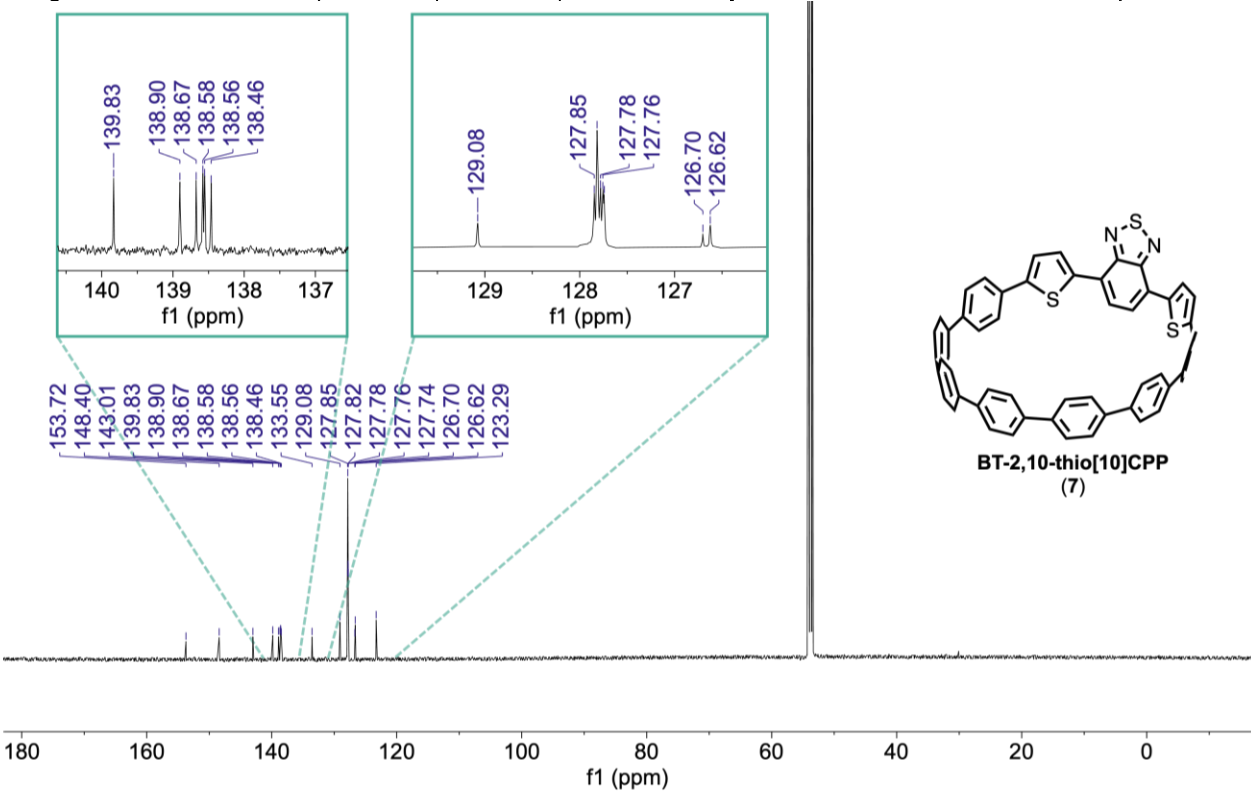

Figure S40. ^{13}C NMR spectrum (151 MHz) of **7** in Methylene Chloride-d_2 at room temperature.

4. Experimental and Computational Photophysical Characterization

a. Experimental Photophysical Characterization

Quantum Yield Data

Quantum yield measurements were performed using the method described in "A Guide to Recording Fluorescence Quantum Yields" by Horiba Scientific.[9] The internal standards used were quinine sulfate (0.1 M H_2SO_4 aq., lit. value Φ = 0.60, fluorescence signal integrated from 400-600 nm) and anthracene (ethanol, lit. value Φ = 0.27, fluorescence signal integrated from 360-480 nm). Absorbance values plotted below were measured at 340 nm. For fluorescence measurements, all compounds were excited at a wavelength of 340 nm with consistent excitation and emission slit widths of 1 nm. Fluorescence signal integrations for compounds of interest are listed below:

Note: A second order diffraction peak was observed at 680 nm - two times the excitation wavelength (340 nm x 2); therefore, did not integrate ± 5 nm from this region.

Table S1. Compound integration ranges in DCM and DMSO.

Compound	Integration Range in DCM (nm)	Integration Range in DMSO (nm)
1	480 - 675	500 - 675 | 685 - 700
2	500 - 675 | 685 - 720	510 - 675 | 685 - 730
3	420 - 600	420 - 600
4	505 - 675 | 685 - 740	530 - 675 | 685 - 750
5	480 - 675 | 685 - 700	500 - 675 | 685 - 730
6	520 - 675 | 685 - 750	550 - 675 | 685 - 770
7	550 - 675 | 685 - 760	570 - 675 | 685 - 780

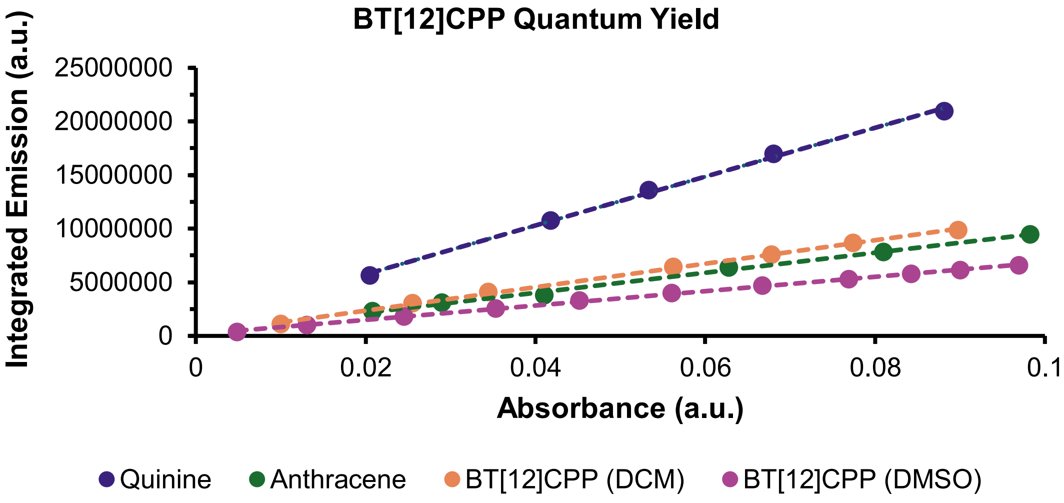

Figure S41. Quantum yield plot for **1**.

Table S2: Quantum yield data for **1** (BT[12]CPP).

Compound	Solvent	Slope	ϕ w.r.t. quinine	ϕ w.r.t. anthracene	Avg.	Std. dev.
Quinine	0.1 M H_2SO_4 (aq.)	2.28×10^8	0.6 (lit.)	0.63	-	-
Anthracene	EtOH	9.35×10^7	0.26	0.27 (lit.)	-	-
1	DCM	1.09×10^8	0.35	0.33	0.34	0.012
1	DMSO	6.70×10^7	0.23	0.22	0.22	0.0079

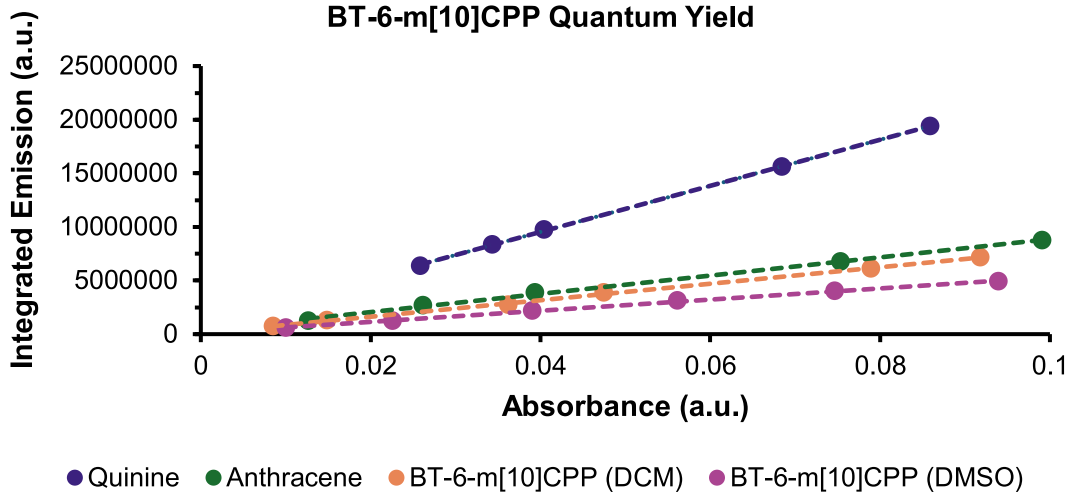

Figure S42. Quantum yield plot for **2**.

Table S3: Quantum yield data for **2** (BT-6-m[10]CPP).

Compound	Solvent	Slope	ϕ w.r.t. quinine	ϕ w.r.t. anthracene	Avg.	Std. dev.
Quinine	0.1 M H_2SO_4 (aq.)	$2.15x10^8$	0.6 (lit.)	0.65	-	-
Anthracene	EtOH	$8.51x10^7$	0.25	0.27 (lit.)	-	-
2	DCM	$7.69x10^7$	0.27	0.25	0.26	0.016
2	DMSO	$5.20x10^7$	0.19	0.18	0.19	0.011

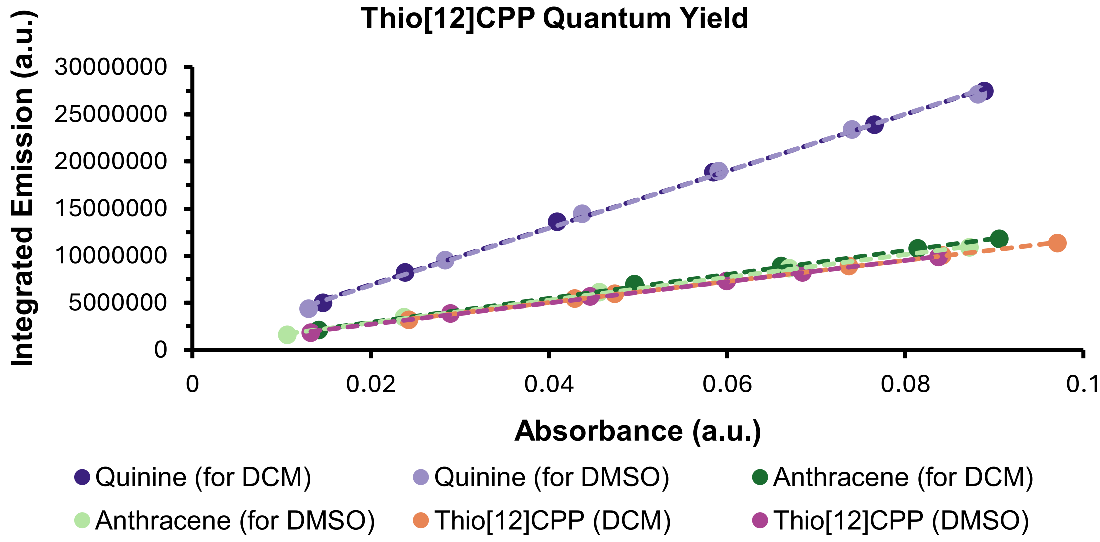

Figure S43. Quantum yield plot for **3**.

Table S4: Quantum yield data for **3** (Thio[12]CPP).

Compound	Solvent	Slope	ϕ w.r.t. quinine	ϕ w.r.t. anthracene	Avg.	Std. dev.
Quinine (for DCM)	0.1 M H_2SO_4 (aq.)	$3.01x10^8$	0.6 (lit.)	0.61	-	-
Anthracene (for DCM)	EtOH	$1.27x10^8$	0.26	0.27 (lit.)	-	-
3	DCM	$1.12x10^8$	0.26	0.25	0.26	0.0044
Quinine (for DMSO)	0.1 M H_2SO_4 (aq.)	$3.03x10^8$	0.6 (lit.)	0.65	-	-
Anthracene (for DMSO)	EtOH	$1.21x10^8$	0.25	0.27 (lit.)	-	-
3	DMSO	$1.13x10^8$	0.30	0.28	0.29	0.016

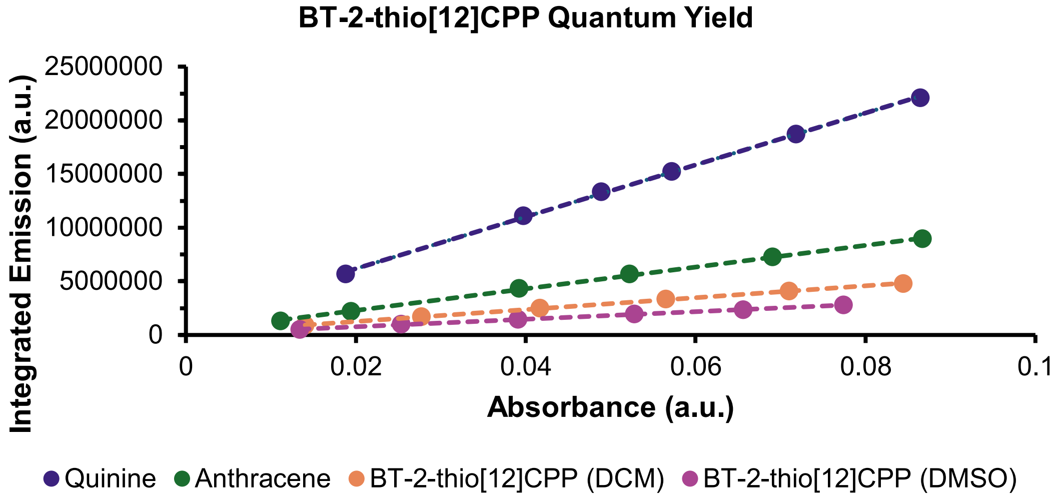

Figure S44. Quantum yield plot for **4**.

Table S5: Quantum yield data for **4** (BT-2-thio[12]CPP).

Compound	Solvent	Slope	ϕ w.r.t. quinine	ϕ w.r.t. anthracene	Avg.	Std. dev.
Quinine	0.1 M H_2SO_4 (aq.)	$2.42x10^8$	0.6 (lit.)	0.61	-	-
Anthracene	EtOH	$1.03x10^8$	0.27	0.27 (lit.)	-	-
4	DCM	$5.54x10^7$	0.16	0.16	0.16	0.0011
4	DMSO	$3.52x10^7$	0.11	0.11	0.11	0.00078

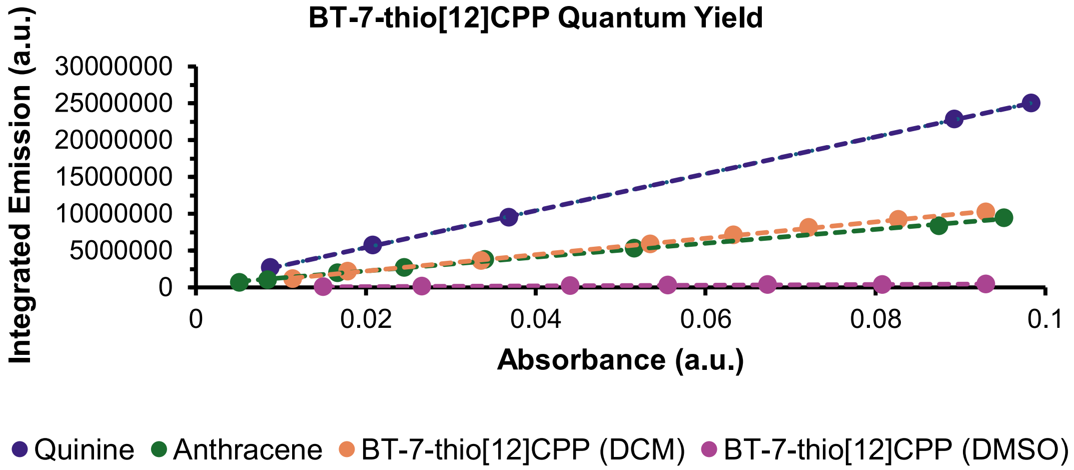

Figure S45. Quantum yield plot for **5**.

Table S6: Quantum yield data for **5** (BT-7-thio[12]CPP).

Compound	Solvent	Slope	ϕ w.r.t. quinine	ϕ w.r.t. anthracene	Avg.	Std. dev.
Quinine	0.1 M H_2SO_4 (aq.)	$2.50x10^8$	0.6 (lit.)	0.61	-	-
Anthracene	EtOH	$1.06x10^8$	0.27	0.27 (lit.)	-	-
5	DCM	$1.11x10^8$	0.31	0.30	0.31	0.0033
5	DMSO	$4.83x10^6$	0.01	0.01	0.01	0.00016

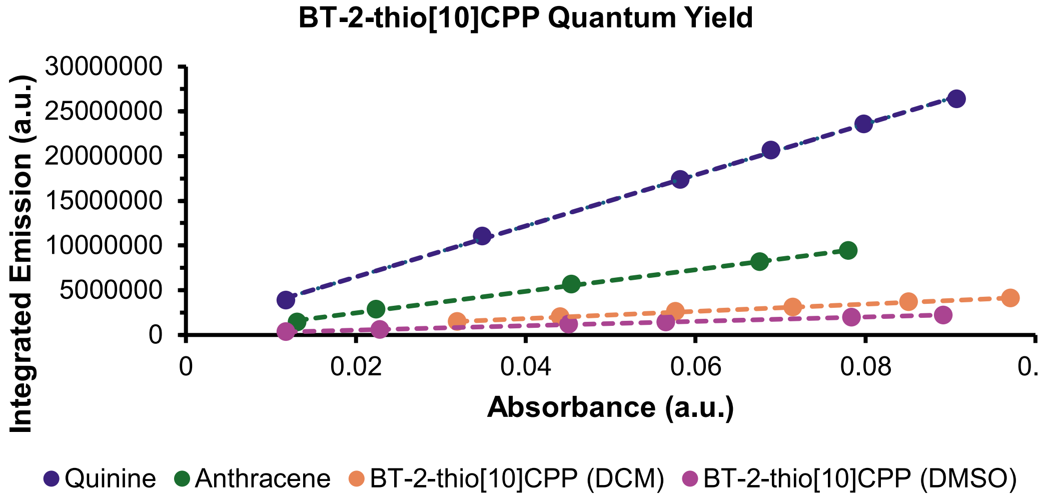

Figure S46. Quantum yield plot for **6**.

Table S7: Quantum yield data for **6** (BT-2-thio[10]CPP).

Compound	Solvent	Slope	ϕ w.r.t. quinine	ϕ w.r.t. anthracene	Avg.	Std. dev.
Quinine	0.1 M H_2SO_4 (aq.)	2.85×10^8	0.6 (lit.)	0.61	-	-
Anthracene	EtOH	1.21×10^8	0.27	0.27 (lit.)	-	-
6	DCM	4.05×10^7	0.10	0.10	0.10	0.0013
6	DMSO	2.45×10^7	0.06	0.06	0.06	0.00085

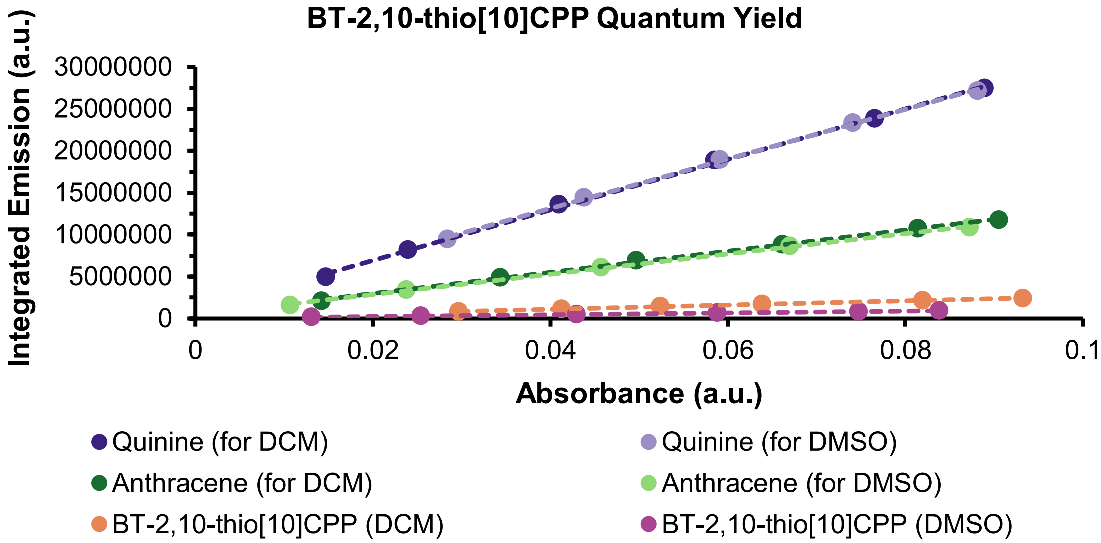

Figure S47. Quantum yield plot for **7**.

Table S8: Quantum yield data for **7** (BT-2,10-thio[10]CPP).

Compound	Solvent	Slope	ϕ w.r.t. quinine	ϕ w.r.t. anthracene	Avg.	Std. dev.
Quinine (for DCM)	0.1 M H_2SO_4 (aq.)	3.01×10^8	0.6 (lit.)	0.62	-	-
Anthracene (for DCM)	EtOH	1.26×10^8	0.26	0.27 (lit.)	-	-
7	DCM	2.53×10^7	0.06	0.06	0.06	0.0012
Quinine (for DMSO)	0.1 M H_2SO_4 (aq.)	2.95×10^8	0.6 (lit.)	0.63	-	-
Anthracene (for DMSO)	EtOH	1.21×10^8	0.26	0.27 (lit.)	-	-
7	DMSO	1.09×10^7	0.03	0.03	0.03	0.001

Solvatofluorochromism Data

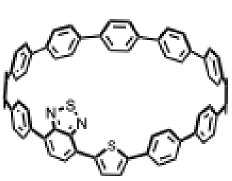

BT-2-thio[12]CPP (4)

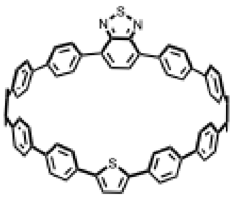

BT-7-thio[12]CPP (5)

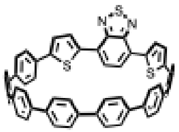

BT-2,10-thio[10]CPP (7)

Table S9: Tabulated solvatofluorochromism data for compounds **4**, **5**, and **7**.

Compound	Major λ_{abs} (nm) CCl$_4$, DCM, DMSO	Minor λ_{abs} (nm) CCl$_4$, DCM, DMSO	λ_{em} (nm) CCl$_4$, DCM, DMSO
**4**	340, 339, 348	467, 457, 470	575, 599, 622
**5**	340, 340, 349	-	550, 565, 587
**7**	342, 342, 350	512, 495, 510	613, 639, 662

The Lippert-Mataga **Equation (1)**[10] was used to plot the effective Stokes shift ($\Delta\nu$) in wavenumbers versus the orientation polarizability (Δf) of the solvent. The orientation polarizability was determined by using lit. known values for the refractive index (η) and the dielectric constant (ε) of the solvent. A large slope means a large dipole moment variation ($\Delta\mu$), which is the difference between the excited state dipole moment (μ_e) and the ground state dipole moment (μ_g).

Equation (1): $\Delta\nu = \frac{2(\Delta\mu)^2}{r^3 hc}\Delta f + C \text{ with } \Delta f = (\frac{\varepsilon-1}{2\varepsilon+1} - \frac{\eta^2-1}{\eta^2+1})$

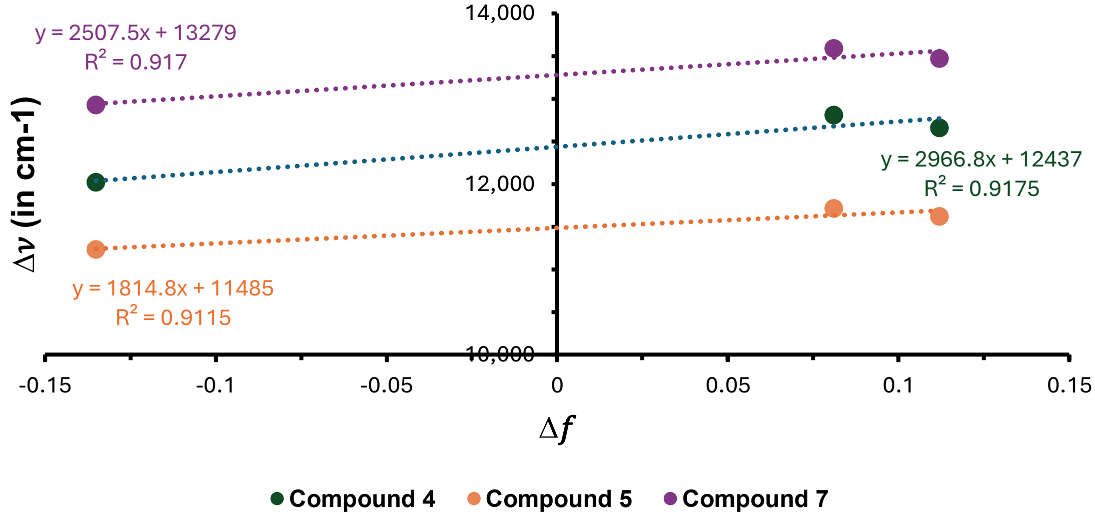

Figure S48. Lippert-Mataga plot for compounds **4**, **5**, and **7**.

Table S10: Tabulated solvatofluorochromism data for compounds **4**, **5**, and **7**.

Compound	$\Delta\nu$ (cm^{-1}) CCl$_4$	$\Delta\nu$ (cm^{-1}) DCM	$\Delta\nu$ (cm^{-1}) DMSO	ε CCl$_4$, DCM, DMSO	η CCl$_4$, DCM, DMSO	Δf CCl$_4$, DCM, DMSO
**4**	12,020	12,804	12,658	2.24, 8.93, 46.7	1.4601, 1.4241, 1.4783	-0.1351, 0.0810, 0.1120
**5**	11,230	11,713	11,618			
**7**	12,927	13,590	13,466			

b. Data for Literature Reported Small Molecule, Linear Analogues

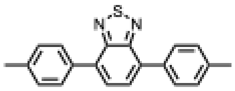

S13

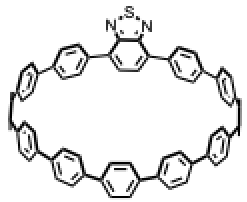

BT[12]CPP (1)

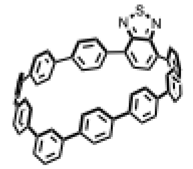

BT-6-m[10]CPP (2)

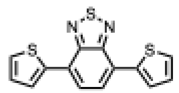

S14

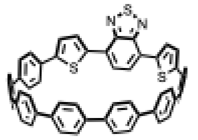

BT-2,10-thio[10]CPP (7)

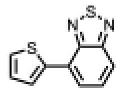

S15

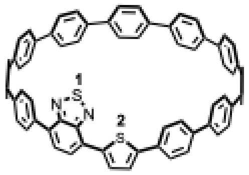

BT-2-thio[12]CPP (4)

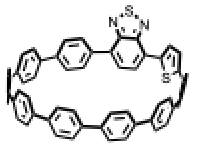

BT-2-thio[10]CPP (6)

Table S11: Literature absorbance and emission values for the small molecule, linear analogues (**S13**, **S14**, and **S15**)[7,11,12] of D-A nanohoops **1**, **2**, **7** (in DCM), and **4** and **6** (in DMSO).

Compound	Solvent	λ_{abs} (HOMO→LUMO, nm)	λ_{em} (nm)
**S13**	DCM	390	505
**1**		440	558
**2**		454	583
**S14**		445	577
**7**		495	639
**S15**	DMSO	-	536
**4**		470	622
**6**		473	631

c. Computational Photophysical Characterization

Predicted Absorbance and Emission Values

All calculations were performed using Gaussian 09. All calculations were conducted using the CAM-B3LYP function, the 6-31G** basis set, and the effect of the solvent was included by the polarizable continuum model (PCM) using either dichloromethane or dimethylsulfoxide as the solvent.

Geometry optimizations: Initial, ground state geometries (S0 orbitals) were obtained using the keyword “Opt Freq”.

Predicted absorbance data: The ground state geometry optimizations were used to perform a time dependent calculation of 12 state to give predicted UV-vis spectra of **1-7**, **BT[10]CPP**, and **Thio[10]CPP**. Calculations were run using the keyword “TD=NSTATE=12”. The results were analyzed using GaussSum.

Excited state geometries: The ground state geometry optimizations were used to perform a time dependent optimization to yield the excited state geometries (S1’ orbitals). Calculations were run using the keyword “Opt TD”.

Predicted fluorescence data: The excited state geometry optimizations were used to perform a time dependent calculation of 12 state to give predicted fluorescence spectra of **1-7**, **BT[10]CPP**, and **Thio[10]CPP**. Calculations were run using the keyword “TD=NSTATE=12”.

Note: Molecular orbital transitions ascribed to major (light blue) and minor (light green) absorbance and emission maxima values are highlighted below.

Link to computational data on FigShare: https://doi.org/10.6084/m9.figshare.32719806

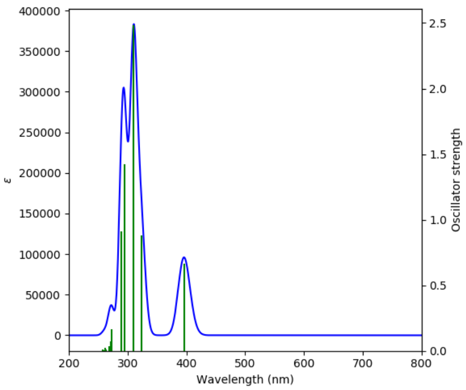

No.	Energy (cm^{-1})	λ (nm)	Osc. Strength	Major Contributions
1	25250.7995	396.0270	0.6632	H-1->LUMO (37%), HOMO->LUMO (45%)
2	30934.5885	323.2627	0.8819	H-2->L+2 (11%), H-1->L+1 (18%), HOMO->L+1 (43%)
3	32233.9477	310.2319	2.4864	H-2->LUMO (26%), H-2->L+1 (12%), HOMO->L+2 (23%)
4	33909.1613	294.9055	1.4223	H-1->L+1 (19%), HOMO->L+3 (29%)
5	34547.9524	289.4527	0.9123	H-2->LUMO (16%), H-2->L+1 (20%), HOMO->L+2 (11%)
6	36584.5023	273.3397	0.1649	H-18->LUMO (49%)
7	36921.6420	270.8438	0.0693	H-1->LUMO (17%), HOMO->LUMO (41%)
8	37300.7226	268.0913	0.0360	H-18->LUMO (18%), H-3->L+1 (11%), HOMO->L+4 (11%)
9	37916.1237	263.7400	0.0084	H-4->L+1 (16%), H-1->L+3 (11%), HOMO->L+5 (16%)
10	38187.1259	261.8683	0.0225	H-8->LUMO (10%)
11	38656.5406	258.6884	0.0027	H-7->L+1 (15%), H-1->L+6 (25%), HOMO->L+6 (20%)
12	38846.0809	257.4262	0.0104	H-24->LUMO (16%)

Figure S49. Calculated absorbance spectrum and electronic transitions for **1** in DCM.

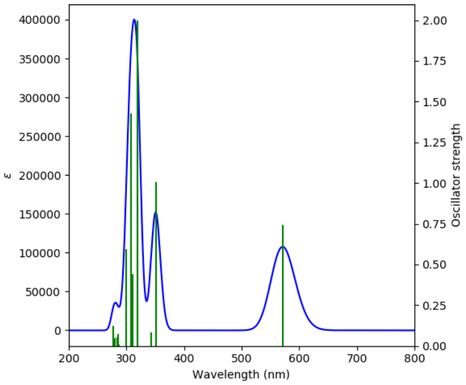

No.	Energy (cm^{-1})	λ (nm)	Osc. Strength	Major Contributions
1	17503.8442	571.3030	0.7437	HOMO->LUMO (92%)
2	28474.5975	351.1902	1.0074	H-4->LUMO (20%), H-2->LUMO (56%)
3	29209.3686	342.3559	0.0818	H-2->L+2 (10%), HOMO->L+1 (22%), HOMO->L+3 (33%)
4	31383.0328	318.6435	1.9996	H-1->L+1 (45%), HOMO->L+3 (20%)
5	32243.6264	310.1388	0.4377	H-3->LUMO (14%), H-1->LUMO (45%)
6	32505.7565	307.6378	1.4297	H-2->L+1 (11%), HOMO->L+2 (31%)
7	33417.1631	299.2474	0.5931	H-18->LUMO (30%)
8	34790.7252	287.4329	0.0079	H-5->LUMO (10%), H-3->LUMO (23%), H-1->LUMO (20%)
9	35108.5077	284.8312	0.0734	H-17->LUMO (19%)
10	35262.5596	283.5869	0.0525	H-3->LUMO (11%)
11	35674.7089	280.3106	0.0495	H-18->LUMO (32%), H-4->LUMO (11%)
12	36072.3402	277.2207	0.1214	H-4->LUMO (17%), H-2->LUMO (20%)

Figure S50. Calculated emission spectrum and electronic transitions for **1** in DCM.

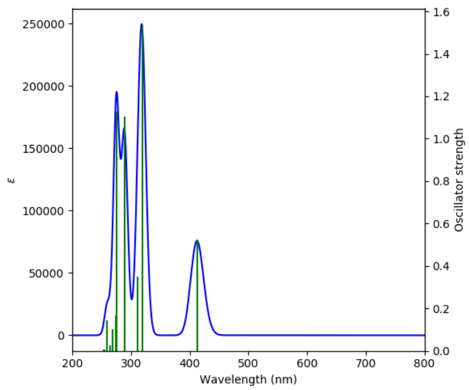

No.	Energy (cm^{-1})	λ (nm)	Osc. Strength	Major Contributions
1	24240.1868	412.5380	0.5233	H-2->LUMO (12%), HOMO->LUMO (79%)
2	31338.6723	319.0945	1.5367	H-1->LUMO (37%), H-1->L+1 (14%), HOMO->L+1 (27%)
3	32093.6072	311.5885	0.3486	H-1->LUMO (13%), H-1->L+2 (17%), HOMO->L+1 (15%), HOMO->L+2 (26%)
4	34596.3456	289.0478	1.1039	H-1->LUMO (21%), H-1->L+1 (11%), HOMO->L+1 (12%), HOMO->L+2 (17%)
5	36269.9461	275.7103	1.1292	H-2->L+2 (16%), HOMO->L+3 (16%)
6	36543.3680	273.6474	0.1648	H-15->LUMO (47%)
7	37244.2638	268.4977	0.1037	H-4->LUMO (19%), H-2->LUMO (32%), HOMO->LUMO (14%)
8	37729.8096	265.0424	0.0083	H-6->LUMO (10%), H-6->L+1 (12%), H-1->L+5 (12%), HOMO->L+5 (24%)
9	37845.1469	264.2346	0.0264	H-15->LUMO (15%), H-7->LUMO (14%), HOMO->L+6 (13%)
10	38564.5934	259.3052	0.1427	H-2->L+1 (13%), H-2->L+2 (14%), HOMO->L+4 (11%)
11	39128.3750	255.5690	0.0068	H-20->LUMO (21%), H-13->LUMO (10%)
12	39507.4555	253.1167	0.0075	H-20->LUMO (31%)

Figure S51. Calculated absorbance spectrum and electronic transitions for **2** in DCM.

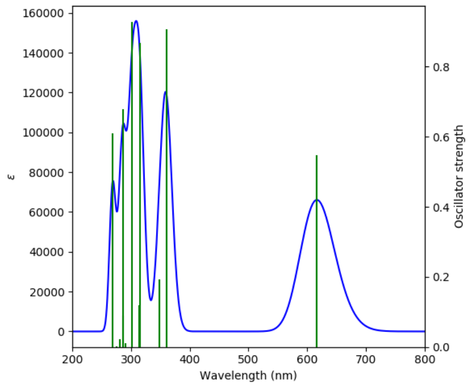

No.	Energy (cm^{-1})	λ (nm)	Osc. Strength	Major Contributions
1	16217.3899	616.6220	0.5474	HOMO->LUMO (95%)
2	27760.7968	360.2202	0.9065	H-3->LUMO (19%), H-1->LUMO (69%)
3	28751.2457	347.8110	0.1936	HOMO->L+1 (61%), HOMO->L+3 (12%)
4	31765.3396	314.8085	0.8683	H-15->LUMO (18%), HOMO->L+2 (33%), HOMO->L+4 (11%)
5	31816.9591	314.2977	0.1184	H-4->LUMO (15%), H-2->LUMO (57%)
6	33135.6756	301.7895	0.9286	H-15->LUMO (16%), HOMO->L+2 (23%)
7	34401.9660	290.6810	0.0118	H-13->LUMO (27%), H-11->LUMO (25%), H-9->LUMO (12%)
8	34905.2560	286.4898	0.0902	H-15->LUMO (38%)
9	34983.4918	285.8491	0.6800	H-2->L+1 (18%), H-1->L+2 (30%), HOMO->L+3 (17%)
10	35609.3780	280.8248	0.0222	H-3->LUMO (39%), H-1->LUMO (18%)
11	36297.3689	275.5020	0.0023	H-20->LUMO (24%), H-19->LUMO (50%)
12	37186.9985	268.9111	0.6098	H-2->L+2 (28%), H-1->L+1 (11%), HOMO->L+4 (14%)

Figure S52. Calculated emission spectrum and electronic transitions for **2** in DCM.

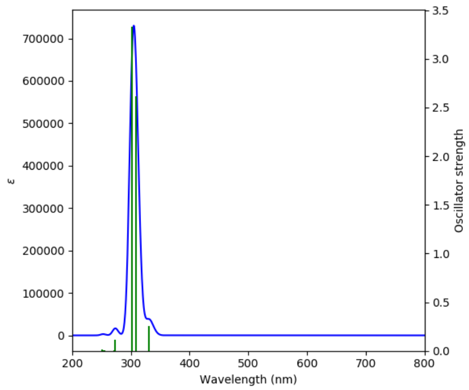

No.	Energy (cm⁻¹)	λ (nm)	Osc. Strength	Major Contributions
1	30192.5584	331.2074	0.2568	H-2->L+2 (14%), H-1->L+1 (19%), HOMO->LUMO (51%)
2	32347.6719	309.1412	2.6151	H-1->LUMO (35%), HOMO->L+1 (32%)
3	33089.7020	302.2088	3.3334	H-2->LUMO (36%), HOMO->L+2 (35%)
4	36554.6598	273.5629	0.1112	H-3->LUMO (22%), H-2->L+2 (23%), H-1->L+1 (19%), HOMO->L+4 (19%)
5	36763.5574	272.0084	0.0035	H-4->LUMO (18%), H-2->L+1 (18%), H-1->L+2 (23%), HOMO->L+3 (23%)
6	39180.8010	255.2270	0.0056	HOMO->L+5 (25%)
7	39430.8329	253.6086	0.0039	H-1->L+7 (15%), HOMO->L+7 (13%)
8	39470.3540	253.3547	0.0008	H-2->L+5 (12%), HOMO->L+6 (23%)
9	39757.4874	251.5249	0.0111	HOMO->L+8 (15%)
10	39905.0869	250.5946	0.0001	HOMO->L+9 (13%)
11	39943.8015	250.3517	0.0005	HOMO->L+10 (15%)
12	40046.2339	249.7113	0.0002	HOMO->L+11 (16%)

Figure S53. Calculated absorbance spectrum and electronic transitions for **3** in DCM.

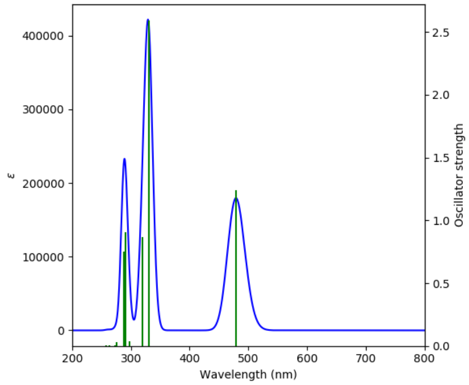

No.	Energy (cm⁻¹)	λ (nm)	Osc. Strength	Major Contributions
1	20875.2417	479.0363	1.2404	HOMO->LUMO (91%)
2	30262.7287	330.4394	2.5940	H-2->LUMO (30%), HOMO->L+2 (34%)
3	31222.5285	320.2815	0.8665	H-2->L+2 (18%), H-1->L+1 (43%)
4	33581.7002	297.7812	0.0384	H-2->LUMO (33%), HOMO->L+2 (29%), HOMO->L+4 (12%)
5	34430.1954	290.4427	0.9066	H-3->LUMO (13%), H-1->L+1 (24%), HOMO->L+3 (14%)
6	34786.6925	287.4662	0.7521	H-4->LUMO (13%), H-2->L+1 (22%), H-1->L+2 (17%)
7	36211.0676	276.1586	0.0275	H-11->LUMO (11%), HOMO->L+5 (15%), HOMO->L+11 (11%)
8	36561.1122	273.5146	0.0040	H-17->LUMO (20%), HOMO->L+6 (18%), HOMO->L+10 (15%)
9	37084.5660	269.6539	0.0006	H-3->LUMO (11%), H-1->LUMO (36%), HOMO->L+1 (30%), HOMO->L+3 (12%)
10	38046.7855	262.8342	0.0066	H-18->LUMO (19%)
11	38687.9962	258.4781	0.0011	-
12	38782.3631	257.8491	0.0016	-

Figure S54. Calculated emission spectrum and electronic transitions for **3** in DCM.

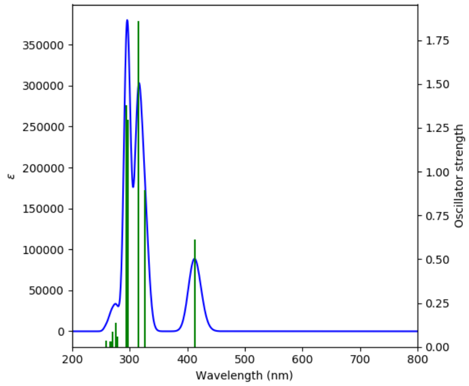

No.	Energy (cm^{-1})	λ (nm)	Osc. Strength	Major Contributions
1	24233.7343	412.6479	0.6117	H-1->LUMO (45%), HOMO->LUMO (41%)
2	30643.4224	326.3343	0.8932	H-2->L+2 (12%), H-1->L+1 (15%), HOMO->L+1 (46%)
3	31770.1789	314.7605	1.8601	H-4->LUMO (13%), H-2->LUMO (32%), HOMO->L+2 (16%)
4	33700.2637	296.7335	1.2933	H-1->L+1 (15%), HOMO->L+2 (14%), HOMO->L+3 (13%)
5	33967.2332	294.4013	1.3771	H-4->LUMO (10%), H-2->L+1 (19%), HOMO->L+3 (17%)
6	35839.2460	279.0237	0.0578	H-1->LUMO (13%), HOMO->LUMO (37%)
7	36199.7758	276.2448	0.1390	H-18->LUMO (54%)
8	37108.7627	269.4781	0.0859	H-3->L+1 (12%), H-2->L+3 (11%), H-1->L+2 (13%), HOMO->L+4 (14%)
9	37502.3612	266.6498	0.0326	H-24->LUMO (19%), H-13->LUMO (15%), H-10->LUMO (18%)
10	37616.8920	265.8380	0.0324	H-2->L+2 (12%), H-1->L+3 (15%), HOMO->L+5 (17%)
11	38559.7541	259.3377	0.0376	H-11->LUMO (20%), H-10->LUMO (24%)
12	38687.9962	258.4781	0.0037	H-7->L+1 (14%), H-1->L+6 (12%), HOMO->L+6 (17%)

Figure S55. Calculated absorbance spectrum and electronic transitions for **4** in DCM.

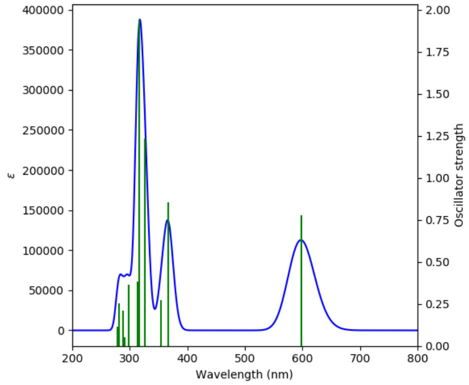

No.	Energy (cm^{-1})	λ (nm)	Osc. Strength	Major Contributions
1	16734.3912	597.5717	0.7783	HOMO->LUMO (94%)
2	27227.6644	367.2735	0.8527	H-4->LUMO (15%), H-2->LUMO (32%), HOMO->L+1 (17%), HOMO->L+2 (11%)
3	28198.7559	354.6255	0.2744	H-2->LUMO (27%), HOMO->L+1 (20%), HOMO->L+3 (17%)
4	30626.4847	326.5147	1.2364	H-1->LUMO (15%), H-1->L+1 (33%), HOMO->L+2 (11%)
5	31643.5499	316.0201	1.9391	H-2->L+1 (11%), HOMO->L+2 (18%), HOMO->L+3 (21%)
6	31947.6209	313.0123	0.3848	H-3->LUMO (14%), H-1->LUMO (46%)
7	33578.4739	297.8098	0.3664	H-18->LUMO (25%)
8	34332.6023	291.2683	0.0498	H-3->LUMO (27%), H-1->LUMO (28%)
9	34672.1617	288.4158	0.2119	-
10	35523.8832	281.5007	0.2528	H-4->LUMO (10%), H-2->LUMO (12%)
11	35652.9319	280.4818	0.0136	H-16->LUMO (19%), H-12->LUMO (12%), H-9->LUMO (11%)
12	35857.7968	278.8793	0.1119	H-4->LUMO (13%), H-3->LUMO (12%), H-2->LUMO (14%)

Figure S56. Calculated emission spectrum and electronic transitions for **4** in DCM.

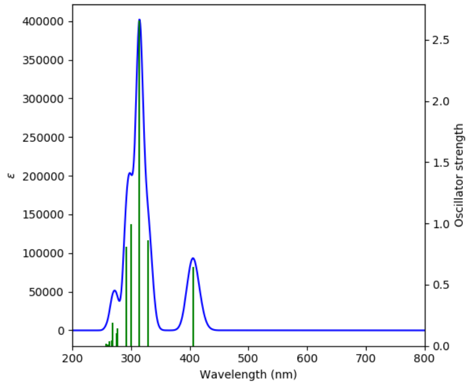

No.	Energy (cm^{-1})	λ (nm)	Osc. Strength	Major Contributions
1	24649.1099	405.6941	0.6447	H-3->LUMO (10%), H-1->LUMO (42%), HOMO->LUMO (41%)
2	30338.5448	329.6136	0.8609	H-2->L+2 (13%), H-1->L+1 (16%), H-1->L+3 (11%), HOMO->L+1 (44%)
3	31791.9559	314.5449	2.6596	H-2->LUMO (31%), H-2->L+1 (15%), HOMO->L+2 (22%)
4	33338.9273	299.9496	0.9951	H-2->L+2 (13%), H-1->L+1 (21%), HOMO->L+3 (29%)
5	34300.3402	291.5422	0.8081	H-4->LUMO (13%), H-2->LUMO (26%), H-2->L+1 (16%), HOMO->L+2 (15%)
6	36143.3170	276.6763	0.1422	H-18->LUMO (43%)
7	36323.9852	275.3001	0.1022	H-18->LUMO (10%), H-1->LUMO (15%), HOMO->LUMO (40%)
8	37192.6443	268.8703	0.1888	H-3->L+1 (15%), H-2->L+2 (14%), H-1->L+3 (19%), HOMO->L+5 (19%)
9	37331.3717	267.8712	0.0448	H-4->L+1 (12%), H-2->L+3 (10%), H-1->L+2 (22%), HOMO->L+4 (17%)
10	38025.8151	262.9792	0.0353	H-18->LUMO (18%), H-9->LUMO (27%)
11	38371.0204	260.6133	0.0111	H-13->LUMO (17%)
12	38787.2024	257.8169	0.0164	H-3->LUMO (20%), H-1->LUMO (21%)

Figure S57. Calculated absorbance spectrum and electronic transitions for **5** in DCM.

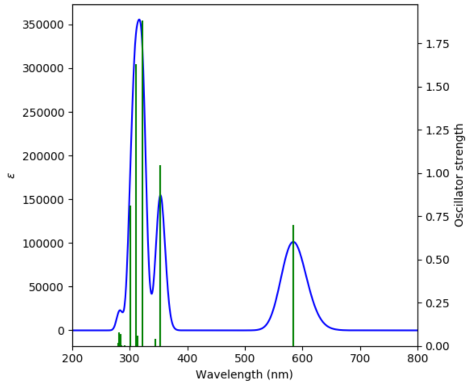

No.	Energy (cm^{-1})	λ (nm)	Osc. Strength	Major Contributions
1	17113.4718	584.3349	0.6994	HOMO->LUMO (92%)
2	28288.2834	353.5032	1.0473	H-4->LUMO (22%), H-2->LUMO (60%)
3	29078.7068	343.8942	0.0430	H-2->L+2 (10%), HOMO->L+1 (23%), HOMO->L+3 (29%)
4	31071.7028	321.8362	1.8838	H-1->L+1 (44%), HOMO->L+3 (21%)
5	31921.8111	313.2654	0.0577	H-3->LUMO (17%), H-1->LUMO (53%)
6	32212.1707	310.4416	1.6314	H-18->LUMO (10%), H-2->L+1 (12%), HOMO->L+2 (34%)
7	33165.5181	301.5179	0.8115	H-18->LUMO (28%), H-10->LUMO (10%), H-2->L+1 (10%)
8	34367.2842	290.9744	0.0046	H-5->LUMO (11%), H-3->LUMO (24%), H-1->LUMO (26%)
9	34943.1640	286.1790	0.0057	H-14->LUMO (10%), H-8->LUMO (10%)
10	35145.6092	284.5305	0.0672	H-13->LUMO (11%), H-10->LUMO (11%)
11	35541.6274	281.3602	0.0775	H-18->LUMO (33%)
12	35746.4922	279.7477	0.0156	H-4->LUMO (13%), H-3->L+1 (12%), H-2->LUMO (11%), H-2->L+2 (15%)

Figure S58. Calculated emission spectrum and electronic transitions for **5** in DCM.

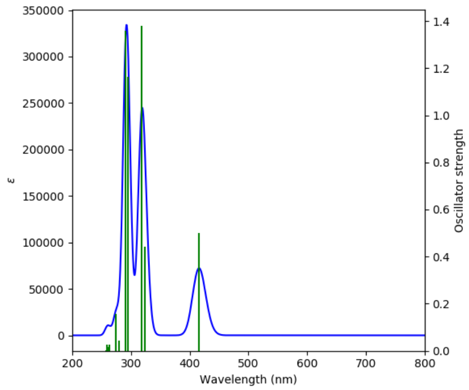

No.	Energy (cm⁻¹)	λ (nm)	Osc. Strength	Major Contributions
1	24040.9678	415.9566	0.4994	H-1->LUMO (25%), HOMO->LUMO (62%)
2	30858.7724	324.0569	0.4416	H-2->L+2 (11%), HOMO->L+1 (47%)
3	31451.5899	317.9489	1.3805	H-2->LUMO (42%), HOMO->L+2 (15%)
4	33888.1908	295.0880	1.1629	H-2->LUMO (10%), H-1->L+1 (27%), HOMO->L+2 (19%), HOMO->L+3 (15%)
5	34433.4216	290.4155	1.3601	H-2->L+1 (25%), HOMO->L+2 (11%), HOMO->L+3 (22%)
6	35686.0007	280.2219	0.0433	H-1->LUMO (37%), HOMO->LUMO (32%)
7	36384.4768	274.8424	0.1576	H-16->LUMO (20%), H-15->LUMO (37%), H-14->LUMO (12%)
8	37912.0909	263.7680	0.0257	H-3->L+1 (10%), H-1->L+2 (18%), HOMO->L+4 (15%)
9	38219.3881	261.6473	0.0142	H-7->LUMO (15%), HOMO->L+5 (11%)
10	38498.4560	259.7506	0.0151	-
11	38649.2816	258.7370	0.0261	H-7->LUMO (23%)
12	38900.9266	257.0632	0.0018	HOMO->L+6 (22%)

Figure S59. Calculated absorbance spectrum and electronic transitions for **6** in DCM.

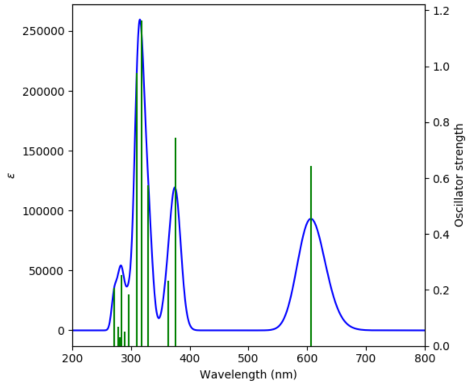

No.	Energy (cm⁻¹)	λ (nm)	Osc. Strength	Major Contributions
1	16489.1987	606.4576	0.6440	HOMO->LUMO (93%)
2	26581.6143	376.1998	0.7441	H-2->LUMO (35%), HOMO->L+1 (32%)
3	27561.5779	362.8239	0.2318	H-2->LUMO (36%), HOMO->L+1 (27%)
4	30322.4137	329.7890	0.5751	H-1->LUMO (36%), H-1->L+1 (13%), HOMO->L+2 (18%)
5	31439.4916	318.0713	1.1644	H-2->L+1 (10%), H-1->LUMO (15%), HOMO->L+2 (29%), HOMO->L+3 (22%)
6	32260.5640	309.9759	0.9767	H-1->LUMO (17%), H-1->L+1 (22%), HOMO->L+3 (10%)
7	33698.6505	296.7477	0.1839	H-16->LUMO (24%), H-14->LUMO (12%), H-8->LUMO (12%)
8	34651.9979	288.5836	0.0511	H-8->LUMO (12%), H-3->LUMO (19%), H-1->LUMO (13%)
9	35257.7203	283.6258	0.2548	H-4->LUMO (19%), H-1->L+2 (14%)
10	35647.2860	280.5262	0.0298	H-16->LUMO (14%), H-12->LUMO (16%), H-6->LUMO (11%)
11	35914.2556	278.4409	0.0684	H-16->LUMO (12%), H-3->LUMO (19%)
12	36812.7572	271.6449	0.2064	H-4->LUMO (14%), H-2->LUMO (11%)

Figure S60. Calculated emission spectrum and electronic transitions for **6** in DCM.

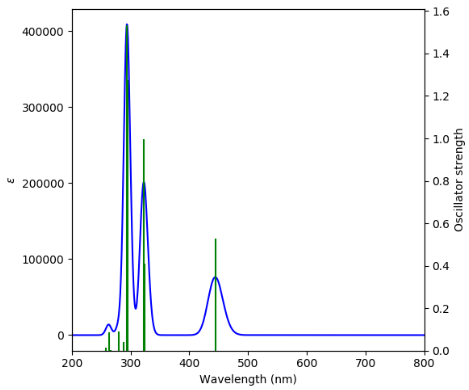

No.	Energy (cm^{-1})	λ (nm)	Osc. Strength	Major Contributions
1	22510.1275	444.2444	0.5274	H-1->LUMO (22%), HOMO->LUMO (71%)
2	30868.4511	323.9553	0.4095	H-2->L+2 (16%), H-1->L+1 (19%), HOMO->L+1 (39%)
3	31062.0241	321.9365	0.9957	H-4->LUMO (23%), H-2->LUMO (51%)
4	33980.9446	294.2825	1.2760	H-1->L+1 (30%), HOMO->L+3 (40%)
5	34074.5049	293.4745	1.5343	H-4->LUMO (10%), H-2->L+1 (29%), HOMO->L+2 (35%)
6	34773.7876	287.5729	0.0395	H-1->LUMO (50%), HOMO->LUMO (23%)
7	35675.5155	280.3042	0.0919	H-15->LUMO (53%), H-7->LUMO (11%)
8	37258.7818	268.3931	0.0005	H-10->LUMO (14%), H-6->LUMO (36%)
9	37649.1541	265.6102	0.0013	H-7->LUMO (16%), H-1->L+2 (17%), HOMO->L+4 (14%)
10	38004.8446	263.1243	0.0021	H-3->L+1 (14%), H-2->L+2 (17%), H-1->L+3 (16%), HOMO->L+5 (21%)
11	38035.4937	262.9123	0.0855	H-15->LUMO (14%), H-7->LUMO (31%)
12	38786.3959	257.8223	0.0146	H-4->LUMO (29%), H-2->LUMO (21%)

Figure S61. Calculated absorbance spectrum and electronic transitions for **7** in DCM.

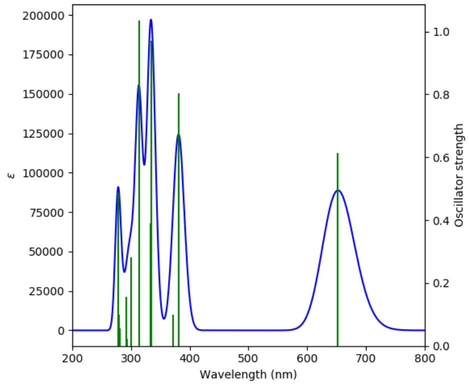

No.	Energy (cm^{-1})	λ (nm)	Osc. Strength	Major Contributions
1	15322.9210	652.6170	0.6134	HOMO->LUMO (95%)
2	26195.2747	381.7482	0.8037	H-4->LUMO (17%), H-2->LUMO (72%)
3	26909.8819	371.6106	0.0991	HOMO->L+1 (47%), HOMO->L+3 (27%)
4	29871.5498	334.7666	0.9711	HOMO->L+2 (67%)
5	30051.4114	332.7630	0.3909	H-3->LUMO (10%), H-1->LUMO (58%)
6	31887.9359	313.5982	1.0359	H-1->LUMO (11%), H-1->L+1 (35%), H-1->L+3 (13%), HOMO->L+3 (11%)
7	33284.0816	300.4439	0.2812	H-16->LUMO (30%), H-9->LUMO (13%)
8	34112.4130	293.1484	0.0229	H-11->LUMO (32%), H-3->LUMO (28%), H-1->LUMO (15%)
9	34176.9373	292.5949	0.1561	H-9->LUMO (31%), H-4->LUMO (23%)
10	35497.2669	281.7118	0.0555	H-15->LUMO (13%), H-3->LUMO (19%), HOMO->L+1 (10%), HOMO->L+5 (13%)
11	35777.9479	279.5017	0.0992	H-16->LUMO (33%), H-9->LUMO (13%), H-4->LUMO (14%)
12	36016.6880	277.6490	0.4878	H-4->LUMO (18%), H-2->LUMO (11%), H-2->L+1 (11%), H-1->L+2 (11%), HOMO->L+4 (14%)

Figure S62. Calculated emission spectrum and electronic transitions for **7** in DCM.

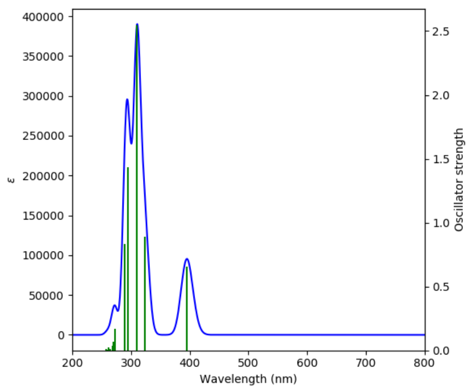

No.	Energy (cm^{-1})	λ (nm)	Osc. Strength	Major Contributions
1	25293.5468	395.3577	0.6587	H-1->LUMO (37%), HOMO->LUMO (44%)
2	30861.9986	324.0230	0.8897	H-2->L+2 (11%), H-1->L+1 (18%), HOMO->L+1 (43%)
3	32210.5576	310.4572	2.5493	H-2->LUMO (25%), H-2->L+1 (12%), HOMO->L+2 (24%)
4	33883.3515	295.1301	1.4363	H-1->L+1 (19%), HOMO->L+3 (30%)
5	34546.3393	289.4662	0.8311	H-2->LUMO (17%), H-2->L+1 (19%), HOMO->L+2 (11%)
6	36568.3712	273.4603	0.1660	H-18->LUMO (49%)
7	36942.6125	270.6901	0.0701	H-1->LUMO (17%), HOMO->LUMO (40%)
8	37287.8178	268.1841	0.0357	H-18->LUMO (18%), H-3->L+1 (11%), HOMO->L+4 (11%)
9	37890.3139	263.9196	0.0091	H-4->L+1 (16%), H-1->L+3 (10%), HOMO->L+5 (16%)
10	38210.5160	261.7080	0.0214	H-16->LUMO (10%)
11	38625.0850	258.8991	0.0025	H-7->L+1 (15%), H-1->L+6 (24%), HOMO->L+6 (20%)
12	38886.4086	257.1592	0.0097	H-24->LUMO (13%), H-15->LUMO (14%)

Figure S63. Calculated absorbance spectrum and electronic transitions for **1** in DMSO.

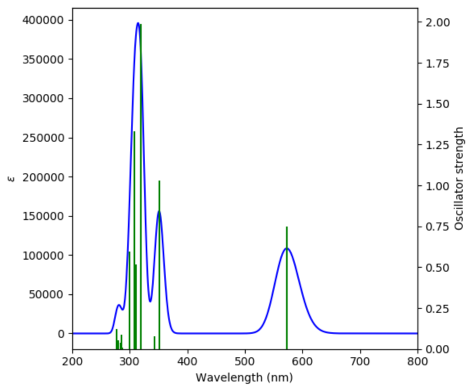

No.	Energy (cm^{-1})	Wavelength (nm)	Osc. Strength	Major Contributions
1	17453.0312	572.9663	0.7495	HOMO->LUMO (92%)
2	28461.6926	351.3494	1.0305	H-4->LUMO (20%), H-2->LUMO (56%)
3	29136.7787	343.2088	0.0800	HOMO->L+1 (22%), HOMO->L+3 (34%)
4	31312.8625	319.3575	1.9893	H-1->L+1 (45%), HOMO->L+3 (20%)
5	32204.9117	310.5116	0.5156	H-3->LUMO (13%), H-1->LUMO (43%)
6	32443.6519	308.2267	1.3290	H-2->L+1 (10%), H-1->LUMO (11%), HOMO->L+2 (29%)
7	33376.0288	299.6162	0.5980	H-18->LUMO (30%), H-15->LUMO (10%)
8	34775.4007	287.5595	0.0072	H-5->LUMO (10%), H-3->LUMO (24%), H-1->LUMO (21%)
9	35083.5045	285.0342	0.0892	H-17->LUMO (18%)
10	35264.9792	283.5674	0.0396	H-3->LUMO (13%)
11	35644.0598	280.5516	0.0531	H-18->LUMO (34%)
12	36056.2091	277.3447	0.1222	H-4->LUMO (18%), H-2->LUMO (21%)

Figure S64. Calculated emission spectrum and electronic transitions for **1** in DMSO.

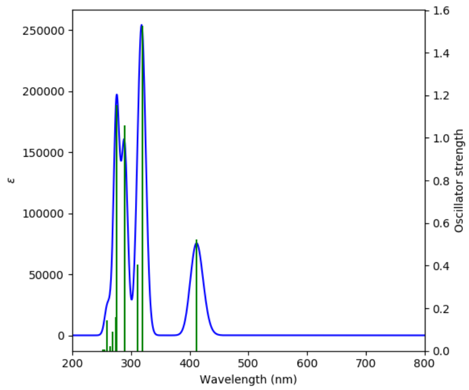

No.	Energy (cm⁻¹)	λ (nm)	Osc. Strength	Major Contributions
1	24283.7407	411.7981	0.5215	H-2->LUMO (12%), HOMO->LUMO (79%)
2	31330.6067	319.1767	1.5270	H-1->LUMO (35%), H-1->L+1 (15%), HOMO->L+1 (29%)
3	32068.6040	311.8314	0.4061	H-1->LUMO (14%), H-1->L+2 (17%), HOMO->L+1 (12%), HOMO->L+2 (28%)
4	34594.7325	289.0613	1.0602	H-1->LUMO (22%), H-1->L+1 (10%), HOMO->L+1 (12%), HOMO->L+2 (16%)
5	36242.5232	275.9189	1.1553	H-2->L+2 (16%), HOMO->L+3 (16%)
6	36536.9156	273.6957	0.1574	H-15->LUMO (43%)
7	37265.2342	268.3466	0.0919	H-4->LUMO (20%), H-2->LUMO (32%), HOMO->LUMO (14%)
8	37720.9375	265.1047	0.0081	H-6->LUMO (10%), H-6->L+1 (13%), H-1->L+5 (12%), HOMO->L+5 (25%)
9	37855.6321	264.1614	0.0232	H-15->LUMO (13%), H-7->LUMO (13%), HOMO->L+6 (13%)
10	38529.9116	259.5386	0.1420	H-2->L+1 (13%), H-2->L+2 (14%), HOMO->L+4 (11%)
11	39163.8634	255.3374	0.0070	H-20->LUMO (15%), H-13->LUMO (12%)
12	39551.8160	252.8328	0.0070	H-20->LUMO (31%)

Figure S65. Calculated absorbance spectrum and electronic transitions for **2** in DMSO.

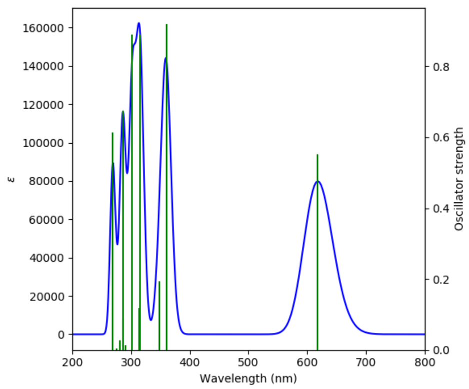

No.	Energy (cm⁻¹)	λ (nm)	Osc. Strength	Major Contributions
1	16175.4490	618.2208	0.5511	HOMO->LUMO (95%)
2	27758.3772	360.2516	0.9204	H-3->LUMO (19%), H-1->LUMO (69%)
3	28673.8165	348.7502	0.1927	HOMO->L+1 (61%), HOMO->L+3 (13%)
4	31698.3956	315.4733	0.8891	H-15->LUMO (17%), HOMO->L+2 (34%), HOMO->L+4 (11%)
5	31784.6969	314.6168	0.1185	H-4->LUMO (15%), H-2->LUMO (57%)
6	33084.0561	302.2603	0.8901	H-15->LUMO (16%), H-12->LUMO (10%), HOMO->L+2 (22%)
7	34420.5168	290.5244	0.0124	H-13->LUMO (29%), H-11->LUMO (24%), H-9->LUMO (11%)
8	34896.3839	286.5626	0.0873	H-15->LUMO (39%), H-8->LUMO (10%)
9	34949.6165	286.1261	0.6745	H-2->L+1 (18%), H-1->L+2 (30%), HOMO->L+3 (17%)
10	35592.4403	280.9585	0.0268	H-3->LUMO (39%), H-1->LUMO (18%)
11	36354.6343	275.0680	0.0029	H-20->LUMO (13%), H-19->LUMO (61%)
12	37157.9625	269.1213	0.6128	H-2->L+2 (28%), H-1->L+1 (11%), HOMO->L+4 (14%)

Figure S66. Calculated emission spectrum and electronic transitions for **2** in DMSO.

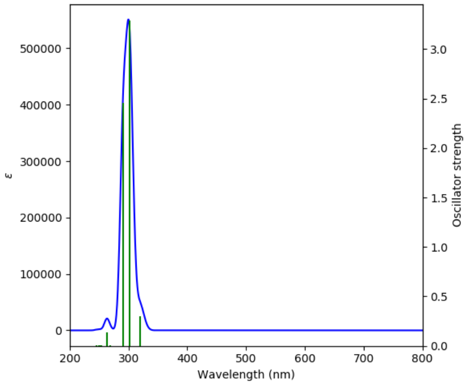

No.	Energy (cm^{-1})	λ (nm)	Osc. Strength	Major Contributions
1	31253.9841	319.9592	0.3019	H-2->L+2 (10%), H-1->L+1 (22%), HOMO->LUMO (53%)
2	33123.5772	301.8997	3.2917	H-1->LUMO (32%), HOMO->L+1 (30%)
3	34351.1531	291.1110	2.4546	H-2->LUMO (21%), H-1->L+1 (23%), HOMO->L+2 (20%)
4	37283.7850	268.2131	0.0077	H-3->LUMO (18%), H-2->L+1 (17%), H-1->L+2 (21%), HOMO->L+3 (16%)
5	37979.0349	263.3031	0.1401	H-3->L+1 (21%), H-1->L+3 (18%)
6	39342.1119	254.1805	0.0002	H-7->LUMO (11%), HOMO->L+6 (16%)
7	39509.8752	253.1012	0.0039	HOMO->L+7 (14%)
8	39747.8088	251.5861	0.0020	HOMO->L+8 (13%)
9	39928.4770	250.4478	0.0014	HOMO->L+11 (10%)
10	40126.8893	249.2094	0.0034	HOMO->L+12 (12%)
11	40424.5079	247.3746	0.0007	-
12	40685.8316	245.7858	0.0058	-

Figure S67. Calculated absorbance spectrum and electronic transitions for **3** in DMSO.

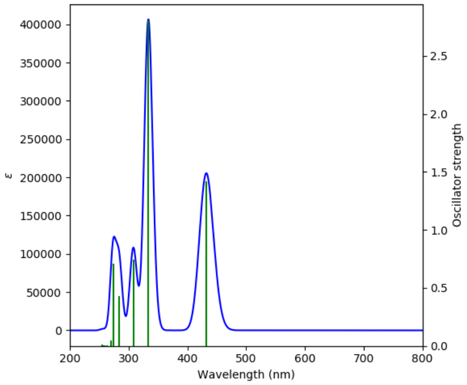

No.	Energy (cm^{-1})	λ (nm)	Osc. Strength	Major Contributions
1	23131.9810	432.3019	1.4192	HOMO->LUMO (88%)
2	29971.5625	333.6496	2.8083	H-1->LUMO (32%), HOMO->L+1 (30%)
3	32454.1371	308.1271	0.7461	H-2->LUMO (14%), H-2->L+2 (14%), H-1->L+1 (36%), HOMO->L+2 (12%)
4	35020.5933	285.5462	0.0010	H-1->LUMO (16%), HOMO->L+1 (17%), HOMO->L+6 (22%)
5	35167.3862	284.3543	0.1920	H-1->LUMO (13%), H-1->L+2 (11%), HOMO->L+1 (12%), HOMO->L+6 (16%)
6	35253.6875	283.6582	0.4298	H-2->L+1 (22%), H-1->L+2 (16%), HOMO->L+3 (12%)
7	36517.5583	273.8408	0.7067	H-3->L+1 (20%), H-1->L+3 (19%)
8	36958.7436	270.5719	0.0477	HOMO->L+7 (13%)
9	38044.3658	262.8510	0.0048	H-19->LUMO (12%)
10	38619.4391	258.9369	0.0022	H-2->LUMO (27%), HOMO->L+2 (28%)
11	38855.7596	257.3621	0.0025	H-17->LUMO (11%)
12	39176.7682	255.2533	0.0087	H-19->LUMO (27%), HOMO->L+18 (11%)

Figure S68. Calculated emission spectrum and electronic transitions for **3** in DMSO.

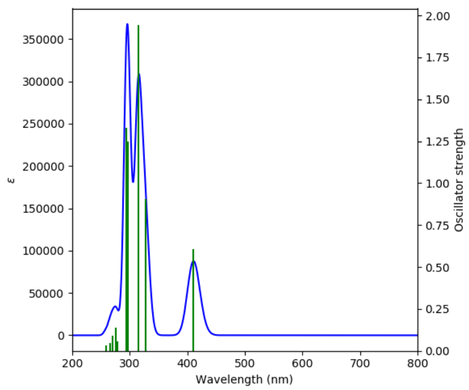

No.	Energy (cm⁻¹)	λ (nm)	Osc. Strength	Major Contributions
1	24339.3930	410.8565	0.6054	H-1->LUMO (46%), HOMO->LUMO (39%)
2	30563.5735	327.1868	0.9056	H-2->L+2 (12%), H-1->L+1 (15%), HOMO->L+1 (47%)
3	31770.9855	314.7525	1.9442	H-4->LUMO (12%), H-2->LUMO (31%), HOMO->L+2 (17%)
4	33688.1653	296.8401	1.2494	H-1->L+1 (15%), HOMO->L+2 (14%), HOMO->L+3 (13%)
5	33942.2300	294.6182	1.3295	H-4->LUMO (10%), H-2->L+1 (19%), HOMO->L+3 (17%)
6	35862.6361	278.8417	0.0568	H-1->LUMO (12%), HOMO->LUMO (36%)
7	36215.9069	276.1217	0.1387	H-18->LUMO (53%), HOMO->LUMO (10%)
8	37088.5988	269.6246	0.0879	H-3->L+1 (12%), H-2->L+3 (11%), H-1->L+2 (13%), HOMO->L+4 (14%)
9	37548.3348	266.3233	0.0472	H-24->LUMO (18%), H-13->LUMO (14%), H-10->LUMO (11%)
10	37621.7313	265.8038	0.0216	H-2->L+2 (10%), H-1->L+3 (13%), HOMO->L+5 (14%)
11	38632.3440	258.8504	0.0327	H-11->LUMO (23%), H-10->LUMO (11%)
12	38644.4423	258.7694	0.0055	H-7->L+1 (13%), H-1->L+6 (13%), HOMO->L+6 (19%)

Figure S69. Calculated absorbance spectrum and electronic transitions for **4** in DMSO.

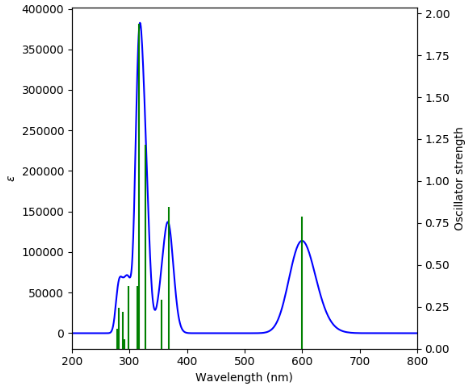

No.	Energy (cm⁻¹)	λ (nm)	Osc. Strength	Major Contributions
1	16669.0603	599.9138	0.7872	HOMO->LUMO (94%)
2	27160.7203	368.1787	0.8474	H-4->LUMO (14%), H-2->LUMO (30%), HOMO->L+1 (17%), HOMO->L+2 (12%)
3	28149.5561	355.2453	0.2908	H-4->LUMO (11%), H-2->LUMO (29%), HOMO->L+1 (19%), HOMO->L+3 (17%)
4	30540.1834	327.4374	1.2171	H-1->LUMO (15%), H-1->L+1 (34%), HOMO->L+2 (11%)
5	31567.7337	316.7791	1.9394	H-2->L+1 (11%), HOMO->L+2 (19%), HOMO->L+3 (21%)
6	31896.8080	313.5109	0.3747	H-3->LUMO (14%), H-1->LUMO (47%)
7	33530.0807	298.2396	0.3730	H-18->LUMO (24%)
8	34300.3402	291.5422	0.0544	H-3->LUMO (27%), H-1->LUMO (28%)
9	34616.5095	288.8795	0.2206	-
10	35493.2342	281.7438	0.2461	H-2->LUMO (12%)
11	35661.8040	280.4120	0.0136	H-16->LUMO (19%), H-12->LUMO (13%)
12	35821.5018	279.1619	0.1170	H-4->LUMO (16%), H-2->LUMO (14%)

Figure S70. Calculated emission spectrum and electronic transitions for **4** in DMSO.

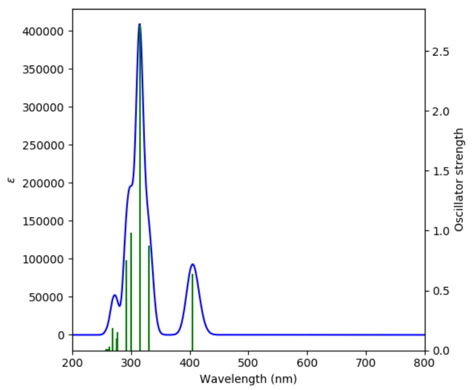

No.	Energy (cm⁻¹)	λ (nm)	Osc. Strength	Major Contributions
1	24679.7589	405.1903	0.6413	H-3->LUMO (10%), H-1->LUMO (42%), HOMO->LUMO (41%)
2	30254.6631	330.5275	0.8720	H-2->L+2 (13%), H-1->L+1 (16%), H-1->L+3 (10%), HOMO->L+1 (44%)
3	31766.1461	314.8005	2.7183	H-2->LUMO (30%), H-2->L+1 (15%), HOMO->L+2 (23%)
4	33305.8585	300.2474	0.9845	H-2->L+2 (13%), H-1->L+1 (21%), HOMO->L+3 (29%)
5	34301.1467	291.5354	0.7501	H-4->LUMO (14%), H-2->LUMO (27%), H-2->L+1 (16%), HOMO->L+2 (15%)
6	36132.0253	276.7627	0.1508	H-18->LUMO (45%)
7	36335.2770	275.2146	0.0966	H-3->LUMO (10%), H-1->LUMO (16%), HOMO->LUMO (42%)
8	37162.8018	269.0862	0.1847	H-3->L+1 (14%), H-2->L+2 (14%), H-1->L+3 (19%), HOMO->L+5 (19%)
9	37310.4013	268.0217	0.0502	H-4->L+1 (11%), H-2->L+3 (10%), H-1->L+2 (22%), HOMO->L+4 (17%)
10	38061.3035	262.7340	0.0339	H-18->LUMO (18%), H-9->LUMO (29%)
11	38400.0563	260.4162	0.0098	H-13->LUMO (17%)
12	38824.3039	257.5706	0.0149	H-3->LUMO (20%), H-1->LUMO (21%)

Figure S71. Calculated absorbance spectrum and electronic transitions for **5** in DMSO.

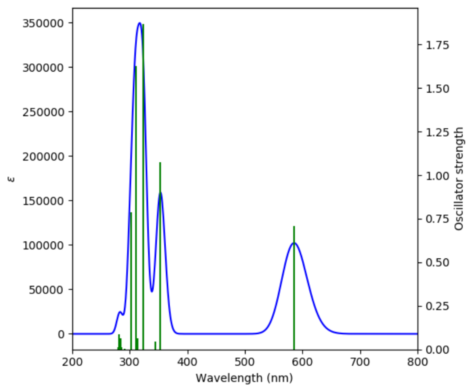

No.	Energy (cm⁻¹)	λ (nm)	Osc. Strength	Major Contributions
1	17074.7572	585.6598	0.7060	HOMO->LUMO (93%)
2	28288.2834	353.5032	1.0740	H-4->LUMO (21%), H-2->LUMO (61%)
3	28997.2448	344.8603	0.0446	H-2->L+2 (10%), HOMO->L+1 (22%), HOMO->L+3 (30%)
4	30990.2408	322.6822	1.8691	H-1->L+1 (44%), HOMO->L+3 (21%)
5	31902.4538	313.4555	0.0640	H-3->LUMO (18%), H-1->LUMO (53%)
6	32145.2267	311.0881	1.6268	H-18->LUMO (10%), H-2->L+1 (12%), HOMO->L+2 (34%)
7	33134.0624	301.8042	0.7862	H-18->LUMO (28%), H-10->LUMO (10%), H-2->L+1 (10%)
8	34376.1563	290.8993	0.0042	H-5->LUMO (11%), H-3->LUMO (25%), H-1->LUMO (27%)
9	34954.4558	286.0865	0.0123	H-16->LUMO (11%)
10	35138.3502	284.5893	0.0639	H-13->LUMO (13%)
11	35507.7521	281.6286	0.0876	H-18->LUMO (36%)
12	35725.5218	279.9119	0.0135	H-4->LUMO (12%), H-3->L+1 (13%), H-2->LUMO (10%), H-2->L+2 (16%)

Figure S72. Calculated emission spectrum and electronic transitions for **5** in DMSO.

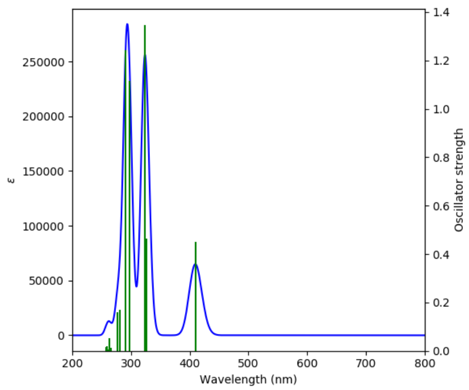

No.	Energy (cm^{-1})	λ (nm)	Osc. Strength	Major Contributions
1	24410.3697	409.6619	0.4491	H-2->LUMO (18%), H-1->LUMO (14%), HOMO->LUMO (55%)
2	30632.9372	326.4460	0.4651	H-1->LUMO (13%), H-1->L+2 (12%), HOMO->L+1 (43%)
3	30957.9786	323.0185	1.3475	H-4->LUMO (12%), H-2->LUMO (22%), H-1->LUMO (23%), HOMO->L+2 (14%)
4	33598.6378	297.6311	1.1151	H-1->L+1 (26%), HOMO->L+2 (28%)
5	34386.6415	290.8106	1.2434	H-2->L+1 (28%), HOMO->L+3 (28%)
6	35502.1063	281.6734	0.1685	H-3->LUMO (10%), H-2->LUMO (12%), H-1->LUMO (16%), HOMO->LUMO (34%)
7	36155.4153	276.5837	0.1600	H-15->LUMO (63%)
8	37636.2493	265.7012	0.0126	H-20->LUMO (13%)
9	37988.7136	263.2360	0.0527	H-2->L+2 (21%), HOMO->L+4 (16%)
10	38430.7054	260.2085	0.0054	-
11	38617.0195	258.9531	0.0196	HOMO->L+5 (16%)
12	38827.5302	257.5492	0.0143	H-9->LUMO (13%)

Figure S73. Calculated absorbance spectrum and electronic transitions for **6** in DMSO.

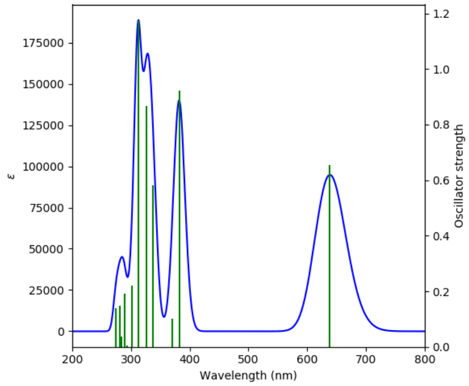

No.	Energy (cm^{-1})	λ (nm)	Osc. Strength	Major Contributions
1	15652.8018	638.8632	0.6550	HOMO->LUMO (94%)
2	26138.0093	382.5846	0.9220	H-4->LUMO (11%), H-2->LUMO (57%), HOMO->L+1 (12%)
3	26980.8587	370.6331	0.1024	H-2->LUMO (12%), HOMO->L+1 (42%), HOMO->L+3 (15%)
4	29653.7801	337.2251	0.5805	H-1->LUMO (28%), HOMO->L+2 (28%)
5	30603.9012	326.7557	0.8675	H-1->LUMO (27%), HOMO->L+2 (31%), HOMO->L+3 (13%)
6	32033.9222	312.1690	1.1737	H-1->L+1 (26%), H-1->L+3 (10%), HOMO->L+3 (14%)
7	33097.7675	302.1351	0.2203	H-16->LUMO (20%), H-9->LUMO (11%)
8	34097.8950	293.2732	0.0052	H-8->LUMO (14%), H-3->LUMO (23%), H-1->LUMO (11%)
9	34636.6733	288.7113	0.1924	H-4->LUMO (21%), H-1->L+2 (11%)
10	35156.9010	284.4391	0.0368	H-16->LUMO (11%), H-14->LUMO (16%), H-3->LUMO (10%)
11	35587.6010	280.9967	0.1468	H-16->LUMO (25%), H-3->LUMO (18%)
12	36457.8732	274.2891	0.1377	H-2->L+2 (12%), H-1->L+3 (13%), HOMO->L+5 (14%)

Figure S74. Calculated emission spectrum and electronic transitions for **6** in DMSO.

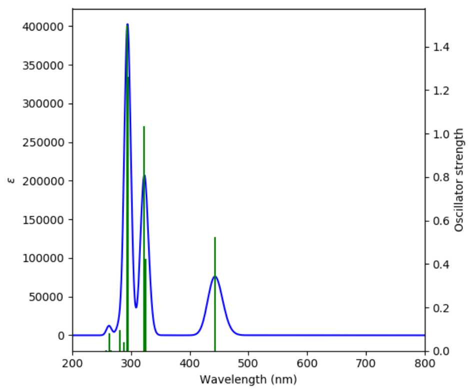

No.	Energy (cm^{-1})	λ (nm)	Osc. Strength	Major Contributions
1	22566.5863	443.1330	0.5257	H-1->LUMO (23%), HOMO->LUMO (71%)
2	30790.2153	324.7785	0.4226	H-2->L+2 (16%), H-1->L+1 (19%), HOMO->L+1 (40%)
3	31051.5389	322.0452	1.0331	H-4->LUMO (22%), H-2->LUMO (51%)
4	33963.2004	294.4363	1.2606	H-1->L+1 (30%), HOMO->L+3 (40%)
5	34031.7575	293.8431	1.5007	H-4->LUMO (10%), H-2->L+1 (29%), HOMO->L+2 (35%)
6	34773.7876	287.5729	0.0399	H-1->LUMO (49%), HOMO->LUMO (24%)
7	35664.2237	280.3930	0.0946	H-15->LUMO (52%)
8	37313.6275	267.9986	0.0004	H-10->LUMO (17%), H-6->LUMO (26%)
9	37643.5083	265.6500	0.0010	H-1->L+2 (20%), HOMO->L+4 (15%)
10	38004.8446	263.1243	0.0016	H-3->L+1 (14%), H-2->L+2 (17%), H-1->L+3 (16%), HOMO->L+5 (20%)
11	38084.6935	262.5726	0.0823	H-15->LUMO (13%), H-9->LUMO (13%), H-7->LUMO (31%)
12	38802.5270	257.7151	0.0013	H-5->L+1 (12%), H-1->L+8 (16%), HOMO->L+6 (12%)

Figure S75. Calculated absorbance spectrum and electronic transitions for **7** in DMSO.

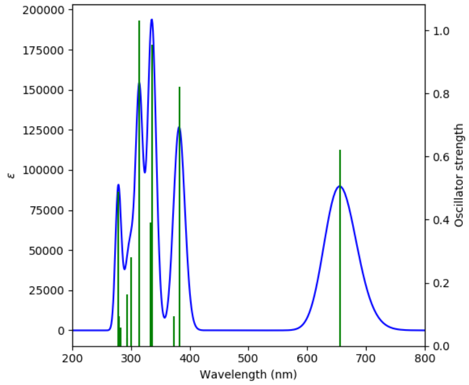

No.	Energy (cm^{-1})	λ (nm)	Osc. Strength	Major Contributions
1	15255.9770	655.4807	0.6204	HOMO->LUMO (95%)
2	26135.5897	382.6200	0.8218	H-4->LUMO (17%), H-2->LUMO (72%)
3	26825.1937	372.7838	0.0928	HOMO->L+1 (47%), HOMO->L+3 (27%)
4	29745.7273	336.1827	0.9540	HOMO->L+2 (67%)
5	29952.2052	333.8652	0.3922	H-1->LUMO (59%)
6	31820.1853	314.2659	1.0319	H-1->LUMO (11%), H-1->L+1 (35%), H-1->L+3 (12%), HOMO->L+3 (11%)
7	33234.0752	300.8959	0.2813	H-16->LUMO (29%), H-9->LUMO (12%)
8	34089.8294	293.3426	0.0245	H-11->LUMO (30%), H-3->LUMO (29%), H-1->LUMO (15%)
9	34138.2227	292.9267	0.1642	H-9->LUMO (29%), H-4->LUMO (23%)
10	35446.4540	282.1156	0.0585	H-15->LUMO (13%), H-3->LUMO (18%), HOMO->L+1 (11%), HOMO->L+5 (14%)
11	35768.2692	279.5774	0.0952	H-16->LUMO (34%), H-9->LUMO (13%), H-4->LUMO (13%)
12	35970.7144	278.0039	0.4874	H-4->LUMO (19%), H-2->LUMO (11%), H-2->L+1 (11%), H-1->L+2 (11%), HOMO->L+4 (14%)

Figure S76. Calculated emission spectrum and electronic transitions for **7** in DMSO.

Molecular Orbitals (MOs)

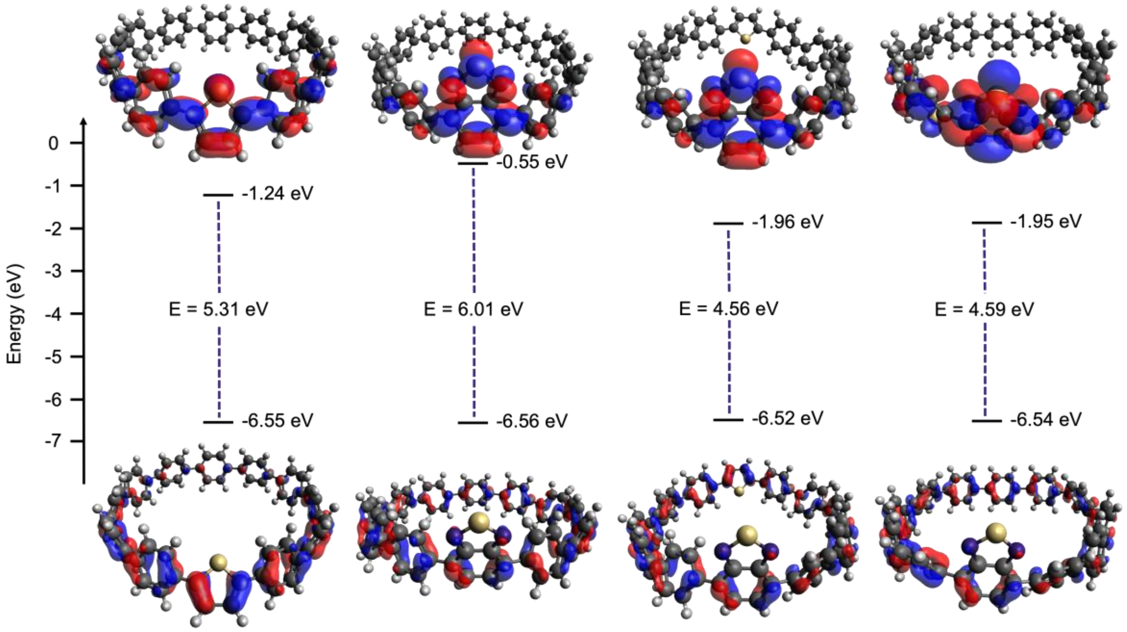

Figure S77. HOMO and $S_{1'}$ of **3**, **1**, **5**, and **4** using a CAM-B3LYP function, a 6-31G** basis set, and the solvent being dichloromethane.

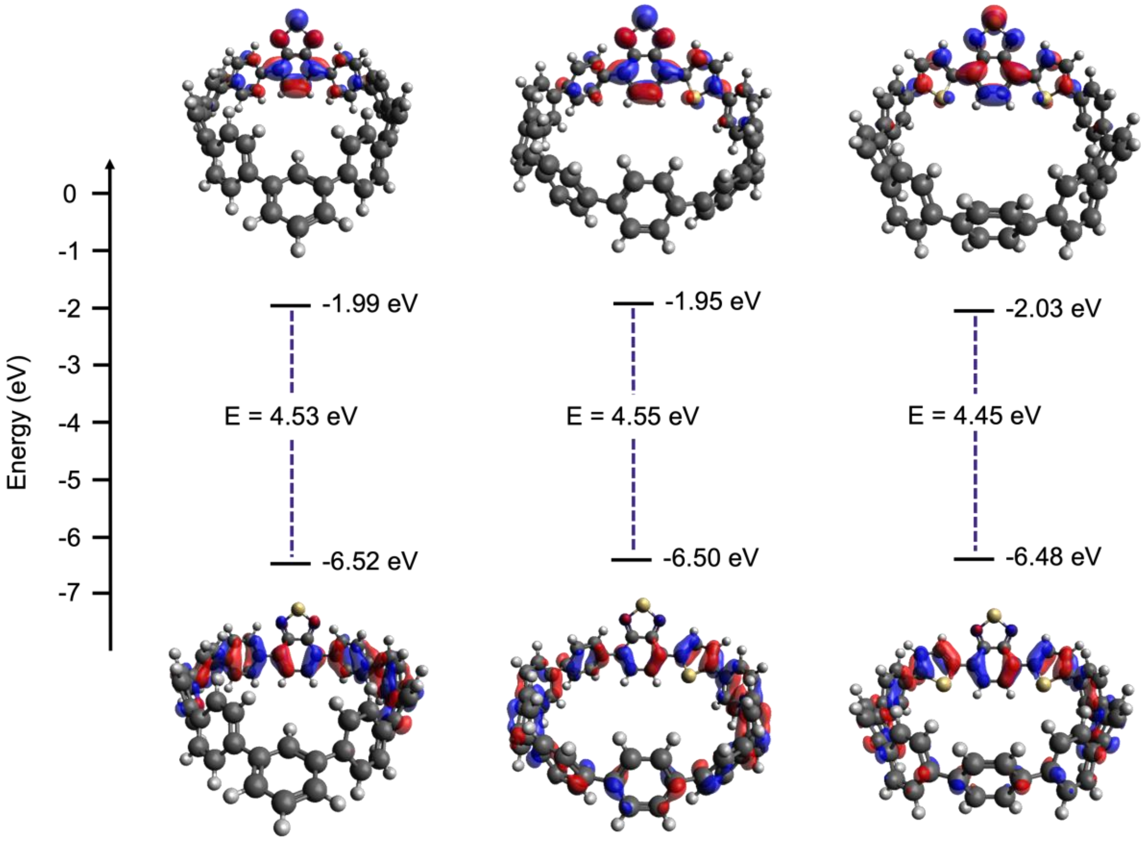

Figure S78. HOMO and $S_{1'}$ of **2**, **6**, and **7** using a CAM-B3LYP function, a 6-31G** basis set, and the solvent being dichloromethane.

5. StrainViz Calculations

All calculations were performed using Gaussian 09. The optimized ground state geometries used to calculate the predicted UV-vis spectra in DCM, were used to prepare the .xyz files of **2**, **7**, **BT[10]CPP**, and **BT-6-thio[10]CPP** and their corresponding fragments. StrainViz calculations were visualized in VMD.

Outputs from individual StrainViz calculations for **2**, **BT[10]CPP**, and **BT-6-thio[10]CPP** were re-submitted with the maximum bond energy set to that of [8]CPP to generate a relative strain scale. This setting re-colors each bond to generate StrainViz structures that are now comparative. In the "bond_scripts.py" file, line 171 "max(norm_values/31" was replaced with the max bond strain value in the "total_bond.tcl" file for [8]CPP. Values in the "total_bond.tcl" file are in kcal mol^{-1} and therefore need to be converted to Hartrees before being inputted in the "bond_scripts.py" file.

Attempts to use the highest strained bond between the three molecules yielded incorrect coloring in the StrainViz heat map (e.g., whole molecule was green). Using the maximum bond energy for [8]CPP was found to re-color the structures analogous to the initial, individual StrainViz calculations. The ground state geometry for [8]CPP, necessary to make the .xyz files, was obtained by using a CAM-B3LYP function, the 6-31G** basis set, and the effect of the solvent was included by the polarizable continuum model (PCM) using dichloromethane.

Fragment geometries are shown below:

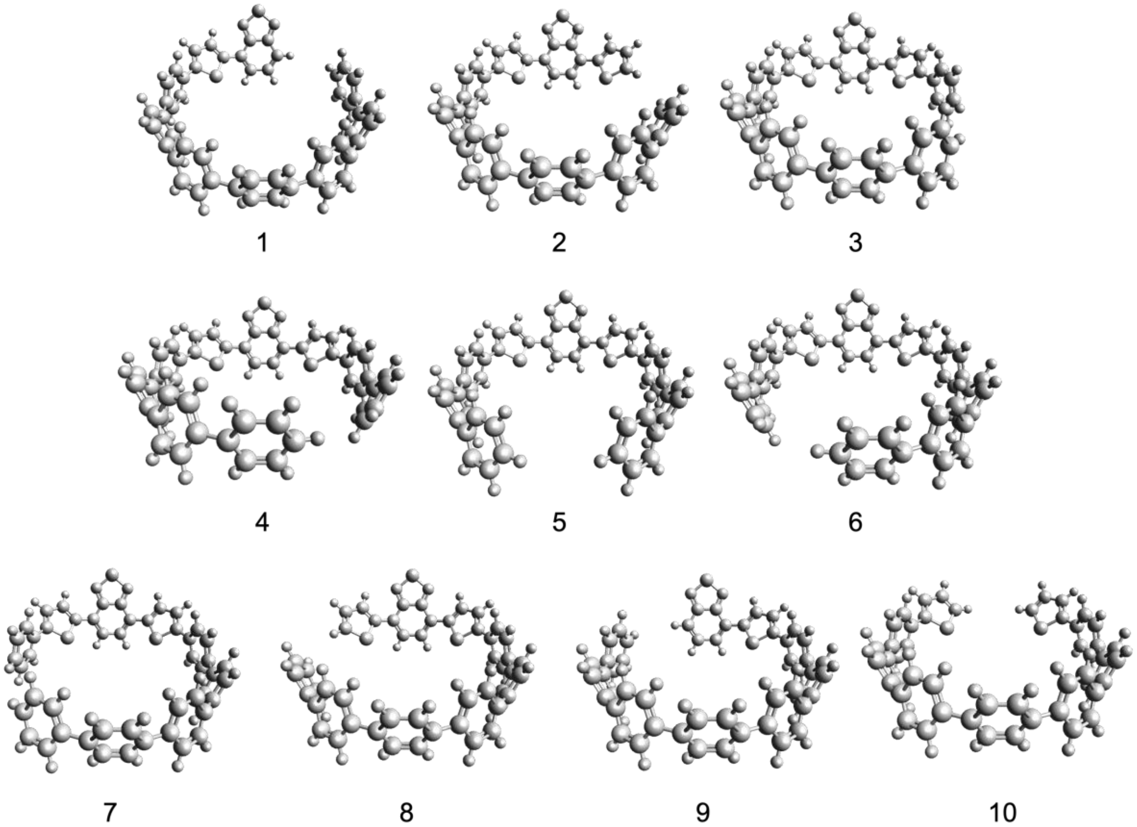

Figure S79. Fragments of **7** for use in StrainViz.

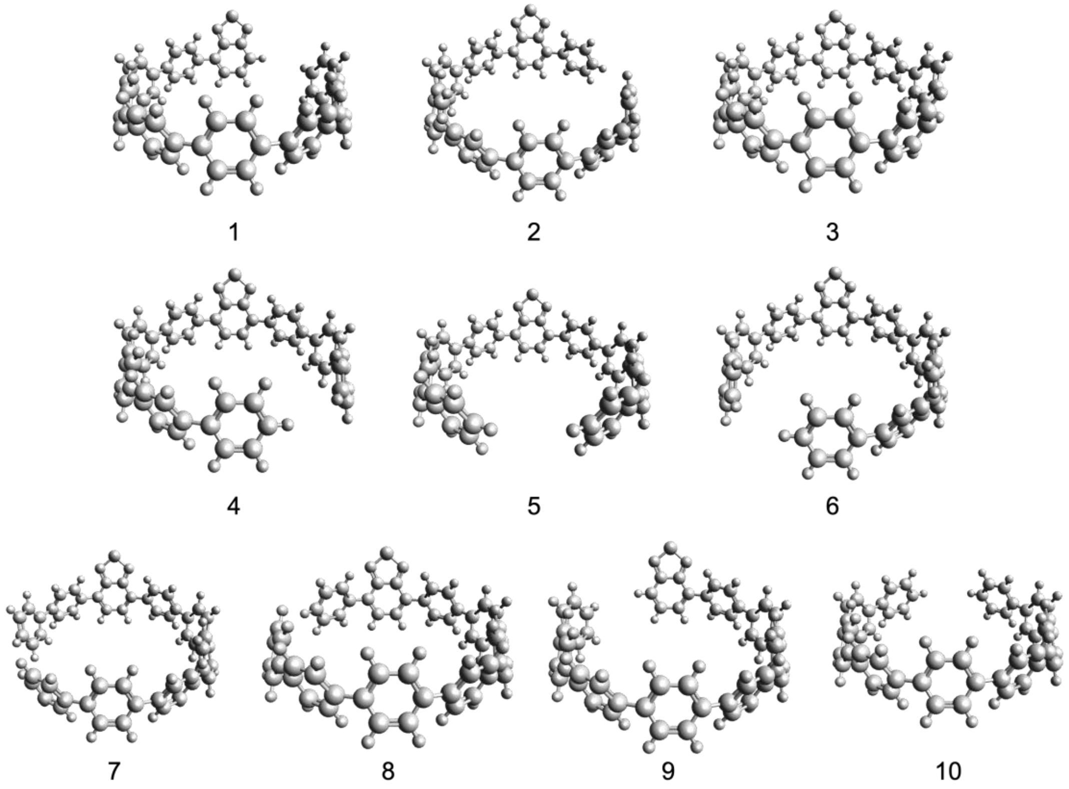

Figure S80. Fragments of **BT[10]CPP** for use in StrainViz.

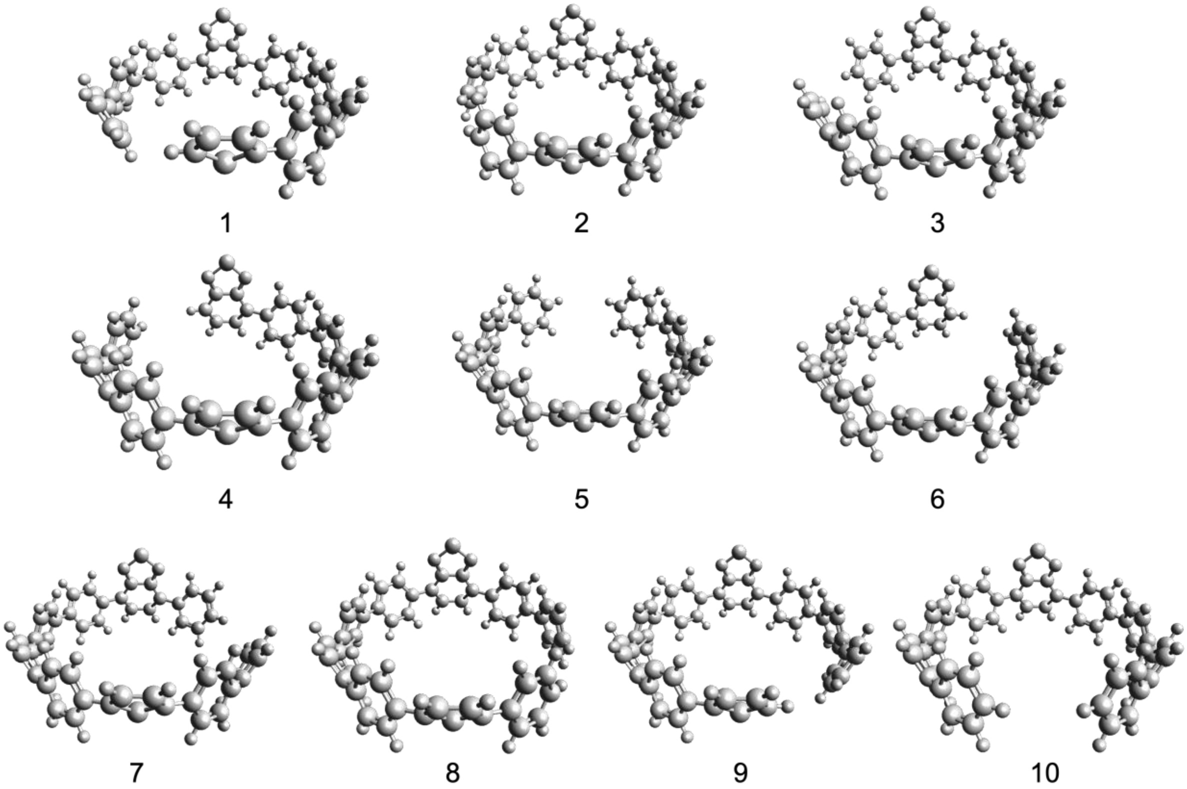

Figure S81. Fragments for **BT-6-thio[10]CPP** for use in StrainViz.

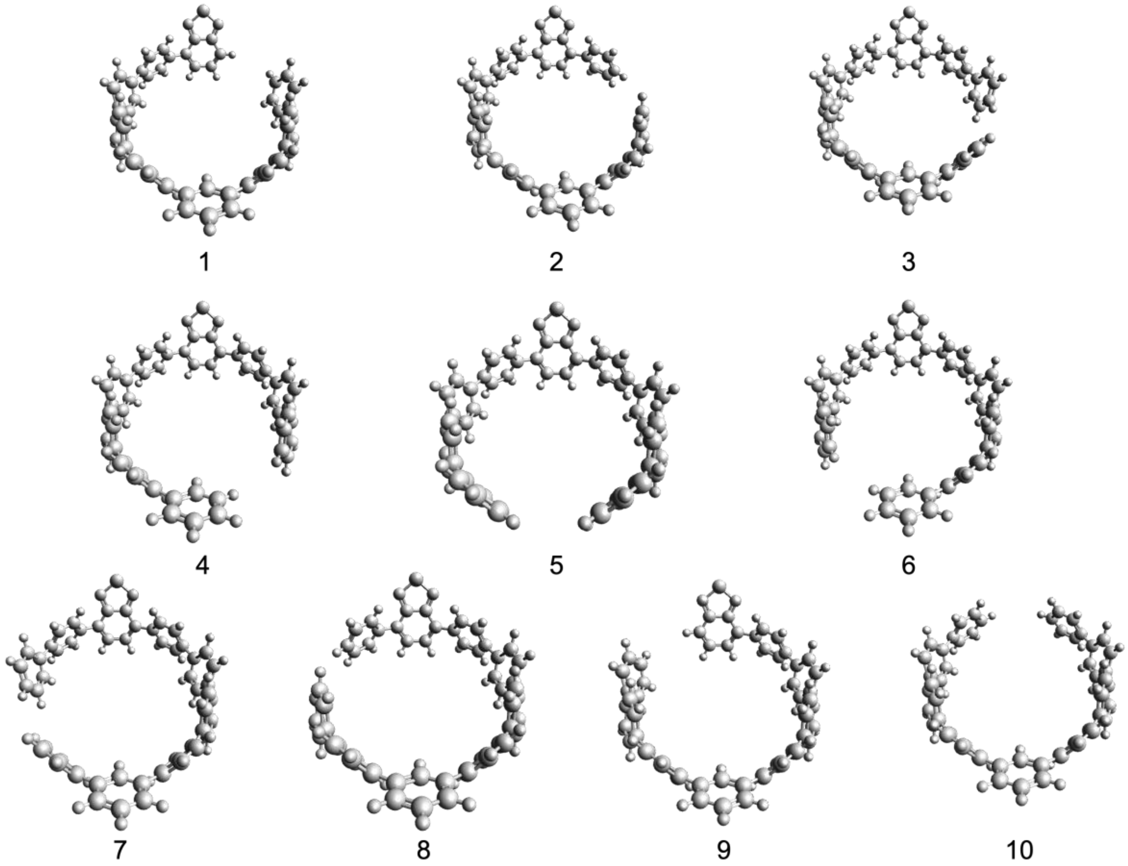

Figure S82. Fragments of **2** for use in StrainViz.

6. X-Ray Crystallography

Single crystals of **7** were grown by slow evaporation of DCM and diethyl ether at room temperature.

X-ray crystallography. Diffraction intensities for **7** were collected at 173 K on Bruker Apex2 diffractometer using CuKα radiation, 1.54178 Å. Space group was determined based on systematic absences. Absorption correction was applied by SADABS.[13] Structure was solved by direct methods and Fourier techniques and refined on F^2 using full matrix least-squares procedures. All non-H atoms were refined with anisotropic thermal parameters. H atoms in the structure were refined in calculated positions in a rigid group model. X-ray diffraction from crystals at high angles was very weak due to a high disorder of solvent molecules inside the structure. Even by using a strong X-ray micro focus source it was possible to collect diffraction data only up to $2\theta_{max}$ = 99.73°. However, the data collected provide appropriate number of measured reflections per refined parameters, 4993 per 550. The main molecules form columns in the crystal structure. Solvent molecules (CH_2Cl_2 and Et_2O were used) fill out a space inside these columns sharing the same positions. Their positions are highly disordered or partially occupied. These solvent molecules were not resolved and were treated by SQUEEZE.[14] They are not included in the formula of the compound given in the CIF file. All calculations were performed by the Bruker SHELXL-2014/7 package.[15]

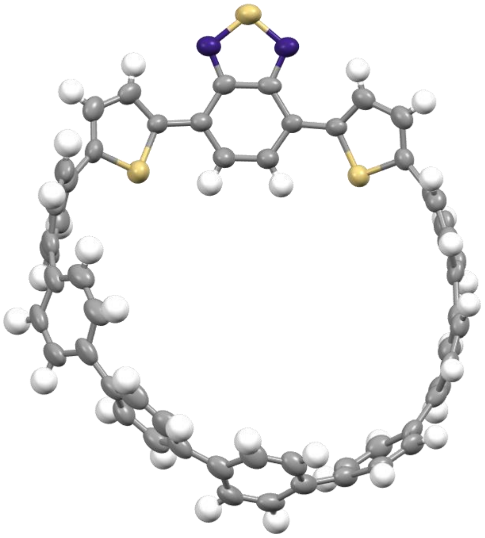

Crystallographic Data for **7** *(CCDC 2550070)*: $C_{56}H_{34}N_2S_3$, M = 831.03, 0.14 x 0.10 x 0.02 mm, T = 173(2) K, Monoclinic, space group *P*2$_1$/*n*, *a* = 9.6588(6) Å, *b* = 28.3941(18) Å, *c* = 10.0364(10) Å, β = 98.163(5)°, *V* = 4896.4(5) Å^3, *Z* = 4, Z' = 1, *D*c = 1.127 Mg/m^3, μ(Cu) = 1.659 mm^{-1}, F(000) = 1728, $2\theta_{max}$ = 99.73°, 17025 reflections, 4993 independent reflections [R_{int} = 0.0820], R1 = 0.0504, wR2 = 0.0993 and GOF = 1.041 for 4993 reflections (550 parameters) with I>2σ(I), R1 = 0.0883, wR2 = 0.1092 and GOF = 1.041 for all reflections, max/min residual electron density +0.174/-0.264 eÅ^{-3}.

7. References

1. T.C. Lovell, C. E. Colwell, L. N. Zakharov, and R. Jasti, *Chem. Sci.* 2019, **10**, 3786–3790.
2. J. M. Fehr, N. Myrthil, A. L. Garrison, T. W. Price, S. A. Lopez, and R. Jasti, *Chem. Sci.* 2023, **14**, 2839–2848.
3. E. R. Darzi, B. M. White, L. K. Loventhal, L. N. Zakharov, and R. Jasti, *J. Am. Chem. Soc.* 2017, **139**, 3106–3114.
4. Sigma-Aldrich (Spectral data were obtained from Advanced Chemistry Development, Inc.
5. N. C. Bruno, M. T. Tudge, and S. L. Buchwald. Chem. Sci., 2013, 4, 916–920.
6. X. Li, L. Liu, L. Jia, Z. Lian, J. He, S. Guo, Y. Wang, X. Chen, and H. Jiang, *Nat Commun* 2025, **16**, 467
7. T. C. Lovell, T. Z. R. Garrison, and R. Jasti, *Angew. Chem. Int. Ed.* 2020, **59**, 14363–14367.
8. A. B. Pangborn, M. A. Giardello, R. H. Grubbs, R. K. Rosen, and F. J. Timmers. Organometallics, 1996, 15, 1518–1520.
9. Horiba Scientific: A Guide to Recording Fluorescence Quantum Yields. https://static.horiba.com/fileadmin/Horiba/Application/Materials/Material_Research/Quantum_Dots/quantumyieldstrad.pdf.
10. N. Mataga, Y. Kaifu, and M. Koizumi, *Bull. Chem. Soc. Jpn.* 1955, **28**, 690-691.
11. S. Canola, L. Mardegan, G. Bergamini, M. Villa, A. Acocella, M. Zangoli, L. Ravotto, S. A. Vinogradov, F. Di Maria, P. Ceroni, and F. Negri, *Photochem. Photobiol. Sci.* 2019, **18**, 2180-2190.
12. R. Yoshida, T. Tachikawa, and S. Ito, *Cryst. Growth Des.* 2022, **22**, 547-558.
13. G. M. Sheldrick, *Bruker/Siemens Area Detector Absorption Correction Program*, Bruker AXS, Madison, WI, 1998.
14. P. Van der Sluis and A. L. Spek, *Acta Cryst., Sect. A* 1990, **A46**, 194-201.
15. G. M. Sheldrick, *Acta Cryst.* 2015 **C71**, 3-8.
